# Aggregating Demand for Three Fundamental Resources to Avoid Burden-Shifting in Climate Policy

**DOI:** 10.1021/acs.est.5c12742

**Published:** 2026-03-26

**Authors:** Jennifer L. Hawkin, Julian M. Allwood

**Affiliations:** Department of Engineering, 2152 University of Cambridge, Trumpington Street, Cambridge CB2 1PZ, U.K.

**Keywords:** climate-change policy, climate-change mitigation, net zero, biomass, energy supply, carbon storage

## Abstract

Most proposals for mitigating climate change assume that economic demand should grow without constraints so depend primarily on technology innovations to substitute today’s activities with emissions-free alternatives. However, the potential for such “invisible technology substitutions”, which could allow high-resource lifestyles to continue unchanged, is often overstated and disguised by burden-shifting. For example, plans may depend on synthetic fuels without accounting for its supply, or on negative emissions technologies without accounting for their power or land area requirements. Here, we show that all net-zero plans depend fundamentally on three resources: emissions-free electricity, biomass, and carbon storage. Using a comprehensive calculator, we reveal the high risk of shortages of these fundamental resources by comparing aggregated demands of net-zero plans, published by business, government, and industry bodies, against likely global availability in 2050. The calculator builds on physical models of 170 processes derived from an extensive literature search. Our results demonstrate that most climate policy proposals, which depend primarily on “invisible technology substitutions”, require an improbable expansion of the fundamental resources in the time available, indicating significant risks of under-delivery. We demonstrate an alternative mitigation plan built on a credible forecast of resource availability, revealing overlooked opportunities for innovations in policy, service supply, and financing: feasible zero-emission futures necessitate end-user participation and changed economic demand, which are largely disregarded in current international policy discussions.

## Graphical abstract

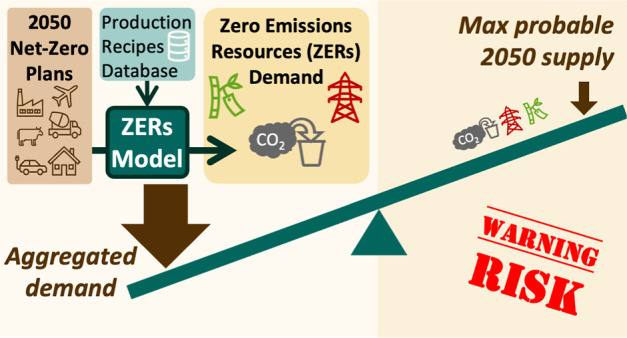

## Introduction

Ambitions to eliminate greenhouse gas emissions are widely shared. As of 2024, around 90% of global emissions were represented in national net-zero plans, while around 50% of the world’s largest 2000 companies included net-zero in their corporate strategy targets,^1^ with the targets linked to corporate, sectoral, and national strategies of varying robustness.^2^ Most mitigation strategies rely primarily on novel technology, substituting the use of fossil-fuels with “green” energy, for example, in electric cars, using hydrogen for steel reduction, or capturing and storing cement emissions, in the hope of limiting requirements for changed behavior, culture, or provisioning systems. This technology-led approach began in the early 1970s with the IPAT equation suggesting that the environmental impacts of increasing population and consumption could be offset by technological advances.^3^ It has been reinforced repeatedly by economic assessments such as the Stern review^4^ ^,^ which portray technology as the main lever of change, advocating for policy around innovation support, in anticipation of future economies of scale.

The prioritization of novel technologies in climate policy is supported by the widespread use of Integrated Assessment Models (IAMs) in policy planning. Some of these models aim to guide overarching policy goals through cost-benefit analysis of the total costs of mitigation against the costs of climate damage.^5^ Specific interventions are evaluated in “cost-effectiveness” models in which aggregated energy demands are derived as continuations of historical trends in efficiency and economic development,^6^ while deployment rates are derived as back-casts from a target emissions trajectory subject to specified economic and socio-political constraints.^7^ These models assume marginal changes from the current economic system, with impacts anticipated as adjustments to a notional equilibrium. This is inconsistent with the deep social, technological, physical and economic transitions required for decarbonization,^8^ and the complexity of interactions between them.^9^

Cost-effectiveness IAMs often reach solutions by allowing improbable access to technologies, particularly negative emissions technologies. CCS costs, for example, are commonly underestimated.^147^ Limits on annual carbon sequestration, meanwhile, are only constrained to around 2.5–17.5 GtCO_2_/year,^10^ significantly higher than could be available by 2040 (0.95–4.3 GtCO_2_/year), based on optimistic analysis of maximum feasible deployment rates.^11^ Such assumptions around deployment rates deny the realities of physical and supply chain operations^12,13^ and the socio-institutional barriers to technology deployment.^13–15^ In reality, the complex construction projects required to deliver energy infrastructure are often “over-time and over-budget”, with long decision-making processes required before construction can be commenced.^16^ Research in recent years has demonstrated that it is possible to improve the representation of technology growth rates within IAM modeling, for example, using historical analogues to represent Direct Air Capture (DAC),^17^ but such approaches are not commonly used within the modeling community.

Yet, despite continued optimism about novel technology substitutions expressed in sectoral or national climate policies, global emissions have risen 60% since the UN Framework Convention on Climate Change was signed by 197 countries in 1992.^18^ A fundamental problem is that with current modeling, political and corporate leaders can announce ambitious plans and claim progress, while in reality, shifting the burden of mitigation elsewhere to justify high consumption pathways to net-zero. Burden-shifting may occur across time, national boundaries, or corporate boundaries. This is revealed by the promises of climate repair and negative emissions technologies,^19^ by the expectations of exponential growth in energy infrastructure—which contrast with evidence of slow and predictable past transitions^20^ —by the difference between production and consumption accounts of emissions,^21^ and by corporates planning to use emissions-free hydrogen but not to make it. Carbon offsets similarly enable emitting organizations to buy emissions reductions elsewhere, exemplifying burden-shifting. This creates mitigation failures today,^22,23^ unless offsets are additional, verifiable, immediate, and durable.^24^ It has been argued that the resulting level of dependence on uncertain negative emissions in some emissions-reduction pathways may contravene international law.^25^ Where there is capacity for burden-shifting within modeling frameworks, mitigation plans and policies will be ineffective. This creates substantial but, as yet, unrecognized risks of mitigation failure, which can be countered only by examining a broader diversity of climate policies.

## Materials and Methods

In order to expose burden-shifting and reveal more credible pathways to mitigation, a different form of modeling is required: an approach to anticipate consolidated supply and demand for physical resources which are disguised in models based on monetary metrics. A suitable model must be global (to avoid burden-shifting across national, corporate, and sectoral boundaries), holistic (to avoid burden-shifting among intermediate energy-carriers by tracing physical demands back to fundamental resources), and temporal (to avoid burden-shifting in time). At first sight, this looks daunting due to the myriad final and intermediate goods required to sustain today’s familiar services. However, by tracing back the dependencies of the technologies specified in climate plans, we have observed that all climate policies depend on three fundamental zero-emission resources (ZERs): emissions-free electricity, carbon storage, and biomass. While these ZERs may themselves produce emissions and demand energy through their lifecycle, we simplify the model by excluding these additional demands for resources. Figure 1 illustrates this dependence, showing, for example, how plans to decarbonize aviation using electric planes, hydrogen, biofuel, or negative emissions technologies, in turn, depend on these three fundamental resources. Burden-shifting can therefore be revealed by aggregating the demands of a global climate policy package for the three ZERs.

**Figure 1:**
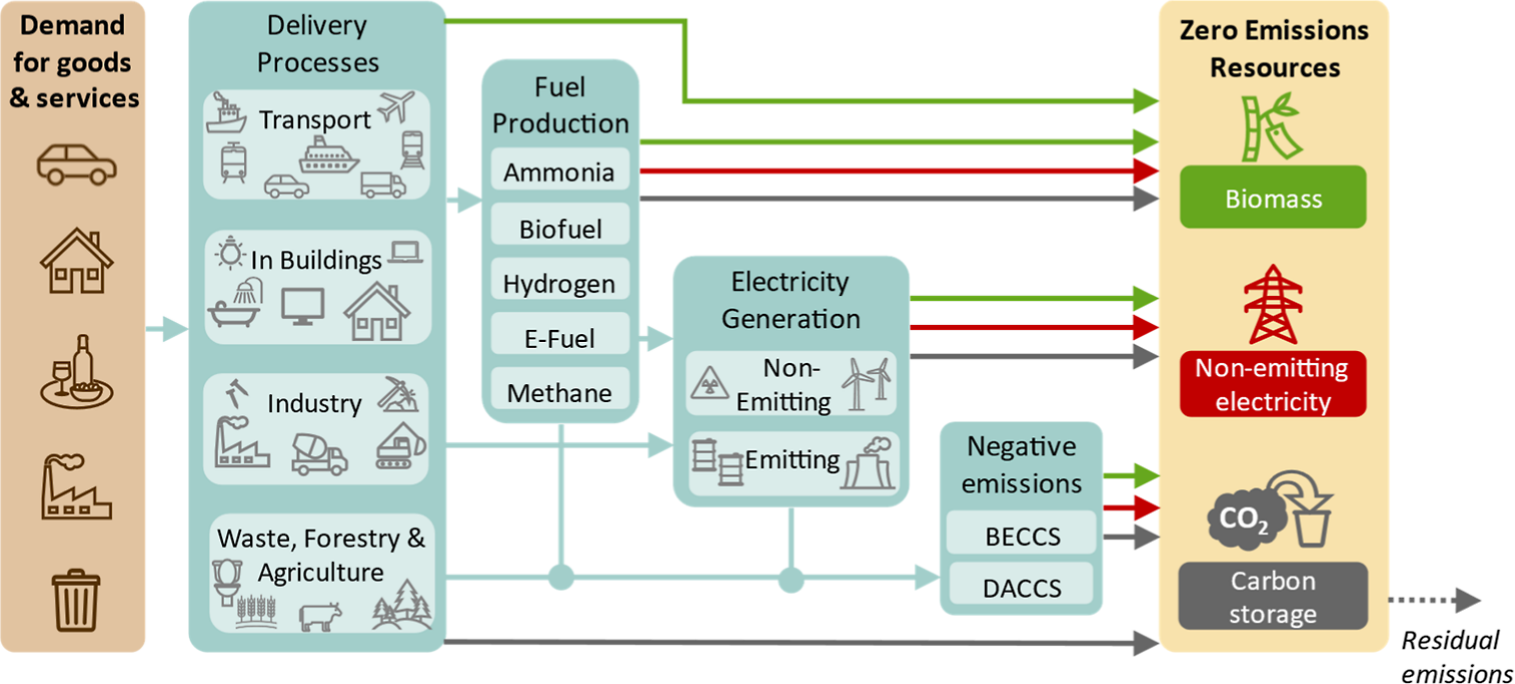
Tracing supply chains of “net-zero” physical production back to the energy resources required to meet global demand for goods and services demonstrates that all climate mitigation plans depend on three zero-emission resources (ZERs): biomass, emissions-free electricity, and carbon storage. The arrows show demands for energy and materials (green for biomass, red for nonemitting electricity, gray for carbon storage, and light blue for intermediary demands). Zero-emission resources are derived by the model from the inputs (demands for goods and services) by quantifying these physical flows. Any residual emissions, which are not compensated by carbon storage, are shown by the dotted gray arrow. The model is generally solved to make this flow zero, i.e., to represent net-zero scenarios. The figure is a conceptual representation only.

We construct a calculator based on Figure 1 to trace global demand for final goods and services back to demand for the three fundamental zero-emission resources (ZERs) and allow comparison with achievable supply. Our study is distinct from previous bottom-up analyses, such as ref 26, not only by our use of the ZERs framing but also because we take a global view (to avoid possible burden-shifting across countries) and we directly evaluate the reality of widely discussed “net-zero strategies” from industry and politicians. The model builds on the maths of Inventory Analysis in Life-Cycle Analysis,^27^ using linear algebra to sum the resource demands required to deliver end-user goods and services. The extensive literature search required to identify the coefficients of an initial basket of 170 processes supplying 45 goods and services is described in Part 5 (Document 2) of the Supporting Information file.

### Model Mathematical Framework

The calculator (outlined in Figure 2) uses linear algebra to sum the resource demands required to meet forecast demand for end-user goods and services. Within the system, a set of N flows (internal and final substances and services) is produced or removed by N activities, configured such that each of the N flows is the primary “output” of one activity but may also be a byproduct of others. In addition to the internal and final resources, the system may draw on external stocks and deposit resources externally. In the current model, there are 45 activities and internal flows, listed in Table 1, and three external flows—non-emitting electricity, biomass, and stored carbon dioxide—which would need to be supplied externally to the system to meet the annual demand calculated by the model. Additional activities and flows could be added to the model, as described in the Supporting Information Part 2.3.

**Figure 2:**
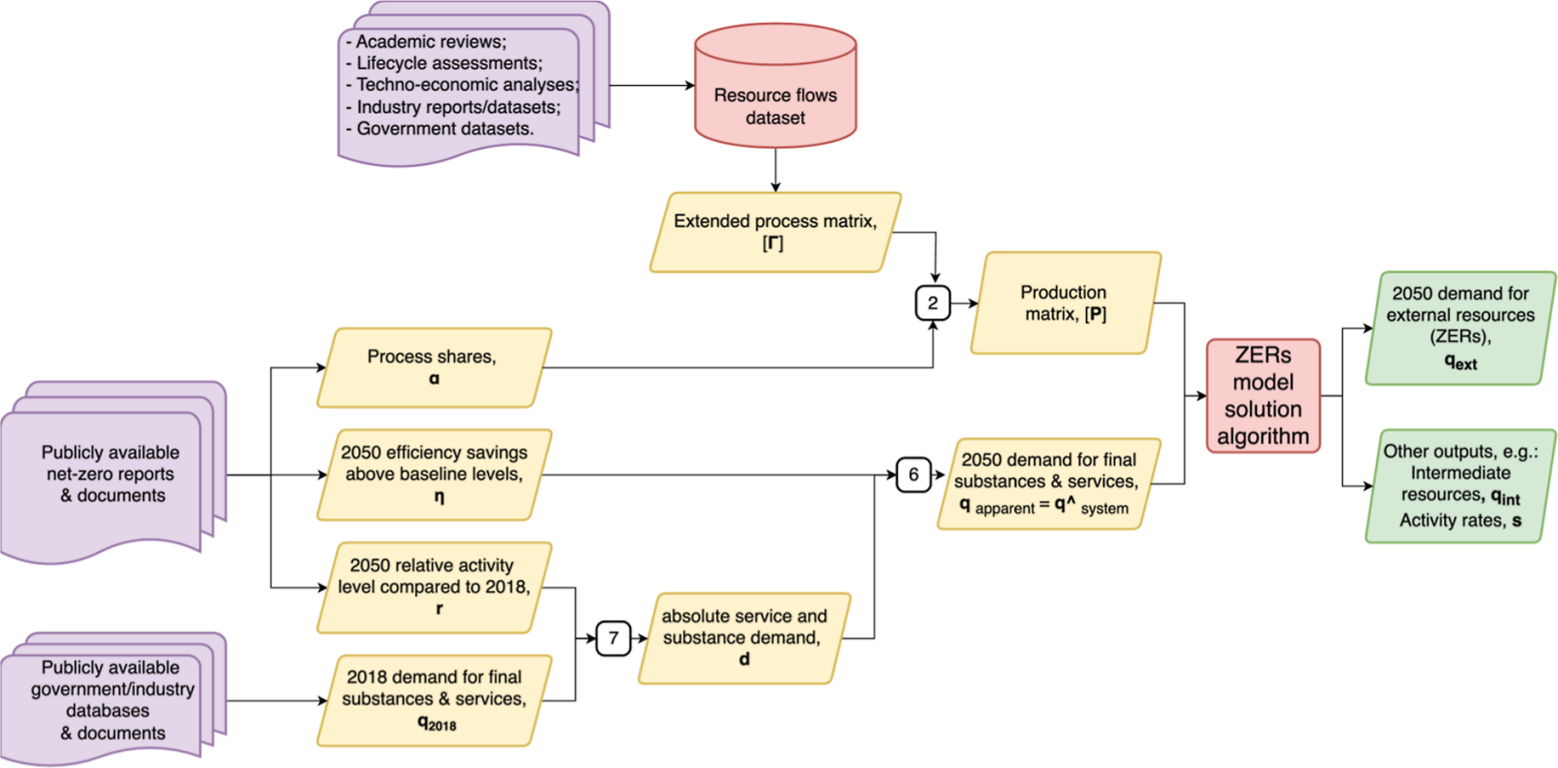
Diagram of the ZERs model information flows. The definitions of model terms are given in Table 2. Numbers given in process boxes refer to the equation numbers in the text. The ZERs solution algorithm is given in Box 1. The resource flows data set is described in the “model coefficients” section of the methods.

**Table 1:** Internal Activities and Their Primary Flows Quantified in the Current Version of the ZERs Model^a^

sector	activity	associated primary flow and unit of measurement
agriculture and forests	farming food	raw food, ×10^15^ kcal
	forestry	wood, Gt
	plant agriculture	other plant biomass, Gt

buildings	cooking	cooking energy, EJ
	cooling the built environment	energy for cooling, EJ
	lighting	energy for lighting, EJ
	space heating	space heating, EJ
	use of appliances	energy for appliances, EJ
	water heating	water heating, EJ

electricity	distribution of electricity	distributed electricity, EJ
	electricity generation	electricity at point of generation, EJ

fuels and feedstocks	ammonia production	ammonia, Gt
	biofuel production	biofuel, Gt
	HVCs production	high-value chemicals, Gt
	hydrogen production	hydrogen, Gt
	methane production	methane, Gt
	methanol production	methanol, Gt
	oil processing and refining	oil, Gt
	plastics production	plastics, Gt
	production of other petrochemical products, not accounted for elsewhere	other petrochemical products, Gt
	synfuel production	synfuel, Gt
	urea production	urea, Gt

industry	aluminum production	aluminum, Gt
	cement production	cement, Gt
	construction	cement and steel used in construction, Gt
	food processing	“on-the-shelf” food, ×10^15^ kcal
	glass production	glass, Gt
	paper production	paper, Gt
	product manufacturing and other industrial processes	products, Gt
	steel production	steel, Gt
	textiles production	textiles, Gt

mining	coal mining	coal, Gt
	minerals and metals mining	minerals and metal ores, Gt
	oil and gas extraction	raw oil and gas, Gt

NETs	residual emissions management (negative emissions tech.)	atmospheric carbon dioxide, Gt
	management of carbon dioxide gas	captured carbon dioxide, Gt

transport	aviation	aviation, ×10^12^pkm
	bus transportation	bus transportation, ×10^12^pkm
	car transportation	car transportation, ×10^12^ car-km
	passenger rail transportation	passenger rail transportation, ×10^12^pkm
	rail freight	rail freight, ×10^12^tkm
	road freight	road freight, ×10^12^tkm
	shipping	shipping, ×10^12^tkm

waste	municipal solid waste management	municipal solid waste, Gt
	wastewater treatment	wastewater, Gt

a kcal = kilo-calories, Gt = gigatonnes, EJ = exajoules, pkm = passenger-kilometer, tkm = tonne-kilometer.

All activities within the system are represented by the production matrix (P), represented in Figure S5 (Supporting Information Section 2.1), where each column of [P] describes a “recipe” to transform one set of substances or services into another. The vector q describes the flows of physical substances and services created by anthropogenic processes, each running at the required rates defined by an activity rate vector, *s*. This representation, building on Inventory Analysis (outlined by Chapter 2 of ref 27), leads to eq 1.

1 q=[P]s

Equation 1 shows that net flows of services and materials (*q*) are the sum of those produced, minus those consumed, by all processes operating at the rates given by *s*. Equation 1 is written as if each flow is the main output of a single process, where in many cases several process options are combined. Each column (*j*) of [P] is therefore derived as a single weighted averaged process to provide service or substance, j so that all potential delivery processes (e.g., a battery electric vehicle or a hydrogen fuel cell EV (HFCEV)) may be considered. If there are *M*_j_ modes of provision for service j, where each mode, i, has a modal share, α_ij_, describing the proportion in which each operate, then the weighted averaged process (the activity) recipe can be found using eq 2.

2 $$P_{j}=\sum_{i=1}^{M_{j}}\Gamma_{j}^{i}\alpha_{ij}=\left[\Gamma_{j}\right]\alpha_{j}$$

This gives the values for columns of [*P*], where [Γ_j_] is the extended process matrix, containing the separate recipes for all the M_j_ unique modes to provide service or substance, j. The sum of modal shares for any given service is exactly one (eq 3).

3 $$\sum_{i=1}^{M_{j}}\left(\alpha_{ij}\right)=1\;\mathrm{for}\;\mathrm{all}\;j$$

The rows of [*P*] and *q* are partitioned vertically into submatrices to distinguish internal flows (in *q*_system_—the known demand for end-user goods and services) and external flows (in *q*_ext_—the unknown demand for ZERs), leading to eq 4 (refer to Section A.5 of ref 27 for an explanation of partitioned matrices)

4 $$\left\{\begin{array}{l} \frac{q_{system}}{q_{ext}} \end{array}\right\}=\left[\begin{array}{l} \frac{P_{system}}{P_{ext}} \end{array}\right]s$$

The model is solved to find the demand for the three ZERs within the *q*_ext_ block of the *q* vector.

Within this model, activities are either production processes (marked by a + sign) or “waste management” processes (marked by a minus sign −), and the flow may be “final” (e.g., car transportation supplied to directly society) or “intermediate” (e.g., oil, required only for downstream processes, such as transportation). Partitioning the submatrices further into these groupings leads to eq 5

5 $$\left\{\begin{array}{l} \frac{\begin{array}{l} q_{final}^{+}\\ q_{final}^{-}\\ q_{\mathrm{int}} \end{array}}{\begin{array}{l} q_{ext}^{+}\\ q_{ext}^{-} \end{array}} \end{array}\right\}=\left[\begin{array}{l} \frac{\begin{array}{l} P_{final}^{+}\\ P_{final}^{-}\\ P_{\mathrm{int}} \end{array}}{\begin{array}{l} P_{ext}^{+}\\ P_{ext}^{-} \end{array}} \end{array}\right]s$$

Each entry in *q*_final_^+^ describes the flow of a physical substance or service created by an anthropogenic process, and each entry in *q*_final_^–^ describes the flow of an anthropogenic waste, removed by a waste management process. Intermediate flows in *q*_int_ may be either products or wastes, which are produced or consumed by other processes. Values for *q*_final_ are found from net-zero proposals (as described below), while for each intermediate flow, *i*, the demanded value, ${\mathrm{q̂}}_{i}$ , is 0.

Three conditions created by model input choices may cause physically meaningless solutions to eq 5. They are controlled as follows.

- For scenarios without NETs, residual emissions must be determined by the rates of all emitting processes. NetEmissions is moved from an internal substance to an external substance, and the relevant column is removed from P.
- Some combinations of delivery processes can imply impossible “circularity” and are therefore unsolvable. For example, this would occur if all methane were produced synthetically from hydrogen and carbon dioxide, while all hydrogen is produced from methane. In this case, increasing the rate of production of methane increases the demand for methane since it is also demanded for its own feedstock. Impossible process combinations can be identified when any off-diagonal element of the Hadamard product of *P*_system_ and its transpose ≥1. Before solving the model, this condition is therefore tested and the user is prompted to alter the delivery process shares.
- The presence of multifunctional processes can produce negative *s* values when solving eq 4, to compensate for overproduction of a byproduct flow. For example, generating electricity (primary flow) from biomass incineration with CCS could in theory produce more negative emissions (the byproduct) than are required to meet net-zero. The process for negative emissions would then require negative *s* for eq 4 to hold. This would indicate the process runs in reverse, a physical impossibility. In these cases, an iterative approach is used, which removes the constraint on net production of the overproduced resource. The associated primary activity is removed from the system, and the resource flow is moved to *q*_ext_^+^, as an addition to external stocks. Similarly, a waste management activity could be a cobenefit of another activity and lead to managing more waste than existing in the internal system. We therefore allow such activities to manage (assumed unlimited) residual stocks of waste as an entry in *q*_ext_^–^.

The total solution algorithm is given in Box 1, using the notation detailed in Table 2. A circumflex ( ^) is used to indicate the user-demanded flows (in contrast to the model calculated flows).

**Table 2:** Definitions of Terms Used in Describing the Mathematical Framework^a^

term	dimension	definition used within this work
*d*	N × 1	demand vector—the absolute service and substance demand
*P*	(N + 3) × N	production matrix of quantified activities (also activity matrix)
*q* (composed of *q*_int_, *q*_final_, and *q*_ext_)	(N + 3) × 1	the annual production (flow) of substances and services (internal, final, and external substances and services, respectively)
*q*_2018_	N × 1	the net annual production (flow) of substances and services in 2018
*q*_apparent_	N × 1	the apparent annual production (flow) of substances and services, which would be supplied at baseline levels of efficiency (those determined by Γ). Values for comparable flows will be lower than *q*_final_ where additional efficiency measures are included in a net-zero plan
*r*	N × 1	relative change vector: the relative activity delivered compared to 2018 (i.e., 100% where there is no change)
*s*	N × 1	activity rates—the production rate of each activity, which is required in a given year to meet the demands, defined by the flows in *q*
α	M × 1	process share (or modal share)—the share of activity provided by each delivery process
Γ	(N + 3) × M	extended process matrix (composed of vectors quantifying flows for all individual delivery processes to provide all services and substances)
η	N × 1	efficiency savings vector: a percentage improvement against the baseline efficiency in Γ
N	1 × 1	the number of quantified flows in the model, excluding the three ZERs. This is also the number of activities represented by the model
M	1 × 1	the number of all quantified delivery processes (all different ways of producing all activities) in the model

a A plus sign (+) in the superscript of a model variable indicates a production flow and a minus sign (−), a “waste management” flow. A circumflex ( ^) above a variable indicates that this is a model input (the desired quantity of in-year flow to be delivered).

Box 1 – Model solution algorithm.

1. Assemble the P matrix from Γ and α, following eq 2.
2. Does the scenario include NETs? If not, move NetEmissions from an internal substance to q_ext_ and P_ext_, and remove the relevant column from P.
3. Check for circularity: is any *P*_systemij_ *P*_systemji_ > 1? If so, adjust model inputs.
4. If det­(*P*_system_) ≠ 0, find s by solving $\left\{\begin{array}{c} {\mathrm{q̂}}_{final}^{+}\\ {\mathrm{q̂}}_{final}^{-}\\ 0 \end{array}\right\}=\left[\begin{array}{c} P_{final}^{+}\\ P_{final}^{-}\\ P_{\mathrm{int}} \end{array}\right]\cdot s$ , where ${\mathrm{q̂}}_{final}^{+}\geq 0$

  Else, the program was terminated and an error message.
5. Test whether any element of *s* is negative. If not, output the solution and the end.
6. If so, iteratively:
  a. Remove activities corresponding to negative s from the matrix *P*_system_ (i.e., remove a column), transfer the associated flow to q_ext_ by moving the relevant row from *P*_system_ to *P*_ext_.
  b. Calculate a new set of values for *s*, and calculate the external flows from {*q*_ext_} = [*P*_ext_]·*s*
  c. Check that all original system flows (including those that were moved to *q*_ext_) are sufficient to meet the demands set by the scenario (i.e., sufficient goods are produced to satisfy needs, and all wastes are “managed”):
    i.
$\left\{\begin{array}{c} q_{final}^{+}\\ q_{\mathrm{int}}^{+} \end{array}\right\}\geq \left\{\begin{array}{c} {\mathrm{q̂}}_{final}^{+}\\ 0 \end{array}\right\}$

  ii.
$\left\{\begin{array}{c} q_{final}^{-}\\ q_{\mathrm{int}}^{-} \end{array}\right\}\leq \left\{\begin{array}{c} {\mathrm{q̂}}_{final}^{-}\\ 0 \end{array}\right\}$

d. If not, reintroduce the relevant flow to the internal system and resolve.
e. Repeat the iteration until all entries in *s* are non-negative and *q* are sufficient (i.e., meet the conditions in (c)).

### Model Coefficients

Table S425 (in Supporting Information 6.8) gives the complete list of activities and processes, which are quantified for each sector. The processes are represented as a set of normalized key input and output flows (Figure 3). These coefficients have been found by an extensive literature search, extracting data and information from peer-reviewed academic papers (e.g.,^28–33^ ), industry, consultancy, and policy reports (e.g.,^34–37^ ), and industry international or national databases (e.g.,^38–41^ ) are documented in Supporting Information Part 5.

**Figure 3:**
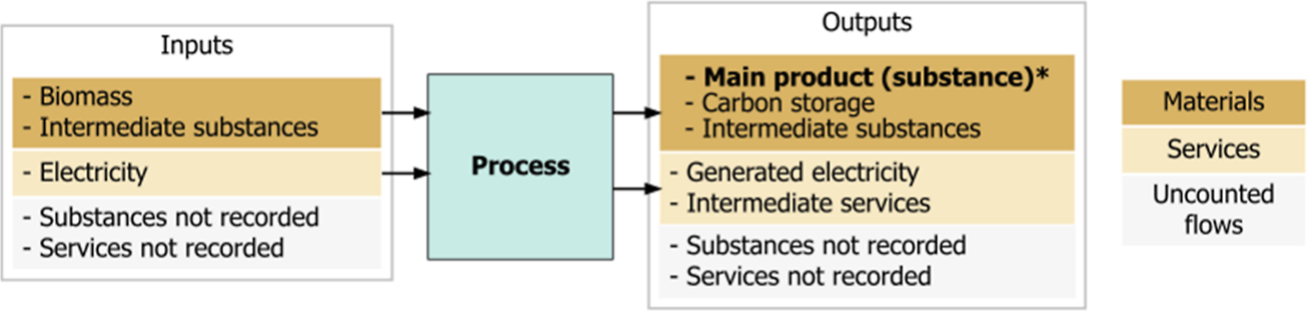
A generic flow diagram, used to quantify all process flows in the model. The example shown is a material production process (such as steel production or biofuel production). Each process in the model is characterized as a linear mapping between inputs and outputs. Only material flows associated with energy consumption (fuels and bulk material flows) or emissions production (including process emissions) are accounted for (in dark brown); if all material flows were accounted for (such as water consumption), each process could be mass balanced. The intended product or service of each process (the functional flow) is labeled in bold, marked by an *.

Coefficients for activities which aggregate a wide variety of products or subactivities, or which include complex process routes, are calculated as top-down estimations of energy consumption and emissions production of the entire sector. Examples of these activities are production of textiles and chemical products and food processing. All other coefficients are calculated from the physical processes involved in providing the service, using predictions of feasible implementation at scale by 2050, or examples of best practice design.

Coefficients were chosen using the following key assumptions.

1. It is assumed that each process will be feasible and available in sufficient quantity to meet the entire demand, i.e., capacity constraints are not considered.
2. Beyond energy, biomass, and emission vectors, interlinkages between activities are not included: for instance, the infrastructure (and related impacts) required for a given service is not accounted for explicitly.
3. Transport of materials and fuels is not explicitly considered.
4. Emissions of individual gases are accounted as CO_2_e based on GWP100.
5. It is assumed that carbon in emissions from biomass is mostly balanced by carbon sequestration in growing biomass within the same year, except for:
a. The production of methane (from waste and agricultural processes).
b. Biogenic carbon transferred to permanent storage (as for bio-energy with carbon capture and storage, BECCS).
c. Biogenic carbon in fuels and feedstocks which could be made from a combination of biogenic and fossil feedstocks.
6. Land-use emissions and sequestration are not explicitly accounted: increased sinks from regeneration and afforestation efforts are assumed to balance any remaining deforestation and degradation emissions. To put this assumption in deeper context, a rigorous expert elicitation study (ref 42) indicates sufficient scientific knowledge to support an accounting system for a global potential of around 10 Gt CO_2_e/yr reduction or sequestration from forest-related activities. This estimate is not time-bound and assumes only 50 years’ stability. Net Land Use Land-Use Change and Forestry emissions, meanwhile, are currently approximately 4 Gt CO_2_e/yr.^43^
7. The only forms of Negative Emissions Technology included are DAC and Storage (DACCS) or Bio-Energy with CCS (BECCCS) in geological formations because deployment of other forms at scale, including Nature-Based Climate Solutions (MBCS) beyond those in assumption (6), is not probable. A review of approaches is included in the Supporting Information Part 1.4.
8. Temporal variation in supply or demand is not considered (neither seasonally nor rapid fluctuations).

The impact of uncertainty in the model coefficients is explored using Monte Carlo analysis of 1000 runs. All coefficients (except functional flows) are assumed to have normally distributed uncertainty with variance of 10%, following guidance for LCI process data without uncertainty information.^44^

### Efficiency Measures

Efficiency measures described in net-zero plans are accounted for in the calculator by using an efficiency vector, η. The absolute values for desired rates of final substance and service flows, ${\mathrm{q̂}}_{system}$ , are replaced in eq 4 by the *apparent* activity rates, *q*_(apparent)_, composed of a demand vector, *d*, premultiplied by an efficiency matrix, (*I*_j_-diag­(η)).

6 $$q_{\left(apparent\right)}=\left(I_{j}-diag\left(\eta \right)\right)d$$

The demand vector (*d*) is estimated as the relative change compared to the 2018 levels, as shown in eq 7. This is defined by a vector of relative changes for each activity (*r*). *q*_2018_ defines the end-user services delivered in 2018.

7 $$d=diag\left(r\right)q_{2018}$$

### Policy Packages and Model Inputs

The values of the final activity flows, *q*_(apparent),final_, are provided in Supporting Information Part 6 for each policy package, derived from estimates of η and r from scouring documents of net-zero plans (given in Supporting Information Part 6). The packages are intended to represent dominant approaches to net-zero from government and industry, as follows.

- The “2050 Industry Accumulated Demands” policy package inputs are intended to represent the demand implied by current corporate strategies, using inputs identified from global industry group or consultancy reports (e.g., refs 45–47 ), where possible, or regional, national, or individual company reports (e.g., ref 48) or ref 49 where no other sources are found. Sources used are listed in Table 3.
- The CCS, Electrification, and Biomass Dominant packages are each given to demonstrate changing technology choices without changing demand. The delivery process shares for each approach are determined manually (described in Supporting Information Section 6).
- The “IEA Net-Zero Energy by 2050” (“IEANZE”) policy package is intended to represent the prominent IEA scenario of that name.^49^ Inputs are estimated directly from the original report^49^ or its update,^50^ other IEA reports and online data,^34,51^ or other industry sources (e.g., refs 52 and 53 ) as required.
- The UK Government Strategy policy package is intended to represent the approach being taken by the UK government (and other similar countries with strong climate commitments). Inputs are estimated from policy strategy documents, supplemented by research from the UK’s Climate Change Committee (e.g., refs 54 and 55 ).

**Table 3:** Sources Used to Assign the End-User Demands (*q̂*_final_) and the Process Shares (α) for All Mitigation Options for the “2050 Industry Accumulated Demands” Policy Package

sources used to quantify 2050 demands	activity model inputs based on those sources
^47,48^	oil and gas extraction
^57^	coal extraction
^58^	food processing, farming, forestry
^59^	methanol and HVC production
^60,61^	ammonia and urea production
^62^	plastic production
^63^	other petrochemical production
^64^	concrete production
^46^	steel production
^65^	aluminum production
^66^	glass production
^51^	paper production
^67^	textiles production
^68^	construction
^53^	aviation
^69^	road freight
^45^	shipping
^70^	waste management
^49^	appliances, cooking, cooling, lighting, space heating, water heating, bus use, car use, rail freight, passenger rail

An additional policy package is derived iteratively from the ZERs model to show a contrasting approach to climate mitigation: the low-ZER demand package. This package prioritizes electrification and participatory options (such as diet change) over other delivery processes. The example aims to stimulate innovation in mitigation strategies, which draw on resource efficiency and sufficiency strategies highlighted by ref 56. Final activity rates in low-ZER demand package are not based on value judgments but are derived by scaling back from today’s rates, based on each activity’s demand for the three ZERs. Activities which demand carbon storage are scaled down until aggregated demand is within estimated 2050 supply, and the approach is repeated for the other two ZERs (details in the Supporting Information Section 6.7). The final activity rates for the CCS, Biomass, Electrification, and UK Policy Packages, however, are the same as the 2050 Accumulated Industry Demands run. Tabular comparisons between the demands and delivery-processes for each package are available in the Supporting Information, Sections 6.2 and 6.3.

### Probable Availability of ZERs

Aggregated demands estimated using the ZER model are compared against “maximum probable” supply for the three ZERs in 2050, where probable supply describes the likely future availability. The “maximum probable” supply trajectories are derived from historical data, drawing on deployment rate literature to extrapolate forward. The approach is summarized here with further details and supporting evidence in the Supporting Information Part 3.

“Maximum probable” Emissions Free Electricity is based on trajectories for individual generation technologies (wind and solar, nuclear, hydropower, and geothermal generation). The forecast projects data of energy supply, not capacity, to facilitate comparison with demand. Historical data (1970–2022) for electricity generation from^71^ is projected forward linearly for nuclear,^72^ hydrothermal,^73^ and geothermal^74^ generation based on historical growth rates and industry reports assuming linear growth due to their established scale.^75^ The future trajectory of wind and solar generation to 2050 is based on a Gompertz model fit for global generation following the approach of ref 76, fitting s-curve growth models against national historical data. The trajectory in ref 76 is updated to account for unexpected growth of solar and wind generation in China since 2021.^77^

In the absence of a bottom-up analysis of probable carbon storage growth, as called for by ref 78, the projected capacity for carbon storage is based on an exponential fit to the capacity of historical and future projects,^79,80^ justified through consideration of trends in future regulation and investment,^80–82^ project completion delays and cancellations,^80,83^ and operational capacity factors,^84–87^ alongside growth constraints.^78,88,89^ The Supporting Information Part 3 uses historical and planned project data from the Global CCS Institute,^80,90^ combined with papers and gray literature, to justify this choice. The available CCS in 2050 has been assumed to be 70% of total capacity based on case studies that estimate current capacity factors of around 60% (Supporting Information 3.1.3, pS58).

Biomass is accounted in the model as the dry weight of plant biomass “used” by humans for food production, energy, and products. Livestock feed and food crop increases have dominated the historical growth of biomass extraction^91^ but have been attributed largely to a doubling of irrigated areas and increasing use of scientifically bred seeds and commercial fertilizer.^92^ Since cropland and pasture expansion cause land-use change emissions, this form of growth is incompatible with the ZER model. The only possible expansion of biomass consumption therefore is by increasing land-use intensity without net change in carbon stocks, and minimizing impacts on biodiversity. Only managed land is considered, consistent with bookkeeping approaches as used in IAMs used to derive the net-zero target.^93^ The “maximum probable” biomass availability in 2050 assumes linear growth between historical consumption in 1960 and 2010,^91^ followed by linear growth to a bottom-up estimate of 2018 ^38,39,94–97^ and maximum sustainable 2050 consumption, enabled though closing yield-gaps and increased residue-use.^98–102^ Details are given in Supporting Information 3.1.4.

Acknowledging that there is significant uncertainty in these “maximum probable” trajectories, a range of possible future supply is also evaluated in the Supporting Information Part 4.2.1, bound by a (more pessimistic) “lower risk” estimate and a (more optimistic) “maximum possible” estimate of 2050 ZER supply.

## Results and Discussion

For one global climate policy package (“2050 Industry Accumulated Demands”, as described in the Methods section), Figure 4 predicts a wide gap between forecast demand and maximum probable supply for the three ZERs, where probable supply describes the likely future availability. This reveals a high risk that these proposals will not deliver and will shift a greater burden of mitigation to future generations. Relying on the feasibility of such proposals will further delay the societal participation required to deliver the wider portfolio of mitigation options available by using today’s technologies differently, for example, through driving smaller, fuller cars or flying less.

**Figure 4:**
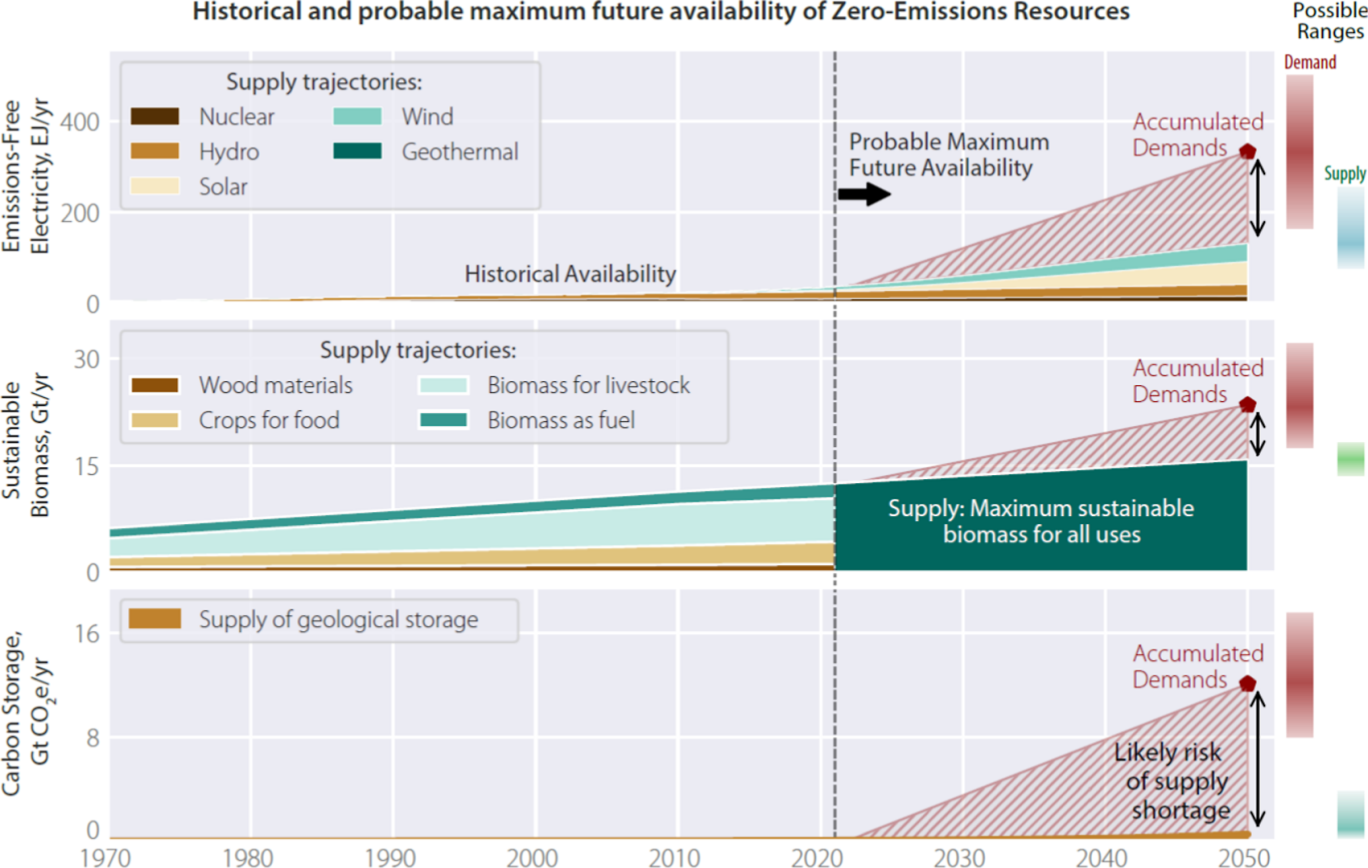
Comparison of global supply and demand for the three ZERs over time for a single climate policy package (“2050 Industry Accumulated Demands”, as described in the Methods section), revealing a substantial shortfall. The right-hand-side shows the possible ranges of 2050 demand and supply. The demand range considers variance of ±10% of the process coefficients (Methods). The supply range shows possible 2050 supply between a “lower-risk” and “maximum-possible” supply estimates (Methods). Anticipated demand and technology shares for goods and services with net zero-emissions come from prevailing corporate and political strategies, identified from global sector reports, where possible, and regional, national, or individual company reports, or ref 49, are used where no other sources are found (see Supporting Information Section 6). Supply trajectories are derived from historical data, projected forward based on analysis of trends in past and future deployment, regulation and investment (Methods).

Drawing on the same supply forecasts as Figure 4, Figure 5 (the main result of this paper) contrasts supply of the three zero-emission resources with the calculated demands required to deliver mitigation via six contrasting policy packages, allowing for uncertainty in process model coefficients. In every case, supply is far short of demand, so it is almost certain that these policy packages cannot deliver the mitigation they promise. This message is underlined by further results in the Supporting Information. Figure S22 shows that supply remains short of demand, even if the variance in model coefficients is expanded to ±30%. At ± 50% variance, demand for emissions-free electricity and biomass can be met in 10% of the scenarios but the supply of carbon storage is still insufficient. Even if the maximum probable supply is expanded ten times, only in 5% of the scenarios with 50% variance in model coefficients can supply of carbon storage meet demand.

**Figure 5:**
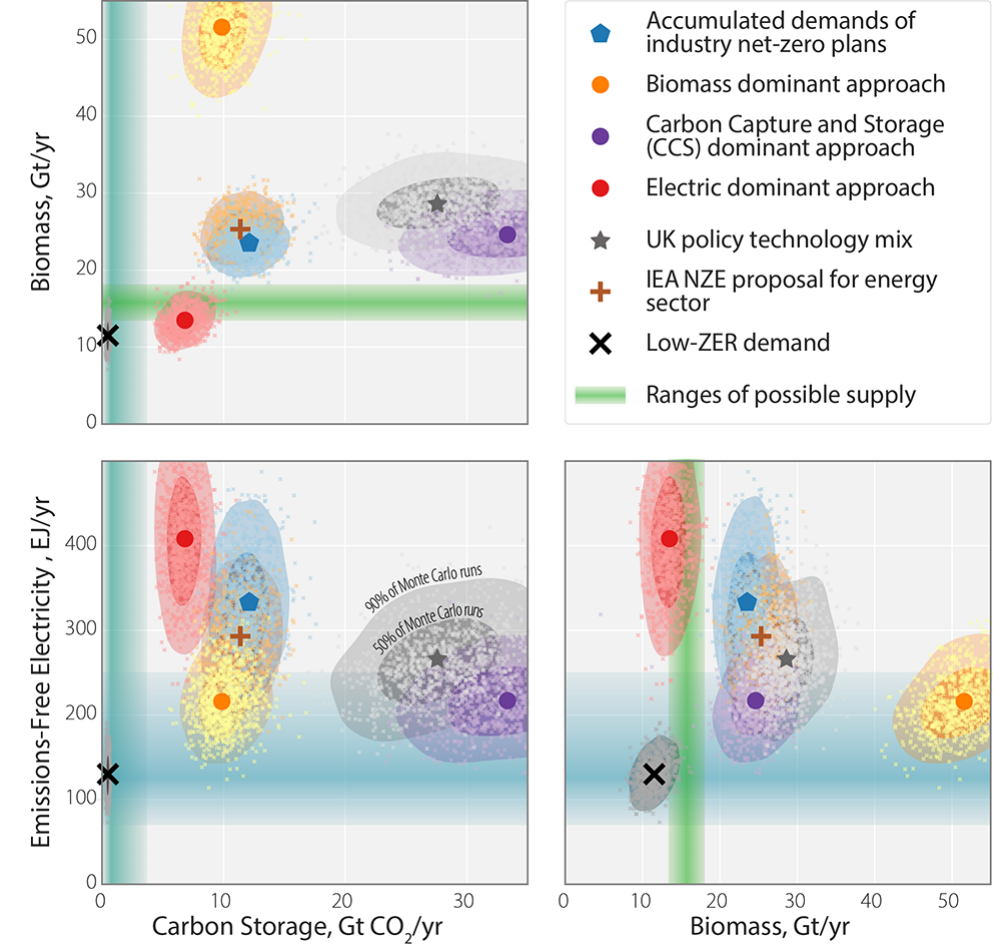
Supply and demand for the three ZERs, as predicted in Figure 2, contrasted against a portfolio of global mitigation policy packages (described in the Methods, “model inputs” section), and allowing for normally distributed uncertainty up to ±10% in all coefficients of delivery processes. On each subplot, the lower lefthand region beneath the shaded bands describes the probable supply. Six policy packages are shown to be outside this lower-risk region for all pairwise plots: The “2050 Industry Accumulated Demands” policy package assembles current prevailing corporate and industry strategies from publicly available reports; the “IEA Net-Zero Energy by 2050” (“IEANZE”) represents the International Energy Agency net-zero scenario;^49^ the CCS, Electrification, and Biomass Dominant Approaches prioritise substitution of each resource in turn, with the goal of delivering today’s services without user-awareness of change; and the UK Government Strategy is chosen to be representative of the government and policy programmes of similar countries with strong climate commitments using inputs from UK government and policy-advisory documents. The low-ZER demand example is shown in Figure 6, chosen to fall within the 2050 ZER constraints. A full list of the model inputs and sources is given in the Supporting Information Part 6. The relatively small uncertainty in biomass supply reflects the different constraints (and, therefore, the modeling approach used). Since emissions-free electricity and carbon storage are constrained by deployment rates, the uncertainty significantly increases over time (capacity increases cumulatively). Biomass, in contrast, is constrained by land capacity—which is fixed—constrained by ecological boundaries and the climate.

Carbon storage is the key constraint on delivering current policy packages and therefore the main mechanism of burden shifting. If it were deployed at scale, existing cement kilns, blast furnaces, and other processes could continue, new intermediate resources like blue hydrogen or ammonia could enable new solutions, and hard-to-abate emissions (ruminants, rice, aviation) could be mopped up by DAC. Without it, the vast majority of these options are removed. Only a small quantity of non-CO_2_ emissions could be allowable for climate change mitigation,^103^ and there is unlikely to be any excess supply of biomass to create a nonemitting substitute. As a result, the target of climate mitigation cannot be “net zero”, because without carbon storage there are no scalable, long-term negative emissions technologies. The target is instead “absolute zero”. While this is inconvenient for current politics and businesses, it is nevertheless true, and in the face of rapid acceleration in rates of global drought, it motivates the need for rapid and radical redesign of climate policy.

To illustrate the requirements for deliverable net-zero plans, Figure 6 contrasts the activity levels anticipated by the International Energy Agency^49^ with those predicted by the calculator when drawing on the maximum probable of supply for the three ZERs. The calculation assumes high electrification, 100% plant-based diets, and the most efficient end-use technologies, for example, delivering space-heating with heat pumps. With limited carbon storage, activities such as large-scale cement production are heavily reduced, and without significant carbon storage or additional biomass, aviation and shipping are also heavily constrained. On average, most end-use goods and services can be delivered at approximately one-third of the demands anticipated by “business as usual”. The balance of activities could be adjusted, increasing one while reducing others, provided the total demand for the three ZERs holds constant. The restraint implied by the figure is transient as it is an estimate for 2050 only, so for example, by 2100, it is possible that supplies of ZERs will be greater, and the resultant activities in Figure 6 can be expanded. Nevertheless, inconvenient or not, Figure 6 shows a credible description of the physical activities of a net-zero economy in 2050 and motivates policy change both to anticipate restraint and to prioritize intensification, to expand the delivery of services from a constrained set of physical activities. Given average cars in the UK, for example, are used for 4 h per week, with an average occupancy of 1.5 people, weighing 12 times less than the vehicle, the required intensification may be much less difficult than Figure 6 initially suggests.

**Figure 6:**
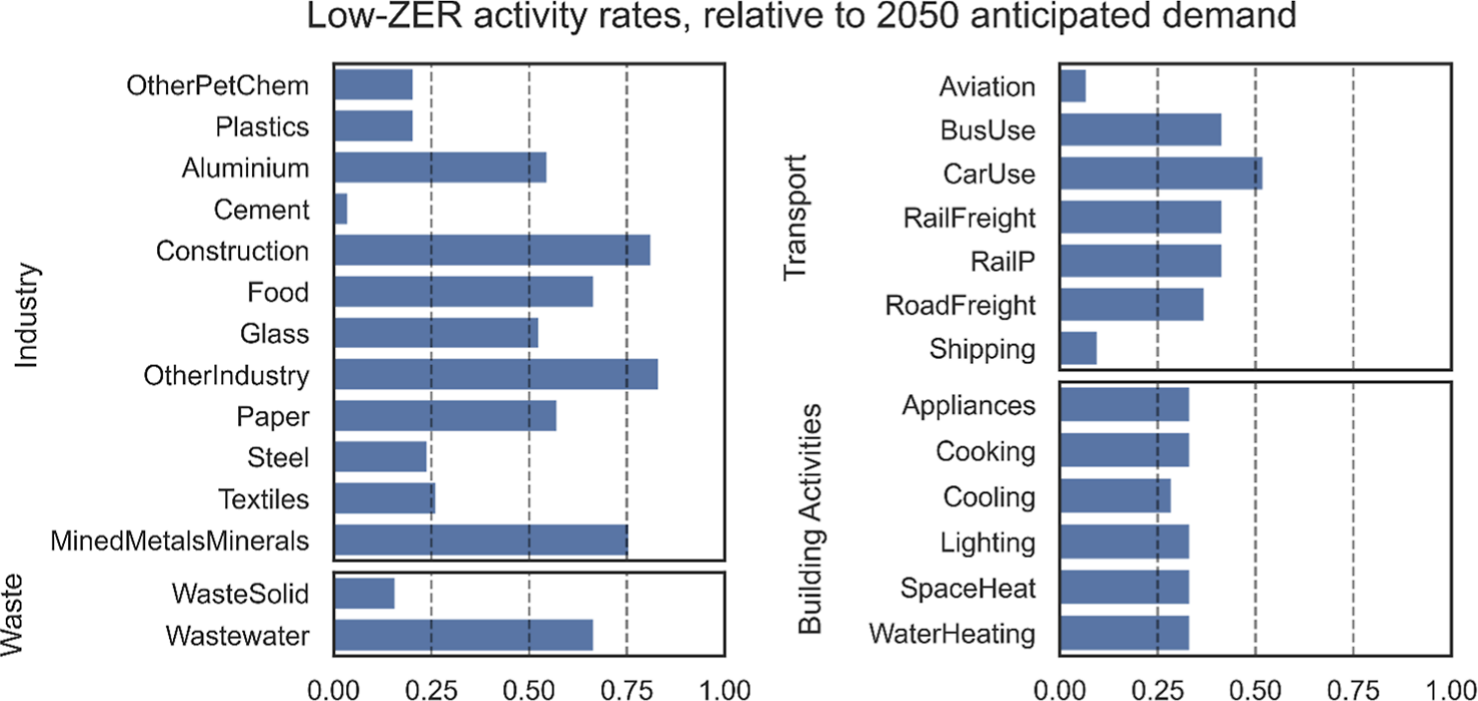
An example of a mitigation plan that can be delivered without burden shifting, within the constraints of available ZER supply (low-ZER demand in Figure 5). The calculator is used in reverse to deliver the maximum possible activity consistent with the maximum probable supplies of the resources as used in Figures 2 and 3. The resulting apparent activity rates are shown relative to the IEA’s Net Zero by 2050 scenario.^49^ Final activity rates in this scenario are not based on value judgements but are derived by scaling back from today’s rates, based on each activity’s demand for the three ZERs. Activities which demand carbon storage are scaled down until aggregated demand is within estimated 2050 supply, and the approach is repeated for the other two ZERs (details in the Supportion Information Section 6.7).

Are the supply forecasts in Figure 4 conservative? Economic models predicated on new large-scale infrastructure are consistently overoptimistic about future trajectories,^14,104–106^ and this is the key unmanaged blindspot of current policy. Past energy transitions have had long preparation phases, followed by apparently exponential growth up to around 5% of final deployment, and then proceeded largely at linear rates.^20,75,107^ This is because large energy infrastructure projects depend on a sequence of decisions, spanning the complex processes of political alignment, planning, finance, and tendering followed by construction, with each step requiring public consent and delivered by a constrained team of public servants.^108^ The gap between anticipated supply and demand in Figure 4 is widest for Carbon Storage, which in the 50 years since first deployment, has been subject to intense marketing, but actual installed capacity grew at a linear rate of 0.004% of global emissions per year for the past decade.^109^ Historical trends may underrepresent future availability, if the consistency of incentives and regulatory environments improve with the increasing urgency of climate change^110^ but storage and pipeline development will cause additional delays.^78,88^ As 70% of current capacity is used to enhance oil extraction, expansion to new applications will be complex, slow, and expensive,^78,111^ not least due to the need for public finance in both construction and operation. A final concern is that the industry publishes capacity not actual storage rates, which may be significantly lower,^84^ as discussed in the Supportion Information Section 3.1.3.

Future biomass supply for human use, meanwhile, depends on land use, crop yields, and the fraction of crop residuals diverted from current uses in nature. Deforestation, often driven by agricultural expansion, produces around 11% of total anthropogenic CO_2_ emissions (4Gt in 2020),^112^ so further increases in land-use should be avoided. Economic development may help to close global yield gaps^99^ but major crop-producing countries may be approaching biophysical limits,^113^ climate change will reduce yields^114^ and the fraction of global land experiencing drought is expanding rapidly.^115^ The supply forecasts in Figure 4 are therefore “maximum probable” and then are subject to significant delivery risk. They define upper limits on the resources that should be considered in climate policy.

Existing approaches to Net Zero, predicated on unnoticed substitution of emitting activities by “invisible technologies”, have high risks of failure because they require an improbable expansion in the supply of the three ZERs. Net-zero plans instead could embrace restraint and anticipate the participation of society in different uses of familiar technologies. In many cases, the supply shortfall in Figure 5 can be compensated by intensification, exemplified in Table 4 which spans the efficiency of provisioning, shifting demand to more resource-efficient provisioning, and avoiding demand.^56^ While “improve” strategies from Table 4 are becoming increasingly prominent in net-zero plans, “shift” and “avoid” approaches are particularly under-represented in climate policy. Such approaches could, however, offer energy and emissions reduction potentials of 40–70%^116^ while also stimulating new opportunities for entrepreneurship^117–119^ and well-being.^116,120,121^ Adopting such approaches depends on societal participation (rather than a top-down approach to mitigation predicated on public investment) and so motivates a switch to collaborative change, as happens in campaigns about public health and citizenship. Demand-side mitigations have attracted growing interest in research,^116,122–124^ increasing prominence in the most recent IPCC cycle^125^ and interest at citizens’ assemblies^126^ but are, as yet, under-represented in climate policy.^127–129^ Using the calculator in policy development could help to catalyze consideration of previously unexplored possibilities hidden by the rigid structures of current approaches to energy modeling.^130^

**Table 4:** Selected Examples of Efficiency and Sufficiency Measures, Which Can Increase the Service Delivered by a Process Operating at the Same Activity Rate^a^

strategy	approach	Examples
improve	technology substitution	heat pumps not gas boilers, electric rather than petrol cars^49^
	device efficiency	improving the efficiency of aluminum reduction^131^
	equipment efficiency	insulating homes or reducing^132^ the weight of cars^133^
shift	material efficiency	reducing scrap in production,^134^ more efficient structural design^135^
	operational efficiency	standby savings for idle equipment^136^ or using sensors to control building heating and cooling^119^
	demand efficiency	transport mode-shifting or increase vehicle utilization^137^
avoid	sufficiency	increasing the temperature set point for air conditioning^138,139^

a Many other examples exist cutting across all industries and sectors. “Shift” and “avoid” strategies^56^ could enable the low-ZER demand scenario in Figure 6 because they are particularly under-represented in the other six policy packages shown in Figure 5.

This paper demonstrates that a purely technological approach to climate mitigation is impossible but is it any more realistic to promote mitigation requiring societal change? Many authors argue not, based on analysis of changes people would find acceptable today. However, the rapid effects of unmitigated global warming over the next decade or two will call those results into question. Societal change is a familiar response to previous environmental concerns, for example, with lead in petrol, CFC gases in aerosol sprays, or the use of asbestos but depends on government level review and confirmation of the environmental harm that requires change.^20,140^ Current policy, which promises purely technological solutions, denies and delays that confirmation, without which participatory change cannot begin.

The problem of resource aggregation revealed in this paper has been overlooked because economic modeling approaches and their outputs have become embedded in policy^141^ without verifying their underlying assumptions.^15^ Continued policy prioritization of afforestation, hydrogen, and carbon capture, for example, belies the fact that none of these approaches have yet delivered mitigation at a meaningful scale, has diverted attention from the risks of mitigation failure,^142^ and has narrowed the range of options under consideration.^130^ Ongoing refinement of the details within IAMs can make the results appear more probable, such that it becomes harder to imagine alternatives, even if they may be more likely.^143^ Such techno-optimism is compatible with liberal market policy style^144^ where powerful incumbent emitting industries have, so far, shaped climate policy.^145^ However, it is rooted in power imbalances, for example, where research and modeling are cofunded by industries “locked” into high-emitting practices^146^ and concentrated in the high-consuming Global North.^145^ Novel technologies typically dominate in “cost-optimal” mitigation strategies developed using IAMs because their unknown operating costs are modeled optimistically^147^ while alternatives are not easily incorporated into IAM frameworks.^12^ Yet France’s “sobriety plan” of October 2022 had public support^138^ and around 40% of the mitigation policy recommendations made by citizen assemblies in ten European countries were for “sufficiency measures” with high approval rates.^126^

Although this is not the first work to consider the feasibility of mitigation scenarios, there are two key novelties to this work. First, no previous work has been found which considered the challenge of aggregating net-zero plans and, second, previous quantitative whole-system feasibility assessments are dominantly based on thresholds of infeasibility (such as ref 148). These are hard to ascertain because it is difficult to anticipate all impacts and interactions that might accelerate or delay deployment and take-up. In this work, conversely, feasibility is judged by the distance to a “probable core”, as suggested by^149^ and demonstrated by,^150^ based on the intuitive understanding that more probable options are also more feasible.

While existing integrated assessment models (IAMs) are well-developed and could theoretically represent the whole system resource demands of net-zero policy packages, they were not used in this assessment for three reasons. First, this study aims to address whether all industry-expected demands for energy resources could be met simultaneously. These industry-expected demands and technology choices (for which they are receiving investment) are therefore the inputs to the model. IAMs, in contrast, require socio-economic inputs and (in general) cannot specify technology choices at a sectoral level. Second, IAMs generally offer only a stylized representation of energy requirements, driven by socio-economic assumptions, rather than accounting for physical flows. IAMs may not, therefore, represent the true physical demands of industries and cannot accurately quantify their physical output. Third, the alternative modeling approach expands the range of scenarios, which can be considered, addressing the concerns that the dominance of IAMs may narrow the set of relevant futures, which can be considered.^151^ Many net-zero plans are themselves derived from IAMs, in which carbon storage is generally modeled using lower-than-expected costs, and with very generous limits on annual sequestration, in comparison to other studies.^10^ These constraints are rarely documented.^10^ The modeling results of IAMs are frequently compared^152^ but accessible and comprehensive information describing the model structures and assumptions is difficult to ascertain. All IAMs are subject to similar constraints and biases, however, because they are all economic models based on today’s system.

Modeling approaches used to inform climate policy today obscure risks of supply shortages in three fundamental physical resources, on which all mitigation depends. This has created an endemic culture of burden-shifting, either delaying actions in the hope that carbon storage technologies will expand at implausible rates or using intermediate fuels to shift the responsibility for mitigation across corporate, sectoral, and national boundaries. By creating a calculator to estimate aggregated demand for these resources, we have demonstrated that resource supply in 2050 will be far short of demand. No similar existing plan is therefore likely to deliver on its promises. By reversing our calculation, we have estimated the scale of activity that could be delivered within the budget of probable resource supply and discussed how this could be expanded through intensification, using existing technologies, and with societal participation. We concluded that incorporating aggregated resource constraints into policy design will release a wider portfolio of mitigation options with associated opportunities for entrepreneurial and societal benefit. Pursuit of such participation must begin immediately as it takes time but is essential to avoid the societal catastrophe of mitigation failure.

## Supplementary Material





## Data Availability

All code necessary to run the ZER calculator is publicly available at GitHub at https://github.com/hawkij/ZERCalc. The model coefficients and model inputs used for the figures in this paper are provided in the Supporting Information file and publicly available at GitHub (https://github.com/hawkij/ZERCalc).

## Notes

SI document 1, including: (1) Context and assumptions; (2) Model framework; (3) ZER trajectories; (4) Model verification (PDF)
SI document 2, including: (5) Model coefficients; (6) Model inputs (PDF)
