# Supplementary material for: Aggregating Demand for Three Fundamental Resources to Avoid Burden-Shifting in Climate Policy
Source: Environ Sci Technol. 2026 Mar 26;60(13):9958–72. doi: 10.1021/acs.est.5c12742 (PMC13063813; doi:10.1021/acs.est.5c12742)
Supplement: Supplementary file 1 [file es5c12742_si_001.pdf]

Supplementary Information (document 1 of 2) for:

Aggregating demand for three fundamental resources to avoid burden-shifting in climate policy

Jennifer L Hawkin and Julian M Allwood*

Department of Engineering, University of Cambridge, Trumpington Street, Cambridge CB2 1PZ.

*Corresponding author: jma42@cam.ac.uk

Document 1 (this document):
- Part 1: Context and assumptions
- Part 2: Model framework
- Part 2: ZER trajectories
- Part 4: Model verification

Document 2:
- *Part 5: Model coefficients*
- *Part 6: Model inputs*

Number of pages: 371

- Parts 1-4: S1-111
- Parts 5&6: S112-371

Number of figures: 158

- Parts 1-4: Figures S1-22
- Parts 5&6: Figures S23-158

Number of tables: 425

- Parts 1-4: Tables S1-19
- Parts 5&6: Tables S20-425

Contents

Contents 2
Introduction 3
Glossary 4
1. Analysis context and assumptions 8
1.1 Concepts and definitions of *risk* and *probable futures* 8
1.2 The climatic impacts of greenhouse gas (GHG) emissions 12
1.3 Emissions and sequestration associated with land and biomass 16
1.4 Negative Emissions Technologies (NETs) and Solar Radiation Management (SRM) 21
1.5 Summary of key assumptions 30
2. The Zero Emissions Resources (ZERs) Model 31
2.1 Mathematical framework 31
2.2 Survey and quantification of low carbon technologies 37
2.3 Extending the model 41
3. Estimating probable future supply 45
3.1 Estimating probable future supply 45
4. Verification approach 74
4.1 System validation against 2018 data 75
4.2 Assessing uncertainty in the analysis 85
References (Parts 1-4) 93

Introduction

This document describes the context and assumptions supporting the analysis in the main paper (in Part 1), additional detail to describe the ZERs model framework (in Part 2) and the trajectories outlined in the main paper (Part 3). The approach to verify these is discussed in Part 4. Details of model coefficients and model inputs are given in Part 5 and 6 of the Supplementary Information (a separate file). Part 5 documents the basis for the model coefficients while Part 6 summarises the model inputs used in the main paper.

Glossary

The glossary below outlines the definitions used in this work.

Term	Definition used within this work	Relevance
**Activity**	Provision of a substance or service, quantified by flows of resources. This may comprise multiple delivery processes.	Mathematical framework
**Biomass**	The dry weight of organic plant matter, used to supply services to humans (see Figure S17). Note that the mass of carbon, approximately half the dry mass of biomass, is often used in other studies.	General
**Byproduct**	Any substance or service produced by an activity or process which are not the primary output.	Mathematical framework
**Burden shifting**	Assuming incomplete responsibility for the resources needed or emissions produced when providing a service or substance. It involves transferring accountability for certain impacts to another entity, such as along the supply chain or across national borders. *Note: the term 'burden-shifting' is used in many fields with the more general understanding of transferring the responsibility or impact of something to another entity, often leading to delayed progress overall.*	General
**Carbon Capture**	Separation of carbon dioxide gas for storage or use, either from the atmosphere (by Direct Air Capture, DAC) or from a flow that would otherwise increase atmospheric CO_2 (such as by Carbon Capture of flue gases in industry)	General
**Carbon Storage**	The mass of carbon dioxide gas placed in long-term geological storage. Carbon dioxide which is captured and then released for other uses is not included (i.e. CCU is not included in this value). Carbon stored in other reservoirs (for example temporary storage as biogenic carbon) is not considered to be a valid alternative to near-permanent storage and so is not included, as justified in Section 1.4.	General
**CCS**	Carbon Capture and Storage - this includes both Carbon Capture and Carbon Storage.	General
**CCU**	Carbon Capture and Usage. A process where carbon dioxide gas is captured for use in products such as chemicals or fuels production but then re-released within a short timeframe (within years or decades).	General
**CCUS**	CCU and CCS (see respective definitions)	General
**Climate mitigation scenario**	An imagined version of the future which limits climate change.	General

Term	Definition used within this work	Relevance
**DAC**	Direct Air Capture. A technology to separate carbon dioxide gas from ambient air. Note: DACCS also includes Carbon Storage.	General
**Delivery process (or process)**	A specific, defined and quantified approach/method to supply a substance or service.	Mathematical framework
**End-use activity**	Provision of a final substance or service (see final substances and services)	Mathematical framework
**Exposure**	The potential scale of people, ecosystems, and services that may be impacted by a given hazard (IPCC, 2022a)	Uncertainty and risk
**External resources (or substances and services)**	Material goods and energy services, which are treated as external to the system - although they may be consumed by processes within the system, or produced as a by-product, there is no system process that produces or consumes them as a primary flow. Biomass is an example of this. It is created by growing processes which are treated as external to the model but it is consumed by many other processes as an input. Another example could be scrap steel - this is not currently in the model but could be added as a by-product to other processes - the model could then quantify total production of scrap steel, as an external substance.	Mathematical framework
**Feasible**	Achievable under realistic assumptions (Jewell & Cherp, 2023)	Uncertainty and risk
**Final resources (or substances and services)**	Material goods and energy services, which are demanded by society. The substances/services currently classified as 'final' could be classified as 'internal' in a future iteration of the model. For example Gt steel produced per year is currently a final substance. If 'cars' and 'buildings' (or even transport and shelter) became a final resource instead, steel would become an internal resource.	Mathematical framework
**Hazard**	An undesirable or dangerous event or trend (IPCC, 2022a)	Uncertainty and risk
**Inputs**	Substances or services which are consumed by an activity or process (represented as a negative value)	Mathematical framework
**Intermediate resources (or substances and services)**	Material goods and energy services, which are needed to provide other substances or services within the system but that are not demanded directly by society	Mathematical framework
**Likelihood**	Used in the context of risk to describe the chance of a particular hazard occurring of defined intensities of exposure and vulnerabilities	Uncertainty and risk

Term	Definition used within this work	Relevance
**Modelled resources (or substances and services)**	Material goods and energy services, which are a primary flow of an activity in the system, produced and consumed by the system or society.	Mathematical framework
**Net-zero plans**	Strategies derived by businesses, industries, institutions or nations which aim to address climate change by reducing greenhouse gas (GHG) emissions. These plans may implicitly or explicitly require ‘offsetting’ to achieve net-zero GHGs by 2050, as considered in the ZERs model. This ‘offsetting’ would demand that residual emissions are compensated by negative emissions elsewhere. Net-zero plans may (or may not) be informed by climate scenarios.	General
**Non-Emitting Electricity (NEE)**	Gross electricity generation, where gross operational emissions are zero. Nuclear and renewable power is therefore included in this definition although biomass-fuelled, BECCS and other generation with CCS are not.	General
**Non-permanent storage**	Storage of atmospheric carbon dioxide for <200 years.	General
**Outputs**	Substances or services which are produced by an activity or process (represented as a positive value)	Mathematical framework
**Permanent storage**	Storage of atmospheric carbon dioxide for a time-period of centuries (>200 years).	General
**Plausible**	"Occurable in exploratory scenarios with internally-consistent assumptions" (Jewell & Cherp, 2023)	Uncertainty and risk
**Possible**	"Imaginable, under disruptions" (Jewell & Cherp, 2023)	Uncertainty and risk
**Primary flow**	A single substance or service, which is either the main intended output of a given activity or the input waste flow that the activity aims to treat or manage.	Mathematical framework
**Primary 'output'**	See primary flow. Note that, for readability, the text sometimes uses primary 'output' to refer to the primary flow, which may be an input flow (for example in the case of solid waste management where the primary flow is an input to the activity).	Mathematical framework
**Probable**	Likely in forecast scenarios (Jewell & Cherp, 2023)	Uncertainty and risk
**Probable future**	The likely forecast, based on both historical deployment and future trends, provided there is strong evidence that trends can be relied upon to continue	Uncertainty and risk
**Resources**	Substances or services with economic value or costs	Mathematical framework

Term	Definition used within this work	Relevance
**Sequestered carbon**	Atmospheric carbon dioxide, which is stored, either temporarily or permanently, in another reservoir, such as in the biosphere, oceans or minerals.	General
**Services**	A physical benefit, utility or good for human well-being. Within the model, only energy-consuming services (energy services) are currently considered. Note that defining energy services is challenging and many varied definitions are used – see Fell (2017) for a review of definitions and applications. The 'services' chosen for this work have sometimes been limited by data availability and modelling capability. This is discussed in Section 5.2, and (where relevant) in the dataset of resource flows (Part 5).	Mathematical framework
**Stocks**	An external reserve of resources which grows if the in-year production is greater than the in-year consumption. Stocks may be produced or consumed by the system or may be supplied externally (e.g. biomass can be considered an external stock). Stocks are conventionally considered as something with value so, if it is produced in the system, it would have a primary flow coefficient of +1.	Mathematical framework
**Substances**	Physical goods, including products and materials (solids, liquids and gases)	Mathematical framework
**Vulnerability**	the degree of potential impact (affected by the "sensitivity or susceptibility to harm and lack of capacity to cope and adapt" (IPCC, 2022a)).	Uncertainty and risk
**Waste management activity**	An activity where the primary flow is an input, rather than an output. This is because the resource is not wanted by society (e.g. solid waste, wastewater or atmospheric emissions).	Mathematical framework
**Wastes**	An external deposit of resources which are unwanted by the internal system . These grow if the in-year production is greater than the in-year consumption. Wastes may be produced or consumed by the system or may be supplied externally (e.g. atmospheric emissions can be considered an external waste). Wastes are conventionally considered as a cost so, if it is consumed by the system, it would have a primary flow coefficient of -1.	Mathematical framework
**Zero Emissions Resources (ZERs)**	Non-Emitting Electricity (NEE), Carbon Storage and Biomass	General

1. Analysis context and assumptions

This section explains the context and assumptions behind the ZERs model in three key areas: (1) the risk assessment framework which underlies the modelling assumptions and approaches; (2) the justification for net-zero emissions, and its interpretation in the model; and (3) the impacts of negative emissions on climate, and therefore the model approach to biogenic carbon, negative emissions technologies and land-use change.

1.1 Concepts and definitions of *risk* and *probable futures*

This analysis is based on two key concepts: that of *risk* and *probable futures*. These are closely interlinked because the level of risk is always dependent on the probability of the desirable outcome (and so the probability of the undesirable one), alongside the impact of that outcome. Although risk is now an ingrained part of IPCC assessments, the risks of climate change mitigation failure (opposed to risks of climate change itself) are rarely assessed explicitly.

Feasibility assessments are increasingly used to compare scenarios but these are not considered within the context of risk. Most approaches compare scenarios against feasibility indicators or thresholds across six dimensions - economic, technological, socio-cultural, institutional, geophysical, and ecological/environmental - following the framework suggested by the IPCC [1]. These approaches have three key weaknesses:

1. The 'multidimensional approach' aims to be comprehensive but may not consider significant barriers or enablers. For example, feasibility of the scale-up rate of supply side technologies is often based on the rate of increase in the *share* of electricity generation for the given technology (e.g. in reference[2]). These thresholds are not absolute values and so cannot capture the technology-specific constraints that exist irrespective of growth in other supply technologies. For example, offshore wind energy deployment may be constrained by vessel and port availability, which would not increase with increasing nuclear deployment.

2. Current approaches compare the outputs from IAMs against threshold feasibility criteria, rather than the input assumptions. They do not, therefore, "rigorously analyse the realism of underlying assumptions", a key role of feasibility assessments, according to reference[3].

3. There is no obvious way to aggregate feasibility scores across dimensions or to be consistent in assigning scores - comparative feasibility is therefore arbitrary.

1.1.1 Risk

According to the IPCC definition[4], risks occur due to interaction between hazards, vulnerability and exposure, where each has the following meaning:

- Hazard: an undesirable or dangerous event or trend
- Exposure: the potential scale of people, ecosystems, and services that may be impacted by a given hazard
- Vulnerability: the degree of potential impact (affected by the "sensitivity or susceptibility to harm and lack of capacity to cope and adapt")

The level of risk is the product of the likelihood of a given hazard and its potential consequences, which are in turn a function of the intensity of the hazard, exposure and vulnerability[5].

$$\textit{Risk level} = \textit{likelihood} \times \textit{consequences} = \textit{likelihood} \times \textit{fn(hazard, exposure \& vulnerability intensities)}$$

Risks are in general cascading and compounding [6]: Risks of climate change mitigation failure impacts not only the risk of climate change, but also the risk of social instability (due to the potential failure to provide human services, and as a consequence of climate change itself – see Figure S1). In this study we consider two potential causes of climate change mitigation failure which can be addressed by modifying our climate mitigation plans. These are the hazards that the rate of demand for resources in 2050 is higher than the rate at which they can be supplied, without exceeding:

- other planetary boundaries (the sustainable rate of availability); and/or

- the constraints of infrastructure deployment i.e. the demands at a given point in time are greater than could be achieved within probable expectations of technology deployment rates.

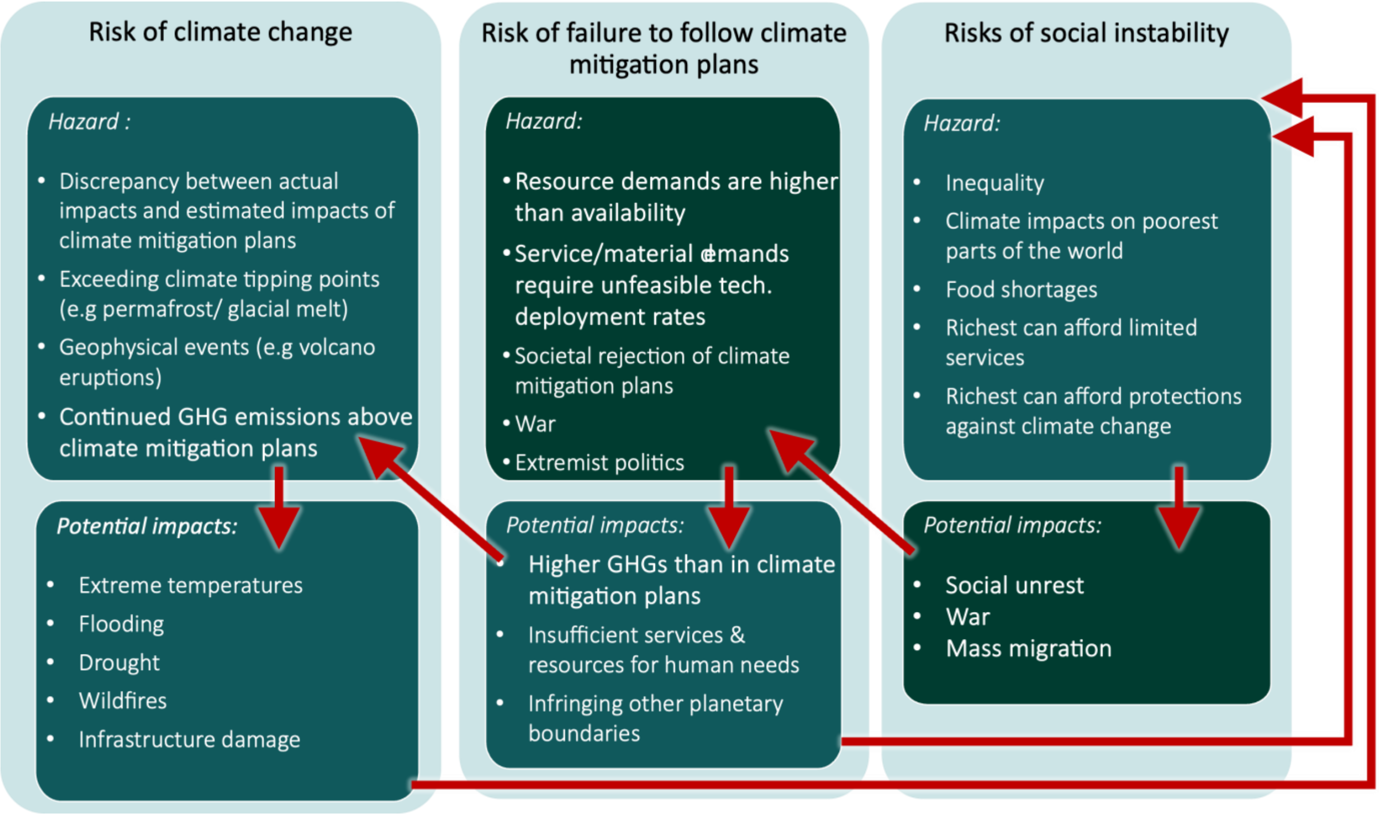

Figure S1 – A schematic representation of some of the cascading interlinkages between risks, hazards and impacts, relevant to this study. Some relationships have not been shown (e.g. the impact of extreme weather on resource availability) to simplify the diagram.

These can be summarised as supply shortage hazards - this work aims to illustrate their likelihood. In the current study only availability of biomass, carbon storage and non-emitting electricity have been considered. The same framework could, however, be applied to any other resource or service of interest.

The likelihood of supply shortages is inversely related to the feasibility of sufficient supply; this can be assessed, using the approach of reference[7], as the "distance to a probable core". For the ZERs model, the probable core is the likely supply of ZERs in 2050 based on probable technology deployment rates of technology and planetary boundaries.

1.1.2 Probable and possible futures

For this analysis, a *probable* future is defined as the likely forecast, based on both historical deployment and future trends, provided there is strong evidence that trends can be relied upon to continue. This understanding of *probable* aims to minimise risks and uncertainties in the supply trajectories, since these would obscure the overall conclusions.

The terms used here are based on the more general definitions suggested by reference[7]:

- Probable: “Likely in forecast scenarios”; and
- Possible: "Imaginable, under disruptions".

The authors also propose the use of ‘feasibility spaces’ to assess scenarios from an ‘outside view’, considering the scenario in the context of statistics of similar scenarios. The ‘feasibility space’ may have multiple ‘feasibility zones’, to separate more feasible from less feasible solutions.

Using the ZERs model, the overall infeasibility of net-zero strategies can be understood to scale with the estimated supply shortage of its demands when compared to the *probable* supply availability. *Probable* supply for each of the ZERs is estimated in Section 3.1, which is used to define the ‘feasibility zone’ where demand is within *probable* future supply. To reflect the uncertainty in modelling the future, additional ‘feasibility zones’ are based on *possible* supply expectations (see Section 4.2).

1.2 The climatic impacts of greenhouse gas (GHG) emissions

The climatic impacts of climate mitigation efforts may not be straightforward since there are multiple chemical, physical and biological processes acting simultaneously. This section aims to summarize the relevant climate science and so justify the assumptions and approaches used in this analysis.

It is now well understood that carbon dioxide (CO_2) emissions may last up to millennia in the atmosphere (as described by, for instance, Solomon et al., 2009), and that there is a nearly linear relationship between cumulative anthropogenic CO_2 emissions and CO_2 induced global warming, as shown by Allen et al. (2009) and Matthews et al. (2009). This suggests that warming stops when CO_2 emissions stop, and the level of warming is determined by the quantity of cumulative CO_2 emissions at that point. Without other sources of CO_2 withdrawals (negative emissions), CO_2-induced surface warming may remain for decades to centuries [11], and average atmospheric temperatures may not reduce significantly for at least 1000 years due to the interactions between oceans, the atmosphere and the climate [8]. More recent research has revealed that there is significant uncertainty in global surface temperature after the point CO_2 emissions stop - either cooling or additional warming (potentially exceeding 15%) is plausible according to reference[12] – but the association between cumulative CO_2 emissions and warming has not been questioned.

Non-CO_2 GHGs generally have shorter residence times but significantly higher effective radiative forcings (ERFs); an equivalent mass of gas will have a stronger warming impact but over a shorter time. Non-CO_2 GHGs are generally better represented in climate modelling by the rate of ongoing emissions rather than cumulative emissions[13]. Reductions in non-CO_2 emissions are especially important to compensate for reductions in aerosol emissions and their resultant cooling effects which will arise with reduced combustion of fossil fuels. Reference[14] used a climate-model simulation to show that eliminating CO_2 and aerosol emissions while maintaining non-CO_2 GHG emissions led to “an immediate and rapid climate warming” of around 1°C, but eliminating all three significantly constrained this warming. It is generally expected that eliminating CO2 and non-CO2 GHGs would lead to a decline in temperatures [15].

These disparities in climate impacts explain why different gases are often treated separately in climate mitigation targets and analyses, such as in *net-zero* plans, as considered in this analysis.

1.2.1 Net zero

The prominence of the *net-zero* concept stems from scientific research in the late 2000s leading to the Paris agreement in 2015, which stated:

> *"the Parties aim to … achieve a balance between anthropogenic emissions by sources and removals by sinks of greenhouse gases in the second half of this century" (Art. 4)*

The idea of *net-zero* then, is that the quantity of emissions into the atmosphere are balanced by removals out of it; carbon dioxide (CO_2) for instance can be stored as carbon in vegetation, soils and water, or as a gas in geological formations by Carbon Capture and Storage, CCS, processes.

The meaning and scientific justification for net-zero

Reports compiled by the IPCC evidence the need for net-zero carbon dioxide (CO_2). The 2018 Special Report on 1.5°C [16] concluded that although global average warming of 1.5 °C creates extensive risks for both natural and human systems, the impacts would be significantly lower than with increased warming [16], and that,

> *"Limiting warming to 1.5°C implies reaching net-zero* CO_2 *emissions globally around 2050 and concurrent deep reductions in emissions of non-*CO_2 *forcers, particularly methane"* [17].

There is broad consensus in the literature that although CO_2 emissions must reach net-zero by around 2050, residual emissions of other greenhouse gases at the same date may be compatible with limiting warming to 1.5°C [16]. This is because non-CO_2 forcers generally have much shorter climatic effects [18].

Treatment of net-zero within the ZER Calculator framework

Since "deep reductions in emissions of non-CO_2 forcers" are needed to limit warming [17], the model includes methane (CH_4) and nitrous oxide (N_2O) in the target for net-

zero. Other approaches to quantify these gases were considered, such as allowing limited non-CO_2 emissions, but these would complicate the model solution algorithm (Section 2.1). Such approaches could be considered in future work.

Although mid-century net-zero non-CO_2 emissions are not necessarily required to limit warming, this approach (including CH_4 and N_2O in the net-zero target) is supported by the following observations:

- Estimated dates for net-zero GHG are dependent on the shape of the assumed emissions trajectories. For example, if near term emissions reductions are not as rapid as has been assumed, the date for net-zero needs to be sooner to achieve the same level of climate change mitigation (IPCC AR6 Ch3 Cross Chapter Box 3). This is significant given that 1.5°C compatible pathways with net-zero GHGs assume rapid short-term emissions reductions with immediate downward trajectories (within five years) which are not reflected in current global policies, according to analysis by reference[19], as can be seen in Figure S2.
- Although there are 1.5°C-compatible pathways which reach net-zero GHG emissions around 2070-2100 (range of median 5-year intervals for relevant pathways in IPCC AR6), the lowest 5th percentile pathways reach net-zero GHG by 2050 (according to IPCC AR6 Ch3 Cross-Chapter Box 3). This means that, excluding modelling uncertainties, our model framework is likely to mostly limit risks and impacts to people, economies and ecosystems in the moderate to high range [16]. This is consistent with our overall approach which aims to demonstrate the risks of supply shortages in mitigation plans; if we make high risk assumptions in the model framework we would obscure the conclusions.
- CH_4 and N_2O made up around 90% of non-CO_2 emissions (when measured in GWP-100[1]) both in 2020, and in the comprehensive GHG abatement strategy developed by reference[20].

[1] The global warming potential (GWP) is an index to measure the radiative forcing associated with a unit emission of a given substance over a chosen time horizon (e.g. 100 years for GWP-100) relative to that of carbon dioxide [4]. GWP is therefore a measure of the overall impact on warming, considering both the time a substance remains in the atmosphere and its warming efficacy. GWP is an example of an emissions metric, “a simplified relationship used to quantify the effect of emitting a unit mass of a given greenhouse gas (GHG) on a specified key measure of climate change” [4]. Other examples include global temperature change potential (GTP), global damage potential, and GWP*. Each has different strengths and weaknesses but they must be used consistently in analyses to draw valid conclusions.

Residual methane and nitrous oxide emissions are compensated by CO_2 sequestration, using GWP over 100 years (consistent with the metric used by IPCC pathways). Given net-zero plans with different ratios of CO_2, CH_4 and N_2O could have vastly different climate impacts [21], a future version of the model could make a distinction between different GHGs to refine this assessment and/or consider more appropriate emissions metrics.

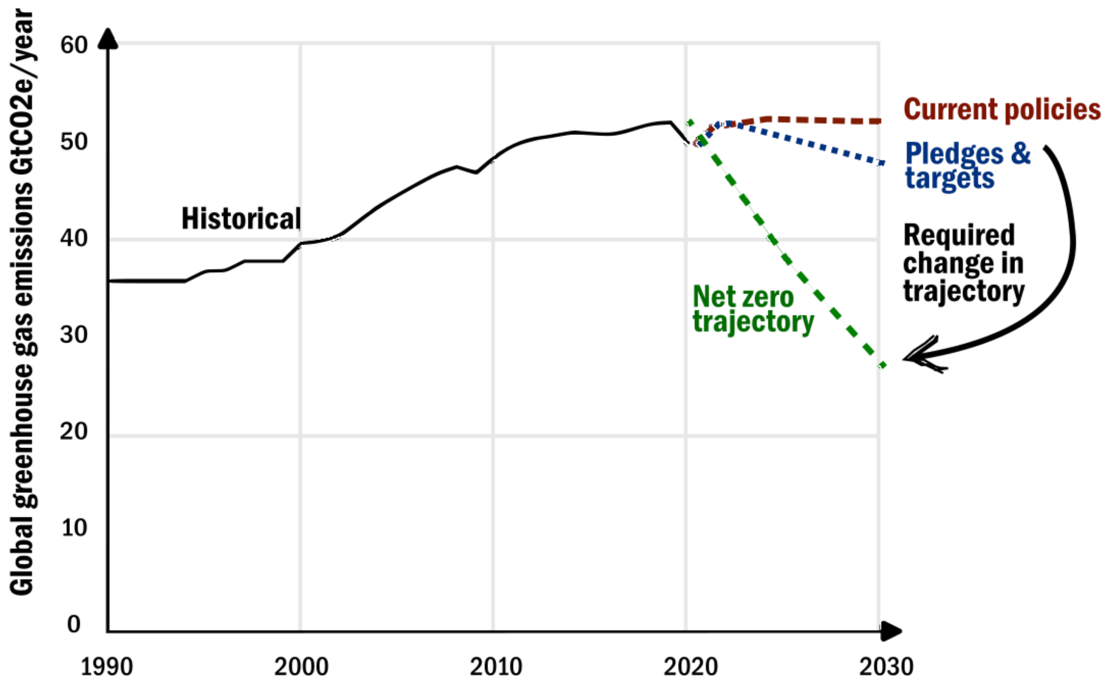

Figure S2 - Figure adapted from analysis by reference[19] which shows the immediate diversion between global policies and 1.5 compatible pathways.

1.3 Emissions and sequestration associated with land and biomass

When plants grow, they sequester carbon. As described by reference[22], this carbon is effectively moved from an atmospheric reservoir into a biospheric one, and so may be continually exchanged by processes of decomposition and combustion, or due to changes in atmospheric CO_2 levels. They argue that, unless this carbon is ultimately transferred to low-leakage geological storage (for example, via Bio-Energy with Carbon Capture and Storage, BECCS), it may continue to impact the climate system, and so cannot compensate for emissions of fossil carbon. In contrast, many climate mitigation proposals include the use of biogenic carbon and changes to land-use and forestry as a form of negative emissions. The following section outlines and justifies the treatment of biogenic carbon in this analysis.

1.3.1 Treatment of biogenic carbon in fuels, products and food

For the model, it is assumed that the carbon in emissions from biomass (from respiration, combustion and decomposition) is balanced by carbon sequestration in growing biomass within the same year, and that there are no net changes in carbon stocks nor net land-use emissions. This means that carbon dioxide absorbed in photosynthesis, and emitted in respiration or combustion, does not need to be explicitly accounted for, consistent with IPCC 2006 Guidelines [23]. In most cases biomass is effectively assigned an emissions factor of 0.

There are three augmentations to this approach:

- Some of the carbon stored in biomass (biogenic carbon) is emitted to the atmosphere as methane, such as in livestock enteric fermentation, from anaerobic decomposition of waste on landfill sites or crop residues, and burning of agricultural biomass. Since methane has a warming impact around 30 times greater than carbon dioxide (on a 100-year basis), these emissions are accounted for explicitly. This is also the same approach used in IPCC 2006 guidelines [23].
- Biogenic carbon which is transferred to permanent storage is quantified as an inflow to the storage process, where it has not been previously accounted. For example, biogenic carbon used as part of a Carbon Capture and Storage process (such as BECCS) is accounted as an inflow of atmospheric carbon-dioxide (a negative emission), corresponding to the carbon stored by the system measured

as its equivalent mass of carbon-dioxide. Biomass in products is generally considered to be temporary storage in this model and so does not include an inflow of atmospheric emissions (negative emissions).

- Use of biogenic carbon in fuels and feedstocks which could be made from a combination of biogenic and fossil feedstocks is quantified as carbon sequestration. Examples include Methane, Methanol and HVCs. In these cases, biogenic carbon sequestration is accounted as an inflow of atmospheric carbon-dioxide (a negative emission), corresponding to the carbon stored by the fuel/feedstock measured as its equivalent mass of carbon-dioxide. This is needed to provide consistency with the emissions factor assigned to using the fuel in other processes. It is assumed here that the products produced from these feedstocks are either combusted (producing emissions) or permanently stored in the product (i.e. that the product is not degradable into chemical components).

The assumption that products derived from long carbon chains are not degradable into chemical components may be approximately true for some plastic products with the carbon "returned to the ground" by controlled landfill [24]. But, as found by Chamas et al. (2020), there is very wide variation in decomposition times, depending on the plastic type, shape and decomposition conditions. Overestimation of sequestration due to this assumption is assumed to be relatively small compared to the uncertainty in other parts of the model and the approach is conservative to the analysis hypothesis (that permanent carbon storage available will be insufficient for the aggregated demands of net-zero plans). A schematic representation of the biomass carbon flows is given in Figure S3.

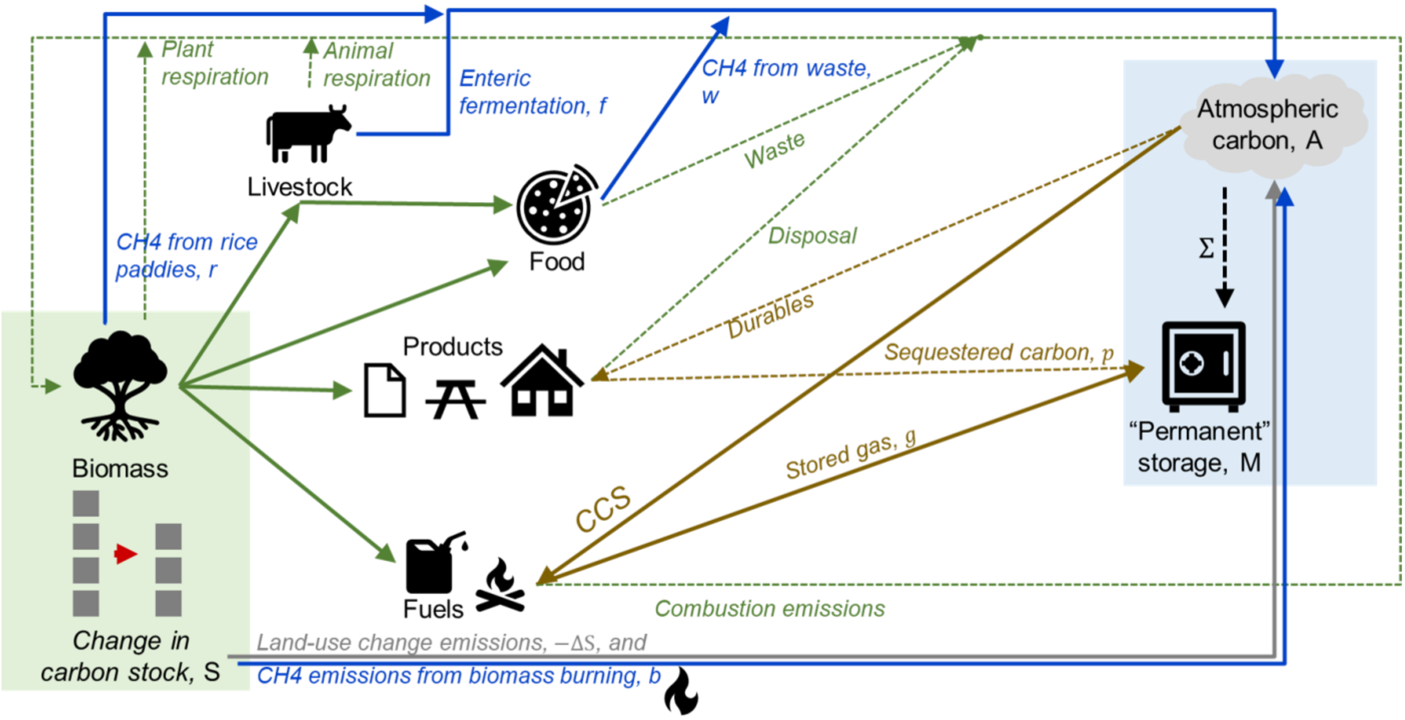

Figure S3 - A schematic of biomass carbon flows through the model; solid lines are explicitly quantified in the model; dotted lines are not explicitly. Green lines show flows of carbon which are assumed to be balanced. Using the same assumptions of IPCC 2006 Guidelines: "the CO_2 photosynthesized by plants is returned to the atmosphere as respired CO_2" and so are not explicitly accounted for. Blue lines show methane emissions, brown lines show flows of anthropogenic carbon sequestration and grey is land-use change (and outside the model scope). The net overall storage in black (Σ) will be the net sum of the quantified flows. Changes in terrestrial and ocean carbon stocks are not quantified in the current model, as described in the main text.

A limitation to this approach is that carbon sequestration in many products (for example as timber in construction) is not quantified. At a very simplified level, greater use of biomass in products would increase the carbon stocks in products, reducing the carbon in the atmosphere (assuming an equilibrium position with no change in growing biomass stocks). However, when these products are disposed there would also be a change to the composition of waste, thus changing emissions from waste management. Using biogenic carbon as production materials therefore simply delays the release of carbon into the atmosphere until the end-of-life of the product, as well described by reference[25].

An exception is if the disposed product is placed in permanent storage, effectively acting as a net carbon sink. To account for the first case (where carbon release is delayed) would need a stock model which is out of scope for this current work. The

second case could be approximated without a stock model, however, by quantifying carbon sequestration as a negative emission at the point of product production; for example, a delivery process for timber construction would include a negative emissions flux. This has not been included at this stage since the assumption of permanent storage is unlikely, and temporary storage is not accounted in the model (page 23).

The potential impact of excluding biogenic sequestration in products is over-estimating the demand for negative emissions. The current use of biomass in products is estimated to be relatively small - Harris et al. (2021) estimated harvested wood products to account for an annual sink of 0.16 Gt CO_2/yr over the period 2001–2018. This is only around 0.3% of current global emissions and would be offset by emissions released in biomass harvesting, processing and transport (as described for timber by Hawkins (2021)) but it could be significant in comparison to the potential availability of carbon storage by 2050. If the results show there is no constraint on the availability of biomass, these assumptions could be revisited.

In summary, biogenic carbon is generally not quantified in the model, based on the assumption that sequestration and emissions of biogenic carbon are balanced. There are three exceptions to this: where carbon is rereleased as methane (such as via enteric fermentation or in waste management), where biogenic carbon is permanently sequestered (such as in BECCS), and where biogenic carbon is used to produce a drop-in fuel which replaces a fossil fuel or feedstock (such as for bio-methane). This approach is inexact but used as a first reasonable approximation. The impact of these assumptions could be investigated in further work.

1.3.2 Accounting for land-use, land-use change and forestry (LULUCF) emissions

Carbon removals by natural sinks (LULUCF) were identified as a key policy option at Rio in 1992 and continue to play a prominent role in mitigation proposals, but there has been little progress reversing this balance[26]. Planted forest sequestered around 60 Gt CO_2e in the period 1850-2015, dwarfed by land-use change and forestry emissions of around 600 Gt CO_2e over the same period, while the net flux from LULUCF in 2015

was still around 4 Gt CO_2e/yr according to the book-keeping analysis by [27]. Furthermore, as the IPCC conclude, "climate change exacerbates the rate and magnitude of several ongoing land degradation processes and introduces new degradation patterns" [28]. It will therefore be extremely challenging to eliminate land degradation and deforestation emissions.

Reforestation and land restoration similarly face four key challenges:

- They are subject to reversal, and so, without extensive monitoring, reporting and validation, cannot be assumed to be permanent sinks. Reversal mechanisms may include drought, harvest, wildfire, disease, or pests [29]. These mechanisms have already been observed in forest carbon projects [30], and these disturbances are likely to increase with climate change [31].
- Reforestation and land restoration may imply conversion of land that had been previously converted to cropland (i.e. would impact the assumptions of biomass potential).
- The rate of potential storage in 2050 is limited by the rate of planting, growth and carbon saturation of vegetation but there is significant uncertainty in understanding forest carbon cycles [32] and geographical variations [33].
- There are conflicting factors and ambiguities that make it hard to determine whether tree planting is net-positive or net-negative for both climate-change as well as broader socio-environmental impacts, as explained by reference[34] in a holistic review of tree planting. Overall uncertainty in estimating land-use emissions is similarly high – emissions from deforestation, for instance, have uncertainties up to 50% according to reference[35].

Given these challenges, emissions associated with land-use and land-use change are not explicitly quantified in the model. Instead, it is assumed that any remaining land-use emissions in 2050 are balanced by equivalent regeneration or afforestation, giving net-zero land use and land use change emissions. The overall future potential of forest restoration, and so potential impact of this assumption, is highly uncertain. Equally, crediting this sequestration would add significant risk into the model and so obscure the conclusions.

1.4 Negative Emissions Technologies (NETs) and Solar Radiation Management (SRM)

Alongside approaches to reduce emissions of greenhouse gases (GHG), many climate mitigation strategies include more novel approaches, including Negative Emissions Technologies (NETs) and Solar Radiation Management (SRM). This section explains how these are considered within this analysis.

The term Negative Emissions Technologies (NETs) describes technologies (and sometimes practices and approaches) which extract Carbon Dioxide (CO_2) from the atmosphere and store them over a long duration. There are also some proposed examples of methane capture and storage but these are very immature and so not considered within this analysis (consistent with IPCC AR6). NETs are crucial to most net-zero strategies so are included in the model in the form of geological carbon storage. The justification for this choice is given in the remainder of this section. In contrast, Solar Radiation Management (SRM) refers to methods that aim to limit global warming by reducing the net radiation acting on the earth by other means than greenhouse gas (GHG) mitigation; for example, reducing the amount of incoming solar radiation by adding aerosols into the atmosphere to increase the brightness and reflectivity of marine clouds. The effects of SRM are complex and poorly understood [36] and are therefore out of scope of this model.

1.4.1 Physical concepts for understanding NETs validity

The use of NETs in most mitigation strategies relies on the concept of *physical equivalence*; that the impact of removing 1t CO_2 has the same impacts on the atmosphere (and therefore climate) as emitting 1t CO_2e GHG. For this to be true, temporal differences and biophysical effects must be accounted for. Temporal differences describe the variation between the actual time sequestration profile of a unit of NET and the emission it is expected to compensate. This is particularly important where the duration of storage is significantly shorter than the length of time emissions persist in the atmosphere. Clearly non-CO_2 emissions are therefore unlikely to be directly equivalent to removal of CO_2 because they have different residence times and climate impacts (as discussed in Section 1.2.1). Biophysical effects, which change the energy balance at the earth's surface, may occur at local and global levels - for

example reforestation may make the surface darker, increasing heat absorption, and introduce or retain more moisture locally. While economic equivalence is also commonly used in climate mitigation optimisation models, for instance by reference[37]. Although convenient for economic analyses, this type of equivalence does not guarantee climate change mitigation and so irrelevant here.

For this analysis, NETs are only included if the temporal differences and negative biophysical effects are expected to be small, when considering emissions of CO_2. Non-equivalence of non-CO_2 emissions may be significant and could be addressed in a future iteration of the model, as mentioned in Section 1.2.1.

Temporal variations can be described by *permanence, risks of reversal*, and *delays to sequestration*. The concepts of *permanence* and *reversal risks* are critical to understanding whether temporal differences are likely to be significant; provided storage can be considered permanent with low reversal risks, temporal differences are considered insignificant for the purposes of this study. As described in Section 1.2, atmospheric carbon dioxide emissions may persist for millennia. The same is true for some forms of negative emissions technologies, such as geological storage where carbon may be sequestered for up to 1000s of years. Other NETs may have significantly shorter intended durations (for example where carbon is stored in products and re-released at the end of life), or where planted forests die and decay at the end of life; these are termed forms of *temporary* or *impermanent* storage.

The additional consideration of *delays to sequestration* may occur when the profile of emissions sequestration is not a simple step profile with a constant quantity of emissions mitigation per year. A simple example is when carbon is sequestered in a tree planting project – actual sequestration will only begin when the trees begin to grow and will increase to a saturation point at maturity, illustrated well by reference[25] and reference[38]. An alternative example of a delay to removal, where carbon is sequestered in oceans, is well described by reference[39]. A time delay occurs while the carbon system equalises, transferring carbon from the atmosphere to the oceans. This type of temporal variation would require a stocks and flows model so cannot be included in the current model which represents only one year's flows.

If the carbon stored compensates for additional GHG emissions but is later re-emitted (i.e. when the storage is impermanent), this can be considered a delay to emissions which results in net warming (see detail in 1). Similarly, all forms of carbon storage have *reversal risks*, whereby carbon may be re-released, again behaving as a delay to emissions. In the case of 'permanent' storage in geological formations, the risk of reversal by gas leakage is small [40], as observed in operational storage such as the Sleipner CO_2 Storage Project, which was commissioned in 1996 but shown "no evidence of leakage or harmful CO_2 movement in the formation" since (Robertson & Mousavian, 2022). Natural storage reservoirs, on the other hand, are subject to many possible reversal mechanisms including drought, harvest, wildfire, disease, or pests which may become less predictable with increasing levels of climate change [29].

1.4.2 Justification for excluding non-permanent storage

Climate change mitigation aims to both reduce future average global temperatures and minimise cumulative warming. Although temporary storage contributes to the second of these goals and can provide significant social value [41], it does not reduce, and may even increase, future temperatures. As explained by reference[34], removing carbon from the atmosphere reduces the driving force for ocean uptake of CO_2. If the sequestered carbon is subsequently rereleased into the atmosphere, the resulting CO_2 concentration and radiative forcing, is therefore higher than if carbon had not been temporarily removed. This means that, counterintuitively, temporary carbon storage can lead to higher future temperatures and is not aligned to net-zero goals.

On this basis, temporary storage and storage in the biosphere can only be counted as a 'removal', if it can be either guaranteed that it will not be rereleased over timescales of centuries, or if the subsequent rerelease would be counted as an anthropogenic emission which would be offset by future removals. Since no mechanism exists to guarantee this, carbon storage with low permanence (<200 years) is not included in the model. Although permanence has been used as a key criterion to determine whether NETs should be accounted for, other aspects of uncertainty have also been considered.

1.4.3 Assessment criteria for NETs inclusion

Carbon dioxide sequestration is only included in the model if its availability in 2050 is *probable*. A framework to determine the *probable* availability of any given NET is shown in Figure S4: availability can be estimated based on the technical potential and deployment rates only if there is sufficient confidence that a given technology is compatible with net-zero aims and timeframes. This framework was developed by considering which commonly considered properties of NETs are relevant for estimating probable future availability, and which aspects are missing from the literature. These were grouped into those aspects which influence the confidence that the technology is compatible within the aims of net-zero targets and will reach commercialisation (confidence), and those which influence its scalability after commercialisation (scalability).

Past research, including a three-part comprehensive review [40,42,43], commonly consider many aspects of NETs in isolation, but these are rarely considered holistically to draw conclusions around the probability of their availability. Reference[44] developed a framework to map uncertainties in measurement and modelling of individual NETs when they are each considered at a project level but they do not consider the global potential, nor aggregated deployment constraints. Other literature often uses economic models to estimate future availability (such as the review of economic limits to seaweed farming for climate change mitigation by reference[45]), but no literature has been found that considers the potential constraint of supply chain growth and infrastructure deployment rates to estimate probable future availability. The next section applies these criteria to determine which technologies to include in the model.

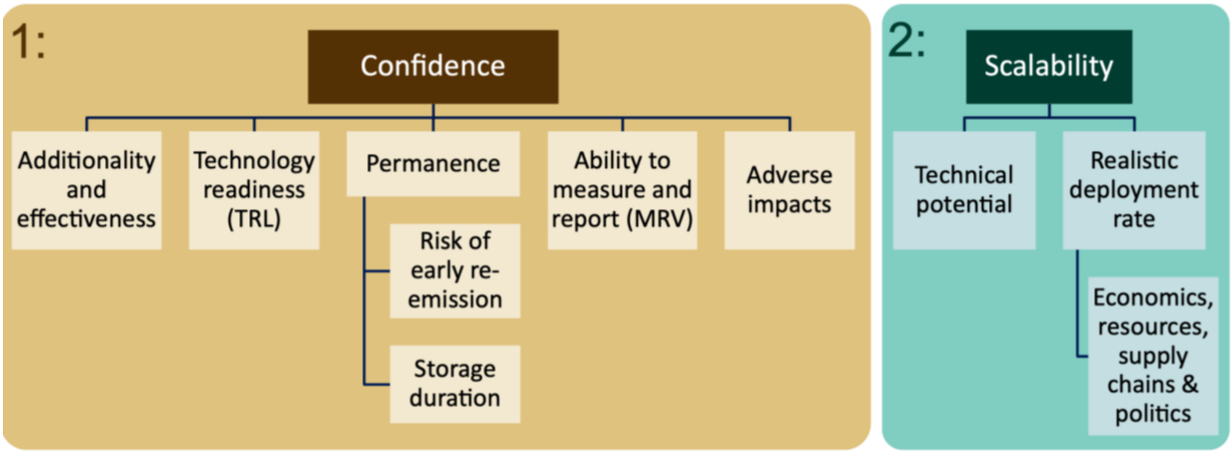

Figure S4 - A framework of criteria to determine NETs probable availability. NETs are included in the model only if there is there is (1) sufficient confidence that they are compatible within the aims and timeframes of net-zero targets and will reach commercialisation, and if (2) the NET is sufficiently scalable to be deployed at a significant rate by 2050. The 'additionality and effectiveness' criterion considers whether the NET would reduce atmospheric CO_2 compared to if it were not implemented. 'Adverse impacts' considers whether it could lead to unwanted environmental or social impacts.

1.4.4 Categorisation and selection of NETs

Types of NETs are sometimes grouped by the method of capture, the means of storage, or the duration of storage. For this study prominent NETs have been characterised in four groups according to their storage medium: the built environment (use of timber in construction, and bio-based products), land-based storage (including above ground biomass, soil and minerals), ocean-based storage (including marine sediment and deep ocean waters), and geological reservoirs (as used to store gases). This section considers these NETs against the compatibility criteria from Figure S4 to determine which should be included in the analysis.

NETs have been assessed against compatibility criteria as high/medium/low risk to meet the requirements set out in Section 1.4.3, as summarised in Table S1. Some key aspects are also briefly discussed below. Only geological based storage via carbon capture and storage were deemed to have no high-risk criteria and are therefore included in the model. This approach is supported by the comprehensive assessment

of carbon removal certainty quantification by reference[44] which considered six carbon dioxide removal approaches.

Although land-based storage, mostly in the form of restoration or creation of forest and peatland, dominate national and regional climate mitigation plans, it is subject to two significant uncertainties. The first is that land-based storage may entail biophysical feedbacks to warming, affecting the energy balance at the earth's surface in a way which is hard to quantify [46]. Reference[47], for instance, found that up to 50% of the climate effect from sequestration in temporary reforestation could be offset by the decreased albedo since forests generally absorb more heat by solar radiation than grasslands or bare ground. The second concern is that sequestered carbon may be rereleased due to extreme weather and climate-related events, which are becoming more frequent with ongoing climate change [48]. The WRI estimated, for example, that wildfires are burning nearly twice as much tree cover as they did 20 years ago [49].

Ocean-based storage is similarly too high risk to be included in this assessment. In particular, methods to assess the carbon uptake and permanence of storage are lacking [50]. This limits any potential economic or regulatory driver for deployment since there are no trusted approaches to monitoring, reporting, and verification (MRV).

In summary, only Carbon Capture and Storage (CCS) with permanent geological sequestration does not include any high-risk criteria (Table S1). Note that CCS is defined in this work as to include Carbon Capture and Carbon Storage, as defined in the glossary, CCS therefore includes DACCS and BECCS. The deployment of BECCS is likely to be constrained by biomass feedstock [51], rather than the deployment of capture and storage technologies but the model accounts for this constraint by quantifying the overall biomass demands. Following the risk assessment framework, described in Section 1.1, CCS is therefore the only form of Negative Emissions Technology (NET) currently quantified in the ZERs model.

Storage medium	Basis for negative emissions	Carbon Dioxide Removal, CDR, Method	Additionality/ Effectiveness	TRL	Permanence: risk of early re-emission	Permanence: storage duration	Monitoring, Reporting & Verification, MRV	Adverse impacts
Buildings & products	Use of biomass as a material for products e.g. plastics	Bio-based products	Maybe: Although biomass harvesting and processing produces emissions, replanting enables carbon reabsorption over the product lifetime. Efficient use of timber can result in net sequestration by the end of life; poorly managed harvesting may not. See Hawkins (2021).	6-9	High: Unless novel waste management processes are put in place, emissions will certainly be re-released at end of life	Emissions re-released at end of life unless CCS is used. 0 - 10 yrs	Medium - Easy: As with Timber in Construction	Biodiversity risks if plantations are one single species. Potential competition with agricultural land.
	Harvesting timber from mature forests allows for new planting and continued carbon uptake while carbon is stored in building materials.	Timber in construction		8-9		30-60 yrs	Medium - Easy: Although greater standardisation of approaches is needed	
Vegetation, soils and sediments	Restoration or creation of high-carbon-density ecosystems which increase carbon stocks	Forests, peatland, wetland	Likely, assuming good accounting practices and measures to avoid leakage. But wetlands/peatlands, could increase methane emissions decreasing impact of net sequestration. Land cover change may cause albedo changes.	8-9	Medium - High: Reversal of carbon removal through wildfire, disease, pests, drought may occur.	0-100 yrs	Medium: Challenge of dispersed widespread monitoring. Satellite and sensor technology may improve feasibility.	Afforestation can affect local and regional conditions (temperature, albedo & precipitation). Potential implications on food & biomass availability due to competition for land. Forest management using fertilisers and introduced species could reduce biodiversity and increase eutrophication & upstream GHG emissions.
	Changing land management practices in such a way as to increase the carbon content of soil. May include reduced tillage, adjusting crop management, and improved water management.	Agricultural practices	Likely: increased nitrous oxide emissions due to higher levels of organic nitrogen in the soil may limit net sequestration. Potential for double-counting if these are considered as reductions in agricultural emissions.	8-9	High: This option depends on continuing practices	Uncertain	Difficult: Disperse and distributed farms make practices hard to monitor; no agreed approach; significant uncertainties in quantification.	Inappropriate deployment at large scale can lead to competition for land with biodiversity conservation and food production

Storage medium	Basis for negative emissions	Carbon Dioxide Removal, CDR, Method	Additionality/ Effectiveness	TRL	Permanence: risk of early re-emission	Permanence: storage duration	Monitoring, Reporting & Verification, MRV	Adverse impacts
Vegetation, soils and sediments	Organic matter is heated in oxygen-limited environments (pyrolysis and gasification) so that carbon is retained. Biochar is then applied to soils.	Biochar	Likely: Production emissions will discount net sequestration. Biomass harvest must be sustainable. Land cover change may cause albedo changes that reduce mitigation effectiveness.	6-7	High: 'accidental' reversal possible by decomposition, erosion & burning; 'intentional' reversal occurs if the treated land is developed or tilled.	10s-1000 years	Difficult: As with other agricultural practices, although the IPCC provides a biochar MRV methodology as an option for national inventories.	Environmental impacts associated with particulates; competition for biomass
In oceans	Addition of nutrients to the ocean increases the photosynthesis rate, and the 'biological pump' of biomass to the deep ocean.	Ocean fertilisation	Sequestration offset by nutrient production & distribution. Potential for changes in ocean biogeochemistry & production of N_2O & CH_4.	1-2	Potential for decadal-to-millennial-scale return to the atmosphere of nearly all the extra carbon removed,	CO_2 reaching durable storage is uncertain, due to re-metabolization.	Large- scale and long-term ship operations would be required	Ecological impacts are difficult to predict. Toxin producing algae have been observed in studies which can cause algal blooms and oxygen depletion.
	Cultivating macroalgae captures CO_2 from the atmosphere as it grows. By deliberately sinking the product (rather than using it) carbon may be stored in the deep ocean.	Ocean biomass sinking (macroalgae)	Significant uncertainties regarding: the carbon share which would sink to the deep ocean, whether biomass decomposes to produce non-CO_2 GHG gases, and the impacts on carbon removal of the broader ecosystem (e.g. phytoplankton).	1-2	Many uncertainties exist around the final destination of sequestered carbon.	0-100 yrs in biomass 1000s yrs in sediments	Verification of how much biomass reaches the deep ocean is challenging, and would vary depending on site locations, currents, and environmental conditions.	Large uncertainties exist: Potential ecosystem and broader environmental impacts.
	Carbon dioxide removal associated with rooted coastal vegetation (e.g. tidal marshes, mangroves and seagrasses)	Blue carbon management (coastal rooted vegetation: tidal marshes, mangroves & seagrasses)	Likely, assuming good accounting practices and measures to avoid leakage.	2-3	Significant uncertainty in the response of coastal wetlands to climate change. If degraded or lost, these ecosystems are likely to release most of their carbon to the atmosphere.	0-100 yrs in biomass 1000s yrs in sediments	Challenging, given dispersed nature, and uncertainties around sequestration mechanisms.	Land rights issues. Potential competition for coasts for revenue generation/growing food.

Storage medium	Basis for negative emissions	Carbon Dioxide Removal, CDR, Method	Additionality/ Effectiveness	TRL	Permanence: risk of early re-emission	Permanence: storage duration	Monitoring, Reporting & Verification, MRV	Adverse impacts
In oceans	Ground silicate rocks (e.g. basalt) spread over large areas of cropland; chemical reactions remove atmospheric CO_2.	Enhanced weathering	Uncertainty around carbon sequestration potential in the field. Net sequestration reduced by emissions from mining, transport and deployment operations.	3-4	Carbon is expected to be stored in soils & dissolved in water which eventually reaches the oceans. The distribution between these, and the permanence in soils is uncertain.	Carbon that reaches marine carbon precipitation could be 1000s yrs but the final destination of carbon is uncertain	Weathering mechanisms are well understood, but field trials needed to understand ultimate destinations of absorbed carbon. No MRV protocols exist.	Mining impacts. Air quality impacts of rock dust when spreading on soil. Uncertainty around carbon long-term environmental and agricultural impacts, e.g. impurities may introduce toxic elements into the soil.
	Distribution of lime or other minerals into the ocean to increase alkalinity - this means it can absorb more atmospheric CO_2.	Ocean alkalinity enhancement	Net sequestration is reduced by process emissions from lime production as well as those from mining, transport and deployment operations	1-2	Unknown - no field trials to date. If local mineral precipitation occurs, waters carbon-carrying capacity is reduced & CO_2 uptake reversed.	100s years	Concerns around precision and costs. There are no MRV protocols.	Large uncertainties exist. Increased seawater pH and saturation states may impact marine biota. Possible release of nutritive or toxic elements and compounds. Mining impacts
Geological formations	Use of chemical processes to extract CO_2 from the atmosphere and store it in deep geological formations.	DACCS	Yes (assuming low carbon energy and water generation).	6	Low - demonstrated by Sleipner CO_2 Storage Project	1000s years	No major obstacles.	High energy & water use.
	Chemical processes are used to extract carbon dioxide from the flue gas as biomass is burned. The CO_2 is then stored in deep geological formations.	BECCS	Likely: Net sequestration may be impacted by bi-directional changes in land carbon (depending on methods of biomass harvest). Land cover change may affect albedo, reducing mitigation effectiveness.	5-6	Low - demonstrated by Sleipner CO_2 Storage Project	1000s years	No major obstacles for the quantity of stored carbon but more difficult to account for any biomass harvest implications.	Potential implications on biodiversity, and for availability of biomass for other needs.

Table S1 – NETs, categorization and feasibility assessment. Red shading indicates high risk, yellow, medium risk and green, low risk. TRL: Technology Readiness Level. The assessment is based on multiple sources including references [29,45,52–55].

1.5 Summary of key assumptions

This section outlined the context and assumptions of the ZERs model. These can be summarised as follows:

1. 'Net zero' is considered to mean net-zero carbon-dioxide, methane (CH_4) and nitrous oxide (N_2O) emissions, where CH_4 and N_2O are quantified using GWP-100 (Section 1.2).
2. It is assumed that net zero must be achieved by 2050 to avoid the worst impacts of climate change (Section 1.2).
3. Biogenic carbon (and so the direct emissions from combustion of biomass and its sequestration in products) is generally not quantified in the model. It is assumed that carbon in emissions from biomass is balanced by carbon sequestration in growing biomass within the same year (Section 1.3). Three exceptions are made to this for;
    a. The production of methane (from waste and agricultural processes).
    b. Biogenic carbon transferred to permanent storage (as for Bio-Energy with Carbon Capture and Storage, BECCS).
    c. Biogenic carbon in fuels and feedstocks which could be made from a combination of biogenic and fossil feedstocks.
4. Land-use and land-use change emissions are not explicitly accounted for but are assumed to balance: there is no emissions nor sequestration from land-use included in the model, and any remaining deforestation or degradation must be balanced by equivalent regeneration or afforestation (Section 1.3.2).
5. The only form of Negative Emissions Technology (NET) included in the model is carbon dioxide captured in CCS, DACCS or BECCS in geological formations (Section 1.4).

2. The Zero Emissions Resources (ZERs) Model

This section expands on the outline of the Zero Emissions Resources (ZERs) model, given in the main paper.

2.1 Mathematical framework

The main framework is outlined in the main paper. The following diagrams and descriptions are supplemental to aid understanding.

2.1.1 The production matrix, P

Within the ZERs model, the system is represented by a production matrix, **P**, depicted by Figure S5. Each column of [**P**] represents an activity in the system, describing a "recipe" to transform substances or services into a primary 'output' (and by-products). The matrix $\boldsymbol{P}_{system}$ is square, has full rank, and has ones (positive or negative) on the leading diagonal – with each column (*j*) describing the activity that primarily produces (or removes) the substance *j* (as shown in Figure S5). The *j*th columns of $\boldsymbol{P}_{ext}$ describe the external inputs and outputs of the activity needed to create (or remove) substance *j*.

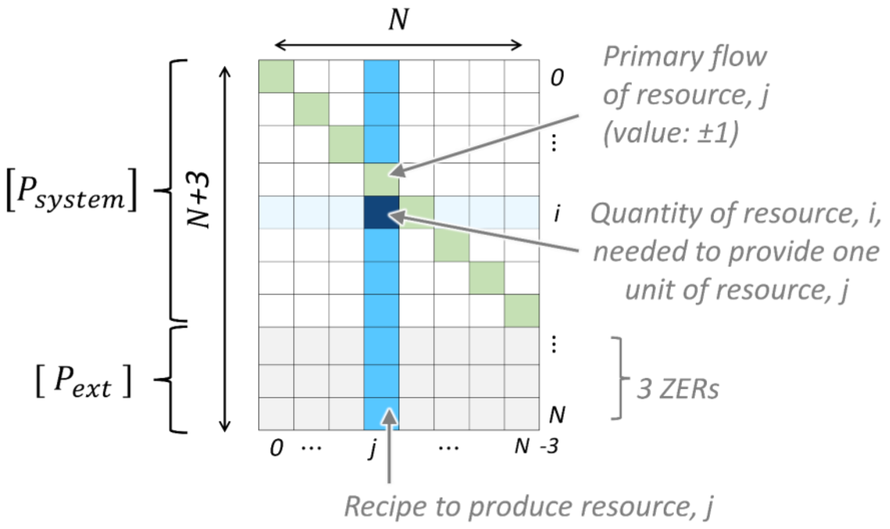

*Figure S5 - The production matrix (**P**) is a stacked partitioned matrix of 'system' ($\boldsymbol{P}_{system}$) and 'external' flows ($\boldsymbol{P}_{ext}$). In the basic current model the external flows are the three ZERs. Rows represent flows of substances while the columns represent the N service activity recipes, each of which produce one functional unit of the service.*

2.1.2 Managing residual and negative emissions

Within the model, the substance, *residual emissions,* appears in $\boldsymbol{q}_{final}$, and is the 'primary flow' of the removal process, *residual emissions management*. As an example, consider a system composed of only *car driving*, *residual emissions management* and *carbon dioxide management*. The *car driving* activity will run at the rate required to meet end-user demands of car transportation creating *residual emissions* as a by-product of fuel combustion. If the value for *residual emissions* in , $\hat{\boldsymbol{q}}_{final}$ is set to zero, the activity, *residual emissions management*, must run at the rate required to reduce *residual emissions* to zero by capturing the equivalent quantity of carbon-dioxide as the model substance *carbon-dioxide*. Provided the value for *carbon-dioxide* in $\hat{\boldsymbol{q}}_{final}$ is also set to zero, the activity *carbon dioxide management* then stores the gas as *captured carbon* in $\boldsymbol{q}^{+}_{ext}$. *Negative emissions technologies* and *carbon dioxide management* are treated as separate activities to allow the model to include Carbon Capture and Use (CCU), e.g. in synthetic fuel production.

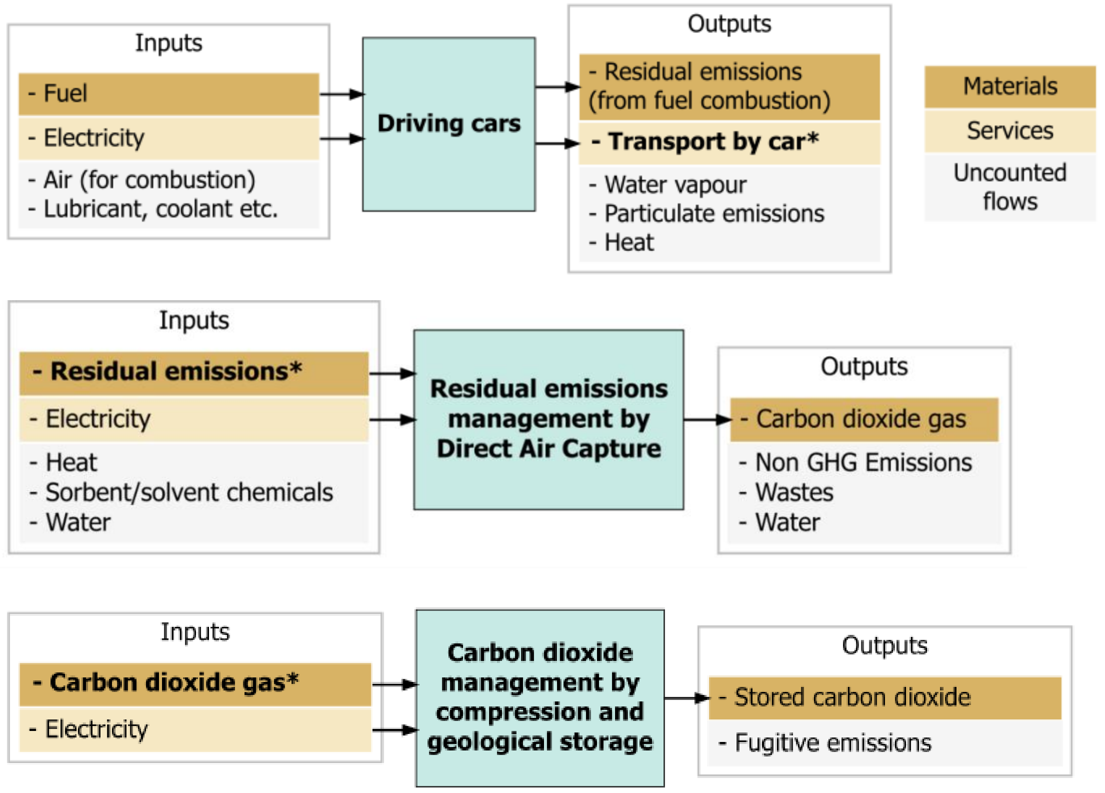

Figure S6 - Example of a simple three activity system. Each activity in the model is similarly characterised as a linear mapping between inputs and outputs. Only flows of materials associated with energy consumption are accounted for (in darker brown); if all material flows were accounted for, each activity could be mass balanced. The primary flow of each activity is labelled in bold, marked by an asterisk (*)

2.1.3 ZERs demand (the model output)

Demand for the three ZERs of non-emitting electricity generation, carbon storage and biomass is calculated by the model. *Biomass, non-emitting electricity,* and *carbon storage* all belong to the external block of **q** as no dedicated process exists to create or remove any of them as its main 'output'. *Biomass*, and *non-emitting electricity* appear in $\boldsymbol{q}_{ext}^{-}$ since the system draws on them, but *carbon storage* is an entry in $\boldsymbol{q}_{ext}^{+}$, since it is produced by the model as a by-product of *negative emissions technologies* and *managing captured carbon dioxide gas*. The quantity of *carbon storage* is calculated by setting *NetEmissions* to zero.

2.1.4 Scenarios without NETs

If a scenario has no options for Negative Emissions Technologies (NETs), the user cannot set a requirement for residual emissions – instead residual emissions will be determined by the rates of all emitting activities. The model can be adapted for this type of scenario by moving *NetEmissions* from an internal substance to an external substance (moving the row to $\mathbf{q}_{\text{ext}}$ and $\mathbf{P}_{\text{ext}}$), and by removing the relevant column from **P**. This setup is needed for historical scenarios used for model validation.

2.1.5 Managing multifunctionality

It is occasionally possible that so much of a *modelled resource* is produced as a by-product of other activities, to exceed the demand in $\hat{\boldsymbol{q}}_{final}$. The *primary activity* for that resource would then run in reverse with a negative value in s. Since this is physically impossible, we use an iterative approach for these cases to solve the model. Initially we remove the constraint that net production of this resource is set to zero, instead allowing it to be released from the system (as an entry in $\boldsymbol{q}_{ext}^{+}$) and added to external stocks; we therefore remove the associated *primary activity* for that resource. Having calculated the resulting activity rates (s is now a vector of length N-1), we check that there is sufficient flow of this service to meet the user demands, and if not, we re-introduce it to as an *modelled resource* into $\boldsymbol{q}_{system}$ and reintegrate its associated *primary activity* into $\boldsymbol{P}_{system}$. Similarly, a waste management activity could be a co-benefit of another activity and lead to managing more waste than is available in the internal modelled system; we can similarly allow the activity to manage (assumed unlimited) residual stocks of waste as an entry in $\boldsymbol{q}_{ext}^{-}$.

2.1.5.1 Accounting for service and material-production efficiencies

A categorisation of efficiency measures has been chosen to help identify, quantify, and fairly represent efficiency improvements in net-zero strategies. Reference[56] categorise efficiency measures dependent on the stage in the energy system but, since the ZERs model does not explicitly differentiate energy system stages (primary to final), only 'Service Efficiency' is relevant. Note that 'Services' described in the following section refer to the provision of both materials and energy services. Service Efficiency is defined by reference[57] as "the provision of a given task with less useful energy without loss of 'service' quality"; all the efficiency types identified here can therefore be considered a type of 'Service Efficiency'. Reference[58] further distinguish between passive system efficiencies and conversion device efficiencies. These labels are useful, but they are not sufficient to describe all energy savings measures identified in net-zero reports. Additional terms have therefore been assigned as listed in Table S2 on page 36.

In Equation 1 in the main paper, $\mathbf{q}$ is a vector of substance or service flows demanded by mitigation proposals, and $\mathbf{s}$ is the required activity rate to supply this. Wherever net-zero proposals include efficiency improvements beyond this, these are accounted by reducing the required activity rate to achieve the same level of service. The absolute values for desired rates of final substance and service flows, $\hat{\mathbf{q}}_{system}$, are replaced in Equation 1 by the ***apparent*** activity rates, $\mathbf{q}_{(apparent)}$.

The apparent flows vector, $\mathbf{q}_{(apparent)}$, represents the equivalent model activity rate required to give the required level of service, not the quantity of material delivered. The apparent activity rate accounts for the effective reduction in resource demands for the service/substance delivery from Device efficiency, Equipment efficiency, Material efficiency, Operational efficiency, Demand efficiency, and Sufficiency, as described in Table S2. This categorisation of efficiency is not in itself important here, except to determine which input needs to be adjusted. The distinction between $diag(\boldsymbol{\eta})$ and d is needed to quantify the actual quantity of service metric delivered.

To make the input values more comprehensible, $\mathbf{d}$, is further subdivided into a percentage change on 2018 values:

$$\mathbf{q}_{(apparent)} = \left(\mathrm{I}_j - diag(\boldsymbol{\eta})\right) diag(\mathbf{r})\, \mathbf{q}_{2018}$$

The interpretation of which category an efficiency measure belongs to is dependent on the choice of service metric. For example, in the current model framework, transport by car is given in the metric 'vehicle-km' and so increasing the number of people per car is a demand efficiency saving (fewer car-km will be needed overall) but were car-transport instead quantified in passenger-km, increasing the number of people per car would be a service efficiency, reducing the energy needed to provide each unit of service.

	Assigned term	Method to increase service from a given activity rate	Modelled by
Improve	Technology substitution efficiency	Substituting technologies<br><br>E.g. using heat pumps rather than gas boilers requires less energy at the point of use (final energy)	Adjusting the technology shares ($\boldsymbol{\alpha}$)
	Device efficiency	Improving 'energy conversion device' efficiency<br><br>E.g. A more efficient BEV motor reduces the energy demand to drive a car of a given size	Some efficiency gains have been accounted for in the model coefficients since they are based on lab-based testing, world best practice values, and future projections. Additional savings can be accounted for in the efficiency vector ($\boldsymbol{\eta}$).
	Equipment efficiency	Modifying 'the passive system'<br><br>E.g. Insulating homes reduces the final energy required to heat and cool them, or reducing the weight of cars reduces the final energy required to drive them.	Efficiency vector, $\boldsymbol{\eta}$
Shift (Changes in provisioning systems)	Operational efficiency	Changed use<br><br>E.g. Improved landing and take-off scheduling in aviation can reduce the time an aeroplane is in the air and reduce overall energy per flight.	Efficiency vector, $\boldsymbol{\eta}$
	Material efficiency	Reducing demand for material production<br><br>E.g. through more efficient utilisation, increased product lifetimes or reduced waste reduces associated production energy and emissions [59]	Demand vector, $\mathbf{d}$
	Demand efficiency	Reducing overall energy demand by changed service provision<br><br>E.g. Mode-shifting by taking the train instead of driving or increasing utilisation by increasing the number of people in a vehicle.	Demand vector, $\mathbf{d}$
Avoid	Sufficiency and reducing over-consumption	Reducing activity levels by considering which activities are needed to provide high levels of wellbeing in society, and through changed perceptions of desirable lifestyles.	Demand vector, $\mathbf{d}$

Table S2 - Efficiency and sufficiency measures which can increase the service provided by a given activity rate. These measures fall into the broad categories used by reference[60] of improving the efficiency of provisioning ("Improve"), shifting demand to more resource-efficient provisioning ("Shift"), and avoiding demand for goods and services ("Avoid"). These latter two groups are under-represented in climate policy but offer energy and emissions reductions potentials of 40-70% [61].

2.1.6 Quantifying model inputs

The model inputs, in terms of the final activity flows, $\mathbf{q}_{(apparent),final}$, are given in Part 6. These were derived from estimates of $\boldsymbol{\eta}$ and $\mathbf{r}$ from scouring documents of net-zero plans, as described in the main paper.

2.2 Survey and quantification of low carbon technologies

The ZERs model requires a self-consistent dataset of resource flows of all novel and conventional processes used in national and corporate net-zero strategies, spanning the breadth of activities in societies (from industry to transport to agriculture). An overview of the dataset is given in the methods section of the main paper. This section of the SI provides more detail on the dataset general assumptions and approaches. Specific details for each process are given in Part 5.

2.2.1 Dataset assumptions and approaches

The dataset aims to provide consistency across processes and sectors. This requires a consistent approach, set of assumptions, and values for key data sources such as emissions factors and energy density values. These are described in this section.

Basis for deriving process values

Where possible, the coefficients have been calculated based on the physical processes involved in providing the activity, but this was not feasible for some activities at the current level of aggregation; for example, production of textiles and chemical products, and food processing. In these cases which aggregate a wide variety of products or sub-activities, or which include complex process routes, values are calculated as top-down estimations of energy consumption and emissions production of the entire sector.

Where coefficients can be based on the physical processes, the values are based on predictions of feasible implementation at scale by 2050, or examples of best practice design. It is assumed that the model will be used for analysis at a global level, and so the coefficients are based on global averages.

Emissions Factors

Many process recipes across sectors have been derived by assuming the emissions are generated when fuels are burnt. For simplicity and consistency, the emissions factors are assumed to vary only by fuel (not by process), except for transport activities which use the fuels in Table S4. For all other processes, where the precise type of fuel is unclear, the values in Table S3 are used instead. Emissions factors used in this model have been chosen based on UK Government GHG Conversion Factors for Company Reporting [62] and IPCC Guidelines for National Greenhouse Gas Inventories [63]. The fuel labels in Table S3 are based on the groupings assigned for IEA world energy balances [64]. The model currently accounts for carbon-dioxide, methane and nitrous oxide emissions only (Section 3.1.2).

In general biomass has been assigned an emissions factor of zero, as described in Section 1.3.1. That section describes some exceptions where emissions or sequestration must be accounted. In these cases, the carbon flows are estimated by assuming the carbon content is around 0.5 Gt C/Gt dry biomass. Given the relative masses of Carbon and Oxygen atoms, this is equivalent to abating 1.83 Gt CO_2/Gt dry biomass in the growth stage and emitting the same value at the combustion phase. As such, the emissions factor for biomass combustion (where it used) is taken to be 1.83 Gt CO_2/Gt dry biomass.

IEA Summary Energy Balance Products	Assumed Mt CO2e/EJ
Coal and coal products	100
Peat and peat products	100
Oil and oil products	70
Natural gas and methane	60
Nuclear	0
Hydro	0
Geothermal	0
Solar/wind/other	0
Biofuels and waste [(a)]	0
Heat production from non-specified combustible fuels [(b)]	0
Electricity [(c)]	See note (c)
Heat [(b)]	0

Note (a) Biofuels and waste are assumed to be dominated by biomass energy
Note (b) Heat is assumed to be either from natural sources or use of otherwise wasted heat.
Note (c) Emissions arising from electricity generation are accounted for in the model service, Electricity Generation.

Table S3 - Fuel combustion emissions factors used within this document. Emissions factors are approximated from UK Government GHG Conversion Factors for Company Reporting [62] and IPCC Guidelines for National Greenhouse Gas Inventories [63]. The fuels considered are based on IEA world energy balances [64]. Processed natural gas is treated as synonymous with methane for this analysis although it is typically closer to 95% methane by molar composition [65].

Explicit fuels or processes	Assumed Mt CO2/EJ
Diesel	71
Petrol	75
Marine fuel	77
LNG	57
Kerosene	72

Table S4 - Fuel combustion emissions factors used for specific activities in the model where values used differ from those in Table S3. Emissions factors are approximated from UK Government GHG Conversion Factors for Company Reporting [62] and IPCC Guidelines for National Greenhouse Gas Inventories [63].

Gravimetric Energy Density Values

Energy density values are based on Gross Calorific Values (GCV) on the assumption that energy in water vapour is generally not recovered. The values used are given in Table S5.

Energy carrier	Energy density	Unit	Reference
Biofuel	35	GJ/t	UK Government Conversion Factors give values from 27 (for bio-ethanol) to 44 (for Hydrogenated Veg Oil, HVO bio-diesel) GJ/t
Biomass	15	GJ/t	Based on assumed carbon content of 50%
Coke and coal	28	GJ/t	UK Government Conversion Factors
Diesel	43	GJ/t	UK Government Conversion Factors
Hydrogen	120	GJ/t	Giddey et al. (2013)
Kerosene	44	GJ/t	UK Government Conversion Factors
LNG	45	GJ/t	UK Government Conversion Factors
Marine fuel	41	GJ/t	UK Government Conversion Factors
Natural gas and methane	45	GJ/t	UK Government Conversion Factors
Oil	43	GJ/t	UK Government Conversion Factors
Petrol	45	GJ/t	UK Government Conversion Factors

Table S5 - Energy density values used within this document. Values are approximated from UK Government GHG Conversion Factors for Company Reporting [62], and (Giddey et al., 2013). Processed natural gas is treated as synonymous with methane for this analysis although it is typically closer to 95% methane by molar composition [65].

Error checking

Coefficients used for individual processes have been compared against other sources where possible to increase confidence that they are representative. Ideally the model would be checked for errors using mass and energy balance approaches but, since the model does not include all material resources, a full mass-balance verification is not possible. Substances are quantified in mass terms as far as possible, however, to facilitate simple 'sense-checks' at a process level. There are some examples where the processes should not be mass-balanced within the current model framework, even if all substances were quantified:

- Since emissions are measured in units of tCO_2e, if a process produces methane or nitrous oxide emissions, the flows will not be mass balanced. The mass outflow of these gases is multiplied by their global warming potential (GWP) to give the equivalent mass of CO_2e.

- In some cases, 'dummy' mass flows are used so that diverse substances can be grouped for quantification. The ZER Biomass, for instance, is quantified as a mass input for Plant Agriculture and Forestry although the actual mass inflow is water, carbon and other chemicals. Quantifying the 'dummy' mass metric allows all biomass use to be aggregated into one metric but duplicates the 'real' flows.
- Since biogenic carbon is not explicitly accounted in the model, processes which create emissions from or sequester biogenic carbon, may not be mass balanced (as described in Section 1.3.1).

2.3 Extending the model

The model can be easily extended to include new activities, processes, substances/services, and zero-emission resources (ZERs). This section outlines the steps required to add new elements to the model. The mathematical variables referred to in this section are described in Section 2.1.

Adding new internal or final substances and services

The addition of a new internal or final substance or service to the model requires the addition of a new activity to produce it. This would grow the production matrix to include a new row in $\boldsymbol{P}_{system}$, describing flows of the new substance or service, and a new column in $\boldsymbol{P}$, describing the resource flows needed to produce it. The model inputs $\boldsymbol{q}$ and $\boldsymbol{\alpha}$ would also increase to include the demand and process shares for the new activity. The following steps are required to add a new substance or service to the model. Although these steps are described in order, there is likely to be some iteration between them.

1. **Determine whether providing the new substance or service is already accounted for within an existing activity.**
   a. Where the new substance is already included within another, the original activity and substance/service must also be modified. For example, adding meat-based food as a separate substance requires subdividing the existing resource *RawFood* into *MeatBasedRawFood* and *NonMeatRawFood*, and the activity, *FarmingFood*, into *FarmingMeat* and *FarmingNonMeat* to produce *MeatBasedFood* and *NonMeatFood*, respectively. Any process

which draws on (or produces) the new substance or service, must also be modified to account for the new substance or service. For example, the process *FoodProcessing* would need to be modified to account for the new substances *MeatBasedFood* and *NonMeatFood*.

b. Where the new substance or service is not already included with others in the model, the new substance must be added to all processes which might produce or consume it. For instance, adding the substance *Quicklime* to the model requires a new activity, *Quicklime production* to produce it. The substance *Quicklime* must also be added to all other activities in the model which consume it, such as production of aluminium, cement, and steel.

2. **Choose a metric and unit for the activity.** For the Quicklime example (in 1a), this would be Gt Quicklime. Where possible the metric should be Gt (in mass terms), consistent with other mass flows in the model and reasonable to describe global flows of bulk materials. In some cases, the output cannot easily be expressed in mass terms. In those cases the metric may be chosen to best reflect the role of the activity in providing for human needs, or to be easily comparable to available data.

3. **Identify the activity process boundaries and draft a flow diagram** of the inputs and outputs (see Fig 3 of the main paper). This will help to identify and avoid any potential overlaps with other activities.

4. **Quantify the demands** of other model substances to provide one unit flow of the activity rate metric - the process coefficients. For the *Quicklime* example, this would be the fuel and emissions required to produce one Gt of Quicklime. The coefficients should be based on best practice design or predictions of feasible implementation at scale by 2050, using the assumptions described in the previous sections.

   a. If the given process can be described by physical models or broken into sub-processes, estimate the mass and energy flows for each sub-process and sum these to find the total accumulated demands per unit flow of output. These estimates may be derived from simple calculations, or models, or taken from other studies (life-cycle analysis or techno-economic assessments, for instance). Other studies may give values which cover the whole process,

rather than sub-processes. These studies may also be used, provided the boundary conditions and assumptions are consistent with this model.

b. Some processes cannot easily be considered as sub-processes or bottom-up physical models because they aggregate a wide variety of products or sub-activities, or include complex process routes. Current examples include production of some chemicals, and agricultural and mining processes. In these cases, a top-down approach can be used instead; coefficients for existing technologies can be estimated from global or regional energy and mass flows, while new technologies can be estimated by considering the relative change from current technologies.

Adding new delivery processes

New delivery processes can be added to existing activities by following step 4 above. In this case, the dimensions of the production matrix, $[P]$, and the demand vector, $\boldsymbol{q}$, do not change. But an additional column is added to the coefficient matrix, $[\Gamma]$, describing the resources required to produce the given activity by the new delivery process. An additional input is needed in the delivery process share vector, $\boldsymbol{\alpha}$, to represent the share of the activity provided by the new process.

Adding new ZERs

The model can be extended to quantify the aggregated demands of other substances or services of interest, such as nickel, rare earth metals or water. This is done by introducing a new Zero-Emission Resource (ZER) - a substance or service which is treated as external to the model system, and so a model output. Introducing a new ZER is similar to introducing a new internal substance or service, except that there is no new activity (or delivery processes): all other processes which draw on the new ZER must have a new coefficient to quantify the new flow. In mathematical terms, another row is added to P_{ext} to describe flows of the new ZER, and the model output, $\boldsymbol{q}_{ext}$, will include an additional term (the aggregated demand of the new ZER).

For example, to include freshwater as a ZER, every process which would use or produce freshwater must have a new coefficient to quantify the water flow. The new

row in P_{ext} represents flows of freshwater. The aggregated demand for freshwater then becomes a model output in $\boldsymbol{q}_{ext}$ which can be compared against likely availability.

3. Estimating probable future supply

Trajectories of probable future supply of the three ZERs are described in the main paper. This section provides additional evidence to support those trajectories. Their uncertainty is considered in Section 4.2.

3.1 Estimating probable future supply

The definitions for Zero Emissions Resources (ZERs) (Section 3.1.1) and the analysis approach used in this work differ from other studies: here the approach is cautiously ambitious but aims to minimise risk inherent within the supply trajectories by quantifying maximum *probable* supply rather than maximum feasible supply (Section 1.1). The aim is to quantify the maximum supply which we can rely on. The current and future probable supply of ZERs have therefore been estimated specifically for this study. Each ZER is considered in turn in the following sections, concluding with quantified scenarios of historical and future supply.

Acknowledging that there is significant uncertainty in these scenarios, a range of supply is also given in Section 4.2.

3.1.1 Zero Emissions Resources (ZERs) definitions

In the current assessment we are interested in three ZERs - Non-Emitting Electricity (NEE), biomass and carbon storage. They can each be interchanged but any change in choices of technologies and energy carriers would simply redistribute demand between the three. While there are clearly many other critical resources and services whose supply may be constrained in future scenarios, these could be added to the model in future work and are not the focus of the current assessment.

Although biomass and electricity generation can be easily defined and quantified, there is often ambiguity how carbon storage is quantified – for example, they may not explicitly state whether carbon capture and use (CCU), NETs (DACCS and BECCS) or CCS from industrial flue gases are included. For this work, carbon capture is the combination of two processes: (1) capture of carbon dioxide gas - either from the atmosphere (by Direct Air Capture, DAC) or from a flow that would otherwise increase atmospheric CO_2 (such as by Carbon Capture of flue gases in industry), - and (2)

compression and injection into deep geological formations, where it is stored with low leakage.

CCU (Carbon Capture and Use) is not counted as carbon storage because the carbon dioxide is later emitted to the atmosphere, for example in production of synthetic fuel which is later burned (Part 5). In contrast, EOR (enhanced oil recovery) can be included. In Enhanced Oil Recovery (EOR) carbon dioxide gas is injected into an oil seam to reduce the viscosity of oil and increase the fraction of oil which can be recovered from a given oil field [66]. Although some CO_2 is brought to the surface with the extracted oil, it is mostly recycled and reused to minimise the costs of oil extraction. According to Hill, Hovorka & Melzer (2013), venting and fugitive emissions in EOR “amount to a few percent or less of the purchased volumes of CO_2”. This means that most of the CO_2 injected remains underground and, although the EOR leads to fuel burn emissions downstream, the CO_2 injected can be counted as carbon storage.

The following definitions are therefore used to account for the Zero Emissions Resources (ZERs) within the model:

- Biomass is accounted as the dry weight of organic plant matter, used to supply services to humans (see Figure S17). Note that the mass of carbon, approximately half the dry mass of biomass, is often used in other studies.
- Non-Emitting Electricity (NEE) is defined as gross electricity generation, where gross operational emissions are zero. Nuclear and renewable power is therefore included in this definition although biomass-fuelled, BECCS and other generation with CCS are not.
- Carbon Storage is accounted as the mass of carbon dioxide gas placed in long-term geological storage. Carbon dioxide which is captured and then released for other uses is not included (i.e. CCU is not included in this value). Carbon stored in other reservoirs (for example temporary storage as biogenic carbon) is not considered to be a valid alternative to near-permanent storage and so is not included, as justified in Section 1.4.

The following sections provide additional detail to support the trajectories of probable future supply of these three ZERs, used in the main paper.

3.1.2 Non-Emitting Electricity generation (NEE)

The future supply of NEE is based on consideration of historic growth rates and industry projections. The trajectory is derived by considering each generation technology in turn which are then aggregated.

Solar and Wind

Globally, solar and wind technologies are still at a relatively early stage of the technology adoption cycle and are therefore expected to follow an ‘s-shape’ growth trajectory. This is sometimes modelled by backcasting using a logistic function with an assumed maximum capacity or generation at a target date. Since this approach is not based on physical realism, we instead fit an s-shape curve to historical empirical generation data. This approach was used by Cherp et al. (2021)[69], who fitted both Logistic and Gompertz models to historical data for solar and wind generation in 60 countries with the largest electricity systems. They found this approach at a global level matched empirical data with considerably higher accuracy than the more optimistic logistic curve fits generated by Grubb et al. (2020)[70].

Following the approach of refernce[68], the trajectory of wind and solar generation to 2050 is estimated using a Gompertz model fit on historical global generation data to 2022. Projections from curve fitting can be very sensitive to the functional form [71]. Reference[68] consider alternative models but argue that Gompertz model may be more suited to technologies originally introduced by policies, such as solar and wind because it is asymmetric with longer growth after the inflection (in contrast to the symmetric logistic model). As detailed by reference[68] for this application, the Gompertz function is represented by:

$$Gompertz\ model, f(t) = Le^{-e^{-k(t-t_0)}}$$

> *“Where e is a constant approximately equal 2.718, k is the growth constant and t_0 is the inflection point. In both models, growth accelerates until it reaches the inflection point, at which it is nearly linear. After the inflection point, growth begins to slow. For the logistic curve, the inflection point is located at the 50% of the asymptote L; for the Gompertz curve, the inflection point is located at 37% of the asymptote L.”* [68]

The projection used here has been updated from that published by reference[68] to account for unexpectedly higher generation in 2021 and 2022, producing around 5% and 10% greater generation than projected for 2021 and 2022, respectively. The projection has been updated because the full dataset significantly affects the estimated 2050 generation, as shown in Figure S7.

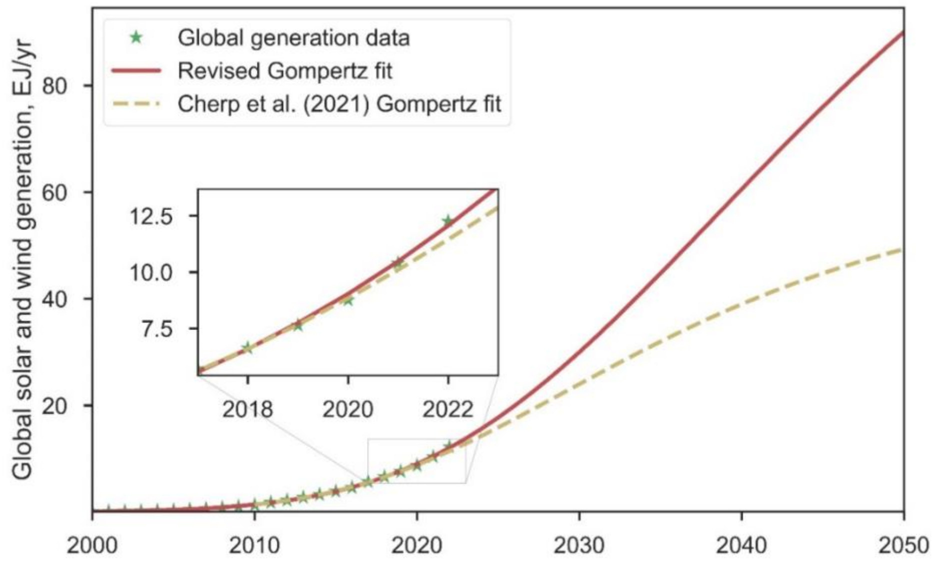

Figure S7 - Comparison between the solar and wind electricity trajectory based on data to 2018 [68] and updated Gompertz fits against empirical generation data to 2022, revised for this study. The revised fit was calculated using the 'curve fit' function of the SciPy 'optimise' module for Python.

[68] used Residual Sum of Squares (RSS) values to compare the accuracy of the fits to empirical data. Updating estimates of the RSS values to account for 2021 and 2022 data points (Table S6) shows that, given the recent growth data, the revised projection used in this study is more suitable than the original projection used by reference[68].

Technology	Empirical data end date	Original RSS	Revised RSS
Solar	2018	700	800
Solar	2022	17000	2400
Wind	2018	2000	3000
Wind	2022	17000	7800

Table S6 - RSS values for the original solar and wind projections by reference[68], based on data to 2018, against the revised projections, based on data to 2022. Although the RSS values are higher for the revised projections when the value is calculated based on data to 2018, RSS values based on data to 2022 are considerably lower than the original projections, suggesting higher accuracy. RSS values have been rounded to the nearest 100 for clarity. Note that the values shown for the original projection are slightly higher than given in the original study due to small differences in the empirical data used.

The additional generation above that expected by reference[68] can be explained by the unprecedented growth of solar and wind in China (Figure S8). Chinese generation has more than compensated for the discrepancy between global generation predicted by reference[68] and actual generation for 2021 and 2022; Chinese additional generation also compensates for shortfalls against predicted generation in other parts of the world (Figure S9).

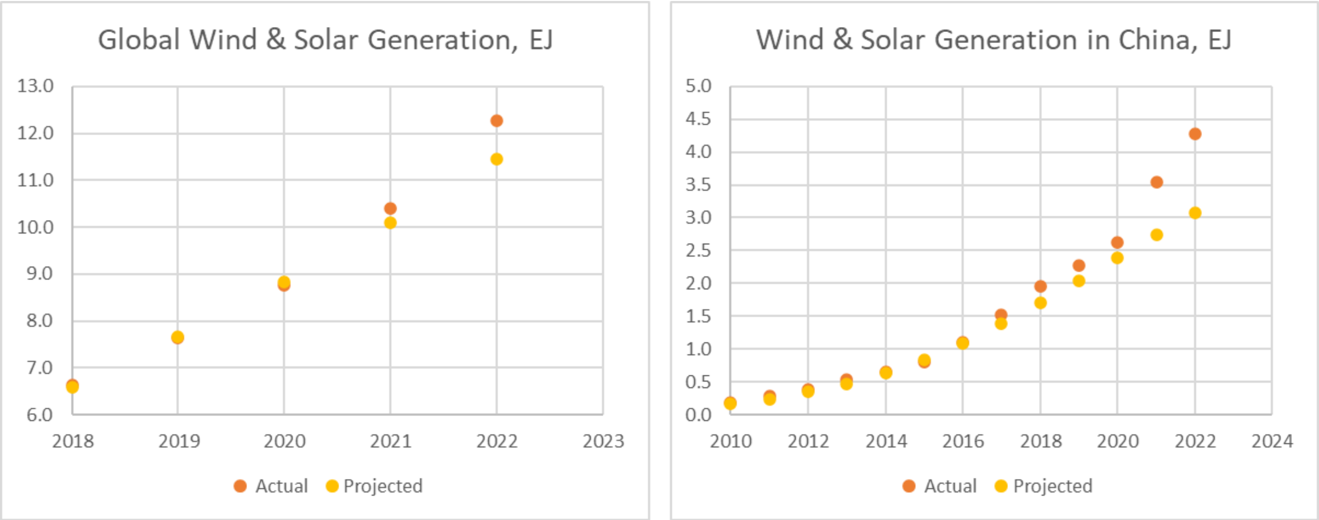

Figure S8 - Actual and predicted generation from solar and wind between 2010 and 2018. Predicted generation by Cherp et al. (2021) is compared against data from Ember yearly electricity generation data (Ember, 2023).

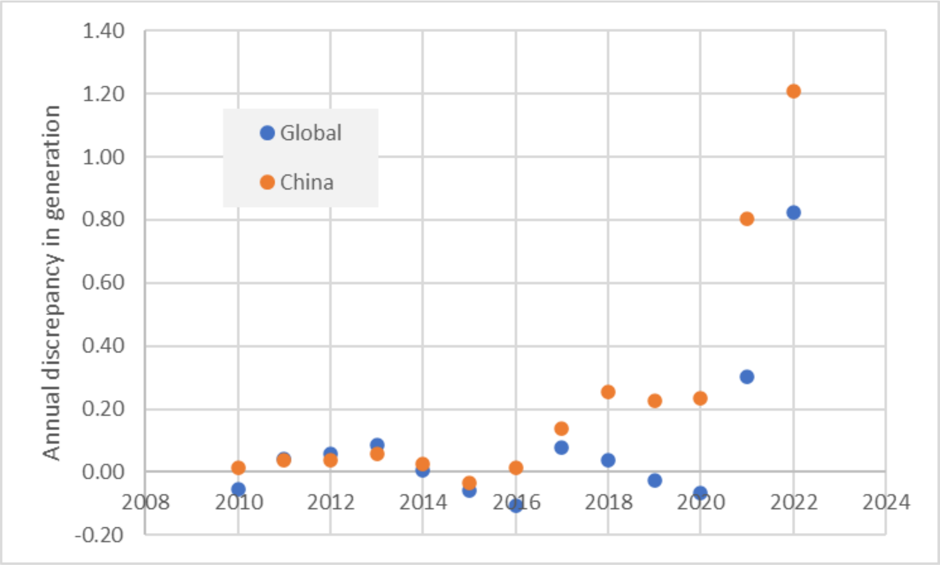

Figure S9 - Discrepancies between actual and predicted generation from solar and wind between 2010 and 2018 for Chinese and global generation. The discrepancy is calculated as

the difference between predicted generation by reference[68] *and Ember yearly electricity generation data (Ember, 2023).*

Nuclear, Hydroelectric and Geothermal Electricity Generation

Nuclear, hydro and geothermal electricity generation by 2050 are estimated based on historical growth rates and industry reports assuming linear growth:

- The International Atomic Energy Agency (IAEA) consider global nuclear generation retirements and additions over the coming decades to project a potential range of generation in 2050 between 12 and 25 EJ/yr [73]. As shown in Figure S10, nuclear generation is assumed to be midway between the IAEA's bounded estimates, and consistent with the recent trend.
- Hydropower is similarly extrapolated from the approximately linear trend given in the IEA Special Market Report on Hydropower [74] which projects growth from 16 EJ/yr in 2020 to 19 EJ/yr in 2030 (Figure S11).
- Geothermal generation makes up only 1% of all NEE today [75]. Its growth rate is assumed to continue at the approximately linear rate of 0.01 EJ per year, observed since 2010 (Figure S12). Next-generation geothermal is not included in the maximum probable supply projection.

It has been proposed, for example by the reference[76], that deployment of next-generation geothermal could dramatically increase the installed geothermal capacity (for electricity generation). These new technologies use techniques from the oil and gas industry to make geothermal electricity (and/or heat) available at greater scales and over much wider areas than conventional geothermal technologies, which are constrained by requiring access to suitable naturally occurring hot water reservoirs [77]. There are two dominant approaches: Closed-Loop Geothermal Systems (CLGS also sometimes referred to as Advanced Geothermal Systems, AGSs) and Enhanced Geothermal Systems (EGS). EGS is the more mature technology but there are only around 5-15 current commercial EGS projects worldwide, with capacity of the order of 0-1 EJ/yr, according to a briefing report compiled by the World Resources Institute [77]. CLGS are only in the initial demonstration stage (WRI, 2024). Supply in the coming decades will be limited by project development timescales and constraints on deployment, including financing, site-surveys and long permitting processes (WRI, 2024) and so has not been included in the maximum probable supply projection. EGS is, however considered in the more optimistic projection, described in Section 4.2.

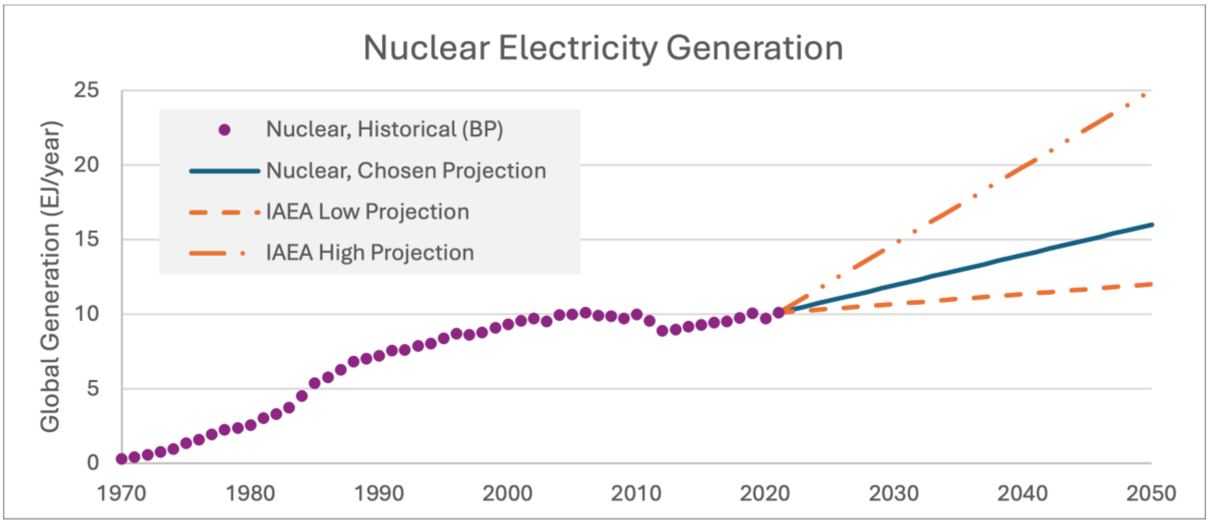

Figure S10 – Historical and future projections for nuclear electricity generation

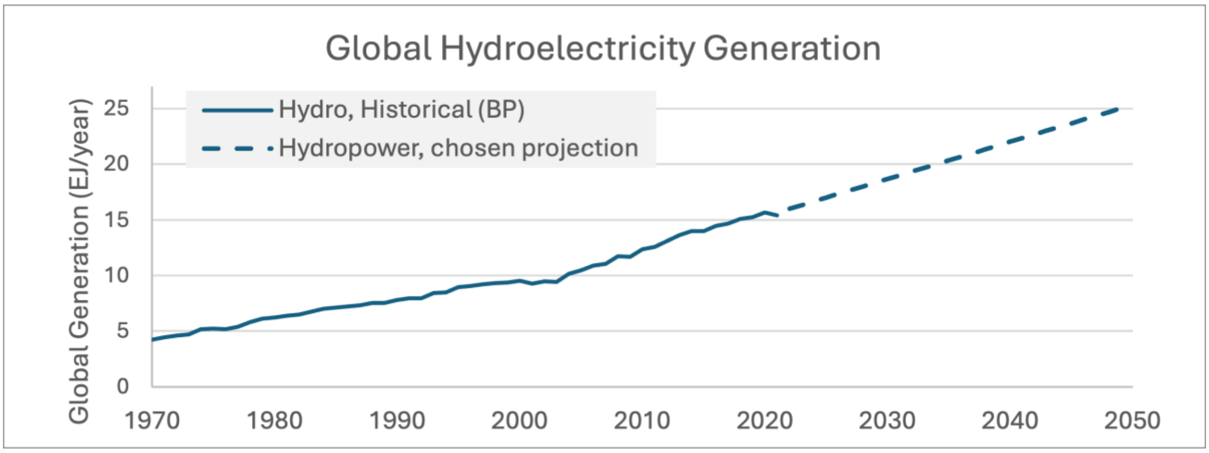

Figure S11 - Historical and future projections for hydro electricity generation

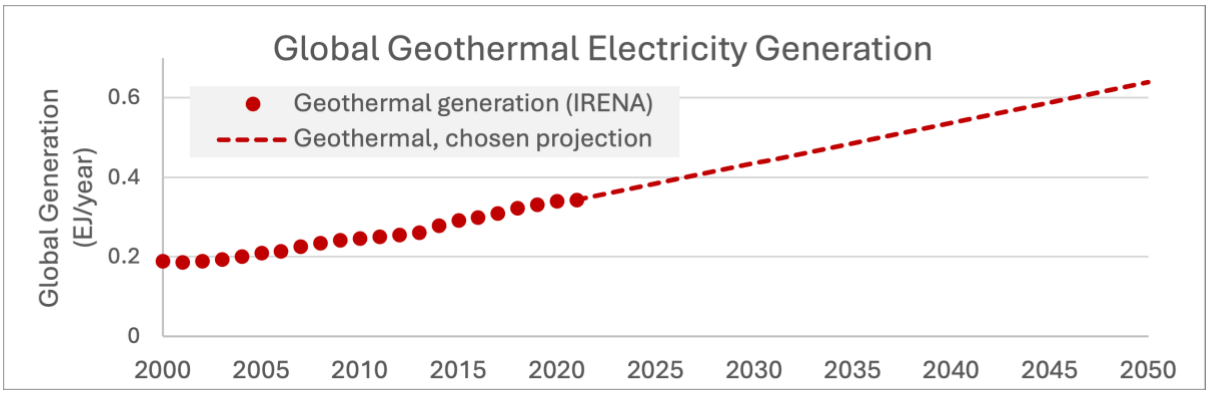

Figure S12 - Historical and future projections for geothermal electricity generation

Summary: Non-Emitting Electricity

Non-Emitting Electricity (NEE) supply is forecast as the sum of individual generation technologies (wind and solar, nuclear, hydropower and geothermal generation). Consistently with historical growth rates and industry reports, the nuclear, hydro- and geothermal electricity generation assume linear growth, while wind and solar generation is based on a Gompertz model fit, following the approach of Cherp et al. (2021). The total NEE trajectory is shown, alongside that for Carbon Storage and Biomass in Section 3.1.5.

3.1.3 Carbon Capture and Storage (CCS)

CCS is an immature technology which has shown relatively slow growth since the 1970s. Historical trends may underrepresent future availability, however, if the consistency of incentives and regulatory environments improve with the increasing urgency of climate change [48]. This appears consistent with recent data, at least at the planning stage - reference[78] found evidence of a second wave of planned deployment from 2018, which would account for an eight-fold capacity increase by 2030. There are three factors opposing this view: the distribution of applications, financing, and the need for dedicated storage.

Firstly, the distribution of applications must change to be compatible with net-zero proposals. Although net-zero proposals rely on using CCS across industry sectors, data from the reference[79] shows that 70% of current capacity is used to remove CO_2 from natural gas streams during extraction and there is only one commercial example of an application in heavy industry (Abu Dhabi CCS iron and steel production, accounting for <2% total capacity, 0.8 Mt CO_2/yr). The application of CCS from one industry to another is not necessarily straightforward - concentration of CO_2 in natural gas processing streams is relatively high compared to other applications which reduces cost and complexity [80]. Secondly, it is not clear how future projects will be financed, given most to date are funded by Enhanced Oil Recovery (EOR); future projects cannot rely on this revenue stream. Lastly, future growth will need dedicated geological storage, developed for only a minority of operations to date (Figure S13). This could be an obstacle to investment and cause delays to deployment [81].

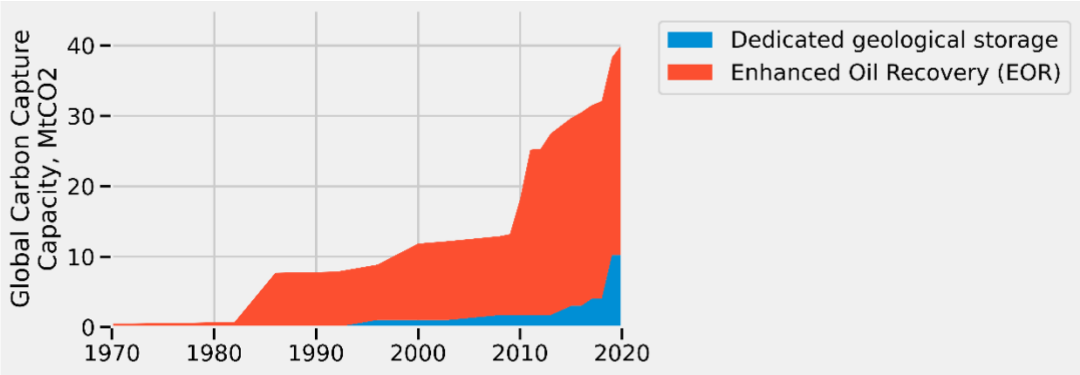

Figure S13 - Historical CCS capacity. Data is from the reference[79].

Perhaps due to this complex landscape, there are few forecasts of CCS capacity growth. Many quantified values for CCS in 2050 are instead '*backcasts*', based on the estimated demand rather than the expected infrastructure deployment. For instance, The IEA's Net Zero by 2050 scenario (IEA, 2021b) assumes global growth to around 1.7 Gt/yr by 2030 and 7.6 Gt/yr by 2050. This trajectory would require capacity by 2030 to be seven times larger than the total planned capacity in the GCCSI database. Given project lead-times of around 8 years [81], it seems extremely unlikely that this growth could be achieved. Taking the opposite approach, Grubler et al. (2018) simplify the problem of estimating future CCS availability by excluding it entirely in their low energy demand scenario – they justify this given the risks of "innovation failure, unacceptable investment risks, and public opposition."

A reasonable approach to estimate probable future growth would be to develop a bottom-up analysis, as suggested by reference[81], using an approach such as agent-based modelling which could consider regional potential for financial investment and policy support, regional storage capacity, and uncertainties around injection dynamics. In the absence of this model, historical data can provide some evidence of plausible future growth.

Forecasted projections of CCS are mostly based on two approaches, either by analogy to other technological transitions, or by extrapolating (the limited data) on historical growth of CCS and Enhanced Oil Recovery (EOR). Van Ewijk & McDowall (2020) use the first approach, estimating potential diffusion of coal powered electricity generation with CCS using the historical analogy of flue gas desulphurization (FGD). They argue that both (as "end-of-pipe abatement technologies") are likely to be regulation enforced and "have important similarities regarding economic and financial viability,

politics, policy and regulation, and costs and scaling dynamics". As the authors acknowledge, there remains a significant difference between the two technologies, since the transportation and storage of carbon dioxide is very different to sulphur dioxide (the product of FGD). This means the approach is not suitable for this work, where the aim is to quantify the *storage* rather than the *capture* potential alone. As reference[81] argue "storage development is and will remain a rate-limiting step on CCS deployment". Other potential historical analogies, such as oil and gas extraction, may more fairly represent the physical and geological challenges but would not represent the lack of financial incentives and other important aspects of CCS.

In the absence of a representative historical analogy, historical CCS and EOR data may be used to extrapolate future growth, but there is generally not enough data to justify the chosen shape of the growth trajectory. Reference[85] use logistic growth models (s-shaped curves) - widely used to describe the growth and decline of resources such as oil and coal, trends in energy systems, infrastructure, and technology development - but and references[86,87] use exponential growth, assuming that growing numbers of countries will adopt financial incentives like those offered in the US. These models predict 2050 annual capacity in the same order of magnitude, as shown in Table S7.

Reference	2050 capacity ($MtCO_2$/yr)	Notes on projection
[88]	700	Excluding CO2 from natural sources, including EOR
[85]	430-1500	Based on scenarios with growth rates between 8.6% and 12.1%, and 2100 cumulative storage targets between 348 and 1218 Gt CO2
[86]		Considering the history and plans for dedicated geological storage (as opposed to EOR)
	75	Based on a projection of 1970-2020 project capacity
	300	Based on storage plans for 2020-2030

Table S7 – CCS projections from historical and planned data.

None of these projections explicitly consider the effect of the interlinked and self-reinforcing hazards to the pipeline of future projects: project completion delays and cancellations; low-capacity factors of operational facilities; and uncertainties & limitations of future financing and regulation. To make a more realistic estimate of operational CCS in 2050, the extent of these constraints has been considered in the

remainder of this section, aiming to reconcile their effects with the existing projections.

Project completion delays and cancellations

Historical global growth rates have not matched up to expectations, particularly in the 2010s when there was particularly high attrition rate of projects (GCCSI data) and, as noted by Scott (2013), several "flagship projects" were cancelled at a late stage in development, including AEP's Mountaineer (West Virginia, USA), ZeroGen (Queensland, Australia), 2Co's Don Valley (Doncaster, UK) and Scottish Power's Longannet (Fife, UK). This period corresponds to the 'first wave' of CCS projects, identified by reference[78], which was found to have a failure rate[2] around 90%.

(Townsend & Gillespie, 2020)Global CSS Institute (GCCSI) (2022)Martin-Roberts et al. (2021) also compare historical planned projects against the actual development history and find that, in addition to large attrition rates, many of the operational projects in 2021, were not amongst those planned in 2009. They attribute this to inconstant CCS policy and funding. Although the historical failure rate could be reduced if CCS policy and funding become more consistent, reference[86] argue that project completion would take longer in new countries where interests are poorly aligned to climate mitigation, even where there is high storage potential (e.g., Saudi Arabia), and even longer in countries without a mature hydrocarbon industry, such as India.

In summary, the current record indicates that it would be a high-risk strategy to assume that stated plans will reflect future capacity. Case studies of performance in operational plants tell a similar story.

Capacity factors of operational facilities

Although there is no publicly available data to quantify actual rates of CCS at a global scale, there is evidence that actual storage rates have been significantly below the listed capacities in databases (the maximum annual storage rates) . Case studies show this may be due to a combination of economics, technology issues, and safety concerns [90].

[2] Defined as the capacity of failed projects over the capacity of planned projects over the period.

The published capacity may also be higher (or lower) than the average lifetime value of the facility because only the current maximum storage rate is listed. This means that in cases of facility upgrades, such as Great Plains Synfuels Plant where the nominal capacity of the facility was increased from 2-3 $MtCO_2$/yr in 2006 [90], only the higher value is shown.

The capacity factors of eight large-scale CCS projects are estimated in Table S8. The capacity factor for this work is defined as the actual annual CO_2 stored, over the published maximum annual storage rate of the facility. These eight projects account for around 45% current global nominal capacity and 65% total global carbon storage (as inferred from the nominal capacities). Comparing these case-studies, annual storage of CO_2 may have been around 60% of the full annual capacity (as shown in the case studies in Table S8). Although we may be able to improve capacity factors through learning, future CCS rates must necessarily be lower than the total capacity.

Site name	Application	Nominal Capacity, $MtCO_2$/yr	Operational date	Capacity factor	Notes	Source
Shute Creek	Natural Gas Processing	7	1986	50%	The plant originally had capacity for 4.8 $MtCO_2$/yr but increased capacity in 2010 to 6-7 $MtCO_2$/yr. 54 Mt CO_2 were planned to be vented but 66 Mt were originally planned for EOR but unsold due to economic conditions.	Robertson & Mousavian (2022)
Sleipner	Natural Gas Processing	1	1996	82%	15.5 Mt were injected from project start (1996) to June 2015. Note that the lower stated capacity in the reference of 0.9 $MtCO_2$/yr implies a higher capacity factor of 90%.	[91]
Great Plains Synfuels Plant	Synthetic Natural Gas	3	2000	63%	Capacity was increased from 2 $MtCO_2$/yr to 3 $MtCO_2$/yr in 2006 and actual carbon capture has been approximately constant through the lifetime at ~1.9 $MtCO_2$/yr, capturing 38 Mt CO_2 for geological sequestration by 2020	Robertson & Mousavian (2022)
Snøhvit	Natural Gas Processing	0.7	2008	82%	"Since commissioning in 2008, Snøhvit has captured more than 7MT of CO_2, averaging 0.55–0.6MT annually". CO_2 capture rate has been consistently high; although there was less CO_2 captured in earlier years; this was due to lower extraction rates of natural gas.	Robertson & Mousavian (2022)
Boundary Dam	Power Generation	1	2014	60%	Averaged 0.615 Mt/pa actual (2015-2021)	Robertson & Mousavian (2022)
Quest	Hydrogen Production	1.3	2015	76%	Delivered 1 $MtCO_2$/yr in first 5 years. Note that Quest project documents quote 1.1 $MtCO_2$/yr capacity which would indicate a higher capacity factor of around 90%.	Robertson & Mousavian (2022)
Illinois Industrial Carbon Capture and Storage	Ethanol Production	1	2017	52%	Capture was underperforming by 48% between 2017 and 2020	Robertson & Mousavian (2022)
Gorgon	Natural Gas Processing	4	2019	33%	5 Mt was actually injected against a target of 15 Mt. The project had a three-year delay to operations (was originally planned to begin CCS in 2016). Australian regulators reduced injection rates in December 2020 for safety reasons.	[92] [93]

Table S8 – A selection of operational projects for which there is data available to estimate the overall capacity factor of operation. Nominal capacity and operational date are as recorded in GCCSI database. Capacity factor is calculated as (actual captured/ capacity).

Future regulation and investment

Deployment of any technology by the private sector requires there to be some degree of certainty in the potential for future revenue. Without this there is no business case for private investment, and the only growth could be in publicly owned technology. Currently there is no viable revenue stream associated CCS for the purpose of Carbon Dioxide Removal (CDR). However, even if stringent carbon pricing were introduced, investment in storage projects will require substantial state financial backing to overcome the breadth and scale of uncertainties in the CCS industry. As outlined by reference[81], investment into carbon storage projects requires confidence in the:

- Storage site characteristics – that it can maintain an economically viable injection rate for long enough to justify the investment, without inducing any geo-mechanical or environmental hazards;
- Long-term security of the CCS value chain – that CO_2 will be available for storage at a predictable cost;
- Public acceptance of continued future operation; and
- Scale of post-injection liabilities.

Investors therefore face a trade-off between potential future revenue and increased up-front costs. Increasing the sequestration target (and potential returns) of a project requires greater investment risk, which can be reduced by increased pre-emptive investment on site-specific data and modelling. With high uncertainty in the rate of returns, this high up-front spending is unattractive for private investors. True storage will thus require state financial involvement. This is consistent with past experience. Considering CCS-analogous technologies, reference[94] conclude that their growth and diffusion have depended on government support, and the credibility of industry incentives. Similarly, reference[95] observe that public funding has dominated CCS investment in the past, and demonstrate, using global CSS project data, that government ownership significantly reduced the risk of project failure.

Given future capacity expansion will depend on government and regional funding, we could expect the capacity growth curve to be a function of the number of countries with an involvement in CCS. Figure S14 shows the increasing number of countries with either an operational facility or a stated plan to develop one. Before 2011, only 3

countries had built a CCS facility since the first in 1972. Since then, however, there has been a steady linear increase in the number of involved countries. There is an apparent planned increase in the rate of new country involvement after 2021 but, given the historical evidence of project delays (page 56), this is likely to be reduced. It is therefore assumed that the current linear growth in involved countries continues.

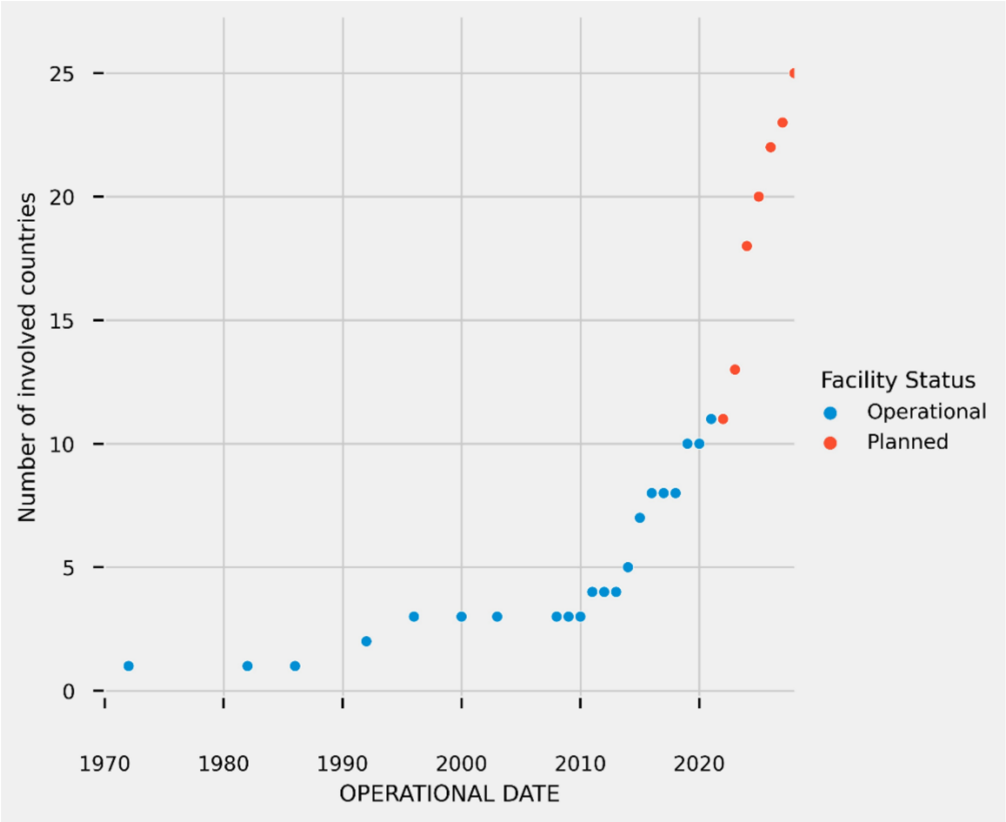

Figure S14 - Number of countries with at least one commercial CCS facility by year. Past data is shown in blue while red shows future planned facilities. Data is from Status of CCS 2022 (GCSSI, 2022).

Most new countries to the market are unlikely to be able to invest in more than one facility initially. Sometime after the first begins operation, however, they are likely to build at least one more. On this basis we would expect the growth in capacity to have a higher order curve than the linear growth of countries involved.

Estimation of probable carbon storage capacity to 2050

Existing and planned CCS capacity data supports faster than linear growth, as suggested by the trend in country involvement, discussed in the previous paragraphs. Although capacity has increased approximately linearly since 2010 (From Figure S15),

the shape of future capacity is not clear from existing project plans. Although the project database includes plans which account for up to 230 Mt CO_2pa by 2030, some of these may refer to different parts of the same project (the storage site and the capture site for instance) and, given historic success rates (page 56), many may not reach completion.

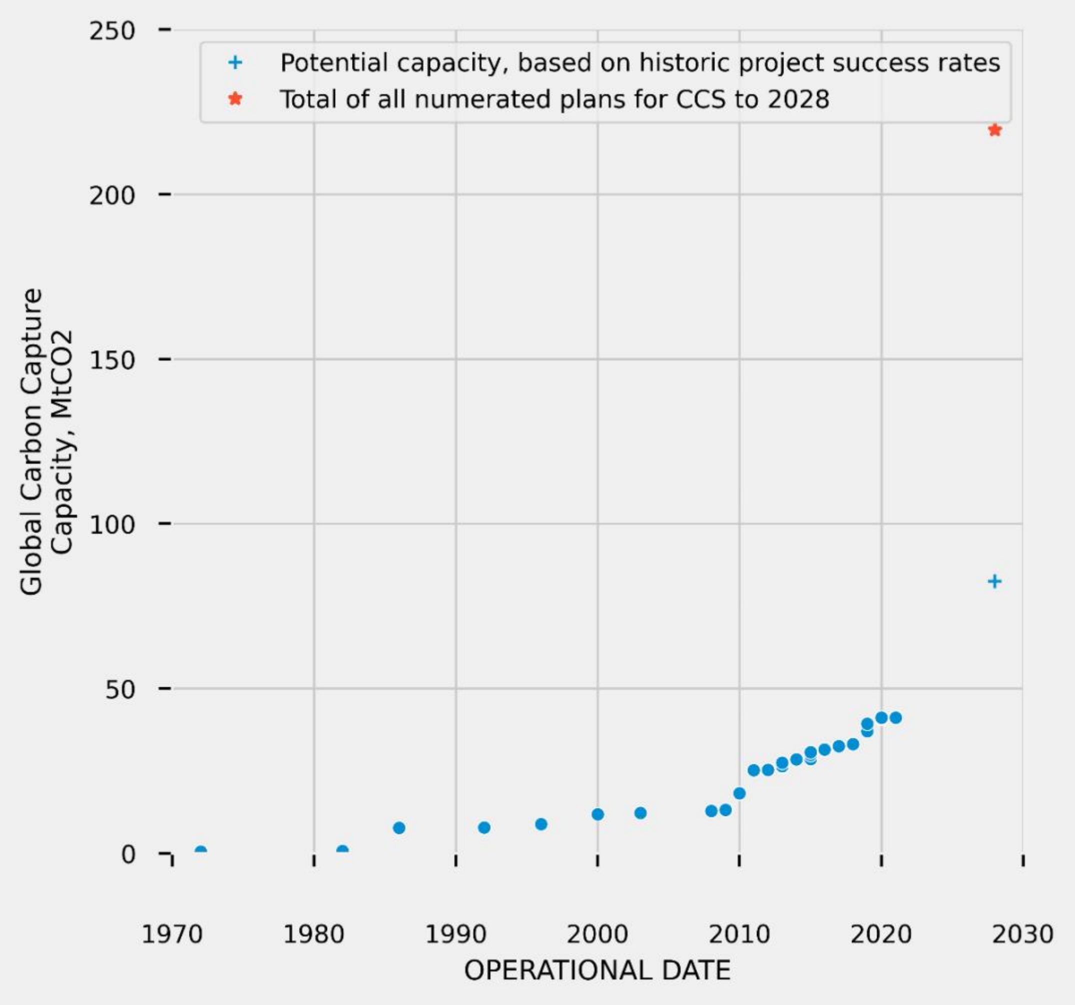

Figure S15 - Historical CCS capacity and future planned capacity to 2028. Data is from Status of CCS 2022 (GCSSI, 2022).

Given no evidence to support an alternative, it is possible that future growth could be exponential. Although based on outdated data, Haszeldine et al. (2018) derived an exponential growth curve from a more granular review of CCS project data than used here. Considering the historical project success rate (page 56) this curve appears to be broadly consistent with future plans. This is shown in Figure S16 assuming 100% realisation of projects "In Construction, 30% of those "In Development" and 20% of

those “In Early Development”. Although failure rates have been around 90% (page 56), it has been assumed that this improves over the coming decade due to past learning and more consistent funding, and that certainty of success will increase as the project nears completion.

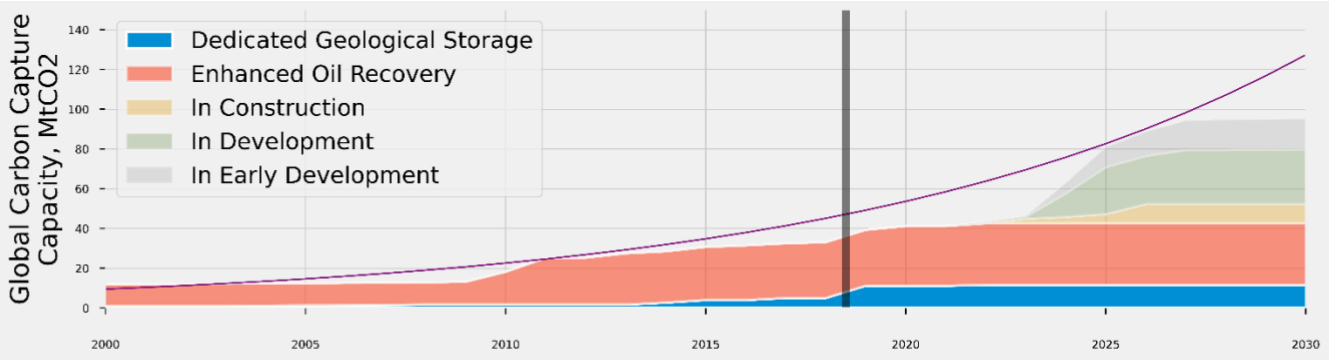

Figure S16 – A CCS exponential growth projection compared against existing plans for future deployment. Shaded areas show the current and planned capacity, assuming 100% realisation of projects “In Construction, 30% of those “In Development” and 20% of those “In Early Development”. The purple line shows the growth projection given by Haszeldine et al. (2018). Capacity data is from Status of CCS 2022 (GCSSI, 2022).

Summary: Carbon storage availability

The projected capacity for carbon storage supply is based on an exponential fit against historical projects, derived by Haszeldine et al. (2019), suggesting around 700 $MtCO_2$/yr capacity in 2050. Due to the immature state of this industry, there is high uncertainty in this growth curve, although it is consistent with industry sector reports of planned projects (Figure S16) and the potential rate of future financial investment in storage (page 59). The capacity factor for CCS in 2050 has been assumed to be 70% of total capacity, up from 60% today (page 56-59). The forecast supply growth curve can be viewed as optimistic: it is well ahead of the gradient of recent deployments (Figure S16), and does not explicitly consider storage or pipeline development, two potentially key constraints on growth [81,96].

3.1.4 Biomass

Biomass future supply requires different considerations to the other two ZERs because its growth is not primarily limited by infrastructure and technology deployment, but

by biological and ecological systems. Although there are many assessments of bioenergy technical and economic potential, these do not explicitly consider demands for food and materials (although these may be considered as an external constraint), produce a very wide range of expectations, and often do not critically consider the practicalities of rapidly scaling bioenergy supply. Refer to reference[97] for a comprehensive critical review of bioenergy assessments.

Biomass is accounted for in the model as the dry weight of plant biomass used to supply services to humans (Figure S17). In the literature, this use of biomass is referred to as Human Appropriation of Primary Production (HANPP) but what is included within this label varies (Figure S17). More specifically the quantity measured here is consistent with the term "used extraction", used by reference[98], and can also be referred to as the indicator domestic extraction (DE) in Material Flow Accounts [99]. In this analysis unused industrial residues are accounted within the total extracted biomass.

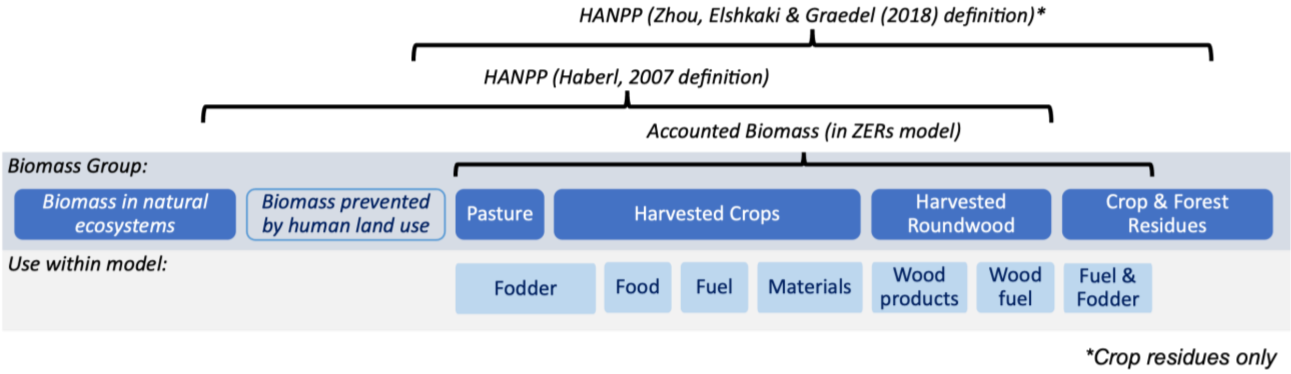

Figure S17 - Definitions of HANNP according to various studies, and as accounted in the model. Zhou et al. (2018) exclude bioenergy generated from non-commercial wood fuel, such as dedicated crops, crop residues, wood residues, municipal solid waste and livestock manure.

Current use of biomass

Although there are respected sources which provide global data for electricity demands and emissions estimates, there is not any single source which documents global production or consumption of all sources of biomass. Instead, this value is estimated from several sources, as outlined in Table S10.

There is high uncertainty in these estimations. Reference[100], for instance, found uncertainty levels in wood flows of -30% to +60%. It is particularly difficult to estimate

the quantity of human-appropriated crop residues and unreported forest biomass [101] but it is likely to be significant [102]. Total crop residues were approximately 7 Gt/yr in 2010, according to reference[103] but forest residues are more difficult to define, and the total human-used share of both is unknown at a global level. Uses include residential fuels [104], cropland fertilisation, animal feed, and industrial paper making [105]. In China for example, around 25% of straw residues are used for feed and paper production, and around 55% of forest residues are already used [105].

For this analysis, only residues used for fuel and for livestock feed are included, as they are the dominant use for residues globally. The mass of residue used for (undocumented) fuel is estimated as the difference between biomass consumed as primary solid biofuels and documented production of biomass as fuelwood. The detailed breakdown of the data, assumptions, and calculations used are given in Table S9. Residues used for livestock feed are based on the values estimated by reference[106], and reference[107], ensuring that the total biomass allocated for livestock feed in this analysis is consistent with the total feed calculated by reference[107]. Livestock feed is the focus of that analysis whereas reference[106] estimates mass flows for all uses of biomass. Although there are significant differences in the data provided by the two studies - reference[106] estimated a 50% higher total than reference[107] - reference[107] has been used as the main source.

Index	Parameter	Value	Unit	Justification
A	Total final consumption of Primary Solid Biofuels	36	EJ	From IEA energy balances
B	Production of commercial wood fuel	0.89	Gt biomass	FAO Forestry Production and Trade statistics
C	Production of commercial industrial roundwood	0.95	Gt biomass	FAO Forestry Production and Trade statistics
D	Production of biomass for non-food uses	0.78	Gt biomass	FAO Food Balances statistics
E	Energy density of primary solid biofuels	15	MJ/kg	Assuming a carbon content around 50%
F	Ratio between (Industrial residues used as energy) and (Commercial industrial roundwood)	31%		Estimated from the map of global wood carbon flows in 2010 compiled by Bais et al. (2015)
G	2010 share of non-food crops for energy in 2010	79%		From Bajželj et al. (2014)
H	Share of bioenergy use which is traditional	60%		For 2020, based on IRENA (2022)
I	Share of traditional fuels which are harvest residues	40%		Daioglou (2016) assumed 50% traditional biofuels are harvest, logging and process residues. Logging and process residues are assumed to make around 20% of that share.
J	Total ZER energy biomass consumption	2.1	Gt biomass	(A / E) - (C x F) Note that this excludes process residues from industrial processes to avoid double counting. This estimate also excludes biomass used for transformation energy (around 10 EJ in 2018 according to IEA Energy Balances).
K	Biomass for energy, excluding commercial wood	1.2	Gt biomass	J - B
L	Of which: biofuel crops	0.62	Gt biomass	D x G
M	Of which: harvest residues	0.58	Gt biomass	A x H x I / E

*Table S9 - **Estimated global use of residues for energy in 2018.** The energy efficiency of using biomass as fuel is not relevant for this calculation because the IEA datapoint gives the energy of fuel consumed, not the final energy required.*

Biomass Type	Wet weight, Gt (Note f)	Estimated dry weight, Gt	Grouped dry weight, Gt	Notes
Wood fuel		0.9		a
Industrial roundwood		0.9		a
Commercial wood			1.8	
Rice	0.7	0.3		b
Other plant-based food	6.8	2.8		b
Plant based fodder (excluding residues and pasture)		1.7		c
Biomass for non-food uses	0.8	0.8		b
Farmed biomass			5.6	
Pasture and livestock forage			3.0	c
Harvest residues for fodder		1.3		d
Harvest residues, unreported wood collection, and animal matter for fuel		0.6		e
Other unreported biomass (residues and forage)			1.9	
TOTAL			12.4	

Table S10 - Estimated consumption of biomass in 2018. Notes & sources:

a: FAO Forest Production & Trade Data [108]
b: FAO Food Balances [109], converted to dry weights using ratios estimated by reference[110].
c: [107]
d: Estimated based on the sum of crop residues and forage, as accounted by reference[106], and to bring the total feed allocation to be consistent with the estimate of [107].
e: Estimated as the difference between total final consumption of Primary Solid Biofuels, reported in IEA World Energy Balances [111], and production of energy crops, wood fuel and processing residues used for energy, estimated from FAO data, reference[106], and reference[100]
f: Only where data is provided in this format

Estimation of a sustainable trajectory of biomass use

To derive a trajectory of sustainable future biomass supply, the estimated maximum sustainable consumption is assumed to be available in 2050. The trajectory assumes linear growth between historical consumption in 1960 and 2010 (estimated from the results of reference[103]), current consumption (as outlined above), and 2050 consumption (detailed in the remainder of this section).

Reference[103] only account for roundwood fuelwood, which is documented by the FAO excluding bioenergy generated from crop residues and wood residues. For consistency with the biomass groupings used in this study, the biomass used as fuel and to feed livestock in 1960 and 2010 has been modified from the values given by reference[103] to also include the residues used for these purposes. Biomass used for fuel and livestock feed have been scaled by 1.67 and 1.27, respectively, for consistency with the relative masses of harvest residues used for fuel and commercial fuel, and masses of fodder from residues compared to crop fodder and pasture, based on the flows in 2018 as calculated in Table S10.

Human use of biomass has grown steadily over the past 40 years with growth in population and levels of affluence [103]. Livestock feed and food crops increases have dominated the historical growth of biomass extraction [103] but have been attributed largely to a doubling of irrigated areas, and increasing use of scientifically bred seeds and commercial fertilizer [112]. Since cropland and pasture expansion cause land-use change emissions, this form of growth is incompatible with the ZER model. The ZER calculator assumes that all avoidable deforestation and land-use degradation stops such that any unavoidable damage (such as from forest fires) is compensated by the net-sink from existing managed forest, as explained in Part 1. Only managed land is considered, consistent with bookkeeping approaches as used in IAMs used to derive the net-zero target (Shukla et al., 2022).

The only possible expansion of biomass consumption therefore is by increasing land-use intensity without net change in carbon stocks, and minimising impacts on biodiversity. Following the simplified carbon cycle used for this model (Figure S3, p18) it is possible to increase land-use intensity with no net change in carbon stocks since increasing the volume of biomass grown increases both sequestration and resulting

emissions. However, this does not account for the time lag between growth and subsequent emissions, which is significant for tree growth. There may be many climate impacts, such as changes in belowground biomass and soil carbon content. Reference[114] argue that the impact of land management changes on surface temperature can be of the same magnitude as land-cover changes but, since these effects are complex and unquantified, they are outside of the scope of this model.

These provisions mean that there may be small increases in wood availability and crop productivity by closing yield gaps and use of residues. For wood availability, although there can be no expansion of intensive plantations, additional recently planted stands will come to maturity in the next decades. Crop production intensity has some potential to increase by reducing the global yield gaps [115] but crop yields in regions of current high productivity are unlikely to increase over the coming decades. Reference[116] used statistical models to analyse historical yield trends of rice, wheat and maize (85% of global cereal production) in major crop-producing countries since 1965, finding that while historical crop yields have increased linearly, there is evidence of stagnation major crop-producing countries, possibly due to biophysical limits. Compounding this, the IPCC concluded with high confidence that climate change will increasingly decrease overall crop productivity in their special report on land and climate change [28]. A higher proportion of crop and forest residues could be used but removal of these resources is limited by their role in other ecological functions, such as maintaining soil nutrient quality [117]. The estimated values for each of these biomass components, and their justifications are given in Table S11 (page 70).

Although novel sources of biomass such as algae may have Gt scale potential [45], a significant growth increase is not considered since their future role is “highly uncertain”[118]. Current use of ‘aquatic’ plants for food and feed is accounted for in the FAO data used to estimate quantity of food biomass production [109]. The IPCC special report on renewable energy sources (Chum et al., 2011) highlighted two potential barriers to near-term future reliance on marine biomass; sources may have detrimental impacts on marine ecosystems and the potential rate and scale of near-term deployment is unknown.

Summary: Biomass supply

Sustainable biomass supply is limited by land availability, and biological and ecological systems. Current and future supply is calculated from a range of data sources since there is no global measurement of used- or sustainable- extraction, as shown in Table S11. In the absence of an approach to quantify sustainable extraction within the scope of this work, it is assumed that today's level of extraction is within environmental limits. Reference[119] argue, however, that current rates of human appropriation of biomass (HANPP) are "well beyond a precautionary planetary boundary". The defined trajectory should therefore be considered an upper-bound of availability.

It is assumed that future extraction could be marginally increased, only as recently planted stands come to maturity, by more effective residue-use and by closing yield gaps. Cropland and pasture expansion cause land-use change emissions so are excluded. Any expansion of biomass appropriation however entails risks and may impact other environmental boundaries, such as biodiversity [120].

Biomass consumption in 2018 and the maximum sustainable rate of biomass extraction in 2050 were estimated to be 12.4 Gt/yr and 15.8 Gt/yr, respectively, as shown in Table S11.

Biomass Type	2018 dry mass, Gt		2050 dry mass, Gt		Justification
Wood fuel	0.90		1.00		Assume only small increases in intensity are possible on existing intensively managed plantations without affecting carbon stocks and mineral availability. Jonard et al. (2014) found downward trends in minerals of European forests, arguing nutrient availability needs to be considered in future productivity assessments.
Industrial roundwood	0.90		1.00		
Commercial wood		1.8		2.0	Wood fuel + Industrial roundwood
Rice	0.3		0.4		Crop yields in regions of current high productivity are assumed to remain constant in the coming decades, based on the statistical analysis of cereal crop yields by Grassini et al. (2013), and evidence that climate change and extreme weather will negatively impact crop productivity (IPCC, 2019; Lesk et al., 2016). Grassini et al. (2013) used statistical analysis to show that while historical crop yields have increased linearly, there is evidence of stagnation major crop-producing countries, possibly due to biophysical limits. Better distribution of agricultural inputs and seed varieties may increase yields of underperforming agricultural land. This yield gap is assumed to close to 75% of the full potential, estimated by Foley et al. (2011) to increase production by 28%.
Other plant-based food	2.8		3.6		
Fodder	1.7		2.2		
Biomass for non-food uses	0.8		1.0		
Farmed biomass		5.6		8.3	Sum of farmed plant biomass products
Pasture		3.0		3.0	Assume that it is not possible to increase the intensity of pasture use without impacting other environmental considerations
Forest residue use	1.9		0.4		Assume a forestry residue generation ratio of 60%, as used by Koopmans et al. (1997), Smeets & Faaij (2007) and broadly consistent with the wide range of values, depending on species and management type, used by Daioglou et al. (2015). Of the 40% residue, it is assumed that around 35% could be realistically harvested (based on the review of Smeets & Faaij (2007) who found estimates ranging from 25-50%). This is assumed to be sustainable on the basis that only around 30% of the conventionally unrecovered residue must be left in the forest to maintain ecological functions (based on the relative shares of ecological and theoretical potentials for residue use calculated by Daioglou et al., 2015). Smeets & Faaij (2007) estimate the 2050 non-food biomass energy potential to be 0.3 Gt.
Crop residue use			3.2		Assume that the residue:crop ratio is approximately 0.8, consistent with 2009 global agricultural flows estimated by Bajželj et al. (2014), of which 45% must be left in the field to maintain ecological functions (based on the relative shares of ecological and theoretical potentials for residue use calculated by Daioglou et al., 2015). Note that this implies 65% of residues are recovered, significantly more than the rate today. This value falls within the higher end of the range of technical potentials for bioenergy from agricultural residues, collated by Haberl et al. (2016).
Foraged biomass (from residues)		1.9		3.6	Sum of unreported biomass. Note that the 2018 value includes informal fuelwood collection (of around 0.5 Gt).
TOTAL		12.4		15.8	

Table S11 - Probable maximum sustainable biomass in 2050. 2018 estimates are taken from Table S10.

3.1.5 Estimated probable future supply of ZERs

The maximum probable supply trajectories for the three ZERs are shown in Figure S18. These are forecast from historical deployment and current trends, provided there is strong evidence that these trends can continue, as described in Sections 3.1.2 (p47), 3.1.3 (p53) and 3.1.4 (p62) for Non-Emitting Electricity generation (NEE), biomass and carbon storage, respectively.

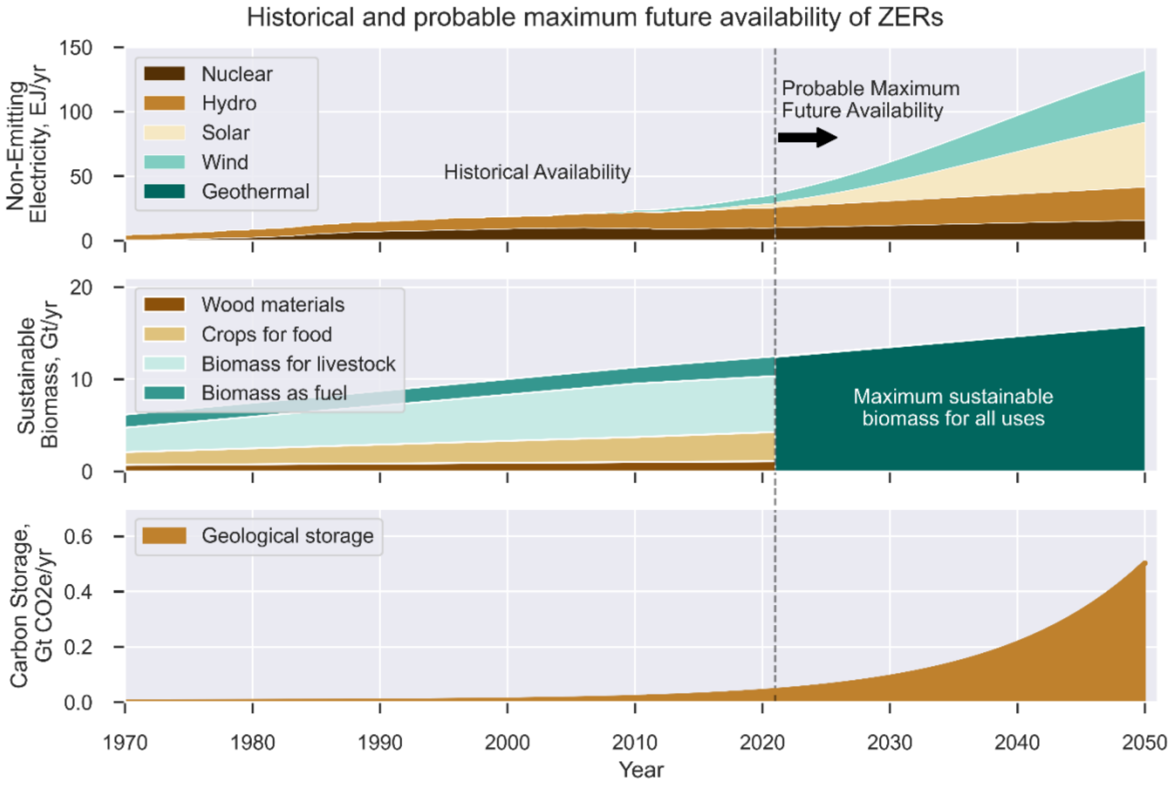

Figure S18 – Historical and probable future trajectories for ZERs, as detailed over p45 - p71. Key sources: [79,90,106,108,121,122].

Confidence in the chosen trajectories of maximum probable supply

The preceding sections have quantified maximum probable supply trajectories for the three ZERs – these aim to represent the maximum supply which we can rely on, given the risk framework, described in Section 1.1.1. The text below considers the confidence in these estimated values. To acknowledge the uncertainty in these projections, however, a broader range of possible supply trajectories is also quantified in Section 4.2

While being based on physical and political feasibility, the chosen trajectories for NEE and carbon storage still demand challenging levels of growth and favourable conditions, and any expansion of biomass appropriation may impact other environmental boundaries, such as biodiversity [120]. Indeed, reference[119] argue, that current impacts on biomass are "well beyond a precautionary planetary boundary". The biomass trajectory here must therefore be considered an absolute upper bound. The realism of the NEE and carbon storage projections is reviewed in the final part of this section.

Although based on historical trends, there may be logistical, technical, economic and environmental limits to the rate and extent of growth of NEE. While nuclear power growth relies on continued public support and an end to multi-year delays and cost escalations, such as those reported at Britain's Hinkley Point C plant [123], the trajectory in this analysis is a reasonable best guess of deployment, being based on bottom-up assessments at a country level, derived by the authority in global nuclear power [73]. The trajectories for renewable generation, however, are more uncertain.

The extent of growth in solar and wind is well below the target agreed by 130 countries at COP28 to triple capacity by 2030 (as reported by the reference[124]) and only a fraction of the global technical potential [125], but increased intermittent generation would increase curtailment. Analysis by the reference[126] suggests that curtailment – the deliberate reduction in generation to balance the grid - has a greater dependency on grid investment than market penetration but such upgrades will be subject to constraints on global equipment and resource availabilities (such as vessels for installation of offshore wind turbines). The IEA found curtailment to be between 1% and 10% for countries with maximum variable renewable energy shares today (around 40%) reference[126].

The projection for hydropower is uncertain in three areas: material demand, socio-environmental constraints, and the geographical distribution of resources. Firstly, the construction of an additional 8 EJ of hydropower generation capacity by 2050 would demand large quantities of construction materials and energy which are not explicitly quantified in the model end-user demands, set by industry expectations. Secondly, it may be challenging to maintain the linear trend of growth in hydropower generation beyond as the remaining potential sites become increasingly less cost-effective.

According to analysis by Xu et al. (2023), although global theoretical hydropower potential is around 220 EJ/yr, the feasible and profitable potentials are only 40 EJ/yr and 19 EJ/yr respectively when broader environmental and social impacts are considered. Lastly the geographical distribution of resources may also challenge this growth since 2/3 of the profitable potential found by Xu et al. (2023) is in the Himalayas.

The assumed probable availability of CCS is perhaps the most uncertain and optimistic of the three ZERs projections. Although the estimate of 500 MtCO_2/yr carbon stored by 2050 is based on real data and considerations of project limitations, the exponential capacity growth (to 700 MtCO_2/yr) is based on assumptions of multiple countries adopting significant funding incentives [87]. It would also demand significant improvements in technological and project learning, of which there is little evidence currently. Comparisons with the growth seen in the solar industry, such as used by reference[78], are unlikely to be valid because the nature of the industries are very different. Unlike solar, CCS has extremely high capital intensity, high uncertainties around future revenue streams, regulation and technical performance, and a complex value-chain. Considering the more analogous growth trajectories of the US nuclear-power industry, the US SO2- scrubber industry, and the global liquefied natural gas (LNG) industry, reference[94] conclude that there is no guarantee of reducing costs with increased technology experience. Furthermore, as Lane, Greig and Garnett (2021) argue, carbon storage specifically is unlikely to follow conventional learning-curves because the most favourable sites will be accessed first.

Countries such as China may have capability to develop large infrastructure at a rate and scale inconceivable elsewhere [79], but, as the pro-CCS GCCSI's progress report in China (2023) records: China's CCUS growth "is facing many challenges", including "lack of effective business models, insufficient incentives & regulatory measures, and difficulties in sources-sinks matching". Given the challenge of storage specifically a large proportion of the expected capacity growth could be used as CCU, rather than storage, and therefore irrelevant for this analysis.

In conclusion, although uncertainties always exist in projections of future resource availability, the maximum probable ZERs trajectories in Figure S18, are credible "best-

estimates" of the supply which we can rely on, following the risk framework, described in Section 1.1.1. The range of uncertainty is considered further in Section 4.2.2.

4. Verification approach

The ZERs model is designed to evaluate whether there will be sufficient ZERs to meet the demands set by net-zero proposals. To draw a robust conclusion, the combination of the uncertainty in the model value for each ZER and in the prediction of its availability should be less than the difference between model value and the prediction. However, the model output uncertainty is difficult to assess because the input uncertainty is largely unknown and there are systematic error sources.

Assuming that the model will be used only as a tool to "sense check" net-zero proposals (versus a detailed policy planning tool), sources of error and uncertainty are not fully quantified and characterized. Rather than purely *positivist validation* (using statistical tests to demonstrate that the model is an accurate representation of reality), this approach uses *relativist validation*, as suggested for exploratory modelling by reference[128]. *Relativist validation* is a more qualitative assessment of whether the model is useful and fit for its intended purpose.

Two approaches are used. Firstly, bottom-up model estimates of 2018 demands are compared against top-down data to indicate the plausibility of the model. Secondly, the uncertainty in supply trajectories and model parameters are considered to determine whether the overall conclusions are affected.

4.1 System validation against 2018 data

The following sections verify the model performs 'acceptably' against a baseline case, using 2018 historical data. 'Acceptability' is judged by considering whether the error would change the conclusions which would be drawn from the model, and whether the size of error is consistent with the uncertainty in the data. The model is not expected to be perfect, but it is expected to be 'good enough' for the purposes of the analysis.

2018 values of non-emitting electricity generated, biomass consumed, and emissions produced are estimated for the top-down comparison, based on data from a range of sources. The bottom-up model output values are compared against these values in the following sections.

4.1.1 Global use of biomass, and generation of non-emitting electricity & emissions

The model (outlined in Section 4) is run using the inputs (activity rates and delivery process shares) listed in Part 6, which are derived from references:[82,109,111,129–141]

Table S12 compares the model estimates for ZER demand in 2018 against estimates from other sources. The model output is within 15% of global estimated flows for all three ZERs. Both Non-Emitting Electricity demand and generated emissions are an underestimate of the actual value, which is encouraging because it indicates that the model is not overestimating the demand of these resources, and is consistent with the nature of a bottom-up model (where some demands will have been overlooked).

Conversely the model appears to have overestimated the demand for biomass but the 'actual biomass' value may be an underestimate. It is a bottom-up estimate, including bottom-up data, compiled by FAO (the official data source for wood production). FAO potentially undercounts actual wood harvests due to illegal logging, which could account for 5% - 10% of additional production based on independent analyses by reference[100] and reference[142]. Reference[143] estimated crop residue consumption in 2005 as approximately 50% greater than the value used in this analysis (1.6 PgC/yr, or approximately 3 Gt dry biomass), derived by summing regional crop specific recovery rates. Reference[143] might be considered an upper bound estimate - they consider all residue uses (rather than only two in this analysis) but may overestimate the quantity

of residues that are *used* by accounting top-down *collection* rates, rather than *use* rates. The error in total biomass demand is less than 10%, and acceptable given the uncertainty in the data.

Resource	Model Prediction	2018 Actual	Percentage Error
Biomass Demand, Gt dry Biomass	13	12	7.0 %
Non-Emitting Electricity Demand, EJ	30	32	-6.5 %
Total Residual Emissions, Gt CO_2e	45	51	-12 %

Table S12 - Model predicted values for the three ZERs for 2018, against actual demand. Actual values are based on data provided in Section 3.1. Percentage error is calculated as (model predicted value - actual value)/actual value.

4.1.2 Rates of Intermediary Activities

While end-user activity rates in the model are set by the user, the rates of intermediary activities required to achieve these are estimated by the model. These can therefore be used to understand whether the model is performing as expected – whether the model is a reasonable representation of the provision of global goods and activities in 2018.

The rates of intermediate activities are compared against actual 2018 data in Table S13. The rates of each intermediate activity were estimated from references: [109,111,129,136,144–150]

Activity	Rate from literature	Model rate	Units	Percentage error (%)
Coal	7.6	4.8	Gt run-of-mine coal extracted	-37
BioFuel	0.13	0.089	Gt biodiesel and bioethanol	-32
Hydrogen	0.045	0.032	Gt hydrogen	-29
Oil	4.6	3.4	Gt oil products	-26
ExtractedOilGas	8.0	6.2	Gt oil and gas extracted	-23
HVCs	0.42	0.51	Gt High Value Chemicals	22
Methane	3.1	2.5	Gt methane	-17
Urea	0.29	0.24	Gt Nitrogen Fertilisers	-16
NonWoodBiomass	9.3	11	Gt biomass produced from agriculture, dry weight	14
Wood	3.1	2.7	Gt woody biomass	-13
CO2Product	0.23	0.20	Carbon dioxide gas	-13
GeneratedElectricity	96	90	EJ electricity produced	-7
Electricity	83	79	EJ consumed	-5
Ammonia	0.17	0.18	Gt ammonia	3
Methanol	0.063	0.061	Gt methanol	-3
RawFood	8.2	8.2	10^15 kcal food at farm gate	0
Synfuel	0	0	Gt synthetic fuel	

Table S13 - Comparison of model predicted rates of activities compared to actual rates for 2018. Percentage error is calculated as (model predicted value - actual value)/actual value. GeneratedElectricity is the net electricity produced before transmission and Electricity is the net electricity, including transmission losses. Additional activity details are given in Part 5.

As Table S13 shows, rates of intermediate activities generally follow the expected trend of data from other sources, but the accuracy is relatively low. Since the maximum percentage error is as large as 37% (Table S13), the model is clearly not suitable for understanding the rates of individual processes. On the other hand, the mean absolute percentage error, 15%, shows that the model is broadly performing as expected, and has an error on a similar scale to the uncertainty levels in some datasets. Reference[151], for instance, use an acceptability threshold of 25% in their uncertainty analysis of UK energy flows based on a review of thresholds used by statistical offices. By considering the direction of error we find the mean percentage error (-11%) is smaller than the absolute percentage error, and negative. This means that the spread of error at the process level acts to reduce the overall error, and that model is generally underestimating intermediate process rates to give a conservative estimate of the overall resource demands.

The largest discrepancies are for energy carriers - this is expected given the model is consistently under predicting intermediate activity rates, and so energy carriers mostly accumulate the error.

4.1.3 Activity Energy Demands

Since the model estimates the total flows of resources required for each activity, it can also be used to estimate their energy demands. Comparing these estimated demands against documented data will reveal whether the energy efficiencies of processes in the model are reflective of real-world processes in 2018.

The model-estimated energy demands are compared against data provided by the International Energy Agency (2021). To compare the two sources, model values for activity for each fuel are scaled by the assumed energy density of the fuel and summed. Since the model and data do not use the same categories, activities have been grouped where necessary. The energy density values and groupings used are given in Table S14 and Table S15, respectively. The use of biomass has been generally assumed to be as a material, except for activities within the categories of *Building Activities* and *Electricity Generation*; an energy density of 15 MJ/kg biomass has been assumed for these activities. *Paper* production, *OtherIndustry* and *Construction* have also been corrected to account for the share of biomass used as fuel - the proportion of biomass used as energy is taken from the value used to derive the model coefficients.

Resource	Energy_Density (MJ/kg)
BioFuel	35
Coal	28
Electricity	1
Methane	45
Oil	42
Synfuel	42

Table S14 - The energy density values used to estimate the model calculated energy consumption per activity. Only fuels shown have been assigned a non-zero energy density; other substances have been assumed to not have significant levels of use as a fuel in 2018. Electricity is quantified in energy units in both sources and so no 'conversion factor' is needed.

IEAComparableGrouping	IEA_Label	Model Activity Label
AgricultureForestry	Agriculture/forestry, Fishing	NonWoodBiomass, RawFood, Wood
Aluminium	Non-ferrous metals	Aluminium
Aviation	World aviation bunkers, Domestic aviation	Aviation
BuildingActivities	Residential, Commercial and public services	Appliances, Cooking, Cooling, Lighting, SpaceHeat, WaterHeating
Construction	Construction	Construction
ElectricitySupplyDistrib	Own use in electricity, CHP and heat plants, Losses	Electricity
Food	Food and tobacco	Food
GeneratedElectricity	Main activity producer electricity plants, Autoproducer electricity plants, Main activity producer CHP plants, Autoproducer CHP plants, Main activity producer heat plants, Autoproducer heat plants, Heat pumps, Electric boilers, Chemical heat for electricity production	GeneratedElectricity
MinedMetalsMinerals	Mining and quarrying	MinedMetalsMinerals
NonMetallicMinerals	Non-metallic minerals	Cement, Glass
OtherIndustry	Transport equipment, Machinery, Wood and wood products, Industry not elsewhere specified, Transport not elsewhere specified, Final consumption not elsewhere specified	OtherIndustry
Paper	Paper, pulp and printing	Paper
Petrochem	Chemical and petrochemical, Pipeline transport, Petrochemical plants, Memo: Non-energy use in chemical/petrochemical	Ammonia, CO2Product, CO2Storage, HVCs, Hydrogen, Methane, Methanol, OtherPetChem, Plastics, Synfuel, Urea
RailTransport	Rail	RailFreight, RailP
RoadTransport	Road	BusUse, CarUse, RoadFreight
Shipping	World marine bunkers, Domestic navigation	Shipping
Steel	Blast furnaces, Coke ovens, Iron and steel, Blast furnaces, Coke ovens	Steel
Textiles	Textile and leather	Textiles

Table S15 - The groupings used to compare IEA consumption data against model predicted energy consumption. Note that Activity Labels are the short description of an activity – production of materials, therefore, are generally labelled by the material itself. For example, the Activity Label 'Steel' represents 'production of steel'.

The comparison between model and IEA values are shown graphically in Figure S19. The model shows a general trend of total energy use for activities similar to the IEA data, but some activities have significant differences from the data. These can mostly be explained, for example:

- Some activities are not currently included in the model - this reduces the demands for intermediate substances, reducing upstream energy demands, for example for electricity supply.
- There is currently no delivery process for heating buildings and water using district heating. For the verification run, this share has been allocated instead to heatpumps which may be more efficient.
- The coefficients for the *Petrochemical* and *Steel* industry are modelled using Best Available Technology (BAT) values for energy use, and so use less than the average today.
- The IEA category of *NonMetallicMinerals* includes all non-metallic mineral production but only cement and glass are included in the model currently.
- *Aviation* only accounts for passenger transportation in the model. Transportation of cargo accounts for around 20% of aviation emissions, according to Graver et al. (2019).
- The model over-predicts energy use in agriculture and forestry - this could be because there is overestimated use of *NonWoodBiomass* but underestimated use of *Wood*, while *NonWoodBiomass* requires more energy for production. There are also high uncertainties in the data for agriculture and forestry, and agriculture energy values are based on a study based on the UK (rather than global data).

Overall, the total error in estimation of energy use is -100EJ, 17% of the total of the activities considered here. Although this is not small, it can be partially explained, is consistent with the levels of uncertainty in the data, and is conservative towards the aims of the model.

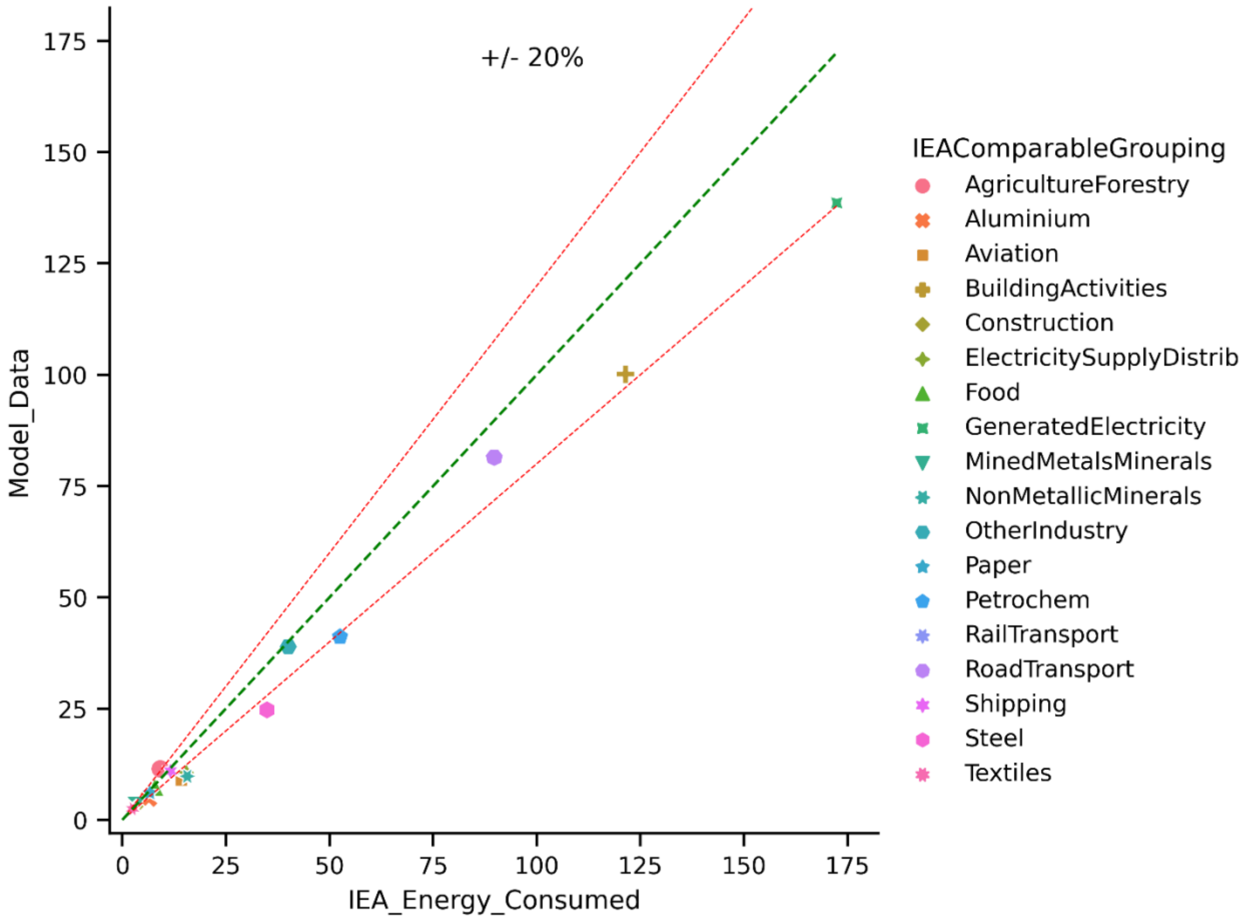

Figure S19 - Comparison of model predicted energy consumption of activities compared to actual energy consumption for 2018; all values in EJ. Actual 2018 consumption data are estimated from IEA Extended World Energy Balances, considering Energy industry own use, Consumption and Transformation Processes. IEA groupings and Model Activities have been grouped for comparison.

4.1.4 Sectoral breakdown of global GHG emissions

The direct contribution of each service to the total produced emissions can be extracted from the model by considering the flow of the model substance *NetEmissions*. This is compared against another data source to understand whether the sectoral distribution of emissions is representative for 2018.

The model output is compared against the sectoral distribution of emissions compiled by reference[152]. To provide comparable groupings the activities have been grouped as shown in Table S16. Although both the datasets have a group for *cement*, the model category includes both process and fuel emissions within this category but the dataset from Minx et al. (2021)[152] assign only process emissions to this category with

combustion emissions assigned to *Other (industry)*; *cement* is therefore included within the grouped service *OtherIndustry*.

As shown in Table S17, the model shows a general trend of emissions for activities similar to the data generated by reference[152], although some activities have significant differences from the data. These are discussed below.

Activity group	Minx et al. (2021) label	Model label
AFLUC	Biomass burning (CH4, N2O), Enteric Fermentation (CH4), Managed soils and pasture (CO2, N2O), Manure management (N2O, CH4), Rice cultivation (CH4), Synthetic fertilizer application (N2O)	NonWoodBiomass, RawFood, Wood
Aviation	Domestic Aviation, International Aviation	Aviation
BuildingActivitys	Non-CO2 (all buildings), Non-residential, Residential	Appliances, Cooking, Cooling, Lighting, SpaceHeat, WaterHeating
Cement	Cement	Cement
Chemical	Chemicals	Ammonia, BioFuel, Glass, HVCs, Hydrogen, Methane, Methanol, OtherPetChem, Synfuel, Urea
CoalMining	Coal mining fugitive emissions	Coal, MinedCoal
GeneratedElectricity	Electricity & heat	GeneratedElectricity, Electricity
MetalIndustry	Metals	Aluminium, Steel
OilAndGas	Oil and gas fugitive emissions, Other (energy systems), Petroleum refining	ExtractedOilGas, Oil
OtherIndustry	Other (industry)	Construction, Food, MinedMetalsMinerals, OtherIndustry, Paper, Plastics, Textiles, NotOtherwiseSpec
RailTransport	Rail	RailFreight, RailP
RoadTransport	Other (transport), Road	BusUse, CarUse, RoadFreight
Shipping	Inland Shipping, International Shipping	Shipping
WasteManagement	Waste	WasteSolid, Wastewater

Table S16 - The groupings used to compare emissions data from Minx et al. (2021) against model predicted emissions for 2018.

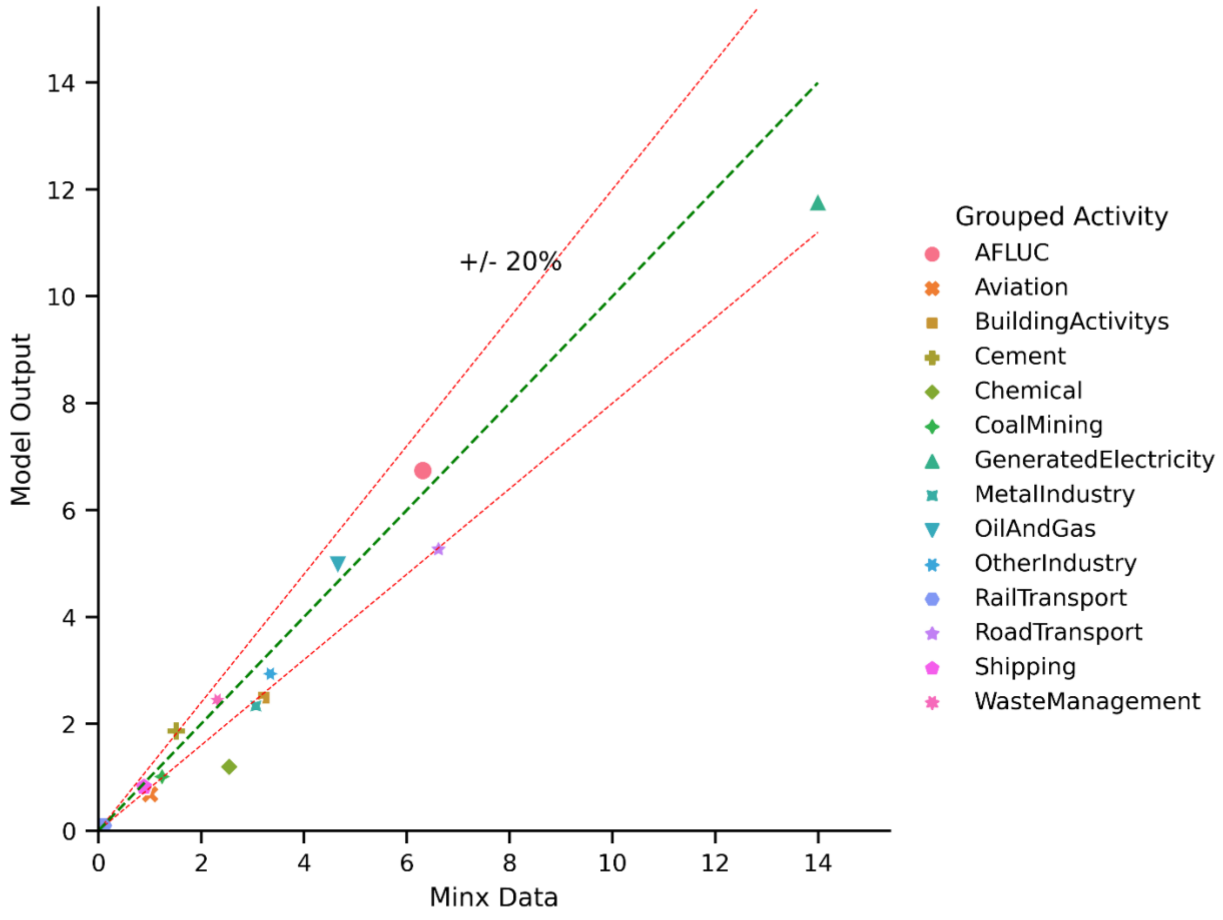

Figure S20 - Comparison of model predicted emissions rates against actual emissions for 2018, measured in $GtCO_2e$. The activity groupings are shown in Table S16.

Grouped Activity	Minx Data	Model Output	Error	Percentage Error
GeneratedElectricity	14	12	-2.2	-16
RoadTransport	6.6	5.3	-1.4	-20
Chemical	2.5	1.2	-1.3	-53
MetalIndustry	3.1	2.3	-0.7	-24
BuildingActivitys	3.2	2.5	-0.7	-23
AFLUC	6.3	6.7	0.4	7
OtherIndustry	3.3	2.9	-0.4	-12
Cement	1.5	1.9	0.4	24
Aviation	1.0	0.7	-0.3	-32
OilAndGas	4.7	5.0	0.3	7
CoalMining	1.2	1.0	-0.2	-18
WasteManagement	2.3	2.4	0.1	6
Shipping	0.9	0.8	-0.07	-8
RailTransport	0.1	0.1	0	-5

Table S17 - Comparison of estimated emissions (in $GtCO_2e/yr$) from Minx et al. (2021) against model predicted emissions for 2018. The activity groupings are shown in Table S16. Percentage error is calculated as (model predicted value - actual value)/actual value.

The differences between the estimated emissions can be explained similarly to those in comparing energy demands but there appears to be an additional significant discrepancy for the group, Chemicals. The current difference accounts for -1.3 Gt CO_2e/yr. This is mostly because significant emissions sources are not currently included, listed in Table S18. Including these emissions sources would reduce the error in this category to -15 %. This is consistent with the lower estimated energy use, since the coefficients are based on Best Available Technology. As such, although there is an issue at the service level, the discrepancy in the model can be explained and is acceptably small at a whole system level; the discrepancy accounts for -3 % of total global emissions. If a higher level of accuracy in the model is needed at a later stage, these processes can be added. The total error in estimation of emissions is -6.2 Gt CO_2e/yr, 12% of the total emissions according to reference[152] data, and conservative to the model aims.

Unaccounted emissions	Quantity (Gt CO2e)	Source for emissions estimation
Process emissions in HVC, plastics, and other chemicals production	0.14	Levi & Cullen (2018)
Lime production	0.32	Edgar v7
Indirect N2O emissions from the atmospheric deposition of nitrogen in NOx and NH3	0.18	Edgar v7
Non-Energy Products from Fuels and Solvent Use	0.20	Edgar v7
Other Process Uses of Carbonates	0.12	Edgar v7
**TOTAL**	**0.96**	

Table S18 - Unaccounted sources of emissions in the Chemical sector (per year). Data sources: [150,153]

4.2 Assessing uncertainty in the analysis

Future economic, political, social, and climate systems are inherently uncertain. Any analysis dependent on these systems (such as the supply of ZERs) is thus subject to uncertainty. Similarly, the model coefficients describe resource flows through processes, which may not currently exist in commercial settings, which may represent the average of many more specialized processes and/or, which may have significant regional variability.

To understand the potential impact of these uncertainties on the analysis conclusions, this section considers the potential range of these uncertainties, and how they might interact. Only uncertainty analysis of the model itself is considered, without sensitivity analysis (using the definitions used by reference[154]). Our interest is not in the detail of the model performance, nor an optimised solution, but in whether our broad conclusions are correct. The impact on the analysis conclusions is discussed in Section 6.

The ZERs model is designed as a Robust Decision Making (RDM) tool to stress-test proposals and develop more robust policies. As described by reference[155], in RDM the focus is not on improving accuracy to make better predictions, but to "yield better decisions under conditions of deep uncertainty". The model provides additional data points to inform those better decisions. A detailed uncertainty characterisation, as described by reference[156] would still be a valuable extension but is beyond the scope of the current analysis. This work is not designed as a precise prediction tool, but aims to provide initial data points for ZER demands where none currently exist, using an alternative approach to dominant economic modelling approaches. Although the output accuracy may not be known, the model outputs suggest that the certainty inherently assumed by users of economic models may be misplaced.

Sources of epistemic uncertainty in the model may include:

- Parameter uncertainty due to:
  - Uncertainty in the input data and assumptions;
  - Misinterpretation of data sources due to imprecise or uncomprehensive documentation;

  - Errors in data collection or processing;
  - Key emissions or resource demands being overlooked; and
  - Ambiguity in allocation and attribution of emissions or energy where there is not a clear boundary between activities, such as the case for agriculture and forestry.
- Structural uncertainty, including:
  - Representation of all activities as linear processes. Where demands do not scale linearly with the activity rate, any significant deviation from the baseline value will introduce inaccuracies. Aviation is a good example; the fuel demands do not have a linear relationship with distance travelled because planes use more fuel in the take-off phase than any other segment of the flight. However, if the distribution of flight lengths does not change significantly from the those assumed in deriving coefficients, the overall error will be small.
  - Grouping errors due to the model granularity. The limited number of delivery processes currently implemented in the model means that some processes are not represented. For example, according to reference[157] around 9% of ammonia is produced using oil as a feedstock but, with no ammonia-from-oil delivery process, it must be allocated to another. Additional processes can be added, as necessary, following the steps in Section 2.3.

4.2.1 Uncertainty in supply trajectories

The *'maximum probable'* supply trajectories in the main paper quantify the maximum supply which we can rely on, following the risk framework, described in Section 1.1.1. Acknowledging that there is significant uncertainty in these trajectories, a range of possible future supply is shown in Figures 4 and 5 of the main paper, bound by a (more pessimistic) *'lower risk'* estimate and a (more optimistic) *'maximum possible'* estimate of 2050 ZER supply. The basis for these estimates is summarised in Table S19.

The *lower risk* estimates for NEE generation and Carbon Storage assume linear rates of deployment, consistent with retrospective studies of large energy infrastructure projects, which show linear growth once it achieves 'materiality' of around 1% global energy[158]. The linear trajectories are based on 2010-2020 supply (using the data described in Section 3.1).

The *lower risk* estimate for Biomass is below the *maximum possible* value to account for the uncertainty in the expectation of biomass availability (discussed in Section 3.1.4. This is quantified as ±15%, consistent with the discrepancy between the ZER model calculated value for 2018 biomass consumption and the top-down estimate, as found in Section 4.1. The *maximum possible* estimate, similarly, accounts for this uncertainty. Further increases in biomass supply are not considered because increasing extreme weather makes this condition improbable, and any expansion of biomass appropriation may impact other environmental boundaries, such as biodiversity [120]. Reference[119] argue, that human appropriation of biomass (HANPP) can be used as a proxy for biosphere integrity, and that current rates are "well beyond a precautionary planetary boundary".

The *maximum possible* estimate for NEE is 249 EJ/yr. This is comparable with the estimated NEE generation in the Net Zero Energy (NZE) scenario, created for the IEA's updated 2023 report [159] of 260 EJ/yr (sum of renewables, nuclear, hydrogen and ammonia minus bioenergy). The report describes the required infrastructure ramp-up requirements as "an enormous undertaking" [159]. This is consistent with the premise of our *maximum possible* estimate, calculated as follows:

- Wind and solar generation are estimated to be **180 EJ**, twice the *maximum probable* value (found in Section 3.1.2) to account for the uncertainty in curve

fitting approaches. These can be sensitive to the number of data points available for the regression (as discussed by reference[71]). This effect is demonstrated in Figure S8, where using an additional four years of historical data (22 rather than 18 years) led to a doubling of the projected solar and wind generation in 2050.

- Nuclear generation is estimated as **28 EJ/yr,** the upper end of the range estimated by the nuclear industry of 15 - 28 EJ/yr [160].
- Geothermal electricity generation is estimated assuming successful implementation of new-generation geothermal (described on page 51). A dramatic 50-fold increase from today to around **15 EJ/yr** is assumed. The reference[76] propose that 800 GW capacity might be possible by 2050 which would equate to around 19 EJ/yr at a 75% capacity factor. A lower value is used because current geothermal investments appear very small, based on data published by reference[161].
- Hydropower generation is estimated as **25 EJ/yr**, the same as the *maximum probable* value, because that estimated value will be challenging to achieve, as discussed on page 71.

The *maximum possible* estimate for Carbon Storage is derived from the analysis of reference[78]. Reference[78], derived 'feasibility frontiers' for carbon storage in 2030 using data of planned developments and failure rates. The database they compile show two clear 'waves' in project plans (Figure S21): The first wave had a high failure rate (88%) but, given five-year+ build times, there is no evidence yet whether this will change for the second wave.

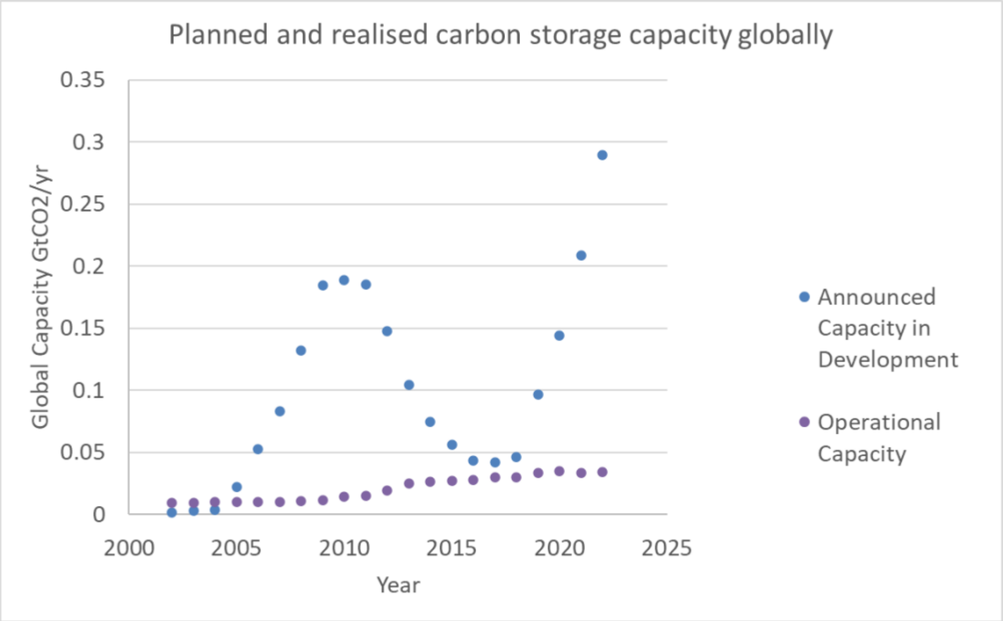

Figure S21 - Planned and realised carbon storage capacity globally, using the data compiled by reference[78].

The basis for their projections to 2040 and beyond uses maximum observed acceleration growth rates for solar, nuclear and wind technologies to define feasibility constraints. These are used (alongside the 2030 feasibility constraint) to filter IPCC AR6 1.5°C and 2°C pathways (IPCC categories 1-4). Assuming the most optimistic constraint on 2030 deployment (assuming that current plans double and failure rates drop dramatically from 88% to 45%) and 2040 acceleration is constrained by the rate of any of wind, solar or nuclear, 2050 carbon storage capacity has the median value of 3.7 GtCO2/yr (inter-quartile range: 3-4.4 GtCO2/yr, full range: 0.035–5.3 GtCO2/yr).

The *maximum possible* carbon storage is therefore estimated to be **3.7 Gt/yr**. This assumes a 70% capacity factor (see the discussion from page 56) applied to the maximum 2050 capacity from any scenario considered feasible by reference[78] under the most optimistic assumptions. Those assumptions are very optimistic, as needed to derive 'feasibility frontiers', rather than plausible expectations. Acceleration rates for instance are likely to be significantly lower: nuclear, solar and wind do not encounter the challenges associated with pipeline transport and geological storage, and were driven by the contexts of oil and energy crises. The *maximum possible* value may, therefore, be overly optimistic, but has been used in the absence of alternative analyses.

	**Biomass**	**NEE**	**Carbon storage**
**Lower risk**	Maximum probable estimate -15% to account for the range of error found in the 2018 validation **(12.8 EJ/yr)**	Linear, based on 2010-2020 **(70 EJ/yr)**	Linear, based on 2010-2020 **(0.1 Mt/yr)**
**Maximum probable**	Increase in residue use and yield gaps close **(15.8 Gt/yr)**	Aggregated estimates for each type of NEE:<br>• Wind/solar: **90 EJ/yr**<br>• Nuclear: **16 EJ/yr**<br>• Hydropower: **26 EJ/yr**<br>• Geothermal: **0.6 EJ/yr**<br>TOTAL: 133 EJ/yr	Exponential fit to CCS data pre-2019 (700 Mt/yr capacity)<br>'Capacity factor': 70%<br>Operating rate: 500 Mt/yr
**Maximum possible**	Maximum probable estimate +15% to account for the range of error found in the 2018 validation **(18.2 EJ/yr)**	Aggregated estimates for each type of NEE:<br>• Wind/solar: **180 EJ/yr**<br>• Nuclear: **28 EJ/yr**<br>• Hydropower: **26 EJ/yr**<br>• Geothermal: **15 EJ/yr**<br>TOTAL: 249 EJ/yr	1.5- and 2-degree scenario pathways within the most optimistic constraints of Kazlou et al. (2024)[78] suggest max capacity of 5.3 Gt/yr.<br>'Capacity factor': 70%<br>Operating rate: 3.7 Gt/yr

Table S19 – Summary of approaches and values used for the estimates of 2050 supply. The 'lower risk' and 'maximum possible' values are justified in the main text of this section. Detailed justifications for the 'maximum probable' values are given in Section 3.1.

4.2.2 Uncertainty in model coefficients

The model depends on many data inputs in the coefficients, each of which include uncertainty because it is impossible to know the future exact global average resource demands of a potential (perhaps currently uncommercialised) process. A sensitivity analysis would reveal which of the model coefficients has the greatest influence on the model results. This could be useful for future work, if it aims to focus on the distribution of technologies and activities possible in a net-zero future. In this analysis however, the aim is to understand risk inherent in existing proposals. A more pragmatic uncertainty analysis approach therefore is used to understand if the range of possible uncertainty in the coefficients is likely to affect the conclusions.

The impact of uncertainty in the model coefficients is explored by assuming all coefficients (except functional flows) have normally distributed uncertainty. The analysis first considers the potential spread in results and impact on the conclusions of a likely level of uncertainty. A variance of 10% is used for the normally distributed uncertainty - following guidance for LCI process data without uncertainty information, given by reference[162] - for Monte Carlo analysis using model inputs (activity rates and process shares) set according to key policy combinations. This is shown in Figure 5 of the main paper.

This analysis is extended by considering how much uncertainty in the model coefficients would be needed to affect the conclusions. The Monte Carlo analysis is repeated using increasing variance. Figure S22 shows the risk of supply shortages in at least one ZER remain very high, even if the variance in model coefficients is expanded to ±40%. Further analysis found that if the coefficient variance is expanded to ±50%, demand for emissions-free electricity and biomass can be met in 10% of the scenarios but the supply of carbon storage is still insufficient. Even if the maximum probable supply is expanded ten times, only in 5% of the scenarios with 50% variance in model coefficients can supply meet demand.

Monte Carlo analysis used in this way is useful to propagate input uncertainty but does not account for potential correlations between parameters, and the impact on output uncertainty. For example, if the emissions factor for methane were an underestimate, all coefficients for emissions derived using this factor would also be underestimated (they have a co-dependency and are therefore correlated). The output error would

therefore be larger than if one uncorrelated coefficient were underestimated. This effect is not represented by the Monte Carlo analysis, which picks each coefficient randomly from the given distribution. If greater precision in the model output is needed, the impact of co-dependencies could be assessed. The current work is instead focused on revealing supply risks which have not previously been considered, and so a higher level of uncertainty is acceptable.

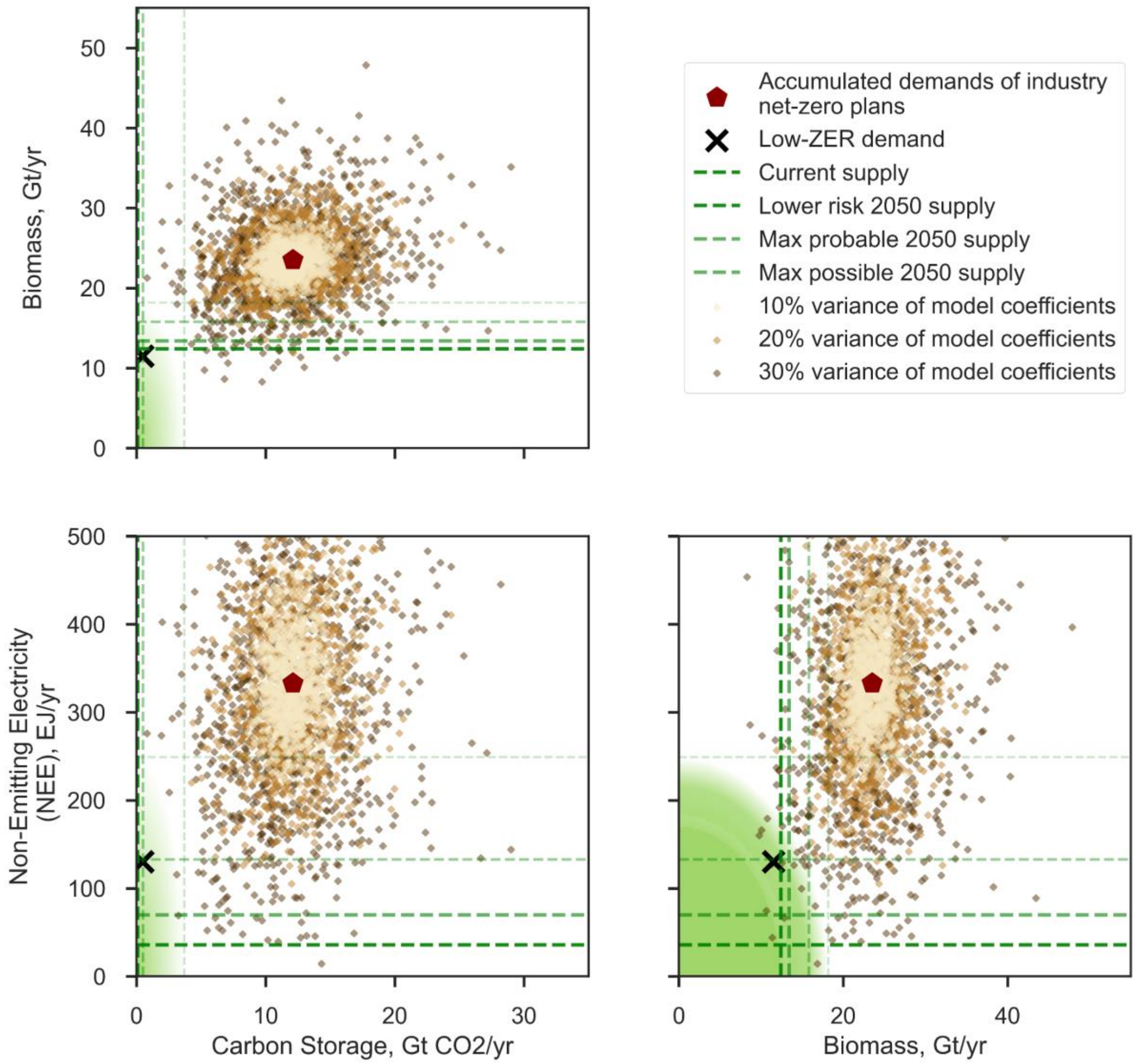

Figure S22 - Demand for ZERs of global net-zero proposals (generated by the ZERs model) contrasted with probable supply indicated by the shaded green region in the bottom left corner. The three graphs of the figure contrast demand and supply of the three zero-emissions resources pairwise. The green region on each graph defines the expected supply of the three resources (Section 4.2.1). The clouds of points arise from a thousand runs, with calculator coefficients selected randomly in a normal distribution of the values reported in SI Part 5. The variance of the uncertainty distribution is shown by the shading. The 'lower risk' and 'max possible' supply are from the analysis in Section 4.2.1.

References (Parts 1-4)

(1) IPCC. Summary for Policymakers. In *Global Warming of 1.5°C. An IPCC Special Report on the impacts of global warming of 1.5°C above pre-industrial levels and related global greenhouse gas emission pathways, in the context of strengthening the global response to the threat of climate change,*; Masson-Delmotte, V., Zhai, P., Pörtner, H.-O., Roberts, D., Skea, J., Shukla, P. R., Pirani, A., Moufouma-Okia, W., Péan, C., Pidcock, R., Connors, S., Matthews, J. B. R., Chen, Y., Zhou, X., Gomis, M. I., Lonnoy, E., Maycock, T., Tignor, M., Waterfield, T., Eds.; Cambridge University Press, 2018.

(2) Brutschin, E.; Pianta, S.; Tavoni, M.; Riahi, K.; Bosetti, V.; Marangoni, G.; van Ruijven, B. J. A Multidimensional Feasibility Evaluation of Low-Carbon Scenarios. *Environmental Research Letters* **2021**, *16* (6), 064069. https://doi.org/10.1088/1748-9326/abf0ce.

(3) Jewell, J.; Cherp, A. On the Political Feasibility of Climate Change Mitigation Pathways: Is It Too Late to Keep Warming below 1.5°C? *WIREs Climate Change* **2020**, *11* (1), 1–12. https://doi.org/10.1002/wcc.621.

(4) IPCC. Annex I: Glossary. In *Climate Change 2022: Mitigation of Climate Change. Contribution of Working Group III to the Sixth Assessment Report of the Intergovernmental Panel on Climate Change*; van Diemen, R., Matthews, J. B. R., Möller, V., Fuglestvedt, J. S., Masson-Delmotte, V., Méndez, C., Reisinger, A., Semenov, S., Eds.; Cambridge University Press: Cambridge, UK and New York, NY, USA, 2022. https://doi.org/10.1017/9781009157926.020.

(5) Zscheischler, J.; Westra, S.; van den Hurk, B. J. J. M.; Seneviratne, S. I.; Ward, P. J.; Pitman, A.; AghaKouchak, A.; Bresch, D. N.; Leonard, M.; Wahl, T.; Zhang, X. Future Climate Risk from Compound Events. *Nat Clim Chang* **2018**, *8* (6), 469–477. https://doi.org/10.1038/s41558-018-0156-3.

(6) Mackie, E.; Connon, I.; Workman, M.; Gilbert, A.; Shuckburgh, E. *Climate Risk Decision-Making: Translation of Decision Support into Policy*; 2022. https://doi.org/10.33774/coe-2022-qnd2z.

(7) Jewell, J.; Cherp, A. The Feasibility of Climate Action: Bridging the inside and the Outside View through Feasibility Spaces. *WIREs Climate Change* **2023**, *14* (5), e838. https://doi.org/https://doi.org/10.1002/wcc.838.

(8) Solomon, S.; Plattner, G.-K.; Knutti, R.; Friedlingstein, P. Irreversible Climate Change Due to Carbon Dioxide Emissions. *Proceedings of the National Academy of Sciences* **2009**, *106* (6), 1704–1709. https://doi.org/10.1073/pnas.0812721106.

(9) Matthews, H. D.; Gillett, N. P.; Stott, P. A.; Zickfeld, K. The Proportionality of Global Warming to Cumulative Carbon Emissions. *Nature* **2009**, *459* (7248), 829–832. https://doi.org/10.1038/nature08047.

(10) Allen, M. R.; Frame, D. J.; Huntingford, C.; Jones, C. D.; Lowe, J. A.; Meinshausen, M.; Meinshausen, N. Warming Caused by Cumulative Carbon Emissions towards the Trillionth Tonne. *Nature* **2009**, *458* (7242), 1163–1166. https://doi.org/10.1038/nature08019.

(11) MacDougall, A. H.; Frölicher, T. L.; Jones, C. D.; Rogelj, J.; Matthews, H. D.; Zickfeld, K.; Arora, V. K.; Barrett, N. J.; Brovkin, V.; Burger, F. A.; Eby, M.; Eliseev, A. V; Hajima, T.; Holden, P. B.; Jeltsch-Thömmes, A.; Koven, C.; Mengis, N.; Menviel, L.; Michou, M.; Mokhov, I. I.; Oka, A.; Schwinger, J.; Séférian, R.; Shaffer, G.; Sokolov, A.; Tachiiri, K.; Tjiputra, J.; Wiltshire, A.; Ziehn, T. Is There Warming in the Pipeline? A Multi-Model Analysis of the Zero Emissions Commitment from \chem{CO_2}. *Biogeosciences* **2020**, *17* (11), 2987–3016. https://doi.org/10.5194/bg-17-2987-2020.

(12) Palazzo Corner, S.; Siegert, M.; Ceppi, P.; Fox-Kemper, B.; Frölicher, T. L.; Gallego-Sala, A.; Haigh, J.; Hegerl, G. C.; Jones, C. D.; Knutti, R.; Koven, C. D.; MacDougall, A. H.; Meinshausen, M.; Nicholls, Z.; Sallée, J. B.; Sanderson, B. M.; Séférian, R.; Turetsky, M.; Williams, R. G.; Zaehle, S.; Rogelj, J. The Zero Emissions Commitment and Climate Stabilization. *Frontiers in Science* **2023**, *1* (November), 1–26. https://doi.org/10.3389/fsci.2023.1170744.

(13) Reisinger, A.; Khourdajie, A. Al; Blok, K.; Clark, H.; Cowie, A.; Fuglestvedt, J. S.; Geden, O.; Ginzburg, V.; Guivarch, C.; House, J. I.; Minx, J. C.; Mrabet, R.; Nabuurs, G.-J.; Peters, G. P.; Riahi, K.; Schaeffer, R.; Slade, R.; Strømman, A. H.; Vuuren, D. P. van. Cross-Chapter Box 2 - GHG Emissions Metrics. In *IPCC, 2022: Climate Change 2022: Mitigation of Climate Change. Contribution of Working Group III to the Sixth Assessment Report of the Intergovernmental Panel on Climate Change*; 2022.

(14) Matthews, H. D.; Zickfeld, K. Climate Response to Zeroed Emissions of Greenhouse Gases and Aerosols. *Nat Clim Chang* **2012**, *2* (5), 338–341. https://doi.org/10.1038/nclimate1424.

(15) Rogelj, J.; Geden, O.; Cowie, A.; Reisinger, A. Net-Zero Emissions Targets Are Vague: Three Ways to Fix. *Nature* **2021**, *591* (7850), 365–368. https://doi.org/10.1038/d41586-021-00662-3.

(16) IPCC. Summary for Policymakers. In *Global Warming of 1.5°C. An IPCC Special Report on the impacts of global warming of 1.5°C above pre-industrial levels and related global greenhouse gas emission pathways, in the context of strengthening the global response to the threat of climate change,*; Cambridge University Press, 2018.

(17) Rogelj, J.; Shindell, D.; Jiang, K.; Fifita, S.; Forster, P.; Ginzburg, V.; Handa, C.; Kheshgi, H.; Kobayashi, S.; Kriegler, E.; Mundaca, L.; Séférian, R.; Vilariño, M. V. Mitigation Pathways Compatible with 1.5°C in the Context of Sustainable Development. In *Global Warming of 1.5°C. An IPCC Special Report on the impacts of global warming of 1.5°C above pre-industrial levels and related global greenhouse gas emission pathways, in the context of strengthening the global response to the threat of climate change,*; Masson-Delmotte, V., Zhai, P., Pörtner, H.-O., Roberts, D., Skea, J., Shukla, P. R., Pirani, A., Moufouma-Okia, W., Péan, C., Pidcock, R., Connors, S., Matthews, J. B. R., Chen, Y., Zhou, X., Gomis, M. I., Lonnoy, E., Maycock, T., Tignor, M., Waterfield, T., Eds.; Cambridge University Press, 2018. https://doi.org/10.1017/9781009157940.004.

(18) Fankhauser, S.; Smith, S. M.; Allen, M.; Axelsson, K.; Hale, T.; Hepburn, C.; Kendall, J. M.; Khosla, R.; Lezaun, J.; Mitchell-Larson, E.; Obersteiner, M.; Rajamani, L.; Rickaby, R.; Seddon, N.; Wetzer, T. The Meaning of Net Zero and How to Get It Right. *Nat Clim Chang* **2022**, *12* (1), 15–21. https://doi.org/10.1038/s41558-021-01245-w.

(19) Climate Action Tracker. *Warming Projections Global Update*; 2023. https://climateactiontracker.org/publications/no-change-to-warming-as-fossil-fuel-endgame-brings-focus-onto-false-solutions/.

(20) Ou, Y.; Roney, C.; Alsalam, J.; Calvin, K.; Creason, J.; Edmonds, J.; Fawcett, A. A.; Kyle, P.; Narayan, K.; O'Rourke, P.; Patel, P.; Ragnauth, S.; Smith, S. J.; McJeon, H. Deep Mitigation of CO2 and Non-CO2 Greenhouse Gases toward 1.5 °C and 2 °C Futures. *Nat Commun* **2021**, *12* (1), 6245. https://doi.org/10.1038/s41467-021-26509-z.

(21) Pierrehumbert, R. T. Short-Lived Climate Pollution. *Annu Rev Earth Planet Sci* **2014**, *42* (Volume 42, 2014), 341–379. https://doi.org/https://doi.org/10.1146/annurev-earth-060313-054843.

(22) Fuglestvedt, J.; Rogelj, J.; Millar, R. J.; Allen, M.; Boucher, O.; Cain, M.; Forster, P. M.; Kriegler, E.; Shindell, D. Implications of Possible Interpretations of 'Greenhouse Gas Balance' in the Paris Agreement. *Philosophical Transactions of the Royal Society A: Mathematical, Physical and Engineering Sciences* **2018**, *376* (2119), 20160445. https://doi.org/10.1098/rsta.2016.0445.

(23) Eggleston, H. S.; Buendia, L.; Miwa, K.; Ngara, T.; Tanabe, K. 2006 IPCC Guidelines for National Greenhouse Gas Inventories. **2006**.

(24) Meng, F.; Wagner, A.; Kremer, A. B.; Kanazawa, D.; Leung, J. J.; Goult, P.; Guan, M.; Herrmann, S.; Speelman, E.; Sauter, P.; Lingeswaran, S.; Stuchtey, M. M.; Hansen, K.; Masanet, E.; Serrenho, A. C.; Ishii, N.; Kikuchi, Y.; Cullen, J. M. Planet-Compatible Pathways for Transitioning the Chemical Industry. *Proceedings of the National Academy of Sciences* **2023**, *120* (8), e2218294120. https://doi.org/10.1073/pnas.2218294120.

(25) Hawkins, W. Timber and Carbon Sequestration. *Structural Engineer* **2021**, *99* (1), 18–20.

(26) McLaren, D.; Markusson, N. The Co-Evolution of Technological Promises, Modelling, Policies and Climate Change Targets. *Nat Clim Chang* **2020**, *10* (5), 392–397. https://doi.org/10.1038/s41558-020-0740-1.

(27) Houghton, R. A.; Nassikas, A. A. Global and Regional Fluxes of Carbon from Land Use and Land Cover Change 1850–2015. *Global Biogeochem Cycles* **2017**, *31* (3), 456–472. https://doi.org/10.1002/2016GB005546.

(28) IPCC. *Climate Change and Land: An IPCC Special Report on Climate Change, Desertification, Land Degradation, Sustainable Land Management, Food Security, and Greenhouse Gas Fluxes in Terrestrial Ecosystems*; Shukla, P. R., Skea, J., Buendia, E. C., Masson-Delmotte, V., Pörtner, H.-O., Roberts, D. C., Zhai, P., Slade, R., Connors, S., Diemen, R. van, Ferrat, M., Haughey, E., Luz, S., Neogi, S., Pathak, M., Petzold, J., Pereira, J. P., Vyas, P., Huntley, E., Kissick, K., Belkacemi, M., Malley, J., Eds.; 2019.

(29) Babiker, M.; Berndes, G.; Blok, K.; Cohen, B.; Cowie, A.; Geden, O.; Ginzburg, V.; Leip, A.; Smith, P.; Sugiyama, M. Cross-Sectoral Perspectives. In *Climate Change 2022 - Mitigation of Climate Change*; Cambridge University Press, 2022; pp 295–408. https://doi.org/10.1017/9781009157926.005.

(30) Anderegg, W. R. L.; Trugman, A. T.; Badgley, G.; Anderson, C. M.; Bartuska, A.; Ciais, P.; Cullenward, D.; Field, C. B.; Freeman, J.; Goetz, S. J.; Hicke, J. A.; Huntzinger, D.; Jackson, R. B.; Nickerson, J.; Pacala, S.; Randerson, J. T. Climate-Driven Risks to the Climate Mitigation Potential of Forests. *Science (1979)* **2020**, *368* (6497), eaaz7005. https://doi.org/10.1126/science.aaz7005.

(31) Seidl, R.; Thom, D.; Kautz, M.; Martin-Benito, D.; Peltoniemi, M.; Vacchiano, G.; Wild, J.; Ascoli, D.; Petr, M.; Honkaniemi, J.; Lexer, M. J.; Trotsiuk, V.; Mairota, P.; Svoboda, M.; Fabrika, M.; Nagel, T. A.; Reyer, C. P. O. Forest Disturbances under Climate Change. *Nat Clim Chang* **2017**, *7* (6), 395–402. https://doi.org/10.1038/nclimate3303.

(32) Bellassen, V.; Luyssaert, S. Carbon Sequestration: Managing Forests in Uncertain Times. *Nature* **2014**, *506* (7487), 153–155. https://doi.org/10.1038/506153a.

(33) Hubau, W.; Lewis, S. L.; Phillips, O. L.; Affum-Baffoe, K.; Beeckman, H.; Cuní-Sanchez, A.; Daniels, A. K.; Ewango, C. E. N.; Fauset, S.; Mukinzi, J. M.; Sheil, D.; Sonké, B.; Sullivan, M. J. P.; Sunderland, T. C. H.; Taedoumg, H.; Thomas, S. C.; White, L. J. T.; Abernethy, K. A.; Adu-Bredu, S.; Amani, C. A.; Baker, T. R.; Banin, L. F.; Baya, F.; Begne, S. K.; Bennett, A. C.; Benedet, F.; Bitariho, R.; Bocko, Y. E.; Boeckx, P.; Boundja, P.; Brienen, R. J. W.; Brncic, T.; Chezeaux, E.; Chuyong, G. B.; Clark, C. J.; Collins, M.; Comiskey, J. A.; Coomes, D. A.; Dargie, G. C.; de Haulleville, T.; Kamdem, M. N. D.; Doucet, J.-L.; Esquivel-Muelbert, A.; Feldpausch, T. R.; Fofanah, A.; Foli, E. G.; Gilpin, M.; Gloor, E.; Gonmadje, C.; Gourlet-Fleury, S.; Hall, J. S.; Hamilton, A. C.; Harris, D. J.; Hart, T. B.; Hockemba, M. B. N.; Hladik, A.; Ifo, S. A.; Jeffery, K. J.; Jucker, T.; Yakusu, E. K.; Kearsley, E.; Kenfack, D.; Koch, A.; Leal, M. E.; Levesley, A.; Lindsell, J. A.; Lisingo, J.; Lopez-Gonzalez, G.; Lovett, J. C.; Makana, J.-R.; Malhi, Y.; Marshall, A. R.; Martin, J.; Martin, E. H.; Mbayu, F. M.; Medjibe, V. P.; Mihindou, V.; Mitchard, E. T. A.; Moore, S.; Munishi, P. K. T.; Bengone, N. N.; Ojo, L.; Ondo, F. E.; Peh, K. S.-H.; Pickavance, G. C.; Poulsen, A. D.; Poulsen, J. R.; Qie, L.; Reitsma, J.; Rovero, F.; Swaine, M. D.; Talbot, J.; Taplin, J.; Taylor, D. M.; Thomas, D. W.; Toirambe, B.; Mukendi, J. T.; Tuagben, D.; Umunay, P. M.; van der Heijden, G. M. F.; Verbeeck, H.; Vleminckx, J.; Willcock, S.; Wöll, H.; Woods, J. T.; Zemagho, L. Asynchronous Carbon Sink Saturation in African and Amazonian Tropical Forests. *Nature* **2020**, *579* (7797), 80–87. https://doi.org/10.1038/s41586-020-2035-0.

(34) Kirschbaum, M. U. F.; Cowie, A. L.; Peñuelas, J.; Smith, P.; Conant, R. T.; Sage, R. F.; Brandão, M.; Cotrufo, M. F.; Luo, Y.; Way, D. A.; Robinson, S. A. Is Tree Planting an

Effective Strategy for Climate Change Mitigation? *Science of The Total Environment* **2024**, *909*, 168479. https://doi.org/https://doi.org/10.1016/j.scitotenv.2023.168479.

(35) van der Werf, G. R.; Morton, D. C.; DeFries, R. S.; Olivier, J. G. J.; Kasibhatla, P. S.; Jackson, R. B.; Collatz, G. J.; Randerson, J. T. CO2 Emissions from Forest Loss. *Nat Geosci* **2009**, *2* (11), 737–738. https://doi.org/10.1038/ngeo671.

(36) The Royal Society. *Geoengineering the Climate: Science, Governance and Uncertainty | Royal Society*; London, 2009.

(37) Groom, B.; Venmans, F. The Social Value of Offsets. *Nature* **2023**, *619* (7971), 768–773. https://doi.org/10.1038/s41586-023-06153-x.

(38) Powis, C. M.; Smith, S. M.; Minx, J. C.; Gasser, T. Quantifying Global Carbon Dioxide Removal Deployment. *Environmental Research Letters* **2023**, *10*, 22408–22418. https://doi.org/10.1088/1748-9326/acb450.

(39) C Zarakas; G Badgley; F Chay. *Comparing carbon removal at different timescales*. CarbonPlan. https://carbonplan.org/research/cdr-timescale-accounting (accessed 2024-01-04).

(40) Minx, J. C.; Lamb, W. F.; Callaghan, M. W.; Fuss, S.; Hilaire, J.; Creutzig, F.; Amann, T.; Beringer, T.; De Oliveira Garcia, W.; Hartmann, J.; Khanna, T.; Lenzi, D.; Luderer, G.; Nemet, G. F.; Rogelj, J.; Smith, P.; Vicente Vicente, J. L.; Wilcox, J.; Del Mar Zamora Dominguez, M. Negative Emissions - Part 1: Research Landscape and Synthesis. *Environmental Research Letters*. 2018. https://doi.org/10.1088/1748-9326/aabf9b.

(41) Balmford, A.; Keshav, S.; Venmans, F.; Coomes, D.; Groom, B.; Madhavapeddy, A.; Swinfield, T. Realizing the Social Value of Impermanent Carbon Credits. *Nat Clim Chang* **2023**, *13* (11), 1172–1178. https://doi.org/10.1038/s41558-023-01815-0.

(42) Nemet, G. F.; Callaghan, M. W.; Creutzig, F.; Fuss, S.; Hartmann, J.; Hilaire, J.; Lamb, W. F.; Minx, J. C.; Rogers, S.; Smith, P. Negative Emissions—Part 3: Innovation and Upscaling. *Environmental Research Letters* **2018**, *13* (6), 063003. https://doi.org/10.1088/1748-9326/aabff4.

(43) Fuss, S.; Lamb, W. F.; Callaghan, M. W.; Hilaire, J.; Creutzig, F.; Amann, T.; Beringer, T.; de Oliveira Garcia, W.; Hartmann, J.; Khanna, T.; Luderer, G.; Nemet, G. F.; Rogelj, J.; Smith, P.; Vicente, J. V. L. V.; Wilcox, J.; Del Mar Zamora Dominguez, M.; Minx, J. C. Negative Emissions - Part 2: Costs, Potentials and Side Effects.

Environmental Research Letters **2018**, *13* (6), 63002. https://doi.org/10.1088/1748-9326/aabf9f.

(44) Chay, F.; Klitzke, J.; Hausfather, Z.; Martin, K.; Freeman, J.; Cullenward, D. *Verification Confidence Levels for carbon dioxide removal*. CarbonPlan. https://carbonplan.org/research/cdr-verification-explainer (accessed 2024-01-02).

(45) DeAngelo, J.; Saenz, B. T.; Arzeno-Soltero, I. B.; Frieder, C. A.; Long, M. C.; Hamman, J.; Davis, K. A.; Davis, S. J. Economic and Biophysical Limits to Seaweed Farming for Climate Change Mitigation. *Nat Plants* **2023**, *9* (1), 45–57. https://doi.org/10.1038/s41477-022-01305-9.

(46) Cullenward, D. *A Framework for Assessing The Climate Value of Temporary Carbon Storage*; 2023. https://carbonmarketwatch.org/publications/a-framework-for-assessing-the-climate-value-of-temporary-carbon-storage/.

(47) Matthews, H. D.; Zickfeld, K.; Koch, A.; Luers, A. Accounting for the Climate Benefit of Temporary Carbon Storage in Nature. *Nat Commun* **2023**, *14* (1), 5485. https://doi.org/10.1038/s41467-023-41242-5.

(48) Dhakal, S.; Minx, J. C.; Toth, F. L.; Abdel-Aziz, A.; Venezuela, M. J. F. M.; Hubacek, K.; Jonckheere, I. G. C.; Kim, Y.-G.; Nemet, G. F.; Pachauri, S.; Tan, X. C.; Wiedmann, T. Emissions Trends and Drivers. In *IPCC, 2022: Climate Change 2022: Mitigation of Climate Change. Contribution of Working Group III to the Sixth Assessment Report of the Intergovernmental Panel on Climate Change*; Shukla, P. R., Skea, J., Slade, R., Khourdajie, A. Al, Diemen, R. van, McCollum, D., Pathak, M., Some, S., Vyas, P., Fradera, R., Belkacemi, M., Hasija, A., Lisboa, G., Luz, S., Malley, J., Eds.; Cambridge University Press: Cambridge, UK and New York, NY, USA, 2022; pp 215–294. https://doi.org/10.1017/9781009157926.004.

(49) MacCarthy, J.; Richter, J.; Tyukavina, S.; Weisse, M.; Harris, N. *The latest data confirms: Forest fires are getting worse*. World Resources Institute. www.wri.org/insights/global-trends-forest-fires (accessed 2024-02-08).

(50) Rose, D. J.; Hemery, L. G. Methods for Measuring Carbon Dioxide Uptake and Permanence: Review and Implications for Macroalgae Aquaculture. *Journal of Marine Science and Engineering*. 2023. https://doi.org/10.3390/jmse11010175.

(51) Heck, V.; Gerten, D.; Lucht, W.; Popp, A. Biomass-Based Negative Emissions Difficult to Reconcile with Planetary Boundaries. *Nat Clim Chang* **2018**, *8* (2), 151–155. https://doi.org/10.1038/s41558-017-0064-y.

(52) Nabuurs, G.-J.; Mrabet, R. Agriculture, Forestry and Other Land Uses (AFOLU). In *IPCC, 2022: Climate Change 2022: Mitigation of Climate Change. Contribution of Working Group III to the Sixth Assessment Report of the Intergovernmental Panel on Climate Change*; 2022; pp 747–860. https://doi.org/10.1017/9781009157926.009.

(53) Macreadie, P. I.; Costa, M. D. P.; Atwood, T. B.; Friess, D. A.; Kelleway, J. J.; Kennedy, H.; Lovelock, C. E.; Serrano, O.; Duarte, C. M. Blue Carbon as a Natural Climate Solution. *Nat Rev Earth Environ* **2021**, *2* (12), 826–839. https://doi.org/10.1038/s43017-021-00224-1.

(54) The Royal Society; The Royal Academy of Engineering. *Greenhouse Gas Removal*; 2018. https://royalsociety.org/news-resources/projects/greenhouse-gas-removal/.

(55) (carbon)plan. *CDR Verification Framework*. https://carbonplan.org/research/cdr-verification (accessed 2024-06-07).

(56) Nakićenović, N.; Gilli, P. V.; Kurz, R. Regional and Global Exergy and Energy Efficiencies. *Energy* **1996**, *21* (3), 223–237. https://doi.org/10.1016/0360-5442(96)00001-1.

(57) Nakićenović, N.; Grübler, A.; Inaba, A.; Messner, S.; Nilsson, S.; Nishimura, Y.; Rogner, H.-H.; Schäfer, A.; Schrattenholzer, L.; Strubegger, M.; Swisher, J.; Victor, D.; Wilson, D. Long-Term Strategies for Mitigating Global Warming. **1993**, *18* (5). https://doi.org/10.1016/0360-5442(93)90019-A.

(58) Cullen, J. M.; Allwood, J. M. The Efficient Use of Energy: Tracing the Global Flow of Energy from Fuel to Service. *Energy Policy* **2010**, *38* (1), 75–81. https://doi.org/10.1016/j.enpol.2009.08.054.

(59) Pauliuk, S.; Heeren, N.; Berrill, P.; Fishman, T.; Nistad, A.; Tu, Q.; Wolfram, P.; Hertwich, E. G. Global Scenarios of Resource and Emission Savings from Material Efficiency in Residential Buildings and Cars. *Nat Commun* **2021**, *12* (1), 5097. https://doi.org/10.1038/s41467-021-25300-4.

(60) Creutzig, F.; Niamir, L.; Bai, X.; Callaghan, M.; Cullen, J.; Díaz-José, J.; Figueroa, M.; Grubler, A.; Lamb, W. F.; Leip, A.; Masanet, E.; Mata, É.; Mattauch, L.; Minx, J. C.; Mirasgedis, S.; Mulugetta, Y.; Nugroho, S. B.; Pathak, M.; Perkins, P.; Roy, J.; de la

Rue du Can, S.; Saheb, Y.; Some, S.; Steg, L.; Steinberger, J.; Ürge-Vorsatz, D. Demand-Side Solutions to Climate Change Mitigation Consistent with High Levels of Well-Being. *Nat Clim Chang* **2021**, *12* (January). https://doi.org/10.1038/s41558-021-01219-y.

(61) Wiedenhofer, D.; Streeck, J.; Wiese, F.; Verdolini, E.; Mastrucci, A.; Ju, Y.; Boza-Kiss, B.; Min, J.; Norman, J.; Wieland, H.; Bento, N.; León, M. F. G.; Magalar, L.; Mayer, A.; Gingrich, S.; Hayashi, A.; Jupesta, J.; Ünlü, G.; Niamir, L.; Cao, T.; Zanon-Zotin, M.; Plank, B.; Vélez-Henao, J.; Masanet, E.; Krey, V.; Akimoto, K.; Grubler, A.; van Ruijven, B.; Pauliuk, S. Industry Transformations for High Service Provisioning with Lower Energy and Material Demand: A Review of Models and Scenarios. *Annu Rev Environ Resour* **2024**, *49* (1), 249–279. https://doi.org/10.1146/annurev-environ-110822-044428.

(62) Department for Business Energy & Industrial Strategy. *Greenhouse Gas Reporting: Conversion Factors*; 2021.

(63) Gómez, D.; Watterson, J.; Americano, B.; Ha, C.; Marland, G.; Matsika, E.; Nenge Namayanga, L.; Osman-Elasha, B.; Kalenga Saka, J.; Treanton, K.; Quadrelli, R. 2.1 Stationary Combustion. In *IPCC Guidelines for National Greenhouse Gas Inventories*; IPCC, 2006.

(64) International Energy Agency - IEA. *World Energy Balances Database Documentation*; 2020. https://stats2.digitalresources.jisc.ac.uk/metadata/IEA/WEB/World_Energy_Balances_2020_Documentation.pdf.

(65) Faramawy, S.; Zaki, T.; Sakr, A. A.-E. Natural Gas Origin, Composition, and Processing: A Review. *J Nat Gas Sci Eng* **2016**, *34*, 34–54. https://doi.org/https://doi.org/10.1016/j.jngse.2016.06.030.

(66) Smit, B. *Introduction to Carbon Capture and Sequestration Berend Smit, Jeffrey A. Reimer, Curtis M. Oldenburg, Ian C. Bourg.*; Berkeley lectures on energy ; v. 1; Imperial College P.: London, 2014.

(67) Hill, B.; Hovorka, S.; Melzer, S. Geologic Carbon Storage through Enhanced Oil Recovery. *Energy Procedia* **2013**, *37*, 6808–6830. https://doi.org/10.1016/j.egypro.2013.06.614.

(68) Cherp, A.; Vinichenko, V.; Tosun, J.; Gordon, J. A.; Jewell, J. National Growth Dynamics of Wind and Solar Power Compared to the Growth Required for Global Climate Targets. *Nat Energy* **2021**, *6* (7), 742–754. https://doi.org/10.1038/s41560-021-00863-0.

(69) Cherp, A.; Vinichenko, V.; Jewell, J.; Suzuki, M.; Antal, M. Comparing Electricity Transitions: A Historical Analysis of Nuclear, Wind and Solar Power in Germany and Japan. *Energy Policy* **2017**, *101*, 612–628. https://doi.org/https://doi.org/10.1016/j.enpol.2016.10.044.

(70) Grubb, M.; Drummond, P.; Hughes, N. The Shape and Pace of Change in the Electricity Transition. **2020**, No. October.

(71) Sorrell, S.; Speirs, J. Using Growth Curves to Forecast Regional Resource Recovery: Approaches, Analytics and Consistency Tests. *Philosophical Transactions of the Royal Society A: Mathematical, Physical and Engineering Sciences* **2014**, *372* (2006), 20120317. https://doi.org/10.1098/rsta.2012.0317.

(72) Ember. *Yearly electricity data*. https://ember-climate.org/data-catalogue/yearly-electricity-data/ (accessed 2024-01-15).

(73) IAEA. *Energy, Electricity and Nuclear Power Estimates for the Period up to 2050*; 2020. https://www.iaea.org/publications/14786/energy-electricity-and-nuclear-power-estimates-for-the-period-up-to-2050 (accessed 2021-02-10).

(74) IEA. Hydropower Special Market Report. *Hydropower Special Market Report* **2021**. https://doi.org/10.1787/07a7bac8-en.

(75) IRENA. Renewable Energy Statistics 2023. Abu Dhabi 2023. https://www.irena.org/Publications/2023/Jul/Renewable-energy-statistics-2023.

(76) IEA. *The Future of Geothermal Energy*; Paris, 2024. https://www.iea.org/reports/the-future-of-geothermal-energy.

(77) McLaughlin, K.; Jaeger, J.; Womble, J.; Bird, L.; Hausker, K. Next-Generation Geothermal: Considerations and Opportunities for Responsible Development. *World Resources Institute* **2024**. https://doi.org/10.46830/wriib.24.00015.

(78) Kazlou, T.; Cherp, A.; Jewell, J. Feasible Deployment Trajectories of Carbon Capture and Storage Compared to the Requirements of Climate Targets. *Nature Climate*

Change. September 18, 2023, pp 1047–1055. https://doi.org/10.21203/rs.3.rs-3275673/v1.

(79) Global CSS Institute (GCCSI). *Global Status of CCS 2022*; 2022. https://status22.globalccsinstitute.com/wp-content/uploads/2022/12/Global-Status-of-CCS-2022_Download_1222.pdf.

(80) Gale, J.; Bradshaw, J.; Chen, Z.; Garg, A.; Gomez, D.; Bradshaw, J.; Chen, Z.; Garg, A.; Gomez, D.; Rogner, H.-H.; Simbeck, D.; Williams, R.; Toth, F.; Vuuren, D. van. Sources of CO2. In *IPCC Special Report on Carbon Dioxide Capture and Storage*; Cambridge University Press, 2005.

(81) Lane, J.; Greig, C.; Garnett, A. Uncertain Storage Prospects Create a Conundrum for Carbon Capture and Storage Ambitions. *Nat Clim Chang* **2021**, *11* (11), 925–936. https://doi.org/10.1038/s41558-021-01175-7.

(82) IEA. *Net Zero by 2050: A Roadmap for the Global Energy Sector*; 2021. https://www.iea.org/reports/net-zero-by-2050.

(83) Grubler, A.; Wilson, C.; Bento, N.; Boza-Kiss, B.; Krey, V.; McCollum, D. L.; Rao, N. D.; Riahi, K.; Rogelj, J.; De Stercke, S.; Cullen, J.; Frank, S.; Fricko, O.; Guo, F.; Gidden, M.; Havlík, P.; Huppmann, D.; Kiesewetter, G.; Rafaj, P.; Schoepp, W.; Valin, H.; Frank, S.; Krey, V.; Rogelj, J.; Rao, N. D.; Fricko, O.; Valin, H.; Rafaj, P.; Boza-Kiss, B.; Bento, N.; De Stercke, S.; Kiesewetter, G.; Havlík, P.; Gidden, M.; Wilson, C.; Riahi, K.; Schoepp, W.; Cullen, J.; Grubler, A.; McCollum, D. L.; Huppmann, D.; Wilson, C.; Bento, N.; Boza-Kiss, B.; Krey, V.; McCollum, D. L.; Rao, N. D.; Riahi, K.; Rogelj, J.; De Stercke, S.; Cullen, J.; Frank, S.; Fricko, O.; Guo, F.; Gidden, M.; Havlík, P.; Huppmann, D.; Kiesewetter, G.; Rafaj, P.; Schoepp, W.; Valin, H. A Low Energy Demand Scenario for Meeting the 1.5 °c Target and Sustainable Development Goals without Negative Emission Technologies. *Nat Energy* **2018**, *3* (6), 515–527. https://doi.org/10.1038/s41560-018-0172-6.

(84) van Ewijk, S.; McDowall, W. Diffusion of Flue Gas Desulfurization Reveals Barriers and Opportunities for Carbon Capture and Storage. *Nat Commun* **2020**, *11* (1), 4298. https://doi.org/10.1038/s41467-020-18107-2.

(85) Zahasky, C.; Krevor, S. Global Geologic Carbon Storage Requirements of Climate Change Mitigation Scenarios. *Energy Environ Sci* **2020**, *13* (6), 1561–1567. https://doi.org/10.1039/d0ee00674b.

(86) Martin-Roberts, E.; Scott, V.; Flude, S.; Johnson, G.; Haszeldine, R. S.; Gilfillan, S. Carbon Capture and Storage at the End of a Lost Decade. *One Earth* **2021**, *4* (11), 1569–1584. https://doi.org/10.1016/j.oneear.2021.10.002.

(87) Haszeldine, S. Carbon Capture and Storage. *Science (1979)* **2009**, *325* (September). https://doi.org/10.1016/C2009-0-19306-6.

(88) Haszeldine, S.; Flude, S.; Johnson, G.; Scott, V. Negative Emissions Technologies and Carbon Capture and Storage to Achieve the Paris Agreement Commitments. *Philosophical Transactions of the Royal Society A: Mathematical, Physical and Engineering Sciences*. The Royal Society Publishing May 13, 2018. https://doi.org/10.1098/rsta.2016.0447.

(89) Scott, V.; Gilfillan, S.; Markusson, N.; Chalmers, H.; Haszeldine, R. S. Last Chance for Carbon Capture and Storage. *Nature Climate Change*. 2013, pp 105–111. https://doi.org/10.1038/nclimate1695.

(90) Robertson, B.; Mousavian, M. *The Carbon Capture Crux: Lessons Learned*; 2022. https://ieefa.org/resources/carbon-capture-crux-lessons-learned.

(91) Carbon Capture and Sequestration Technologies at MIT. *Sleipner Fact Sheet: Carbon Dioxide Capture and Storage Project*. https://sequestration.mit.edu/tools/projects/sleipner.html (accessed 2023-08-07).

(92) Smyth, J.; Sheppard, D. Monster Problem: Gorgon Project Is a Test Case for Carbon Capture. *Financial Times* **2021**.

(93) The Royal Society. *Locked Away – Geological Carbon Storage*; 2022.

(94) Rai, V.; Victor, D. G.; Thurber, M. C. Carbon Capture and Storage at Scale: Lessons from the Growth of Analogous Energy Technologies. *Energy Policy* **2010**, *38* (8), 4089–4098. https://doi.org/10.1016/j.enpol.2010.03.035.

(95) Wang, N.; Akimoto, K.; Nemet, G. F. What Went Wrong? Learning from Three Decades of Carbon Capture, Utilization and Sequestration (CCUS) Pilot and Demonstration Projects. *Energy Policy* **2021**, *158* (August), 112546. https://doi.org/10.1016/j.enpol.2021.112546.

(96) Roberts, C.; Nemet, G. Lessons for Scaling Direct Air Capture from the History of Ammonia Synthesis. *Energy Res Soc Sci* **2024**, *117*, 103696. https://doi.org/10.1016/j.erss.2024.103696.

(97) Slade, R.; Bauen, A.; Gross, R. Global Bioenergy Resources. *Nat Clim Chang* **2014**, *4* (2), 99–105. https://doi.org/10.1038/nclimate2097.

(98) Krausmann, F.; Erb, K.-H.; Gingrich, S.; Lauk, C.; Haberl, H. Global Patterns of Socioeconomic Biomass Flows in the Year 2000: A Comprehensive Assessment of Supply, Consumption and Constraints. *Ecological Economics* **2008**, *65* (3), 471–487. https://doi.org/https://doi.org/10.1016/j.ecolecon.2007.07.012.

(99) Haberl, H. The Energetic Metabolism of Societies Part I: Accounting Concepts. *J Ind Ecol* **2001**, *5* (1), 11–33.

(100) Bais, A. L. S.; Lauk, C.; Kastner, T.; Erb, K. Global Patterns and Trends of Wood Harvest and Use between 1990 and 2010. *Ecological Economics* **2015**, *119*, 326–337. https://doi.org/10.1016/j.ecolecon.2015.09.011.

(101) Koopmans, A.; Koppejan, J. *Agricultural and Forest Residues - Generation, Utilization and Availability*; Kuala Lumpur, Malaysia, 1997. https://www.fao.org/3/AD576E/ad576e00.pdf.

(102) FAO. *Global Forest Resources Assessment 2005*; FAO: Rome (Italy), 2005. https://unece.org/info/publications/pub/22163.

(103) Zhou, C.; Elshkaki, A.; Graedel, T. E. Global Human Appropriation of Net Primary Production and Associated Resource Decoupling: 2010–2050. *Environ Sci Technol* **2018**, *52* (3), 1208–1215. https://doi.org/10.1021/acs.est.7b04665.

(104) Lauri, P.; Havlík, P.; Kindermann, G.; Forsell, N.; Böttcher, H.; Obersteiner, M. Woody Biomass Energy Potential in 2050. *Energy Policy* **2014**, *66*, 19–31. https://doi.org/10.1016/j.enpol.2013.11.033.

(105) Cuiping, L.; Yanyongjie; Chuangzhi, W.; Haitao, H. Study on the Distribution and Quantity of Biomass Residues Resource in China. *Biomass Bioenergy* **2004**, *27* (2), 111–117. https://doi.org/https://doi.org/10.1016/j.biombioe.2003.10.009.

(106) Bajželj, B.; Richards, K. S.; Allwood, J. M.; Smith, P.; Dennis, J. S.; Curmi, E.; Gilligan, C. A. Importance of Food-Demand Management for Climate Mitigation. *Nat Clim Chang* **2014**, *4* (10), 924–929. https://doi.org/10.1038/nclimate2353.

(107) Mottet, A.; de Haan, C.; Falcucci, A.; Tempio, G.; Opio, C.; Gerber, P. Livestock: On Our Plates or Eating at Our Table? A New Analysis of the Feed/Food Debate. *Glob Food Sec* **2017**, *14*, 1–8. https://doi.org/https://doi.org/10.1016/j.gfs.2017.01.001.

(108) FAO. *FAOSTAT Forestry Production and Trade*. https://www.fao.org/faostat/en/?#data/FO.

(109) FAO. *FAOSTAT Food Balances*. https://www.fao.org/faostat/en/?#data/FBS.

(110) Alexander, P.; Brown, C.; Arneth, A.; Finnigan, J.; Moran, D.; Rounsevell, M. D. A. Losses, Inefficiencies and Waste in the Global Food System. *Agric Syst* **2017**, *153*, 190–200. https://doi.org/10.1016/j.agsy.2017.01.014.

(111) International Energy Agency. *Dataset: International Energy Agency World Energy Balances, 1960-2020*. https://doi.org/10.5257/iea/web/2021.

(112) WRI. Course 2 Increase Food Production without Expanding Agricultural Land. In *Creating a Sustainable Food Future*; 2019.

(113) Shukla, P.; Skea, J.; Reisinger, A.; Slade, R.; Pathak, M.; Khourdajie, A. *Working Group III Contribution to the Sixth Assessment Report of the Intergovernmental Panel on Climate Change*; 2022. https://www.ipcc.ch/report/ar6/wg3/.

(114) Luyssaert, S.; Jammet, M.; Stoy, P. C.; Estel, S.; Pongratz, J.; Ceschia, E.; Churkina, G.; Don, A.; Erb, K.; Ferlicoq, M.; Gielen, B.; Grünwald, T.; Houghton, R. A.; Klumpp, K.; Knohl, A.; Kolb, T.; Kuemmerle, T.; Laurila, T.; Lohila, A.; Loustau, D.; McGrath, M. J.; Meyfroidt, P.; Moors, E. J.; Naudts, K.; Novick, K.; Otto, J.; Pilegaard, K.; Pio, C. A.; Rambal, S.; Rebmann, C.; Ryder, J.; Suyker, A. E.; Varlagin, A.; Wattenbach, M.; Dolman, A. J. Land Management and Land-Cover Change Have Impacts of Similar Magnitude on Surface Temperature. *Nat Clim Chang* **2014**, *4* (5), 389–393. https://doi.org/10.1038/nclimate2196.

(115) Foley, J. A.; Ramankutty, N.; Brauman, K. A.; Cassidy, E. S.; Gerber, J. S.; Johnston, M.; Mueller, N. D.; O'Connell, C.; Ray, D. K.; West, P. C.; Balzer, C.; Bennett, E. M.; Carpenter, S. R.; Hill, J.; Monfreda, C.; Polasky, S.; Rockström, J.; Sheehan, J.; Siebert, S.; Tilman, D.; Zaks, D. P. M. Solutions for a Cultivated Planet. *Nature* **2011**, *478* (7369), 337–342. https://doi.org/10.1038/nature10452.

(116) Grassini, P.; Eskridge, K. M.; Cassman, K. G. Distinguishing between Yield Advances and Yield Plateaus in Historical Crop Production Trends. *Nat Commun* **2013**, *4* (1), 2918. https://doi.org/10.1038/ncomms3918.

(117) Daioglou, V.; Stehfest, E.; Wicke, B.; Faaij, A.; van Vuuren, D. P. Projections of the Availability and Cost of Residues from Agriculture and Forestry. *GCB Bioenergy* **2016**, *8* (2), 456–470. https://doi.org/10.1111/GCBB.12285.

(118) Chum, H.; Faaij, A.; Moreira, J.; Berndes, G.; Dhamija, P.; Dong, H.; Gabrielle, B.; Eng, A. G.; Lucht, W.; Mapako, M.; Cerutti, O. M.; McIntyre, T.; Minowa, T.; Pingoud, K.; Bain, R.; Chiang, R.; Dawe, D.; Heath, G.; Junginger, M.; Patel, M.; Yang, J.; Warner, E.; Paré, D.; Ribeiro, S. K. Bioenergy. In *Renewable Energy Sources and Climate Change Mitigation: Special Report of the Intergovernmental Panel on Climate Change*; von Stechow, C., Hansen, G., Seyboth, K., Edenhofer, O., Eickemeier, P., Matschoss, P., Pichs-Madruga, R., Schlömer, S., Kadner, S., Zwickel, T., Sokona, Y., Eds.; Cambridge University Press: Cambridge, 2011; pp 209–332. https://doi.org/DOI: 10.1017/CBO9781139151153.006.

(119) Richardson, K.; Steffen, W.; Lucht, W.; Bendtsen, J.; Cornell, S. E.; Donges, J. F.; Drüke, M.; Fetzer, I.; Bala, G.; von Bloh, W.; Feulner, G.; Fiedler, S.; Gerten, D.; Gleeson, T.; Hofmann, M.; Huiskamp, W.; Kummu, M.; Mohan, C.; Nogués-Bravo, D.; Petri, S.; Porkka, M.; Rahmstorf, S.; Schaphoff, S.; Thonicke, K.; Tobian, A.; Virkki, V.; Wang-Erlandsson, L.; Weber, L.; Rockström, J. Earth beyond Six of Nine Planetary Boundaries. *Sci Adv* **2024**, *9* (37), eadh2458. https://doi.org/10.1126/sciadv.adh2458.

(120) Haberl, H.; Erb, K.-H.; Plutzar, C.; Fischer-Kowalski, M.; Krausmann, F. Human Appropriation of Net Primary Production (HANPP) as Indicator for Pressures on Biodiversity. In *Sustainability indicators*; SCOPE, Island Press Washington, DC, Covelo/London, 2007; pp 271–288.

(121) BP p.l.c. Bp Statistical Review of World Energy 2022. **2022**.

(122) Vinichenko, V.; Jewell, J.; Jacobsson, J.; Cherp, A. Historical Diffusion of Nuclear, Wind and Solar Power in Different National Contexts: Implications for Climate Mitigation Pathways. *Environmental Research Letters* **2023**, *18* (9), 094066. https://doi.org/10.1088/1748-9326/acf47a.

(123) White, S. P. J. M. R. *UK nuclear plant hit by new multiyear delay and could cost up to £46bn*. Financial Times.

(124) International Renewable Energy Agency. *Tracking COP28 outcomes: Tripling renewable power capacity by 2030*. https://www.irena.org/Digital-Report/Tracking-COP28-outcomes-Tripling-renewable-power-capacity-by-2030 (accessed 2024-06-07).

(125) Clarke, L.; Wei, Y.-M.; Navarro, A. D. L. V.; Garg, A.; Hahmann, A. N.; Khennas, S.; Azevedo, I. M. L.; Löschel, A.; Singh, A. K.; Steg, L.; Strbac, G.; Wada, K. Energy

Systems. In *IPCC, 2022: Climate Change 2022: Mitigation of Climate Change. Contribution of Working Group III to the Sixth Assessment Report of the Intergovernmental Panel on Climate Change*; Shukla, P. R., Skea, J., Slade, R., Khourdajie, A. Al, R. van Diemen, D. M., Pathak, M., Some, S., P. Vyas, R. F., Belkacemi, M., Hasija, A., Lisboa, G., Luz, S., Malley, J., Eds.; Cambridge University Press: Cambridge, UK and New York, NY, USA, 2022. https://doi.org/10.1017/9781009157926.008.

(126) IEA. *Renewable Energy Market Update: Outlook for 2023 and 2024*; 2023. https://www.iea.org/reports/renewable-energy-market-update-june-2023/will-more-wind-and-solar-pv-capacity-lead-to-more-generation-curtailment.

(127) Xu, R.; Zeng, Z.; Pan, M.; Ziegler, A. D.; Holden, J.; Spracklen, D. V.; Brown, L. E.; He, X.; Chen, D.; Ye, B.; Xu, H.; Jerez, S.; Zheng, C.; Liu, J.; Lin, P.; Yang, Y.; Zou, J.; Wang, D.; Gu, M.; Yang, Z.; Li, D.; Huang, J.; Lakshmi, V.; Wood, Eric. F. A Global-Scale Framework for Hydropower Development Incorporating Strict Environmental Constraints. *Nature Water* **2023**, *1* (1), 113–122. https://doi.org/10.1038/s44221-022-00004-1.

(128) Eker, S.; Rovenskaya, E.; Obersteiner, M.; Langan, S. Practice and Perspectives in the Validation of Resource Management Models. *Nat Commun* **2018**, *9* (1), 5359. https://doi.org/10.1038/s41467-018-07811-9.

(129) International Energy Agency. The Future of Petrochemicals. **2018**.

(130) International Aluminium Institute. *Beyond 2 Degrees: The Outlook for The Aluminium Sector Methodology Report*; 2021. https://international-aluminium.org/resource/beyond-2-degrees-the-outlook-for-the-aluminium-sector-factsheet/ (accessed 2022-09-05).

(131) Global Cement and Concrete Association. *GNR Project*. https://gccassociation.org/gnr/ (accessed 2022-07-30).

(132) Westbroek, C. D.; Bitting, J.; Craglia, M.; Azevedo, J. M. C.; Cullen, J. M. Global Material Flow Analysis of Glass: From Raw Materials to End of Life. *J Ind Ecol* **2021**, *25* (2), 333–343. https://doi.org/10.1111/jiec.13112.

(133) Confederation of European Paper Industries (CEPI). European Pulp & Paper Industry - Key Statictics 2020. **2021**.

(134) World Steel Association. *Steel Statistical Yearbook 2020 Concise Version*; 2020. https://worldsteel.org/wp-content/uploads/Steel-Statistical-Yearbook-2020-concise-version.pdf (accessed 2024-05-03).

(135) Textile Exchange. *Preferred Fiber & Materials: Market Report 2019*; 2019. https://store.textileexchange.org/product/2019-preferred-fiber-materials-report/.

(136) UNEP IRP. *Global Material Flows Database*. https://www.resourcepanel.org/global-material-flows-database (accessed 2022-06-10).

(137) Graver, B.; Zhang, K.; Rutherford, D. *CO2 Emissions from Commercial Aviation, 2018*; 2019. https://theicct.org/publication/co2-emissions-from-commercial-aviation-2018/ (accessed 2021-02-17).

(138) International Road Federation (IRF). IRF World Road Statistics (WRS) Data. 2022. https://worldroadstatistics.org/wrs-data/data/.

(139) The World Bank. World Development Indicators. Data Source: Internation Union of Railways (UIC Railisa Database), OECD Statistics. https://databank.worldbank.org/source/world-development-indicators.

(140) Kaza, S.; Yao, L. C.; Bhada-Tata, P.; Van Woerden, F. *What a Waste 2.0: A Global Snapshot of Solid Waste Management to 2050*; Washington, DC: World Bank, 2018. https://doi.org/10.1596/978-1-4648-1329-0.

(141) Jones, E. R.; van Vliet, M. T. H.; Qadir, M.; Bierkens, M. F. P. Country-Level and Gridded Estimates of Wastewater Production, Collection, Treatment and Reuse. *Earth Syst Sci Data* **2021**, *13* (2), 237–254. https://doi.org/10.5194/essd-13-237-2021.

(142) Seneca Creek Associates LLC. *Illegal Logging and Global Wood Markets: The Competitive Impacts on the US Wood Products Industry*; American Forest \& Paper Association, 2004.

(143) Krausmann, F.; Erb, K.-H.; Gingrich, S.; Haberl, H.; Bondeau, A.; Gaube, V.; Lauk, C.; Plutzar, C.; Searchinger, T. D. Global Human Appropriation of Net Primary Production Doubled in the 20th Century. *Proceedings of the National Academy of Sciences* **2013**, *110* (25), 10324–10329. https://doi.org/10.1073/pnas.1211349110.

(144) U.S. Geological Survey. *Mineral Commodity Summaries*; 2020. https://doi.org/https://doi.org/10.3133/mcs2020.

(145) IEA. Putting CO2 to Use. **2019**, No. September, 86.

(146) IEA. *Renewable Energy Market Update 2021*; Paris, 2021. https://www.iea.org/reports/renewable-energy-market-update-2021.

(147) IEA. *Dataset: World Electricity and Heat Supply and Consumption*. https://ukdataservice.ac.uk/ (accessed 2023-03-17).

(148) Saygin, D.; Gielen, D. Zero-Emission Pathway for the Global Chemical and Petrochemical Sector. *Energies (Basel)* **2021**, *14* (13). https://doi.org/10.3390/en14133772.

(149) IRENA; FAO. *Renewable Energy and Agri-Food Systems: Advancing Energy and Food Security towards Sustainable Development Goals*; 2021. https://doi.org/10.4060/cb7433en.

(150) Levi, P. G.; Cullen, J. M. Mapping Global Flows of Chemicals: From Fossil Fuel Feedstocks to Chemical Products. *Environ Sci Technol* **2018**, *52* (4), 1725–1734. https://doi.org/10.1021/ACS.EST.7B04573/ASSET/IMAGES/LARGE/ES-2017-04573E_0002.JPEG.

(151) Paoli, L.; Lupton, R. C.; Cullen, J. M. Useful Energy Balance for the UK: An Uncertainty Analysis. *Appl Energy* **2018**, *228*, 176–188. https://doi.org/10.1016/j.apenergy.2018.06.063.

(152) Minx, J. C.; Lamb, W. F.; Andrew, R. M.; Canadell, J. G.; Crippa, M.; Döbbeling, N.; Forster, P. M.; Guizzardi, D.; Olivier, J.; Peters, G. P.; others. A Comprehensive and Synthetic Dataset for Global, Regional, and National Greenhouse Gas Emissions by Sector 1970-2018 with an Extension to 2019. *Earth Syst Sci Data* **2021**, *13* (11), 5213–5252.

(153) European Commission JRC (Datasets). EDGAR (Emissions Database for Global Atmospheric Research) Community GHG Database (a Collaboration between the European Commission, Joint Research Centre (JRC), the International Energy Agency (IEA), and Comprising IEA-EDGAR CO2, EDGAR CH4, EDGAR N2O, EDG. **2022**.

(154) Heijungs, R. Sensitivity. In *Probability, Statistics and Life Cycle Assessment: Guidance for Dealing with Uncertainty and Sensitivity*; Springer International Publishing: Cham, 2024; pp 679–785. https://doi.org/10.1007/978-3-031-49317-1_9.

(155) Marchau, V. A. W. J.; Walker, W. E.; Bloemen, P. J. T. M.; Popper, S. W. *Decision Making under Deep Uncertainty: From Theory to Practice*, 1st ed.; Cham: Springer International Publishing AG: Cham, 2019.

(156) Reed, P. M.; Hadjimichael, A.; Malek, K.; Karimi, T.; Vernon, C. R.; Srikrishnan, V.; Gupta, R. S.; Gold, D. F.; Lee, B.; Keller, K.; Thurber, T. B.; Rice, J. S. *Addressing Uncertainty in Multisector Dynamics Research*; Zenodo, 2022. https://doi.org/10.5281/zenodo.6110623.

(157) Saygin, D.; Patel, M. K.; Tam, C.; Gielen, D. J. *IEA Information Paper: Chemical and Petrochemical Sector*; 2009. https://www.iea.org/reports/chemical-and-petrochemical-sector.

(158) Kramer, G. J.; Haigh, M. No Quick Switch to Low-Carbon Energy. *Nature* **2009**, *462* (7273), 568–569. https://doi.org/10.1038/462568a.

(159) IEA. *Net Zero Roadmap: A Global Pathway to Keep the 1.5 °C Goal in Reach*; Paris, 2023. https://www.iea.org/reports/net-zero-roadmap-a-global-pathway-to-keep-the-15-0c-goal-in-reach.

(160) International Atomic Energy Agency (IAEA). *Energy, Electricity and Nuclear Power Estimates for the Period up to 2050*; Reference Data Series; International Atomic Energy Agency, 2024. https://doi.org/10.61092/iaea.e3qb-hsrr.

(161) IRENA. *Geothermal*. https://www.irena.org/Energy-Transition/Technology/Geothermal-energy (accessed 2025-05-21).

(162) Heijungs, R. Guidance for Standard LCA. In *Probability, Statistics and Life Cycle Assessment: Guidance for Dealing with Uncertainty and Sensitivity*; Springer International Publishing: Cham, 2024; pp 1055–1077. https://doi.org/10.1007/978-3-031-49317-1_15.
