# Supplementary material for: Aggregating Demand for Three Fundamental Resources to Avoid Burden-Shifting in Climate Policy
Source: Environ Sci Technol. 2026 Mar 26;60(13):9958–72. doi: 10.1021/acs.est.5c12742 (PMC13063813; doi:10.1021/acs.est.5c12742)
Supplement: Supplementary file 2 [file es5c12742_si_002.pdf]

Supplementary Information (document 2 of 2) for: Aggregating demand for three fundamental resources to avoid burden-shifting in climate policy

Document 1 contains parts 1-4. This document (document 2) contains parts 5-6.

Jennifer L Hawkin and Julian M Allwood

2025

Contents

5 Model Coefficients **S113**

5.1 Structure of SI Part 5 . . . S113
5.2 Sectors and Activities Included and Excluded from the Model . . . S114
5.3 Mining and Extraction . . . S116
5.4 Electricity Generation and Distribution . . . S133
5.5 Agriculture and Forestry . . . S143
5.6 Chemicals, Chemical Products and Fuels . . . S161
5.7 Industry . . . S213
5.8 Transport . . . S248
5.9 Energy-use in Buildings . . . S294
5.10 Waste Management . . . S312
5.11 Negative Emissions Technologies, NETs . . . S319

6 Model Inputs **S324**

6.1 Overview . . . S324
6.2 Summary of final apparent activity rates ($\mathbf{q_{Apparent}}$) for all runs . . . S325
6.3 Summary of delivery process shares (α) for all runs . . . S326
6.4 IEA Net-Zero Energy by 2050 Scenario . . . S330
6.5 2050 Industry Accumulated Demands Example . . . S340
6.6 UK Government Strategy . . . S349
6.7 Low Zero Emissions Resources (Low ZER) Example . . . S356
6.8 Model processes . . . S359

Part 5 and 6 References **S363**

5 Model Coefficients

SI Part 5 outlines the derivation of coefficients of the Extended Process Matrix (as described in the Methods section of the main paper) which represent the inputs and outputs of all delivery processes. Processes have been grouped by activity and sector, with each sector making a separate chapter of the document. The contents below gives the full structure of the document and a simple list of activities and sectors included in the model is given in Section 5.2, Table S20. To make it easier to compare against other datasets and models (such as IEA World Energy Balances), most processes have been allocated to ISIC (International Standard Industrial Classification) codes - this is given in a table at the start of the section for each sector. Processes within the Transport and Building-Use sectors do not have a simple representation in ISIC since these include non-commercial activities, such as heating residential homes and driving personal cars. There are some key processes which have not been included in the model at this stage (Table S21).

Key assumptions used to derive the coeffients in this document are given in the SI Part 2.2.

5.1 Structure of SI Part 5

Each section of Part 5 describes a different sector, starting from the sector context, and key data sources. Within each sector, every activity in turn is described by a flow diagram (Figure S23) and the delivery processes, any key assumptions, sources and/or input data are listed. The derived coefficients for each delivery process are given both at the process subsection level and in a summary table for the sector, given at the end of each sector chapter. In these tables the *Activity* and *Process* describe the recipe to which these coefficients belong, while *Resources* are the flows (inputs or outputs). Positive coefficient values are outputs of the process while negative values are inputs (see the methods section of the main paper).

Each activity is described using a flow diagram similar to the generic example in Figure S23. These diagrams are used to show the flow of materials and energy through the process. An additional flow diagram is given for delivery processes where the flows differ significantly from the activity-level diagram (for example including additional flows or excluding many at the activity level). The example shown in Figure S23 represents a material production process (such as steel production or biofuel production) but two other types of processes also exist in the model and are also represented in the same format. Processes which represent provision of economic activities (e.g. transport by train) or waste management (e.g. carbon dioxide removal) differ only in that the main flow is within the output activities or input materials, respectively, rather than output materials.

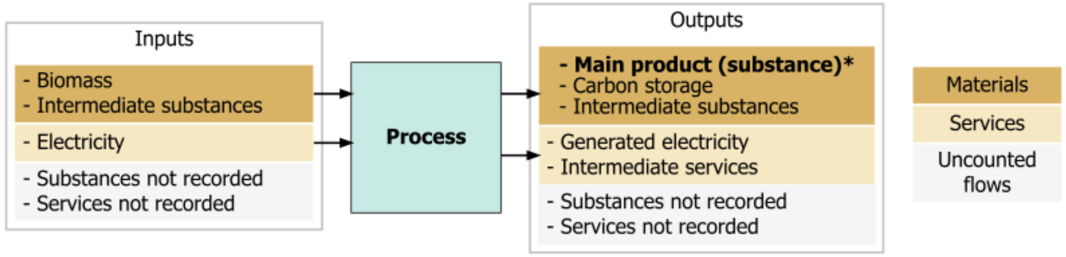

*Figure S23: A generic flow diagram, used to represent all processes in the model. The example shown is a material production process (such as steel production or biofuel production). Each process in the model is characterised as a linear mapping between inputs and outputs. Only flows of materials associated with energy consumption are accounted for (in darker brown); if all material flows were accounted for, each process could be mass-balanced. The intended product or service of each process (the functional flow) is labelled in bold, marked by an *.*

5.2 Sectors and Activities Included and Excluded from the Model

Sector	Activity	Model Name	ISIC Code	End-Use
AgricultureForests	Farming Food	RawFood	14, 15, 17, 3	
AgricultureForests	Forestry	Wood	2	
AgricultureForests	Plant Agriculture	NonWoodBiomass	11, 12, 13, 16	
Buildings	Cooking	Cooking		✓
Buildings	Cooling the Built Environment	Cooling		✓
Buildings	Lighting	Lighting		✓
Buildings	Space Heating	SpaceHeat		✓
Buildings	Use of Appliances	Appliances		✓
Buildings	Water Heating	WaterHeating		✓
Electricity	Distribution of Electricity	Electricity	351	
Electricity	Electricity Generation	GeneratedElectricity	351	
FuelsFeedstocks	Ammonia Production	Ammonia	2012	
FuelsFeedstocks	Biofuel Production	BioFuel	383, 382	
FuelsFeedstocks	HVCs Production	HVCs	2013	
FuelsFeedstocks	Hydrogen Production	Hydrogen	2011	
FuelsFeedstocks	Methane Production	Methane	352	
FuelsFeedstocks	Methanol Production	Methanol	2011	
FuelsFeedstocks	Oil Processing and Refining	Oil	192	
FuelsFeedstocks	Plastics Production - end-use quantity excludes plastics for fabrics	Plastics	2013	✓
FuelsFeedstocks	Production of other petrochemical products, not accounted for elsewhere	OtherPetChem	202, 203	✓
FuelsFeedstocks	Synfuel Production	Synfuel	383, 382	
FuelsFeedstocks	Urea Production	Urea	2012	
Industry	Aluminium Production	Aluminium	242	✓
Industry	Cement Production	Cement	2394, 2395	✓
Industry	Construction	Construction	41, 42, 43	✓
Industry	Food Processing	Food	10, 11, 12	✓
Industry	Glass Production	Glass	231, 2310	✓
Industry	Paper Production	Paper	17, 18	✓
Industry	Product Manufacturing and other Industrial Processes	OtherIndustry	16, 22, 25, 26, 27, 28, 29, 30, 31, 32, 8422	✓
Industry	Steel Production	Steel	191, 241	✓
Industry	Textiles Production	Textiles	13, 14, 15	✓
Mining	Coal Mining	Coal	5	
Mining	Minerals and Metals Mining	MinedMetalsMinerals	7, 8, 99	✓
Mining	Oil and Gas Extraction	ExtractedOilGas	6, 91	
NETs	Management of residual emissions (negative emissions tech.)	NetEmissions		
NETs	Management of carbon dioxide gas	CO2Product	2011	
Transport	Aviation	Aviation		✓
Transport	Bus Transportation	BusUse		✓
Transport	Car Transportation	CarUse		✓
Transport	Passenger Rail Transportation	RailP		✓
Transport	Rail Freight	RailFreight		✓
Transport	Road Freight	RoadFreight		✓
Transport	Shipping	Shipping		✓
Waste	Management of Municipal Solid Waste	WasteSolid	382	✓
Waste	Wastewater treatment	Wastewater	36, 37	✓

Table S20: Sectors and activities included in the model. The demand for the end-use Activities is specified by the user model inputs (the net-zero proposals); other Activities are treated as purely intermediary in the current analysis. ISIC codes listed here are used as an indication but may not corespond exactly as a one-one translation.

ISIC Code	Description (ISIC Classification)	ISIC flow excluded from model
2011	Manufacture of basic chemicals	All other basic chemicals (aside carbon dioxide, hydrogen and methanol)
2012	Manufacture of fertilizers and nitrogen compounds	Non-urea fertilizer and other nitrogen compounds
21	Manufacture of basic pharmaceutical products and pharmaceutical preparations	Pharmaceuticals, medicinal chemical and botanical products
2391	Manufacture of refractory products	Refractory motars/cements, and ceramic products
2392	Manufacture of clay building materials	Clay building materials
2393	Manufacture of other porcelain and ceramic products	Other porcelains and ceramics
2394	Manufacture of cement, lime and plaster	Lime and plaster
2395	Manufacture of articles of concrete, cement and plaster	Concrete making process excluded
2396	Cutting, shaping and finishing of stone	Stone products
2399	Manufacture of other non-metallic mineral products n.e.c.	Other non-metallic minerals (aside glass and cement)
242	Manufacture of basic precious and other non-ferrous metals	Other precious and non-ferrous metals (aside aluminium)
353	Steam and air conditioning supply	Steam and air conditioning supply (although air conditioning is considered within Cooking, Cooling, Lighting, SpaceHeat, WaterHeating, Appliances sector)
381	Waste collection	Waste collection
383	Materials recovery	Secondary raw materials recovery and processing (although production from scrap accounted in the Industry sector)
4921	Urban and suburban passenger land transport	Tramway, streetcar, trolley bus, underground and elevated railways etc
493	Transport via pipeline	Pipeline transport
51	Air transport	Freight air transport

Table S21: Key ISIC divisions, groups or classes not accounted for in the model. Additional economic activities (such as ‘Financial and insurance activities’) are also not explicitly accounted for and not listed here since that disagreggation is not relevant for the model

5.3 Mining and Extraction

This section outlines the derivation of coefficients for extracting oil & gas, mining coal, and mining metal ores & industrial minerals. This sector covers both the exploration and extraction of raw materials, and is grouped into three activities (shown in Table S22). Extracted oil & gas, and minerals & metal ores are each treated as single combined substances to be consistent with data sources for global oil and gas extraction (Section 5.3.2). Although there is significant variation between the extraction processes, emissions, and scale of different commodities within these activities, the granularity is limited by aggregation in data.

Downstream processing of these materials is not included in this sector. For oil & gas, downstream processes are accounted for in Section 5.6. For metal ores and industrial minerals, downstream processes are accounted for in the Industry Sector (Section 5.7).

Sector	Activity	Model Name	ISIC Code	End-Use
Mining	Coal Mining	Coal	5	
Mining	Minerals and Metals Mining	MinedMetalsMinerals	7, 8, 99	✓
Mining	Oil and Gas Extraction	ExtractedOilGas	6, 91	

Table S22: Model flows categorised by ISIC divisions, groups and classes for this Sector. For activities labelled as 'End-Use', the activity rate is set by the model inputs, rather than the demands of other activities.

5.3.1 Overarching Sector Assumptions

Within each activity, coefficients are approximated based on fuel use for each sector in 2018, using the following assumptions:

- All materials require the same energy for extraction, processing, and transportation.
- There is no significant change from current energy intensity of extraction; in reality energy intensity may rise (especially for metals) given decreasing grades of metal ores (Azadi et al., 2020) and increasing scarcity of resources.
- The impact of mitigation choices for end-uses on manufacturing demands for materials are not considered (assumed instead that these have been exogenously considered and that input values are self-consistent).
- Fugitive emissions are insignificant for metal ores and industrial minerals. For fossil fuels, fugitive emissions are estimated as a proportion of methane leakage per tonne of fuel extracted. This is likely to be a lower bound of estimated emissions since:
  - There is significant uncertainty in estimation of fugitive emissions and sources are more likely to be omitted than double-counted;
  - Fugitive emissions may remain significant even after coal mines have been abandoned, according to Kholod et al. (2020); and
  - Global Energy Monitor's bottom-up database indicates that proposed future coal mines could be gassier than existing ones (2021).

The resource demands for each delivery process are calculated by considering the relative efficiency and emissions of providing power by alternative fuels. In the absence of a detailed understanding of the processes involved, each process of energy provision have been assumed to operate at the same level of efficiency.

5.3.2 Sector-Wide Data Sources

The coefficients are derived from the energy and emissions intensity of emissions extraction in 2018. These intensity values are calculated from the following data sources:

- Material volumes extracted from the United Nations Environment Programme International Resource Panel Global Material Flows Database (UNEP IRP, 2021)
- Energy consumption from IEA Extended Energy Balances (International Energy Agency, 2021a)
- Emissions factors for combustion of fuels, as described in the SI Part 2.

Although the energy consumption values given by International Energy Agency (2021a) provide a first-pass estimate for the resource demands in this sector, this approach means that there are some additional processes which are not currently accounted for in the model.

- For mining metals and minerals, the category used in this analysis (*Mining and quarrying*) does not include energy for processing minerals (except crushing, grinding, cutting, cleaning, drying, sorting and mixing). Since metal production processes in the Industry Sector (Section 5.7) also do not account for these processes there will be energy consumption and residual emissions which are unaccounted for in the model.
- For oil and gas, only the energy which is used for oil and gas extraction is included, flared gas is not.

5.3.2.1 2018 Production Rates Activity demand for mining and extraction are accounted for by the mass extracted in Gt. Mined metals are accounted for on a run-of-mine basis, ie the amount of material before pre-concentration or processing, excluding overburden. 2018 demand estimates are used to derive the delivery process coefficients; these are taken from Global Material Flows Database (UNEP IRP, 2021) and are shown in Table S23.

Coal, oil and gas are treated as intermediate products in the analysis; in contrast metal ores and raw minerals are considered a final end-use product since there is no linkage of these materials with other processes in the model.

Material Group	2018 Extraction, Gt
Coal	7.6
Other Fossil Fuels	8.0
Metals	9.4
Industrial Minerals	44

Table S23: 2018 activity demand for mining and extraction processes. Data are taken from (UNEP IRP, 2021), where metals are counted on a run-of-mine-ore basis.

5.3.3 Coal Mining

This activity describes the exploration and mining associated with extracting coal products. The coefficients for coal mining are derived from the global energy use in 2018 based on the category, *Energy industry own use - Coal mines*, in IEA energy balances (International Energy Agency, 2021a). This category represents "energy which is used directly within the coal industry for hard coal and lignite mining" (International Energy Agency - IEA, 2020), including benefaction processes (such as crushing, screening, washing, and flotation) but excluding energy used for processing coal into coke or briquettes. The pre-processing of coal products is accounted for in the processes for steel production (Section 5.7.3) but is not explicitly accounted for elsewhere.

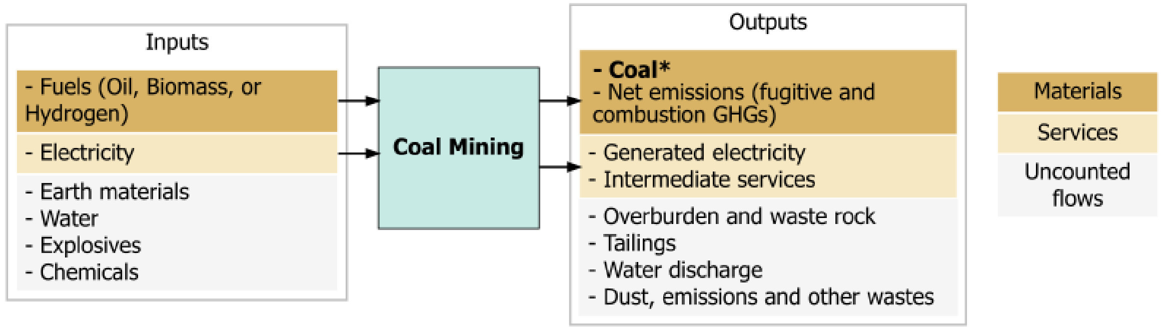

Figure S24: Inputs and outputs for extracting coal products.

Each process (as listed in Table S24) assumes a fuel switch and/or use of abatement technology to eliminate fugitive emissions by capturing methane for electricity generation. The energy demands and emissions calculated for each delivery process are normalised by the quantity of coal mined in 2018, as given in Table S25. Fugitive emissions (where included) are estimated using the assumptions in Table S25.

Fugitive emissions in coal mining are taken to be the emissions of coal mine methane (CMM) from active mines. CMM is gas trapped in coal seams that is released during mining (International Energy Agency, 2019b), and which may continue to escape after the mine has closed (Kholod et al., 2020). CMM may also be produced in post-mining coal handling, processing, and transportation (Irving & Tailakov, 2000). The value used in this analysis is a mid-range value from literature, given in Table S25. This is a conservative estimate since more recent, and more detailed studies, consistently predict the higher values, and given old mines may continue to release methane, the quantity will increase as more mines are created. Furthermore, Kholod et al. (2020) argue that estimates of coal mine methane have been widely under-reported, demonstrating analysis showing 2010 emissions from active mines to be ~80 Mt (and 100 Mt if inactive mines are considered), at least twice the estimate of the International Energy Agency (2019b). The value used for this analysis (shown in Table S25) is within the range consistent with IPCC Tier 1 default emissions factors for methane from coal mining and handling: factors vary, depending on the mine category, up to around 0.42 t CO2e/t coal (with underground mining varying between 0.17 and 0.42 t CO2e/t coal).

For modelling convenience, it has been assumed that processes with emissions abatement technology eliminate 100% fugitive emissions although this would be impossible. Realistic fugitive emissions capture potential is closer to 50% according to the IEA (IEA, 2023c), and can be adjusted using the delivery process shares. Maximum abatement is limited because some sources, such as ventilation air and post-mining handling, processing, and transportation, are disperse or have very low methane concentrations of around 0.1-1% (Irving & Tailakov, 2000).

Activity	Delivery process	Detail
Coal	NET	Conventional Methods
Coal	Elec	Electrical Processes replace Fossil Fuels
Coal	H2	Hydrogen replaces Fossil Fuels
Coal	NETNoFugitive	Conventional Methods with elimination of fugitive emissions
Coal	ElecNoFugitive	Electrical Processes replace Fossil Fuels with elimination of fugitive emissions
Coal	H2NoFugitive	Hydrogen replaces Fossil Fuels with elimination of fugitive emissions

Table S24: Delivery processes for coal mining.

Property	Values	Justification
2018 Global Extraction, Gt (run-of-mine ore basis)	7.6	The United Nations Environment Programme International Resource Panel Global Material Flows Database
2018 Coal Mine Methane (Gt Methane)	0.060	Mid-range of literature estimates (varying between 0.04 and 0.08 for active mines); IEA (2019), Kholod (2020)
Methane GWP (Gt CO2e/Gt)	25	100 year Global Warming Potential as used in Bogner et al. 2007
Fugitive emissions (Gt CO2e /Gt Coal)	0.20	Coal Mine Methane / Quantity Extracted x Methane GWP

Table S25: Data used to derive coefficients for coal mining delivery processes. References: (Bogner et al., 2007; International Energy Agency, 2019b; Kholod et al., 2020; UNEP IRP, 2021)

5.3.3.1 Conventional coal mining processes

This delivery process describes coal mining using current approaches (Figure S24). The coefficients are derived from the global energy use in 2018 as shown in Table S26, normalised by the quantity of coal mined in 2018, as given in Table S25, less coal used in mining. Fugitive emissions are estimated using the assumptions in Table S25 and added to the combustion emissions in Table S26. The resulting coefficients are given in Table S27.

Parameter	Coal	Oil	Electricity	Total	Justification
**Summary of key data inputs and assumptions**					
Energy use, 2018; EJ/yr	0.7	0.4	0.5	**1.6**	Global totals from IEA energy balances
Energy density; GJ/t	28	42	N/A		See Model Assumptions Section
Fuel mass flow, 2018; Gt/yr	0.027	0.009	N/A		(energy use) / (energy density)
Emissions factor; Mt/EJ	100	70	0	**0**	See Model Assumptions Section
**Calculated coefficients (inputs)**					
Fuel demand coeff (Gt/Gt or EJ/Gt)		0.0012	0.066	**0.21**	(fuel flow or energy)/(2018 coal extracted - coal used)
**Calculated coefficients (outputs)**					
Combustion emissions (Gt CO2e/Gt)	0.010	0.003	0	**0.013**	(emissions factor) x (energy used) / (2018 mass extracted)
Fugitive emissions (Gt CO2e/Gt)				**0.20**	Mid range of estimates

Table S26: Assumptions used to derive coefficients for coal mining using conventional methods. IEA energy balances (*Energy industry own use - Oil and gas extraction*) are used to estimate the fuel demands and combustion emissions, using the model assumptions for energy densities and emissions factors as described in the SI Part 2. Mass of coal extracted in 2018 and the sources for fugitive emissions are given in Table S25.

Activity	Process	Resource	Inputs	Outputs	Unit
Coal	NET	Electricity	**-0.066**		EJ/Gt_Coal
Coal	NET	NetEmissions		**0.21**	Gt_CO2e/Gt_Coal
Coal	NET	Oil	**-0.0012**		Gt/Gt_Coal
Coal	NET	Coal		**1.0**	Gt_Coal/Gt_Coal

Table S27: Coefficients for coal mining using conventional methods.

Table S27 is the first example of the standard coefficient table which will be used for each process throughout this document. The process inputs are shown in the teal column on the left and process outputs in the red column on the right. Consistently with the model maths (as described in the Maths SI document (Part 2.1)), process inputs are negative values while outputs are positive. The values in Table S27, are derived in Table S26 - a similar format will be used for most other processes.

5.3.3.2 Electrification of coal mining

This delivery process describes coal extraction (Figure S24), where all processes are fully electrified. Although this is an unlikely extreme, it can be used to explore opportunities for some electrification. The coefficients are derived from the global energy use in 2018 as shown in Table S28 normalised by the quantity of coal mined in 2018, as given in Table S25. Fugitive emissions are estimated using the assumptions in Table S25 and added to the combustion emissions in Table S28. The resulting coefficients are given in Table S29.

Parameter	Coal	Oil	Electricity	Total	Justification
**Summary of key data inputs and assumptions**					
Energy use, 2018; EJ/yr	0.7	0.4	0.5	**1.6**	Global totals from IEA energy balances
Assumed relative efficiency	1.0	1.0	1.0		In the absence of a detailed understanding of the processes involved, all fuels are assumed to have the same efficiency level
Assumed energy for electrified mining, EJ/yr			1.6	**1.6**	All energy demand met by electricity
**Calculated coefficients (inputs)**					
Demand coefficient (EJ/Gt)			0.21		(total energy demand) / (2018 mass extracted)
**Calculated coefficients (outputs)**					
Fugitive emissions (Gt CO2e/Gt)				**0.20**	Mid range of estimates

Table S28: Assumptions used to derive coefficients for coal mining with electrified processes. IEA energy balances (*Energy industry own use - Oil and gas extraction*) are used to estimate the fuel demands and combustion emissions, using the model assumptions for energy densities and emissions factors as described in the SI Part 2. Mass of coal extracted in 2018 and the sources for fugitive emissions are given in Table S25.

Activity	Process	Resource	Inputs	Outputs	Unit
Coal	Elec	Electricity	**-0.21**		EJ/Gt_Coal
Coal	Elec	NetEmissions		**0.20**	Gt_CO2e/Gt_Coal
Coal	Elec	Coal		**1.0**	Gt_Coal/Gt_Coal

Table S29: Coefficients for coal mining with electrified processes.

5.3.3.3 Hydrogen-powered coal mining

This delivery process describes coal extraction (Figure S24), where all processes that are conventionally powered by fossil fuels are instead powered by hydrogen. The coefficients are derived from the global energy use in 2018 as shown in Table S30 normalised by the quantity of coal mined in 2018, as given in Table S25. Fugitive emissions are estimated using the assumptions in Table S25 and added to the combustion emissions in Table S30. The resulting coefficients are given in Table S31.

Parameter	Coal	Oil	Electricity	Hydrogen	Total	Justification
**Summary of key data inputs and assumptions**						
Energy use, 2018; EJ/yr	0.7	0.4	0.5		**1.6**	Global totals from IEA energy balances
Assumed relative efficiency	1.0	1.0	N/A	1.0		In the absence of a detailed understanding of the processes involved, all fuels are assumed to have the same efficiency level
Assumed energy for H2-powered mining, EJ/yr			0.5	1.1	**1.6**	2018 fossil-fuel demand is met by hydrogen
Energy density; GJ/t	28	42	N/A	120.0		See Model Assumptions Section
**Calculated coefficients (inputs)**						
Fuel demand coeff (Gt/Gt or EJ/Gt)			0.066	0.0012		(energy use) / (energy density) / (2018 mass extracted)
**Calculated coefficients (outputs)**						
Fugitive emissions (Gt CO2e/Gt)					**0.20**	Mid range of estimates

Table S30: Assumptions used to derive coefficients for coal mining with hydrogen-powered processes. IEA energy balances (*Energy industry own use - Oil and gas extraction*) are used to estimate the fuel demands and combustion emissions, using the model assumptions for energy densities and emissions factors as described in the SI Part 2. Mass of coal extracted in 2018 and the sources for fugitive emissions are given in Table S25.

Activity	Process	Resource	Inputs	Outputs	Unit
Coal	H2	Electricity	**-0.066**		EJ/Gt_Coal
Coal	H2	NetEmissions		**0.20**	Gt_CO2e/Gt_Coal
Coal	H2	Hydrogen	**-0.0012**		Gt/Gt_Coal
Coal	H2	Coal		**1.0**	Gt_Coal/Gt_Coal

Table S31: Coefficients for coal mining using hydrogen-powered processes.

5.3.3.4 Conventional mining processes with elimination of fugitive emissions

This delivery process describes mining coal using fossil-fuelled machinery using coal mine methane abatement technologies to capture methane for electricity generation (Figure S24). The coefficients are derived from the process without abatement technologies (Table S32) but where the electricity demand is offset by the quantity generated using captured coal mine methane, CMM (Table S33). The resulting coefficients are given in Table S34.

For modelling convenience, it has been assumed that 100% fugitive emissions are captured although this would be impossible in reality. Realistic capture potential is closer to 50% according to the IEA (IEA, 2023c), and can be adjusted using the delivery process shares. Maximum abatement is limited because some sources, such as ventilation air and post-mining handling, processing, and transportation, are disperse or have very low methane concentrations of around 0.1-1% (Irving & Tailakov, 2000).

Parameter	Coal	Oil	Electricity	Total	Justification
**Summary of values for the process without CMM abatement**					
Fuel demand coeff (Gt/Gt or EJ/Gt)		0.0012	0.066	**0.21**	(fuel flow or energy)/(2018 coal extracted - coal used)
Combustion emissions (Gt CO2e/Gt)	0.010	0.003	0	**0.013**	(emissions factor) x (energy used) / (2018 mass extracted)

Table S32: Calculated values for the equivalent process without coal mine methane (CMM) abatement technologies. Details are given in Table S26.

Property	Values	Justification
**Summary of key data inputs and assumptions for CMM abatement**		
Fugitive emissions from mining (Gt CO2e /Gt Coal)	0.0079	See caption.
Energy density of methane (EJ/Gt)	45	See document assumptions.
Captured methane from coal mining and handling	100%	For modelling convenience. Realistic capture potential is closer to 50% but can be adjusted using the delivery process shares.
Assumed use of methane for electricity generation	100%	Lower quality and compression requirements than pipeline methane.
Efficiency of electricity generation	20%	Due to sizing, reciprocating or internal combustion (IC) engines most likely (Karacan et al., 2011). Efficiency estimate based on values used by Paoli et al. (2018).
Proportion of net generation (remainder required for gas separation or compression etc. for abatement itself)	98%	Power requirements assumed to be small, but would increase with increasing proportion of abated methane (as concentration decreases)
**Calculated values (outputs)**		
Electricity generated (EJ/Gt)	0.070	Product of data/assumptions listed above

Table S33: Data and assumptions to calculate energy generated by coal mine methane (CMM) abatement technologies. Fugitive emissions data is given in Table S25, and energy density in the SI Part 2. Other references: (Karacan et al., 2011; Paoli et al., 2018)

Activity	Process	Resource	Inputs	Outputs	Unit
Coal	NETNoFugitive	Electricity		**0.0037**	EJ/Gt_Coal
Coal	NETNoFugitive	NetEmissions		**0.013**	Gt_CO2e/Gt_Coal
Coal	NETNoFugitive	Oil	**-0.0012**		Gt/Gt_Coal
Coal	NETNoFugitive	Coal		**1.0**	Gt_Coal/Gt_Coal

Table S34: Coefficients for coal mining using fossil-fuel powered technology and coal mine methane abatement technology to capture and generate electricity.

5.3.3.5 Electrification of coal mining with elimination of fugitive emissions

This delivery process describes mining coal using electrically powered machinery using coal mine methane abatement technologies to capture methane for electricity generation (Figure S24), following the same approach as in Section 5.3.3.4. The coefficients are derived from the process without abatement technologies (Table S35) but where the electricity demand is offset by the quantity generated using captured coal mine methane, CMM (Table S36).

Parameter	Electricity	Justification
**Electricity input required for the process without CMM abatement**		
Demand coefficient (EJ/Gt)	0.21	(total energy demand) / (2018 mass extracted)

Table S35: Calculated values for the equivalent process without coal mine methane (CMM) abatement technologies. Details are given in Table S28.

Property	Values	Justification
**Electricity generated from captured coal mine methane**		
Electricity generated (EJ/Gt)	0.070	Product of data/assumptions listed above

Table S36: Electricity generated from captured coal mine methane. See details in Table S33.

Activity	Process	Resource	Inputs	Outputs	Unit
Coal	ElecNoFugitive	Electricity	-0.14		EJ/Gt_Coal
Coal	ElecNoFugitive	Coal		1.0	Gt_Coal/Gt_Coal

Table S37: Coefficients for coal mining with electrified processes and elimination of fugitive emissions.

5.3.3.6 Hydrogen-powered coal mining with elimination of fugitive emissions

This delivery process describes mining coal using hydrogen powered machinery using coal mine methane abatement technologies to capture methane for electricity generation (Figure S24), following the same approach as in Section 5.3.3.4. The coefficients are derived from the process without abatement technologies (Table S38) but where the electricity demand is offset by the quantity generated using captured coal mine methane, CMM (Table S36).

Parameter	Hydrogen	Electricity	Justification
**Summary of values for the process without CMM abatement**			
Fuel demand coeff (Gt/Gt or EJ/Gt)	0.0012	0.066	(energy use) / (energy density) / (2018 mass extracted)

Table S38: Calculated values for the equivalent process without coal mine methane (CMM) abatement technologies. Details are given in Table S30.

Property	Values	Justification
**Electricity generated from captured coal mine methane**		
Electricity generated (EJ/Gt)	0.070	Product of data/assumptions listed above

Table S39: Electricity generated from captured coal mine methane. See details in Table S33.

Activity	Process	Resource	Inputs	Outputs	Unit
Coal	H2NoFugitive	Electricity		0.0039	EJ/Gt_Coal
Coal	H2NoFugitive	Hydrogen	-0.0012		Gt/Gt_Coal
Coal	H2NoFugitive	Coal		1.0	Gt_Coal/Gt_Coal

Table S40: Coefficients for hydrogen-powered coal mining with elimination of fugitive emissions.

5.3.4 Extraction of Oil & Gas

This activity describes exploration and extraction of oil and gas from fossil fuel reserves. Extracted oil and gas is currently treated as one combined substance, consistent with data sources for global oil and gas extraction (Section 5.3.2). Oil and natural gas are often found in and extracted from the same site. The substances could be treated separately in future, as described in SI Part 2.3.

The delivery processes are derived from the global energy use in 2018 taken from *Energy industry own use - Oil and gas extraction* in IEA energy balances (International Energy Agency, 2021a). Oil and gas extraction produces emissions by the accidental or deliberate release of hydrocarbons (fugitive and vented emissions), by burning hydrocarbon gases released during oil extraction (flaring), and by burning fossil fuels for energy. Fugitive, venting and flaring emissions cannot be eliminated (International Association of Oil & Gas Producers, 2000) but can be reduced by approaches such as: capturing gas rather than flaring and venting, improvements to equipment, and leak detection and repair systems (McKinsey & Company, 2020).

Each delivery process in the model assumes a fuel switch and/or full avoidance of fugitive emissions. For modelling convenience, it has been assumed that processes venting, flaring and leakage abatement eliminate 100% fugitive emissions although this would be impossible. Realistic reductions in these emissions can be adjusted using the delivery process shares. The energy demands and emissions calculated for each delivery process are normalised by the quantity of oil & gas extracted in 2018, as given in Table S41. Vented and fugitive emissions (where included) are estimated using the assumptions in Table S41.

Around 30 Mt carbon dioxide is used today for Enhanced Oil Recovery (based on data provided by Global CSS Institute (GCCSI) (2022)). In EOR carbon dioxide gas is injected into oil seam to reduce the viscosity of oil and increase the fraction of oil which can be recovered from a given oil field (Smit, 2014). Although some carbon dioxide is brought to the surface with the extracted oil, it is mostly recycled and reused to minimise costs of oil extraction. According to Hill et al. (2013) venting and fugitive emissions in EOR “amount to a few percent or less of the purchased volumes of CO2”. This means that most of the carbon dioxide injected remains underground and so should be accounted as carbon storage. The coefficients for carbon dioxide gas and stored carbon are estimated by normalising carbon dioxide used today for EOR by the quantity of oil extracted in 2018 (Table S41).

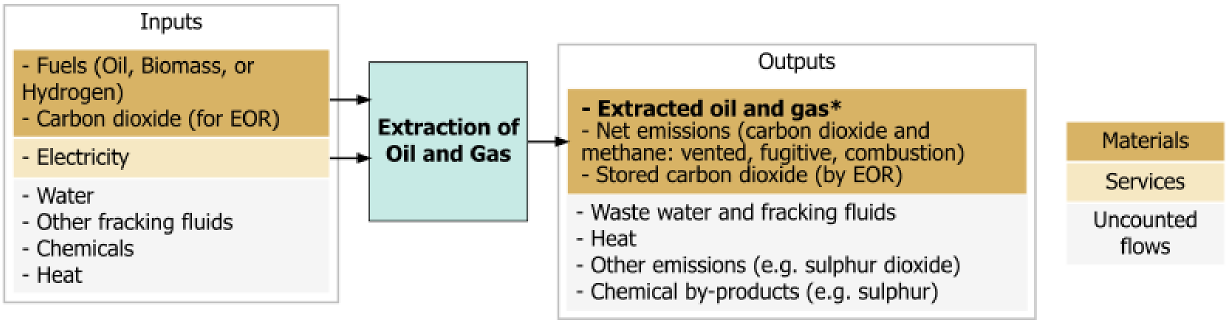

Figure S25: Inputs and outputs for extracting oil and gas. There are many diverse processes involved in oil and gas extraction which depend on geological conditions, amongst many other factors. The general flows given here are based on the basic process descriptions given by (Meili et al., 2022): in primary oil extraction, the storage site pressure is sufficient to extract oil without additives; if the field pressure is not enough to transport oil to the bottom of the borehole, fluids (water and/or gases), heat or chemicals may be used to reduce the oil viscosity and drive the oil to the borehole - these are termed secondary or tertiary oil extraction; in gas extraction, chemicals may be required to remove water, water vapour and sulphur. This is termed Enhanced Oil Recovery (EOR) - carbon dioxide for EOR and subsequent storage is accounted for in this process.

Activity	Delivery process	Detail
ExtractedOilGas	NET	Conventional Methods
ExtractedOilGas	Elec	Electrically Powered
ExtractedOilGas	NETNoFugitive	Conventional Methods with elimination of fugitive emissions
ExtractedOilGas	ElecNoFugitive	Electrical Processes replace Fossil Fuels with elimination of fugitive emissions

Table S42: Delivery processes for extracting oil and gas.

Property	Values	Justification
**Summary of key data inputs and assumptions**		
2018 Global Extraction, Gt	8.0	The United Nations Environment Programme International Resource Panel Global Material Flows Database
Fugitive and vented methane (Gt/Gt)	0.013	Based on the estimate of Howarth (2014); estimates vary from ~0.4 - 9%
Methane GWP (Gt CO2e/Gt)	25	100 year Global Warming Potential as used in Bogner et al. 2007
Flaring emissions (Gt CO2e)	0.5	IEA (2023), Tracking Clean Energy Progress 2023
Carbon dioxide used for EOR (Mt/year)	30	Data provided bu the global CCS Institute (2022)
**Calculated values**		
Non-energy related emissions (Gt CO2e /Gt)	0.39	(Methane Leakage x Methane GWP) + (Flaring emissions / 2018 extraction)
Carbon dioxide used in extraction (Gt/Gt)	0.0038	CO2 used/Global extraction

Table S41: Data used to derive coefficients for oil & gas extraction delivery processes. References: (Bogner et al., 2007; Howarth, 2014; UNEP IRP, 2021)

5.3.4.1 Conventional extraction processes

This delivery process describes extracting fossil oil and gas using current approaches (Figure S25). The coefficients are derived from the global energy use in 2018 as shown in Table S43, normalised by the quantity extracted in 2018, as given in Table S41. Venting, flaring and fugitive emissions are estimated based on the data and assumptions in Table S41 and added to the combustion emissions in Table S43.

Parameter	Natural gas	Oil products	Electricity	Heat	Total	Justification
**Summary of key data inputs and assumptions**						
Energy use, 2018 (EJ)	-7.7	-1.3	-1.0	-0.2	**-10.2**	From IEA energy balances
Emissions factor (kg CO2/GJ)	60	70	0	0	**N/A**	See Model Assumptions Section
Energy density (GJ/t)	45	42	N/A	N/A	**N/A**	See Model Assumptions Section
**Calculated coefficients (inputs)**						
Fuel demand coeff (Gt/Gt)	-0.021	-0.004	N/A	N/A	**N/A**	(energy use in 2018) / (extracted mass in 2018)
Fuel demand coeff (EJ/Gt)	N/A	N/A	-0.13	0	**N/A**	(energy use in 2018) / (energy density) / (extracted mass in 2018) ; Heat is not accounted for
**Calculated coefficients (outputs)**						
Combustion emissions (Gt CO2e/Gt)	0.057	0.011	N/A	N/A	**0.068**	(emissions factor) x (energy used) / (2018 mass extracted)
Fugitive, vented & flaring emissions (Gt CO2e/Gt)	N/A	N/A	N/A	N/A	**0.39**	Mid range of estimates

Table S43: Assumptions used to derive coefficients for extracting oil and gas using conventional methods. IEA energy balances (*Energy industry own use - Oil and gas extraction*) are used to estimate the fuel demands and combustion emissions, using the model assumptions for energy densities and emissions factors as described in the SI Part 2. Mass of oil and gas extracted in 2018 and the sources for fugitive emissions are given in Table S41. Heat is assumed to be available from other sources as waste heat and so not accounted for here. Negative values are inputs to the process, positive are outputs.

Activity	Process	Resource	Inputs	Outputs	Unit
ExtractedOilGas	NET	CO2Storage		**0.0038**	Gt_CO2/Gt
ExtractedOilGas	NET	CO2Product	**-0.0038**		Gt_CO2/Gt
ExtractedOilGas	NET	Electricity	**-0.13**		EJ/Gt
ExtractedOilGas	NET	Electricity	**-0.021**		Gt/Gt
ExtractedOilGas	NET	NetEmissions		**0.46**	Gt_CO2e/Gt
ExtractedOilGas	NET	Oil	**-0.0037**		Gt/Gt
ExtractedOilGas	NET	ExtractedOilGas		**1.0**	Gt/Gt

Table S44: Coefficients for extracting oil and gas using conventional methods. Coefficients for carbon dioxide and carbon dioxide storage assume that the current rate of Enhanced Oil Recovery with carbon dioxide continues (page S124).

5.3.4.2 Electrification of oil and gas extraction

This delivery process describes extracting fossil oil and gas using electrified processes (Figure S25). The coefficients are derived from the global energy use in 2018 as shown in Table S43, normalised by the quantity extracted in 2018, as given in Table S41. Venting, flaring and fugitive emissions are estimated based on the data and assumptions in Table S41 and added to the combustion emissions in Table S43.

Figure S26: Process schematic for extraction of oil and gas (fossil fuels) using electrically powered processes.

Parameter	Natural gas	Oil products	Electricity	Heat	Total	Justification
**Summary of key data inputs and assumptions**						
Energy use, 2018 (EJ)	-7.7	-1.3	-1.0	-0.2	**-10.2**	From IEA energy balances
Assumed relative energy consumption (electrification)	100%	100%	100%	0%	**N/A**	Heat assumed to be available from other sources as waste heat
**Calculated coefficients (inputs)**						
Fuel demand coeff (EJ/Gt)	N/A	N/A	-1.2	N/A	**N/A**	(energy use in 2018) x (relative energy demand) / (extracted mass in 2018)
**Calculated coefficients (outputs)**						
Fugitive, vented & flaring emissions (Gt CO2e/Gt)	N/A	N/A	N/A	N/A	**0.39**	Mid range of estimates

Table S45: Assumptions used to derive coefficients for extracting oil and gas with electrified processes. IEA energy balances (*Energy industry own use - Oil and gas extraction*) are used to estimate the fuel demands and combustion emissions. Mass of oil and gas extracted in 2018 and the sources for fugitive emissions are given in Table S41. Negative values are inputs to the process, positive are outputs.

Activity	Process	Resource	Inputs	Outputs	Unit
ExtractedOilGas	Elec	CO2Storage		**0.0038**	Gt_CO2/Gt
ExtractedOilGas	Elec	CO2Product	**-0.0038**		Gt_CO2/Gt
ExtractedOilGas	Elec	Electricity	**-1.2**		EJ/Gt
ExtractedOilGas	Elec	NetEmissions		**0.39**	Gt_CO2e/Gt
ExtractedOilGas	Elec	ExtractedOilGas		**1.0**	Gt/Gt

Table S46: Coefficients for extracting oil and gas using conventional methods. Coefficients for carbon dioxide and carbon dioxide storage assume that the current rate of Enhanced Oil Recovery with carbon dioxide continues (page S124).

5.3.4.3 Conventional extraction processes with elimination of fugitive emissions

This delivery process describes extracting fossil oil and gas using current approaches (Figure S25) using methods to eliminate venting, flaring and fugitive emissions. For modelling convenience, it has been assumed that processes venting, flaring and leakage abatement eliminate 100% fugitive emissions although this would be impossible (International Association of Oil & Gas Producers, 2000); realistic reductions in these emissions can be adjusted using the delivery process shares. Fugitive, venting and flaring emissions can be reduced by approaches such as: capturing gas rather than flaring and venting, improvements to equipment, and leak detection and repair systems (McKinsey & Company, 2020).

The coefficients are derived from the global energy use in 2018 as shown in Table S43, normalised by the quantity extracted in 2018, as given in Table S41.

Activity	Process	Resource	Inputs	Outputs	Unit
ExtractedOilGas	NETNoFugitive	CO2Storage		**0.0038**	Gt_CO2/Gt
ExtractedOilGas	NETNoFugitive	CO2Product	**-0.0038**		Gt_CO2/Gt
ExtractedOilGas	NETNoFugitive	Electricity	**-0.13**		EJ/Gt
ExtractedOilGas	NETNoFugitive	Electricity	**-0.021**		Gt/Gt
ExtractedOilGas	NETNoFugitive	NetEmissions		**0.068**	Gt_CO2e/Gt
ExtractedOilGas	NETNoFugitive	Oil	**-0.0037**		Gt/Gt
ExtractedOilGas	NETNoFugitive	ExtractedOilGas		**1.0**	Gt/Gt

Table S47: Coefficients for extracting oil and gas with elimination of fugitive emissions. Coefficients for carbon dioxide and carbon dioxide storage assume that the current rate of Enhanced Oil Recovery with carbon dioxide continues (page S124).

5.3.4.4 Electrification of oil and gas extraction with elimination of fugitive emissions

This delivery process describes extracting fossil oil and gas using electrified processes (Figure S25) using methods to eliminate venting, flaring and fugitive emissions. For modelling convenience, it has been assumed that processes venting, flaring and leakage abatement eliminate 100% fugitive emissions although this would be impossible (International Association of Oil & Gas Producers, 2000); realistic reductions in these emissions can be adjusted using the delivery process shares. Fugitive, venting and flaring emissions can be reduced by approaches such as: capturing gas rather than flaring and venting, improvements to equipment, and leak detection and repair systems (McKinsey & Company, 2020).

The coefficients are derived from the global energy use in 2018 as shown in Table S45, normalised by the quantity extracted in 2018, as given in Table S41.

Activity	Process	Resource	Inputs	Outputs	Unit
ExtractedOilGas	ElecNoFugitive	CO2Storage		**0.0038**	Gt_CO2/Gt
ExtractedOilGas	ElecNoFugitive	CO2Product	**-0.0038**		Gt_CO2/Gt
ExtractedOilGas	ElecNoFugitive	Electricity	**-1.2**		EJ/Gt
ExtractedOilGas	ElecNoFugitive	ExtractedOilGas		**1.0**	Gt/Gt

Table S48: Coefficients for extracting oil and gas using electrified processes and elimitating fugitive emissions. Coefficients for carbon dioxide and carbon dioxide storage assume that the current rate of Enhanced Oil Recovery with carbon dioxide continues (page S124).

5.3.5 Mining Metals and Minerals

This activity describes extraction of minerals and metal ores. Extracted minerals and metal ores is currently treated as one combined substance since the data is available in this aggregated form (Section 5.3.2). The substances could be treated separately in future, as described in SI Part 2.3. Processing of the extracted materials is not included in this sector, but accounted for in the Industry Sector (Section 5.7).

The delivery processes are derived from the global energy use in 2018 taken from *Mining and quarrying* in IEA energy balances (International Energy Agency, 2021a). Each process assumes a fuel switch. Fugitive emissions are assumed negligible (the majority of emissions are from fuel use according to Jolleys & Duddy (2022)). The energy demands and emissions calculated for each delivery process are normalised by the quantity of metals and industrial minerals mined (on a run-of-mine ore basis) in 2018, as given in Table S49.

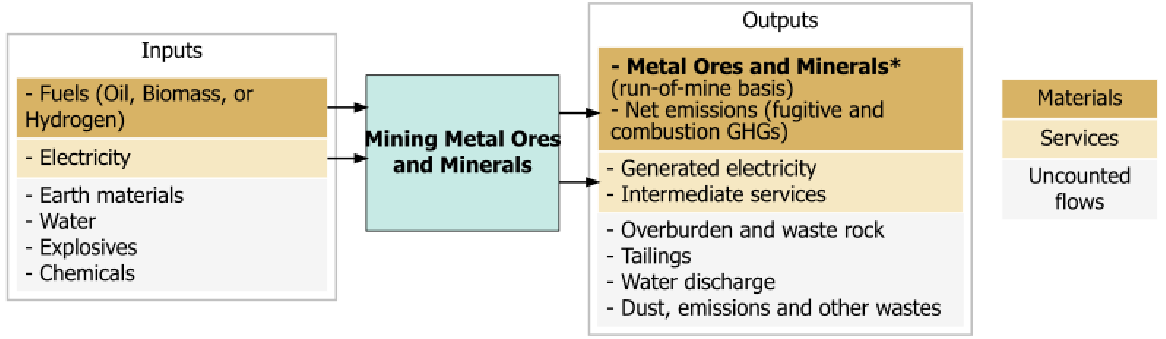

Figure S27: Inputs and outputs for mining minerals and metal ores.

Activity	Delivery process	Detail
MinedMetalsMinerals	NET	Conventional Methods
MinedMetalsMinerals	Elec	Electrical Processes replace Fossil Fuels
MinedMetalsMinerals	H2	Hydrogen replaces Fossil Fuels

Table S50: Delivery processes for mining minerals and metal ores.

Property	Values	Justification
2018 Global Extraction, Gt (run-of-mine ore basis)	53.0	The United Nations Environment Programme International Resource Panel Global Material Flows Database
Fugitive emissions (Gt CO2e /Gt material)	0	Assumed negligible since majority of emissions are from fuel use according to Jolleys and Duddy (2022)

Table S49: Data used to derive coefficients for metal & mineral mining delivery processes. References: (UNEP IRP, 2021), (Jolleys & Duddy, 2022)

5.3.5.1 Conventional metal & mineral mining processes

This delivery process assumes the current state and processes for metal & mineral mining.

The coefficients are derived from the global energy use in 2018 as shown in Table S51 normalised by the quantity of metals & minerals mined in 2018, as given in Table S49. The resulting coefficients are given in Table S52.

Parameter	Coal	Oil	Natural gas	Electricity	Total	Justification
**Summary of key data inputs and assumptions**						
Energy use, 2018; EJ/yr	0.3	1.0	0.5	1.5	**3.2**	From IEA energy balances
Energy density; GJ/t	28	42	45	N/A		See Model Assumptions Section of document
Fuel mass flow, 2018; Gt/yr	0.011	0.024	0.011	N/A	**128.0**	(energy use) x (emissions factor)
Emissions factor; Mt/EJ	100	70	60	0		In the absence of a detailed understanding of the processes involved, all fuels are assumed to have the same efficiency level
**Calculated coefficients (inputs)**						
Fuel demand coeff (Gt/Gt or EJ/Gt)	0.00020	0.00045	0.00020	0.028		(fuel flow or energy)/(2018 mass extracted)
**Calculated coefficients (outputs)**						
Combustion emissions (Gt CO2e/Gt)	0.001	0.001	0.001		**0.0024**	(emissions factor) x (energy used) / (2018 mass extracted)

Table S51: Assumptions used to derive coefficients for mining metal ores and minerals using conventional methods. IEA energy balances (*Mining and quarrying*) are used to estimate the fuel demands and combustion emissions, using the model assumptions for energy densities and emissions factors as described in the SI Part 2. The mass extracted in 2018 and the sources for fugitive emissions are given in Table S49.

Activity	Process	Resource	Inputs	Outputs	Unit
MinedMetalsMinerals	NET	Electricity	**-0.028**		EJ/Gt
MinedMetalsMinerals	NET	NetEmissions		**0.0024**	Gt_CO2e/Gt
MinedMetalsMinerals	NET	Coal	**-0.00020**		Gt/Gt
MinedMetalsMinerals	NET	Oil	**-0.00045**		Gt/Gt
MinedMetalsMinerals	NET	Methane	**-0.00020**		Gt/Gt
MinedMetalsMinerals	NET	MinedMetalsMinerals		**1.0**	Gt/Gt

Table S52: Coefficients for metal & mineral mining using conventional methods.

5.3.5.2 Electrification of metal & mineral mining

This delivery process assumes that all processes for metal & mineral mining are fully electrified. Although this is an unlikely extreme, it can be used to explore opportunities for some electrification.

The coefficients are derived from the global energy use in 2018 as shown in Table S53 normalised by the quantity of metals & minerals mined in 2018, as given in Table S49. The resulting coefficients are given in Table S54.

Parameter	Coal	Oil	Natural gas	Electricity	Total	Justification
**Summary of key data inputs and assumptions**						
Energy use, 2018; EJ/yr	0.3	1.0	0.5	1.5	**3.2**	From IEA energy balances
Assumed relative energy consumption (electrification)	100%	100%	100%	100%		In the absence of a detailed understanding of the processes involved, all fuels are assumed to have the same efficiency level
Assumed energy for electrified mining, EJ/yr	0	0	0	3.2	**3.2**	All energy demand met by electricity
**Calculated coefficients (inputs)**						
Demand coefficient (EJ/Gt)			0.061			(total energy demand) / (2018 mass extracted)

Table S53: Assumptions used to derive coefficients for MinedMetalsMinerals mining with electrified processes. IEA energy balances (*Mining and quarrying*) are used to estimate the fuel demands using the model assumptions for energy

densities given in the SI Part 2. The mass extracted in 2018 and the sources for fugitive emissions are given in Table S49.

Activity	Process	Resource	Inputs	Outputs	Unit
MinedMetalsMinerals	Elec	Electricity	-0.061		EJ/Gt
MinedMetalsMinerals	Elec	MinedMetalsMinerals		1.0	Gt/Gt

Table S54: Coefficients for metal & mineral mining with electrified processes.

5.3.5.3 Hydrogen-powered metal & mineral mining

This delivery process assumes that processes which are conventionally powered by fossil fuels, are instead powered by hydrogen.

The coefficients are derived from the global energy use in 2018 as shown in Table S55 normalised by the quantity of metals & minerals mined in 2018, as given in Table S49. The resulting coefficients are given in Table S56.

Parameter	Coal	Oil	Natural gas	Electricity	Hydrogen	Total	Justification
**Summary of key data inputs and assumptions**							
Energy use, 2018; EJ/yr	0.3	1.0	0.5	1.5		**3.2**	From IEA energy balances
Assumed relative energy consumption (hydrogen powered)	100%	100%	100%	N/A	100%		In the absence of a detailed understanding of the processes involved, all fuels are assumed to have the same efficiency level
Assumed energy for H2-powered mining, EJ/yr				1.5	1.8	**3.2**	All energy demand met by electricity
Energy density; GJ/t	28	42	45	N/A	120		See Model Assumptions Section of document
**Calculated coefficients (inputs)**							
Fuel demand coeff (Gt/Gt or EJ/Gt)			0.00028	0.028			(energy use) / (energy density) / (2018 mass extracted)

Table S55: Assumptions used to derive coefficients for MinedMetalsMinerals mining with hydrogen-powered processes. IEA energy balances (*Mining and quarrying*) are used to estimate the fuel demands and combustion emissions, using the model assumptions for energy densities and emissions factors as described in the SI Part 2. The mass extracted in 2018 and the sources for fugitive emissions are given in Table S49.

Activity	Process	Resource	Inputs	Outputs	Unit
MinedMetalsMinerals	H2	Electricity	-0.028		EJ/Gt
MinedMetalsMinerals	H2	Hydrogen	-0.00028		Gt/Gt
MinedMetalsMinerals	H2	MinedMetalsMinerals		1.0	Gt/Gt

Table S56: Coefficients for metal & mineral mining using hydrogen-powered processes.

5.3.6 Mining and Extraction Coefficient Summary

ResourceFlow	ExtractedOilGas_NET	ExtractedOilGas_Elec	ExtractedOilGas_NETNoFugitive	ExtractedOilGas_ElecNoFugitive	Coal_NET	Coal_H2	Coal_Elec	Coal_NETNoFugitive	Coal_H2NoFugitive	Coal_ElecNoFugitive	MinedMetalsMinerals_NET	MinedMetalsMinerals_H2	MinedMetalsMinerals_Elec
CO2Storage, Gt_CO2	0.0038	0.0038	0.0038	0.0038									
CO2Product, Gt_CO2	-0.0038	-0.0038	-0.0038	-0.0038									
Electricity, EJ	-0.13	-1.2	-0.13	-1.2	-0.066	-0.066	-0.21	0.0037	0.0039	-0.14	-0.028	-0.028	-0.061
Electricity, Gt	-0.021		-0.021										
NetEmissions, Gt_CO2e	0.46	0.39	0.068		0.21	0.20	0.20	0.013			0.0024		
Oil, Gt	-0.0037		-0.0037		-0.0012			-0.0012			-0.00045		
ExtractedOilGas, Gt	1.0	1.0	1.0	1.0									
Coal, Gt_Coal					1.0	1.0	1.0	1.0	1.0	1.0			
Hydrogen, Gt						-0.0012			-0.0012			-0.00028	
Coal, Gt											-0.00020		
Methane, Gt											-0.00020		
MinedMetalsMinerals, Gt											1.0	1.0	1.0

Table S57: Coefficients for mining and extraction processes. The table shows the coefficients for the mining and extraction sector. Negative values are inputs to the process, positive are outputs.

5.4 Electricity Generation and Distribution

This section outlines the derivation of coefficients for electricity generation, i.e. the flows needed to provide electricity to power any downstream process.

There are two activities in this section: electricity generation (the production of electricity from other energy sources, producing the model flow, *GeneratedElectricity*) and electricity distribution (the distribution of electricity from the point of generation to the point of use, producing the model flow, *Electricity*), as listed in Table S58.

Sector	Activity	Model Name	ISIC Code
Electricity	Distribution of Electricity	Electricity	351
Electricity	Electricity Generation	GeneratedElectricity	351

Table S58: Model flows categorised by ISIC divisions, groups and classes for this Sector.

5.4.1 Sector-wide Data Sources

Coefficients are for electricity generation are mostly derived from median generation efficiency values given in Annex III of the Contribution of Working Group III to the Fifth Assessment Report of the Intergovernmental Panel on Climate Change (Schlömer et al., 2014). These values are combined with energy density estimates and emissions factors for the fuels used (from the SI Part 2) to estimate the fuel demands and residual emissions. A summary of the data and assumptions used for all delivery processes is given in Table S59.

Parameter	Justification	Coal (PC)	Coal with CCS (PC)	Gas (CC)	Gas with CCS (CC)	Dedicated Biomass	BECCS
Efficiency	Estimated from Table A.III.1 of IPCC WG3 AR5 (Schloemer et al., 2014)	39%	30%	55%	47%	31%	22%
Fuel energy density (EJ/Gt)	See assumptions section of document	28	28	45	45	15	15
Emissions factor (kg CO2/GJ fuel)	See assumptions section of document	100	100	60	60	0	122
Assumed capture rate	Consistent with assumptions used by Muratori et al. (2017), and values in Gomez (2006)	0%	90%	0%	90%	0%	90%

Note:
CC: Combined Cycle; CCS: Carbon Capture and Storage; PC: Pulverized Coal
Processes have been chosen to be at the higher end of efficiency values for each fuel type.

Table S59: Assumptions and data used to estimate coefficients for electricity generation. Efficiency estimates are taken to be the median values from Table A.III.1 of IPCC WG3 AR5 Annex III, with the exception of Biomass Energy with CCS (BECCS) (Schlömer et al., 2014). For the BECCS process, there is no value given in the reference; instead the efficiency is assumed to have the same efficiency penalty from the biomass fuelled process, as for other fuels. Fuel energy density and emissions values are from the SI Part 2.

5.4.2 Electricity Generation

This activity describes the production of electricity from other energy sources (Figure S28). Modelled delivery processes are listed in Table S60.

For electricity generation from non-emitting sources, the energy inputs are quantified as the demand for a source of non-emitting electricity (rather than fuel or or other energy sources) since the aim of the model is to quantify the overall demand for non-emitting electricity (see Section 5.4.2.1).

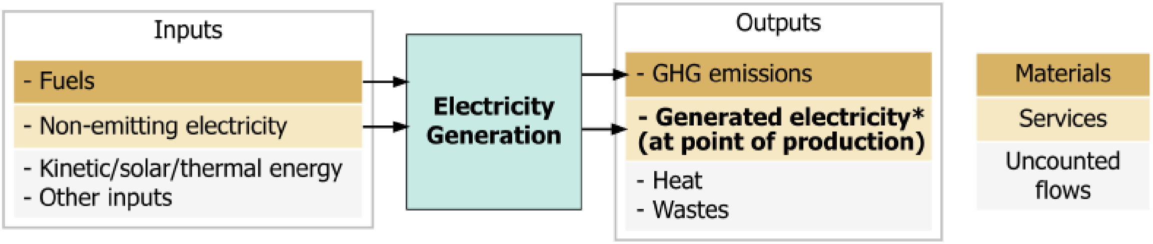

Figure S28: Inputs and outputs for generating electricity.

Activity	Process	Process Long Name
GeneratedElectricity	NonEmitting	Non-Emitting generation (renewable and nuclear)
GeneratedElectricity	Coal	Coal Powered
GeneratedElectricity	Gas	Gas Powered
GeneratedElectricity	CoalCCS	Coal Powered with integrated carbon capture
GeneratedElectricity	GasCCS	Gas Powered with integrated carbon capture
GeneratedElectricity	Bio	From Biomass (without carbon capture)
GeneratedElectricity	BECC	Bio-Energy with carbon capture (BECC)

Table S60: Delivery processes for this activity. The coefficients for each delivery process are given and explained in the remainder of this section. The contents

5.4.2.1 Non-Emitting Electricity (NEE) generation

This process describes production of electricity from non-emitting sources (non-biomass renewables and nuclear). The energy inputs are quantified as the demand for a source of non-emitting electricity - rather than fuel or or other energy sources - since the aim of the model is to quantify the overall demand for non-emitting electricity. In this way, the flow, *Non-Emitting Electricity*, is treated as an indicator or metric. In a future development of the model, the process to produce *Non-Emitting Electricity* could be split into processes for each type of non-emitting electricity generation technology. In that way it would be possible to also quantify demands for nuclear fuel, land use for solar panels, rare earth metals or other resources of interest. See more detail about extending the model in SI Part 2.3.

Although life-cycle emissions can be significant (Schlömer et al., 2014), these have been excluded at this stage; overall demands may thus be higher than estimated here. Excluding infrastructure and supply-chain emissions gives zero remaining emissions for most renewable energy sources except hydro-power, which creates environments for methane generation (Schlömer et al., 2014). It is assumed here that the relative contribution of hydro power will be small so that these residual emissions are relatively small. On this basis, the coefficients for Non-Emitting Electricity Generation are shown in Table S61.

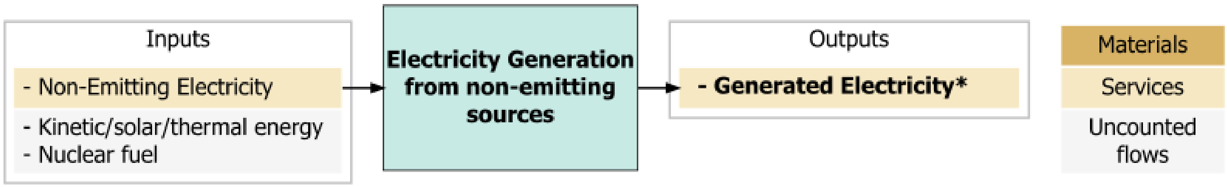

Figure S29: Inputs and outputs for generating electricity from non-emitting sources.

Activity	Process	Resource	Inputs	Outputs	Unit
GeneratedElectricity	NonEmitting	NEElectricity	-1.0		EJ/EJ
GeneratedElectricity	NonEmitting	GeneratedElectricity		1.0	EJ/EJ

Table S61: Coefficients for Non-Emitting Electricity Generation

5.4.2.2 Conventional coal-powered electricity generation

This process describes electricity generation from combustion of coal, based on pulverized coal (PC) plants. Coefficients are based on efficiency estimates compiled by the IPCC in Table S59. The plant efficiency is combined with energy density estimates and emissions factors for the fuels used to estimate the fuel demands and residual emissions (Table S62). The coefficients are given in Table S63.

Only direct emissions are considered here. Fugitive methane emissions are considered in the mining sector (Section 5.3).

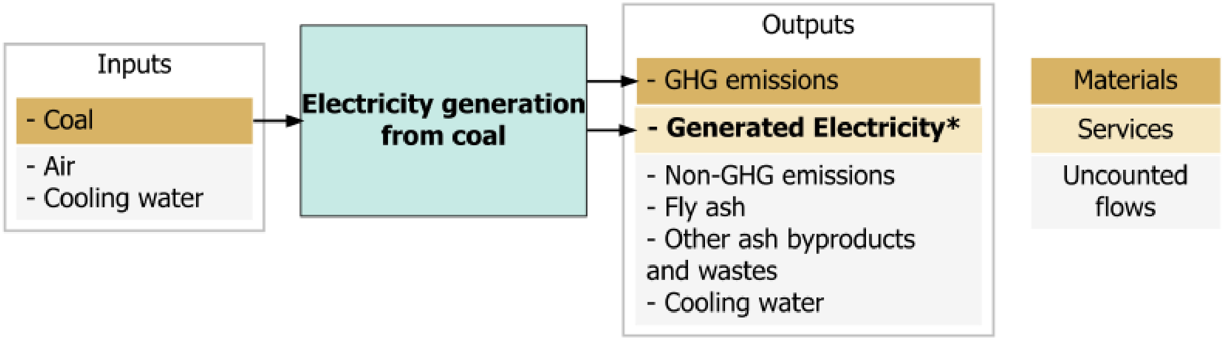

Figure S30: Inputs and outputs for generating electricity from coal.

Parameter	Coal (PC)	Justification
**Summary of key data inputs and assumptions**		
Efficiency	39%	Estimated from Table A.III.1 of IPCC WG3 AR5 (Schloemer et al., 2014)
Fuel energy density (EJ/Gt)	28	See assumptions section of document
Emissions factor (kg CO2/GJ fuel)	100	See assumptions section of document
**Calculated coefficients (inputs)**		
Fuel demand (t/GJ elec)	0.09	1/ (efficiency)/ (energy density)
**Calculated coefficients (outputs)**		
Combustion emissions (t CO2/GJ elec)	0.26	1/ (efficiency) x (emissions factor)

Note:
PC: Pulverized Coal

Table S62: Assumptions and data used to estimate coefficients for coal powered electricity generation. The efficiency estimate is the median values from Table A.III.1 of IPCC WG3 AR5 Annex III (Schlömer et al., 2014). Fuel energy density and emissions values are from the SI Part 2.

Activity	Process	Resource	Inputs	Outputs	Unit
GeneratedElectricity	Coal	Coal	-0.092		Gt/EJ
GeneratedElectricity	Coal	NetEmissions		0.26	Gt_CO2e/EJ
GeneratedElectricity	Coal	GeneratedElectricity		1.0	EJ/EJ

Table S63: Coefficients for conventional coal powered electricity generation

5.4.2.3 Conventional gas powered electricity generation

This process describes electricity generation from combustion of methane, based on combined cycle gas plants. Coefficients are based on efficiency estimates compiled by the IPCC in Table S59. The plant efficiency is combined with energy density estimates and emissions factors for the fuels used to estimate the fuel demands and residual emissions (Table S64). The coefficients are given in Table S63.

Only direct emissions are considered here. Emissions from leakage (or synthetic methane production) are considered in the processes for extracting gas (Section 5.3) and methane production (Section 5.6.4).

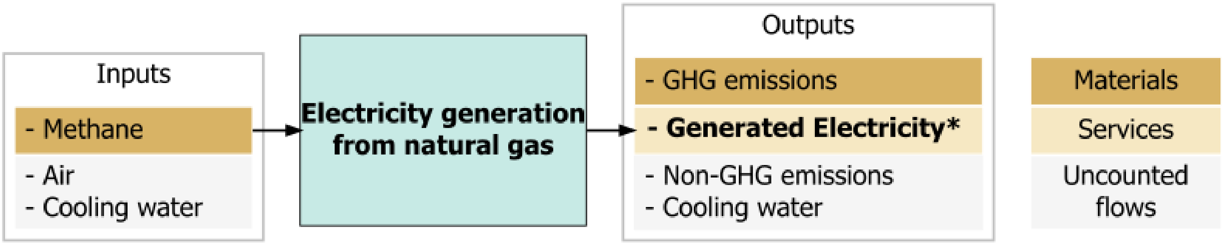

Figure S31: Inputs and outputs for generating electricity from methane or natural gas.

Parameter	Gas (CC)	Justification
**Summary of key data inputs and assumptions**		
Efficiency	55%	Estimated from Table A.III.1 of IPCC WG3 AR5 (Schloemer et al., 2014)
Fuel energy density (EJ/Gt)	45	See assumptions section of document
Emissions factor (kg CO2/GJ fuel)	60	See assumptions section of document
**Calculated coefficients (inputs)**		
Fuel demand (t/GJ elec)	0.04	1/ (efficiency)/ (energy density)
**Calculated coefficients (outputs)**		
Combustion emissions (t CO2/GJ elec)	0.11	1/ (efficiency) x (emissions factor)

Note:
CC: Combined Cycle

Table S64: Assumptions and data used to estimate coefficients for gas powered electricity generation. The efficiency estimate is the median values from Table A.III.1 of IPCC WG3 AR5 Annex III (Schlömer et al., 2014). Fuel energy density and emissions values are from the SI Part 2.

Activity	Process	Resource	Inputs	Outputs	Unit
GeneratedElectricity	Gas	Methane	-0.040		Gt/EJ
GeneratedElectricity	Gas	NetEmissions		0.11	Gt_CO2e/EJ
GeneratedElectricity	Gas	GeneratedElectricity		1.0	EJ/EJ

Table S65: Coefficients for conventional gas powered electricity generation

5.4.2.4 Electricity generation from coal with carbon-dioxide capture

This process describes electricity generation from combustion of coal with carbon-dioxide capture. Coefficients are based on efficiency estimates compiled by the IPCC in Table S59. The plant efficiency is combined with an estimated energy density and emissions factor to calculate the fuel demands and total emissions (Table S66). An assumed capture rate of 90% is used to allocate the combustion emissions against demand for carbon storage, and residual emissions. The capture rate is consistent with values in Gómez et al. (2006) and assumptions used by Muratori et al. (2017). The coefficients are given in Table S67.

Only direct emissions are considered here. Emissions from fugitive emissions in mining are considered in the processes for coal mining (Section 5.3).

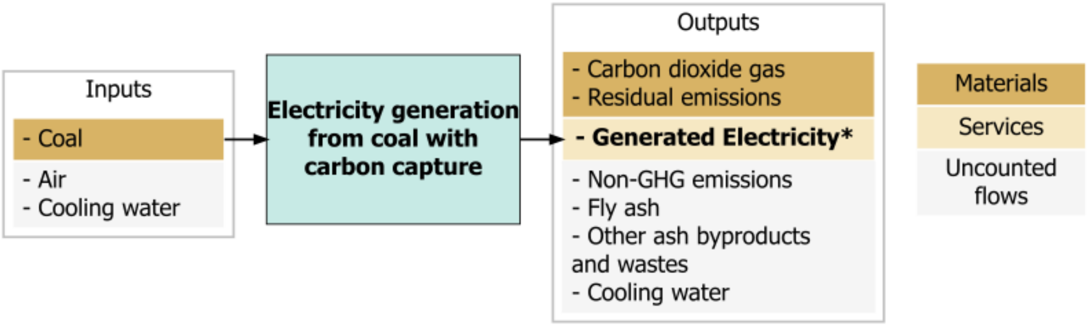

Figure S32: Inputs and outputs for generating electricity from coal with carbon-dioxide capture.

Parameter	Coal with CCS (PC)	Justification
**Summary of key data inputs and assumptions**		
Efficiency	30%	Estimated from Table A.III.1 of IPCC WG3 AR5 (Schloemer et al., 2014)
Fuel energy density (EJ/Gt)	28	See assumptions section of document
Emissions factor (kg CO2/GJ fuel)	100	See assumptions section of document
Assumed capture rate	90%	Consistent with assumptions used by Muratori et al. (2017), and values in Gomez (2006)
**Calculated coefficients (inputs)**		
Fuel demand (t/GJ elec)	0.12	1/ (efficiency)/ (energy density)
**Calculated coefficients (outputs)**		
Combustion emissions (t CO2/GJ elec)	0.33	1/ (efficiency) x (emissions factor)
Captured CO2 (t CO2/GJ elec)	0.30	(emissions) x (capture rate)
Residual CO2 (t CO2/GJ elec)	0.03	(emissions) - (captured CO2)

Note:
CCS: Carbon Capture and Storage; PC: Pulverized CoalCCS

Table S66: Assumptions and data used to estimate coefficients for coal powered electricity generation with carbon-dioxide capture. The efficiency estimate is the median values from Table A.III.1 of IPCC WG3 AR5 Annex III (Schlömer et al., 2014). Fuel energy density and emissions values are from the SI Part 2.

Activity	Process	Resource	Inputs	Outputs	Unit
GeneratedElectricity	CoalCCS	Coal	-0.12		Gt/EJ
GeneratedElectricity	CoalCCS	CO2Product		0.30	Gt_CO2/EJ
GeneratedElectricity	CoalCCS	NetEmissions		0.033	Gt_CO2e/EJ
GeneratedElectricity	CoalCCS	GeneratedElectricity		1.0	EJ/EJ

Table S67: Coefficients for Electricity Generation from coal with carbon-dioxide capture

5.4.2.5 Electricity generation from gas with carbon-dioxide capture

This process describes electricity generation from combustion of methane with with carbon-dioxide capture. Coefficients are based on efficiency estimates compiled by the IPCC in Table S59. The plant efficiency is combined with an estimated energy density and emissions factor to calculate the fuel demands and total emissions (Table S68). An assumed capture rate of 90% is used to allocate the combustion emissions against demand for carbon storage, and residual emissions. The capture rate is consistent with values in Gómez et al. (2006) and assumptions used by Muratori et al. (2017). The coefficients are given in Table S69.

Only direct emissions are considered here. Emissions from leakage (or synthetic methane production) are considered in the processes for extracting gas (Section 5.3) and methane production (Section 5.6.4).

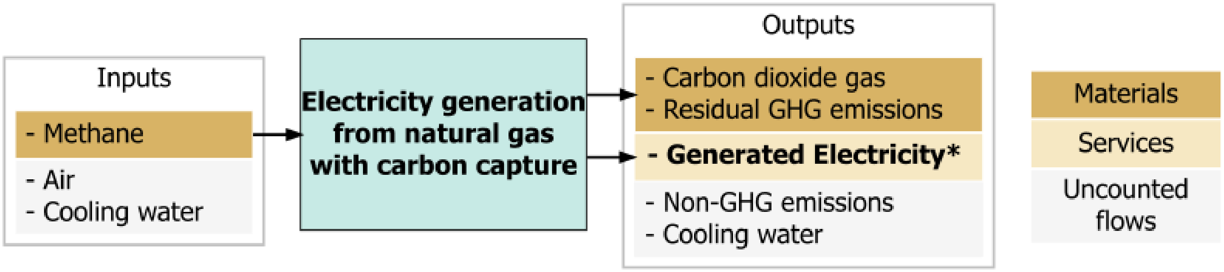

Figure S33: Inputs and outputs for generating electricity from methane or natural gas with carbon-dioxide capture.

Parameter	Gas with CCS (CC)	Justification
**Summary of key data inputs and assumptions**		
Efficiency	47%	Estimated from Table A.III.1 of IPCC WG3 AR5 (Schloemer et al., 2014)
Fuel energy density (EJ/Gt)	45	See assumptions section of document
Emissions factor (kg CO2/GJ fuel)	60	See assumptions section of document
Assumed capture rate	90%	Consistent with assumptions used by Muratori et al. (2017), and values in Gomez (2006)
**Calculated coefficients (inputs)**		
Fuel demand (t/GJ elec)	0.05	1/ (efficiency)/ (energy density)
**Calculated coefficients (outputs)**		
Combustion emissions (t CO2/GJ elec)	0.13	1/ (efficiency) x (emissions factor)
Captured CO2 (t CO2/GJ elec)	0.11	(emissions) x (capture rate)
Residual CO2 (t CO2/GJ elec)	0.01	(emissions) - (captured CO2)

Note:
CC: Combined Cycle; CCS: Carbon Capture and Storage

Table S68: Assumptions and data used to estimate coefficients for gas powered electricity generation with carbon-dioxide capture. The efficiency estimate is the median values from Table A.III.1 of IPCC WG3 AR5 Annex III (Schlömer et al., 2014). Fuel energy density and emissions values are from the SI Part 2.

Activity	Process	Resource	Inputs	Outputs	Unit
GeneratedElectricity	GasCCS	Methane	-0.047		Gt/EJ
GeneratedElectricity	GasCCS	CO2Product		0.11	Gt_CO2/EJ
GeneratedElectricity	GasCCS	NetEmissions		0.013	Gt_CO2e/EJ
GeneratedElectricity	GasCCS	GeneratedElectricity		1.0	EJ/EJ

Table S69: Coefficients for Electricity Generation from gas with carbon-dioxide capture

5.4.2.6 Biomass Fuelled Electricity Generation

This process describes electricity generation from a dedicated biomass process (rather than co-firing). Several technical challenges need to be overcome for this to be viable at scale, according to Mander et al. (2017); these are not considered here. For context, the range of efficiency expected from biomass-fuelled plants is given in Table S70.

Coefficients are based on efficiency estimates compiled by the IPCC in Table S59. The plant efficiency is combined with energy density estimates and emissions factors for the fuels used to estimate the fuel demands and residual emissions (Table S71). The coefficients are given in Table S72.

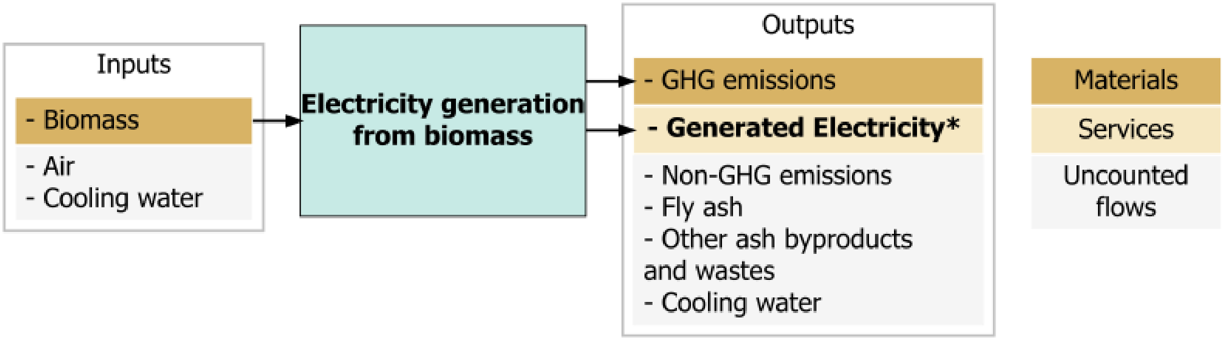

Figure S34: Inputs and outputs for generating electricity from biomass.

Plant type	Min	Median	Max
Biomass-CHP	0.14	0.29	0.36
Biomass-cofiring	0.38	0.41	0.48
Biomass-dedicated	0.20	0.31	0.48

Table S70: Biomass electrical generation efficiencies from Schlömer et al. (2014)

Parameter	Dedicated Biomass	Justification
**Summary of key data inputs and assumptions**		
Efficiency	31%	Estimated from Table A.III.1 of IPCC WG3 AR5 (Schloemer et al., 2014)
Fuel energy density (EJ/Gt)	15	See assumptions section of document
Emissions factor (kg CO2/GJ fuel)	0	See assumptions section of document
**Calculated coefficients (inputs)**		
Fuel demand (t/GJ elec)	0.22	1/ (efficiency)/ (energy density)
**Calculated coefficients (outputs)**		
Combustion emissions (t CO2/GJ elec)	0.00	1/ (efficiency) x (emissions factor)

Table S71: Assumptions and data used to estimate coefficients for biomass powered electricity generation. The efficiency estimate is the median value from Table A.III.1 of IPCC WG3 AR5 Annex III (Schlömer et al., 2014). Fuel energy density and emissions values are from the SI Part 2.

Activity	Process	Resource	Inputs	Outputs	Unit
GeneratedElectricity	Bio	Wood	-0.22		Gt_DryBio/EJ
GeneratedElectricity	Bio	GeneratedElectricity		1.0	EJ/EJ

Table S72: Coefficients for Electricity Generation from a dedicated biomass facility.

5.4.2.7 Bio-Energy with carbon-dioxide capture (BECC)

This process describes electricity generation from combustion of biomass with carbon capture. Storage is not included in this process but in the process for carbon dioxide storage (Section 5.11.2) to allow for Carbon Capture and Use (CCU) applications. The biomass input is derived from an estimate of the plant efficiency: the plant with carbon-dioxide capture is assumed to operate similarly to the biomass-fuelled delivery process (Section 5.4.2.6) but with a 9% efficiency penalty. This is consistent with the efficiency loss from adding CCS to generation from other fossil-fuels (Table S59). Approximated values for biomass energy density and carbon content are used to calculated the fuel demands and total emissions (Table S73). An assumed capture rate of 90% is used to allocate the combustion emissions against demand for carbon storage, and residual emissions, consistent with the other processes for electricity generation with carbon-dioxide capture.

Residual emissions appear negative for this process. For the model in general, it is assumed that the carbon in emissions from biomass (from respiration, combustion and decomposition) is balanced by carbon sequestration in growing biomass within the same year and so assigned an emissions factor of zero (see Biogenic Carbon Section in the document, SI Part 1). Where carbon stored in biomass is combusted as part of a Carbon Capture and Storage process such as this one, however, the carbon stored in the system is assumed to be permanently sequestered. This imbalance must be accounted for as a negative emission equal in value to the amount of carbon dioxide stored by the system.

For any potential facility, there could be a wide variation in the quantities of electricity generated and carbon captured from a given input of biomass. This will depend on operational decisions and priorities i.e. whether to maximise energy production or carbon capture. To reflect this breadth there are two processes for BECCS; one of which is this process, representing a system optimised for electricity generation, the other is a NET process, representing a system optimised for carbon capture (see Section 5.11). The values chosen for this (electricity generation) process are broadly consistent with the model developed by Almena et al. (2022) for a BECC system, optimised for electricity generation.

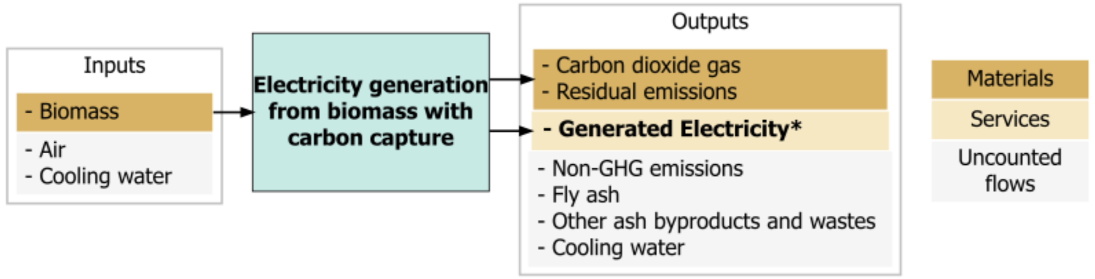

Figure S35: Inputs and outputs for generating electricity from biomass with carbon capture and storage.

Parameter	BECCS	Justification BECCS
**Summary of key data inputs and assumptions**		
Efficiency	22%	Estimated from Table A.III.1 of IPCC WG3 AR5 (Schloemer et al., 2014) with energy penalty for CCS as for coal
Fuel energy density (EJ/Gt)	15	See assumptions section of document
Emissions factor (kg CO2/GJ fuel)	122	See assumptions section of document
Assumed capture rate	90%	Consistent with assumptions used by Muratori et al. (2017), and values in Gomez (2006)
**Calculated coefficients (inputs)**		
Fuel demand (t/GJ elec)	0.30	1/ (efficiency)/ (energy density)
**Calculated coefficients (outputs)**		
Combustion emissions (t CO2/GJ elec)	0.55	1/ (efficiency) x (emissions factor)
Captured CO2 (t CO2/GJ elec)	0.50	(emissions) x (capture rate)
Residual CO2 (t CO2/GJ elec)	-0.50	Since carbon sequestration is not counted in Agriculture, it must accounted for here. See document assumptions section.

Table S73: Assumptions and data used to estimate coefficients for biomass powered electricity generation with carbon-dioxide capture. The efficiency is derived from the median values from Table A.III.1 of IPCC WG3 AR5 Annex III (Schlömer et al., 2014). Fuel energy density and emissions values are from the SI Part 2.

Activity	Process	Resource	Inputs	Outputs	Unit

Table S74: Coefficients for Electricity Generation with BECCS

5.4.3 Electricity Distribution

This activity describes the distribution of electricity from the point of generation to the point of use. The energy losses in distribution are approximated based on 2018 data. The 2018 ratio of *energy supplied* to *calculated consumption* is used to scale the available electricity to downstream activities. From International Energy Agency (2022), global electricity produced in *transformation processes* was 96EJ for a *total final consumption* of 83 EJ, giving distribution losses of 13.5%.

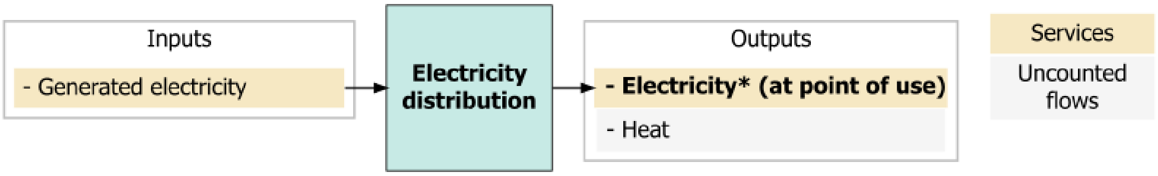

Figure S36: Inputs and outputs for electricity distribution.

Activity	Process	Resource	Inputs	Outputs	Unit
Electricity	Distribution	GeneratedElectricity	-1.1		EJ/EJ
Electricity	Distribution	Electricity		1.0	EJ/EJ

Table S75: Coefficients for electricity distribution.

5.4.4 Coefficient summary

Table S76 shows all process coefficients for the activities, electricity generation and electricity distribution.

		Electricity generation processes						
ResourceFlow	Distribution	Coal	Gas	CoalCCS	GasCCS	Bio	BECC	NonEmitting
GeneratedElectricity, EJ	-1.1	1.0	1.0	1.0	1.0	1.0	1.0	1.0
Electricity, EJ	1.0							
Coal, Gt		-0.092		-0.12				
NetEmissions, Gt_CO2e		0.26	0.11	0.033	0.013		-0.50	
Methane, Gt			-0.040		-0.047			
CO2Product, Gt_CO2				0.30	0.11		0.50	
Wood, Gt_DryBio						-0.22	-0.30	
NEElectricity, EJ								-1.0

Table S76: Coefficients for Electricity Generation

5.5 Agriculture and Forestry

This section outlines the derivation of coefficients for processes which provide resources from agriculture and forests. There are considered to be three activities within this category, shown in Table S80.

The processes within this sector produce different types of biomass from *BiomassCapacity*, one of the three ZERs. Biomass is treated as two different resources, woody biomass from trees (*Wood*) and biomass from agriculture or waste (*NonWoodBiomass*). Growing pasture and using residues are treated as separate delivery process options within the activity for biomass agriculture (*NonWoodBiomass*).

The activity metrics have been chosen to represent the end-use activity as closely as possible; this means that although biomass is accounted for in tonnes (consistent with other fuels and materials in the model), food production is quantified in terms of calories. Using calories helps in describing options for diet change.

Sector	Model Flows Produced	ISIC Code	Description (ISIC Classification)
AgriForestry	NonWoodBiomass	11, 12, 13, 16	Growing of non-perennial crops, Growing of perennial crops, Plant propagation, Support activities to agriculture and post-harvest crop activities
AgriForestry	RawFood	14, 15, 17, 3	Animal production, Mixed farming, Hunting, trapping and related service activities, Fishing and aquaculture
AgriForestry	Wood	2	Forestry and logging

Table S80: Model flows categorised by ISIC divisions, groups and classes for this Sector.

5.5.1 Overarching sector assumptions

The following key assumptions have been used in deriving coefficients for agriculture and forestry:

- Sequestration of carbon in biomass is not explicitly accounted for

Since the majority of carbon sequestered in biomass is relatively quickly returned to the atmosphere, it is assumed that the carbon in emissions from biomass (from respiration, combustion and decomposition) is balanced by carbon sequestration in growing biomass within the same year. This means that carbon dioxide absorbed in photosynthesis, and emitted in respiration or combustion, does not need to be explicitly accounted for, consistent with IPCC 2006 Guidelines (Eggleston et al., 2006).

- Land use and land use change is not accounted for

Although a significant part of the environmental impact of both agriculture and forestry is their effect on natural carbon-sinks, these land-use and land-use change (LULUC) emissions are not accounted for in the model. LULUC emissions are difficult to assign to any specific process (Geist & Lambin, 2002), are specific to the local conditions, and cannot be assigned to a specific snapshot in time without an understanding of the change trajectory. Instead, we have assumed that almost all emissions from land-use change (deforestation and forest degradation) stops by 2050 but there is no permanent net benefit from reforestation or other land-use, giving us net zero emissions in 2050 from LULUC. Refer to the Supply Estimation SI document for a longer discussion of LULUC.

- Only urea-based nitrogen fertiliser is accounted for

Nitrogen (N) fertiliser is accounted for as in input to Biomass Agriculture since it is created from Ammonia which is used as a an energy source for other processes within the model. N-fertiliser made up around 60% of all fertiliser mass used in agriculture in 2018 according to data in (FAO, 2022b) (with the residual almost equal parts phosphate and potash by mass), while around 65% of N-fertilisers are urea based (Gao & Cabrera Serrenho, 2023).

5.5.2 Sector-wide data sources and data manipulation

Production quantity data for the Agriculture and Forestry sector has mostly been taken from FAOSTAT databases (FAO (2022a), FAO (2022d), FAO (2022c), FAO (2022b)). Energy consumption data has been taken from IEA World Energy Balances (International Energy Agency, 2021a), and emissions data from a synthetic dataset compiled by Minx et al. (2021), used in the IPCC AR6 report.

The manipulation of these data sources to derive useful estimates for process coefficients is described in the rest of this section.

5.5.2.1 Emissions and emissions attribution Having excluded LULUC emissions (see Section 5.5.1), the most significant source of emissions in agriculture and forestry are process emissions, including methane production in rice paddies and from ruminant farming. 2018 emissions data for process emissions have been attributed to each activity using an allocation matrix to distribute emissions amongst drivers. The total emissions for each activity were then divided by the physical quantity of activity in 2018 to give the process emissions per unit product used. The 2018 emissions data for AgricultureForests are taken from the synthetic dataset compiled by Minx et al. (2021), used in the IPCC AR6 report (Nabuurs & Mrabet, 2022).

The allocation of emissions amongst activities is given in Table S81. Emissions from manure have been assumed to be split 50:50 between ruminant and non-ruminant meat products. This is based on the distribution of animal products that are ruminant; according to (FAO, 2022c) this is 43% when accounted for in calories, and 55% when accounted for in mass terms.

Emissions Process	Farming (plant-based food)	Farming (non-ruminants)	Farming (ruminants)	Biomass agriculture	2018 Emissions Data, Gt CO2e
**Enteric Fermentation**			100%		**2.9**
**Managed soils & pasture**		10%	10%	80%	**1.4**
**Rice cultivation**	100%				**1.0**
**Manure management**		50%	50%		**0.4**
**Synthetic fertiliser application**				100%	**0.4**
**Biomass burning (agricultural residue)**				100%	**0.07**
**TOTAL Attributed Emissions, Gt CO2e**	**1.0**	**0.4**	**3.3**	**1.7**	**6.3**

Table S81: The chosen emissions allocation matrix. Total emissions Emissions data for this section are taken from a synthetic dataset compiled by Minx et al. (2021). Emissions distribution is based on assumptions and data from FAO (2022c).

5.5.2.2 Energy consumption data and allocation Energy consumption in agriculture is derived from the review by Woods et al. (2010) of energy use in the food system. For simplicity, the fuel distribution is assumed to be a mix of oil (diesel) and electricity only. Energy consumption from forestry is then calculated as the remaining unaccounted energy use in agriculture forestry and fishing in 2018 from IEA Summary Energy Balances (2021a).

- **For biomass agriculture:** Based on Woods et al. (2010), arable crops require an energy input of around 2 GJ/t in England and Wales, of which around 50% is associated with fertiliser and pesticide manufacture, 10% with post harvest (e.g. storing in a cooled environment), and 40% is for diesel for farm machinery. This suggests around 0.2 GJ/t electricity use and 0.8 GJ/t oil consumption. There is significant differences between different crops and farming methods; values cited by Woods et al. (2010) vary from 0.2 GJ/t for sugarcane in Brazil to 6 GJ/t for organic oilseed rape in England and Wales.
- **For ruminant and non-ruminant farming:** Based on Woods et al. (2010), production of animal-based food in England and Wales has an energy requirement of around 20 GJ/t (varying from around 3 GJ/t for milk and 12 GJ/t for eggs to around 20 for pig- or lamb- meat and 30 GJ/t for beef), of which only around 25% is direct energy (with the remainder for feed). The values cited by Woods et al. (2010) suggest around 40% of primary energy use is oil. Here we assume the remainder is provided by electricity.
  - It is assumed that 4.5 GJ/t are needed for non-ruminant animal-products, of which 1.8 GJ/t is oil and 2.7 GJ/t is electricity.
  - For ruminant animal products, since energy intensity of dairy is significantly lower, the assumed requirements are 1.2 GJ/t oil and 1.8 GJ/t electricity. These values are converted to the values per kcal (as required for the production metric used in the model) using 2018 production quantities in Table S83 and Table S84.
- **For forestry:** Energy use is calculated as the residual unaccounted energy use in agriculture forestry and fishing in 2018 from IEA Summary Energy Balances (2021a). It is assumed that for agriculture and forestry overall 40% energy is supplied as electricity and 60% as oil - this is a simplification of 2018 energy consumption where 53% was supplied as oil products, 30% as electricity and heat, and other fuels supplying the residual.

Quantity	Farmed biomass	Ruminent	Non-ruminent	Forestry	Source
2018 Production (Gt)	5.6	0.63	0.63	1.8	FAOSTAT data (Gt, edible carcass weight for meat, dry weight for wood)
2018 Production (10^15 kcal)	N/A	0.63	0.85	N/A	FAOSTAT data
Service Metric	Gt (dry)	10^15 kcal	10^15 kcal	Gt (dry)	
Oil (EJ/Gt)	0.8	1.2	1.8	0.5	Based on the review by Woods et al. (2010)
Electricity (EJ/Gt)	0.2	1.8	2.7	0.02	Based on the review by Woods et al. (2010)
Oil (Gt/Service Metric)	0.019	0.029	0.026	0.013	(Oil demand)/(Energy density x 2018 production)
Electricity (EJ/Service Metric)	0.2	1.8	1.7	0.02	(Electricity demand)/(2018 production)

Table S82: Estimation of energy use for agriculture and forestry delivery processes. References: (FAO, 2022d, 2022c; Woods et al., 2010)

5.5.2.3 Fertiliser use Only urea-based nitrogen fertiliser is considered as an input to the process (see *Section 5.5.1*). The quantity of fertiliser used in 2018 is taken from the FAOSTAT *Fertiliser Production and Use* domain (FAO, 2022b). 100% of fertiliser used for agriculture in 2018 is assumed to be used in the process for Biomass Agriculture; the coefficient is found by normalising this value (0.1 Gt) by the quantity of plant biomass produced (see *Section 5.5.3*).

5.5.2.4 2018 Production Rates Production rates are mostly derived from FAO data. The database provides the production and supply volumes in terms of calorie content and mass for granular categories (such as *bananas*, *olive oil*, *eggs*, *crustaceans*, and *rice and products*). To estimate the activity demand used here, the database categories have been manually allocated into the product groups needed to derive coefficients for the delivery processes in this model. The breakdown of data into categories of plant biomass and food are shown in Table S83 and Table S84, and the total activity demands for 2018 are shown in Table S85. Spices and alcoholic beverages are also included in plant-based food. They contributed around 2% of food supply calories and 5% mass in 2018.

Biomass type	2018 production, Gt dry bio.	Share of dry mass	Wet:Dry ratio, used to convert data to dry basis	Justification
Rice	0.3	5%	2.4	FAOSTAT Food Balances: ”Production” - ”Other uses (non-food)” - ”Feed”. Individual entries manually assigned to rice. Data is converted to a dry mass basis using the relative weights for food crop commodities, as estimated by Alexander et al. (2017).
Other plant-based food	2.8	51%	2.4	As for rice, considering entries manually assigned to plants.
Fodder	1.7	30%	N/A	Estimated global livestock feed for 2016 excluding pasture from Mottet et al. (2017) Table SI2 and Fig 2. This value excludes crop residues (1.3 Gt).
Biomass for non-food uses (reported)	0.8	14%	1.1	FAOSTAT Food Balances ”Other uses (non-food)”. Only plant matter have been included.
**Total**	**5.6**	**100%**		**Sum of ’consumed’ farmed biomass.**

[a] FAOSTAT food balances data is converted to a dry mass basis using the relative weights for harvested crop commodities or Animal products: Food, as estimated by Alexander et al. (2017).

Table S83: Groups of biomass types accounted for to quanitify total farmed plant biomass in 2018. Data is taken from FAO (2022c).

Food group	2018 food supply (x10^15 kcal)	Share of calorific nutrition
Rice	1.5	18%
Other plant-based food	5.1	63%
Non-ruminant animal products	0.8	11%
Ruminant animal products	0.6	8%
**Total food supply**	**8.0**	**100%**

Table S84: Breakdown of calorific content of food produced in 2018. These values are used to derive coefficients for farming delivery processes. Data is taken from FAO (2022c).

Service	2018 Service Demand	Unit	Source
Farming: Food supply	8.0	x10^15 kcal	FAOSTAT Food Balances
Forestry: Woody biomass	1.8	Gt	FAOSTAT Forestry Production, assuming a density of 458 kg/m3, as used in the 2019 Refinement to the 2006 IPCC Guidelines for NGGI for Sawnwood and Wood Fuel.
Biomass agriculture: Plant biomass	5.6	Gt	Sum of biomass uses within this model. Data fom FAOSTAT Food Balances converted to a dry mass basis using the relative weights for harvested crop commodities, as estimated by Alexander et al. (2017).

Table S85: 2018 Production Rates for the Agriculture and Forestry Sector. Data is taken from FAO (2022c) and FAO (2022d); the breakdown of these values are given in Table S83 and Table S84

5.5.3 Plant Agriculture

This process describes production of plant biomass via agricultural methods. Pasture and use of residues are treated as individual delivery process options (*Sections 5.5.3.6* and *5.5.3.5*), with all other processes quantifying agricultural crop production. The main sources of emissions in plant agriculture are from the use of fertilisers and fuel combustion. Methane from rice paddies is accounted for in the activity for food production (*Section 5.5.4*) since it is dependent on dietary choice.

The delivery processes account for the energy consumed, emissions from use of fertilisers, and emissions from energy consumption (see Figure S37). Land-use change emissions and non-urea based fertilisers are not accounted for, as described in Section 5.5.1.

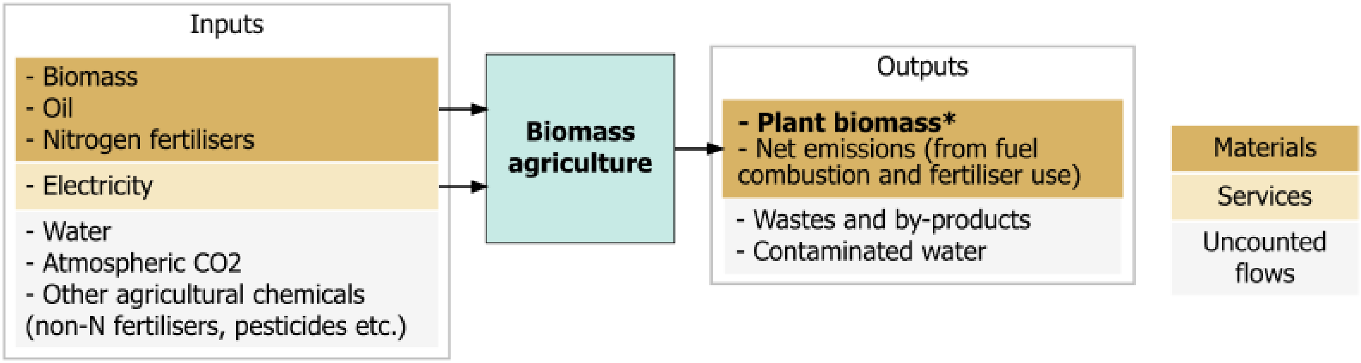

Figure S37: Inputs and outputs for growing plant biomass.

Process	Process Long Name	Description
NETs	Conventional crop agriculture	Conventional agricultural practices
Elec	Electrified	Assume fully electrified agricultural processes to produce biomass
LowFertiliser	Low fertiliser crop agriculture	Use of advanced nutrient management to reduce fertiliser demands (with otherwise conventional practices)
ElecLowFertiliser	Low fertiliser, electrified crop agriculture	Fully electrified process and reduced fertiliser application
Residues	Use of residues	Assume that biomass is sourced from existing residues. Note that there are physical and sustainability limits on the share of this mode.
Pasture	Use of pasture	Assume that pasture requires relatively little human intervention compared with crops (noting that some N2O emissions are assigned to animal rearing rather than to pasture)

Table S86: Delivery processes for this activity

5.5.3.1 Conventional plant agriculture

This delivery process represents crop production, assuming that the current methods and processes are used. The process is represented by the flow diagram in Figure S37. Process coefficients are based on global-level data sources and top-down allocation assumptions, as described in *Section 5.5.2*. This approach has been chosen since specific input demands and emissions would vary significantly with plant type, climatic and weather conditions, levels of irrigation, soil nutrients, and farming practices (amongst others). Much of the detailed data and calculations are given in *Section 5.5.2 - Sector-wide data sources* but key data and assumptions are given below.

Property	Quantity	Unit	Justification
**Summary of key data inputs**			
2018 production of farmed plant biomass (for processes accounted for in the model)	5.61	Gt	FAOSTAT Food Balances
2018 consumption of nitrogen fertilisers in agriculture	0.11	Gt N	FAOSTAT Fertilizers by Nutrient
Quantity of N in Urea	46%	Gt N/ Gt Urea	FAOSTAT Fertilizers by Nutrient
2018 process emissions	1.65	Gt CO2e	Emissions associated crop residues and synthetic fertilisers (FAOSTAT)
**Key assumptions used in calculations**			
Assumed electrical share of energy	30%		Based on IEA data, as described in the Section: Sector Wide Data Sources
Oil demand	0.80	EJ/Gt	Based on IEA data and Woods et al. (2010), as described in the Section: Sector Wide Data Sources
Combustion emissions	0.06	Gt CO2e/Gt	Oil demand x emissions factor (see Model Assumptions Section)
**Calculated coefficients (inputs)**			
Biomass	1.00	Gt biomass/ Gt plant biomass	
Electricity demand	0.20	EJ/Gt	Based on IEA data and Woods et al. (2010), as described in the Section: Sector Wide Data Sources
Oil demand intensity	0.02	Gt/ Gt plant biomass	(oil demand)/ (oil energy density)
Urea demand intensity	0.04	Gt/ Gt plant biomass	(2018 N Fertilizer Consumption)/ (biomass supplied to processes within this model) / (N content of urea)
**Calculated coefficients (outputs)**			
Total emissions intensity	0.30	Gt CO2e/ Gt plant biomass	(process + combustion emissions - sequestered emissions)/ (farmed biomass)

Table S87: Assumptions used to derive coefficients for plant biomass agriculture via conventional processes. Data inputs and assumptions are described in detail in *Section 5.5.2 - Sector-wide data sources*. Other key references: (FAO, 2022c; International Energy Agency, 2021a; Woods et al., 2010); *the SI Part 2.2.3, "Dataset assumptions and approaches"*.

Activity	Process	Resource	Inputs	Outputs	Unit
NonWoodBiomass	NETs	BiomassCapacity	-1.0		Gt/Gt
NonWoodBiomass	NETs	Electricity	-0.20		EJ/Gt
NonWoodBiomass	NETs	Oil	-0.019		Gt/Gt
NonWoodBiomass	NETs	NetEmissions		0.30	Gt_CO2e/Gt
NonWoodBiomass	NETs	Urea	-0.042		Gt/Gt
NonWoodBiomass	NETs	NonWoodBiomass		1.0	Gt/Gt

Table S88: Coefficients for plant biomass agriculture via conventional processes.

5.5.3.2 Electrified plant agriculture

This delivery process represents crop production (Figure S37), assuming that all energy consuming processes can be electrified. This implies that all energy is provided by electricity, and there are zero combustion emissions. The coefficients are estimated from the conventional coefficients, described in Section 5.5.3.1. It is assumed that the operation efficiency does not change with electrification - this is unlikely to be the case but it is hard to quantify the direction of the change without a better understanding of the processes involved.

Activity	Process	Resource	Inputs	Outputs	Unit
NonWoodBiomass	Elec	BiomassCapacity	-1.0		Gt/Gt
NonWoodBiomass	Elec	Electricity	-1.0		EJ/Gt
NonWoodBiomass	Elec	NetEmissions		0.29	Gt_CO2e/Gt
NonWoodBiomass	Elec	Urea	-0.042		Gt/Gt
NonWoodBiomass	Elec	NonWoodBiomass		1.0	Gt/Gt

Table S89: Coefficients for plant biomass agriculture via electrified processes.

5.5.3.3 Conventional plant agriculture with reduced fertiliser use

This delivery process represents crop production (Figure S37), assuming that conventional energy sources are used within agriculture but fertiliser use and associated emissions can be reduced without yield losses by changing farming practices, and by using nitrogen inhibitors. The coefficients are derived from the conventional coefficients, described in Section 5.5.3.1, with a reduction in fertiliser use.

The reduction in fertiliser demand is based on the analysis of Gao & Cabrera Serrenho (2023), which assumes that global nitrogen use efficiency can increase from 42% today to 67%. This would imply an approximately 30% reduction in demand for fertilisers. This is broadly similar to the conclusions of Jensen et al. (2020) who found that demand could be reduced by 25% from the conventional delivery process using methods such as intercropping. Following the approach by Gao & Cabrera Serrenho (2023), it is also assumed that the rate of emissions associated with fertiliser use can be reduced by around 50% by using nitrogen inhibitors. This leads to an overall reduction in emissions of 1 - (1 - 30%) x 50% = 65%.

Activity	Process	Resource	Inputs	Outputs	Unit
NonWoodBiomass	LowFertiliser	BiomassCapacity	-1.0		Gt/Gt
NonWoodBiomass	LowFertiliser	Electricity	-0.20		EJ/Gt
NonWoodBiomass	LowFertiliser	Oil	-0.019		Gt/Gt
NonWoodBiomass	LowFertiliser	NetEmissions		0.11	Gt_CO2e/Gt
NonWoodBiomass	LowFertiliser	Urea	-0.029		Gt/Gt
NonWoodBiomass	LowFertiliser	NonWoodBiomass		1.0	Gt/Gt

Table S90: Coefficients for plant biomass agriculture with reduced fertiliser application.

5.5.3.4 Electrified plant agriculture with reduced fertiliser use

This delivery process represents crop production (Figure S37), assuming that: (1) All energy consuming processes can be electrified such that all energy is provided by electricity without a change in efficiency (Section 5.5.3.2); and (2) Fertiliser use and associated emissions are significantly reduced, without reducing yields, as assumed for the Low Fertiliser process (Section 5.5.3.3). The coefficients are estimated from the conventional coefficients (Section 5.5.3.1).

Activity	Process	Resource	Inputs	Outputs	Unit
NonWoodBiomass	ElecLowFertiliser	BiomassCapacity	-1.0		Gt/Gt
NonWoodBiomass	ElecLowFertiliser	Electricity	-1.0		EJ/Gt
NonWoodBiomass	ElecLowFertiliser	NetEmissions		0.10	Gt_CO2e/Gt
NonWoodBiomass	ElecLowFertiliser	Urea	-0.029		Gt/Gt
NonWoodBiomass	ElecLowFertiliser	NonWoodBiomass		1.0	Gt/Gt

Table S91: Coefficients for plant biomass agriculture via electrified processes with reduced fertiliser application.

5.5.3.5 Agricultural residue collection

This process describes the recovery of agricultural residues for downstream use in place of agricultural crops (Figure S38). All energy and emissions associated with growing agricultural products are associated with the harvested biomass (represented by the processes described in Sections 5.5.3.1 to 5.5.3.4). For simplicity, no additional energy has been attributed to the process of collecting residues. The proportion of *NonWoodBiomass* supplied by this delivery process must be limited to within the maximum range of residues produced by agriculture. The maximum sustainable use of agricultural residues is approximately 0.45:1 (mass residues:mass harvested product)[1].

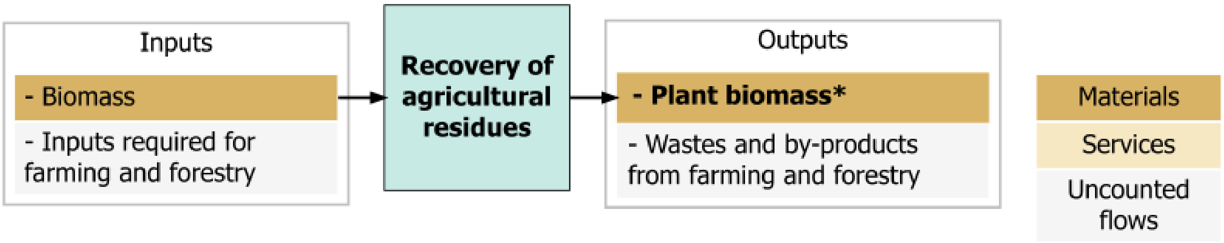

Figure S38: Inputs and outputs for residue production and recovery from agriculture. In the absence of data, all energy associated with production is accounted for in the processes for *NonWoodBiomass* (Section 5.5.3), and it is assumed that energy and emissions associated with residue recovery would be relatively small.

Activity	Process	Resource	Inputs	Outputs	Unit
NonWoodBiomass	Residues	BiomassCapacity	-1.0		Gt/Gt
NonWoodBiomass	Residues	NonWoodBiomass		1.0	Gt/Gt

Table S92: Coefficients for plant biomass production by collecting residues. In the absence of data, no additional energy has been attributed to the process of collecting residues. This process represents consumption of biomass resources without energy or emissions - the proportion of NonWoodBiomass supplied by this process is therefore limited, as described above.

[1]This is based on an assumed residue:crop ratio of 0.8, consistent with 2009 global agricultural flows estimated by Bajželj et al. (2014), and that 45% of this residue must be left in the field to maintain ecological functions (based on the relative shares of ecological and theoretical potentials for residue use calculated by Daioglou et al. (2016).

5.5.3.6 Growing pasture

This process describes the production of pasture (rather than farmed feedstock) to feed livestock (Figure S39). In the absence of data no energy-use is associated with growing and maintaining pasture directly. It is assumed this would be relatively small compared to that used for crop production. The proportion of *NonWoodBiomass* supplied by this delivery process must be limited to within a plausible range. Lower overall use of biomass suggests a larger percentage of pasture may be possible (since pasture availability will be limited by land availability). Around 30% of biomass currently appropriated by humans is from pasture (on a mass basis, as estimated in the supply availability SI document). The coefficients are given in Table S93.

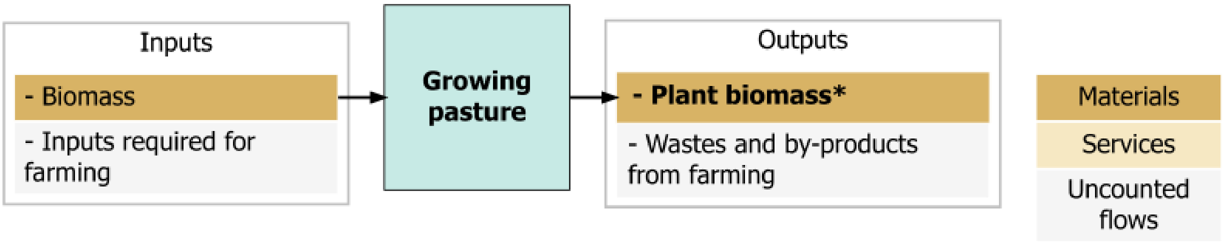

Figure S39: Inputs and outputs for growing pasture. In the absence of data, no energy-use is associated with growing pasture directly. It is assumed this would be relatively small compared to crop production.

Activity	Process	Resource	Inputs	Outputs	Unit
NonWoodBiomass	Pasture	BiomassCapacity	-1.0		Gt/Gt
NonWoodBiomass	Pasture	NonWoodBiomass		1.0	Gt/Gt

Table S93: Coefficients for growing pasture to produce plant biomass. In the absence of data, no additional energy has been attributed to the process of growing pasture. This process therefore represents consumption of biomass resources without energy or emissions. The proportion of *NonWoodBiomass* which can be supplied by this process is limited by land-use.

5.5.4 Farming Food

Farming food describes the production of raw food (including meat, animal products, vegetables, pulses and grains etc) which is considered an input to food processing (Section 5.7). Farming food is treated separately to growing *Plant Biomass* (Section 5.5.3) so that all food types can be considered within one substance. *Plant Biomass* is an input to the activity (Figure S40) which is used either as the food directly, for plant-based foods, or as fodder for livestock. Each delivery process considers production of a subset of all food (animal products, ruminant animal products or plant-based food) so that the impact of dietary choice can be explored. Novel farming practices, such as use of feed-additives to reduce emissions from ruminants can also be included. The delivery processes currently included in the model are given in Table S94.

The flow diagram for all delivery processes is given in Figure S40. The delivery processes account for the energy consumed, combustion emissions from fuel use, and process emissions (from livestock, livestock wastes, soils and rice paddies, as shown in Table S81, Section 5.5.2). Land-use change emissions are not accounted for, as described in Section 5.5.1. The processes each account for energy and emissions from growing biomass (either for the food itself, or as fodder for livestock) via the input value for farmed biomass (from Section 5.5.3).

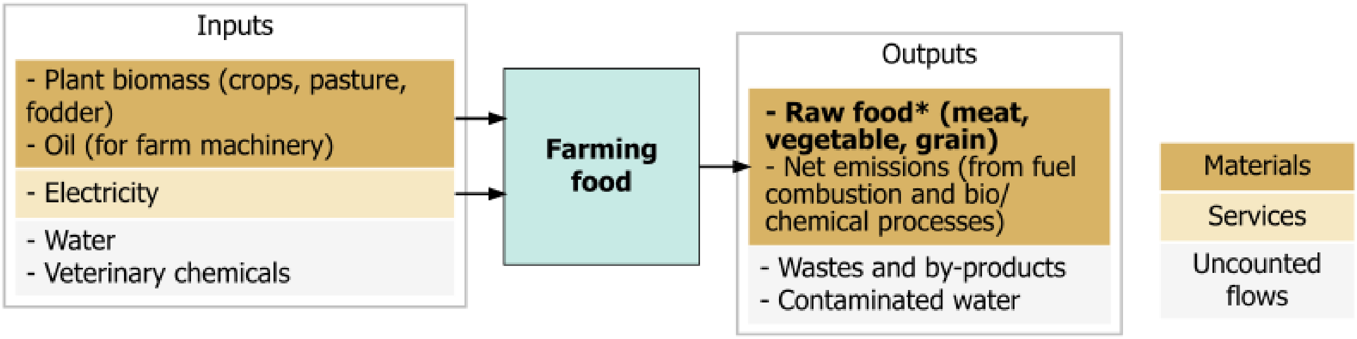

Figure S40: Inputs and outputs for the activity, farming food. Energy related to growing biomass, either crops or for use as animal feed are not accounted for here but in the processes for *NonWoodBiomass* (Section 5.5.3).

Process	Process Long Name	Description
PlantBasedFood	Plant-based food only agriculture	Plant-based food only agriculture: 100% plant products
PlantFoodLowMethaneRice	Plant-based food only agriculture with low-methane rice	100% plant products with practices for low-methane rice
PlantFoodNoRice	Plant-based food only agriculture without rice farming (increasing the share of this mode decreases the proportion of rice in the average global diet)	100% plant products with practices for low-methane rice
NonRuminantMeat	Animal agriculture, non-ruminant	100% animal products (meat, eggs etc) but no red meat nor dairy
RuminantMeat	Ruminant only agriculture	100% animal products (meat, eggs, dairy etc) from ruminant animals only
RuminantFeedAdditives	Ruminant only agriculture with feed additives	100% ruminant products using feed additives to reduce methane production

Table S94: Delivery processes for this activity

5.5.4.1 Provision of plant-based food

This delivery process (Figure S41) represents raw food production, assuming that all food is plant-based and is grown using the same methods and processes, and under the same conditions, as in 2018. Process coefficients are based on global-level data sources and top-down allocation assumptions, as described in *Section 5.5.2*. This approach has been chosen since specific input demands would vary significantly with farming practices and the specific food being produced.

Consumption of energy and emissions from fertiliser use are accounted for in the process for *Biomass Agriculture* (Section 5.5.3) which is an input to this process. Methane emissions from rice farming are accounted for in this process. Much of the detailed data and calculations are given in *Section 5.5.2 - Sector-wide data sources* but key data and assumptions used to derive the coefficients are summarised in Table S95 and the final values in Table S96.

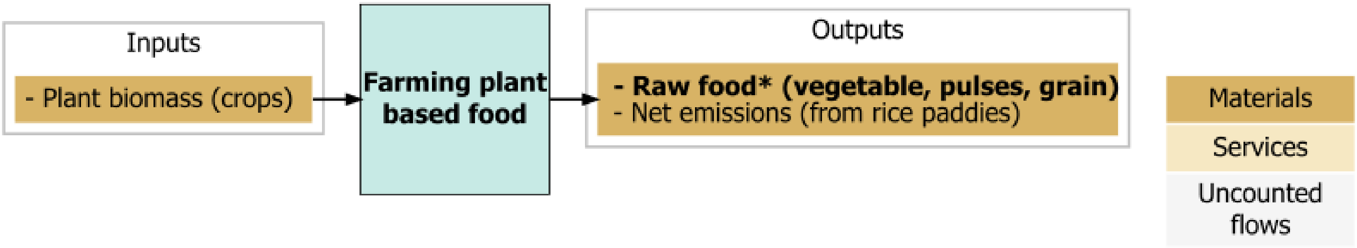

Figure S41: Inputs and outputs for the provision of plant-based food. Note that energy and emissions associated with agriculture of generic plant biomass is accounted for in the delivery processes for Plant Agriculture, (Section 5.5.3)

Property	Quantity	Unit	Justification
**Summary of key data inputs**			
2018 supply of plant foods (Calories)	6.5	10^15 kcal	FAOSTAT Food Balances
2019 production of plant foods (mass basis)	3.1	Gt	FAOSTAT Food Balances
2018 process emissions	1.02	Gt CO2e	FAOSTAT. Emissions from rice cultivation only, other emissions associated with plant agriculture is accounted for in NonWoodBiomass
**Calculated coefficients (inputs)**			
Energy demand	0	EJ	Biomass Agriculture accounts for energy use in plant-based farming
Biomass demand intensity	0.47	Gt/ 10^15 kcal	(mass of plant food)/(calories of plant food). Note that biomass yield losses are accounted for in Biomass Agriculture.
**Calculated coefficients (outputs)**			
Total emissions intensity	0.16	Gt CO2e/ 10^15 kcal	(process + combustion emissions)/ (kcal from plant-based foods)

Table S95: Assumptions used to derive coefficients for provision of plant-based food. Data inputs and assumptions are described in detail in *Section 5.5.2 - Sector-wide data sources*. Other key references: (FAO, 2022c; International Energy Agency, 2021a; Woods et al., 2010); *the SI Part 2.2.3, "Dataset assumptions and approaches"*.

Activity	Process	Resource	Inputs	Outputs	Unit
RawFood	PlantBasedFood	NonWoodBiomass	**-0.47**		Gt/10^15kcal
RawFood	PlantBasedFood	NetEmissions		**0.16**	Gt_CO2e/10^15kcal
RawFood	PlantBasedFood	RawFood		**1.0**	10^15kcal/10^15kcal

Table S96: Coefficients for provision of plant-based food.

5.5.4.2 Provision of plant-based food with low methane rice

This delivery process represents raw food production, assuming a change in farming practice for rice production. The process is represented by the same diagram used in Figure S41, since the only change from conventional plant based food production is a reduction in the quantity of methane produced.

The use of *alternate wetting and drying* can reduce methane emissions in rice cultivation by more than 50% according to various studies reviewed by Adhya et al. (2014). Methane emissions from rice cultiviation occur due to the wet anaerobic environment used in traditional methods of rice cultivation. If rice continues to be a large proportion of global diets (or increases, given population growth in asian countries), methods to mitigate these emissions will be important. Adhya et al. (2014) reported techniques (such as *dry-seeding*) that could reduce emissions by up to 90% but the lower estimate of 50% is used here since there is considerable uncertainty in the estimates and there may be increased nitrous oxide. Other techniques include mid-season drainage, reported to reduce emissions by around 35-40% (Griscom et al., 2017).

The coefficients are based on those for conventional plant-based food farming described in Table S95 but with only 50% of the emissions from rice cultivation. Coefficients are given in Table S97.

Activity	Process	Resource	Inputs	Outputs	Unit
RawFood	PlantFoodLowMethaneRice	NonWoodBiomass	**-0.47**		Gt/10^15kcal
RawFood	PlantFoodLowMethaneRice	NetEmissions		**0.078**	Gt_CO2e/10^15kcal
RawFood	PlantFoodLowMethaneRice	RawFood		**1.0**	10^15kcal/10^15kcal

Table S97: Coefficients for provision of plant-based food with low methane rice.

5.5.4.3 Provision of plant-based food without rice

This delivery process represents raw food production, assuming that we stop farming rice to avoid the methane emissions inherent in farming in the wet anaerobic environments needed for rice cultivation. It's not clear whether there are alternative crops that could be grown on land currently used for rice cultivation. The share of this process may be limited while still providing sufficient food, given rice is an important staple crop throughout much of the world.

The coefficients are based on those derived for plant-based food (Section 5.5.4.1), excluding the emissions associated with rice farming.

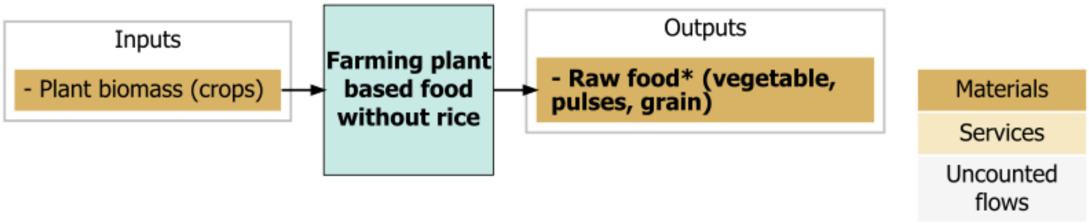

Figure S42: Inputs and outputs for the provision of plant-based food without rice. Note that energy and emissions associated with agriculture of generic plant biomass is accounted for in the delivery processes for Plant Agriculture, (Section 5.5.3.)

Activity	Process	Resource	Inputs	Outputs	Unit
RawFood	PlantFoodNoRice	NonWoodBiomass	**-0.47**		Gt/10^15kcal
RawFood	PlantFoodNoRice	NetEmissions	**-0.00000010**		Gt_CO2e/10^15kcal
RawFood	PlantFoodNoRice	RawFood		**1.0**	10^15kcal/10^15kcal

Table S98: Coefficients for provision of plant-based food without rice.

5.5.4.4 Provision of animal products (non-ruminant)

This delivery process represents raw food production, assuming that all food is derived from non-ruminant livestock (i.e. poultry, pork, eggs etc), farmed using the same methods and processes, and under the same conditions, as in 2018. Resource use for the production of fodder is accounted for in the process for *Biomass Agriculture* (Section 5.5.3) which is an input to this process. Much of the detailed data and calculations are given in *Section 5.5.2 - Sector-wide data sources* but a summary of the key data and assumptions used to derive the coefficients is given below.

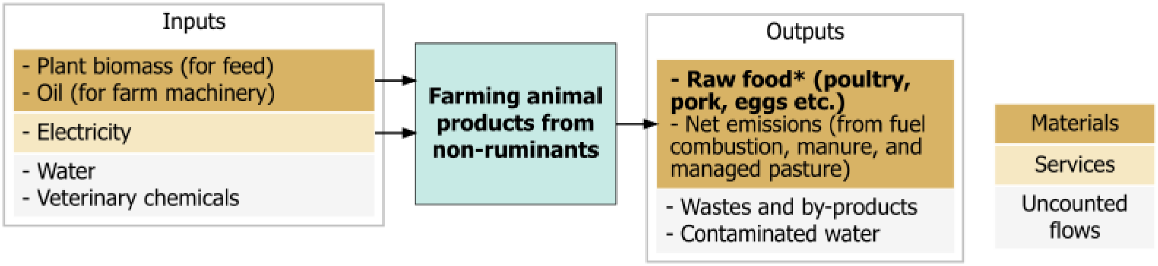

Figure S43: Inputs and outputs for the provision of non-ruminant animal products.

Property	Quantity	Unit	Justification
**Summary of key data inputs and assumptions**			
2018 supply	0.85	10^15 kcal	FAOSTAT Food Balances "Food" (manually allocated to non-ruminant animal products)
2018 supply	0.52	Gt	FAOSTAT Food Balances
Feed demand	1.1	Gt	Estimated global monogastric feed for 2016 from Mottet et al. (2017) Table SI2 and Fig 2.
2018 process emissions	0.35	Gt CO2e	50% emissions associated with manure (Section: Sector Wide Data Sources)
Electricity demand	2.70	MJ/kg	Based on IEA data and Woods et al. (2010), as described in the Section: Sector Wide Data Sources
Oil demand	1.80	MJ/kg	Based on IEA data and Woods et al. (2010), as described in the Section: Sector Wide Data Sources
Combustion emissions	0.13	kg CO2/1000 kcal	Oil demand x emissions factor (see Model Assumptions Section)
**Calculated coefficients (inputs)**			
Feed	1.36	kg/1000 kcal	(total attributed feed)/ (kcal from non-ruminant products)
Electricity	1.66	MJ/1000 kcal	(elec demand) x (mass/ kcal from non-ruminant products)
Oil	0.03	kg/1000 kcal	(oil demand) x (mass/ kcal from non-ruminant products) / oil energy density
**Calculated coefficients (outputs)**			
Total emissions	0.55	kg CO2e/1000 kcal	combustion emissions + process emissions/non-ruminant kcal production)

Table S99: Assumptions used to derive coefficients for provision of food from non-ruminant animals. Data inputs and assumptions are described in detail in *Section 5.5.2 - Sector-wide data sources.* Other key references: (FAO, 2022c; International Energy Agency, 2021a; Mottet et al., 2017; Woods et al., 2010); *the SI Part 2.2.3, "Dataset assumptions and approaches".*

Activity	Process	Resource	Inputs	Outputs	Unit
RawFood	NonRuminantMeat	NonWoodBiomass	-1.4		Gt/10^15kcal
RawFood	NonRuminantMeat	Electricity	-1.7		EJ/10^15kcal
RawFood	NonRuminantMeat	Oil	-0.026		Gt/10^15kcal
RawFood	NonRuminantMeat	NetEmissions		0.55	Gt_CO2e/10^15kcal
RawFood	NonRuminantMeat	RawFood		1.0	10^15kcal/10^15kcal

Table S100: Coefficients for provision of food from non-ruminant animals.

5.5.4.5 Provision of ruminant animal products (beef and dairy etc)

This delivery process represents raw food production, assuming that all food is derived from ruminant livestock, farmed using the same methods and processes, and under the same conditions, as in 2018. Resource use for the production of fodder is accounted for in the process for *Biomass Agriculture* (Section 5.5.3) which is an input to this process. Much of the detailed data and calculations are given in *Section 5.5.2 - Sector-wide data sources* but a summary of the key data and assumptions used to derive the coefficients is given below.

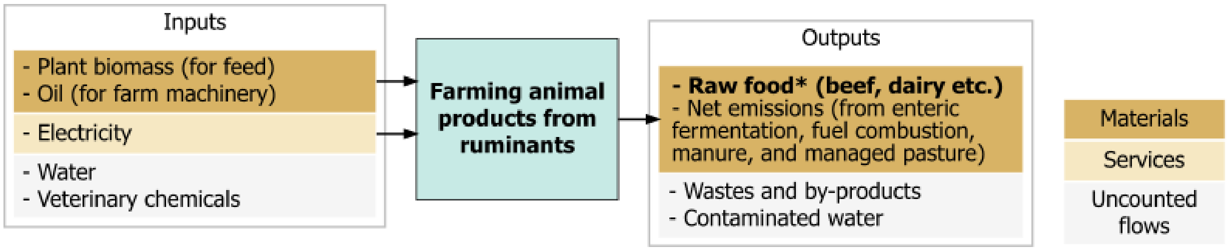

Figure S44: Inputs and outputs for the provision of ruminant animal products.

Property	Quantity	Unit	Justification
**Summary of key data inputs and assumptions**			
2018 supply	0.63	10^15 kcal	FAOSTAT Food Balances ”Food” (manually allocated to non-ruminant animal products)
2018 supply	0.63	Gt	FAOSTAT Food Balances
2018 process emissions	3.3	Gt CO2e	Described in Section: Sector Wide Data Sources
Feed demand	4.85	Gt	Estimated total global ruminent feed for 2016 from Mottet et al. (2017) Table SI2 and Fig 2. This value includes feed, pasture, residues and by-products.
Electricity demand	1.80	EJ/Gt	Based on IEA data and Woods et al. (2010), as described in the Section: Sector Wide Data Sources
Oil demand	1.20	EJ/Gt	Based on IEA data and Woods et al. (2010), as described in the Section: Sector Wide Data Sources
Combustion emissions	0.08	Gt CO2e/ 10^15 kcal	Oil demand x emissions factor (see Model Assumptions Section)
**Calculated coefficients (inputs)**			
Feed	7.73	Gt/ 10^15 kcal	(total attributed feed)/ (kcal from non-ruminant products)
Electricity	1.81	EJ/ 10^15 kcal	(elec demand) x (mass/ kcal from non-ruminant products)
Oil	0.03	Gt/ 10^15 kcal	(oil demand) x (mass/ kcal from non-ruminant products) / oil energy density
**Calculated coefficients (outputs)**			
Total emissions	5.31	Gt CO2e/ 10^15 kcal	combustion emissions + process emissions/non-ruminant kcal production)

Table S101: Assumptions used to derive coefficients for provision of food from non-ruminant animals. Data inputs and assumptions are described in detail in *Section 5.5.2 - Sector-wide data sources.* Other key references: (FAO, 2022c; International Energy Agency, 2021a; Mottet et al., 2017; Woods et al., 2010); *the SI Part 2.2.3, “Dataset assumptions and approaches”.*

Activity	Process	Resource	Inputs	Outputs	Unit
RawFood	RuminantMeat	NonWoodBiomass	-7.7		Gt/10^15kcal
RawFood	RuminantMeat	Electricity	-1.8		EJ/10^15kcal
RawFood	RuminantMeat	Oil	-0.029		Gt/10^15kcal
RawFood	RuminantMeat	NetEmissions		5.3	Gt_CO2e/10^15kcal
RawFood	RuminantMeat	RawFood		1.0	10^15kcal/10^15kcal

Table S102: Coefficients for provision of ruminant animal products.

5.5.4.6 Provision of ruminant animal products using feed additives

This delivery process represents raw food production, assuming that all food is derived from ruminant livestock but where livestock are fed a seaweed-based feed additive to reduce enteric methane production. These emissions are "a natural by-product of microbial fermentation of nutrients in the digestive tract of animals", and can be affected by the composition of livestock feed (Roque et al., 2019). The process is represented by the same diagram used in Figure S44, since the only change from conventional ruminant production is a reduction in the quantity of methane produced.

The coefficients are based on the same calculations used to estimate coefficients for conventional ruminant animal products (Section 5.5.4.5) but with 50% lower emissions from enteric fermentation, consistent with the findings of a study by Roque et al. (2019) using seaweed type feed additives (at 1% to cattle). This result is at the more effective end of the ranges from previous studies which mostly demonstrated reductions from 2 to 40% (although one study gave +27%). Feed demand is assumed to remain unchanged since the yield of meat from feed was found to be slightly higher with additives while the yield of milk decreased.

Activity	Process	Resource	Inputs	Outputs	Unit
RawFood	RuminantFeedAdditives	NonWoodBiomass	**-7.7**		Gt/10^15kcal
RawFood	RuminantFeedAdditives	Electricity	**-1.8**		EJ/10^15kcal
RawFood	RuminantFeedAdditives	Oil	**-0.029**		Gt/10^15kcal
RawFood	RuminantFeedAdditives	NetEmissions		**2.9**	Gt_CO2e/10^15kcal
RawFood	RuminantFeedAdditives	RawFood		**1.0**	10^15kcal/10^15kcal

Table S103: Coefficients for provision of food derived from ruminant animal products using feed additives.

5.5.5 Forestry

Forestry covers the activity to produce woody biomass from forests for use in other processes. The process accounts for the energy consumed, and emissions from energy consumption (see Figure S45). Land-use change emissions are not accounted for, as described in Section 5.5.1.

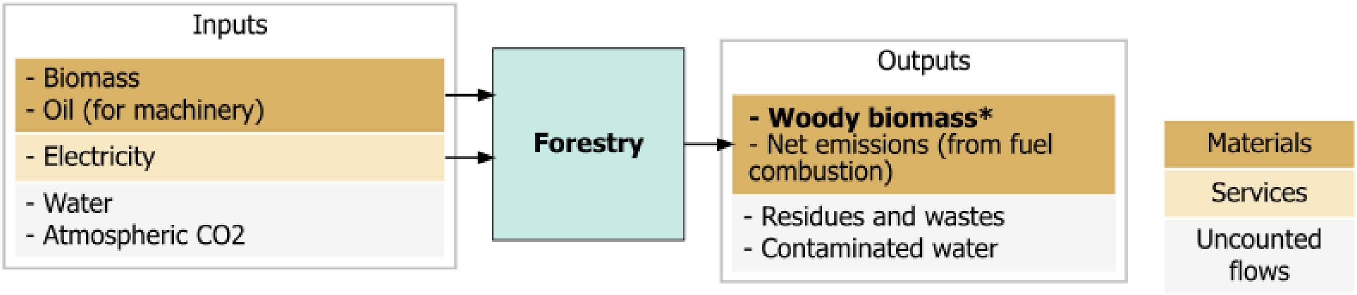

Figure S45: Inputs and outputs for the activity, forestry.

Process	Process Long Name	Description
NETs	Conventional	Using conventional fuels to gather woody biomass from forests (logging and forest residue collection)
Elec	Electrified	Assume fully electrified processes to gather woody biomass

Table S104: Delivery processes for this activity

5.5.5.1 Conventional forestry

This delivery process represents production of woody biomass from forestry, assuming current methods and processes. Process coefficients are based on global-level data sources and top-down allocation assumptions, as described in *Section 5.5.2*. This approach has been chosen since specific input demands would vary significantly with forest type, maturity levels, local conditions and management approach. Much of the detailed data and calculations are given in *Section 5.5.2 - Sector-wide data sources* but a summary of the key data and assumptions used to derive the coefficients is given below.

Energy use is calculated as the residual unaccounted energy use in agriculture forestry and fishing in 2018 from IEA Summary Energy Balances (2021a), after agriculture has been accounted for, as explained in Section 5.5.2. It is assumed that for agriculture and forestry overall, 40% energy is supplied as electricity and 60% as oil - this is a simplification of 2018 energy consumption where 53% was supplied as oil products, 30% as electricity and heat, and other fuels supplying the residual.

Property	Quantity	Unit	Justification
**Summary of key data inputs and assumptions**			
2018 production of roundwood	4014	million cubic meter	FAOSTAT Forestry Production and Trade
Assumed roundwood density	458	kg/m3 = kt/ mill m3	2019 Refinement to the 2006 IPCC Guidelines for NGGI
2018 production (mass terms)	1.8	Gt	(volume produced) x (density)
Oil demand	0.55	EJ/Gt	Based on IEA data and Woods et al. (2010), as described in the Section: Sector Wide Data Sources
**Calculated coefficients (inputs)**			
Electricity demand	0.02	EJ/Gt	Based on IEA data and Woods et al. (2010), as described in the Section: Sector Wide Data Sources
Oil demand intensity	0.01	Gt/ Gt	(oil demand) / (oil energy density from Model Assumptions Section)
**Calculated coefficients (outputs)**			
Combustion emissions	0.04	Gt CO2e/Gt	(oil demand) x (emissions factor from Model Assumptions Section)

Table S105: Assumptions used to derive coefficients for forestry via conventional processes. Data inputs and assumptions are described in detail in *Section 5.5.2 - Sector-wide data sources*. Other key references: (FAO, 2022d; International Energy Agency, 2021a); *the SI Part 2.2.3, "Dataset assumptions and approaches"*.

Activity	Process	Resource	Inputs	Outputs	Unit
Wood	NETs	BiomassCapacity	**-1.0**		Gt/Gt
Wood	NETs	Electricity	**-0.018**		EJ/Gt
Wood	NETs	Oil	**-0.013**		Gt/Gt
Wood	NETs	NetEmissions		**0.038**	Gt_CO2e/Gt
Wood	NETs	Wood		**1.0**	Gt/Gt

Table S106: Coefficients for forestry via conventional processes.

5.5.5.2 Electrification of forestry

This process assumes that all energy consuming processes within forestry can be electrified such that all energy is provided by electricity and there are zero combustion emissions. The coefficients are estimated from the conventional coefficients, described in Section 5.5.5.1.

Activity	Process	Resource	Inputs	Outputs	Unit
Wood	Elec	BiomassCapacity	**-1.0**		Gt/Gt
Wood	Elec	Electricity	**-0.56**		EJ/Gt
Wood	Elec	NetEmissions	**-0.00000010**		Gt_CO2e/Gt
Wood	Elec	Wood		**1.0**	Gt/Gt

Table S106: Coefficients for forestry via conventional processes.

5.5.6 Agriculture and Forestry Coefficient Summary

ResourceFlow	NonWoodBiomass__Residues	NonWoodBiomass__Pasture	NonWoodBiomass__NETs	NonWoodBiomass__Elec	NonWoodBiomass__LowFertiliser	NonWoodBiomass__ElecLowFertiliser	RawFood__PlantBasedFood	RawFood__PlantFoodLowMethaneRice	RawFood__PlantFoodNoRice	RawFood__NonRuminantMeat	RawFood__RuminantMeat	RawFood__RuminantFeedAdditives	Wood__NETs	Wood__Elec
BiomassCapacity, Gt	-1.0	-1.0	-1.0	-1.0	-1.0	-1.0							-1.0	-1.0
NonWoodBiomass, Gt	1.0	1.0	1.0	1.0	1.0	1.0	-0.47	-0.47	-0.47	-1.4	-7.7	-7.7		
Electricity, EJ			-0.20	-1.0	-0.20	-1.0				-1.7	-1.8	-1.8	-0.018	-0.56
Oil, Gt			-0.019		-0.019					-0.026	-0.029	-0.029	-0.013	
NetEmissions, Gt_CO2e			0.30	0.29	0.11	0.10	0.16	0.078	-0.00000010	0.55	5.3	2.9	0.038	-0.00000010
Urea, Gt			-0.042	-0.042	-0.029	-0.029								
RawFood, 10^15kcal							1.0	1.0	1.0	1.0	1.0	1.0		
Wood, Gt													1.0	1.0

Table S107: Summary of coefficients for the Agriculture and Forestry sector.

5.6 Chemicals, Chemical Products and Fuels

This section outlines the derivation of coefficients for producing resources and substances currently produced in the petrochemical sector, and those which would replace petrochemical products (as represented in Figure S46 and listed in Table S108). The production processes in this section do not account for the emissions captured or released downstream (at the "user" level) or far upstream (e.g. in production of biomass). Those emissions are accounted for in other sectors, extraction of raw fossil fuels, for example, is covered in Mining and Extraction (Section 5.3), while emissions arising from fertiliser application is covered in Agriculture and Forestry (Section 5.5). Emissions from electricity production is accounted for in the coefficients for electricity generation (Section 5.4).

The petrochemcical industry is an important component of modern society; it is one of the largest sources of industrial emissions and consumption of energy but modern society is dependent on its wide range of products. The variety of products and complexity of the process routes make it difficult to analyse. The high inter-linkage and multi-step processes are easily evident in the work of Levi & Cullen (2018) which maps global flows of chemicals (from feedstocks to products), using a combination of top-down and bottom-up methods. They do not include secondary production routes (based on recyclates), nor alternative feedstocks (non-fossil fuel).

The approach taken here is to group conventional petrochemical products into three blocks of primary chemicals (HVCs, Ammonia, Methanol) and three blocks of downstream production (polymers, fertilisers, and other), consistent with the groupings used by the International Energy Agency (2018). These groupings were mapped onto the global chemical mass flows compiled by Levi & Cullen (2018) to estimate aggregated mass flows. The reason to group the industry in this way is well-justified by the International Energy Agency (2018) in terms of primary chemical production energy and emissions:

> Primary chemicals account for around 2/3 energy consumption and 60% of total CO_2 emissions in the chemical sector; ammonia production contributes ~50% of primary chemicals emissions, while high-value chemicals (HVCs) and methanol each contribute ~25% (International Energy Agency, 2018).

Additional activities in this section cover the production of feedstocks for primary chemicals (e.g. Hydrogen), and processing of fossil fuels and 'low-carbon' fuels (e.g. biofuels and synthetic fuels which have the potential to replace conventional petrochemicals). The activities within this category are shown in Figure S46 and listed in Table S108.

Sector	Activity Long Name	Produces
FuelsFeedstocks	Ammonia Production	Ammonia
FuelsFeedstocks	Biofuel Production	BioFuel
FuelsFeedstocks	HVCs Production	High Value Chemicals
FuelsFeedstocks	Hydrogen Production	Hydrogen
FuelsFeedstocks	Methane Production	Methane
FuelsFeedstocks	Methanol Production	Methanol
FuelsFeedstocks	Oil Processing and Refining	Oil
FuelsFeedstocks	Plastics Production - end-use quantity excludes plastics for fabrics	Plastics
FuelsFeedstocks	Production of other petrochemical products, not accounted for elsewhere	Other Petrochemical Products
FuelsFeedstocks	Synfuel Production	Synfuel
FuelsFeedstocks	Urea Production	Urea

Table S108: Activities within this chapter

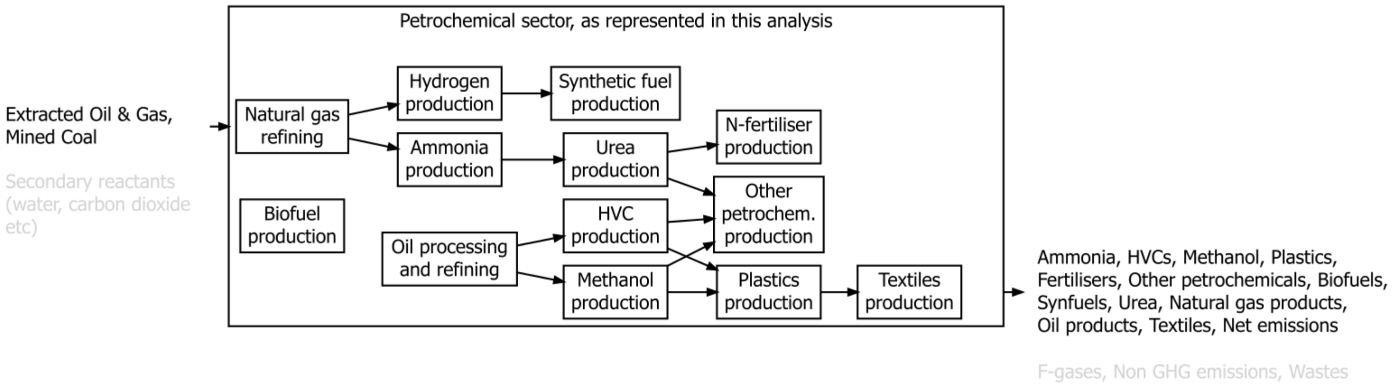

Figure S46: Processes and material flows considered within this section. All modelled activity processes are represented by boxes and key material flows marked by arrows. Not all possible flows are shown here to simplify the diagram (for instance, natural gas is an input to many processes but these flows have not been drawn).

5.6.1 Overarching Sector Assumptions

Best Practice Technologies (BPT) are assumed

The coefficients in this section are mostly based on values for BPT (Best Practice Technologies), as defined by Saygin et al. (2009) [2]. High levels of energy integration and heat cascading, and Combined Heat and Power (CHP) are not accounted for in the values used in that analysis. For the level of accuracy and granularity here, developments in technology since the source publication are assumed to be negligible.

Steam consumption and exports

The data sources include negative values to account for steam exports (excess steam) from production processes with exothermic reactions; in this analysis these are treated as providing an energy saving to the fuel requirements stated. The reduction in fuel to account for steam exports is estimated by assuming that 90% steam generation efficiency from fuel (as used by Levi & Cullen (2018) for natural gas conversion to steam), and that 80% of exported steam can be used by another process. The reduction in fuel is therefore:

$$Fuel\ Reduction\ =\ \frac{0.8\ \times\ Steam\ Energy}{0.9}$$

.

Where different fuels are used to generate steam (e.g. for some plastics production processes), the steam generation efficiency is adjusted to represent the efficiency of the relevant technology.

Similarly, additional fuel demand to account for steam use in any given process is estimated as:

$$Additional\ Fuel\ =\ \frac{Steam\ Energy}{0.9}$$

[2] Following the approach of Saygin et al. (2009), "BPT represents the most advanced technologies that are currently in use at industrial scale", in contrast to best available techniques, BAT, which may not be viable at large-scale. In some cases, they could be the same.

5.6.2 Sector-wide Data Sources

The key references used to derive coefficients for multiple activities in this chapter are outlined in Table S109. Additional references may be used for specific activities and processes, and are detailed in the relevant sections.

Reference	Title	Used for:
(Levi & Cullen, 2018)	Mapping Global Flows of Chemicals: From Fossil Fuel Feedstocks to Chemical Products	Understanding process routes, defining simplified chemical groupings, and approximation of relative proportions of chemicals within aggregated groups (for scaling process energy intensities)
(International Energy Agency, 2018)	The Future of Petrochemicals	Overall industry understanding, choice of alternative production routes, main basis for estimation of coefficients based on tabulated values for fuel/steam/electricity demand on a process basis (within SI document).
(Saygin & Gielen, 2021)	Zero-Emission Pathway for the Global Chemical and Petrochemical Sector	Feedstock estimation, fuel/steam/electricity demand on a product basis – conventional routes only
(Saygin et al., 2009)	IEA Information Paper: Chemical and Petrochemical Sector	Feedstock estimation, fuel/steam/electricity demand on a product basis – conventional and alternative routes

Table S109: Key references used to derive coefficients for petrochemical industry production processes

5.6.3 Oil Processing and Refining

This activity describes downstream processing and refining to produce oil products from feedstocks. All oil products are currently treated as one substance, with only one delivery process representing current technologies and processes.

5.6.3.1 Conventional oil processing and refining

This process describes downstream processing of fossil fuel feedstocks from the oil and gas extraction process (see Section 5.3) into oil products, using conventional technologies and processes. The coefficients are derived from the energy and emissions intensity of emissions extraction in 2018. As described in Section 5.3.4, extracted oil and gas is currently treated in the model as one combined substance, consistent with data sources for global oil and gas extraction. Oil and natural gas are often found in and extracted from the same site. Petroleum Coke, Refinery Gas and LPG have been included here within the substance flow *ExtractedOilGas*

Hydrogen is used in oil refineries to "remove impurities and upgrade heavy oil fractions"(IRENA, 2022). According to a report by IRENA (2022), around 40 Mt Hydrogen was used for this purpose in 2022. This values is divided by 2018 production volume (4.25 Gt, as shown in Table S110) to estimate the coefficient for hydrogen demand associated with oil refineries.

Fugitive emissions from fossil fuel processing are not considered here; they are included in the extraction of fuels (Section 5.3).

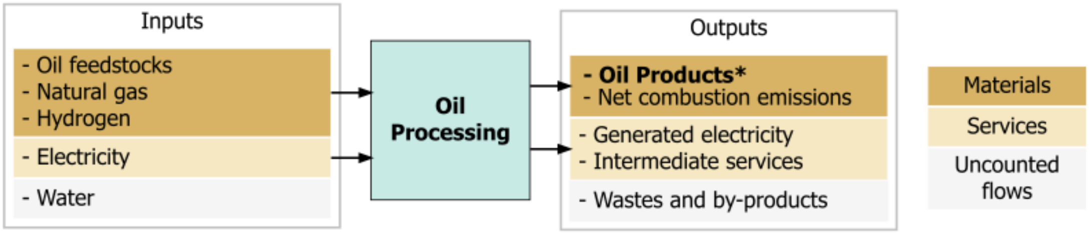

Figure S48: Inputs and outputs for processing oil into oil products. Extracted Oil feedstocks are supplied from the oil extraction process, Section 5.3. Hydrogen is used for upgrading (see main text).

Activity	Process	Resource	Inputs	Outputs	Unit
Oil	FF	ExtractedOilGas	-1.1		Gt/Gt
Oil	FF	Methane	-0.016		Gt/Gt
Oil	FF	Electricity	-0.27		EJ/Gt
Oil	FF	NetEmissions		0.64	Gt_CO2e/Gt
Oil	FF	Hydrogen	-0.0094		Gt/Gt
Oil	FF	Oil		1.0	Gt/Gt

Table S111: Chosen coefficients for oil refining from fossil fuel feedstocks

Property	Crude, NGL and feedstocks	Oil products	Net oil consumption	Natural gas	Petroleum Coke	Refinery Gas and LPG	Electricity	Justification
2018 tranformation, EJ	-183	180	**-3.2**					IEA World Energy Balances (Energy industry transformation, oil refineries)
2018 energy use, EJ			**-1.2**	-2.97	-0.74	-5.06	-1.15	IEA World Energy Balances (Energy industry own-use, oil refineries). Note that heat (1.1 EJ) is not accounted for here
Mass flow, Gt	-4.35	4.25		-0.07	-0.03	-0.11		(2018 Flow) / (Energy Density)
Normalised flow, Gt/Gt or EJ/Gt oil	-1.02	1.00		-0.02	-0.01	-0.03	-0.27	Mass flow normalised by oil product output
Normalised emissions, t CO2e/t oil			**0.08**	0.18	0.07	0.30	0.00	(Fuel consumed) x (emissions factor)

Table S110: Calculation of coefficients for refining oil. The energy flow is from the *oil refineries* flow in International Energy Agency (2021a). Energy density and emissions factors are from Part 2.

5.6.4 Production of Methane

This activity covers the production of methane. Processed natural gas is treated as synonymous with methane for this analysis although it is typically closer to 95% methane by molar composition (Faramawy et al., 2016). This activity includes both processing and refining extracted fossil fuel to produce natural gas, as well as non-fossil fuelled routes. These more novel routes may produce methane from biomass by biochemical processes, or by thermochemical processes using one of two key methanation reactions:

- the Sabatier reaction

$$CO_2 + 4H_2 \rightarrow CH_4 + 2H_2O$$

- the Fischer-Tropsch process

$$CO + 3H_2 \rightarrow CH_4 + H_2O$$

Biomass anaerobic digestion is used as the example biochemical process (Bio-Methane), outlined in Section 5.6.4.3. The Sabatier process is assumed for the thermochemical route. The Fischer-Tropsch reaction is used as a delivery process to provide generic synthetic fuels for transport processes (Section 5.6.11) but has not been included here. The chosen delivery processes are listed in Table S112.

Coefficients for all the novel routes are based on values from one paper for consistency; values are based on modelling for a scenario analysis of a transition to a renewable energy system in Europe by 2050 by Connolly et al. (2016).

Activity	Model Name	Delivery Process
Methane	FF	Methane from fossil-fuel reserves
Methane	SynDAC	Synthetic methane production via the Sabatier Process with Carbon Dioxide as a feedstock
Methane	Biogas	Bio-methane production via anaerobic digestion of biomass

Table S112: Delivery processes for this activity. The coefficients for each delivery process are given and explained in the remainder of this section.

5.6.4.1 Methane from natural gas reserves

This delivery process takes in natural gas from the oil and gas extraction process (see Section 5.3) to produce methane. Physically it represents downstream transportation and processing of natural gas. As described in Section 5.3.4, extracted oil and gas is currently treated in the model as one combined substance, consistent with data sources for global oil and gas extraction. Oil and natural gas are often found in and extracted from the same site.

Currently only the energy and emissions related to pipeline transport are accounted for. The energy (in the form of natural gas) used for pipeline transport was 2.9 EJ in 2018, according to IEA World Energy Balances (equivalent to 2% of total supply, equivalent to a coefficient of -1.02). It is assumed that all of this fuel is used to run the compressors for natural gas pipeline transport and creates emissions via combustion; no fugitive emissions are considered.

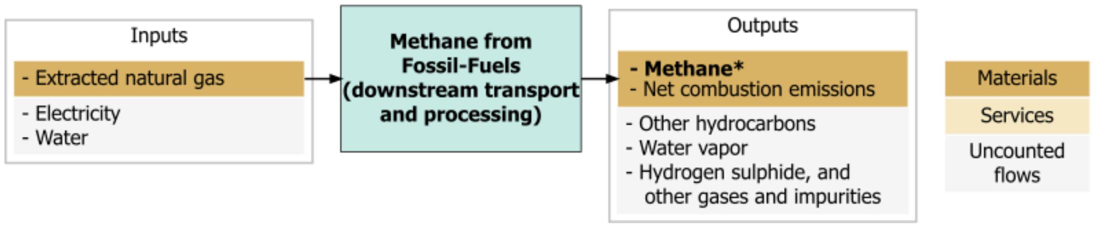

Figure S49: 'Production' of fossil-fuel methane

Activity	Process	Resource	Inputs	Outputs	Unit
Methane	FF	ExtractedOilGas	-1.0		Gt/Gt
Methane	FF	NetEmissions		0.057	Gt_CO2e/Gt
Methane	FF	Methane		1.0	Gt/Gt

Table S113: Chosen coefficients for methane from natural gas reserves.

5.6.4.2 Synthetic Methane via the Sabatier Process with carbon dioxide feedstock

This process combines carbon dioxide with hydrogen to create methane via the Sabatier Reaction (given at the start of Section 5.6.4). The hydrogen production process is accounted for in Section 5.6.5, and carbon dioxide may be produced by any process with carbon-dioxide capture.

Coefficients are based on values used by Connolly et al. (2016) in their scenario analysis of a transition to a renewable energy system in Europe by 2050, assuming the system boundary is reduced to the methanation process alone. This is the same paper used for the bio-methane process to provide some level of consistency. The chosen coefficients are shown in Table S114. These are derived based on the assumptions listed in Table S115.

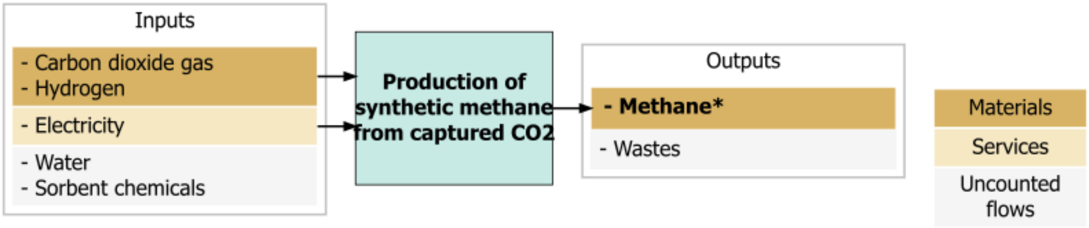

Figure S50: Production of methane via the sabatier reaction with carbon dioxide.

Parameter	Quantity	Unit	Justification
Electricity input for methane compression	1.2	EJ/Gt Methane	0.027 PJ/PJ CH4 from Fig. 6 : Connolly, Mathiesen and Ridjan (2016)
Hydrogen input	0.50	Gt H2/Gt Methane	Stochiometric mass balance for Sabatier reaction. This is equivalent to the quantity of CO2 released in combustion of methane, which is accounted for at the point of use.
CO2 input to process	2.8	Gt CO2/Gt Methane	Stochiometric mass balance for Sabatier reaction. This is equivalent to the quantity of CO2 released in combustion of methane, which is accounted for at the point of use.

Table S115: Key assumptions for estimating the coefficients for synthetic methane production via the Sabatier Process with Carbon Dioxide as a feedstock. The values are based on the process used by Connolly et al. (2014).

Activity	Process	Resource	Inputs	Outputs	Unit
Methane	SynDAC	CO2Product	-2.8		Gt_CO2/Gt
Methane	SynDAC	Electricity	-1.2		EJ/Gt
Methane	SynDAC	Hydrogen	-0.50		Gt/Gt
Methane	SynDAC	Methane		1.0	Gt/Gt

Table S114: Chosen coefficients for synthetic methane production via the Sabatier Process with Carbon Dioxide as a feedstock.

5.6.4.3 Bio-Methane

This process combines syngas (carbon monoxide and hydrogen) from anaerobic digestion of biomass with hydrogen to form methane. Coefficients are based on values used by Connolly et al. (2014) in their analysis of renewable transport fuels in which they quantify physical production pathways for comparison. This is the same reference used for the synthetic methane process to provide some level of consistency. As described in the SI Part 1, although biogenic carbon is generally not explicitly accounted in the model, biogenic sequestration is quantified in fuels and feedstocks which may be produced from both biomass and fossil feedstocks. This means that a constant emissions factor can be assigned to downstream combustion of fuel, independent of the proportion of biogenic carbon in the fuel. Assumptions to derive the coefficients are given in Table S116.

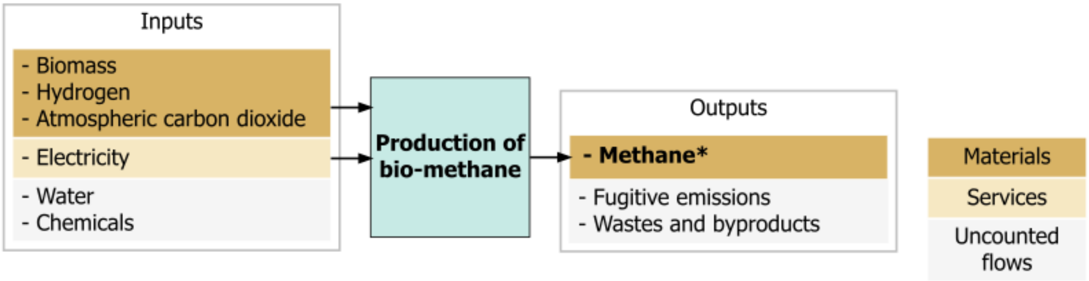

Figure S51: Inputs and outputs for production of methane from anaerobic digestion of biomass. Biogenic carbon sequestration is quantified in this process, as described in the SI Part 1.

Parameter	Quantity	Unit	Justification
**Summary of key data inputs**			
Methane energy density used in source	55.6	kJ/g or GJ/t or EJ/Gt	890 kJ/mol from Connolly, Mathiesen and Ridjan (2015)
Biomass energy density	15	MJ/kg	See document assumptions section
Hydrogen energy density	120	EJ/Gt Hydogen (LHV)	(Giddey et al., 2013)
Carbon content of methane	75%	By mass	From atomic masses
Molecular mass of C	12	g/mol	
Molecular mass of CO2	44	g/mol	
**Calculated coefficients (inputs)**			
Input biomass	2.0	Gt Bio/Gt	0.54 PJ/PJ CH4 from Fig. 7 : Connolly, Mathiesen and Ridjan (2014)
Equivalent carbon dioxide in biomethane	2.8	Gt CO2/Gt	(C content) x (CO2 mass) / (C mass)
Electricity input for methane compression	1.5	EJ/Gt	0.027 PJ/PJ CH4 from Fig. 7 : Connolly, Mathiesen and Ridjan (2014)
Hydrogen input	0.25	Gt H2/Gt	0.54 PJ/PJ CH4 Fig. 7 : Connolly, Mathiesen and Ridjan (2014)

Table S116: Key assumptions for estimating the coefficients for bio-methane production via anaerobic digestion of biomass. The values are based on the process used by Connolly et al. (2014). The treatment of biogenic carbon is described in the SI Part 1.

Activity	Process	Resource	Inputs	Outputs	Unit
Methane	Biogas	Electricity	-1.5		EJ/Gt
Methane	Biogas	NetEmissions	-2.8		Gt_CO2/Gt
Methane	Biogas	NonWoodBiomass	-2.0		Gt_DryBio/Gt
Methane	Biogas	Hydrogen	-0.25		Gt_H2/Gt
Methane	Biogas	Methane		1.0	Gt/Gt

Table S117: Chosen coefficients for bio-methane production via anaerobic digestion of biomass.

5.6.5 Production of Hydrogen

This process describes the production of hydrogen gas. Current hydrogen demand is only around 1% of global energy supply (Staffell et al., 2019) but this may change if hydrogen and its derivatives are used as low-carbon fuels. Current use is mostly within oil refineries to remove impurities and upgrade heavy oil fractions, and a feedstock for other chemicals. Hydrogen is currently almost entirely produced from fossil fuels, with less than 1% of global hydrogen production coming from electrolysis of water (IEA, 2023a). The delivery processes in the model (Table S118) represent both renewable production from electrolysis, as well as the main current fossil-fuel production routes, each with an option for CCS.

Activity	Process	Process Long Name
Hydrogen	SMR	Steam Methane Reformation
Hydrogen	SMRCCS	Steam Methane Reformation with carbon capture
Hydrogen	ATR	AutoThermal Reforming, ATR, of methane
Hydrogen	ATRCCS	AutoThermal Reforming, ATR, of methane with Carbon Capture
Hydrogen	CG	Coal Gasification
Hydrogen	CGCCS	Coal Gasification with carbon capture
Hydrogen	Elec	Electrolysis of water

Table S118: Delivery processes for this activity. The coefficients for each delivery process are given and explained in the remainder of this section.

Hydrogen distribution and leakage are not accounted for in the model on the assumption that these have relatively small effects on overall resource demands. For scenarios where hydrogen plays a major role in the energy mix, that may not be a valid assumption; in future, an additional process for *Hydrogen Distribution* could be added to account for these losses.

Compression is needed to distribute hydrogen for down-stream activities. If hydrogen were liquefied for distribution at ~80 MPa (as planned for the US (DOE Hydrogen and Fuel Cell Technologies Office, 2012)), the process would account for around 30% additional energy losses (Prussi et al., 2020). On the other hand, distribution at lower pressures ~10 MPa, such as the in the proposal compiled by Element Energy Ltd (2018), is likely to have significantly lower energy penalties (less than 10%, based on the values given by Gardiner & Satyapal (2009)). Assuming the latter case, we have not included the energy demands for compression.

Hydrogen leakage would have two impacts on the model coefficients:

1. Increase the input coefficients by the leakage percentage.
2. Any leakage of hydrogen at any point in the supply chain would indirectly lead to atmospheric warming by interfering with reactions which control methane and ozone levels (Derwent, 2018).

These impacts are strongly dependent on the leakage rate, which has a large uncertainty. In their analysis of a UK hydrogen supply chain, Element Energy Ltd (2018) use a leakage rate of 0.5% of the total system flow *"following Ofgem methodology"*, assuming a 10 MPa transmission pressure. That may appear consistent with the US target for leakage in distribution of 0.5% (DOE Hydrogen and Fuel Cell Technologies Office, 2012), although in that case hydrogen is liquefied. Strikingly, these may be optimistic projections given, according to Arrigoni & Bravo Diaz (2022) current leakage rates are between 5% and 20%.

Assuming a leakage rate of around 0.5% is achievable, additional hydrogen demand is not significant but the emissions impact is more unclear. The GWP100 of Hydrogen is highly uncertain but estimated at around 6 kg CO_2e/kg H2 (Derwent, 2018). A leakage of around 0.5% would be equivalent to around 0.03 Gt_{CO2e}/ $Gt_{Hydrogen}$ or 2.5×10^{-4} $Gt_{CO2e}/EJ_{Hydrogen}$. This has not been included in the coefficients, consistent with the overall approach to only consider emissions of carbon dioxide, methane and nitrous oxide (the SI Part 1).

5.6.5.1 Production via SMR of natural gas

This delivery process represents hydrogen production via Steam Methane Reforming (SMR), the dominant form of hydrogen production today, producing ~ 48% of current global supply (Balcombe et al., 2018). As described by Balcombe et al. (2018), the SMR process is composed of two reactions. The first is between methane and steam at 800 C and 30 bar to form hydrogen and carbon monoxide; this mixture is also known as synthesis gas (syngas), and is represented by the steam reforming reaction: $CH_4 + H_2O \rightleftharpoons CO + 3H_2$. The second is a reaction of carbon monoxide with steam to produce more hydrogen (and carbon dioxide), also referred to as a water-gas-shift reaction: $CO + H_2O \rightleftharpoons CO_2 + H_2$. Since the first reaction is highly endothermic, it requires a significant amount of energy to drive the reaction. The second reaction is mildly exothermic. The overall reaction can be summarised in terms of the direct stream reaction:

$$CH_4 + 2H_2O \rightleftharpoons CO_2 + 4H_2$$

Process emissions based on stochiometry of this overall equilibrium reaction are 5.5 kg CO_2/kg H_2 produced. Additional emissions are arise from fuel combustion.

Coefficients for the process have been derived from the results of a process simulation reported by Oni et al. (2022) as part of a comparative assessment. This same reference has been used for all delivery processes from a natural gas feedstock to provide some level of consistency.

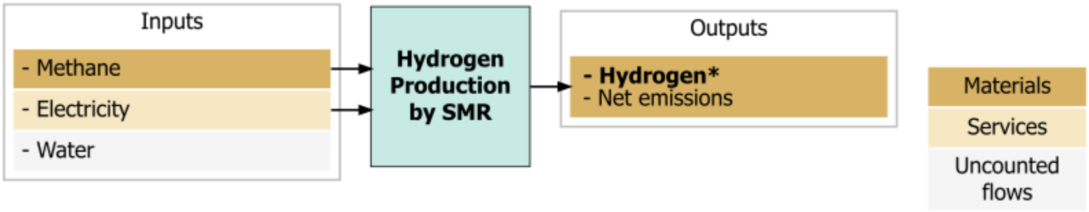

Figure S52: Inputs and outputs for hydrogen production by Steam Methane Reforming (SMR).

Parameter	SMR	Unit	Justification
**Summary of key data inputs**			
Natural gas (feedstock)	2.7	kg/ kg H2	Oni et al. (2022)
Natural gas (fuel)	1.3	kg/ kg H2	Oni et al. (2022)
Emissions (fuel combustion)	3.6	kg CO2e/ kg H2	Oni et al. (2022)
Emissions (process)	5.6	kg CO2e/ kg H2	Assumed to be the onsite emissions for non-CCS processes from Oni et al. (2022) with combustion emissions subtracted.
**Calculated coefficients (inputs)**			
Electricity	3.5	MJ/ kg H2	Oni et al. (2022)
Natural gas	4.0	kg/ kg H2	Sum of fuel and feedstock demands
**Calculated coefficients (outputs)**			
Residual emissions	9.2	kg CO2/ kg H2	Sum of process and combustion emissions, minus any carbon dioxide captured

Table S119: Key assumptions for estimating the coefficients for hydrogen production via SMR. Values provided by Oni et al. (2022) have been converted to the units given here using the energy density and emissions factors in the Assumptions Section (the SI Part 1).

Activity	Process	Resource	Inputs	Outputs	Unit
Hydrogen	SMR	Electricity	-3.5		EJ/Gt
Hydrogen	SMR	Methane	-4.0		Gt/Gt
Hydrogen	SMR	NetEmissions		9.2	Gt_CO2e/Gt
Hydrogen	SMR	Hydrogen		1.0	Gt/Gt

Table S120: Chosen coefficients for hydrogen production by Steam Methane Reforming (SMR).

5.6.5.2 Production via SMR with carbon-dioxide capture (CC)

This delivery process is for hydrogen production via Steam Methane Reforming with carbon-dioxide capture (SMR-CC). As with other production routes from natural gas, the process is based on a process simulation by Oni et al. (2022). The modelled process has capture points in both the furnace and process flue streams, and a capture rate of 85%, using water–gas shift (WGS) reactors and pressure swing adsorption (PSA). The values given here include the energy needed to compress hydrogen to 7 MPa, and carbon dioxide to 8.5 MPa (both 25°C).

The demands are comparable to rough estimates derived from the modelled SMR process (Section 5.6.5.1) using the assumptions of additional fuel demands (40%) and capture rate efficiency (80%) from the review by Balcombe et al. (2018): 5.3 kg CH_4/kg H_2 and 2.0 kg CO_2/kg H_2 emissions.

The data and assumptions used to derive the coefficients are given in Table S121, and the chosen coefficients are shown in Table S122.

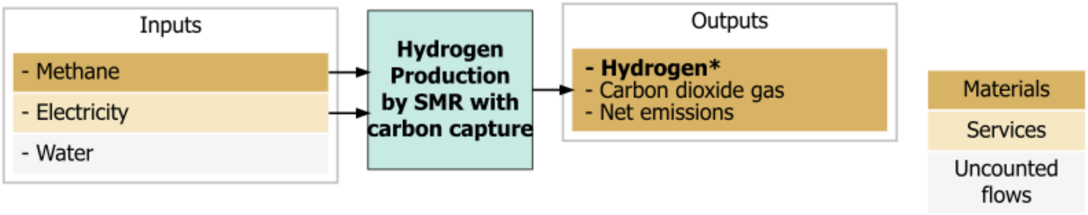

Figure S53: Inputs and outputs for hydrogen production by Steam Methane Reforming with carbon-dioxide capture (SMR-CC).

Parameter	SMR-CCS	Unit	Justification
**Summary of key data inputs**			
Natural gas (feedstock)	2.7	kg/ kg H2	Oni et al. (2022)
Natural gas (fuel)	2.9	kg/ kg H2	Oni et al. (2022)
Emissions (fuel combustion)	7.8	kg CO2e/ kg H2	Oni et al. (2022)
Emissions (process)	5.6	kg CO2e/ kg H2	Assumed to be the onsite emissions for non-CCS processes from Oni et al. (2022) with combustion emissions subtracted.
**Calculated coefficients (inputs)**			
Electricity	16	MJ/ kg H2	Oni et al. (2022)
Natural gas	5.6	kg/ kg H2	Sum of fuel and feedstock demands
**Calculated coefficients (outputs)**			
CO2 for storage	11	kg CO2e/ kg H2	Oni et al. (2022) supplementary information
Residual emissions	2.0	kg CO2/ kg H2	Sum of process and combustion emissions, minus any carbon dioxide captured

Table S121: Key assumptions for estimating the coefficients for hydrogen production via SMR with carbon-dioxide capture. Values provided by Oni et al. (2022) have been converted to the units given here using the energy density and emissions factors in the Assumptions Section (the SI Part 1).

Activity	Process	Resource	Inputs	Outputs	Unit
Hydrogen	SMRCCS	Electricity	-16		EJ/Gt
Hydrogen	SMRCCS	Methane	-5.6		Gt/Gt
Hydrogen	SMRCCS	NetEmissions		2.0	Gt_CO2e/Gt
Hydrogen	SMRCCS	CO2Product		11	Gt_CO2/Gt
Hydrogen	SMRCCS	Hydrogen		1.0	Gt/Gt

Table S122: Chosen coefficients for hydrogen production via SMR with carbon-dioxide capture.

5.6.5.3 Production via ATR of natural gas

This delivery process represents hydrogen production via AutoThermal Reforming (ATR). In the ATR process purified oxygen is used, along with steam, to react with methane and produce hydrogen. Alongside the reactions which occur in SMR (given in Section 5.6.5.2), ATR also involves the following reaction (Speight, 2020):

$$2\ CH_4 + O_2 \rightleftharpoons CO + 4\ H_2$$

The coefficients are derived from a process simulation by Oni et al. (2022). Energy demands for oxygen production using an Air Separation Unit (ASU), and to compress hydrogen to 7 MPa (25°C) are included in the coefficients. The data and assumptions used to derive the coefficients are given in Table S123, and the chosen coefficients are shown in Table S124.

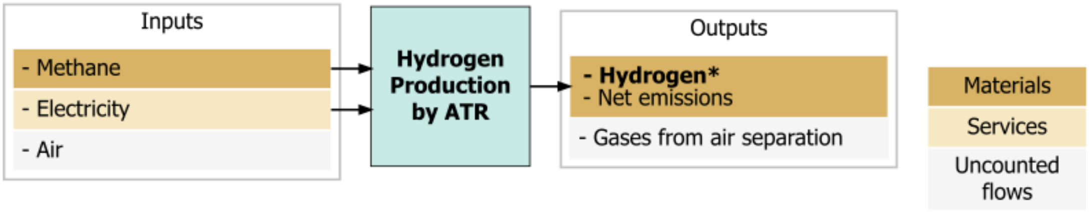

Figure S54: Inputs and outputs for hydrogen production by AutoThermal Reforming (ATR).

Parameter	ATR	Unit	Justification
**Summary of key data inputs**			
Natural gas (feedstock)	3.3	kg/ kg H2	Oni et al. (2022)
Natural gas (fuel)	- 0.00000010	kg/ kg H2	Oni et al. (2022)
Emissions (fuel combustion)	- 0.00000010	kg CO2e/ kg H2	Oni et al. (2022)
Emissions (process)	8.4	kg CO2e/ kg H2	Assumed to be the onsite emissions for non-CCS processes from Oni et al. (2022) with combustion emissions subtracted.
**Calculated coefficients (inputs)**			
Electricity	8.5	MJ/ kg H2	Oni et al. (2022)
Natural gas	3.3	kg/ kg H2	Sum of fuel and feedstock demands
**Calculated coefficients (outputs)**			
Residual emissions	8.4	kg CO2/ kg H2	Sum of process and combustion emissions, minus any carbon dioxide captured

Table S123: Key assumptions for estimating the coefficients for hydrogen production via ATR. Values provided by Oni et al. (2022) have been converted to the units given here using the energy density and emissions factors in the Assumptions Section (the SI Part 1).

Activity	Process	Resource	Inputs	Outputs	Unit
Hydrogen	ATR	Electricity	-8.5		EJ/Gt
Hydrogen	ATR	Methane	-3.3		Gt/Gt
Hydrogen	ATR	NetEmissions		8.4	Gt_CO2e/Gt
Hydrogen	ATR	Hydrogen		1.0	Gt/Gt

Table S124: Chosen coefficients for hydrogen production from ATR.

5.6.5.4 Production via ATR with carbon-dioxide capture (CC)

This delivery process is for hydrogen production via AutoThermal Reforming with carbon-dioxide capture (ATR-CC). The process is based on a process simulation by Oni et al. (2022), with a capture rate of 91%. Energy demands for oxygen production using an Air Separation Unit (ASU), and to compress hydrogen to 7 MPa, and carbon dioxide to 8.5 MPa (both 25°C) are included in the coefficients. The data and assumptions used to derive the coefficients are given in Table S125, and the chosen coefficients are shown in Table S126.

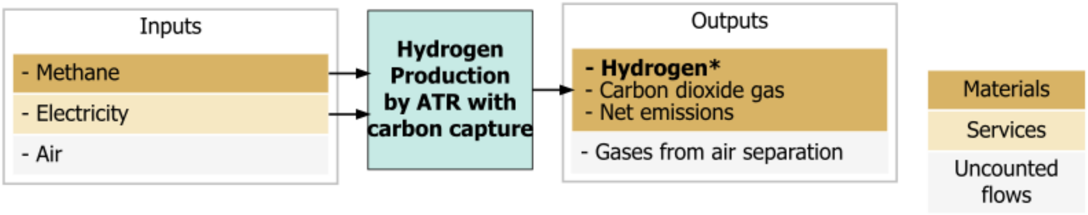

Figure S55: Inputs and outputs for Ammonia production with carbon-dioxide capture

Parameter	ATR-CCS	Unit	Justification
**Summary of key data inputs**			
Natural gas (feedstock)	3.3	kg/ kg H2	Oni et al. (2022)
Natural gas (fuel)	-0.00000010	kg/ kg H2	Oni et al. (2022)
Emissions (fuel combustion)	-0.00000010	kg CO2e/ kg H2	Oni et al. (2022)
Emissions (process)	8.4	kg CO2e/ kg H2	Assumed to be the onsite emissions for non-CCS processes from Oni et al. (2022) with combustion emissions subtracted.
**Calculated coefficients (inputs)**			
Electricity	13	MJ/ kg H2	Oni et al. (2022)
Natural gas	3.3	kg/ kg H2	Sum of fuel and feedstock demands
**Calculated coefficients (outputs)**			
CO2 for storage	7.7	kg CO2e/ kg H2	Oni et al. (2022) supplementary information
Residual emissions	0.71	kg CO2/ kg H2	Sum of process and combustion emissions, minus any carbon dioxide captured

Table S125: Key assumptions for estimating the coefficients for hydrogen production via ATR with carbon-dioxide capture. Values provided by Oni et al. (2022) have been converted to the units given here using the energy density and emissions factors in the Assumptions Section (the SI Part 1).

Activity	Process	Resource	Inputs	Outputs	Unit
Hydrogen	ATRCCS	Electricity	-13		EJ/Gt
Hydrogen	ATRCCS	Methane	-3.3		Gt/Gt
Hydrogen	ATRCCS	NetEmissions		0.71	Gt_CO2e/Gt
Hydrogen	ATRCCS	CO2Product		7.7	Gt_CO2/Gt
Hydrogen	ATRCCS	Hydrogen		1.0	Gt/Gt

Table S126: Chosen coefficients for hydrogen production via ATR with carbon-dioxide capture.

5.6.5.5 Production via Coal Gasification

This process describes hydrogen production by Coal Gasification (CG). The process, which made up around 20% of production in 2018, uses high temperature redox reactions of coal to form hydrogen, as described by Balcombe et al. (2018). The process is complex, involving a series of reactions.

The coefficients are derived from a Life-Cycle Assessment (LCA) by Li & Cheng (2020), based on data from a direct coal liquefaction project operated by China Energy Investment Group. The coal demand is a relatively central estimate compared to values based on Balcombe et al. (2018), Sadeghi et al. (2020) and Li et al. (2022), but the emissions and electricity demand are towards the upper end of the range suggested by other sources. Values chosen here are all based on Li & Cheng (2020) so they provide self-consistent values. Details of the assumptions and calculations are given in Table S127, with the final values summarised in Table S128.

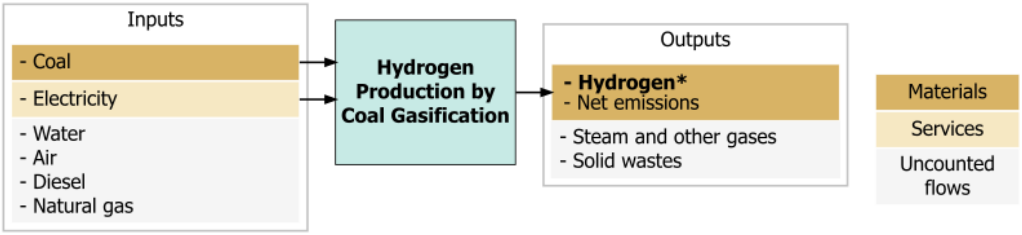

Figure S56: Inputs and outputs for hydrogen production by coal gasification.

Parameter	Quantity	Unit	Justification
**Summary of key data inputs**			
Coal demand	186	MJ/kg H2	Li & Cheng (2020)
Coal energy density	28.0	MJ/kg coal	See Model Assumptions Section
**Calculated coefficients (inputs)**			
Coal demand	6.64	kg/kg H2	Coal demand x H2 energy density / Coal energy density
Electricity demand	20.3	MJ/kg H2	Li & Cheng (2020)
**Calculated coefficients (outputs)**			
Direct emissions from coal conversion process	17.3	kg CO2e/kg H2	Li & Cheng (2020)

Table S127: Key assumptions for estimating the coefficients for hydrogen production by coal gasification . Values for the conventional coal gasification process are taken from Table S127. Energy density and emissions factors are taken from the Document Assumptions Section (the SI Part 1). References: Li & Cheng (2020)

Activity	Process	Resource	Inputs	Outputs	Unit
Hydrogen	CG	Coal	-6.6		Gt/Gt
Hydrogen	CG	Electricity	-20		EJ/Gt
Hydrogen	CG	NetEmissions		17	Gt_CO2e/Gt
Hydrogen	CG	Hydrogen		1.0	Gt/Gt

Table S128: Chosen coefficients for hydrogen production via coal gasification .

5.6.5.6 Production via Coal Gasification with carbon-dioxide capture (CC)

This process describes hydrogen production by Coal Gasification (CG) with carbon-dioxide capture (Figure S57). The coefficients are based on the values used for a carbon footprint assessment by Li et al. (2022) of coal-based hydrogen production in China with carbon-dioxide capture.

The chosen coefficients are shown in Table S129. These are derived based on the assumptions in Table S130.

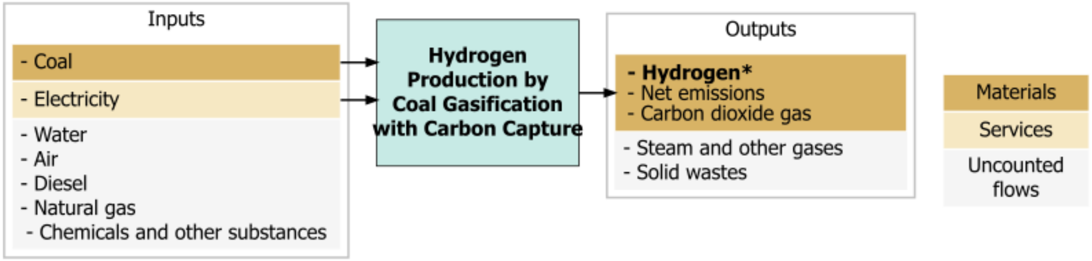

Figure S57: Inputs and outputs for hydrogen production by coal gasification with carbon capture.

Parameter	Quantity	Unit	Justification
**Summary of key data inputs**			
Electricity demand for hydrogen production	15.8	MJ/kg H2	Li et al. (2022)
Electricity demand for carbon capture & compression	12.1	MJ/kg H2	Li et al. (2022)
Direct emissions without CCS	17.8	kg CO2e/kg H2	Li et al. (2022)
Direct emissions with CCS	2.5	kg CO2e/kg H2	Li et al. (2022)
**Calculated coefficients (inputs)**			
Coal demand	8.90	kg/kg	Li et al. (2022)
Total electricity demand	27.9	MJ/kg H2	Sum of electricity demands
**Calculated coefficients (outputs)**			
Carbon for storage	15.3	kg CO2e/kg H2	(emissions without CCS) - (emissions with CCS)
Residual emissions	2.5	kg CO2e/kg H2	Li et al. (2022)

Table S130: Key assumptions for estimating the coefficients for hydrogen production by CG with carbon-dioxide capture. Values for the conventional CG process are taken from Table S130. References: Li et al. (2022)

Activity	Process	Resource	Inputs	Outputs	Unit
Hydrogen	CGCCS	CO2Product		15	Gt/Gt
Hydrogen	CGCCS	Coal	-8.9		Gt/Gt
Hydrogen	CGCCS	Electricity	-28		EJ/Gt
Hydrogen	CGCCS	NetEmissions		2.5	Gt_CO2e/Gt
Hydrogen	CGCCS	Hydrogen		1.0	Gt/Gt

Table S129: Chosen coefficients for hydrogen production via CG with carbon-dioxide capture.

5.6.5.7 Production by Electrolysis

This process process describes hydrogen production by electrolysis (Figure S58). In this process hydrogen is produced from electricity by electrolysis of water. An electrolysis cell is composed of an anode, cathode, electrolyte and membrane but the details of these components vary with different types of electrolyser. There are three main types:

- PEM: Proton Exchange Membrane Electrolysers are well suited for combination with renewable power sources (can adapt well to changing energy input). The output pressure can be 3 - 8 MPa according to Element Energy Ltd (2018), or 1-3 MPa from the literature review of Balcombe et al. (2018).
- Alkaline Electrolysis: a "proven technology with almost 90 years of operational experience" according to Element Energy Ltd (2018) with typical output pressures of 2 – 3 MPa (3.5 MPa according to Balcombe et al. (2018)).
- Solid Oxide: High temperature solid oxide electrolysis (SOE) is an immature technology but could be important in future since it can use industrial waste heat to achieve high efficiency (Element Energy Ltd, 2018). Largest systems existing are ~10 to 100kW as proof of principle units. Given this is an immature technology, there is considerable uncertainty in the projection of future efficiency levels.

The coefficients are based on the data compiled by Element Energy Ltd for the UK government (BEIS) in 2018 (Element Energy Ltd, 2018) and the review paper compiled by Balcombe et al. (2018) (Table S131). The Element Energy report includes descriptions of different hydrogen production and distribution technologies and their significance, estimates of efficiency and costs, and future scenarios for the supply chain development (including roll-out of end-use technologies) (Element Energy Ltd, 2018). The electrolyser efficiency values compiled by Element Energy Ltd (2018) are largely consistent with the review by Balcombe et al. (2018) who find that electrolyser efficiency estimates vary between 50% and 90%. Ancillary processes (water purification, gas/liquid separation and gas compression) have been assumned to be relatively small, and within the level of uncertainty of electrolyser efficiency estimates.

Electrolyser efficiency is taken to be 70%; this is above current levels but consistent with mid-range literature estimates of more mature technologies, and with 2050 capability estimates from Element Energy Ltd (2018). The coefficients do not explicitly account for heat demands for high temperature solid oxide technologies as it is a less mature technology.

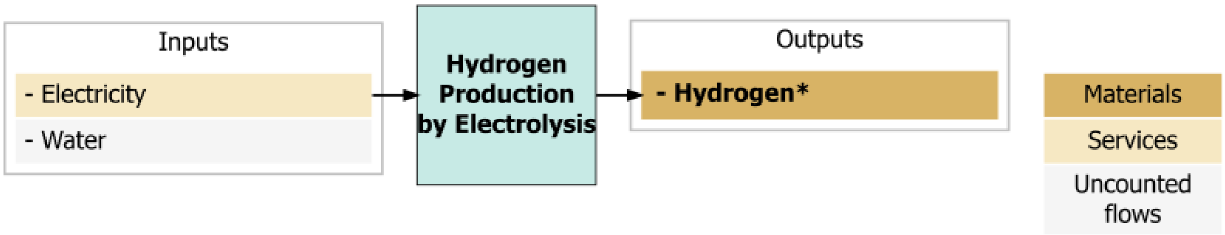

Figure S58: Inputs and outputs for hydrogen production by electrolysis.

	Element Energy Ltd. Best Guess		Balcombe et al., 2018: from literature		
Electrolyser Technology	2020	2050	Lower	Average	Higher bound
PEM electrical efficiency	61%	69%	50%	69%	85%
Alkaline electrical efficiency	65%	69%	48%	68%	83%
Solid Oxide electrical efficiency	85%	95%	60%	80%	90%
Solid Oxide heat demand (GJ heat/GJ H2)	0.24 GJ heat/GJ H2	0.33 GJ heat/GJ H2			

Table S131: Estimates of net electrolyser efficiencies from Element Energy Ltd (2018) and Balcombe et al. (2018).

Parameter	Quantity	Unit	Justification
**Summary of key data inputs**			
Electrolyser efficiency	70%		Mid-range estimate from 2020 technology efficiencies from Element Energy (2018) assuming Solid Oxide with waste heat is unlikely to have an impact at scale
Hydrogen energy density	120	GJ/t	See assuptions section of document
**Calculated coefficients (outputs)**			
Electricity demand	171	EJ/ Gt H2	1/efficiency x H2 energy density

Table S132: Key assumptions for estimating the coefficients for hydrogen production by electrolysis.

Activity	Process	Resource	Inputs	Outputs	Unit
Hydrogen	Elec	Electricity	**-170**		EJ/Gt
Hydrogen	Elec	Hydrogen		**1.0**	Gt/Gt

Table S133: Chosen coefficients for hydrogen production by electrolysis.

5.6.6 Production of Methanol

This activity describes production of methanol from fossil fuels, biomass and other chemical feedstocks. Today, almost 100% of methanol (CH3OH, sometimes referred to as MeOH or methyl alcohol) is produced from fossil fuels, of which around 85% is produced by steam methane reforming natural gas, and around 15% from coal Kauw et al. (2015). There may be emissions associated with fossil fuel combustion in the production of methanol, and where a fossil-fuel feedstock is used, also in downstream combustion of methanol-based products and fuels. Production emissions can be reduced using carbon-dioxide capture technologies (Section 5.6.6.2) while *"negative emissions"* production routes use alternative feedstocks to compensate for downstream emissions. The source of carbon may be via biomass (Section 5.6.6.5) or from carbon-dioxide capture technologies (Section 5.6.6.4), such as those used in Direct Air Capture as described in Section 5.11. The biomass feedstock route currently quantified is biomass gasification because this is the most common approach but other routes are possible such as anaerobic digestion, thermochemical conversion and pyrolysis (Ghasemzadeh et al., 2018).

The delivery processes for methanol production are listed in Table S134 and described in more detail in the remainder of this section.

Activity	Delivery Process	Detail
Methanol	SMR	Steam Methane Reforming
Methanol	CCS	Production from methane with carbon capture
Methanol	Coal	Coal Gasification
Methanol	FromH2	Synthetic Methanol (from Hydrogen)
Methanol	BioGas	Bio-Methanol (by gasification)

Table S134: Delivery Processes for methanol production.

5.6.6.1 Methanol production from natural gas

This delivery process (Figure S59) represents methanol production by Steam Methane Reforming (SMR). According to Dalena et al. (2018), the process comprises three basic steps:

- Producing synthesis gas by steam reforming;
- Converting syngas into crude methanol;
- Distilling the reactor effluent (crude methanol) to the desired purity.

The coefficients are derived from the same sources as used for other petrochemicals (5.6.2) as outlined in Table S135. The calculated emissions are consistent with the value found by Jiang et al. (2024) using a chemical process-based material flow model for production in China (0.63 t CO_2/t).

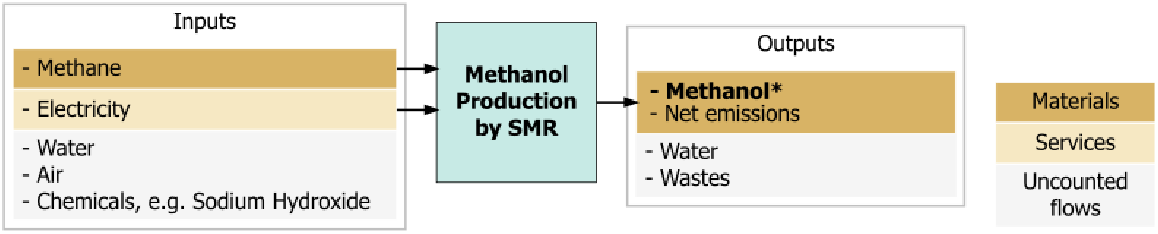

Figure S59: Diagram of Methanol production via Steam Methane Reforming, SMR, of natural gas. Source: Li et al. (2018)

Parameter	Quantity	Unit	Justification
**Summary of key data inputs**			
Natural gas (process energy demand)	10	GJ/t	From Saygin (2021), comparable with IEA (2018) and Saygin (2009)
Natural gas (feedstock demand)	20	GJ/t	Based on tabulated feedstock demand values in (Saygin et al. 2021)
Methane energy density	45	GJ/t	See assuptions section of document
Emissions factor (for fuel usage)	60	kg/GJ	Approx for natural gas as dominant fuel
**Calculated coefficients (inputs)**			
Natural gas demand	0.67	t/ t	Total demand / Methane energy density
Electricity	0.30	GJ/t	Based on tabulated process values in (IEA, 2018)
**Calculated coefficients (outputs)**			
Emissions	0.60	t CO2e / t	Fuel use x Emissions factor

Table S135: Assumptions used to derive coefficients for Methanol via SMR. Energy density and emissions factors are taken from the Document Assumptions Section (the SI Part 1). References: International Energy Agency (2018); Saygin et al. (2009); Saygin & Gielen (2021)

Activity	Process	Resource	Inputs	Outputs	Unit
Methanol	SMR	Methane	-0.67		Gt/Gt
Methanol	SMR	NetEmissions		0.60	Gt_CO2e/Gt
Methanol	SMR	Electricity	-0.30		EJ/Gt
Methanol	SMR	Methanol		1.0	Gt/Gt

Table S136: Coefficients for Methanol via SMR of natural gas.

5.6.6.2 Methanol production from natural gas with carbon-dioxide capture (CC)

This delivery process (Figure S60) describes methanol production by Steam Methane Reforming with carbon-dioxide capture (SMR-CC). The process is based on the process without CC (Section 5.6.6.1) with additional CC demands based on an analysis of Dutch industry by Saygin et al. (2013) and a comparative assessment of CO_2 capture technologies by Kuramochi et al. (2012). The data and assumptions used to derive the coefficients are given in Table S137, with the final values summarised in Table S138.

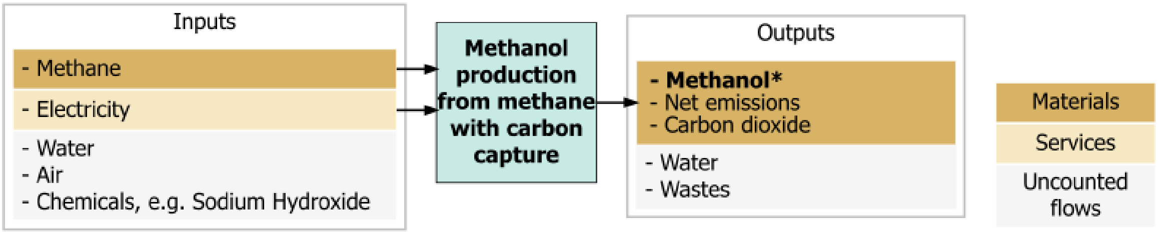

Figure S60: Diagram of Methanol production via Steam Methane Reforming, SMR, of natural gas with carbon-dioxide capture. Source: Li et al. (2018)

Parameter	Quantity	Unit	Justification
**Summary of key data inputs**			
Methane demand w/o CC	0.7	Gt/Gt	SMR process
Electricity demand w/o CC	0.3	EJ/Gt	SMR process
CO2 capture rate	90%		As assumed by Kuramochi et al. (2012)and Saygin et al. (2013)
Unabated emissions from fuel combustion	0.66	Gt CO2/Gt	As derived for the process without CC, scaled by 1.1 to account for additional fuel for production of heat for CC process
CO2 capture electricity demand	0.6	EJ /Gt CO2	As assumed by Saygin et al. (2013)
CO2 capture heat demand	3.6	EJ heat/Gt CO2	As assumed by Saygin et al. (2013)
Heat from waste heat	50%		Assumed
Heat demand from fuel	1.1	EJ heat/Gt	(CO2 captured) x (heat demand) x (1 - waste heat share)
Methane energy density	45	GJ/t	See assuptions section of document
**Calculated coefficients (inputs)**			
Total methane demand	0.690	Gt / Gt	(demand w/o CC) + (heat demand / energy density)
Total electricity demand	0.9	EJ/ Gt	(Electricity demand w/o CCS) + (Electricity for CC)
**Calculated coefficients (outputs)**			
Carbon storage	0.59	Gt CO2/Gt	(CO2 capture rate) x (unabated emissions)
Residual emissions	0.066	Gt CO2e/Gt	(unabated emissions) - (carbon storage)

Table S137: Assumptions used to derive coefficients for methanol production by SMR with carbon-dioxide capture. Energy density and emissions factors are taken from the Document Assumptions Section (the SI Part 1). The process without CCS is described in Section 5.6.6.1. Other references: Kuramochi et al. (2012); Saygin et al. (2013). *w/o CC: without carbon-dioxide capture.*

Activity	Process	Resource	Inputs	Outputs	Unit
Methanol	CCS	Methane	-0.69		Gt/Gt
Methanol	CCS	NetEmissions		0.066	Gt_CO2e/Gt
Methanol	CCS	Electricity	-0.90		EJ/Gt
Methanol	CCS	CO2Product		0.59	Gt_CO2/Gt
Methanol	CCS	Methanol		1.0	Gt/Gt

Table S138: Coefficients for methanol production from SMR with carbon-dioxide capture .

5.6.6.3 Methanol from coal gasification

This delivery process represents methanol production from coal gasification (Figure S61). As with production from natural gas, the process broadly comprises three steps: syngas production, crude methanol synthesis, and purification. In this process syngas is produced by gasification of coal, where 'gasification' describes a high temperature thermo-chemical conversion method to produce gaseous mixtures from solid carbon sources, assisted by gasifying agents such as air/oxygen, steam, and flue gases (Dalena et al., 2018). The process involves many chemical reactions and produces process emissions.

The coefficients are based on Best Practice Technology (BPT) data from Saygin et al. (2009) and International Energy Agency (2018) (as used for other processes), supplemented by data from two other sources. The electricity demand given by Saygin et al. (2009) was 5-10 times higher than values found in other sources (e.g. Li et al., 2018; Liu et al., 2020) so the chosen value was instead taken from a life cycle analysis by Li et al. (2018) of multiplex coal slurry gasification technology in China. The value for process emissions is taken from an analysis of chemical flows in China by Jiang et al. (2024).

The chosen coefficients are shown in Table S139 based on the assumptions in Table S140. The calculated total emissions are consistent with the value found by Jiang et al. (2024) using a chemical process-based material flow model for production in China (3.9 t CO_2/t), and the range in literature (4 - 4.5 t CO_2/t).

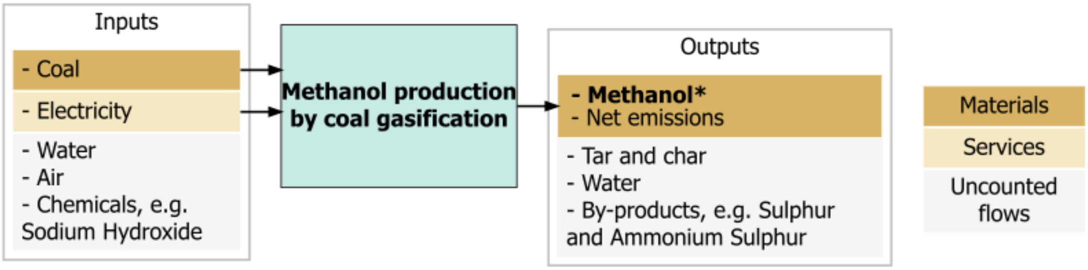

Figure S61: Diagram of Methanol production via Coal Gasification. Source: Li et al. (2018)

Parameter	Quantity	Unit	Justification
**Summary of key data inputs**			
Coal (fuel demand)	21	EJ/Gt	IEA (2018), BPT
Steam energy demand	-4	EJ/Gt	IEA (2018), BPT
Coal (feedstock demand)	20	EJ/Gt	Based on the assumptions used by Saygin, 2009 (BPT values)
Process emissions	2.6	Gt CO2/Gt	Value estimated by Jiang et al. (2024)
Coal energy density	28	GJ/t	See document assuptions
Coal emissions factor	100	Mt CO2/EJ	See document assumptions
Fuel credit for steam export	-3.9	EJ/Gt	See section assumptions
Combustion emissions	1.7	Gt CO2/Gt	(Fuel demand +steam credit) x (Emissions factor)
**Calculated coefficients (inputs)**			
Coal demand	1.3	Gt/Gt	(Sum of coal and steam demands) / (Coal energy density)
Electricity demand	0.7	EJ/Gt	Value used by Li et al. (2018)
**Calculated coefficients (outputs)**			
Emissions			(Process emissions) + (Combustion emissions)

Table S140: Assumptions used to derive coefficients for producing methanol from coal. Excess steam is treated as a reduction in fuel demands, as described in Section 5.6.1. Energy density and emissions factors are taken from the Document Assumptions Section (the SI Part 1). References: International Energy Agency (2018); Saygin et al. (2009); Li et al. (2018); Jiang et al. (2024)

Activity	Process	Resource	Inputs	Outputs	Unit
Methanol	Coal	Coal	-1.3		Gt/Gt
Methanol	Coal	NetEmissions		4.3	Gt_CO2/Gt
Methanol	Coal	Electricity	-0.69		EJ/Gt
Methanol	Coal	Methanol		1.0	Gt/Gt

Table S139: Coefficients for Methanol via coal gasification.

5.6.6.4 Synthetic Methanol

This delivery process describes production of methanol via synthesis of carbon-dioxide and hydrogen Figure S62. Since carbon-dioxide is an unreactive molecule, high temperatures and pressures are needed alongside a suitable catalyst. The process is comprised of three stages: compression, methanol synthesis and distillation.

This process assumes that hydrogen is used as both a fuel and feedstock - it follows that there are no direct residual emissions associated with the process. The coefficients are estimated based on a simulation run by Zang et al. (2021) for a LCA of synthetic methanol production, and the assumptions used by Saygin & Gielen (2021) in developing a global decarbonisation pathway for the chemicals sector. The values found by Zang et al. (2021) are likely to be more a more comprehensive assessment of all process stages since it is more focussed on this specific process but Saygin & Gielen (2021) represents Best Practice Technology (as used for other processes); coefficients are therefore based on the mid-way point between the two sources.

The chosen coefficients are shown in Table S141 based on the assumptions in Table S142.

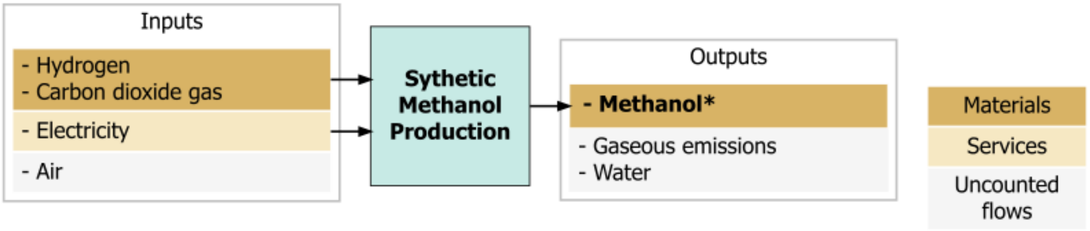

Figure S62: Diagram of Methanol production via synthesis of Hydrogen and Carbon Dioxide. In this process, it is assumed that hydrogen is used both as fuel and feedstock (so that there are no residual emissions). Water is produced as a by-product of the reaction.

Parameter	Quantity	Unit	Justification
Hydrogen	0.20	Gt/Gt Methanol	Zang et al. (2021)
CO2	1.53	Gt/Gt Methanol	Mid-range value from Zang et al. (2021) and Saygin et al. (2021): range of 1.2 - 1.66 t/t.
Electricity	1.62	EJ/Gt Methanol	Mid-range value from Zang et al. (2021) and Saygin et al. (2021): range of 1.2 - 2.0 GJ/t

Table S142: Assumptions used to derive coefficients for synthetic methanol production from hydrogen and carbon dioxide.

Activity	Process	Resource	Inputs	Outputs	Unit
Methanol	FromH2	Hydrogen	-0.20		Gt/Gt
Methanol	FromH2	CO2Product	-1.5		Gt_CO2/Gt
Methanol	FromH2	Electricity	-1.6		EJ/Gt
Methanol	FromH2	Methanol		1.0	Gt/Gt

Table S141: Coefficients for synthetic methanol production from hydrogen and carbon dioxide.

5.6.6.5 Bio-Methanol (by gasification)

This delivery process describes production of methanol from biomass by gasification (Figure S63). The process is based on the assumptions used by Saygin & Gielen (2021) to estimate best practice technology values, using bagasse as the biomass feedstock. Bagasse is the dry fibre left after crushing plants (generally sugarcane/sorghum). The electricity and steam for the process are assumed to be generated onsite from additional biomass. The process has four main stages: gasification to form biogas, biogas cleaning, methanol synthesis and distillation (Rinaldi et al., 2023).

Using biomass as a feedstock for methanol production is treated as a sequestration of carbon because downstream use of methanol produces either plastics (which do not decompose unless incinerated) or fuels (which release CO_2 when burned) [3].

A schematic diagram of the production route is shown in Figure S63. The chosen coefficients are shown in Table S143 based on the assumptions in Table S144.

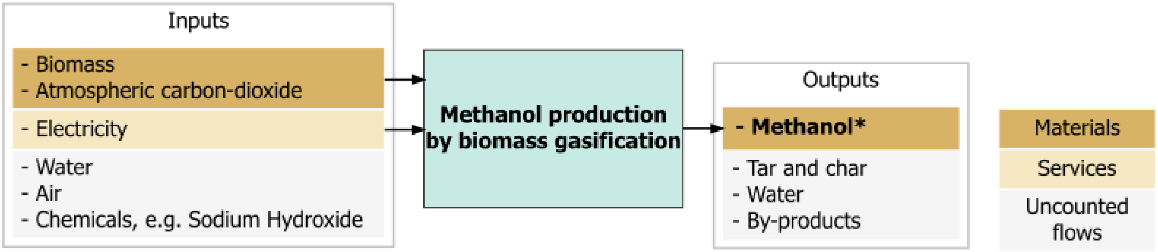

Figure S63: Diagram of Methanol production via gasifaction of biomass. In this process, it is assumed that biomass is used both as fuel and feedstock. Atmospheric carbon-dioxide is treated as an input to this process (see the footnote below).

Parameter	Quantity	Unit	Justification
**Summary of key data inputs**			
Fuel demand (basgasse to provide steam and electricity onsite)	22	EJ/Gt	Using the same assumption as used by Saygin et al. (2021)
Feedstock demand (bagasse)	34	EJ/Gt	Using the same assumption as used by Saygin et al. (2021)
Bagasse energy density	17	EJ/Gt	Table 4 as documented by Annamalai et al. (2014)
Emissions factor (for fuel usage)	0	Mt CO2/EJ	See document assumptions
Carbon content of methanol	0.38	gC/g	From atomic masses
Molecular mass of Carbon	12	g/mol	
Molecular mass of CO2	44	g/mol	
**Calculated coefficients (inputs)**			
Total bagasse demand	3.2	Gt /Gt	(fuel + feedstock)/energy density
CO2 sequestered in biomethanol	1.4	Gt CO2e/ Gt	(C content) x (CO2 mass) / (C mass)

Table S144: Key assumptions for estimating the coefficients for bio-methanol production. Carbon sequestration is accounted for in this process to provide consistency when methanol is combusted (see the footnote below). Other references: Annamalai et al. (2014)

Activity	Process	Resource	Inputs	Outputs	Unit
Methanol	BioGas	NetEmissions	-1.4		Gt_CO2e/Gt_Methanol
Methanol	BioGas	NonWoodBiomass	-3.2		Gt_DryBio/Gt_Methanol
Methanol	BioGas	Methanol		1.0	Gt/Gt

Table S143: Coefficients for methanol production from gasification of biomass.

[3] Sequestration of carbon in biomass and emissions from biomass combustion are not generally accounted for in the model because these are assumed to balance out. The use of biomass to make fuels which can contain a combination of fossil and biogenic carbon is exception to this approach - see the SI Part 1.

5.6.7 Production of High-Value-Chemicals (HVCs)

This activity describes the production of High Value Chemicals (HVCs - Ethylene, Propylene and BTX aromatics), key precursors to plastics and other chemical production. The delivery processes are based on three aggregated routes, given in Table S145 and Figure S64. The aggregated approach is used currently to minimise the modelling complexity but could be disagregated into constituent chemicals and processes in a future iteration of the model (as described in SI Part 2.3.

Coefficients for all delivery processes for aggregated HVC production are derived by scaling energy and feedstock demands for more specific production processes by the assumed proportions of production. Values for energy consumption are based on values used in the IEA report, the Future of Petrochemicals (2018). These are tabulated in Table S146 alongside production shares; the distribution of production for the conventional production process is based on the flow map developed by Levi & Cullen (2018) and the data in Table S147, assuming the base 2018 case. To simplify modelling, it is assumed that there is no significant change in these production shares. The realism of that assumption, and the impact of changing production shares could be investigated in future work. The feedstock demand is based on data from Saygin & Gielen (2021), shown in Table S147.

Activity	Process	Process Long Name
HVCs	BioEth	Bio-compatible route: Ethylene from Bioethanol dehydration, other HVCs from methanol
HVCs	NET	Conventional Production with NETs
HVCs	MTOA	Methanol (methyl alcohol) to olefins/aromatics
HVCs	CCS	Production with carbon capture

Table S145: Delivery processes for this activity. The coefficients for each delivery process are given and explained in the remainder of this section.

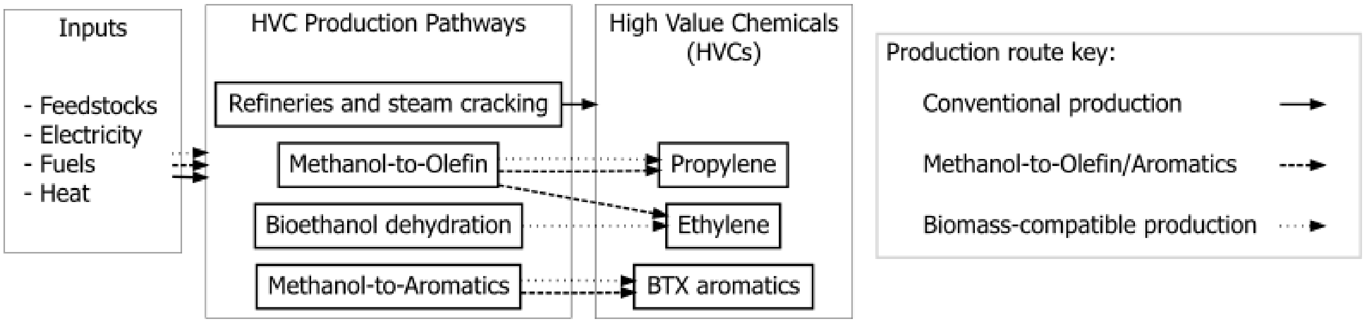

Figure S64: Production routes (delivery processes) for High Value Chemicals (HVCs).

Process	Primary product(s)	BPT energy demand (GJ/t) Fuel	Steam	Elec.	Assumed proportion of HVCs (by mass) Conventional route	Methanol-to-olefin route	Bio route
**Disagreggated HVC Processes**							
Refinery sourced by-products	HVCs		2.0	0.10	46%		
Ethane steam cracking	HVCs	14	-1.4	0.30	8%		
Naphtha steam cracking	HVCs	13	-1.4	0.30	31%		
LPG steam cracking	HVCs	14	-1.4	0.30	6%		
Gas oil steam cracking	HVCs	12	-1.4	0.30	3%		
Naphtha catalytic cracking	HVCs	11	-1.2	0.30	5%		
Ethanol dehydration	Ethylene	1.6	46	1.90	0%		
Propane dehydrogenation	Propylene	8.9	2.2	0.10	1%		
Methanol to olefins	Ethylene, propylene	5.9	-1.1	0.20	1%	50%	20%
Methanol to aromatics BTX	aromatics	5.9	-1.1	0.20	0%	50%	45%
**Weighted averages for aggregated HVC processes**							
Conventional processes		7.0	0.18	0.21	100%		
Methanol-to-olefins/aromatics		5.9	-1.1	0.20		100%	
Bio-compatible route: Ethylene from Bioethanol dehydration, other HVCs from methanol		3.8	-0.72	0.13			65%

Table S146: Tabulated assumptions of Best Practice Technologies (BPT) energy demands for specific HVC processes, as used in (International Energy Agency, 2018), scaled by assumptions from this work to give aggregated demands for fuel, steam and electricity. The production shares for the conventional route are based on data in Table S147, International Energy Agency (2018) - the Future of Petrochemicals, and the SI of Levi & Cullen (2018). The remaining 35% for the bio-route is attributed to the ethylene from biomass process used by Saygin & Gielen (2021). The production shares are derived for the base 2018 case and it is assumed that there is no significant change in future to simplify modelling.

Product	Process route	Production Share	Feedstock Use (GJ/t)
Ethylene	steam cracking	32%	45
Propylene	steam cracking	12%	45
Propylene	fluid catalytic cracking	5%	45
Benzene	steam cracking	4%	0
Benzene	naphtha extraction	11%	40
Toluene		6%	20
Xylene		11%	41
Butadiene	steam cracking	4%	0
Butadiene	C4 separation	4%	45
Butylene		7%	45
Carbon black		4%	33
**TOTAL**	**weighted average**	**100%**	**38**

Table S147: Data used for estimating feedstock demands and production shares of conventional HVC

chemicals used in Table S146. Data is taken from the 2017 scenario used by Saygin & Gielen (2021)

5.6.7.1 Conventional production of HVCs

This delivery process describes production of HVCs following the dominant current practice, process routes and technologies, i.e. from steam-cracking and refineries directly (Figure S65). Values are derived from the process energy data and production shares in Table S146, feedstock values in Table S147, and the resulting specific assumptions in Table S148. The derived coefficients are shown in Table S149.

The distribution of fuels has been based on the EU domestic production in 2015, derived from the emissions distribution found by Cabernard et al. (2022). The global distribution had a significantly higher dependence on coal (Cabernard et al., 2022) but it is assumed that future production routes will prioritise lower emissions fuels, such as those currently used in Europe. This route is based on Best Practice Technology assumptions. These are significantly lower than the average technology values as reported by International Energy Agency (2018). Using the average technology values would increase the overall fuel demand to 8.2 EJ/Gt HVCs and increase emissions to 0.7 $GtCO_2$/Gt HVCs.

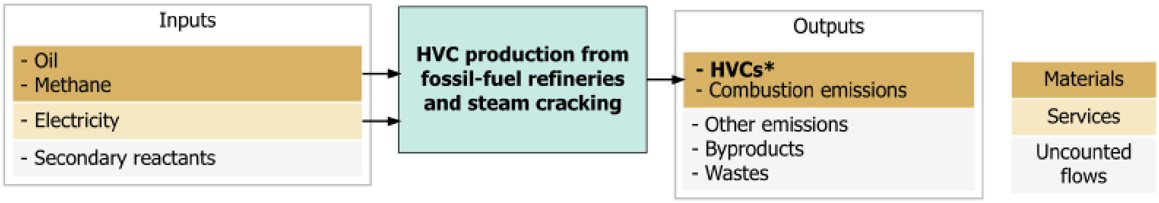

Figure S65: Inputs and outputs for HVC production from fossil-fuel refineries and steam cracking.

Property	Quantity	Unit	Justification
**Summary of key data inputs**			
Fuel demand	7.0	EJ/Gt	Fuel use from IEA (2018), scaled by mass of each chemical from Levi & Cullen (2013)
Steam demand	0.18	EJ/Gt	Steam use from IEA (2018), scaled by mass of each chemical from Levi & Cullen (2013)
Total fuel demand	7.2	EJ/Gt	Assuming additional fuel required for steam generation, as described in section assumptions
Oil (fuel)	0.12	Gt/Gt	Share of total fuel scaled by the emissions distribution found by Cabernard et al. (2022) for domestic EU production in 2015
Oil (feedstock)	0.92	Gt/Gt	38.5 EJ/Gt, based on tabulated feedstock demand values in (Saygin et al., 2009; Saygin et al. 2021)
**Calculated coefficients (inputs)**			
Oil (fuel and feedstock)	1.0	Gt/Gt	Sum of fuel and feedstock
Coal (fuel)	0.04	Gt/Gt	As for oil (fuel)
Methane (fuel)	0.027	Gt/Gt	As for oil (fuel)
Electricity	0.21	EJ/Gt	From tabulated process values in (Saygin et al., 2009; IEA, 2018)
**Calculated coefficients (outputs)**			
Direct emissions	0.52	Gt CO2/Gt	Derived from assumed fuel demands and emissions factors

Table S148: Assumptions used to derive coefficients for HVC production via fossil-fuel refineries and steam cracking. Assumptions are based on data in Table S146 and Table S147. Energy density and emissions factors are taken from the Document Assumptions Section (the SI Part 1).

Activity	Process	Resource	Inputs	Outputs	Unit
HVCs	NET	Coal	-0.038		Gt/Gt
HVCs	NET	Oil	-1.0		Gt/Gt
HVCs	NET	Methane	-0.027		Gt/Gt
HVCs	NET	NetEmissions		0.52	Gt_CO2e/Gt
HVCs	NET	Electricity	-0.21		EJ/Gt
HVCs	NET	HVCs		1.0	Gt/Gt

Table S149: Coefficients for HVC production via conventional processes

5.6.7.2 Methanol (methyl alcohol) to olefins/aromatics

This process describes the production of HVCs from methanol feedstock. Methanol-to-olefins is a relatively newly commercialised technology to produce light olefins (ethylene and propylene) from methanol feedstock (Levi & Cullen, 2018). Methanol-to-aromatics is only at the pilot plant stage (International Energy Agency, 2018)) but has been included here to give a common route for all HVCs. A schematic diagram of the production route is shown in Figure S66. The chosen coefficients are shown in Table S150. Values are derived from the process energy data and production shares in Table S146, feedstock values in Table S147, and the resulting specific assumptions in Table S151.

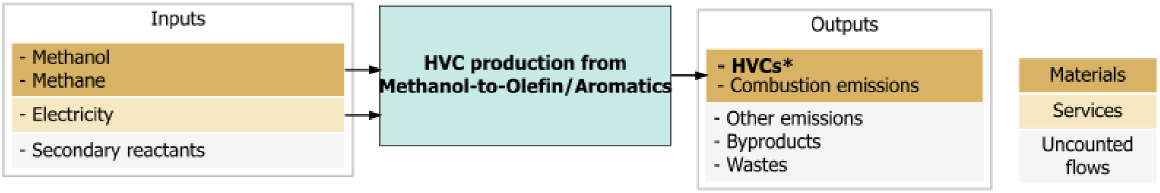

Figure S66: Diagram of HVC production via Methanol-to-Olefin/Aromatics.

Property	Quantity	Unit	Justification
**Summary of key data inputs**			
Refinery sourced olefins and aromatics	0%	Of all HVC production	
Methane (fuel)	5.9	EJ/Gt	Fuel use from IEA (2018), scaled by mass of each chemical from Levi & Cullen (2013)
Steam demand	-1.10	EJ/Gt	Steam use from IEA (2018), scaled by mass of each chemical from Levi & Cullen (2013)
Total fuel demand	4.2	EJ/Gt	Assuming fuel credit for steam exports, as described in section assumptions
Methane energy density	45	GJ/t	See assuptions section of document
Methane emissions factor	60	Mt CO2/EJ fuel	See assuptions section of document
**Calculated coefficients (inputs)**			
Methanol (feedstock)	3.1	Gt/Gt	Based on assumptions used by Levi & Cullen (2018)
Methane	0.1	Gt/Gt	(methane demand) / (methane energy density)
Electricity	0.2	EJ/Gt	From tabulated process values in (Saygin et al., 2009; IEA, 2018)
**Calculated coefficients (outputs)**			
Direct emissions	0.25	Gt CO2/Gt	(Fuel +steam credit) x emissions factor

Table S151: Assumptions used to derive coefficients for HVC production via Methanol-to-Olefin/Aromatics. Assumptions are based on data in Table S146 and Table S147.

Activity	Process	Resource	Inputs	Outputs	Unit
HVCs	MTOA	Methane	-0.094		Gt/Gt
HVCs	MTOA	Methanol	-3.1		Gt/Gt
HVCs	MTOA	NetEmissions		0.25	Gt_CO2e/Gt
HVCs	MTOA	Electricity	-0.20		EJ/Gt
HVCs	MTOA	HVCs		1.0	Gt/Gt

Table S150: Coefficients for HVC production via Methanol-to-Olefin/Aromatics.

5.6.7.3 Bio-compatible route: Ethylene from Bioethanol dehydration, other HVCs from methanol

This process describes production of ethylene by bioethanol dehydration, and other HVCs from methanol. This route has been chosen as an option to demonstrate a focussed dependence on biomass. Other HVCs cannot be produced from ethanol directly and so are assumed to be produced from methanol (which could be produced via biomass gasification or other bio- routes). A schematic diagram of the production route is shown in Figure S67. Values are derived from the process energy data and production shares in Table S146, feedstock values in Table S147, and the resulting specific assumptions in Table S152. The derived coefficients are shown in Table S153.

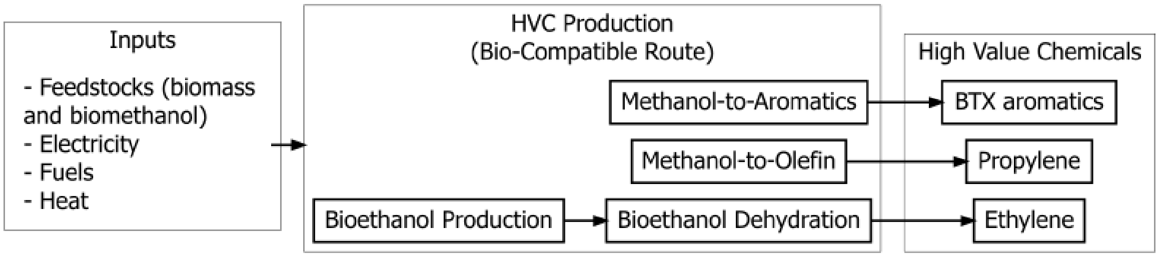

Figure S67: HVC production via a fully biomass compatible route (assuming methanol from biomass).

Property	Quantity	Unit	Justification and Notes
**Ethylene from bioethanol dehydration**			
Biomass (feedstock and fuel)	35	EJ/Gt	Assumption used by Saygin et al. (2021): "Bio-based ethylene production requires in total 100 GJ bio/ t ethylene. ", scaled by the share of ethylene in HVCs
Biomass energy density	15	EJ/Gt dry bio	See assuptions section of document
Electricity	0.7	EJ/Gt	Value used by Saygin et al. (2021), scaled by production volumes
**Other HVCs from methanol**			
Fuel	3.8	EJ/Gt	Fuel use from IEA (2018), scaled by each chemical's mass from Levi & Cullen (2013)
Steam	-1.1	EJ/Gt	Steam use from IEA (2018), scaled by masses from Levi & Cullen (2013)
Total fuel	3.1	EJ/Gt	Assuming fuel credit for steam exports
Methanol (feedstock)	2	Gt/Gt	Using assumption used by Levi & Cullen (2018) of 3.1 t methanol/ t HVC, scaled by production volumes from Levi & Cullen (2013)
Electricity	0.13	EJ/Gt	Based on the ranges of electricity use from IEA (2018), scaled by production volumes from Levi & Cullen (2013).
Biomass	2.3	Gt/Gt	Biomass input / energy density
**Calculated coefficients (inputs)**			
Electricity	0.80	EJ/Gt	Sum of electricity demand for ethylene and other HVCs
Methanol	1.99	Gt/Gt	Feedstock demand for non-ethylene HVCs
Methane	0.061	Gt/Gt	Total demand / methane energy density
**Calculated coefficients (outputs)**			
Direct emissions	0.16	Gt CO2/Gt	Total fuel demands x emissions factors

Table S152: Assumptions used to derive coefficients for HVC production via a fully biomass compatible route (assuming methanol from biomass). Assumptions are based on data in Table S146 and Table S147.

Activity	Process	Resource	Inputs	Outputs	Unit
HVCs	BioEth	Methane	-0.061		Gt/Gt
HVCs	BioEth	Methanol	-2.0		Gt/Gt
HVCs	BioEth	NonWoodBiomass	-2.3		Gt/ EJ_fuel
HVCs	BioEth	NetEmissions		0.16	Gt_CO2e/Gt
HVCs	BioEth	Electricity	-0.80		EJ/Gt
HVCs	BioEth	HVCs		1.0	Gt/Gt

Table S153: Coefficients for HVC production from biomass and methanol.

5.6.8 Production of Plastics

This activity describes the production of plastics; plastics are treated as an aggregated product which is made from a feedstock of (aggregated) HVCs (High Value Chemicals) or waste plastics. The production of high-value chemicals is described in Section 5.6.7. Plastics shaping to form final products is accounted for in the Section on other industrial processes. Plastics production is energy and emissions intensive due to the high temperatures required. The delivery processes describe plastics production from HVCs with different energy sources, and electrified production of plastics from waste plastics (recycling). Only mechanical recycling ('primary' or 'secondary') is included at this stage since this is lower energy demand than chemical recycling or feedstock recycling ('tertiary' recycling). 'Primary' recycling melts down a single plastic type to create a product to meet the same purpose as the original plastic, while 'secondary' (mechanical) recycling produces materials for different purposes, often of lower value (Rahimi & García, 2017). 'Tertiary' recycling in contrast, aims to break down the plastic into its constituent monomers or basic chemicals for reuse in the production of new plastics or other products, for instance by pyrolysis (Rahimi & García, 2017).

Coefficients for plastic production are mostly based on BPT values used by Saygin et al. (2009). Energy demands for production of individual plastic types (as used by Saygin et al. (2009)) have been aggregated, based on assumed representative process routes, and scaled to give approximate fuel, steam and electricity intensity values for a "generic plastic" (see Table S154). These values are used as the basis for deriving the delivery process coefficients. The process routes are based on the map compiled by Levi & Cullen (2018). Where processes quantified by Saygin et al. (2009) occur sequentially on the map, the values are summed, but where the processes appear as parallel routes, average values are used for the generic process.

Values for HVC feedstock demand are assumed to be the same for all production routes, assuming a yield rate of around 0.85. This assumption is based on the 45 EJ fossil feedstock demand for plastics used by Saygin & Gielen (2021) compared to the derived feedstock demand found for aggregated HVC production in Section 5.6.7 (39 EJ). Only HVCs are considered as feedstock. Other chemicals (including methanol, ammonia and urea) made up less than 5% of the chemical input for plastic production in 2013, based on the map compiled by Levi & Cullen (2018).

Activity	Process	Process Long Name
Plastics	NET	Conventional Methods
Plastics	Elec	Electrical Processes replace Fossil Fuels
Plastics	Bio	BioFuels replace Fossil Fuels
Plastics	CCS	Plastic production with integrated carbon capture
Plastics	RecycleElec	Electrified mechanical recycling

Table S155: Delivery processes for this activity. The coefficients for each delivery process are given and explained in the remainder of this section.

Process	Electricity	Fuel	Steam	Share of total plastics
**Polyethylene production**				
Polyethylene, high density (HDPE)	0.9		1.0	
Polyethylene, low density (LDPE)	3.5		-2.1	
Polyethylene, (LLDPE)	0.4		1.6	
**Mean values**	**1.6**		**0.2**	**29%**
**PVC production**				
Vinyl chloride monomer	0.4	2.7	0.0	
Polyvinyl chloride (PVC)	0.6	0.5	1.2	
**Sum of values**	**1.0**	**3.2**	**1.2**	**11%**
**PP production**				
Polypropylene (PP)	0.9		0.1	
**Mean values**	**0.9**		**0.1**	**18%**
**Polystyrene production**				
Styrene			7.7	
Polystyrene (PS)	0.4	0.5		
**Sum of values**	**0.4**	**0.5**	**7.7**	**9%**
**Synthetic textiles and fibres production**				
Ethylene glycol (EG)	0.2	0.8	3.5	
Purified terephthalic acid (PTA)	0.3		2.6	
Polyethylene terephthalate (PET)	0.7	4.1		
Formaldehyde & fibres	0.2		2.0	
Synthetic rubber & latex	2.5		19.9	
**Mean and Sum of values**	**2.3**	**4.5**	**14.0**	**15%**
**Other plastics**				
Ethylene oxide (EO)	0.8	2.5		
Ethylene dichloride (EDC)	0.2	4.4		
Phenolic resins/Phenol formaldehyde resins			10.0	
Polycarbonate	2.2		10.3	
**Sum of values**	**2.1**	**6.9**	**10.2**	**19%**
**Generic plastic**				
	**1.5**	**2.4**	**4.9**	**100%**

Table S154: Energy demand data (GJ/t) and assumptions used to derive production coefficients for plastic production. Data for individual plastic production process are taken from Saygin et al. (2009). The combination of these data to form more generalised demand values is based on assumed representative process routes using the map compiled by Levi & Cullen (2018) as a reference - where processes appear to occur in parallel, an average is taken but where they seem to be sequential, the final value is the sum of the data. Assumed production shares are also based on the map of Levi & Cullen (2018). All data given in GJ/t unless specified.

5.6.8.1 Fossil-fuelled processes

This process (Figure S68) describes plastics production from HVCs, where most process energy is from fossil fuels (as today). Natural gas, coal and oil are assumed to provide steam for the process, and combustion emissions are considered to be the only significant residual emissions in production. The distribution of fuels is based on that found by Cabernard et al. (2022) for the global plastics supply chain in 2015. The coefficients are derived based on the assumed energy demands and production shares shown in Table S154, and the assumptions in Table S156.

The residual emissions are approximately consistent with the process modelled by Gabrielli et al. (2023) (0.75 $GtCO_2$/Gt plastic). Note that hydroflurocarbon emissions are not accounted for; these accounted for around 2% of the carbon footprint of global plastics in 2015 according to Cabernard et al. (2022)).

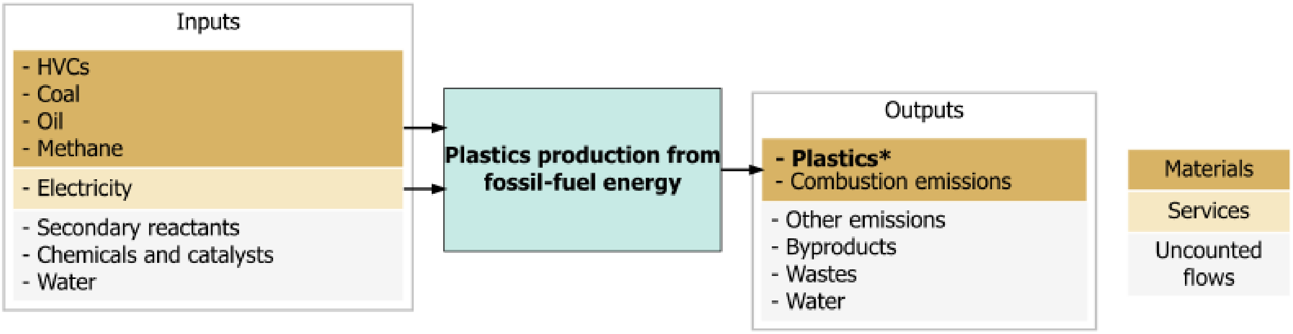

Figure S68: Inputs and outputs for plastics production using fossil-fuels.

Property	Quantity	Unit	Justification
**Summary of key assumptions**			
Fuel demand	2.4	EJ/Gt	”Generic plastic” energy demands
Steam demand	4.9	EJ Steam/Gt	”Generic plastic” energy demands
Steam production efficiency	0.9	EJ Steam/EJ Fuel	Steam produced per EJ methane, as used by Paoli, Lupton & Cullen (2018)
Coal share of energy supply	48%		Based on the fuel distribution found by Cabernard et al. (2022) for the whole global plastics supply chain in 2015
Oil share of energy supply	43%		As for coal
Methane share of energy supply	9%		As for coal
**Calculated coefficients (inputs)**			
HVC feedstock demand	1.1	Gt HVCs/Gt	Estimated from the flow map produced by Levi & Cullen (2018)
Electricity	1.5	EJ/Gt	”Generic plastic” energy demands
Coal demand	0.13	EJ/Gt	Steam and fuel demands
Oil demand	0.082	EJ/Gt	Steam and fuel demands
Methane demand	0.015	EJ/Gt	Steam and fuel demands
**Calculated coefficients (outputs)**			
Emissions	0.66	Gt CO2/Gt	Derived from assumed fuel demands and emissions factors

Table S156: Key assumptions for estimating the coefficients for plastics production using fossil fuels. Electricity, fuel and steam demands for “generic plastic” production are taken from Table S154. Fuel and emissions values are derived using the energy density values and emissions factors in the SI Part 2. Other references: Paoli et al. (2018), Cabernard et al. (2022), Saygin & Gielen (2021)

Activity	Process	Resource	Inputs	Outputs	Unit
Plastics	NET	HVCs	-1.1		Gt/Gt
Plastics	NET	Coal	-0.13		Gt/Gt
Plastics	NET	Oil	-0.082		Gt/Gt
Plastics	NET	Methane	-0.015		Gt/Gt
Plastics	NET	NetEmissions		0.66	Gt_CO2e/Gt
Plastics	NET	Electricity	-1.5		EJ/Gt
Plastics	NET	Plastics		1.0	Gt/Gt

Table S157: Chosen coefficients for plastics production using fossil fuels.

5.6.8.2 Electrified processes

This process describes plastics production from HVCs where all energy is provided by electricity (Figure S69). Coefficients are derived based on the assumed energy demands and production shares shown in Table S154, and the assumptions in Table S158.

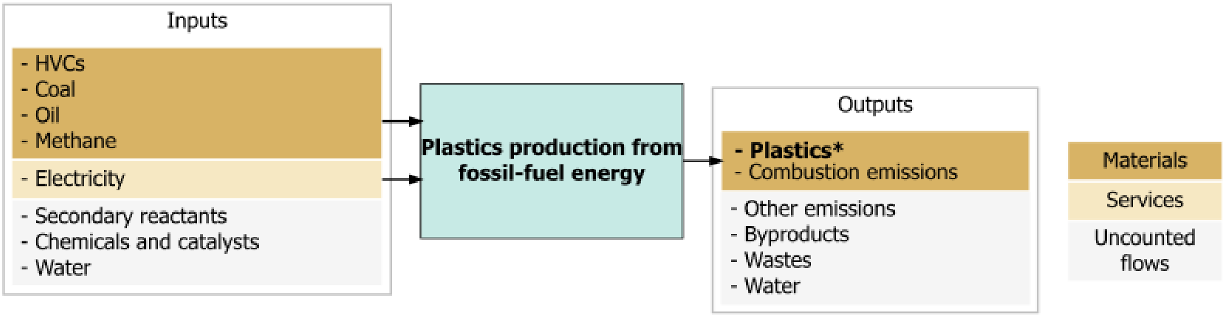

Figure S69: Inputs and outputs for electrified plastics production.

Property	Quantity	Unit	Justification
**Summary of key assumptions**			
Steam demand	4.9	EJ Steam/Gt	"Generic plastic" energy demands
Steam production efficiency	1.0	EJ Elec/EJ Steam	Assumed electricity required for steam production.
Conventional fuel demand	2.4	EJ/Gt	"Generic plastic" energy demands
Electrification efficiency	0.9	EJ Elec/EJ Fuel	Assumed efficiency of electrified heating processes to be more targetted relative to fossil fuel
Electricity for fuel and steam	6.8	EJ/Gt	Sum of fuel and steam using assumed relative efficiencies
Electricity process demand	1.5	EJ/Gt	"Generic plastic" energy demands
**Calculated coefficients (inputs)**			
Total electricity demand	8.3	EJ/Gt	Steam and fuel
HVC feedstock demand	1.1	Gt HVCs/Gt	Estimated from the flow map produced by Levi & Cullen (2018)

Table S158: Key assumptions for estimating the coefficients for plastics production via electrified processes. Electricity, fuel and steam demands for "generic plastic" production are taken from Table S154. Other references: Saygin & Gielen (2021)

Activity	Process	Resource	Inputs	Outputs	Unit
Plastics	Elec	HVCs	-1.1		Gt/Gt
Plastics	Elec	Electricity	-8.3		EJ/Gt
Plastics	Elec	Plastics		1.0	Gt/Gt

Table S159: Chosen coefficients for plastics production via electrified processes.

5.6.8.3 Biomass-fuelled processes

This process describes the production of plastics from HVCs, using biomass to provide both fuel and steam for the process (Figure S70). Combustion emissions (of biomass) are considered to be the only significant residual emissions in production but these are assumed to be compensated by sequestration during growth and so not explicitly accounted (the SI Part 1). Coefficients are derived based on the assumed energy demands and production shares shown in Table S154, and the assumptions in Table S160.

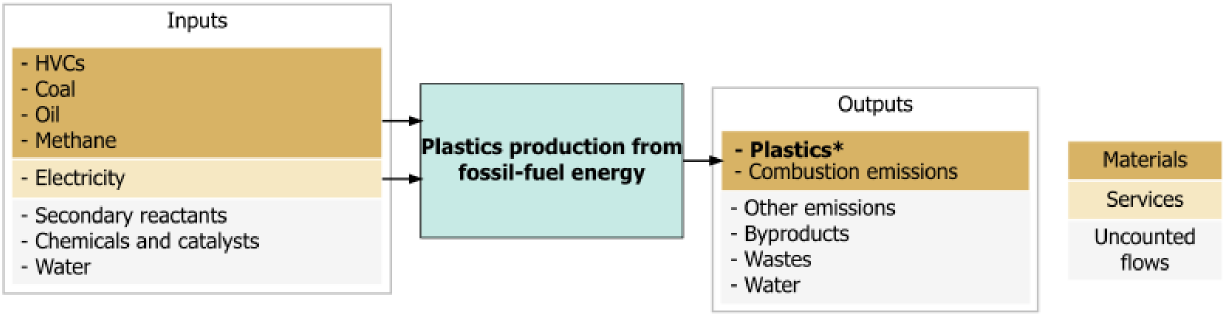

Figure S70: Inputs and outputs for plastics production using biomass to generate heat and steam.

Property	Quantity	Unit	Justification
**Summary of key assumptions**			
Conventional fuel demand	2.4	EJ/Gt	”Generic plastic” energy demands
Steam demand	4.9	EJ Steam/Gt	”Generic plastic” energy demands
Steam production efficiency	0.7	EJ steam/EJ Biomass	The same assumption as used by Paoli, Lupton & Cullen (2018) for biomass boilers
Biomass fuel replacement efficiency	1.0	EJ biomass/EJ fuel	Assumed efficiency of biomass relative to fossil fuel
Biomass (fuel demand)	9.4	EJ/Gt	Sum of fuel and steam using assumed relative efficiencies
Bagasse energy density	17.3	EJ/Gt dry bio	Table 4 as documented by Annamalai et al. (2014)
HVC feedstock demand	1.05	Gt HVCs/Gt	Estimated from the flow map produced by Levi & Cullen (2018)
**Calculated coefficients (inputs)**			
Electricity	1.5	EJ/Gt	”Generic plastic” energy demands
Total biomass demand	0.54	Gt dry bio/Gt	

Table S160: Key assumptions for estimating the coefficients for plastics production via natural gas fuelled processes. Electricity, fuel and steam demands for “generic plastic” production are taken from Table S154. Fuel and emissions values are derived using the energy density values and emissions factors in the SI Part 2. Other references: Paoli et al. (2018), Annamalai et al. (2014), Saygin & Gielen (2021)

Activity	Process	Resource	Inputs	Outputs	Unit
Plastics	Bio	HVCs	-1.1		Gt/Gt
Plastics	Bio	NonWoodBiomass	-0.54		Gt_DryBio/Gt
Plastics	Bio	Electricity	-1.5		EJ/Gt
Plastics	Bio	Plastics		1.0	Gt/Gt

Table S161: Chosen coefficients for plastics production via natural gas fuelled processes.

5.6.8.4 Plastic production with flue gas carbon capture

This process describes the production of plastics with flue gas carbon capture (Figure S71). The process is derived from the demands for conventional plastics production in Section 5.6.8.1. For the process with integrated CCS, there are additional demands for heat and electricity to capture carbon dioxide from the flue gas. These additional demands are based on the estimates used by Saygin et al. (2013) who assume chemical absorption (MEA) technology for the carbon-dioxide capture process with a CO_2 concentration of 11% in the flue gas. As outlined in Table S162, heat is assumed to be partially provided as waste process heat with the remainder produced by natural gas. This additional demand for natural gas increases the unabated combustion emissions per unit of plastic produced compared to the process in Section 5.6.8.1.

The chosen coefficients are shown in Table S163. These are derived based on the assumed energy demands and production shares shown in Table S154, and the assumptions in Table S162.

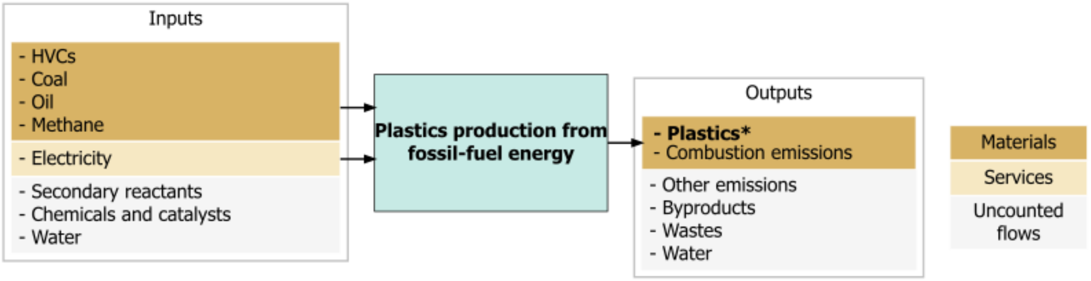

Figure S71: Inputs and outputs for plastics production using fossil-fuels to generate heat and steam with flue gas carbon capture.

Property	Quantity	Unit	Justification
**Summary of key assumptions**			
CO2 capture (CC) rate	90%		As assumed by Kuramochi et al. (2012) and Saygin et al. (2013)
Relative increase in fuel demands for CC	1.4		By iteration
Unabated emissions from fuel combustion	0.92	Gt CO2/Gt	As derived for the unabated fossil fuel delivery process, scaled by 1.4 to account for additional fuel for production of heat for CC process
Additional heat	3.0	EJ heat/Gt	3.6 GJ/t CO2 captured, as assumed by Saygin (2013)
Additional electricity	0.5	EJ/Gt	0.6 GJ/Gt CO2 captured, as assumed by Saygin (2013)
Unabated process electricity	1.5	EJ/Gt	As per unabated processes
Assumed heat from waste heat	50%		
Fuel demand for CC process heat	1.5	EJ/Gt	(1 - Waste heat share) x (Heat demand)
Unabated process methane demand	3.8	EJ/Gt	Steam and fuel demands for the unabated process
Relative increase in emissions for CC (to check assumptions)	1.4		(CCS process fuel demands)/(unabated process fuel demands)
**Calculated coefficients (inputs)**			
HVC feedstock demand	1.1	Gt HVCs/Gt	As per unabated processes
Total electricity demand	2.0	EJ/Gt	(Unabated demand) + (Additional demand for CC)
Methane demand	0.12	Gt/Gt H2	(Unabated demand) + (Additional demand for CC)
**Calculated coefficients (outputs)**			
Carbon storage	0.83	Gt CO2/Gt	(Capture rate) x (Unabated emissions)
Residual emissions	0.092	Gt CO2/Gt	(Unabated emissions)- (Carbon storage)

Table S162: Key assumptions for estimating the coefficients for plastics production using fossil-fuels to generate heat and steam with flue gas carbon capture (CC). Values for the conventional natural gas fuelled process are taken from Table S156. Fuel and emissions values are derived using the energy density values and emissions factors in the SI Part 2. Other references: Kuramochi et al. (2012), Saygin et al. (2013)

Activity	Process	Resource	Inputs	Outputs	Unit
Plastics	CCS	HVCs	-1.1		Gt/Gt
Plastics	CCS	Electricity	-2.0		EJ/Gt
Plastics	CCS	NetEmissions		0.092	Gt_CO2e/Gt
Plastics	CCS	CO2Product		0.83	Gt_CO2/Gt
Plastics	CCS	Methane	-0.12		Gt/Gt
Plastics	CCS	Plastics		1.0	Gt/Gt

Table S163: Chosen coefficients for plastics production using fossil-fuels and flue gas carbon capture.

5.6.8.5 Plastic production from recycled plastics

This process describes the production of plastics by mechanical recycling where all energy is from electricity. The coefficients are derived from the process energy data compiled by Meys et al. (2020) based on industry data for recycling of plastic packaging. The energy intensity of production of different plastic types are combined to estimate an overall demand for 'generic plastic', weighted by the total shares of plastics produced globally in 2015, as used by Zheng & Suh (2019). The total energy demand includes energy for the sub-processes: wet-processing pretreatment, extrusion, and waste-water treatment. Wet-processing pretreatment includes sorting, shredding, washing, and drying. Extrusion is the process of melting and forming the plastic into pellets. Emissions related to plastic recycling today are mostly indirect emissions due to electricity use (Uekert et al., 2023). In this model emissions related to electricity production are accounted for in that activity (Section 5.4 - Electricity Generation) so there are no emissions accounted for in this process. Data is shown in Table S164. The chosen coefficients are shown in Table S165.

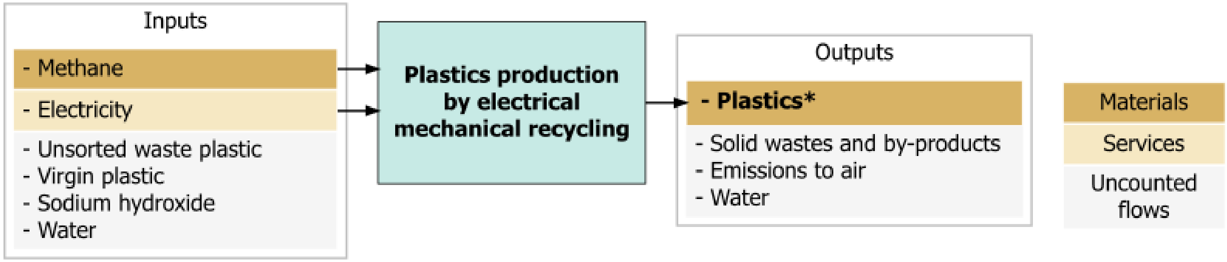

Figure S72: Inputs and outputs for the production of plastics by mechanical recycling where all energy is from electricity.

Plastic type	Acronym	Share of total plastics production	Share of recycled plastics	Energy for mechanical recycling (GJ/t plastic produced)
High-density polyethylene	HDPE	13%	21%	2.6
Low Density Poly-Ethylene	LDPE	16%	26%	3.5
Polypropylene	PP	17%	28%	2.6
Polyethylene Terephthalate	PET	8%	14%	2.3
Polystyrene	PS	6%	10%	2.6
**Total**		**59%**	**100%**	**2.8**

Table S164: Key data for estimating the coefficients for plastics production by mechanical recycling. Energy for mechanical recycling is based on the data compiled by Meys et al. (2020), assuming a yield of 0.8 kg product/kg waste for PET and 0.75 kg/kg for all other plastics. The share of production is based on the total shares of plastics produced globally in 2015, as used by Zheng & Suh (2019).

Activity	Process	Resource	Inputs	Outputs	Unit
Plastics	RecycleElec	Electricity	-2.8		EJ/Gt
Plastics	RecycleElec	Plastics		1.0	Gt/Gt

Table S165: Chosen coefficients for production of plastics by electrified recycling.

5.6.9 Production of Ammonia

This activity describes the production of ammonia, a key precursor to fertilisers and other chemical production. Ammonia is also proposed as a low carbon fuel for applications such as shipping. All processes are based on the Haber Bosch process, which uses a catalyst to react nitrogen and hydrogen at high temperatures and pressures:

$$N_2 + 3H_2 \leftrightharpoons 2NH_3$$

Other more novel methods for ammonia synthesis are not currently included in the model as they are all relatively immature (< TRL 4, according to The Royal Society (2020)). Examples include biological nitrogen fixation, electrochemical production directly from water and nitrogen, and chemical looping processes (The Royal Society, 2020). Existing conventional Haber Bosch plants produce ammonia using natural gas (50% globally), oil (31%) or coal (19%) as a feedstock for hydrogen production (Smith et al., 2020).

Activity	Delivery Process	Detail
Ammonia	SMR	Steam Methane Reforming and Haber Bosch process
Ammonia	SMRCCS	Steam Methane Reforming with Carbon Capture
Ammonia	CG	Ammonia from coal gasification
Ammonia	Elec	Ammonia from electrolysis of water

Table S166: Delivery Processes for Ammonia production.

5.6.9.1 Ammonia production from natural gas

This process describes ammonia production via steam methane reforming (SMR) and the Haber Bosch process. Fertilizers Europe (2000) and Smith et al. (2020) give detailed process descriptions, outlining the following steps: **(1)** Desulphurisation of natural gas; **(2)** Steam methane reforming (SMR) to produce synthesis gas (hydrogen and carbon monoxide); **(3)** Water gas shift reaction to convert carbon monoxide to carbon dioxide; **(4)** CO_2 removal and methanation (to avoid catalyst poisoning in the ammonia synthesis step); **(5)** Ammonia synthesis by the Haber Bosch process (catalytic reaction at high temperature and pressure).

The coefficients are derived from the data and assumptions listed in Table S167. The carbon dioxide removal value used is a Best Available Technology estimate. Current (2016) levels can be closer to 40% (Haugen et al., 2017), equivalent to 0.7 t CO_2/t. The global average for 2019 was 0.73 t/t (IEA, 2021a).

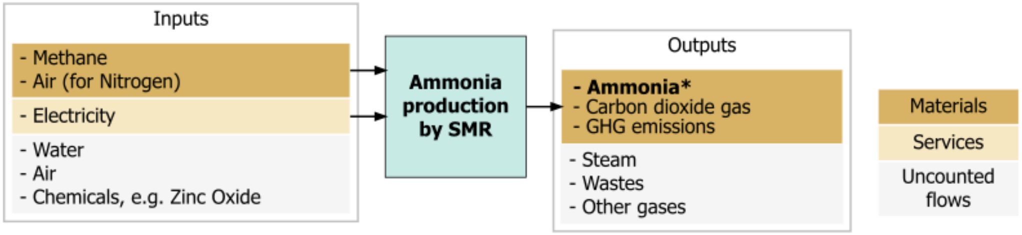

Figure S73: Inputs and outputs for ammonia production via SMR of Natural Gas.

Parameter, Unit	Quantity	Justification
**Summary of key data inputs**		
Feedstock, GJ/t	21	BPT value used by Saygin (2009)
Fuel, GJ/t	11	BPT value used by Saygin (2009)
Steam, GJ/t	-3.9	BPT value used by Saygin (2009)
Fuel energy density, GJ/t fuel	45	See document assumptions
Fuel emissions factor, t CO2/GJ fuel	0.060	See document assumptions
Process emissions, t CO2/t	1.0	From Levi & Cullen (2018)
Steam production efficiency	90%	See section assumptions
Exported steam use efficiency	80%	See section assumptions
Fuel credit for steam export, GJ/t	-3.47	Steam/Production efficiency x Use efficiency
Emissions from combustion, t CO2/t	0.45	Emissions factor x (Fuel + Steam credit)
Total CO2 production, t/t	1.4	Process emissions + combustion emissions
**Calculated coefficients (inputs)**		
Total methane demand t/t	0.63	(Fuel + Feedstock + Steam export credit)/Energy density
Electricity, GJ/t	0.30	BPT value used by Saygin (2009)
**Calculated coefficients (outputs)**		
Production line CO2 removed, t CO2/ t	1.2	Mid-range BAT value (Fertilizers Europe, 2000)
Residual emissions, t CO2/t	0.22	(Total CO2 production) - (Production line CO2 removed)

Table S167: Key assumptions for estimating the coefficients for ammonia production via SMR and Haber-Bosch. The treatment of steam exports is described in Section 5.6.1. Emissions factors and energy density values are taken from the SI Part 2. Other sources: Saygin et al. (2009), Levi & Cullen (2018), Fertilizers Europe (2000)

Activity	Process	Resource	Inputs	Outputs	Unit
Ammonia	SMR	Methane	-0.63		Gt/Gt
Ammonia	SMR	NetEmissions		0.22	Gt_CO2e/Gt
Ammonia	SMR	CO2Product		1.2	Gt/Gt
Ammonia	SMR	Electricity	-0.30		EJ/Gt
Ammonia	SMR	Ammonia		1.0	Gt/Gt

Table S168: Chosen coefficients for conventional ammonia production via SMR of Natural Gas.

5.6.9.2 Steam Methane Reforming (SMR) with flue gas carbon-dioxide capture

The process describes ammonia production by SMR and Haber-Bosch with flue gas carbon-dioxide capture. The coefficients are estimated based on the values used by Gao & Cabrera Serrenho (2023) for ammonia production with CCS in Europe for a study to assess emissions and reduction potential in global flows of ammonia. Key data and assumptions are shown in Table S169 and the coefficients are shown in Table S170.

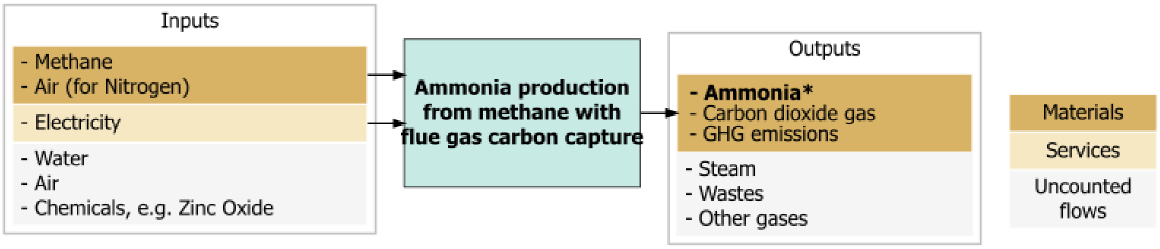

Figure S74: Inputs and outputs for ammonia production via SMR of Natural Gas and Haber Bosch with flue gas carbon-dioxide capture.

Parameter, Unit	Quantity	Justification
**Summary of key data and assumptions**		
Feedstock and fuel, GJ/t	34	Gao & Serrenho (2023)
Feedstock, GJ/t	21	BPT value used by Saygin (2009)
Fuel, GJ/t	13	(Feedstock and fuel) - (Feedstock)
Steam, GJ/t	-1.5	Gao & Serrenho (2023)
Fuel energy density, GJ/t fuel	45	See assumptions section
Fuel emissions factor, t CO2/GJ fuel	0.060	See assumptions section
Process emissions, t CO2/t	1.0	From Levi & Cullen (2018)
Production line CO2 removed, t CO2/ t	1.2	Mid-range BAT value (Fertilizers Europe, 2000)
Fraction of CO2 in flue gas captured	0.90	Gao & Serrenho (2023)
Steam production efficiency	90%	See section assumptions
Exported steam use efficiency	80%	See section assumptions
Fuel credit for steam export, GJ/t	-1.10	Steam/Production efficiency x Use efficiency
Emissions from combustion, t CO2/t	0.7	Emissions factor x (fuel consumption + steam credit)
CO2 in flue gas, tCO2/t	0.5	Total emissions - CO2 captured in conventional process
**Calculated coefficients (inputs)**		
Total methane demand t/t	0.73	(Fuel+Feedstock + Steam credit)/energy density
Electricity, GJ/t	0.85	Gao & Serrenho (2023)
**Calculated coefficients (outputs)**		
Captured CO2, tCO2/t	1.7	CO2 in flue gas x capture rate + conventional CO2 removed
Residual emissions, t CO2/t	0.05	(Total CO2 production) - (Production line CO2 removed)

Table S169: Key assumptions for estimating the coefficients for ammonia production by SMR and Haber Bosch with flue gas carbon-dioxide capture. The treatment of steam exports is described in Section 5.6.1. Emissions factors and energy density values are taken from the SI Part 2. Other sources: European production with CCS case from SI of Gao & Cabrera Serrenho (2023); Saygin et al. (2009); Levi & Cullen (2018); Fertilizers Europe (2000)

Activity	Process	Resource	Inputs	Outputs	Unit
Ammonia	SMRCCS	Methane	-0.73		Gt/Gt
Ammonia	SMRCCS	NetEmissions		0.051	Gt_CO2e/Gt
Ammonia	SMRCCS	CO2Product		1.7	Gt/Gt
Ammonia	SMRCCS	Electricity	-0.85		EJ/Gt
Ammonia	SMRCCS	Ammonia		1.0	Gt/Gt

Table S170: Chosen coefficients for ammonia production by SMR and Haber Bosch with carbon-dioxide capture.

5.6.9.3 Ammonia production from coal

This process describes ammonia production via coal gasification and the Haber Bosch process. Coal gasification (CG) produces about 20% of global ammonia production today (Saygin et al., 2009). The process can be summarised by the reactions given in the SI by Levi & Cullen (2018). The reactions produce carbon dioxide in high concentrations which is captured for use in other processes (mostly Urea). There are additional emissions arising from fuel combustion which are produced in a less concentrated stream and so not economical to capture in the conventional process.

The coefficients derived here are based on the chemical reactions and mass flows documented by Levi & Cullen (2018), the energy demands for BPT given by Saygin et al. (2009), and the quantity of CO_2 extracted consistent with the assumptions Gao & Cabrera Serrenho (2023). The assumption for carbon dioxide removal from this process is optimistic (high) compared to current rates; coal gasification contributes around 30% of production currently (Saygin et al., 2009) but the global average carbon dioxide capture from ammonia production is 0.7 t/t (IEA, 2021a), also lower than the value for production from SMR which made up the remaining 70% of production (Saygin et al., 2009).

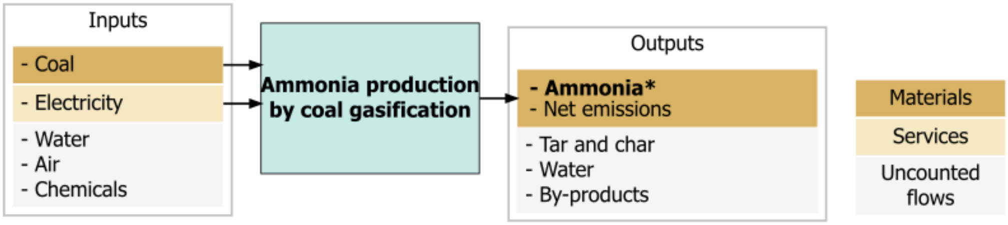

Figure S75: Inputs and outputs of ammonia production via coal gasification.

Parameter, Unit	Quantity	Justification
**Summary of key data and assumptions**		
Electricity, GJ/t	3.7	BPT value used by Saygin (2009)
Feedstock, GJ/t	21	BPT value used by Saygin (2009)
Fuel, GJ/t	17	BPT value used by Saygin (2009)
Steam, GJ/t	-1.3	BPT value used by Saygin (2009)
Process emissions, t CO2/t	3.2	From Levi & Cullen (2018)
Steam production efficiency	90%	See section assumptions
Exported steam use efficiency	80%	See section assumptions
Fuel credit for steam export, GJ/t	-1.16	Steam/Production efficiency x Use efficiency
Emissions from combustion, t CO2/t	1.6	Emissions factor x (Fuel consumption + Steam credit)
Total CO2 production, t/t	4.8	Process emissions + combustion emissions
**Calculated coefficients (inputs)**		
Total coal demand t/t	1.3	(Fuel + Feedstock + Steam export credit)/Energy density
**Calculated coefficients (outputs)**		
CO2 removed, t CO2/ t	2.6	From conventional production line (Gao & Serrenho, 2023)
Residual emissions, t CO2/t	2.3	(Total CO2) - (Production line CO2 removed)

Table S171: Key assumptions for estimating the coefficients for ammonia production via coal gasification. The treatment of steam exports is described in Section 5.6.1. Emissions factors and energy density values are taken from the SI Part 2. Other sources: Saygin et al. (2009); Levi & Cullen (2018); Gao & Cabrera Serrenho (2023)

Activity	Process	Resource	Inputs	Outputs	Unit
Ammonia	CG	Coal	-1.3		Gt/Gt
Ammonia	CG	NetEmissions		2.3	Gt_CO2e/Gt
Ammonia	CG	CO2Product		2.6	Gt/Gt
Ammonia	CG	Electricity	-3.7		EJ/Gt
Ammonia	CG	Ammonia		1.0	Gt/Gt

Table S172: Chosen coefficients for conventional ammonia production via Coal Gasification.

5.6.9.4 Ammonia by electrolysis

This process describes ammonia production by electrolysis. Ammonia is formed by the Haber-Bosch process (see page S196) with hydrogen feedstock produced by electrolysis of water, and nitrogen by electrically powered air separation. The mass demand for hydrogen and nitrogen are derived from a stochiometric mass balance. The electricity demands are the sum of energy for: *(1)* electrolysis of water to produce hydrogen; *(2)* air separation and purification to produce nitrogen; and *(3)* ammonia synthesis.

Nitrogen is abundant in the atmosphere but energy is required to separate it from air. Commercial technologies to achieve this include cryogenic distillation, pressure swing adsorption, membrane permeation, and hydrocarbon combustion processes (Hardenburger & Ennis, 2005). Cryogenic distillation is the most cost-effective technology for production of large quantities of relatively pure nitrogen and is the most commonly used, the value used here is based on this technology. Pressure swing adsorption and membrane permeation are the most economical processes for production of lower purity nitrogen in low to moderate volume ranges. Nitrogen purification requires around 0.3-2 GJ/tNH_3 (Rouwenhorst et al., 2021). The value used for nitrogen separation here is within that range (as shown in Table S173.)

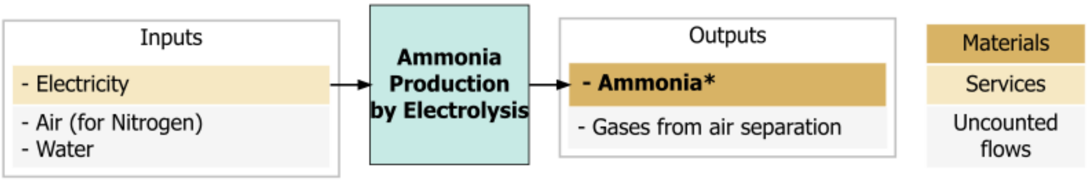

Figure S76: Inputs and outputs for ammonia production via electrolysis of water.

Parameter	Quantity	Unit	Justification
**Summary of key data and assumptions**			
Ammonia mass (NH3)	17	g/mol	From atomic masses
H2 mass	2	g/mol	From atomic masses
N2 mass	28	g/mol	From atomic masses
Electricity intensity of H2 production by electrolysis	170	GJ/t	As used for hydrogen by electrolysis. N.B. Gao & Cabrera Serrenho (2023) use 190 GJ/t
Electricity for N2 from Cryogenic distillation	0.58	GJ/ t N2	Gao & Cabrera Serrenho (2023)
Electricity for NH3 synthesis and compression	1.17	GJ/ t	Gao & Cabrera Serrenho (2023)
**Calculated values**			
Hydrogen stochiometric input	0.18	Gt H2/Gt	Haber-Bosch reaction
Nitrogen stochiometric input	0.82	Gt N2/Gt	Haber-Bosch reaction
Electricity for H2 production	30.00	GJ/ t NH2	Electricity intensity x stochiometric input
Electricity for N2 synthesis	0.48	GJ/ t	Electricity intensity x stochiometric input
**Calculated coefficients (inputs)**			
Total electricity demand	31.64	GJ/ t	Sum of electricity for H2 and N2 production and NH3 synthesis

Table S173: Key assumptions for estimating the coefficients for ammonia production via electrolysis of water. References: Section 5.6.5.7; Gao & Cabrera Serrenho (2023)

Activity	Process	Resource	Inputs	Outputs	Unit
Ammonia	Elec	Electricity	-32		EJ/Gt
Ammonia	Elec	Ammonia		1.0	Gt/Gt

Table S174: Chosen coefficients for ammonia production from electrolysis to produce hydrogen.

5.6.10 Urea Production

This activity describes production of urea (also known as carbamide, $CO(NH_2)_2$). No other fertilisers are currently included in the model and so urea is assumed to represent all inorganic fertilizer production - see 5.5.2.3.

The process is based on two equilibrium reactions, where the reaction heat from the first reaction drives the second (Meessen, 2014). The reactions are:

1. Carbamate formation: fast exothermic reaction at high temperature and pressure to form ammonium carbamate:

$$2NH_3 + CO_2 \Longleftrightarrow H_2NCO_2NH_4$$

2. Urea conversion: the slower endothermic decomposition of ammonium carbamate:

$$H_2NCO_2NH_4 \Longleftrightarrow (NH_2)_2CO + H_2O$$

Conditions that most favour carbamate formation have an unfavourable effect on the urea conversion equilibrium and so a "stripping concept" is used (addition of CO_2 to the products and unreacted chemicals) (Meessen, 2014).

Urea production coefficients are based on the fuel demands used by Saygin et al. (2009), and feedstock mass requirements are based on stochiometric mass balances. The carbon dioxide is likely to be provided by post-combustion capture at an adjacent ammonia plant. As such, carbon dioxide is treated as an input to the process or a reduction in the system-level demand for carbon storage. The emissions will be subsequently re-released during use and are accounted for in agricultural processes (Section 5.5).

Two processes are modelled for urea production: one using natural gas to produce steam, and another where all energy is provided by electricity (Table S175). The coefficients for each process are given in the remainder of this section.

Activity	Process	Process Long Name
Urea	Gas	Natural gas fuelled steam production
Urea	Elec	Electrical fuelled steam production

Table S175: Delivery processes for this activity. The coefficients for each delivery process are given and explained in the remainder of this section.

5.6.10.1 Urea Production, fuelled by natural gas

This process describes urea production, using natural gas to produce steam. The coefficients are derived from the data and assumptions listed in Table S176.

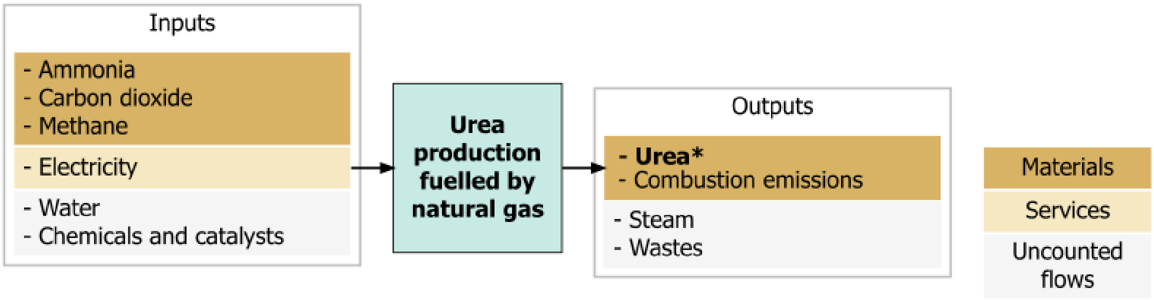

Figure S77: Inputs and outputs for urea production

Parameter	Quantity	Unit	Justification
**Summary of key data and assumptions**			
Steam demand	2.2	EJ Steam/Gt Urea	Saygin et al. assumptions (2009)
Steam production efficiency	0.90	EJ Steam/EJ Methane	Natural gas to steam. Same assumption as used by Paoli, Lupton & Cullen (2018)
Methane energy density	45	GJ/t Methane	See assumptions section
Methane emissions factor	0.060	t CO2/ GJ Methane	See assumptions section
**Calculated coefficients (inputs)**			
Ammonia	0.57	Gt/ Gt Urea	By stochiometric mass balance: 2 mol of Ammonia for 1 mol Urea
Carbon dioxide	0.73	Gt/ Gt Urea	By stochiometric mass balance: 1 mol of CO2 for 1 mol Urea
Electricity	0.30	GJ/t Urea	Saygin et al. assumptions (2009)
Methane	0.054	t Methane/t Urea	steam demand/production efficiency/energy density
**Calculated coefficients (outputs)**			
Residual emissions	0.15	t CO2e/t Urea	methane demand x emissions factor

Table S176: Key data and assumptions for estimating the coefficients for urea production, where steam is produced by natural gas. The treatment of steam exports is described in Section 5.6.1, emissions factors and energy density values are taken from the SI Part 2. Other sources: Saygin et al. (2009); Paoli et al. (2018)

Activity	Process	Resource	Inputs	Outputs	Unit
Urea	Gas	Ammonia	-0.57		Gt_Ammonia/Gt
Urea	Gas	Electricity	-0.054		EJ/Gt
Urea	Gas	CO2Product	-0.73		Gt_CO2/Gt
Urea	Gas	Methane	-0.054		Gt/Gt
Urea	Gas	NetEmissions		0.15	Gt_CO2e/Gt
Urea	Gas	Urea		1.0	Gt/Gt

Table S177: Coefficients for urea production, where steam is produced by natural gas.

5.6.10.2 Electrified Urea Production

This process describes urea production, using electricity to produce steam. The coefficients are derived from the data and assumptions in Table S178.

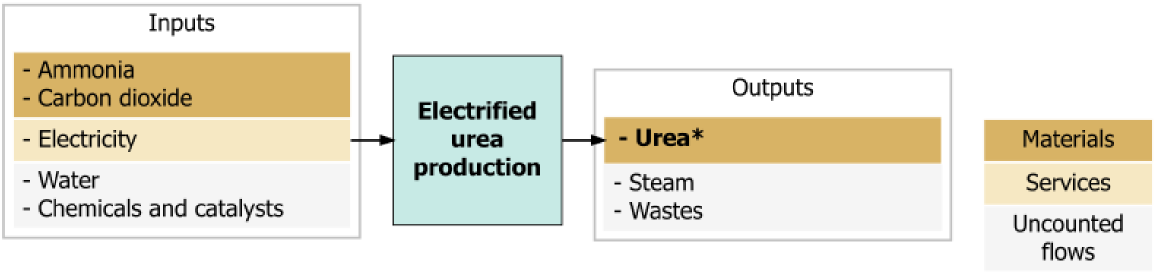

Figure S78: Inputs and outputs for urea production

Parameter	Quantity	Unit	Justification
**Summary of key data and assumptions**			
Electricity process demand	0.3	EJ/Gt Urea	Saygin et al. assumptions (2009)
Steam demand	2.2	EJ Steam/Gt Urea	Saygin et al. assumptions (2009)
Steam production efficiency	1.00	EJ Steam/EJ Elec	Assumed electricity required for steam production.
**Calculated coefficients (inputs)**			
Ammonia	0.57	Gt/ Gt Urea	By stochiometric mass balance: 2 mol of Ammonia for 1 mol Urea
Carbon dioxide	0.73	Gt/ Gt Urea	By stochiometric mass balance: 1 mol of CO2 for 1 mol Urea
Electricity	2.5	EJ/Gt Urea	Steam and electricity

Table S178: Assumptions used to derive coefficients for fully electrified urea production.

Activity	Process	Resource	Inputs	Outputs	Unit
Urea	Elec	Ammonia	**-0.57**		Gt/Gt
Urea	Elec	Electricity	**-2.5**		EJ/Gt
Urea	Elec	CO2Product	**-0.73**		Gt_CO2/Gt
Urea	Elec	Urea		**1.0**	Gt/Gt

Table S179: Coefficients for fully electrified urea production.

5.6.11 Synthetic Fuel Production

This activity describes the production of Synfuel, synthetic hydrocarbon fuels, produced from combining carbon dioxide or carbon monoxide with hydrogen. They can be produced from air, water and electricity (if carbon dioxide is obtained by direct air capture (Section 5.11.1.1) and hydrogen from electrolysis of water). For this reason, synfuel is also sometimes referred to as *Power-to-Liquid (PtL) fuels* or *electro-fuels (e-fuels)*. Synfuel can be used as a drop-in replacement fuel and so is a prominent proposal for the aviation industry. In that context it is considered an example of *Sustainable Aviation Fuels* (SAFs)[4]. The term *synthetic fuel* may also be used to describe liquid or gaseous fuels produced from coal, other fossil-fuels or biomass - these are not explicitly included within the activity described here. Although synthetic fuels may be produced for a variety of applications, the coefficients have been derived on the assumption that the resource demands are similar.

In general, synthetic hydrocarbons are produced by (a) combining a source of carbon with hydrogen to form long chain hydrocarbons (b) "upgrading" these into usable fuels. Although various routes have been proposed, there is only one process currently modelled, using the Fischer-Tropsch process to combine Hydrogen, with carbon monoxide (CO) from CO_2. Synthesis using methanol as an intermediary is another option but based on the breakdown of cost estimates given by the German Environment Agency (2020) for the two routes, there is not likely to be much difference in terms of energy demands. Transport and distribution of fuel has not been accounted for here since these are similar to conventional methods and so would be accounted for within the transport sector. Isolating these demands would add additional uncertainties.

5.6.11.1 Synfuel production via Fischer Tropsch Synthesis

This process describes the production of synfuel via Fischer-Tropsch (FT) synthesis (Figure S80). The coefficients are derived from the data and assumptions listed in Table S180. The chosen coefficients are shown in Table S181. The coefficients are based on the life cycle analysis by Van Der Giesen et al. (2014).

Fischer-Tropsch synthesis is a mature commercial process which converts a mixture of hydrogen and carbon monoxide (known as syngas) into a mixture of hydrocarbons. It is based on an exothermic reaction, requiring a catalyst.

Van Der Giesen et al. (2014) assume that a Reversed Water Gas Shift (RWGS) reaction is used to convert Carbon Dioxide to Carbon Monoxide (CO) which is blended with Hydrogen to form Syngas (the input to FT synthesis). The same assumptions are used here for the formation of CO:

- Reaction conditions: 2.5 MPa, 230 °C;
- Excess heat from the FT process supports the endothermic equilibrium reaction;
- 1 kg of carbon monoxide requires inputs of: 1.57 kg of carbon dioxide and 0.07 kg of hydrogen.

The assumptions for the FT reaction are also taken as those used by Van Der Giesen et al. (2014):

- Reaction conditions: 2.5 MPa, 230 °C;
- Reaction efficiency (energy conversion from syngas to fuel): 80%, with the remaining 20% used to generate energy for the plant.

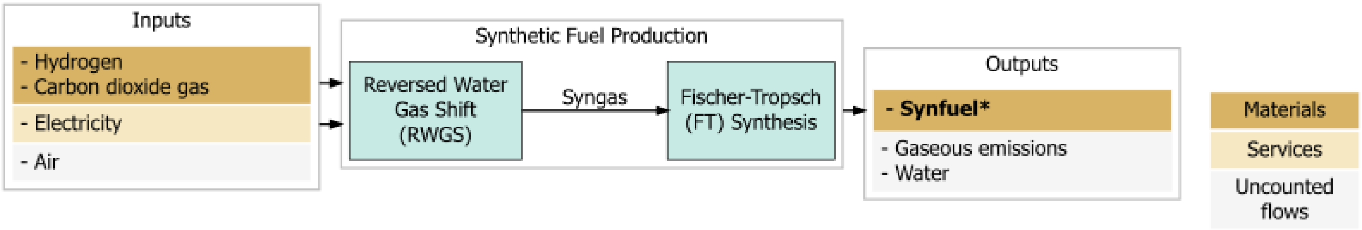

Figure S80: Inputs and outputs for Synfuel production via Fischer Tropsch Synthesis.

[4] SAFs include both bio-based aviation fuels (which are accounted for under biofuels in Section 5.6.12) and synfuels (described in this section).

Parameter	Quantity	Unit	Justification
**Summary of key data and assumptions**			
CO2 input for RWGS	1.57	kg CO2/ kg CO	As assumed by Van Der Giesen et al. (2014)
Hydrogen input for RWGS	0.07	kg H2/ kg CO	As assumed by Van Der Giesen et al. (2014)
Additional energy input for RWGS	0		As assumed by Van Der Giesen et al. (2014), the process is fulled entirely by waste heat from FT reaction
Syngas input to FT reaction	2.3	kg syngas/ kg synfuel	As assumed by Van Der Giesen et al. (2014)
CO input to Syngas	88%		Based on atomic masses, assuming syngas composition of 2H2 + CO
H input to Syngas	13%		Based on atomic masses, assuming syngas composition of 2H2 + CO
CO input to FT reaction	2.0	kg/ kg synfuel	Mass share of assumed syngas input
H input to FT reaction	0.29	kg/ kg synfuel	Mass share of assumed syngas input
Additional energy input to FT reaction	0		As assumed by Van Der Giesen et al. (2014), the process is 80% efficient with all processes fuelled by the input Syngas
**Calculated coefficients (inputs)**			
CO2 input for Synfuel production	3.2	kg/ kg synfuel	CO2 input for RWGS x CO input for FT
H2 input for Synfuel production	0.43	kg/ kg synfuel	H2 FT input + (H2 RWGS input x CO FT input)

Table S180: Assumptions used to derive coefficients for SynFuel production Fischer Tropsch Synthesis. RGWS: Reversed Water Gas Shift reaction; FT: Fischer Tropsch reaction. References: Van Der Giesen et al. (2014)

Activity	Process	Resource	Inputs	Outputs	Unit
Synfuel	FT	CO2Product	**-3.2**		Gt_CO2/Gt
Synfuel	FT	Hydrogen	**-0.43**		Gt/Gt
Synfuel	FT	Synfuel		**1.0**	Gt/Gt

Table S181: Coefficients for SynFuel production via Fischer Tropsch Synthesis

5.6.12 Biofuel Production

This activity describes production of liquid biofuels from biomass. There are a wide range of biofuels but they are often grouped into three generations:

- First generation biofuels generally include sugarcane ethanol, corn ethanol, oilseed rape biodiesel and palm oil biodiesel (Sims et al., 2008). These have mature commercial markets (although limited to around 2% of transport fuels) and are well-understood technologies. Although first generation biofuels may also include biomethane (as land-fill gas or from fermentaion of organic waste), biomethane is not considered in this section as it is covered in Sections 5.6.4 and 5.10. There are concerns that first generation biofuels compete with food production.
- Second generation biofuels have been proposed to overcome this limitation although there is no guarantee that these would avoid land-use change. Second generation biofuels use non-food ligno-cellulosic feedstocks: these may be from agricultural and forest residues, or from specialist energy crops (Sims et al., 2008). Energy crops are likely to have high GJ/ha yields than food crops and could be grown on lower quality lands. These are relatively immature technologies but, as such, their production efficiencies have more potential to improve than first generation processes.
- Third generation biofuels use marine biomass as the feedstock. These are not included here because there have been concerns raised about the scalability of this route and other environmental impacts of harvesting algal biomass (as described in the Analysis Framework SI Document).

Three delivery processes are quantified to produce biofuels, considering first and second generation fuels, as listed in Table S182. Land use change (direct and indirect) is not explicitly accounted in the model, as explained and justified in the SI Part 1, Section 3. The coefficients for each delivery process are given in the remainder of this section.

Activity	Process	Process Long Name
BioFuel	BioDiesel	Conventional (1st generation) bio-diesel from farmed biomass
BioFuel	BioEthanol	Conventional (1st generation) bio-ethanol from farmed biomass
BioFuel	AdvBioFuel	Advanced (2nd generation) biofuels - based on the process for cellulosic ethanol by thermochemical methods

Table S182: Delivery processes for this activity. The coefficients for each delivery process are given and explained in the remainder of this section.

5.6.12.1 Conventional bio-diesel

This delivery process descibes the production of biofuel from a feedstock of farmed biomass (Figure S81). The process is based on the process flows for Production of Biodiesel from Oilseed Rape, compiled for a study by Sheffield Hallam University and Forest Research for the UK Department of Trade and Industry (Elsayed et al., 2003). The process produces by-products of glycerine and rape meal. Since these have further down-stream uses (for instance in the food/pharmaceuticals industry, and as animal feed, respectively), their mass is subtracted from the demand for biomass to avoid double-counting demand for primary biomass. Emissions produced during biomass production are accounted for in the production process. Only emissions from fossil-fuel use are accounted for in this process. Key data and assumptions used to compile the coefficients are listed in Table S183. The chosen coefficients are shown in Table S184.

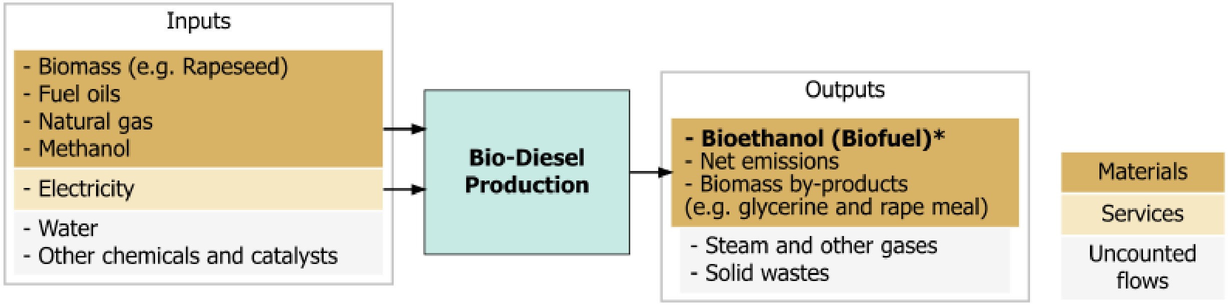

Figure S81: Inputs and outputs for biodiesel production.

Parameter	Quantity	Unit	Justification
**Assumptions**			
Energy density methane	45	GJ/t	Model assumptions
Emissions factor methane	60	kg CO2/GJ	Model assumptions
Energy density oil	42	GJ/t	Model assumptions
Emissions factor oil	70	kg CO2/GJ	Model assumptions
**Mass flows**			
Dry rapeseed feedstock	2.7	t/t Biodiesel	Elsayed et al. (2003)
Methanol (for esterification)	0.21	MJ/t Biodiesel	Elsayed et al. (2003)
By-product: Glycerine	0.10	t/t Biodiesel	Elsayed et al. (2003)
By-product: Rape meal	1.6	t/t Biodiesel	Elsayed et al. (2003)
**Energy flows: drying biomass & solvent extraction**			
Oil	510	MJ/t Biodiesel	Elsayed et al. (2003)
Electricity	204	MJ/t Biodiesel	Elsayed et al. (2003)
Natural gas	1512	MJ/t Biodiesel	Elsayed et al. (2003)
**Energy flows: refining**			
Electricity	10	MJ/t Biodiesel	Elsayed et al. (2003)
Natural gas	163	MJ/t Biodiesel	Elsayed et al. (2003)
Oil	157	MJ/t Biodiesel	Elsayed et al. (2003)
**Energy flows: esterification**			
Electricity	72	MJ/t Biodiesel	Elsayed et al. (2003)
Natural gas	1218	MJ/t Biodiesel	Elsayed et al. (2003)
Oil	280	MJ/t Biodiesel	Elsayed et al. (2003)
**Calculated coefficients (inputs)**			
Allocated biomass	1.0	t/ t	(Biomass input) - (By-products produced)
Electricity	0.29	GJ/t Biodiesel	Total electricity
Natural gas	0.064	t/t Biodiesel	Total fuel / Energy density
Oil	0.010	t/t Biodiesel	Total fuel / Energy density
**Calculated coefficients (outputs)**			
Emissions	0.20	t CO2e/t Biodiesel	For each fuel: (Total fuel) x (Emissions factor)

Table S183: Key data and assumptions used to derive coefficients for biodiesel production, taken from the mass and energy flows for production of biodiesel from oilseed rape, compiled for a study by Sheffield Hallam University and Forest Research for the UK Department of Trade and Industry (Elsayed et al., 2003).

Activity	Process	Resource	Inputs	Outputs	Unit
BioFuel	BioDiesel	NonWoodBiomass	**-1.0**		Gt_DryBio/Gt
BioFuel	BioDiesel	Oil	**-0.010**		Gt/Gt
BioFuel	BioDiesel	Electricity	**-0.29**		EJ/Gt
BioFuel	BioDiesel	Methane	**-0.064**		Gt/Gt
BioFuel	BioDiesel	Methanol	**-0.21**		Gt/Gt
BioFuel	BioDiesel	NetEmissions		**0.20**	Gt_CO2e/Gt
BioFuel	BioDiesel	BioFuel		**1.0**	Gt/Gt

Table S184: Chosen coefficients for conventional bio-diesel production.

5.6.12.2 Conventional bio-ethanol

This process describes conventional (1st Generation) ethanol production from a feedstock of farmed biomass (Figure S82). The process coefficients are based on process flows for ethanol production from sugar beet, compiled for a study by Sheffield Hallam University and Forest Research for the UK Department of Trade and Industry (Elsayed et al., 2003). The process also produces a by-product stream which is conventionally used as animal feed. It is assumed that this is all usefully used and so is subtracted from the demand for farmed biomass. Emissions produced during biomass production are accounted for in the production process. Only emissions from fossil-fuel combustion are accounted for in this process; although the process produces carbon-dioxide by fermentation, the carbon is from a biogenic source and assumed to be compensated by carbon sequestered in biomass growth, as described in the SI Part 1. Key data and assumptions used to compile the coefficients are listed in Table S185. The chosen coefficients are shown in Table S186.

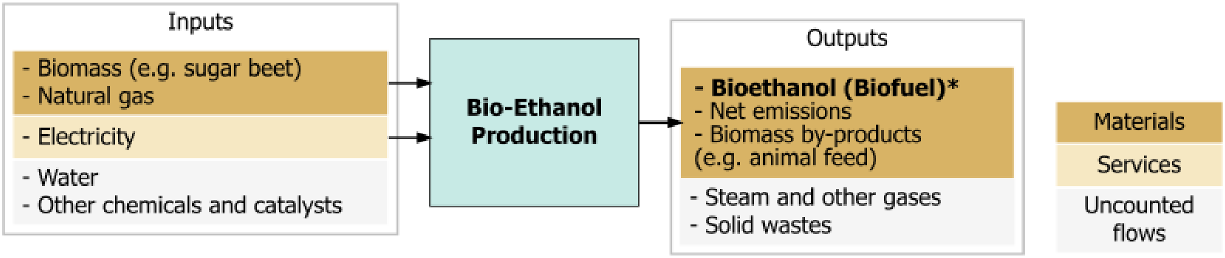

Figure S82: Inputs and outputs for bioethanol production.

Parameter	Quantity	Unit	Justification
**Assumptions**			
Energy density methane	45	GJ/t	Model assumptions
Emissions factor methane	60	kg CO2/GJ	Model assumptions
Moisture content of sugar beet	82%		Babu et al. (2024): ”Sugar beet contains about 75%–86% of moisture”
**Mass flows**			
Feedstock: clean sugar beet (wet mass)	12.9	t/t	Elsayed et al. (2003)
By-product: Animal feed (dry weight)	0.68	t/t	Elsayed et al. (2003): 0.57 t feed, 9% moisture content
**Energy flows: Biomass processing**			
Electricity	219	MJ/t	Elsayed et al. (2003)
**Energy flows: Diffusion & distillation**			
Electricity	181	MJ/t	Elsayed et al. (2003)
**Energy flows: Pasturisation, fermentation & distillation**			
Natural gas for steam generation	9217	MJ/t	Elsayed et al. (2003) steam demand and assumed boiler efficiency of 90% as within the range of 84-91% given by Paoli & Cullen (2018)
**Calculated coefficients (inputs)**			
Electricity	0.40	GJ/t	Total electricity
Natural gas	0.20	t/t	Total fuel / Energy density
Allocated biomass	1.6	t/ t	[Wet biomass input x (1 - Moisture content)] - (By-products produced, dry)
**Calculated coefficients (outputs)**			
Emissions	0.55	t CO2e/t	Fuel x Emissions factor

Table S185: Key data and assumptions used to derive coefficients for bioethanol production, taken from the mass and energy flows for production of bioethanol from sugar beet, compiled for a study by Sheffield Hallam University and Forest Research for the UK Department of Trade and Industry (Elsayed et al., 2003). Carbon-dioxide from fermentation is not accounted in this process because the carbon is from a biogenic source - these emissions are assumed to be compensated by carbon sequestered in biomass growth, as described in the SI Part 1.

Activity	Process	Resource	Inputs	Outputs	Unit
BioFuel	BioEthanol	NonWoodBiomass	-1.6		Gt_DryBio/Gt
BioFuel	BioEthanol	Electricity	-0.40		EJ/Gt
BioFuel	BioEthanol	Methane	-0.20		Gt/Gt
BioFuel	BioEthanol	NetEmissions		0.55	Gt_CO2e/Gt
BioFuel	BioEthanol	BioFuel		1.0	Gt/Gt

Table S186: Chosen coefficients for conventional bio-ethanol production.

5.6.12.3 Advanced biofuels - lignocellulosic ethanol production

The process describes production of advanced biofuels (Figure S83). The process is based on Lignocellulosic Ethanol production, compiled for a study by Sheffield Hallam University and Forest Research for the UK Department of Trade and Industry (Elsayed et al., 2003). The cellusosic feedstock could be straw, corn stover, bagasse, or wood but is considered here to be wood biomass only. Wood biomass is produced by delivery processes in the AFLUC Section (Section 5.5).

The process defined here also includes flows of sulphuric acid, lime, acetic acid and ash which are not accounted for in the model. The demand for sulphuric acid and lime are significant and would increase the overall resource demands. Unfermentables and lignin wastes from the process are assumed to be burned to generate electricity (following the process documented by Elsayed et al. (2003)). Emissions produced during biomass production are accounted for in the production process. Only emissions from fossil-fuel combustion are accounted for in this process; although the process produces carbon-dioxide by fermentation, the carbon is from a biogenic source and assumed to be compensated by carbon sequestered in biomass growth, as described in the SI Part 1.

Additional processing would be needed in reality to upgrade this fuel into the longer-chain hydrocarbons needed for aviation or marine applications. This is not accounted for in the model at this stage.

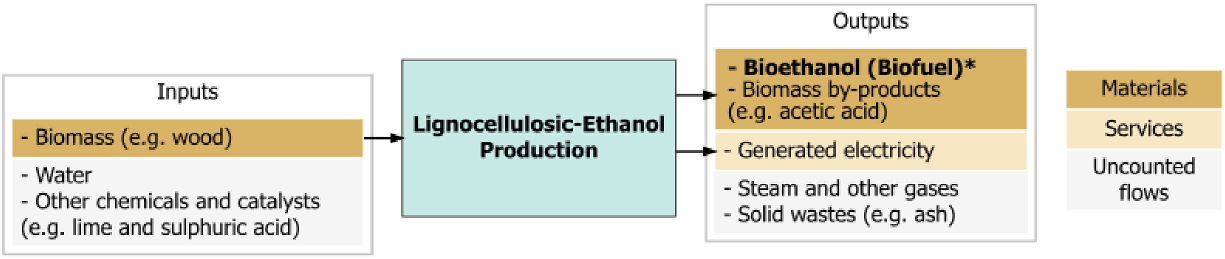

Figure S83: Inputs and outputs for lignocellulosic ethanol production.

Activity	Process	Resource	Inputs	Outputs	Unit
BioFuel	AdvBioFuel	Wood	-3.5		Gt_DryBio/Gt
BioFuel	AdvBioFuel	Electricity		1.8	EJ/Gt
BioFuel	AdvBioFuel	BioFuel		1.0	Gt/Gt

Table S187: Chosen coefficients for advanced bio-fuels production based on lignocellulosic ethanol production with waste products combusted to produce process energy and electricity.

Parameter	Quantity	Unit	Justification
**Assumptions**			
Energy density methane	45	GJ/t	Model assumptions
Emissions factor methane	60	kg CO2/GJ	Model assumptions
Energy density oil	42	GJ/t	Model assumptions
Emissions factor oil	70	kg CO2/GJ	Model assumptions
**Mass flows**			
Feedstock: Dry wheat straw	3.6	t/t	Elsayed et al. (2003)
Input: Sulphuric Acid	0.036	t/t	Elsayed et al. (2003): 10 kg/t dry wheat straw
Input: Lime	0.036	t/t	Elsayed et al. (2003): 10 kg/t dry wheat straw
By-product/waste: Ash	0.38	t/t	Elsayed et al. (2003)
By-product: Acetic acid	0.12	t/t	Elsayed et al. (2003)
**Calculated coefficients (inputs)**			
Allocated biomass	3.5	t/t	(Feedstock mass) - (By-product mass)
**Calculated coefficients (outputs)**			
Electricity generated from combustion of lignin and unfermentable by-products	1.8	GJ/t	Elsayed et al. (2003)

Table S188: Key data and assumptions used to derive coefficients for lignocellulosic ethanol production, taken from the mass and energy flows for production of lignocellulosic ethanol from wheat straw, compiled for a study by Sheffield Hallam University and Forest Research for the UK Department of Trade and Industry (Elsayed et al., 2003). Carbon-dioxide from fermentation is not accounted in this process because the carbon is from a biogenic source - these emissions are assumed to be compensated by carbon sequestered in biomass growth, as described in the SI Part 1. Although the data is taken from a process using wheat straw feedstock, it is assumed here that using a feedstock of wood biomass would give similar coefficients.

5.6.13 Production of Other Petrochemicals

This activity describes the production of all other petrochemicals that are not otherwise accounted for (Figure S47).

In the absence of a more detailed understanding of “other petrochemical products”, these have been treated as a generic product which requires feedstock of ammonia, methanol and HVCs. As a crude approximation, based on the map compiled by Levi & Cullen (2018), the yield is assumed to be 100% with feedstock split given by the proportions of flows from the SI of Levi & Cullen (2018).

The energy intensity is assumed to be relatively small compared to other chemical products since it’s assumed that most of the energy intensive reactions have already taken place in the formation of primary chemicals. Energy inputs for these processes are therefore not accounted for.

Activity	Process	Resource	Inputs	Outputs	Unit
OtherPetChem	NET	Ammonia	**-0.17**		Gt/Gt
OtherPetChem	NET	Methanol	**-0.22**		Gt/Gt
OtherPetChem	NET	HVCs	**-0.58**		Gt/Gt
OtherPetChem	NET	Urea	**-0.030**		Gt/Gt
OtherPetChem	NET	OtherPetChem		**1.0**	Gt/Gt

Table S189: Chosen coefficients for production of other petrochemical products which are not accounted for elsewhere.

5.6.14 Coefficient Summary

ResourceFlow	Ammonia__Elec	Ammonia__SMR	Ammonia__SMRCCS	Ammonia__CG	BioFuel__BioDiesel	BioFuel__BioEthanol	BioFuel__AdvBioFuel	HVCs__NET	HVCs__CCS	HVCs__MTOA	HVCs__BioEth	Hydrogen__ATR	Hydrogen__ATRCCS	Hydrogen__CG	Hydrogen__CGCCS	Hydrogen__Elec	Hydrogen__SMR	Hydrogen__SMRCCS
Electricity, EJ	-32	-0.30	-0.85	-3.7	-0.29	-0.40	1.8	-0.21	-0.50	-0.20	-0.80	-8.5	-13	-20	-28	-170	-3.5	-16
Ammonia, Gt	1.0	1.0	1.0	1.0														
Methane, Gt		-0.63	-0.73		-0.064	-0.20		-0.027	-0.048	-0.094	-0.061	-3.3	-3.3				-4.0	-5.6
NetEmissions, Gt__CO2e		0.22	0.051	2.3	0.20	0.55		0.52		0.25	0.16	8.4	0.71	17	2.5		9.2	2.0
CO2Product, Gt		1.2	1.7	2.6											15			
Coal, Gt				-1.3				-0.038	-0.037					-6.6	-8.9			
NonWoodBiomass, Gt__DryBio					-1.0	-1.6												
Oil, Gt					-0.010			-1.0	-1.0									
Methanol, Gt					-0.21					-3.1	-2.0							
BioFuel, Gt					1.0	1.0	1.0											
Wood, Gt__DryBio							-3.5											
HVCs, Gt								1.0	1.0	1.0	1.0							
NetEmissions, Gt__CO2									0.056									
CO2Product, Gt__CO2									0.50				7.7					11
NonWoodBiomass, Gt											-2.3							
Hydrogen, Gt												1.0	1.0	1.0	1.0	1.0	1.0	1.0
Hydrogen, Gt__H2																		
ExtractedOilGas, Gt																		
Urea, Gt																		
OtherPetChem, Gt																		
Plastics, Gt																		
Synfuel, Gt																		
Ammonia, Gt__Ammonia																		

Table S190: Coefficients for production of substances associated with the conventional petrochemicals sector (fuels, feedstocks and chemical-based products). Part 1 of 2

ResourceFlow	Methane__SynDAC	Methane__Biogas	Methane__FF	Methanol__Coal	Methanol__SMR	Methanol__CCS	Methanol__FromH2	Methanol__BioGas	Oil__FF	OtherPetChem__NET	Plastics__NET	Plastics__Bio	Plastics__Elec	Plastics__RecycleElec	Plastics__CCS	Synfuel__FT
Electricity, EJ	-1.2	-1.5		-0.69	-0.30	-0.90	-1.6		-0.27		-1.5	-1.5	-8.3	-2.8	-2.0	
Ammonia, Gt										-0.17						
Methane, Gt	1.0	1.0	1.0		-0.67	-0.69			-0.016		-0.015				-0.12	
NetEmissions, Gt__CO2e			0.057		0.60	0.066		-1.4	0.64		0.66				0.092	
CO2Product, Gt																
Coal, Gt				-1.3							-0.13					
NonWoodBiomass, Gt__DryBio		-2.0						-3.2				-0.54				
Oil, Gt									1.0		-0.082					
Methanol, Gt				1.0	1.0	1.0	1.0	1.0		-0.22						
BioFuel, Gt																
Wood, Gt__DryBio																
HVCs, Gt										-0.58	-1.1	-1.1	-1.1		-1.1	
NetEmissions, Gt__CO2		-2.8		4.3												
CO2Product, Gt__CO2	-2.8					0.59	-1.5								0.83	-3.2
NonWoodBiomass, Gt																
Hydrogen, Gt	-0.50						-0.20		-0.0094							-0.43
Hydrogen, Gt__H2		-0.25														
ExtractedOilGas, Gt			-1.0						-1.1							
Urea, Gt										-0.030						
OtherPetChem, Gt										1.0						
Plastics, Gt											1.0	1.0	1.0	1.0	1.0	
Synfuel, Gt																1.0
Ammonia, Gt__Ammonia																

Table S191: Coefficients for production of substances associated with the conventional petrochemicals sector (fuels, feedstocks and chemical-based products). Part 2 of 2

5.7 Industry

This section outlines the derivation of coefficients for production of materials and goods in industry, and for infrastructure construction. Industry is grouped into the activities shown in Table S192. These are mostly the production of the bulk materials which have the largest energy demands and emissions. Less impactful production processes are grouped in a single agglomerated activity, *Product Manufacturing and Other Industrial Activities*. Production of substances which are conventionally considered to be petrochemical products (i.e. plastics and fertilisers) are not accounted for in this section but in the section on Fuels, Feedstocks and Chemical Products (Section 5.6).

Although most industrial 'activities' (as defined here), such as *Steel* and *Cement*, can be easily represented in mass terms, three activities have been represented by different units:

- *Construction* is represented by the mass of bulk construction materials used: 50% of steel production and 100% cementitious material.
- *Product Manufacturing and Other Industrial Activities* are represented by a proxy metric. The metric for production quantity is not obvious since the type, material, size, weight etc of products is very varied and all of these factors may have an influence on the energy demand. As an indicator value only, remaining industry is accounted for as the sum of number of road vehicles and number of smartphones produced each year (Table S193).
- *Food Processing* is measured in terms of kcal (consistent with the approach used for edible farm produce (Section 5.5.4)).

Sector	Activity	Model Name	ISIC Code	End-Use
Industry	Aluminium Production	Aluminium	242	✓
Industry	Cement Production	Cement	2394, 2395	✓
Industry	Construction	Construction	41, 42, 43	✓
Industry	Food Processing	Food	10, 11, 12	✓
Industry	Glass Production	Glass	231, 2310	✓
Industry	Paper Production	Paper	17, 18	✓
Industry	Product Manufacturing and other Industrial Processes	OtherIndustry	16, 22, 25, 26, 27, 28, 29, 30, 31, 32, 8422	✓
Industry	Steel Production	Steel	191, 241	✓
Industry	Textiles Production	Textiles	13, 14, 15	✓

Table S192: Model flows categorised by ISIC divisions, groups and classes for this Sector. For activities labelled as 'End-Use', the activity rate is set by the model inputs, rather than the demands of other activities.

5.7.1 Sector-wide Data Sources

Most delivery processes within this sector are derived from data sources specific to the given industry. However some activities have been modelled using a top-down approach from current global production and energy consumption data. Energy consumption data is taken from the IEA Energy Balances (International Energy Agency, 2021a).

5.7.2 Cement Production

This activity describes the production of cement from raw materials, excluding preprocessing of supplementary cementitious materials (SCMs). Cement production can be considered to be composed of 3 stages: raw materials preparation, clinker-making and finish grinding. Raw materials (such as limestone, chalk, and clay) are crushed, and ground; materials are burned at high temperature resulting in a chemical reaction to form clinker; and the clinker is ground with SCMs (such as fly ash, blast furnace slag, and calcined clay) to form cement. Addition of SCMs can reduce the clinker factor and therefore the energy demand and emissions of the process. The current process does not explicitly model the flows and pre-processing of SCMs but instead assumes a constant (optimistic) clinker factor. The process is shown in Figure S84. The chosen approach to the amount of clinker in cement and and use of waste is common for all the processes and is discussed below.

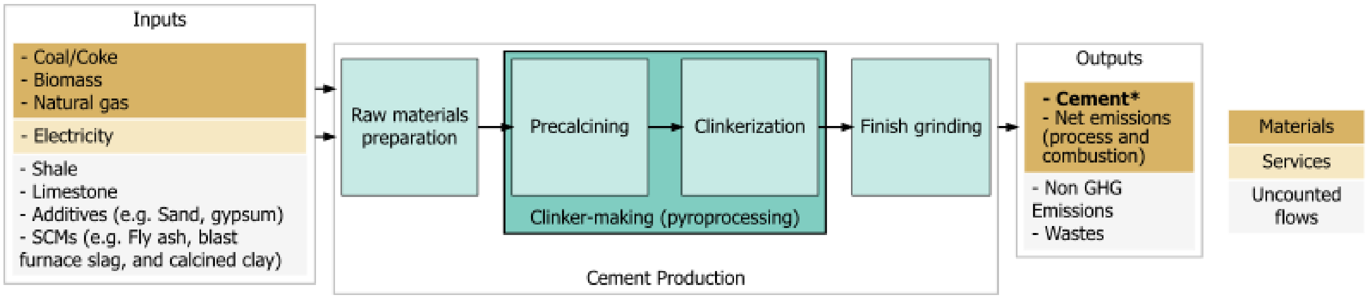

Figure S84: Inputs and outputs for cement production, considering processes in use today. Sub-processes are shown to demonstrate the system boundary but may not have been modelled explicitly for all processes. Pre-processing of supplementary cementitious materials (SCMs) is not accounted for in this process. Where inputs or outputs differ from this diagram, flow diagrams are given in the relevant sections.

Clinker factor The resource demands are highly dependent on the clinker ratio, since production of clinker is the most energy intensive process in cement-making (accounting for 70-80% of energy use according to Worrell et al. (2007)). In this analysis the clinker: cement ratio is assumed to be 0.57 which is the 2050 assumption used by the IEA in their roadmap (down from 0.71 in 2020 and 0.65 in 2030) (IEA, 2021b). This is a very optimistic assumption which is unlikely to be achieved without significant additional energy demands; the only scalable sources of SCMs either demand significant energy for pre-processing (calcined clay), or are by-products from fossil-fuelled processes which would require energy-intensive negative emissions (blast furnace slag from steel production or fly ash from coal-fired power plants). The European Cement Association has a less ambitious target ratio of 0.74 for 2030 (CEMBUREAU, 2020). Although it hasn't been included at this stage, it would be simple to add these flows to the model to investigate the feasibility of assuming such low clinker factors in a net-zero scenario.

Assuming the clinker factor is reduced as much as possible, the main mitigation options for cement production are either CCS or fuel substitution which are described by each of the delivery processes in this section (listed in Table S194). It has been assumed that the process efficiency is not affected by the fuel used. Proposed material substitutions or products which can only be used as a pre-cast building material are not considered here since would require broader system changes. These include use of timber as well as concrete production using CO_2 sequestration in inert carbonate materials (mineralisation) since it cannot be used for ready-mix concrete or for on-site use (it must be pre-cast).

Waste as fuel
Although waste has been increasingly used as an alternative fuel in the cement industry in recent years, particularly in Europe (Beer et al., 2017), the model does not include a delivery process which accounts for this. This is because it would require a more detailed analysis of the composition of waste than the model currently includes, and would potentially require more resource categories to distinguish between different types of waste. Data collected by the Global Cement and Concrete Association (GCCA) suggests that around 20% of cement production globally is fuelled by biomass waste and alternative fuels (mostly non-biomass waste) but this is based on data representing only around 50% of global production, with higher coverage in Europe and North America (GCCA GNR Data for Coverage, and Thermal Energy Consumption, from Global Cement and Concrete Association (n.d.)). Given the data is likely biased towards European plants, where co-processing of waste is more common, this is likely an overestimate of the global average. In the EU around 50% of non-biomass alternative fuels are plastics and tyres and the remainder mostly

other fossil-based sources (International Finance Corporation, 2017). This means combustion of these wastes produce emissions, at a similar level to fossil-fuels. In future, the available supply of these waste streams may also be limited by other environmental measures, such as waste reduction and increased recycling.

Activity	Process	Process Long Name
Cement	Coal	Conventional production fuelled by coal
Cement	Ngas	Conventional production fuelled by natural gas
Cement	Bio	Conventional production fuelled by biomass
Cement	H2	Conventional production fuelled by hydrogen
Cement	CCS	Production with Carbon Capture (oxyfuel)
Cement	BioCCS	Production with carbon capture (oxyfuel), fuelled 100% by woody fuels

Table S194: Delivery processes for this activity. The coefficients for each delivery process are given and explained in the remainder of this section.

5.7.2.1 Conventional cement production (fuelled by coal)

This process describes current best practice cement production assuming that fuel demands are met by coal (Figure S84), and a clinker factor of 0.57 (as discussed on page S214). The coefficients have been derived from best practice energy demand data in Worrell et al. (2007). Emissions are estimated as a sum of process emissions (calculated from stochiometry of limestone calcination: $CaCO_3 \rightarrow CaO + CO_2$) and fuel burn emissions (calculated from the fuel consumed). The calculations are outlined in Table S195. The values chosen are comparable to other best practice values in literature (Table S196).

Label	Property	Value	Unit	Justification
**Clinker Preparation**				
A	Electricity: Raw Materials Prep	0.1	GJ/t clinker	Best practice values - Worrell et al. (2007)
B	Electricity: Solid Fuels Prep	0.01	GJ/t clinker	Best practice values - Worrell et al. (2007)
C	Electricity: Clinker Making	0.08	GJ/t clinker	Best practice values - Worrell et al. (2007)
D	Fuel: Clinker Making	2.85	GJ/t clinker	Best practice values - Worrell et al. (2007)
E	Emissions from Calcination	0.51	t CO2/t clinker	From stochiometry
F	Total Clinker Electricity	0.19	GJ/t clinker	A+B+C
**Cement Making**				
G	Electricity: Additives Prep	0.2	GJ/t additive	Best practice values - Worrell et al. (2007)
H	Fuel: Additives Prep	0.6	GJ/t additive	Best practice values - Worrell et al. (2007)
I	Electricity: Finish Grinding	0.1	GJ/t cement	Best practice values - Worrell et al. (2007)
J	Assumed Clinker: Cement Ratio	0.57		IEA Roadmap to 2050 assumption for 2050
**Total demands: coal-fuelled cement**				
K	Total Electricity	0.29	GJ/t cement	J*F + (1 - J)*G + I
L	Total Fuel	1.88	GJ/t cement	J*D + (1 - J)*H
M	Fuel Emissions Factor	100	kg CO2/GJ	UK Government Conversion Factors (BEIS)
N	Fuel Energy Density	28	GJ/t fuel	UK Government Conversion Factors (BEIS)
O	Total Fuel	0.067	t fuel/t cement	L/N
P	Clinker Fuel Burn Emissions	0.29	t CO2/t clinker	D x M/1000
Q	Additive Prep Emissions	0.060	t CO2/t clinker	H x M/ 1000
R	Total Residual Emissions	0.48	t CO2/t clinker	J*(E + P) + (1 - J)*Q

Table S195: Calculations and assumptions used to derive coefficients for conventional cement production. References: (Worrell et al., 2007), (IEA, 2021b), (Department for Business Energy & Industrial Strategy, 2018). *Note that the clinker factor used in this analysis (J) is potentially challenging to achieve, as discussed on page S214.*

Property	Model values	Model values (adjusted for 0.8 clinker factor)	Voldsund et al. (2019) Reference case	Moya, Pardo and Mercier (2010)	Hills, Florin and Fennell (2016)	MPA (2019) Table 6.1, BAT
Fuel, GJ/ t cement	**1.9**	**2.4**	2.6	2.8		
Electricity, kWh/t	**81**	**81**	105			97
Emissions, kg CO2/t	**482**	**652**	680	672	836	720
Clinker factor	**0.57**	**0.8**		0.8	1.0	0.8

Where values are missing, they were not given in the source. The chosen model values are provided here alongside what they would be if the clinker factor was chosen to be 0.8 as used in the other studies.

Table S196: Comparison of a selection of data for cement production from the literature against the values used in this analysis. This study has assumed a clinker factor of 0.57 (as discussed on page S214). References: (Hills et al., 2016; Mineral Products Association, 2019; J. Moya et al., 2010; Voldsund et al., 2019).

Activity	Process	Resource	Inputs	Outputs	Unit
Cement	Coal	Coal	**-0.067**		Gt/Gt
Cement	Coal	Electricity	**-0.29**		EJ/Gt
Cement	Coal	NetEmissions		**0.48**	Gt_CO2e/Gt
Cement	Coal	Cement		**1.0**	Gt/Gt

Table S197: Chosen coefficients for cement production fuelled by coal.

5.7.2.2 Natural-gas fuelled cement production

This process describes current best practice cement production using natural gas as the primary fuel (Figure S84), and assuming a clinker factor of 0.57 (as discussed on page S214). The coefficients are given in Table S198. These have been derived following the same approach as described in Table S195 but using emissions factors and energy densities for natural gas from the SI Part 2.

Activity	Process	Resource	Inputs	Outputs	Unit
Cement	NGas	Methane	**-0.042**		Gt/Gt
Cement	NGas	Electricity	**-0.29**		EJ/Gt
Cement	NGas	NetEmissions		**0.41**	Gt_CO2e/Gt
Cement	NGas	Cement		**1.0**	Gt/Gt

Table S198: Chosen coefficients for cement production fuelled by natural gas.

5.7.2.3 Biomass fuelled cement production

This process describes current best practice cement production using biomass as the primary fuel (Figure S84), and assuming a clinker factor of 0.57 (as discussed on page S214). The coefficients are given in Table S199. These have been derived following the same approach as described in Table S195 but using emissions factors and energy densities for natural gas from the SI Part 2.

Activity	Process	Resource	Inputs	Outputs	Unit
Cement	Bio	NonWoodBiomass	-0.13		Gt_DryBio/Gt
Cement	Bio	Electricity	-0.29		EJ/Gt
Cement	Bio	NetEmissions		0.29	Gt_CO2e/Gt
Cement	Bio	Cement		1.0	Gt/Gt

Table S199: Chosen coefficients for cement production fuelled by biomass.

5.7.2.4 Hydrogen fuelled cement production

This process describes current best practice cement production using hydrogen as the primary fuel, and a clinker factor of 0.57 (as discussed on page S214). The coefficients are given in Table S200. These have been derived following the same approach as described in Table S195 but using emissions factors and energy densities for natural gas from the SI Part 2.

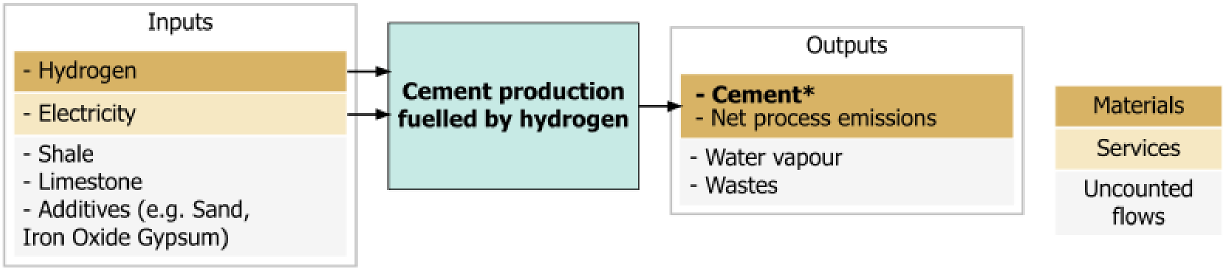

Figure S85: Inputs and outputs for cement production fuelled by hydrogen.

Activity	Process	Resource	Inputs	Outputs	Unit
Cement	H2	Hydrogen	-0.016		Gt/Gt
Cement	H2	Electricity	-0.29		EJ/Gt
Cement	H2	NetEmissions		0.29	Gt_CO2e/Gt
Cement	H2	Cement		1.0	Gt/Gt

Table S200: Chosen coefficients for cement production fuelled by hydrogen.

5.7.2.5 Cement production with carbon-dioxide capture

This process describes cement production with carbon-dioxide capture, assuming oxyfuel combustion in the precalciner, and a clinker factor of 0.57 (as discussed on page S214). Oxyfuel type combined CCS is the preferred technology in the literature (Pamenter & Myers, 2021). This is supported by industry stakeholders and experts interviewed for a study by the Mineral Products Association (Material Economics, 2019), and the Global Cement and Concrete Association (GCCA), who justify it as one of the most promising technologies because of its relatively lower costs (GCCA, 2024).

The coefficients have been based on estimations made by Barker et al. (2009), derived from performance equations of typical industry data and thermodynamic properties. The process here assumes a very high level of technology improvement on those values by increasing the capture rate to 70%. Although there are theoretical estimates of significantly higher capture rates for cement production (Table S201), we found no evidence that these have been demonstrated in practical testing or pilot plants. For example, the "world's first CO_2-capture facility in the cement industry", Heidelberg Materials' Brevik CCS Facility, will achieve only a 50% reduction in plant emissions (Heidelberg Materials Sement Norge, 2024).

Barker et al. (2009) raise a number of technical issues with oxyfuel CCS in the cement industry which helps to explain this discrepancy between theoretical academic papers and real-world performance. Issues include the likelihood of increased wear and tear on equipment due to the higher temperatures involved, likelihood of significant inflows of air into the kiln which would contaminate the CO_2 rich flue gas (part of normal operation of a conventional plant), the need to maintain reducing conditions in the clinker production process, and a critical challenge of process chemistry uncertainties, including whether clinker formation in a changed atmosphere would produce a useful product. On this basis, Barker et al. (2009) consider oxy-combustion in the precalciner only (with air combustion in the kiln). Their modelling of an oxy-combustion process finds 60% of the emissions from a base case plant could be avoided (Table S202). Electricity consumption compared to their base case plant is approximately doubled, but demands for coal and petroleum coke increase by less than 5%. To derive coefficients for CCS production, we adjust the conventional coal cement production process (Sections 5.7.2.1) by the proportional changes in energy demands observed by Barker et al. (2009) for the oxy-combustion case against the reference case. We assume that some improvement may be made against the capture rate modelled by Barker et al. (2009), generously increasing the capture rate to 70%. The coefficients are given in Table S203.

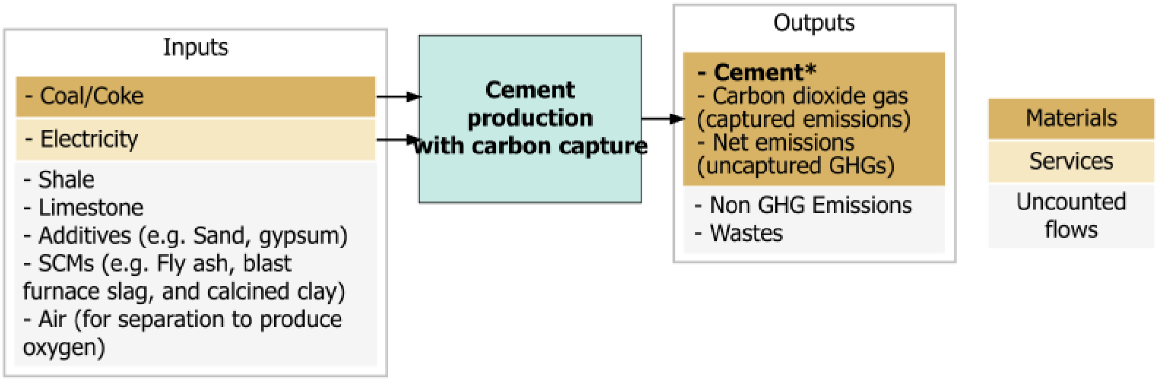

Figure S86: Inputs and outputs for Cement production with carbon-dioxide capture

Parameter	Ref. Plant	MEA	Oxy-Fuel	CAP	MAL	CaL-Tail End	CaL-Integrated
**Energy demands, EJ/Gt cement**							
**Coal**	**1.8**	**1.8**	**1.8**	**1.8**	**1.8**	**4.0**	**3.1**
**Natural gas**		**1.8**		**1.1**			
Power consumption	0.27	0.50	0.62	0.41	0.85	0.82	0.73
Power generation			-0.05			-0.70	-0.37
**Net electricity use**	**0.27**	**0.50**	**0.58**	**0.41**	**0.85**	**0.12**	**0.36**
**Emissions, Gt CO2e/Gt cement**							
Generated	0.48	1.5	0.50	1.1	0.48	0.74	0.65
**Captured**		**1.3**	**0.45**	**0.96**	**0.43**	**0.70**	**0.62**
**Residual**	**0.48**	**0.15**	**0.05**	**0.11**	**0.05**	**0.04**	**0.03**

Table S201: Data from computational modelling of various CCS technologies for cement production, adapted from results of Voldsund et al. (2019) for a reference plant and the following CCS technologies: MEA Adsorption, Oxy-Fuel, Chilled Ammonia Process (CAP), Membrane-Assisted CO_2 Liquefaction (MAL), and Calcium Looping- Tail-End and Integrated.

Parameter	Unit	Base case	Oxy-combustion	Relative change from the base case
Coal feed	kt/y	63	72	**14%**
Pet coke feed	kt/y	33	27	**-18%**
**Total coal and coke feed**	**kt/y**	**96**	**99**	**3%**
**Average net power consumption**	**MW**	**10**	**23**	**123%**
CO2 captured	kt/y	N/A	465	
CO2 emitted on-site	kt/y	728	283	**-61%**
**Total CO2 produced**	**kt/y**	**728**	**748**	**3%**
**Capture rate**		**N/A**	**62%**	

Table S202: Relative changes in emissions and energy demands for oxyfuel CCS cement production compared to conventional production, using data estimated by Barker et al. (2009). Barker et al. (2009) estimate mass and energy flows into subprocesses, calculated from performance equations of typical industry data and thermodynamic properties to find total demands for a base case cement plant (1 Mt/y capacity plant, based in the UK: 5-stage preheater with precalciner dry process) and a new-build plant with Oxy-Combustion CO_2 Capture.

Activity	Process	Resource	Inputs	Outputs	Unit
Cement	CCS	Coal	**-0.069**		Gt/Gt
Cement	CCS	Electricity	**-0.65**		EJ/Gt
Cement	CCS	NetEmissions		**0.15**	Gt_CO2e/Gt
Cement	CCS	CO2Product		**0.35**	Gt_CO2/Gt
Cement	CCS	Cement		**1.0**	Gt/Gt

Table S203: Chosen coefficients for cement production with carbon-dioxide capture.

5.7.2.6 Cement production using biomass fuel with carbon-dioxide capture

This process describes cement production with carbon-dioxide capture, assuming oxyfuel combustion in the precalciner, and all fuel provided by biomass. The clinker factor is taken as 0.57 (as discussed on page S214). The coefficients are based on the delivery process modelled for coal-powered cement production with carbon-dioxide capture (Section 5.7.2.5), assuming that coal can be replaced by the energy equivalent value of wood. Cavalett et al. (2022) show that energy demands do not change significantly with varying proportions of biomass as fuel; the difference in energy demand for processes that use biomass and coal in those studies is smaller than the discrepancy in energy demand estimations of Cavalett et al. (2022) and Voldsund et al. (2019). The residual emissions are based on the carbon balance assumption for biomass (see the SI Part 2). This means that emissions captured and stored from biomass combustion should be counted as negative emissions (since sequestration in biomass growth is not explicitly accounted for. For this calculation, the carbon content of biomass is assumed to be 50% (see the SI Part 2). The coefficients are given in Table S204.

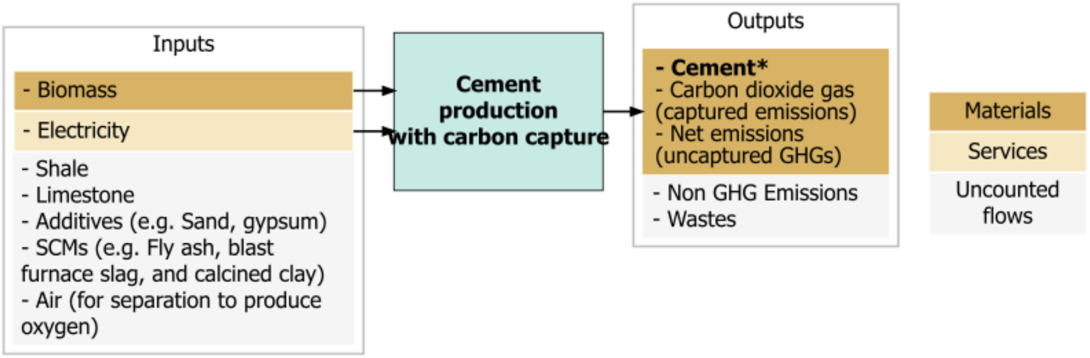

Figure S87: Inputs and outputs for Cement production

Activity	Process	Resource	Inputs	Outputs	Unit
Cement	BioCCS	Wood	-0.13		Gt/Gt
Cement	BioCCS	Electricity	-0.65		EJ/Gt
Cement	BioCCS	NetEmissions	-0.054		Gt_CO2/Gt
Cement	BioCCS	CO2Product		0.37	Gt_CO2/Gt
Cement	BioCCS	Cement		1.0	Gt/Gt

Table S204: Chosen coefficients for cement production with carbon-dioxide capture.

5.7.3 Steel Production

This activity covers the manufacture of iron and steel both from iron ore and from scrap. The process includes material preparation (such as coking, sintering and pelletizing), iron and steel making to produce crude steel, and finishing processes to provide semi-finished products for down-stream manufacturing processes (this is modelled as hot-rolled steel bars or thin slab casting). The process steps included within the modelled delivery process are illustrated in Figure S88 with the simplified process flows shown in Figure S89. Modelled delivery processes are listed in Table S205.

Coefficients for each delivery process have been estimated from a range of sources (listed in Table S206 and summarized Table S207). To maximise consistency, the energy demands for all proceses have been considered against each other, and used to derive coefficients. Emissions values are estimated from the fuel usage, using the emissions factors in the SI Part 2, Table S78.

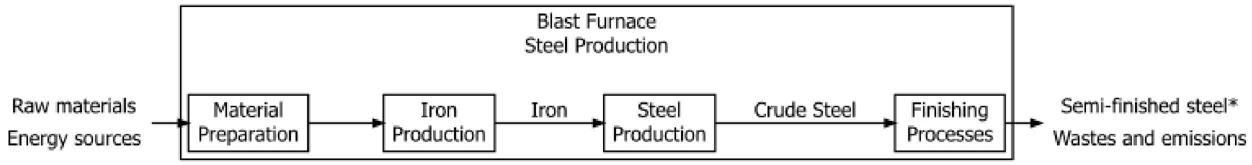

Figure S88: Sub-processes included within Steel Production

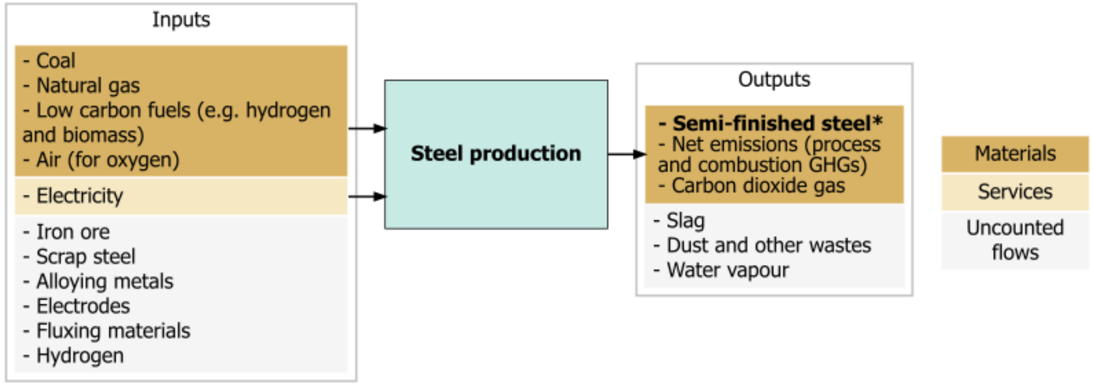

Figure S89: Inputs and outputs for steel production. Hydrogen as an auxiliary reducing agent is not accounted in the model[5].

Activity	Process	Process Long Name
Steel	BOF	Basic Oxygen Furnace primary steel
Steel	EAF	Scrap Steel Recycling via Electric Arc Furnace
Steel	MethaneDRI	Natural gas fuelled steelmaking (MIDREX technology)
Steel	HDRI	Hydrogen DRI (Direct Iron Reduction)
Steel	CCS	Blast Furnace with Top Gas Recycling and Carbon Capture

Table S205: Delivery processes for this activity. The coefficients for each delivery process are given and explained in the remainder of this section.

[5]According to IRENA (2022), hydrogen may be used as an auxiliary reducing agent in the blast furnace process, which could reduce the carbon intensity of the process. Global use of hydrogen in steel production was around 4Mt in 2020 (IRENA, 2022).

Product	Value type	TOTAL Final Energy	TOTAL Fuel	Coal and Coke	Natural Gas	Oil	Hydro-gen	Bioen-ergy	Steam	Electric-ity	Oxygen	Emis-sions (t/t)	Scrap	Source
**EAF from scrap**														
Crude steel	Industry reported (excludes finishing)	**2.1**										0.040	100%	IEA - Iron and Steel Technology Roadmap 2019
Crude steel	World's best practice	**2.4**	**0.60**							1.5	0.30		100%	Worrell et al. (2007)
Hot rolled bar	Assumptions for future German steel industry	**4.2**	**1.3**	0.12	1.2					2.7	0.27	0.088	100%	Arens et al. (2017)
**BF-BOF**														
Crude steel	World's best practice	**14.6**	**13.3**						0.10	0.60	0.60		10-25%	Worrell et al. (2007)
Hot rolled bar (assumed)	Approx 2019 world average	**26.4**	**23.7**	21.0	2.2	0.34		0.20		3.3			0%	Estimated based on production quantities and energy consumption
Crude steel	Industry reported	**21.4**										1.2		IEA - Iron and Steel Technology Roadmap 2020
Crude steel	Industry reported	**19.7**	**18.8**	18.5		0.29				0.85		1.9	0%	SSAB, LKAB and Vattenfall (2017)
Hot rolled bar	Assumptions for future German steel industry	**18.1**	**17.0**	14.8	1.7	0.44				0.41	0.7	1.6	0%	Arens et al. (2017)
**CCS (TGR-BF & BOF)**														
Crude steel	ULCOS TGR-BF Version 4	**11.9**	**10.6**	10.4	0.17					1.3		0.46	11%	Keys, Van Hout and Daniëls (2019), Fig 23. Coke oven gas and heat production are not included.
**Smelt reduction**														
Hot rolled bar	Assumptions for future German steel industry (Hisarna)	**14.5**	**13.6**	11.9	1.4	0.36				0.33	0.57	1.3	0%	Arens et al. (2017)
Crude steel	World's best practice (COREX)	**19.1**	**16.4**						0.20	1.0	1.5		10-25%	Worrell et al. (2007)
**DRI and EAF**														
Hot rolled bar	Assumptions for future German steel industry	**20.7**	**17.5**	0.10	17.4					2.9	0.30	1.1	0%	Arens et al. (2017)
Crude steel	World's best practice	**16.7**	**15.9**						-0.20	0.70	0.30		0%	Worrell et al. (2007)
Crude steel	Industry reported (Hydrogen DRI)	**14.6**	**11.6**	0.15			9.5	2.0		2.9		0.020	0%	SSAB, LKAB and Vattenfall (2017); HYBRIT process
Crude steel	Hydrogen DRI	**9.0**	**6.3**	0.15			6.1	0.0		2.7		0.053	0%	Vogl, Åhman and Nilsson (2018)

Table S207: Summary of the data for steel production considered to estimate the coefficients used in this analysis. All values are given in units of GJ/t Steel, unless stated otherwise. [6]

[6] EAF: Electric Arc Furnace; BF: Blast Furnace; BOF: Basic Oxygen Furnace; CCS: Carbon Capture and Storage; TGR: Top Gas Recycling; DRI: Direct Reduced Iron. HYBRIT, ULCOS, COREX and HISarna are pilot projects or early-stage commercial ventures. ULCOS TGR-BF Version 4 has a capture rate of 60% and accounts for carbon dioxide storage of 0.7 t/t steel.

Reference	Data Source
International Energy Agency (2020)	IEA Iron and Steel Technology Roadmap
Worrell et al. (2007)	Estimated world's best practice guidelines for various industrial processes, compiled by the Lawrence Berkeley National Laboratory
Arens et al. (2017)	Academic paper: Pathways to a low-carbon iron and steel industry in the medium-term in the case of Germany
International Energy Agency (2021a)	World Energy Balances for 2018
World Steel Association (2019)	Steel industry data for 2018
SSAB et al. (2017)	Industry report on the HYBRIT Pre-Feasibility Study
Keys et al. (2019)	Academic paper: The role of hydrogen in the decarbonisation of the iron and steel sector
Vogl et al. (2018)	Academic paper: The role of hydrogen in the decarbonisation of the iron and steel sector

Table S206: Data sources for the values given in Table S207.

The finishing processes for all technologies are assumed to 50% from casting and hot rolling and 50% via thin slab casting (near net-finish processes). Values are estimated as world's best practice values from Worrell et al. (2007), and it is assumed that the fuel is provided by natural gas (Table S208). Thin slab casting would have lower energy demands and combustion emissions but this is compensated by using high efficiency process values. The values for the process with carbon-dioxide capture are estimated by applying the same assumptions as used by Pardo et al. (2012).

The chosen values for all processes are listed in energy terms in Table S209. Conversion to the final coefficients (mostly in mass units) is given in the sub-sections for each delivery process, using the energy density values in the SI Part 2, Table S79.

Finishing Process	Natural Gas	Electricity
Casting and Hot Rolling	1.7	0.3
Thin Slab Casting	0.1	0.2

Table S208: Energy demands (GJ/t) for finishing processes used to estimate process coefficients. (Worrell et al., 2007)

Delivery Process	TOTAL Final Energy	TOTAL Fuel	Coal (& coke)	Natural Gas	Hydrogen	Biomass	Electricity	Emissions (t CO2)	Storage (t CO2)	Scrap
Conventional process (BOF)	**17.8**	**17.0**	15	2.0			0.8	1.62		0%
BOF with CCS	**18.6**	**17.0**	15	2.0			1.6	0.24	1.38	0%
HDRI/EAF	**14.9**	**11.6**	0.2	0.9	8.5	2.0	3.3	0.07		0%
EAF from scrap	**3.7**	**1.4**	0.2	1.2			2.3	0.09		100%

All values are to produce one tonne of semi-finished steel in units of MJ (unless specified).
BOF: Basic Oxygen Furnace; CCS: Carbon Capture and Storage; HDRI: Hydrogen Direct Reduced Iron; EAF: Electric Arc Furnace

Table S209: Chosen values for steel production delivery processes (GJ/t Steel, unless stated otherwise), based on the data in Table S207

5.7.3.1 Conventional primary production (Basic Oxygen Furnace, BOF)

This process is production of semi-finished steel from ore by Blast Furnace (iron production) and Basic Oxygen Furnace (steel production). Finishing processes are assumed to be 50% casting and hot rolling and 50% thin slab casting. The coefficients are estimated based on the data in Table S207 and Table S208, and have been converted into mass units using the energy density values in Table S79. The chosen values are a mid-range estimate between the current energy intensity of production and best practice for future production.

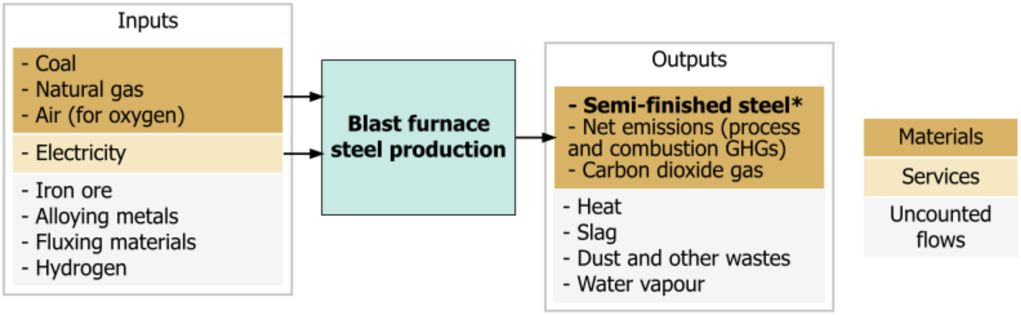

Figure S90: Inputs and outputs for steel production from ore by Basic Oxygen Furnace, BOF. Hydrogen as an auxiliary reducing agent is not accounted in the model.

Activity	Process	Resource	Inputs	Outputs	Unit
Steel	BOF	Coal	-0.54		Gt/Gt_Steel
Steel	BOF	Methane	-0.044		Gt/Gt_Steel
Steel	BOF	Electricity	-0.75		EJ/Gt_Steel
Steel	BOF	NetEmissions		1.6	Gt_CO2e/Gt_Steel
Steel	BOF	Steel		1.0	Gt_Steel/Gt_Steel

Table S210: Chosen coefficients for blast furnace steel production (conventional primary production).

5.7.3.2 Conventional secondary production (Electric Arc Furnace, EAF)

This process is production of semi-finished steel from scrap by Electric Arc Furnace. Finishing processes are assumed to be 50% casting and hot rolling and 50% thin slab casting. The coefficients are estimated based on the data in Table S207 and Table S208, and have been converted into mass units using the energy density values in Table S79. The chosen values are a mid-range estimate between the current energy intensity of production and best practice for future production.

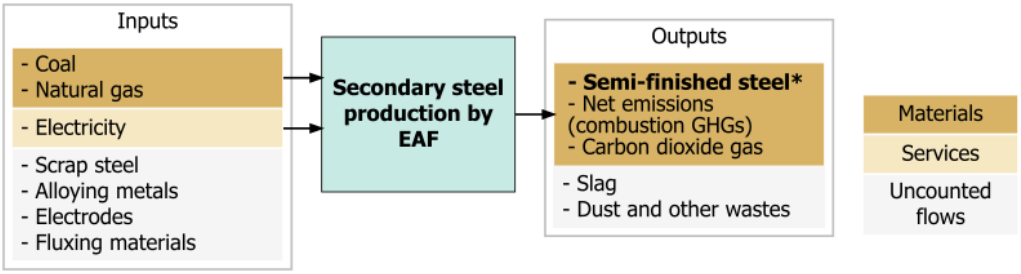

Figure S91: Inputs and outputs for steel production from scrap steel in an Electric Arc Furnace (EAF).

Activity	Process	Resource	Inputs	Outputs	Unit
Steel	EAF	Coal	-0.0071		Gt/Gt_Steel
Steel	EAF	Methane	-0.027		Gt/Gt_Steel
Steel	EAF	NetEmissions		0.092	Gt_CO2e/Gt_Steel
Steel	EAF	Electricity	-2.3		EJ/Gt_Steel
Steel	EAF	Steel		1.0	Gt_Steel/Gt_Steel

Table S211: Chosen coefficients for EAF steel production (conventional secondary production).

5.7.3.3 Methane Direct Reduced Iron, DRI, and Electric Arc Furnace, EAF

This process is production of semi-finished steel from ore by Methane Direct Reduced Iron, DRI (iron making) and Electric Arc Furnace (steel making). The finishing processes are assumed to be 50% casting and hot rolling and 50% thin slab casting. The coefficients are estimated using the data in Table S207 and Table S208, and have been converted into mass units using the energy density values in Table S79.

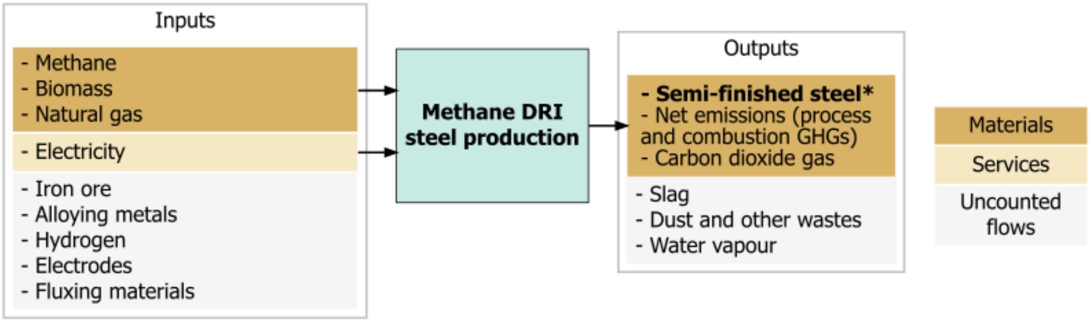

Figure S92: Inputs and outputs for steel production from Direct Reduction of Iron (DRI) using methane and Electric Arc Furnace (EAF).

Activity	Process	Resource	Inputs	Outputs	Unit
Steel	MethaneDRI	Methane	-0.31		Gt/Gt_Steel
Steel	MethaneDRI	NetEmissions		0.85	Gt_CO2e/Gt_Steel
Steel	MethaneDRI	Electricity	-2.2		EJ/Gt_Steel
Steel	MethaneDRI	Steel		1.0	Gt_Steel/Gt_Steel

Table S212: Chosen coefficients for steel production from Direct Reduction of Iron (DRI) using methane and Electric Arc Furnace (EAF).

5.7.3.4 Hydrogen DRI steel production

This process is production of semi-finished steel from ore by Hydrogen Direct Reduced Iron, DRI (iron making) and Electric Arc Furnace (steel making). The coefficients are estimated using the data in Table S207 and Table S208 based on the HYBRIT process (Pei et al., 2020), and have been converted into mass units using the energy density values in Table S79.

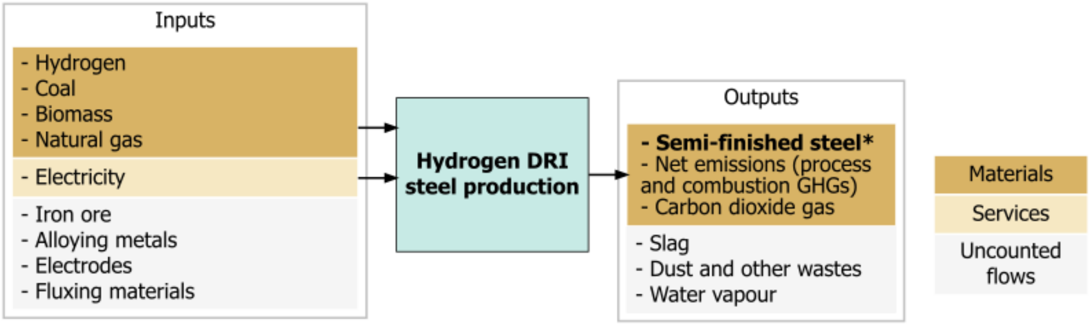

Figure S93: Inputs and outputs for steel production from hydrogen Direct Reduced Iron (DRI)

Activity	Process	Resource	Inputs	Outputs	Unit
Steel	HDRI	Coal	-0.0071		Gt/Gt_Steel
Steel	HDRI	Methane	-0.020		Gt/Gt_Steel
Steel	HDRI	Hydrogen	-0.071		Gt/Gt_Steel
Steel	HDRI	NetEmissions		0.074	Gt_CO2e/Gt_Steel
Steel	HDRI	Wood	-0.13		Gt_DryBio/Gt_Steel
Steel	HDRI	Electricity	-3.3		EJ/Gt_Steel
Steel	HDRI	Steel		1.0	Gt_Steel/Gt_Steel

Table S213: Chosen coefficients for hydrogen DRI steel production.

5.7.3.5 Steel production with carbon-dioxide capture

This process is production of semi-finished steel from ore by Blast Furnace with carbon-dioxide capture. Finishing processes are assumed to be 50% casting and hot rolling and 50% thin slab casting. The coefficients are estimated using the data in Table S207 and Table S208, and have been converted into mass units using the energy density values in Table S79. The values are estimated based on the conventional process by applying the same assumptions as used by Pardo et al. (2012) - capture rate of 85% with an energy penalty of 0.8 GJ/t. Note that this gives a significantly higher overall energy intensity (but lower emissions) than Top-Gas-Recycling (TGR) Blast Furnace with carbon-dioxide capture, being investigated in the ULCOS program (Table S207). The approach used here has been chosen to reflect the wider range of CCS technology options. Many CCS technology options are proposed as new technologies that, if implemented, could increase overall efficiency while reducing emissions (J. A. Moya & Pardo, 2013) but it seems unlikely that these dual benefits could be realised at a commercial level, given the current low level of technical maturity.

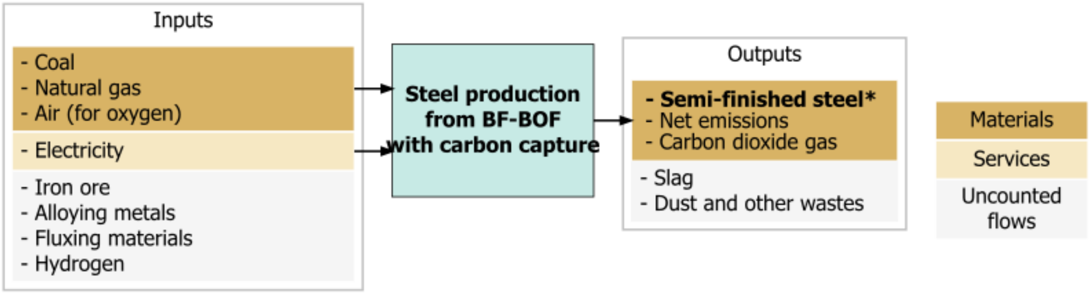

Figure S94: Inputs and outputs for steel production from ore by blast furnace with carbon-dioxide capture. Hydrogen as an auxiliary reducing agent is not accounted in the model [7].

Activity	Process	Resource	Inputs	Outputs	Unit
Steel	CCS	Coal	-0.54		Gt/Gt_Steel
Steel	CCS	Methane	-0.044		Gt/Gt_Steel
Steel	CCS	Electricity	-1.6		EJ/Gt_Steel
Steel	CCS	NetEmissions		0.24	Gt_CO2e/Gt_Steel
Steel	CCS	CO2Product		1.4	Gt_CO2/Gt_Steel
Steel	CCS	Steel		1.0	Gt_Steel/Gt_Steel

Table S214: Chosen coefficients for blast furnace steel production with carbon-dioxide capture.

[7] According to IRENA (2022), hydrogen may be used as an auxiliary reducing agent in the blast furnace process, which could reduce the carbon intensity of the process. Global use of hydrogen in steel production was around 4Mt in 2020 (IRENA, 2022).

5.7.4 Aluminium Production

This activity describes production of aluminium. It includes both primary production and secondary production (from scrap). These have been considered in four delivery processes: conventional primary production, primary production using inert anodes to eliminate process emissions, and secondary production, fuelled either by natural gas or electricity. Bauxite extraction needed for primary production is an energy intensive part of the process but is excluded from these processes; it is (implicitly) accounted for in this model in mining processes but these processes are not connected.

Activity	Process	Process Long Name
Aluminium	NET	Conventional primary production
Aluminium	InertAnodes	Inert Anodes
Aluminium	ScrapElec	Electrified secondary production from scrap
Aluminium	ScrapNG	Secondary production from scrap, fuelled by natural gas

Table S215: Delivery processes for this activity. The coefficients for each delivery process are given and explained in the remainder of this section.

5.7.4.1 Conventional Primary Production (Pre-Baked Anodes)

This process describes conventional primary aluminium production from bauxite (Figure S95). Conventional primary aluminium production may use one of two types of electrolysis technology (smelters): cells with prebaked anodes, or cells with anodes baked-in-situ anodes (known as Søderberg cells). According to the International Aluminium Institute, in 2019 global aluminium facilities were 95% Prebake and 5% Søderberg processes (International Aluminium Institute, 2022); the process for conventional aluminium production is therefore based on the electrolysis with prebaked anodes which is also the most energy-efficient aluminum electrolysis process in conventional use (Worrell et al., 2007).

The process is modelled here as four distinct stages: alumina production from bauxite, anode manufacture, electrolysis to form molten aluminium, and ingot casting & alloying. Fuel, electricity and emissions have been approximated for each of these production stages and then scaled by estimated yield rates to give the overall resource demands. This is summarised in Table S216.

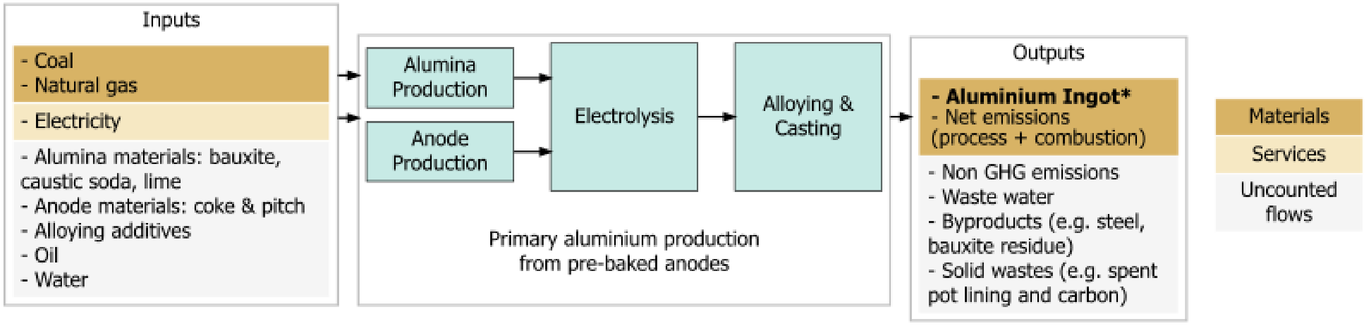

Figure S95: Inputs and outputs for primary aluminium production from prebaked anodes. Sources: International Aluminium Institute (2022); Worrell et al. (2007).

Parameter	Quantity	Unit	Justification
**Assumptions**			
Coal emissions factor	100	kg CO2e/GJ	See Model Assumptions Section
Coal energy density	28	GJ/t coal	See Model Assumptions Section
Methane energy density	45	GJ/t Methane	See Model Assumptions Section
Methane emissions factor	60	kg CO2e/GJ	See Model Assumptions Section
**Alumina production data**			
Electricity	0.73	GJ/t Alumina	Value from Worrell et al. (2007)
Methane	2.9	GJ/t Alumina	Fuel demand from Worrell et al. (2007), distribution from IAI data
Coal	6.8	GJ/t Alumina	Fuel demand from Worrell et al. (2007), distribution from IAI data
Alumina for Aluminium	1.9	t/t Al ingot	Value from Worrell et al. (2007)
**Anode production data**			
Electricity	0.5	GJ/t Anode	Value from Worrell et al. (2007)
Methane	2.5	GJ/t Anode	Value from Worrell et al. (2007)
Anode for Aluminium	0.42	t/t Al ingot	Value from Worrell et al. (2007)
**Electrolysis data**			
Electricity	50	GJ/t Aluminium	Value from Worrell et al. (2007) and IAI (2022)
Process emissions	2.3	t CO2e/t Aluminium	Global reported LCI data from IAI (2022)
Molten Al for ingot	1.0	t/t Al ingot	Value from Worrell et al. (2007)
**Alloying and casting data**			
Electricity	0.8	GJ/t Al ingot	Value between Worrell et al. (2007) and IAI (2022) data
**Aggregated process inputs**			
Electricity	52	GJ/t Al ingot	Summed demand for alumina & anode production, electrolysis, alloying and casting
Coal	13	GJ/t Al ingot	Summed demand for alumina & anode production
Methane	6.6	GJ/t Al ingot	Summed demand for alumina & anode production
Coal demand	0.47	t/t Al ingot	Summed demand for alumina & anode production
Methane demand	0.15	t/t Al ingot	Summed demand for alumina & anode production
**Aggregated process outputs**			
Process emissions	2.3	t CO2e/t Al ingot	From electrolysis
Fuel burn emissions	1.7	t CO2e/t Al ingot	Summed emissions for all fuels calculated as: (total fuel demand) x (emissions factor)
Total emissions	4.0	t CO2e/t Al ingot	Sum of process and combustion emissions

Note:
IAI: International Aluminium Institute, LCI: Life-Cycle Inventory

Table S216: Summary of the assumptions used for conventional primary aluminium production using prebaked anodes. The sources for energy densities and emissions factors are given in the SI Part 2. Other references: International Aluminium Institute (2022); Worrell et al. (2007)

Activity	Process	Resource	Inputs	Outputs	Unit
Aluminium	NET	Coal	-0.47		Gt/Gt
Aluminium	NET	Methane	-0.15		Gt/Gt
Aluminium	NET	Electricity	-52		EJ/Gt
Aluminium	NET	NetEmissions		4.0	Gt_CO2e/Gt
Aluminium	NET	Aluminium		1.0	Gt/Gt

Table S217: Chosen coefficients for conventional primary aluminium production (prebaked anode electrolysis).

5.7.4.2 Aluminium Production using Inert Anodes

This process describes primary aluminium production using inert carbon-free anodes (Figure S96). The technology is in a pilot stage but the aim is that oxygen is produced at the anodes rather than carbon dioxide (Cusano et al., 2017). The process here is modelled as a direct replacement for the conventional process (Section 5.7.4.1), with the same energy demands but no process emissions, based on the assessment by Kvande & Haupin (2001). The authors estimate that the energy consumption of inert anode electrolysis are likely to be similar to aluminium smelting with conventional carbon anodes, while eliminating process emissions. It has also been assumed that the anode is consumed at the same rate as a carbon anode: although the inert anode may have a longer lifetime according to Gautam et al. (2017), it has not been quantified. A longer anode lifetime could (at most) reduce the overall demands by 0.2 GJ electricity and 0.02 Gt Natural Gas - and related combustion emissions of 0.07 Gt CO2 - per tonne Al ingot, given the assumed values used for the conventional process.

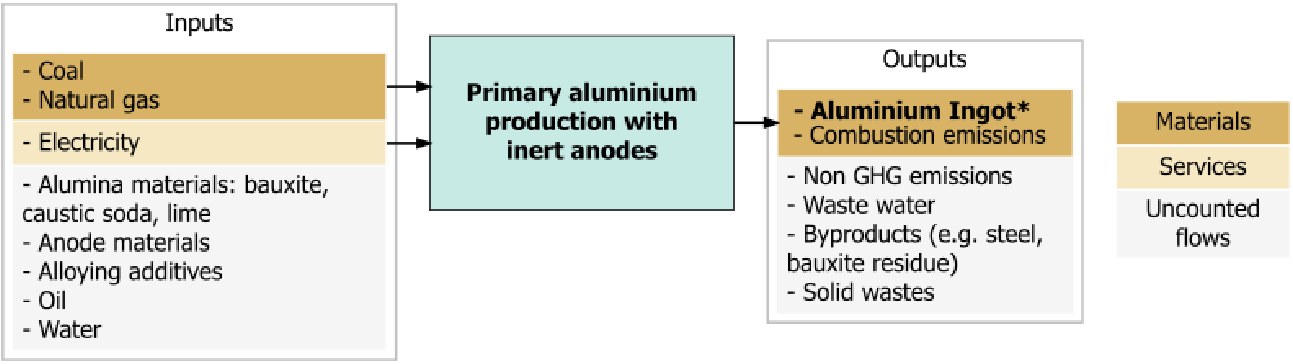

Figure S96: Inputs and outputs for primary aluminium production using inert anodes, based on the conventional process (Section 5.7.4.1 and Kvande & Haupin (2001)

Activity	Process	Resource	Inputs	Outputs	Unit
Aluminium	InertAnodes	Coal	-0.47		Gt/Gt
Aluminium	InertAnodes	Methane	-0.15		Gt/Gt
Aluminium	InertAnodes	Electricity	-52		EJ/Gt
Aluminium	InertAnodes	NetEmissions		1.7	Gt_CO2e/Gt
Aluminium	InertAnodes	Aluminium		1.0	Gt/Gt

Table S218: Chosen coefficients for primary aluminium production using inert anodes.

5.7.4.3 Secondary Aluminium Production - Electrical

This process describes secondary aluminium production using a fully electrified process (Figure S97). Electrical aluminium production from scrap is approximated to demand 3.6 GJ electricity/t Aluminium which is the value quoted by the EU Joint Research Centre report on Best Available Techniques for the Non-Ferrous Metals Industries as an example of an electrically heated gas furnace (Cusano et al., 2017). This is also towards the lower end of the range given by Worrell et al. (2007) for best practice energy consumption for a natural gas fired furnace (3-9 GJ/t). Pretreatment of scrap (including sorting and washing) is not accounted for in this process as it is assumed to have relatively small energy and material requirements compared to the smelting process.

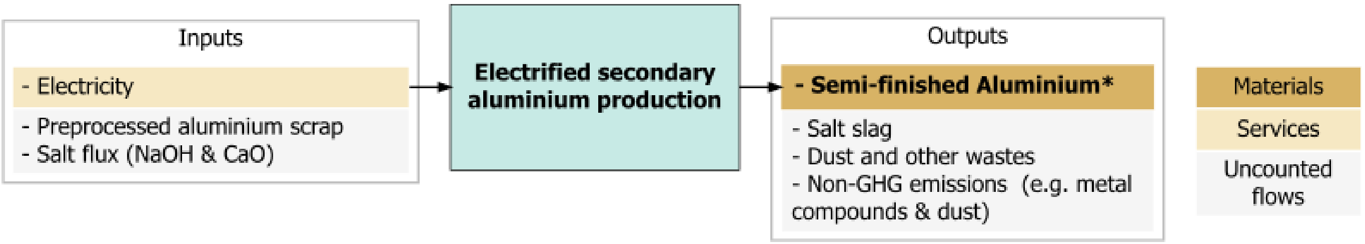

Figure S97: Inputs and outputs for electrified secondary aluminium production. Source: Cusano et al. (2017).

Activity	Process	Resource	Inputs	Outputs	Unit
Aluminium	ScrapElec	Electricity	-3.6		EJ/Gt
Aluminium	ScrapElec	Aluminium		1.0	Gt/Gt

Table S219: Chosen coefficients for electrically powered secondary aluminium production (from scrap).

5.7.4.4 Secondary Aluminium Production - Fossil-Fuel

This process describes secondary aluminium production fuelled by natural gas (Figure S98). According to Worrell et al. (2007), best practice for aluminium production from scrap is using a natural gas fired reverbatory furnace which consume between 3 and 9 GJ fuel/t Al (or as low as 2.5 GJ/t when combined with state-of-the-art computer controls). For consistency with the electrically powered process (Section 5.7.4.3), a value of 3.6 GJ/t is assumed here. This has been converted into mass units and fuel burn emissions are calculated using the factors in Table S78 and Table S79.

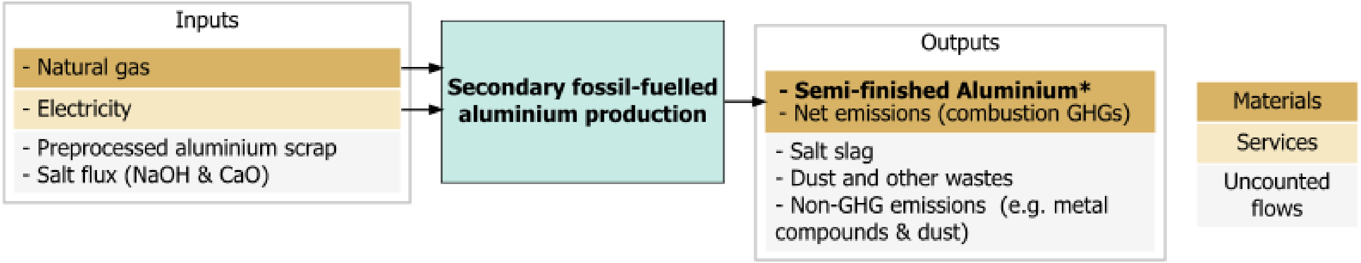

Figure S98: Inputs and outputs for electrified secondary aluminium production. Source: Cusano et al. (2017).

Activity	Process	Resource	Inputs	Outputs	Unit
Aluminium	ScrapNG	Methane	-0.080		Gt/Gt
Aluminium	ScrapNG	NetEmissions		0.22	Gt_CO2e/Gt
Aluminium	ScrapNG	Aluminium		1.0	Gt/Gt

Table S220: Chosen coefficients for secondary aluminium production (from scrap) fuelled by natural gas.

5.7.5 Paper and Pulp Production

This activity covers manufacture of paper and paper products and printing and reproduction of recorded media (ISIC 17 and 18). The activity therefore includes both the production of pulp and then paper/board. Manufacturing processes to produce “finished paper products” (under SIC 17.2) are not included - these would be accounted for in the Activity, *Other Industrial Processes*. Two delivery processes are currently accounted in the model (Table S221).

This is an energy intensive industry but has wide variation in energy demands across the range of products (Suhr et al., 2015). Heat (generally high-pressure steam) is used to generate electrical power with medium- or low-pressure steam from the turbine also used for heating and drying processes, while chemical, mechanical and transport processes are often electrically powered (Suhr et al., 2015).

Given the variation in energy across product types, coefficients are modelled using a top-down approach, based on European production statistics. It is assumed that paper is made exclusively from wood and recycled paper; in reality some non-wood pulp is used in Asia (particularly China and India) e.g. straw. Process emissions occurring from the cyclical reactivation of quicklime in Kraft chemical pulping are not accounted for in this analysis, since these do not appear significant from European statistics (Confederation of European Paper Industries (CEPI), 2021); the total emissions given in the statistics are comparable to those estimated by assuming emissions factors for the fuels used.

Activity	Process	Process Long Name
Paper	NET	Conventional Methods
Paper	Elec	Electrical Processes replace Fossil Fuels

Table S221: Delivery processes for this activity. The coefficients for each delivery process are given and explained in the remainder of this section.

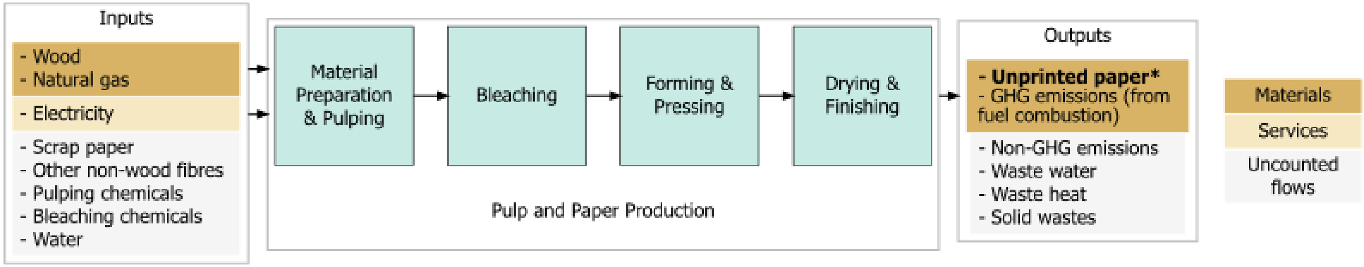

Figure S99: Inputs and outputs for pulp and paper manufactutre. Sources: Suhr et al. (2015); Obrist et al. (2022).

5.7.5.1 Conventional Paper Production

This process describes conventional paper production from wood (Figure S99). The energy intensity of paper production varies significantly depending on the paper-product, specific process, mill size, and material input. For example, Best Available Technology (BAT) energy intensity values for paper-making vary from around 6 GJ/t (making board from recycled paper) to around 15 GJ/t (tissue paper from wood pulp), according to Rogers et al. (2018). The types of pulping process also has a significant impact on the energy demands (examples of best practice are given by Worrell et al. (2007)).

Since the energy intensity of paper production varies significantly depending on the paper-product, specific process, mill size, and material input, the coefficients are estimated “top-down” from European industry statistics. Table S222 lists the assumptions used, and the final values are given in Table S223. It is assumed for this delivery process that all of the energy from fossil fuels is provided by natural gas, and all biomass fuel comes from process residues. In Europe in 2019, bought electricity provided 11% energy use in the paper industry with additional electricity from combined heat and power (Confederation of European Paper Industries (CEPI), 2021). Heat and power were generated from biomass (55% of energy input, mostly from process residues) and fossil fuels (33% energy input, of which 85% natural gas) (Confederation of European Paper Industries (CEPI), 2021). The European paper industry total material input was around 30% recycled paper in 2019, equivalent to around 0.55 t/t paper (Confederation of European Paper Industries (CEPI), 2021).

The overall energy intensity of the generic process used for the model, derived from the European statistics for 2019, is 15 GJ/t paper (including electricity, in addition to fossil and biomass fuels). This is comparable to values identified

as world's best practice values for integrated paper mills by Worrell et al. (2007). Integrated paper mills can be more efficient than stand-alone pulp and paper mills because the pulp does not need to be dried in-between processes, and steam and excess heat can be used more efficiently.

Property	Value	Unit	Notes
**Summary of key data inputs and assumptions**			
Fossil fuel input	6.1	GJ/t paper	CEPI statistics, 2019
Assumed recycled input	30%	As a proportion of total material input	Consistent with CEPI stats
Scrap paper input	0.55	t/t paper	CEPI statistics, 2019
**Calculated coefficients (inputs)**			
Methane energy density	45	GJ/t	Assumptions section
Methane emissions factor	60	kg CO2/GJ	Assumptions section
Electricity demand	1.7	GJ/t paper	CEPI statistics: bought electricity (2019-20)
**Calculated coefficients (outputs)**			
Methane demand	0.14	t/t paper	Assuming natural gas is used as fuel: energy demand / energy density

Table S222: Summary of the assumptions used for conventional paper production. Values are based on European industry statistics for 2019 (Confederation of European Paper Industries (CEPI), 2021). Emissions factors and energy density values are given in the SI Part 2. Biomass fuel is assumed to be entirely met by process residues.

Activity	Process	Resource	Inputs	Outputs	Unit
Paper	NET	Wood	-1.3		Gt_Wood/Gt_Paper
Paper	NET	Electricity	-1.7		EJ/Gt_Paper
Paper	NET	Methane	-0.14		Gt/Gt_Paper
Paper	NET	NetEmissions		0.36	Gt_CO2e/Gt_Paper
Paper	NET	Paper		1.0	Gt_Paper/Gt_Paper

Table S223: Chosen coefficients for conventional paper production.

5.7.5.2 Electrified Paper Production

This process describes electrified paper production, i.e. it assumes that the heat used in conventional production is supplied from electrical power rather than fossil fuels. The process still comprises the steps given in Figure S99, although fuel combustion emissions are eliminated. The efficiency of heat production by electricity is assumed to be the same as for fossil fuels. This is based on the assumptions used by Obrist et al. (2022) that both natural gas and electrical steam boilers have the same efficiency of around 90%.

The coefficients for electrical production are based on the assumptions in Table S222 but where the fuel demand is met by electricity instead of natural gas. Since the only emissions in this activity are from fuel combustion, there are no residual emissions for this delivery process. The resulting coefficients are give in Table S224.

Activity	Process	Resource	Inputs	Outputs	Unit
Paper	Elec	Wood	-1.3		Gt_Wood/Gt_Paper
Paper	Elec	Electricity	-7.8		EJ/Gt_Paper
Paper	Elec	Paper		1.0	Gt_Paper/Gt_Paper

Table S224: Chosen coefficients for electrified paper production.

5.7.6 Glass Production

This activity describes production of glass, and is modelled based on processes for flat and container glass. Coefficients are estimated based on energy intensities derived from literature. Emissions from glass production are a combination of process emissions from the decomposition of carbonates in primary production, and from fuel combustion.

Coefficient values could vary significantly depending on the distribution of glass types (soda-lime, borosilicate, E-glass and lead crystal), the relative shares of glass product types (including container glass, flat glass and glass wool for insulation), and the proportion of cullet (recycled glass). For simplicity the glass industry is assumed to be similar in these respects to the glass industry in Europe in 2007 (the year analysed by Schmitz et al. (2011)). Within Europe, flat and container glass made up 83% of production in 2007 (Schmitz et al., 2011), compared to around 90% globally (according to Westbroek et al. (2021)). The energy demand for container glass production (58% of production in data used by Schmitz et al. (2011)) can be considered "broadly indicative for the industry [except for]... the mineral wool sector" which has additional demands in fiberizing and curing, according to Scalet et al. (2013). Soda-lime is the dominant type of glass produced (making almost all flat and container glass) but other types of glass have lower process emissions (Schmitz et al., 2011).

For reference, a simple schematic of glass production is given in Figure S100. The overall process is illustrated in Figure S101 with the simplified general process shown in Figure S101. Modelled delivery processes are listed in Table S225.

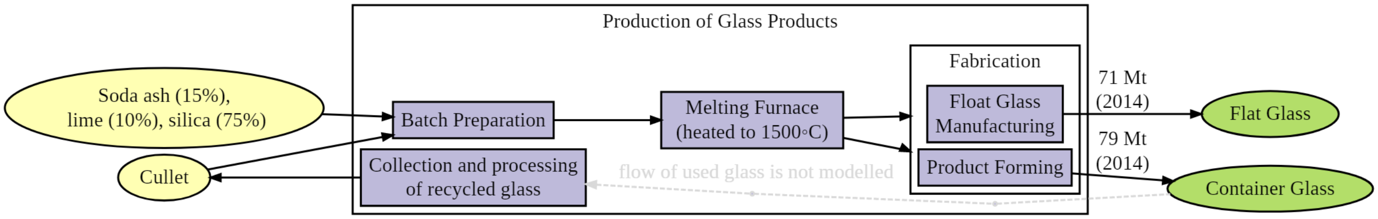

Figure S100: Schematic of the processes considered in glass production. The schematic is based on details given by Westbroek et al. (2021).

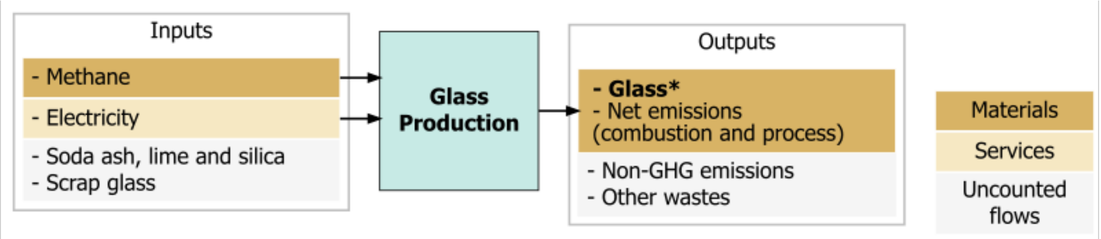

Figure S101: Inputs and outputs for glass production

Activity	Process	Process Long Name
Glass	NET	Conventional processes (45% cullet rate)
Glass	Elec	Electrified process (45% cullet rate)

Table S225: Delivery processes for this activity. The coefficients for each delivery process are given and explained in the remainder of this section.

5.7.6.1 Conventional production

This process describes production of glass using current conventional methods and technologies. The coefficients are based on values estimated by Schmitz et al. (2011) for the European glass industry in 2007, using data from the EU ETS for the period 2005–2007. The estimates from that paper and assumptions used here are given in Table S226. Furnaces can be fired by either heavy fuel oil or natural gas (Scalet et al., 2013) - natural gas is assumed for this analysis since the residual emissions from combustion would be lower. The chosen energy intensity value is slightly below the EU average in 2007. The value is chosen since the EU, as an industrialized region, is likely to have a relatively high level of efficiency on the global scale. The resulting coefficients are given in Table S227.

Property	Value	Unit	Notes
**Data for the EU25 in 2007 from Schmitz et al. (2011)**			
Fuel use	7.8	GJ/t	
Natural gas share of fuel	79%		
Fuel oil share	20%		
Electricity use	1.5	GJ/t	
Process emissions	0.12	t CO2/ t glass product	
**Assumed values for model**			
Cullet use	45%		As indicated for EU in 2007
Fuel demand	7.5	GJ/t	Assumed 100% natural gas
Electricity demand	1.5	GJ/t	
Processs emissions	0.12	t CO2/ t glass product	As indicated for EU in 2007
Combustion emissions	0.45	t CO2/ t glass product	From fuel burn

Table S226: Summary of the assumptions used for conventional glass production. Reference: (Schmitz et al., 2011)

Activity	Process	Resource	Inputs	Outputs	Unit
Glass	NET	Methane	**-0.17**		Gt/Gt_Glass
Glass	NET	Electricity	**-1.5**		EJ/Gt_Glass
Glass	NET	NetEmissions		**0.57**	Gt_CO2e/Gt_Glass
Glass	NET	Glass		**1.0**	Gt_Glass/Gt_Glass

Table S227: Chosen coefficients for conventional production of glass products.

5.7.6.2 Electrified glass production

This process describes fully electrified glass production. Total energy demand is calculated from the average melting energy (3.3 GJ/ t glass) from 2005 survey data from Scalet et al. (2013) (based on three electric furnaces of 47 - 60 tonnes glass/day). This value is scaled to give a total energy demand using the following assumptions:

- The melting furnace is approximately 80% total energy demand for container glass (as given by Scalet et al. (2013)),
- Increasing the cullet share by 5%, decreases the energy consumption by about 1.5% (as mentioned by Schmitz et al. (2011))
- The additional energy for non-container glass can be approximated using a factor of 1.2 to account for the higher overall average production energy intensity compared to container glass (as found by comparing the estimates given by Scalet et al. (2013)).

Activity	Process	Resource	Inputs	Outputs	Unit
Glass	Elec	Electricity	**-5.1**		EJ/Gt_Glass
Glass	Elec	NetEmissions		**0.12**	Gt_CO2e/Gt_Glass
Glass	Elec	Glass		**1.0**	Gt_Glass/Gt_Glass

Table S228: Chosen coefficients for production of glass products using electrified processes.

5.7.7 Construction

This activity describes construction of buildings and infrastructure, based on [ISIC Rev. 4 Divisions 41 to 43], consistent with the IEA world energy balances activity, *Construction* (International Energy Agency, 2021a).

The absence of data, and heterogeneity of this part of industry, means that it is hard to assign resource requirements for specific processes without a detailed analysis of the industry. Instead, the coefficients are derived "top-down" as energy and emissions intensities from IEA energy-use data. The approach used here is to assume that all of these processes could be electrified or fuelled by hydrogen, estimated using relative efficiency values. It is assumed that there are no process emissions.

Since the mass of constructed buildings and infrastructure is not easy to measure, the production metric is taken as the amount of key bulk construction materials produced in 2018: 50% of steel production and 100% cementitious material; i.e. "construction energy/emissions per ton key bulk materials". Production data is shown in Table S229).

In the absence of better data, the demand for wood as a construction material is approximated by assuming that all *sawnwood* produced is used for construction, with the production quantity for 2018 taken from FAO (2022d); all wood based panels and veneer sheets are used for furniture manufacture, accounted for in the delivery processes for *Other Industry*. According to FAO data, 0.23 Gt (FAO, 2022d) of sawnwood was produced in 2018, to produce 4.9 Gt of construction materials' worth of buildings and infrastructure (Table S229). The wood construction materials demand intensity is therefore:

$$0.23/4.9 = 0.047\ t\ sawnwood\ per\ t\ construction\ materials$$

.

Material for metric	2018 Quantity	Unit	Source
Global crude steel production	1.8	Gt_Steel	https://worldsteel.org/wp-content/uploads/Steel-Statistical-Yearbook-2019-concise-version.pdf
Global cement production	4.0	Gt_Cement	https://gccassociation.org/gnr/
TOTAL Key Bulk Construction Materials	4.9	Gt_BulkConstruction **Materials**	**Sum of 50% steel and 100% cement**

Table S229: Data used to define the activity metric.

Activity	Process	Process Long Name
Construction	NET	Conventional construction technologies and techniques
Construction	Elec	Electrified
Construction	H2Power	Fuelled by hydrogen

Table S230: Delivery processes for this activity. The coefficients for each delivery process are given and explained in the remainder of this section.

5.7.7.1 Conventional construction

This process describes conventional construction, using fossil fuels (Figure S102). Coefficients are estimated as the normalised energy demand in 2018 as outlined in Table S231.

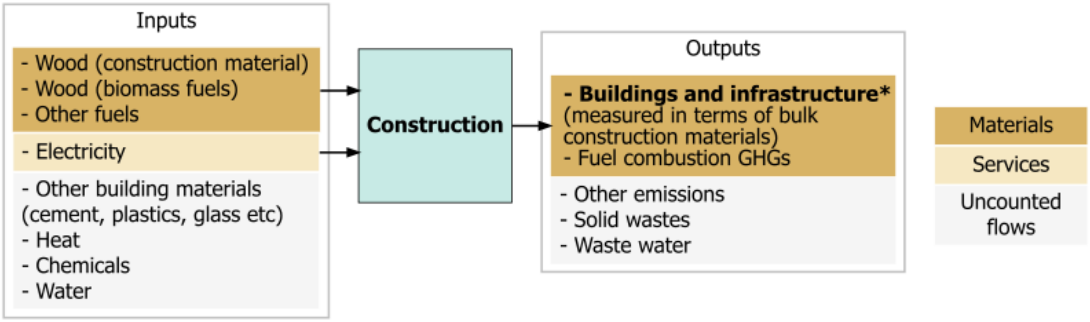

Figure S102: Inputs and outputs for the activity, construction.

Property	Basis for values	Coal/coal products	Oil products	Natural gas	Solid biofuels	Electric-ity	Heat	Total
**Summary of key data inputs and assumptions**								
2018 Energy Used, EJ	IEA World Summary Balances	0.2	1.5	0.4	0.3	0.7	0.1	**3.1**
Energy density, GJ/t	Model Assumptions	28	42	45	15	0	0	
Emissions Factors, Mt/EJ	Model Assumptions	100	70	60	0	0	0	
**Calculated coefficients (inputs)**								
Fuel demand instensity, Gt/Gt	(2018 energy use) / (energy density) / (2018 production)	0.0011	0.0074	0.0017	0.0038	N/A	N/A	**N/A**
Electricity demand intensity, EJ/Gt	(2018 electricity use) / (2018 production)					0.15		
**Calculated coefficients (outputs)**								
Emissions intensity, Gt CO2/Gt	(2018 energy use) x (emissions factor) / (2018 production)	0.003	0.022	0.004	0	0	0	**0.030**

Table S231: Assumptions used to derive coefficients for conventional construction using fossil fuels. IEA energy balances (*Construction*) are used to estimate the fuel demands and combustion emissions, using the model assumptions for energy densities and emissions factors as described in the SI Part 2. The value representing 2018 'production' of construction is given in Table S229. Heat is assumed to be available from other sources as waste heat and so not accounted for here. Primary solid biofuels are assumed to be (or be derived from) wood, and so added to wood for construction materials in the coefficients (page S235).

Activity	Process	Resource	Inputs	Outputs	Unit
Construction	NET	Electricity	-0.15		EJ/Gt_BulkConstruction
Construction	NET	Coal	-0.0011		Gt/Gt_BulkConstruction
Construction	NET	Methane	-0.0017		Gt/Gt_BulkConstruction
Construction	NET	Oil	-0.0074		Gt/Gt_BulkConstruction
Construction	NET	NetEmissions		0.030	Gt_CO2e/Gt_BulkConstruction
Construction	NET	Wood	-0.051		Gt/Gt_BulkConstruction
Construction	NET	Construction		1.0	Gt_BulkConstruction/Gt_BulkConstruction

Table S232: Chosen coefficients for conventional construction.

5.7.7.2 Electrified construction

This process describes construction using electric machinery only (Figure S102). Electrification of construction is estimated from the normalised energy demand in 2018 using the metric in Table S229 and the relative efficiencies in Table S233.

Property	Basis for values	Coal/coal products	Oil products	Natural gas	Solid biofuels	Electricity	Heat	Total
**Summary of key data inputs and assumptions**								
2018 Energy Used, EJ	IEA World Summary Balances	0.2	1.5	0.4	0.3	0.7	0.1	**3.1**
Electrification conversion factor	Assumed	1.0	1.0	1.0	1.0	1.0	0	
**Calculated coefficients (inputs)**								
Equivalent electricity demand, EJ/Gt	(electrification factor) x (2018 energy use) / (2018 production)	0.03	0.31	0.07	0.06	0.15	0	**0.62**

Table S233: Assumptions used to derive coefficients for construction using electrified machinery. Energy densities and emissions factors are given in the SI Part 2. The value representing 2018 ‘production’ of construction is given in Table S229. Heat is assumed to be available from other sources as waste heat and so not accounted for here.

Activity	Process	Resource	Inputs	Outputs	Unit
Construction	Elec	Electricity	**-0.62**		EJ/Gt_BulkConstruction
Construction	Elec	Wood	**-0.048**		Gt/Gt_BulkConstruction
Construction	Elec	Construction		**1.0**	Gt_BulkConstruction/Gt_BulkConstruction

Table S234: Chosen coefficients for electrified construction. Wood demand is for construction materials (page S235).

5.7.7.3 Hydrogen-powered construction

This process describes hydrogen powered construction (Figure S102). Coefficients are estimated from the normalised energy demand in 2018 using the metric in Table S229 and the relative efficiencies in Table S235.

Property	Basis for values	Coal/coal products	Oil products	Natural gas	Solid biofuels	Electricity	Heat	Total
**Summary of key data inputs and assumptions**								
2018 Energy Used, EJ	IEA World Summary Balances	0.2	1.5	0.4	0.3	0.7	0.1	**3.1**
Hydrogen conversion factor	Assumed	1.0	1.0	1.0	1.0	1.0	0	
**Calculated coefficients (inputs)**								
Equivalent hydrogen demand, Gt H2/Gt	(hydrogen factor) x (2018 energy use) / (2018 production) / (hydrogen energy density)	0.0003	0.0026	0.0006	0.0005	0.0012	0	**0.0052**

Table S235: Assumptions used to derive coefficients for construction using hydrogen-powered machinery. Energy densities and emissions factors are given in the SI Part 2. The value representing 2018 ‘production’ of construction is given in Table S229. Heat is assumed to be available from other sources as waste heat and so not accounted for here.

Activity	Process	Resource	Inputs	Outputs	Unit
Construction	H2Power	Hydrogen	**-0.0052**		Gt/Gt_BulkConstruction
Construction	H2Power	Wood	**-0.048**		Gt/Gt_BulkConstruction
Construction	H2Power	Construction		**1.0**	Gt_BulkConstruction/Gt_BulkConstruction

Table S236: Chosen coefficients for electrified construction. Wood demand is for construction materials (page S235).

5.7.8 Food Processing

This activity, *Food Processing*, describes the production of processed food products (as sold in supermarkets for example) from raw food (at the farm gate). It includes "primary processing (such as milling, malting or slaughtering) as well as processing complex prepared foods" consistent with the UK definition of the food manufacturing sector (Tassou et al., 2014).

Emissions in food processing are largely from two sources, according to Garnett (2011): fossil fuel energy use, and refrigerant emissions. The current model accounts for carbon-dioxide, methane and nitrous oxide emissions (the SI Part 1) and so refrigerant emissions are not accounted here. Leakage of hydrocarbon refrigerants can account for about 15% of greenhouse gases emitted in commercial systems (Garnett, 2007).

Since the energy intensity of food processing varies significantly depending on the food product, and region, the coefficients are estimated "top-down" from the global energy use and fuel split in 2018; data is taken from IEA World Summary Energy Balances for the Food and Tobacco industries (International Energy Agency, 2021a). Delivery processes for this activity describe food processing using alternative fuels (Table S237).

The measured output of the activity *Food Processing* is *Processed Food*, measured in the energy units, kcal, rather than in mass units. This approach has been chosen to be consistent with the activity, *Farming Food* (Section 5.5.4)), and makes it more intuitive to consider diet changes as a mitigation option.

Activity	Process	Process Long Name
Food	NET	Conventional Processing Methods
Food	BioF	BioFuels replace Fossil Fuels
Food	Elec	Electrical Processes replace Fossil Fuels

Table S237: Delivery processes for this activity. The coefficients for each delivery process are given and explained in the remainder of this section.

5.7.8.1 Conventional food processing

This process describes the production of processed food products from raw food, using conventional, fossil-fuelled processes (Figure S103). Since the specific processes involved are diverse, and depend on the type of food, the coefficients are estimated from the energy demands in 2018. Emissions are estimated based on the use of fossil fuels, using the emissions factors in the SI Part 2, Table S78. These are outlined in Table S238 giving the coefficients in Table S239.

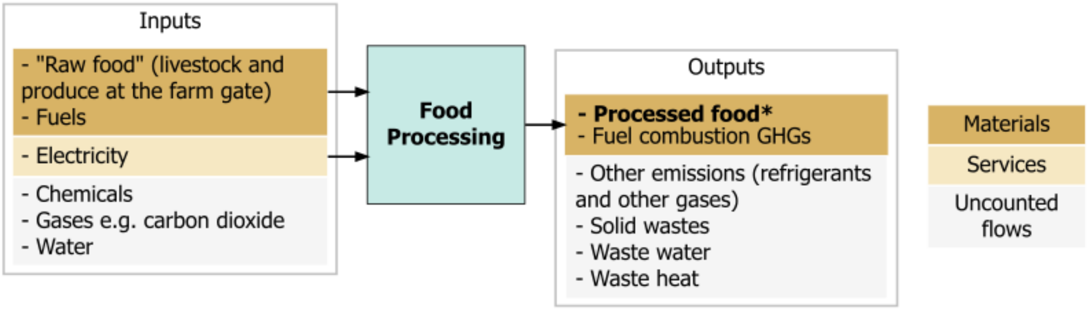

Figure S103: Inputs and outputs for food processing, using conventional fuels and processes.

Property	Coal/ coal products	Oil products	Natural gas	Biofuels and waste	Electricity	Heat	Total	Justification
**Summary of key data inputs and assumptions**								
2018 energy use, EJ	0.9	0.4	2.1	1.8	2.1	0.5	**7.9**	IEA Energy Balances
Emissions factor, kg CO2e/GJ	100	70	60	0	0	0	**N/A**	Document assumptions
Energy density, GJ/t	28	42	45	35	N/A	N/A	**N/A**	Document assumptions
**Calculated coefficients (inputs)**								
Fuel demand intensity (Gt/10^15kcal)	0.0041	0.0011	0.0057	0.0064	N/A	N/A	**N/A**	(energy use) / (energy density) / (2018 food supply)
Electricity demand intensity (EJ/10^15kcal)					0.26	0.00		(energy use) / (2018 food supply); heat is assumed to be available from waste sources
**Calculated coefficients (outputs)**								
Emissions intensity (Gt/10^15kcal)	0.012	0.003	0.015	0	0	0	**0.030**	(energy use) x (emissions factor) / (2018 food supply)

Table S238: Assumptions used to derive coefficients for food processing using conventional methods. IEA energy balances (*Food and tobacco*) are used to estimate the fuel demands and combustion emissions, using the model assumptions for energy densities and emissions factors as described in the SI Part 2. Total food supply in 2018 is taken from FAO (2022c), and equal to 8.2 x10^15kcal. Heat is assumed to be available from other sources as waste heat and so not accounted for here.

Activity	Process	Resource	Inputs	Outputs	Unit
Food	NET	Coal	**-0.0041**		Gt/10^15kcal
Food	NET	Oil	**-0.0011**		Gt/10^15kcal
Food	NET	Methane	**-0.0057**		Gt/10^15kcal
Food	NET	BioFuel	**-0.0064**		Gt/10^15kcal
Food	NET	RawFood	**-1.0**		10^15kcal/10^15kcal
Food	NET	NetEmissions		**0.030**	Gt_CO2e/10^15kcal
Food	NET	Electricity	**-0.26**		EJ/10^15kcal
Food	NET	Food		**1.0**	10^15kcal/10^15kcal

Table S239: Chosen coefficients for conventional food processing.

5.7.8.2 Food processing using biofuel

This process describes the production of processed food products from raw food, using biofuel to replace conventionally fossil-fuelled processes (Figure S104). Without a more detailed understanding of the processes involved, it is assumed that this fuel change does not affect process efficiency. Combustion emissions are eliminated since biogenic carbon is treated as net-neutral (this is explained in part 1 of the SI documents on the analysis framework and assumptions). Emissions related with biofuel production are accounted for in the biomass and biofuel production processes. The coefficients are listed in Table S240.

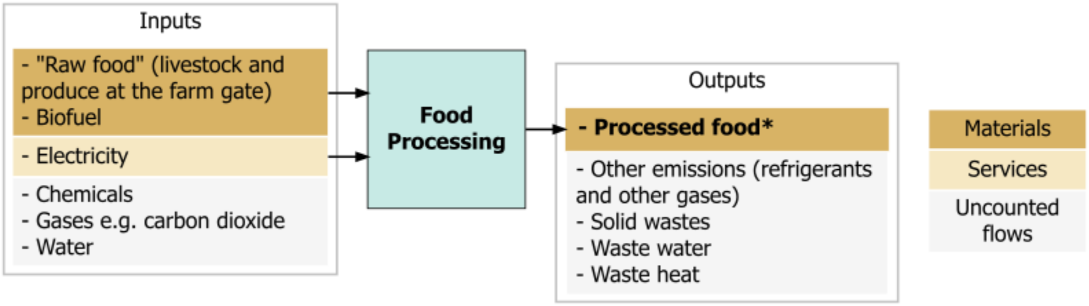

Figure S104: Inputs and outputs for food processing, where fossil fuels are replaced by biofuels or electrification.

Activity	Process	Resource	Inputs	Outputs	Unit
Food	BioF	BioFuel	-0.017		EJ/10^15kcal
Food	BioF	Electricity	-0.26		EJ/10^15kcal
Food	BioF	RawFood	-1.0		10^15kcal/10^15kcal
Food	BioF	Food		1.0	10^15kcal/10^15kcal

Table S240: Chosen coefficients for biofuel powered food processing.

5.7.8.3 Electrified food processing

This process describes the production of processed food products from raw food, where current fossil-fuel powered processes are electrified (Figure S104). The coefficients are based on the values used for the conventional process (Section 5.7.8.1), scaled to account for the variation in efficiency of electrified food-processing activities.

Atuonwu & Tassou (2021) use three categories to classify electrification of heat in food-processing: electro-heating technologies, electrified non-thermal processing technologies, and electricity-driven waste heat upgrade technologies.

The authors tabulate potential energy and time-savings for specific processes within these categories from the literature. Estimated energy savings against conventional approaches range between 20 and 90%, depending on the process and chosen technology. There are also additional potential energy savings which can be more difficult to quantify, such as reduced need for space cooling in facilities if heat can be better direct to the process. The use of heat-pumps in some applications could also reduce the overall energy demand according to Zühlsdorf et al. (2019).

In the absence of a more specific analysis, an average energy saving of 40% compared to fossil-fuels is assumed here. With electricity replacing fossil fuels, combustion emissions are eliminated, and biofuel use is based on 2018 values (Table S238). The coefficients are listed in Table S241.

Activity	Process	Resource	Inputs	Outputs	Unit
Food	Elec	BioFuel	-0.0064		EJ/10^15kcal
Food	Elec	Electricity	-0.51		EJ/10^15kcal
Food	Elec	RawFood	-1.0		10^15kcal/10^15kcal
Food	Elec	Food		1.0	10^15kcal/10^15kcal

Table S241: Chosen coefficients for electrified food processing.

5.7.9 Textiles Production

This activity describes production of textiles from fibres, based on [ISIC Rev. 4 Divisions 13 to 15] as used in the IEA's world energy balances (International Energy Agency - IEA, 2020).

The heterogeneity of the sector means that it is hard to assign resource requirements for specific processes without a detailed analysis of the material flows. Instead, the coefficients are derived as energy and emissions intensities from IEA energy-use data (International Energy Agency, 2021a), with additional biomass to account for natural fibre production. Growing or synthesis of fibres themselves is not considered in this category but in agriculture and chemicals (respectively).

The production quantity is used to normalise the energy use and emissions from the textile industry and is based on data estimated in the 2019 *Fiber & Material Market Report* by Textile Exchange (2019) and analysis of the materials flow of clothing by Ellen MacArthur Foundation (2017). According to the Textile Exchange (2019), global production of fibres was 107 Gt in 2018. The production of textiles is estimated by assuming a 90% yield rate for textile production, consistent with the materials flow of clothing mapped by the Ellen MacArthur Foundation (2017). The 2018 production rate is therefore estimated to be 96 Gt of textiles (Table S242).

Leather processing is not accounted for since it is assumed that the resource requirements for animal rearing (accounted under agriculture) would dominate overall leather production. Global leather production was around 7 Gt globally in 2015 (Textile Exchange, 2019).

Approximate proportions and quantities of global production of fibres (excluding wool, down and silk ~1.5%) are also given by Textile Exchange (2019). These are used to estimate the demand for oil feedstock and biomass (such as cotton or other plant fibres) used to create the material. The estimated biomass demands are given in Table S242 based on the proportions given in the report:

- Synthetic oil-based fibres (polyester, polyamide & other): 51.5+5+5.7 = 63 %
- Cotton: 25%
- Manmade cellulosics fibers (MMCFs) (made from wood - mostly viscose): 6%
- Other plant based (flax & jute etc): 6%

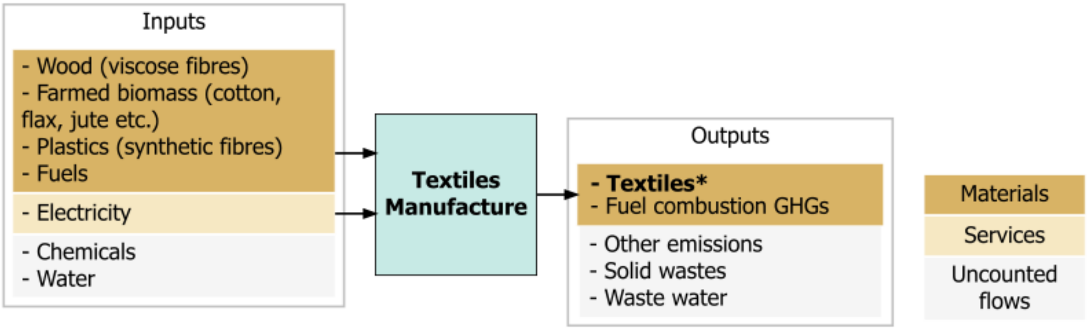

Figure S105: Inputs and outputs for textiles manufacturing.

Activity	Process	Process Long Name
Textiles	NET	Conventional Methods
Textiles	Elec	Electrical Processes replace Fossil Fuels

Table S243: Delivery processes for this activity. The coefficients for each delivery process are given and explained in the remainder of this section.

Property	Quantity	Unit	Justification
**Summary of key data inputs and assumptions**			
Global fibre production	107	Mt fibre	Textile Exchange, Market Report 2019
Textile yield rate	90%	of all fibres	Assuming that clothing is similar to other textile production from (Ellen MacArthur Foundation, 2017)
2018 production of textiles	0.10	Gt Textiles	Yield rate x Fibre production
Natural fibres (inc. cotton, flax & jute)	31%	of all fibres	Textile Exchange, Market Report 2019
Wood based fibres (inc. viscose)	6%	of all fibres	Textile Exchange, Market Report 2019
Plastics based fibre proportion	63%	of all fibres	
**Calculated coefficients (inputs)**			
Farmed biomass demand	0.07	Gt DryBio/ Gt Textiles	(Natural fibre %) x (Fibre production) / (Textile production)
Woody biomass demand	0.34	Gt DryBio/ Gt Textiles	(Wood fibre %) x (Fibre production) / (Textile production)
Plastics demand	0.70	Gt/ Gt Textile	(Synthetic fibre %) x (Fibre production) / (Textile production)

Table S242: Data and calculations for biomass demands in textile production. References: Ellen MacArthur Foundation (2017); Textile Exchange (2019)

5.7.9.1 Conventional Production

This delivery process descibes conventional production methods for textiles, using fossil fuels. The coefficients (Table S244) have been estimated as follows:

- Biomass demand is given as the proportion of fibres produced in 2017 which were plant-based; according to Textile Exchange (2019) this was around 40%, of which around 15% from wood (see Table S242).
- All energy is assumed to be provided by natural gas and electricity, where each is approximated from the global energy use in 2018 from (International Energy Agency, 2021a).
- Emissions are approximated from the consumption of energy in 2018, and the emissions factor for methane (see the SI Part 2).

Activity	Process	Resource	Inputs	Outputs	Unit
Textiles	NET	Methane	**-0.30**		Gt/Gt_Textiles
Textiles	NET	NonWoodBiomass	**-0.34**		Gt_DryBio/Gt_Textiles
Textiles	NET	Wood	**-0.067**		Gt_DryBio/Gt_Textiles
Textiles	NET	NetEmissions		**0.80**	Gt_CO2/Gt_Textiles
Textiles	NET	Electricity	**-13**		EJ/Gt_Textiles
Textiles	NET	Plastics	**-0.70**		Gt/Gt_Textiles
Textiles	NET	Textiles		**1.0**	Gt_Textiles/Gt_Textiles

Table S244: Chosen coefficients for conventional textiles production.

5.7.9.2 Electrification

This delivery process describes electrified production of textiles. The coefficients (Table S245) have been estimated as follows:

- Biomass demand is given as the proportion of fibres produced in 2017 which were plant-based; according to Textile Exchange (2019) this was 41%, of which 6% from wood (see Table S242).
- All energy is assumed to be provided by electricity, based on the global energy use in 2018 from (International Energy Agency, 2021a).
- Final energy demand for electrified processes is assumed to be 80% of final energy demand from conventionally fuelled processes. This is justified as heat can be more precisely directed to specific processes, and through use of heat-pumps. An efficiency saving of 20% is consistent with the value used for electrification of the food processing industry (Section 5.7.8. Given the diversity of processes, it is difficult to estimate the relative energy costs or benefits associated with this fuel shift in greater detail for this level of analysis.

Activity	Process	Resource	Inputs	Outputs	Unit
Textiles	Elec	NonWoodBiomass	**-0.34**		Gt_DryBio/Gt_Textiles
Textiles	Elec	Wood	**-0.067**		Gt_DryBio/Gt_Textiles
Textiles	Elec	Electricity	**-23**		EJ/Gt_Textiles
Textiles	Elec	Plastics	**-0.70**		Gt/Gt_Textiles
Textiles	Elec	Textiles		**1.0**	Gt_Textiles/Gt_Textiles

Table S245: Chosen coefficients for electrified textiles production.

5.7.10 Other Industrial Processes and Manufacturing

This is the category to include manufacturing of products and any materials which have not been considered in other sectors. This grouping is based on the *Machinery*, *Transport equipment*, *Wood and wood products* and *Industry*, *Transport* and *Final Consumption not elsewhere specified* within the IEA world energy balances [ISIC Rev. 4 Divisions 16, 22 & 25-32] (International Energy Agency, 2021a).

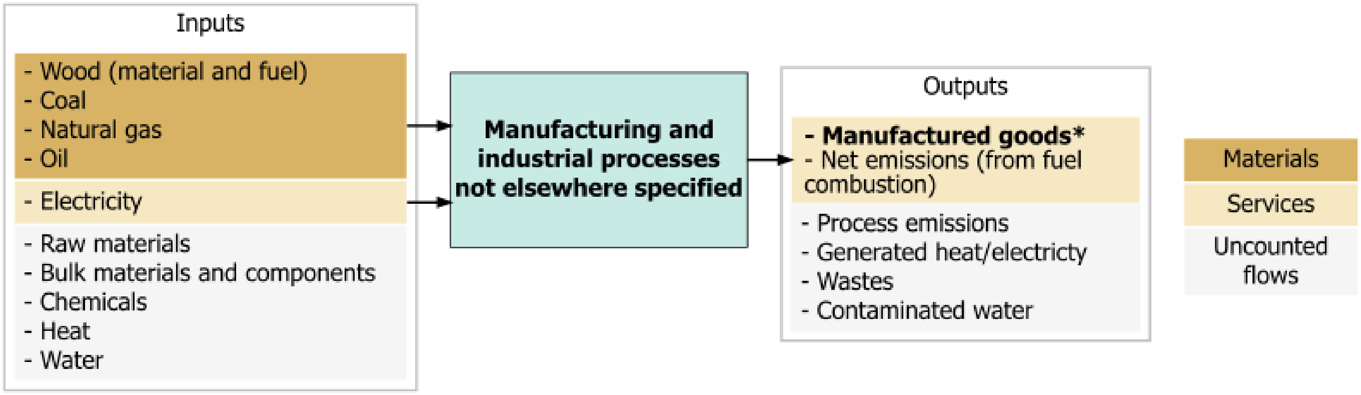

Figure S106: Inputs and outputs for industrial processes not elsewhere specified

The absence of data, and heterogeneity of this part of industry, means that it is hard to assign resource requirements for specific processes without a detailed analysis of the material flows. Instead, the coefficients are derived as energy and emissions intensities from IEA energy-use data. Process emissions are not accounted for. Key data and assumptions used are given in Table S246.

In the absence of better data, the demand for wood in construction is approximated by assuming that all *wood based panels* and *veneer sheets* produced are used for furniture manufacture and accounted for within this activity, with the production quantity for 2018 taken from FAO (2022d). *Sawnwood* is instead assumed to be used entirely for construction and is accounted for in the *construction* delivery processes.

Biofuel and waste consumption in IEA data for this process is dominated by charcoal. In this model, this consumption is attributed to wood. Heat energy has not been included in the conventional process, and has been assumed to be provided by heat pumps in the electrified process.

The metric for production quantity is not obvious since the type, material, size, weight etc of products is very varied and all of these factors may have an influence on the energy demand. As an indicator value only, remaining industry is accounted for as the sum of number of road vehicles and number of smartphones produced each year (Table S193).

Property	Basis for values	Coal and coal products	Crude, NGL and feed-stocks	Oil products	Natural gas	Biofuels and waste	Electric-ity	Heat	Total
Emissions Factors, Mt/EJ	Model Assumptions	100	100	70	60	0	0	0	
2018 Energy Used, EJ	IEA World Summary Balances	5.1	0.1	5.6	8.9	4.2	15.2	0.9	**40**
Emissions, Mt	Emissions factor x EJ 2018 fuel	507	7	391	532	0	0	0	**1437**
Electrification conversion factor	Assumed	1.0	1.0	1.0	1.0	1.0	1.0	0.3	
Electricity demand, if electrified, EJ	Electrification factor x EJ 2018 fuel	5.1	0.1	5.6	8.9	4.2	15.2	0.3	**39**

Table S246: 2018 data for “other industry”. Chosen emissions factors are approximate values, consistent with the model context (the SI Part 2). (International Energy Agency, 2021a)

Material for metric	2018 Quantity	Unit	Source
Global new smartphone sales	1.5	Billion Units	Mordor Intelligence. (2024). Smartphone Market - Mobile, Cell Phone Industry Analysis & Size. https://www.mordorintelligence.com/industry-reports/smartphones-market
Vehicles produced	0.10	Billion Units	OICA (International Organization of Motor Vehicle Manufacturers). (n.d.). 2018 Production Statistics. Retrieved July 29, 2022, from https://www.oica.net/category/production-statistics/2018-statistics/
**Production Indicator**	**0.25**	**Units of Production (arbitrary metric)**	**Vehicles + Smartphones/10**

Table S193: Data used to define the activity metric. The production quantity of smartphones is divided by 10 to make the scale comparable with vehicle manufacture.

5.7.10.1 Conventional industrial processing

This process describes the current manufacturing approach and technologies. Conventional production is estimated as the normalised energy demand in 2018 (using the metric in (Table S193). Emissions are estimated based on the use of fossil fuels, using the emissions factors in the SI Part 2, Table S78. These are outlined in Table S246 giving the coefficients in Table S247.

Activity	Process	Resource	Inputs	Outputs	Unit
OtherIndustry	NET	Electricity	**-62**		EJ/Units_ManufacturingIndicator
OtherIndustry	NET	Coal	**-0.74**		Gt/Units_ManufacturingIndicator
OtherIndustry	NET	Methane	**-0.80**		Gt/Units_ManufacturingIndicator
OtherIndustry	NET	Oil	**-0.54**		Gt/Units_ManufacturingIndicator
OtherIndustry	NET	NetEmissions		**7.9**	Gt_CO2e/Units_ManufacturingIndicator
OtherIndustry	NET	Wood	**-1.9**		Gt/Units_ManufacturingIndicator
OtherIndustry	NET	OtherIndustry		**1.0**	Units_ManufacturingIndicator/Units_ManufacturingIndicator

Table S247: Chosen coefficients for conventional industrial processes, unaccounted for by other activities.

5.7.10.2 Electrified industrial processing

This process describes electrification of industrial processing, eliminating combustion emissions. Electricity demand is estimated from the normalised energy demand in 2018 (using the metric in (Table S193) and the relative efficiencies in Table S246, giving the coefficients in Table S247.

Activity	Process	Resource	Inputs	Outputs	Unit
OtherIndustry	Elec	Electricity	**-160**		EJ/Units_ManufacturingIndicator
OtherIndustry	Elec	Wood	**-0.74**		Gt/Units_ManufacturingIndicator
OtherIndustry	Elec	OtherIndustry		**1.0**	Units_ManufacturingIndicator/Units_ManufacturingIndicator

Table S248: Chosen coefficients for electrified industrial processes, unaccounted for by other activities.

5.7.11 Industry Coefficient Summary

ResourceFlow	Aluminium_NET	Aluminium_InertAnodes	Aluminium_ScrapNG	Aluminium_ScrapElec	Cement_Coal	Cement_NGas	Cement_Bio	Cement_H2	Cement_CCS	Cement_BioCCS	Construction_H2Power	Construction_Elec	Construction_NET
Coal, Gt	-0.47	-0.47			-0.067				-0.069				-0.0011
Methane, Gt	-0.15	-0.15	-0.080			-0.042							-0.0017
Electricity, EJ	-52	-52		-3.6	-0.29	-0.29	-0.29	-0.29	-0.65	-0.65		-0.62	-0.15
NetEmissions, Gt_CO2e	4.0	1.7	0.22		0.48	0.41	0.29	0.29	0.15				0.030
Aluminium, Gt	1.0	1.0	1.0	1.0									
Cement, Gt					1.0	1.0	1.0	1.0	1.0	1.0			
NonWood-Biomass, Gt_DryBio							-0.13						
Hydrogen, Gt								-0.016			-0.0052		
CO2Product, Gt_CO2									0.35	0.37			
Wood, Gt										-0.13	-0.048	-0.048	-0.051
NetEmissions, Gt_CO2										-0.054			
Construction, Gt_BulkConstruction											1.0	1.0	1.0
Oil, Gt													-0.0074
BioFuel, Gt													
RawFood, 10^15kcal													
Food, 10^15kcal													
BioFuel, EJ													
Glass, Gt_Glass													
OtherIndustry, Units_ManufacturingIndicator													
Wood, Gt_Wood													
Paper, Gt_Paper													
Steel, Gt_Steel													
Wood, Gt_DryBio													
Plastics, Gt													
Textiles, Gt_Textiles													

Table S249: Coefficients for industrial production and activities (part 1 of 2).

ResourceFlow	Construction__NET	Food__NET	Food__BioF	Food__Elec	Glass__NET	Glass__Elec	OtherIndustry__Elec	OtherIndustry__NET	Paper__NET	Paper__Elec	Steel__BOF	Steel__CCS
Coal, Gt	-0.0011	-0.0041						-0.74			-0.54	-0.54
Methane, Gt	-0.0017	-0.0057			-0.17			-0.80	-0.14		-0.044	-0.044
Electricity, EJ	-0.15	-0.26	-0.26	-0.51	-1.5	-5.1	-160	-62	-1.7	-7.8	-0.75	-1.6
NetEmissions, Gt__CO2e	0.030	0.030			0.57	0.12		7.9	0.36		1.6	0.24
Aluminium, Gt												
Cement, Gt												
NonWood-Biomass, Gt__DryBio												
Hydrogen, Gt												
CO2Product, Gt__CO2												1.4
Wood, Gt	-0.051						-0.74	-1.9				
NetEmissions, Gt__CO2												
Construction, Gt__BulkConstruction	1.0											
Oil, Gt	-0.0074	-0.0011						-0.54				
BioFuel, Gt		-0.0064										
RawFood, 10^15kcal		-1.0	-1.0	-1.0								
Food, 10^15kcal		1.0	1.0	1.0								
BioFuel, EJ			-0.017	-0.0064								
Glass, Gt__Glass					1.0	1.0						
OtherIndustry, Units__ManufacturingIndicator							1.0	1.0				
Wood, Gt__Wood									-1.3	-1.3		
Paper, Gt__Paper									1.0	1.0		
Steel, Gt__Steel											1.0	1.0
Wood, Gt__DryBio												
Plastics, Gt												
Textiles, Gt__Textiles												

Table S250: Coefficients for industrial production and activities (part 2 of 2)

5.8 Transport

This section outlines the derivation of coefficients for transporting people and goods. Transportation is grouped into the seven activities shown in Table S251. Activities are mostly separated into freight and passenger transport to reflect their very different requirements. Aviation and shipping are exceptions to this since transport by these means cannot be easily substituted by another mode of transport. Similarly, personal transport by car or bus is difficult to assign to transportation of things or people.

Car transportation is accounted for in terms of Vehicle-km to avoid having to distinguish between car journeys to transport things and people. The same approach is used for aviation. This approach is not helpful for other means of transportation since the movement of the vehicle is not relevant but the movement of people or things. Instead, buses are considered to usefully transport people only (and so are accounted for in terms of passenger-km); similarly passenger-rail are accounted for in passenger-km. Freight-Shipping is assumed to dominate the mode and so is accounted for in terms of tonne-km, alongside the other modes of freight transportation (road and rail).

Sector	Activity Long Name	Produces
Transport	Aviation	Passenger air travel
Transport	Bus Transportation	Passenger bus travel
Transport	Car Transportation	Car travel
Transport	Passenger Rail Transportation	Passenger rail travel
Transport	Rail Freight	Rail freight
Transport	Road Freight	Road freight
Transport	Shipping	Freight shipping

Table S251: Activities within this chapter

5.8.1 Sector-wide data sources

As discussed above, transport activities are accounted for in various units dependent on whether the activity is mostly transportation of people or goods. Data for the 2018 activity demand are mostly not directly available since these metrics are not easily measured, and, where they are approximated, data may be held behind a paywall. Activity data for OECD countries are available for some transport modes in terms of *Passenger load factor (pkm/vkm)*, *Passenger-kilometres energy intensity (MJ/pkm)* and *Total final energy (PJ)* (Buses, Cars/light trucks, Domestic passenger airplanes; Domestic passenger ships; Freight trucks; Motorcycles; Passenger trains) (International Energy Agency, 2021b). Other modes are provided in terms of energy only.

The seat-occupancy/packing-density is significant in terms of the overall energy intensity of service delivery. The coefficients are derived on the assumption that there is no significant change from global averages today. Increasing the passenger load factor would reduce the energy intensity of the service, and can be considered as an efficiency saving, as described in the Maths SI document.

Since the available data is not at a global level, activity levels are instead estimated from a combination of various bottom up studies (such as Graver et al. (2019) for aviation) or from energy consumption data converted into the activity metrics used in this model using the coefficients for conventional transport for each mode.

5.8.2 Transportation by car

This activity describes moving people and goods by car. Each delivery process describes use of a different fuel to power cars (Table S252). The coefficients for each process are derived from the assumed tank-to-wheel (TTW) efficiencies. Emissions from the process are calculated based on the fuel consumed, using an emissions factor for each fuel.

Activity	Delivery process	Detail
CarUse	FFICE	Fossil Fuelled
CarUse	BEV	Battery Electric
CarUse	BioICE	Biofuel Powered Internal Combustion Engine
CarUse	HFCEV	Hydrogen Fuel Cell Powered
CarUse	SynICE	Synfuel Powered Internal Combustion Engine

Table S252: Delivery processes for transportation by car.

5.8.2.1 Fossil-Fuelled (Conventional) This delivery process describes conventional fossil-fuelled transportation by car (Figure S107). The process assumes Internal Combustion Engines (ICE) powered by petrol or diesel. The coefficients are derived from assumed tank-to-wheel (TTW) efficiencies for both petrol and diesel cars, and their assumed share of the global fleet. The TTW values for each type of vehicle are chosen based on the data shown in Table S253. The key assumptions and calculations are shown in Table S254 and the chosen coefficients in Table S255.

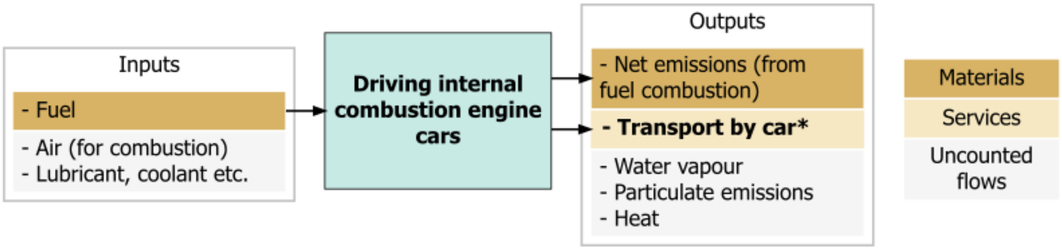

Figure S107: Inputs and outputs for transporting people and things by fossil fuel powered cars.

Data Source	Vehicle Type	TTW efficiency (MJ/km)
**Petrol car data**		
Siegemund et al. (2017)	European Petrol Car - real world consumption (2020)	2.2
Siegemund et al. (2017)	European Petrol Car - real world consumption (2050)	1.8
spritmonitor.de	Most fuel efficient petrol car (on 28th Feb 22),Suzuki Celerio	2.1
**Diesel car data**		
Siegemund et al. (2017)	European Diesel Car - real world consumption (2020)	1.9
Siegemund et al. (2017)	European Diesel Car - real world consumption (2050)	1.5
spritmonitor.de	Most fuel efficient diesel car (on 28th Feb 22), Audi A2 3L	1.5
**All cars**		
DfT, Table ENV0103	Fuel efficiency of the average car sold in the UK in 2019; DfT, Table ENV0103	2.7

Acronyms
DfT: Department For Transport (UK); TTW: Tank to Wheel

Table S253: Data used to estimate the TTW (Tank to Wheel) efficiencies for petrol and diesel cars. References: Siegemund et al. (2017); Fisch und Fischl GmbH (2022); Department for Transport (2020).

Property	Quantity	Unit	Justification
**Data and assumptions - petrol cars**			
TTW efficiency petrol	2.0	MJ/car km	Between the most efficient petrol car available in 2022 and the estimates by Siegemund et al. (2017) for real world 2050 consumption.
Petrol Energy Density	45	GJ/t	UK Government Conversion Factors
Petrol Emissions Factor	75	kg CO2e/GJ	UK Government Conversion Factors
Petrol fuel use	0.045	kg/ car km	TTW efficiency / fuel energy density
Petrol residual emissions	0.15	kg/ car km	Emissions factor x TTW efficiency
Proportion of car fleet petrol	85%		Estimate for global fleet in 2020
**Data and assumptions - diesel cars**			
TTW efficiency diesel	1.7	MJ/car km	As for petrol cars.
Diesel Energy Density	43	GJ/t	UK Government Conversion Factors
Diesel Emissions Factor	71	kg CO2e/GJ	UK Government Conversion Factors
Diesel car fuel use	0.040	kg/ car km	TTW efficiency / fuel energy density
Diesel residual emissions	0.12	Gt CO2e/ 10^12_car_km	Emissions factor x TTW efficiency
Proportion of car fleet diesel	15%		Estimate for global fleet in 2020
**Calculated coefficients - inputs**			
Fuel use	0.044	Gt/ 10^12_car_km	Weighted average of petrol and diesel, based on assumed fleet composition
**Calculated coefficients - outputs**			
Residual Emissions	0.15	Gt CO2e/ 10^12_car_km	Weighted average of petrol and diesel, based on assumed fleet composition

Acronyms
TTW - Tank to Wheel

Table S254: Key assumptions for estimating the coefficients for transportation by conventional fossil-fuelled cars. References: Department for Business Energy & Industrial Strategy (2018)

Activity	Process	Resource	Inputs	Outputs	Unit
CarUse	FFICE	Oil	**-0.044**		Gt/10^12_car_km
CarUse	FFICE	NetEmissions		**0.15**	Gt_CO2e/10^12_car_km
CarUse	FFICE	CarUse		**1.0**	10^12_car_km/10^12_car_km

Table S255: Chosen coefficients for transportation by conventional fossil-fuelled cars.

5.8.2.2 Battery Electric

This delivery process is for Battery Electric Vehicle (BEV) transportation by car (Figure S108). The coefficients are derived from an assumed tank-to-wheel (TTW) efficiency of 0.55 $EJ_{elec}/\ 10^{12}_{car-km}$ based on the data in Table S256.

The derived coefficients are shown in Table S257.

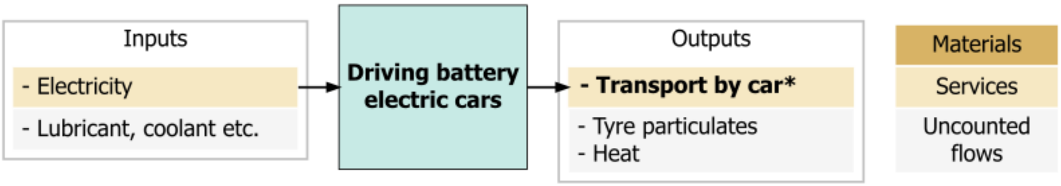

Figure S108: Inputs and outputs for transporting people and things by battery electric cars (BEVs).

Data Source	Vehicle Type	TTW efficiency (MJ/km)
Huss and Weingerl (2020), Tables 5-10	C-segment 5-seater sedan; 2015 value: Simulation of NEDC. European basis. Includes charging losses.	0.46
Siegemund et al. (2017)	2020 BEV. Mid-range value from a wide range of sources, considering real-world use and changing losses. European basis.	0.60
Siegemund et al. (2017)	2050 BEV. Mid-range value from a wide range of sources, considering real-world use and changing losses. European basis.	0.53
Official manufacturer value: Nissan company website	Nissan Leaf ACENTA based on WLTP	0.74

Acronyms
HFCEV: Hydrogen Fuel Cell Electric Vehicle; NEDC: The New European Driving cycle; TTW: Tank to Wheel; WLTP: Worldwide harmonized Light duty Test Procedure

Table S256: Key data used to estimate the TTW efficiency of electric cars. References: Huss & Weingerl (2020); Siegemund et al. (2017); www-europe.nissan-cdn.net/content/dam/Nissan/gb/brochures/Vehicles/Nissan_Leaf_UK.pdf (Accessed: 21 Nov 2021).

Activity	Process	Resource	Inputs	Outputs	Unit
CarUse	BEV	Electricity	-0.55		EJ elec/10^12_car_km
CarUse	BEV	CarUse		1.0	10^12_car_km/10^12_car_km

Table S257: Chosen coefficients for transportation by battery electric car.

5.8.2.3 Biofuel Powered Internal Combustion Engine

This delivery process is for biofuel powered car transportation, assuming an internal combustion engine similar to a conventional fossil fuelled internal combustion engine (Figure S107). The efficiency of a car powered by biofuel is assumed to be similar to a petrol car. The process to produce biofuels is accounted for in Section 5.6.12.

The key assumptions and calculations are shown in Table S258 and the chosen coefficients in Table S259.

Property	Quantity	Unit	Justification
**Data and assumptions**			
Internal Combustion Engine TTW efficiency	2.0	MJ/km = EJ/ 10^12_car_km	Assume same engine efficiency (on energy basis) as for a petrol car
Bio-fuel Energy Density	35	GJ/t	UK Government Conversion Factors give values from 27 (for bio-ethanol) to 44 (for Hydrogenated Veg Oil, HVO bio-diesel) GJ/t
**Calculated coefficients (inputs)**			
Bio-fuel use	0.056	Gt/ 10^12_car_km	TTW efficiency / fuel energy density
**Calculated coefficients (outputs)**			
Residual Emissions	0	Gt CO2e/ 10^12_car_km	Emissions from the carbon in biomass (and production of biomass) are accounted for in the biomass production process. Emissions from biofuel production are accounted for in the biofuel production process.

Acronyms
TTW: Tank to Wheel

Table S258: Key assumptions for estimating the coefficients for biofuel powered car transportation. References: Department for Business Energy & Industrial Strategy (2018)

Activity	Process	Resource	Inputs	Outputs	Unit
CarUse	BioICE	BioFuel	**-0.056**		Gt/10^12_car_km
CarUse	BioICE	CarUse		**1.0**	10^12_car_km/10^12_car_km

Table S259: Chosen coefficients for biofuel powered car transportation.

5.8.2.4 Hydrogen Fuel Cell Powered

This delivery process is for Hydrogen Fuel Cell Electric Vehicle (HFCEV) car transportation (Figure S109). The coefficients are derived from an assumed tank-to-wheel (TTW) efficiency based on the data in Table S260. The key assumptions and calculations are shown in Table S261 and the chosen coefficients in Table S262.

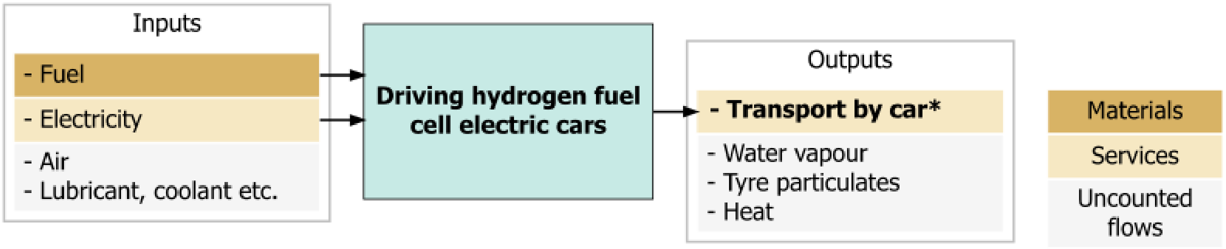

Figure S109: Inputs and outputs for transporting people and things by hydrogen fuel cell electric (HFCEV) cars.

Data Source	Vehicle Type	TTW efficiency (MJ/km)
Huss and Weingerl (2020)	C-segment 5-seater sedan; WLTP Simulation	0.7
Siegemund et al. (2017)	2020 HFCEV. Mid-range value from a wide range of sources, considering real-world use and changing losses. European basis.	1.1
Siegemund et al. (2017)	2050 HFCEV. Mid-range value from a wide range of sources, considering real-world use and changing losses. European basis.	0.8
spritmonitor.de	Average user input values as of Feb 2022	1.1

Acronyms
HFCEV: Hydrogen Fuel Cell Electric Vehicle; NEDC: The New European Driving cycle; TTW: Tank to Wheel; WLTP: Worldwide harmonized Light duty Test Procedure

Table S260: Key data for estimating the coefficients for transportation by hydrogen fuel cell electric car.
References: Huss & Weingerl (2020); Siegemund et al. (2017); Fisch und Fischl GmbH (2022).

Property	Quantity	Unit	Justification
**Assumptions**			
TTW efficiency	0.90	MJ/km = EJ/ 10^12_car_km	Upper central estimate of the european-based values to include real world losses
Hydrogen energy density	120	EJ/Gt Hydogen	LHV - Giddey et al. (2013)
**Calculated coefficients (inputs)**			
Fuel demand	0.0075	Gt/ 10^12_car_km	TTW efficiency / fuel energy density

Acronyms
TTW: Tank to Wheel; HFCEV: Hydrogen Fuel Cell Electric Vehicle; LHV: Lower Heating Value

Table S261: Key assumptions for estimating the coefficients for transportation by hydrogen powered cars. References: Giddey et al. (2013)

Activity	Process	Resource	Inputs	Outputs	Unit
CarUse	HFCEV	Hydrogen	-0.0075		Gt/10^12_car_km
CarUse	HFCEV	CarUse		1.0	10^12_car_km/10^12_car_km

Table S262: Chosen coefficients for transportation by hydrogen fuel cell electric car.

5.8.2.5 Synfuel Powered Internal Combustion Engine

This delivery process is for synthetic-fuel powered car transportation, assuming an internal combustion engine similar to a conventional fossil fuelled internal combustion engine (Figure S107). The efficiency is assumed to be similar to a petrol car. The process to produce synthetic hydrocarbon fuels is accounted for in Section 5.6.11. The assumptions in Table S263 are used to derive the chosen coefficients in Table S264.

Property	Quantity	Unit	Justification
**Data and assumptions**			
Internal Combustion Engine TTW efficiency	2.0	MJ/km = EJ/ 10^12_car_km	Assume same engine efficiency (on energy basis) as for a petrol car
Synthetic fuel Energy Density	43	GJ/t	Van Der Giesen et al. (2014)
CO2 input for Synfuel production	3.2	kg/ kg synfuel	From Synfuel production process by Fischer-Tropsch
**Calculated coefficients (inputs)**			
Synthetic fuel use	0.046	Gt/ 10^12_car_km	TTW efficiency / fuel energy density
**Calculated coefficients (outputs)**			
Residual Emissions	0.15	Gt CO2e/ 10^12_car_km	Carbon dioxide to produce synfuel must be released during combustion. Residual emissions are thus: CO2 content of SynFuel x SynFuel consumption.

Acronyms
TTW: Tank to Wheel

Table S263: Key assumptions for estimating the coefficients for synthetic-fuel powered car transportation. References: Van Der Giesen et al. (2014)

Activity	Process	Resource	Inputs	Outputs	Unit
CarUse	SynICE	Synfuel	**-0.046**		Gt/10^12_car_km
CarUse	SynICE	NetEmissions		**0.15**	Gt_CO2e/10^12_car_km
CarUse	SynICE	CarUse		**1.0**	10^12_car_km/10^12_car_km

Table S264: Chosen coefficients for synthetic-fuel powered car transportation.

5.8.3 Transportation by bus

This activity describes moving people by bus. Coefficients are derived using fuel consumption estimates (TTW, tank-to wheel) for each drive-train technology from a range of sources - these references consider urban bus travel rather than long-distance. The fuel consumption per km can vary significantly with driving conditions and the operational context (Mahmoud et al., 2016; Zemo Partnership, 2022); the technology configuration for these different applications would have different requirements, such as range, leading to further variation. As such, it is assumed that the values here are sufficient to represent all bus travel for a first approximation only.

Any residual emissions are calculated from the fuel consumption using an assumed emissions factor for the fuel. The "per bus" values are divided by an assumed passenger occupancy to determine the "per passenger" metrics. It is assumed that the distance where the bus travels empty (eg to and from depots) is minimal.

The number of passengers is highly uncertain and will depend on the bus-route and network configuration, amongst many other socio-economic factors. For a crude approximation we assume 20 passengers per bus; this would be consistent with Siegemund et al. (2017) who assume 23 passengers per bus, and Correa et al. (2019) who assume the weight of passengers to be 1500 kg.

Activity	Delivery process	Detail
BusUse	FFICE	Conventional fossil-fuelled bus transport
BusUse	BioICE	Biofuel powered bus passenger transport
BusUse	SynICE	Synthetic fuel powered bus passenger transport
BusUse	BEV	Battery electric bus passenger transport
BusUse	HFCEV	Hydrogen fuel cell bus passenger transport

Table S265: Delivery processes for passenger transportation by bus.

	Mean fuel consumption (MJ/km)					
Power train	Correa, Muñoz & Rodriguez (2019)	Mahmoud et al. (2016)	Schmidt et al. (2016) - 2020	Schmidt et al. (2016) - 2050	Zemo Partnership (2022)	Chosen values
BEV	6.5	6.8			2-7	4
Diesel	15.9	13.5	13.6	10.8		12
HFCEV	12.1	10.5	14.0	11.4		11

Acronyms
BEV: Battery Electric Vehicle; HFCEV: Hydrogen Fuel Cell Electric Vehicle

Table S266: Key data sources for estimating the fuel consumption of buses and the chosen values used in this analysis. References: (Correa et al., 2019; Mahmoud et al., 2016; Schmidt et al., 2016; Zemo Partnership, 2022)

5.8.3.1 Fossil-Fuelled (Conventional) Bus

This delivery process is for conventional fossil-fuelled passenger transportation by bus (Figure S110). Average fuel consumption is estimated based on the sources shown in Table S266. This value is combined with energy density and emissions factors to calculate the coefficients, as shown in Table S267. The derived coefficients are shown in Table S268.

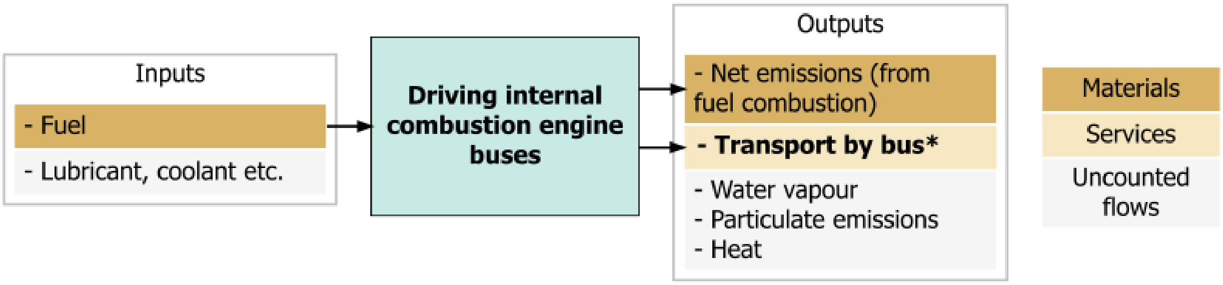

Figure S110: Inputs and outputs for transporting people by bus, powered by internal combustion engines.

Property	Quantity	Unit	Justification
**Data and assumptions**			
Passengers per bus	20	passengers	Similar to the assumptions used by Sigemund et al. (2017) and Correa, Muñoz & Rodriguez (2019)
Fuel consumption	12	MJ/ bus km	Correa, Muñoz & Rodriguez (2019); Mahmoud et al. (2016); Schmidt et al. (2016)
Diesel Energy Density	43	GJ/t	UK Government Conversion Factors
Diesel Emissions Factor	71	Mt CO2e/EJ	UK Government Conversion Factors (BEIS)
**Calculated coefficients (inputs)**			
Diesel Bus fuel use	0.280	Gt/ 10^12_bus_km	Fuel consumption / fuel energy density
**Calculated coefficients (outputs)**			
Diesel residual emissions	0.85	Gt CO2e/ 10^12_bus_km	Emissions factor x Fuel consumption

Table S267: Key assumptions for estimating the coefficients for transportation by conventional fossil-fuelled bus. References: Department for Business Energy & Industrial Strategy (2018)

Activity	Process	Resource	Inputs	Outputs	Unit
BusUse	FFICE	Oil	-0.014		Gt/10^12pkm
BusUse	FFICE	NetEmissions		0.042	Gt_CO2e/10^12pkm
BusUse	FFICE	BusUse		1.0	10^12pkm/10^12pkm

Table S268: Chosen coefficients for conventional fossil-fuelled bus use.

5.8.3.2 Biofuel Powered Internal Combustion Engine Bus

This delivery process is for biofuel powered passenger bus transportation, assuming an internal combustion engine similar to a conventional fossil fuelled internal combustion engine (Figure S110). The efficiency of a bus powered by biofuel is assumed to be similar to a diesel bus. The process to produce biofuels is accounted for in Section 5.6.12. The key assumptions and calculations are shown in Table S269 and the chosen coefficients in Table S270.

Property	Quantity	Unit	Justification
**Data and assumptions**			
Passengers per bus	20	passengers	Similar to the assumptions used by Sigemund et al. (2017) and Correa, Muñoz & Rodriguez (2019)
Fuel consumption	12	MJ/ bus km	Correa, Muñoz & Rodriguez (2019); Mahmoud et al. (2016); Schmidt et al. (2016)
Bio-fuel Energy Density	35	GJ/t	UK Government Conversion Factors (BEIS) give values from 27 (for bio-ethanol) to 44 (for Hydrogenated Veg Oil, HVO bio-diesel) GJ/t
**Calculated coefficients (inputs)**			
Bio-fuel use	0.34	Gt/ 10^12_bus_km	Fuel consumption / fuel energy density
**Calculated coefficients (outputs)**			
Residual Emissions	0	Gt CO2e/ 10^12_bus_km	Emissions from the carbon in biomass (and production of biomass) are accounted for in the biomass production process. Emissions from biofuel production are accounted for in the biofuel production process.

Table S269: Key assumptions to estimate biofuel-powered bus coefficients. References: Department for Business Energy & Industrial Strategy (2018); Correa et al. (2019); Mahmoud et al. (2016); Schmidt et al. (2016)

Activity	Process	Resource	Inputs	Outputs	Unit
BusUse	BioICE	BioFuel	-0.017		Gt/10^12pkm
BusUse	BioICE	BusUse		1.0	10^12pkm/10^12pkm

Table S270: Chosen coefficients for biofuel powered bus transportation.

5.8.3.3 Synfuel Powered Internal Combustion Engine Bus

This delivery process is for synthetic-fuel powered passenger bus transportation, assuming an internal combustion engine similar to a conventional fossil fuelled internal combustion engine (Figure S110). The efficiency of a synthetic fuel bus is assumed to be similar to a diesel bus. The process to produce synthetic hydrocarbon fuels is accounted for in Section 5.6.11. The key assumptions and calculations are shown in Table S271 and the chosen coefficients in Table S272.

Property	Quantity	Unit	Justification
**Data and assumptions**			
Passengers per bus	20	passengers	Similar to the assumptions used by Sigemund et al. (2017) and Correa, Muñoz & Rodriguez (2019)
Fuel consumption	12	MJ/ bus km	Correa, Muñoz & Rodriguez (2019); Mahmoud et al. (2016); Schmidt et al. (2016)
Synthetic fuel Energy Density	43	GJ/t	Van Der Giesen et al. (2014)
CO2 input for Synfuel production	3.2	kg/ kg synfuel	From Synfuel production process by Fischer-Tropsch
**Calculated coefficients (inputs)**			
Synthetic fuel use	0.28	Gt/ 10^12_bus_km	Fuel consumption / fuel energy density
**Calculated coefficients (outputs)**			
Residual Emissions	0.89	Gt CO2e/ 10^12_bus_km	carbon dioxide to produce synfuel must be released during combustion. Residual emissions are thus: CO2 content of SynFuel x SynFuel consumption.

Table S271: Key assumptions for estimating the coefficients for synthetic-fuel powered bus transportation. References: Van Der Giesen et al. (2014); Correa et al. (2019); Mahmoud et al. (2016); Schmidt et al. (2016)

Activity	Process	Resource	Inputs	Outputs	Unit
BusUse	SynICE	Synfuel	**-0.014**		Gt/10^12pkm
BusUse	SynICE	NetEmissions		**0.044**	Gt_CO2e/10^12pkm
BusUse	SynICE	BusUse		**1.0**	10^12pkm/10^12pkm

Table S272: Chosen coefficients for synthetic-fuel powered bus transportation.

5.8.3.4 Battery Electric Bus

This delivery process is for Battery Electric Vehicle (BEV) bus passenger transport (Figure S111). Average fuel consumption is estimated based on the sources shown in Table S266. This value is combined with energy density and emissions factors to calculate the coefficients, as shown in Table S273. The derived coefficients are shown in Table S274.

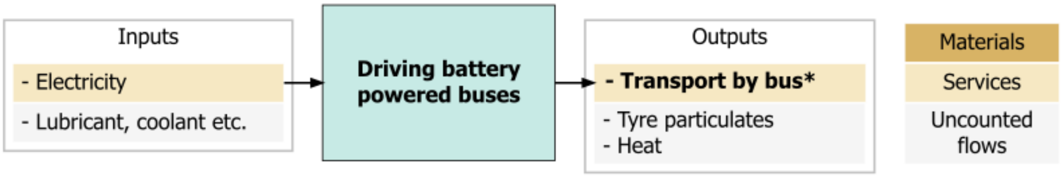

Figure S111: Inputs and outputs for transporting people by battery electric buses.

Property	Quantity	Unit	Justification
**Data and assumptions**			
Electricity use	4	MJ/ bus km	Correa, Muñoz & Rodriguez (2019); Mahmoud et al. (2016); Schmidt et al. (2016)
Passengers per bus	20	passengers	Similar to the assumptions used by Sigemund et al. (2017) and Correa, Muñoz & Rodriguez (2019)
**Calculated coefficients (inputs)**			
Electricity demand per passenger	0.20	MJ/ passenger bus km	(electricity use) / (passengers per bus)

Table S273: Key assumptions for estimating the coefficients for transportation by battery electric bus. References: Correa et al. (2019); Mahmoud et al. (2016)

Activity	Process	Resource	Inputs	Outputs	Unit
BusUse	BEV	Electricity	-0.20		EJ elec/10^12pkm
BusUse	BEV	BusUse		1.0	10^12pkm/10^12pkm

Table S274: Chosen coefficients for transportation by battery electric bus.

5.8.3.5 Hydrogen Fuel Cell Powered Bus

This delivery process is for Hydrogen Fuel Cell Electric Vehicle (HFCEV) bus transportation. The fuel consumption value is based on the sources shown in Table S266.

The key assumptions and calculations are shown in Table S275 and the chosen coefficients in Table S276.

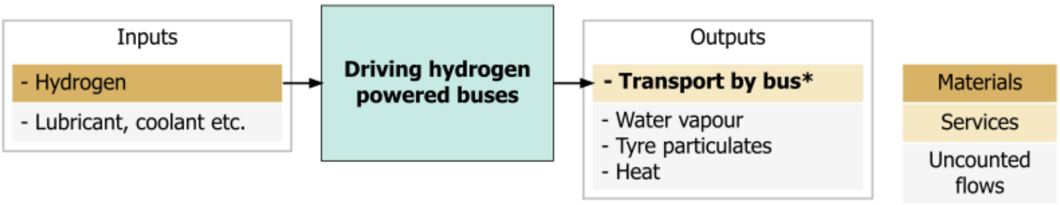

Figure S112: Inputs and outputs for transporting people by hydrogen-powered buses.

Property	Quantity	Unit	Justification
**Data and assumptions**			
Fuel consumption	11	MJ/ bus km	Correa, Muñoz & Rodriguez (2019); Mahmoud et al. (2016); Schmidt et al. (2016)
Passengers per bus	20	passengers	Similar to the assumptions used by Sigemund et al. (2017) and Correa, Muñoz & Rodriguez (2019)
Hydrogen energy density	120	EJ/Gt Hydogen	LHV - Giddey et al. (2013)
**Calculated coefficients (inputs)**			
Fuel demand	0.092	Gt/ 10^12_bus_km	Fuel consumption / fuel energy density

Table S275: Key assumptions for estimating the coefficients for passenger transportation by hydrogen powered bus. References: (Giddey et al., 2013), (Correa et al., 2019), (Mahmoud et al., 2016), (Schmidt et al., 2016)

Activity	Process	Resource	Inputs	Outputs	Unit
BusUse	HFCEV	Hydrogen	**-0.0046**		Gt/10^12pkm
BusUse	HFCEV	BusUse		**1.0**	10^12pkm/10^12pkm

Table S276: Chosen coefficients for transportation by hydrogen fuel cell electric bus.

5.8.4 Passenger transportation by rail

This activity describes passenger transportation by rail. The coefficients are derived from assumptions of energy intensity for for each delivery process (where each uses a different drive-train technology and/or fuel). Some data is provided in the form of energy intensity per train-km - for comparison this data is divided by an assumed passenger occupancy of 300 passengers per train. The value is chosen to reflect the significantly higher occupancy in countries such as India (around 1450) and China (around 900) compared to IEA member countries which had an average passenger load factor for 2018 of 130 (International Energy Agency, 2019a), (International Energy Agency, 2021b). Passenger occupancy rates also vary significantly by train type; the number of passengers may vary from around 100 to over 900 per train for metro and high speed trains in China, and between 100, 160 and 310 for conventional, metro and high-speed in Europe (International Energy Agency, 2019a).

The chosen energy intensity values, and the data on which they are based, are shown in Table S277.

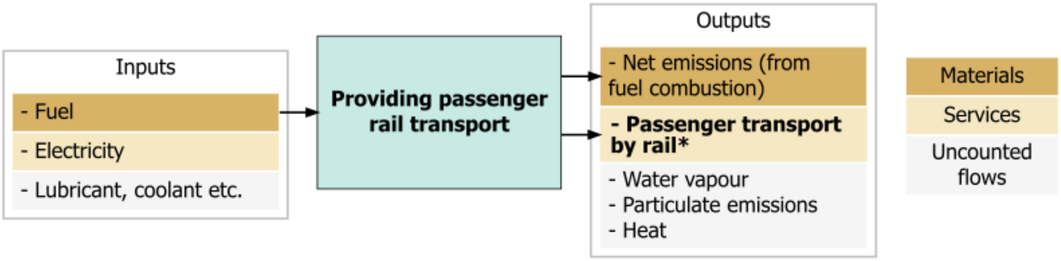

Figure S113: Inputs and outputs for transporting passengers by rail.

Activity	Delivery process	Detail
RailP	FFICE	Fossil Fuelled
RailP	Elec	Electrically Powered
RailP	BioICE	Biofuel Powered Internal Combustion Engine
RailP	SynICE	Synfuel Powered Internal Combustion Engine
RailP	HFCEV	Hydrogen Fuel Cell Powered

Table S278: Delivery processes for passenger transportation by rail.

5.8.4.1 Diesel-Fuelled (Conventional)

This delivery process is for conventional diesel-fuelled passenger rail transportation (Figure S114). These are derived based on the assumptions in Table S279. The data and assumptions which provide the basis for the fuel consumption estimate are outlined in Table S277. The derived coefficients are shown in Table S280.

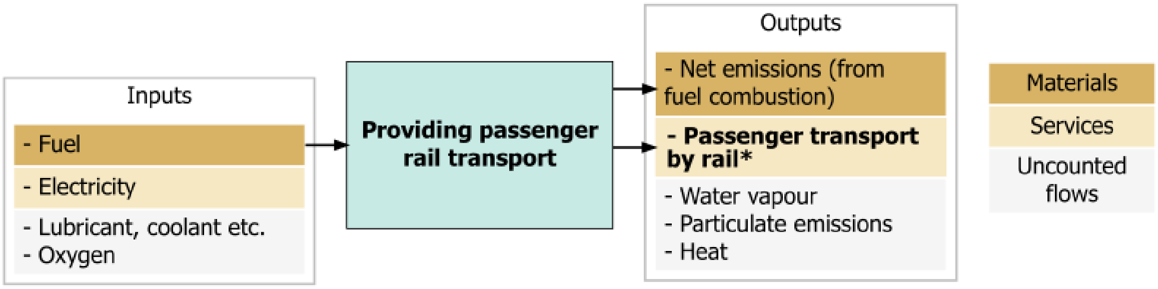

Figure S114: Inputs and outputs for transporting people by diesel-fuelled passenger rail.

Rail category	Drive/ fuel type	Fuel use, MJ/km	Note/ Source	Derived MJ/ p-km
Short distance - Germany	Diesel	60	Central value from Schmidt et al. (2016) - data ranges from 34-124 MJ/km	0.20
Regional trains	Diesel	68	Data as used by IEA Future of Rail report (2019) to generate future cost projections, Box 2.1	0.23
**Chosen value**	**Diesel**			**0.20**

Table S277: Summary of the data and chosen values for fuel consumption used in this analysis. Energy intensity values are calculated based on an assumed occupancy of 300 passengers/train with the exception of the 2018 value for IEA member countries which uses the IEA member average of 130 passengers/train References: (International Energy Agency, 2019a), (International Energy Agency, 2021b), (Schmidt et al., 2016), (Logan et al., 2020)

Property	Quantity	Unit	Justification
**Data and assumptions**			
Fuel consumption	0.20	MJ/ passenger km	Refer to the introduction to this section for the service: passenger rail
Diesel Energy Density	33	GJ/t	UK Government Conversion Factors
Diesel Emissions Factor	71	Mt CO2e/EJ	UK Government Conversion Factors (BEIS)
**Calculated coefficients (inputs)**			
Diesel fuel consumption	0.0060	Gt/ 10^12_p_km	Fuel consumption / fuel energy density
**Calculated coefficients (outputs)**			
Diesel residual emissions	0.014	Gt CO2e/ 10^12_p_km	Emissions factor x Fuel consumption

Table S279: Key assumptions for estimating the coefficients for conventional fossil-fuelled passenger rail transportation. References: (Department for Business Energy & Industrial Strategy, 2018)

Activity	Process	Resource	Inputs	Outputs	Unit
RailP	FFICE	Oil	-0.0060		Gt/10^12pkm
RailP	FFICE	NetEmissions		0.014	Gt_CO2e/10^12pkm
RailP	FFICE	RailP		1.0	10^12pkm/10^12pkm

Table S280: Chosen coefficients for conventional fossil-fuelled passenger rail transportation.

5.8.4.2 Electrified

This delivery process is for electrified passenger rail transportation (Figure S115). The data used to estimate the coefficients are given in Table S281 and the coefficients in Table S282.

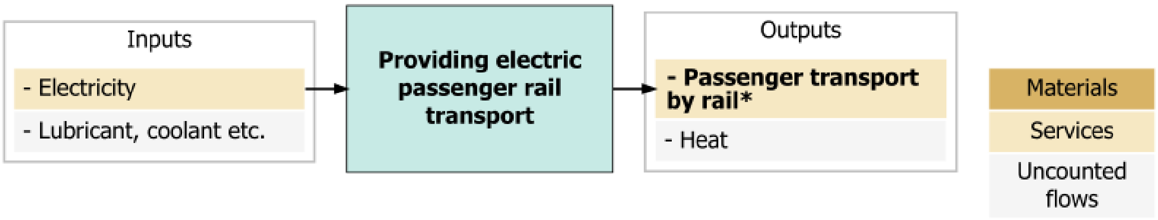

Figure S115: Inputs and outputs for transporting people by diesel-fuelled passenger rail.

Rail category	Drive/ fuel type	Fuel use, MJ/km	Note/ Source	Derived MJ/ p-km
High speed rail - Germany	Electricity	75	Central value of data from Schmidt et al. (2016) ranging between 45 and 103	0.25
Short distance - Germany	Electricity	45	Schmidt et al. (2016)	0.15
Regional trains	Connected-electric	21	As used by IEA Future of Rail (2019)	0.07
Regional trains	Battery-electric	22	As used by IEA Future of Rail (2019)	0.07
UK	Electric	16	Data used by Logan et al. (2020)	0.05
**Chosen value**	**Electrically driven**			**0.15**

Table S281: Summary of the data and chosen values for fuel consumption used in this analysis. Energy intensity values are calculated based on an assumed occupancy of 300 passengers/train with the exception of the 2018 value for IEA member countries which uses the IEA member average of 130 passengers/train References: (International Energy Agency, 2019a), (International Energy Agency, 2021b), (Schmidt et al., 2016), (Logan et al., 2020)

Activity	Process	Resource	Inputs	Outputs	Unit
RailP	Elec	Electricity	-0.15		EJ elec/10^12pkm
RailP	Elec	RailP		1.0	10^12pkm/10^12pkm

Table S282: Chosen coefficients for battery electric passenger rail transportation.

5.8.4.3 Biofuel Powered

This delivery process is for biofuel powered passenger rail transportation, assuming an engine similar to a conventional diesel engine (Figure S114). The efficiency of a locomotive powered by biofuel is assumed to be similar to a diesel powered locomotive. The process to produce biofuels is accounted for in Section 5.6.12.

The derived coefficients are derived based on the assumptions in Table S283.

Property	Quantity	Unit	Justification
**Data and assumptions**			
Fuel consumption	0.20	MJ/ passenger km	Refer to the introduction to this section for the service: passenger rail
Bio-fuel Energy Density	41	GJ/t	UK Government Conversion Factors (BEIS) give values from 27 (for bio-ethanol) to 44 (for Hydrogenated Veg Oil, HVO bio-diesel) GJ/t
Residual Emissions	0	Gt CO2e/ 10^12_p_km	Emissions from the carbon in biomass (and production of biomass) are accounted for in the biomass production process. Emissions from biofuel production are accounted for in the biofuel production process.
**Calculated coefficients (inputs)**			
Bio-fuel use	0.0049	Gt/ 10^12_p_km	Fuel consumption / fuel energy density

Table S283: Key assumptions for estimating the coefficients for biofuel powered passenger rail transportation. References: (Department for Business Energy & Industrial Strategy, 2018).

Activity	Process	Resource	Inputs	Outputs	Unit
RailP	BioICE	BioFuel	-0.0049		Gt/10^12pkm
RailP	BioICE	RailP		1.0	10^12pkm/10^12pkm

Table S284: Chosen coefficients for biofuel powered passenger rail transportation.

5.8.4.4 Synfuel Powered Internal Combustion Engine

This delivery process is for synthetic-fuel powered passenger rail transportation, assuming an internal combustion engine similar to a conventional fossil fuelled internal combustion engine (Figure S114). The efficiency of a locomotive powered by synthetic fuel is assumed to be similar to a diesel-fuelled locomotive. The process to produce synthetic hydrocarbon fuels is accounted for in Section 5.6.11.

The derived coefficients are derived based on the assumptions in Table S285.

Property	Quantity	Unit	Justification
**Data and assumptions**			
Fuel consumption	0.20	MJ/ passenger km	Refer to the introduction to this section for the service: passenger rail
Synthetic fuel Energy Density	43	GJ/t	Van Der Giesen et al. (2014)
CO2 input for Synfuel production	3.2	kg/ kg synfuel	From Synfuel production process by Fischer-Tropsch
**Calculated coefficients (inputs)**			
Synthetic fuel use	0.0046	Gt/ 10^12_p_km	Fuel consumption / fuel energy density
**Calculated coefficients (outputs)**			
Residual Emissions	0.015	Gt CO2e/ 10^12_p_km	carbon dioxide to produce synfuel must be released during combustion. Residual emissions are thus: CO2 content of SynFuel x SynFuel consumption.

Table S285: Key assumptions for estimating the coefficients for synthetic-fuel powered passenger rail transportation. References: (Van Der Giesen et al., 2014)

Activity	Process	Resource	Inputs	Outputs	Unit
RailP	SynICE	Synfuel	-0.0046		Gt/10^12pkm
RailP	SynICE	NetEmissions		0.015	Gt_CO2e/10^12pkm
RailP	SynICE	RailP		1.0	10^12pkm/10^12pkm

Table S286: Chosen coefficients for synthetic-fuel powered passenger rail transportation.

5.8.4.5 Hydrogen Powered

This delivery process is for hydrogen powered passenger transportation by rail (Figure S116). The derived coefficients are derived from the data and assumptions in Table S287 and Table S288, and shown in Table S289.

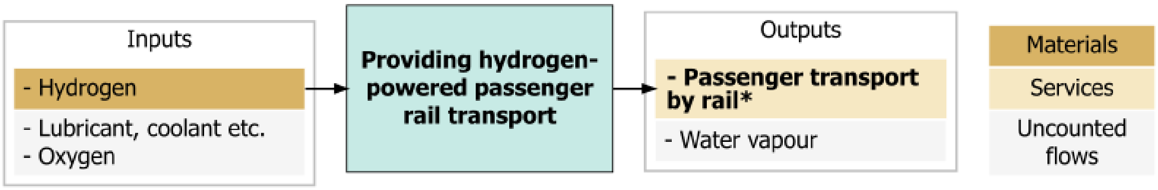

Figure S116: Inputs and outputs for transporting people by hydrogen-powered passenger rail.

Rail category	Drive/ fuel type	Fuel use, MJ/km	Note/ Source	Derived MJ/ p-km
Short distance - Germany	Hydrogen	21	Schmidt et al. (2016)	0.07
Regional trains	Hydrogen fuel cell hybrid	33	As used by IEA Future of Rail (2019)	0.11
UK	Hydrogen	36	Data used by Logan et al. (2020)	0.12
**Chosen value**	**Hydrogen**			**0.11**

Table S287: Summary of the data and chosen values for fuel consumption used in this analysis. Energy intensity values are calculated based on an assumed occupancy of 300 passengers/train with the exception of the 2018 value for IEA member countries which uses the IEA member average of 130 passengers/train References: (International Energy Agency, 2019a), (International Energy Agency, 2021b), (Schmidt et al., 2016), (Logan et al., 2020)

Property	Quantity	Unit	Justification
Fuel consumption	0.11	MJ/ passenger km	Refer to the introduction to this section for the service: passenger rail
Hydrogen energy density	120	EJ/Gt Hydogen	LHV - Giddey et al. (2013)
Fuel demand	0.00092	Gt/ 10^12_p_km	Fuel consumption / fuel energy density

Table S288: Key assumptions for estimating the coefficients for hydrogen powered passenger transportation by rail. References: (Giddey et al., 2013)

Activity	Process	Resource	Inputs	Outputs	Unit
RailP	HFCEV	Hydrogen	-0.00092		Gt/10^12pkm
RailP	HFCEV	RailP		1.0	10^12pkm/10^12pkm

Table S289: Chosen coefficients for hydrogen-fuelled passenger transportation by rail.

5.8.5 Aviation

This activity describes passenger transportation by aeroplane. Cargo transport by plane is not accounted for within the model as it is assumed that the proportion of air-freight is small compared to other modes of freight transportation, and that air-freight is small compared to passenger aviation. The second assumption is weak based on current use; in 2018 Graver et al. (2019) attributed 19% of total aviation emissions to freight (in the "belly" of passenger jets and dedicated operations), and statistics from the International Air Transport Association show consistent growth in air cargo demand since 2021 (IATA, 2024). Excluding this activity will result in lower accumulated resource demands than may be expected in reality. Other forms of aviation (including military and helicopter flights) are also not accounted for here and would increase the accumulated resource demands further.

Hydrogen-fuelled and electric aviation may differ from conventional kerosene-fulled gas-turbine aviation in two key ways. Firstly these options are likely to require alternative aircraft structures and configurations compared to a gas-turbine powered plane to account for the differing volume, mass and shape of the energy storage system. Additionally, alternative fuels may not be used for the same mission profiles which are used today. Electric aircraft in particular are not viable for longer routes since the weight of the battery would need to be carried for the whole distance (compared to chemical energy sources whose weight decreases through the flight). Electric aircraft are currently only proposed for commuter and regional routes (<~1000 km) (Gnadt et al., 2019). The values chosen here assume that electric aircraft would only operate for these journeys (and so only operate in their most efficient range, rather than making long trips of many small segments).

The linear coefficients derived here for aviation are accurate only when considering aviation at a global level since the energy intensity varies significantly with the flight length (dominant fuel consumption is during the take-off segment of the flight) and with the aircraft type (aircraft with more seats have a lower per-passenger energy intensity).

As listed in Table S290, there are two delivery processes for each different fuel proposed for aviation (with the exception of battery electric); one delivery process accounts only for direct CO2 emissions, while the other accounts for the total environmental warming impact. When aeroplanes fly at altitude, they emit gases and create physical changes in the surrounding atmosphere (pressure and movement). Non-CO2 emissions, including water vapour, soot particles and gases such as oxides of nitrogen (NOx) cause a wide range of chemical and physical processes in the atmosphere, varying with altitude and weather conditions; these are complex indirect effects which lead to an overall warming impact (Lee et al., 2021). Aviation is not the only activity inducing complex indirect effects on the atmosphere (Eyring et al., 2010) but aviation indirect warming impacts are significant and so the option to account for them is included in the model (although these processes are not used for the main example scenarios in the main paper).

Activity	Delivery process	Detail
Aviation	JetA	JetA kerosene conventional aviation - direct CO2 emissions only
Aviation	JetAT	JetA kerosene conventional aviation - including non-CO2 warming impacts
Aviation	LH2	Liquified hydrogen aviation - direct CO2 emissions only
Aviation	LH2T	Liquified hydrogen aviation - including non-CO2 warming impacts
Aviation	LNG	Liquified natural gas fuelled aviation - direct CO2 emissions only
Aviation	LNGT	Liquified natural gas fuelled aviation - including non-CO2 warming impacts
Aviation	ElecT	Battery electric aviation (only valid for short flights)
Aviation	PtL	Synthetic fuel powered aviation - direct CO2 emissions only
Aviation	PtLT	Synthetic fuel powered aviation - including non-CO2 warming impacts
Aviation	Bio	Biofuel powered aviation - direct CO2 emissions only
Aviation	BioT	Biofuel powered aviation - including non-CO2 warming impacts

Table S290: Delivery processes for passenger transportation by aeroplane.

Coefficients for each delivery process are derived by estimating the climate impact per unit of fuel consumed (Table S291), and the quantity of fuel per passenger-km of aviation (Table S292). Fuel demands for each process are estimated by considering the relative energy demands compared to a baseline of conventional kerosene-fulled aviation. The fuel consumption of conventional aviation is estimated as a 10% efficiency improvement from the global average passenger aviation energy intensity in 2018. This improvement in energy intensity is conservative compared to the assumed reduction of of 1.3% per year, Dray et al. (2022) derive from assumptions about aircraft improvements and fleet replacement. The 2018 fuel consumption is estimated from the bottom-up study by Graver et al. (2019) who find that global passenger aviation of 8.5 $\times 10^{12}$ RPK ($Revenue\ Passenger\ Km$) was accountable for 747 $Mt\ CO_2$ (see Table S293). A summary of the fuel consumption and emissions factors used for the delivery processes for aviation are given in Table S292.

The total climate warming associated with kerosene-fuelled aviation is highly uncertain (visible in the large uncertainty ranges in Table S291) but several studies have now quantified the effect of individual or multiple climate forcers, in terms of a variety of metrics. Lee et al. (2021) estimate the total impact to be around 1.7 times greater than CO2 alone (considering aviation in 2018 with a GWP-100 metric). Most non-CO2 emissions are attributed to the formation of contrails by both Dray et al. (2022) and Lee et al. (2021). These could be mitigated to some extent by changing the cruise flight altitude to avoid regions where contrails would form.

Emissions coefficients for all aviation processes are estimated from the values derived by Dray et al. (2022), shown in Table S291. Dray et al. (2022) quantified the total warming impact of alternative fuels by estimating adjustment factors against kerosene-powered aviation for each climate forcer produced by each fuel. The values used by Dray et al. (2022) assume that "50% of contrail length can be avoided at a 1% increase in fuel burn" estimated from a review of five studies. Contrail avoidance is not included in the delivery processes currently quantified in the model - this why model values are at the upper end of the range suggested by Dray et al. (2022) Table S291. Delivery processes including this strategy could be added at a later date.

	Climate impact intensity, gCO2(eq) MJ-1			
	Values used by Dray et al. (2022) (inclusive of contrail avoidance)		Assigned values for this analysis (no contrail avoidance)	
Fuel	TTW CO2	Non-CO2	CO2 only	Total warming impacts
Jet-A	73.2	30.8 (9.4-54)	72	127
Hydrogen	0.0	35.1 (11-68)	0	68
LNG	56.4	39.1 (13-73)	56	129
Electricity	0.0	0	0	0
PtL	70.4	23.7 (6-47)	70	117
Biofuel	70.4	23.7 (6-47)	0	117

Note
Biofuel shows zero direct CO2 emissions since emissions from carbon in biomass are accounted for in biomass production.
Values in brackets show the uncertainty range.
TTW: Tank-To-Wake; LNG: Liquified Natural Gas; PtL: Power-to-Liquid Fuel (SynFuel)

Table S291: Summary of data and chosen values for emissions factors for aviation. The ratio between total warming and CO2 warming for kerosene aviation is chosen to be consistent with the value found by Lee et al. (2021) (1.7). Note that biofuel is given an emissions factor of zero, consistent with the carbon neutral assumption of biomass-use explained in the SI Part 1 and in part 1 of the SI. CO2 fuel emissions factors used here are chosen to also be consistent with values used in other sectors, as described in the document assumptions (the SI Part 2). Other references: Dray et al. (2022).

Fuel	Relative fuel burn	Fuel consumption, g fuel/p-km	Fuel consumption, MJ fuel/p-km	Justification
Jet-A Kerosene		25	1.1	Assuming a 9% efficiency improvement of the global average for 2018, estimated from total RPK and CO2 emissions from a bottom-up analysis of 2018 global aviation by Graver et al. (2019)
Liquified-Hydrogen	1	9.2	1.1	Scaled from the Jet-A estimate using the same relative fuel burn as used by Dray et al. (2022)
LNG	1	24	1.1	Scaled from the Jet-A estimate using the same relative fuel burn as used by Dray et al. (2022)
Electricity	0.72		0.8	Relative fuel burn estimated from the energy intensity values used by Schaefer et al. (2019): 180 Wh/RPK for an electric aircraft against a reference aircraft of 0.9 MJ fuel/RPK
PtL	1	25	1.1	Assumed to be the same as Jet-A
Biofuel	1	25	1.1	Assumed to be the same as Jet-A

Note
LNG: Liquified Natural Gas; PtL: Power-to-Liquid Fuel (SynFuel); RPK: Revenue Passenger Km

Table S293: Summary of estimated fuel consumption for each aviation delivery process. The 10% efficiency compared to today's values is assumed given that the efficiency of new aircraft could be expected to improve by around 1% per year. References: Graver et al. (2019); Dray et al. (2022); Schäfer et al. (2019)

	Fuel consumption		Residual Emissions, Gt CO2e/ 10^12_p_km	
Delivery Processes	Value	Units (per 10^12 passenger km)	CO2 only	Total warming impacts
Jet-A Kerosene	0.025	Gt	0.079	0.140
Hydrogen	0.009	Gt	0	0.075
LNG	0.024	Gt	0.062	0.142
Electricity	0.793	EJ	0	0
PtL	0.025	Gt	0.077	0.129
Biofuel	0.025	Gt	0	0.129

Note
LNG: Liquified Natural Gas; PtL: Power-to-Liquid Fuel (SynFuel)

Table S292: Summary of fuel consumption and emissions values used to derive model coefficients based on the data and assumptions in Table S291 and Table S293.

5.8.5.1 Conventional Jet-A Kerosene Aviation (direct CO2 only)

This process describes transporting passengers by kerosene-fuelled gas-turbine-powered aeroplanes, excluding non-CO2 climate impacts (Figure S117). The coefficients are estimated using the data and approach outlined at the start of this section (see Table S292). The derived values are in Table S294.

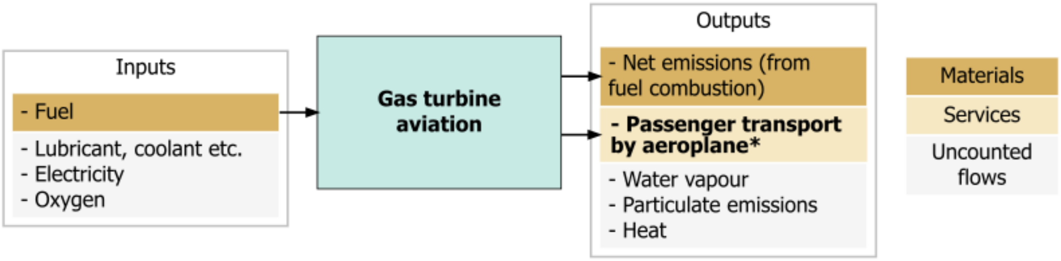

Figure S117: Inputs and outputs for transporting passengers by gas-turbine fuelled aeroplanes.

Activity	Process	Resource	Inputs	Outputs	Unit
Aviation	JetA	Oil	-0.025		Gt/10^12pkm
Aviation	JetA	NetEmissions		0.080	Gt_CO2e/10^12pkm
Aviation	JetA	Aviation		1.0	10^12pkm/10^12pkm

Table S294: Chosen coefficients for conventional fossil-fuelled aviation, considering CO2 emissions only.

5.8.5.2 Conventional Jet-A Kerosene Aviation (total climate impact)

This process describes transporting passengers by kerosene-fuelled gas-turbine-powered aeroplanes, including non-CO2 climate impacts (Figure S118). The coefficients are estimated using the data and approach outlined at the start of this section (see Table S292). The derived values are in Table S295.

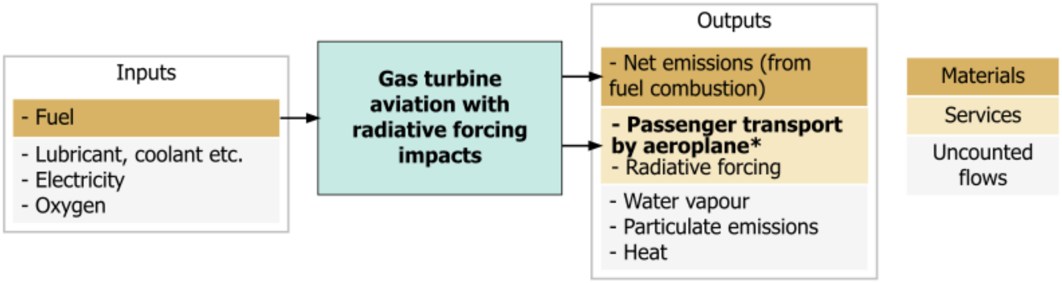

Figure S118: Inputs and outputs for transporting passengers by gas-turbine fuelled aeroplanes including non-CO2 climate impacts.

Activity	Process	Resource	Inputs	Outputs	Unit
Aviation	JetAT	Oil	-0.025		Gt/10^12pkm
Aviation	JetAT	NetEmissions		0.14	Gt_CO2e/10^12pkm
Aviation	JetAT	Aviation		1.0	10^12pkm/10^12pkm

Table S295: Chosen coefficients for conventional fossil-fuelled aviation, considering the total climate impact.

5.8.5.3 Hydrogen Powered Aviation (direct CO2 only)

This process describes transporting passengers by hydrogen-fuelled gas-turbine aeroplanes excluding non-CO2 climate impacts (Figure S119). The coefficients are estimated using the data and approach outlined at the start of this section (see Table S292.

Additionally electricity demand is attributed to this process to account for the energy to liquify hydrogen. This is assumed to be 22 GJ/t LH2, consistent with the value used for liquified hydrogen fuel in shipping (and assumed by Gardiner & Satyapal (2009)). 22 GJ/t LH2 is also consistent with the 2050 liquification energy demand assumed by Dray et al. (2022) (falling from 36 GJ/t LH2 in 2020). The derived values are in Table S296.

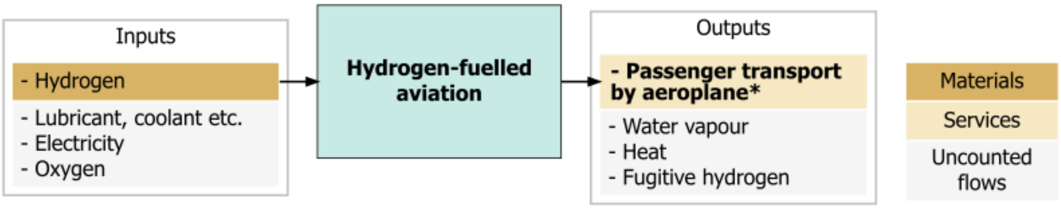

Figure S119: Inputs and outputs for transporting passengers by hydrogen-powered planes including non-CO2 climate impacts.

Activity	Process	Resource	Inputs	Outputs	Unit
Aviation	LH2	Hydrogen	-0.0093		Gt/10^12pkm
Aviation	LH2	Electricity	-0.20		EJ/10^12pkm
Aviation	LH2	Aviation		1.0	10^12pkm/10^12pkm

Table S296: Chosen coefficients for hydrogen-fuelled aviation, considering CO2 emissions only.

5.8.5.4 Hydrogen Powered Aviation (total climate impact)

This process describes transporting passengers by hydrogen-fuelled gas-turbine aeroplanes including non-CO2 climate impacts (Figure S120). The coefficients are estimated using the data and approach outlined at the start of this section (see Table S292.

Additionally, electricity demand is attributed to this process to account for the energy to liquify hydrogen. This is assumed to be 22 GJ/t LH2, consistent with the value used for liquified hydrogen fuel in shipping (and assumed by Gardiner & Satyapal (2009)). 22 GJ/t LH2 is also consistent with the 2050 liquification energy demand assumed by Dray et al. (2022) (falling from 36 GJ/t LH2 in 2020). The derived values are in Table S297.

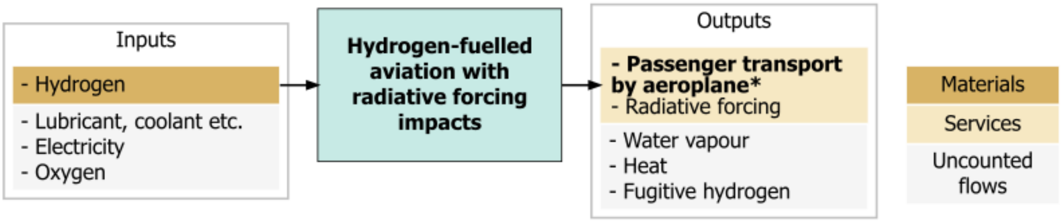

Figure S120: Inputs and outputs for transporting passengers by hydrogen-powered planes including non-CO2 climate impacts.

Activity	Process	Resource	Inputs	Outputs	Unit
Aviation	LH2T	Hydrogen	-0.0093		Gt/10^12pkm
Aviation	LH2T	NetEmissions		0.076	Gt_CO2e/10^12pkm
Aviation	LH2T	Electricity	-0.20		EJ/10^12pkm
Aviation	LH2T	Aviation		1.0	10^12pkm/10^12pkm

Table S297: Chosen coefficients for hydrogen-fuelled aviation, considering the total climate impact.

5.8.5.5 Liquified Natural Gas (LNG) Powered Aviation (direct CO2 only)

This process describes transporting passengers by liquified natural gas (LNG) turbine powered aeroplanes excluding non-CO2 climate impacts (Figure S117). The coefficients are estimated using the data and approach outlined at the start of this section (see Table S292).

Additional energy demand (electricity) is attributed to this process to account for liquifaction. It has been assumed that energy for liquifaction is 10% of the fuel supplied, based on industry published values for low-energy liquification (CNBC, 2018). The derived values are in Table S298.

Activity	Process	Resource	Inputs	Outputs	Unit
Aviation	LNG	Methane	-0.027		Gt/10^12pkm
Aviation	LNG	NetEmissions		0.062	Gt_CO2e/10^12pkm
Aviation	LNG	Aviation		1.0	10^12pkm/10^12pkm

Table S298: Chosen coefficients for Liquified Natural Gas (LNG)-fuelled aviation, considering CO2 emissions only.

5.8.5.6 Liquified Natural Gas (LNG) Powered Aviation (total climate impact)

This process describes transporting passengers by liquified natural gas (LNG) turbine powered aeroplanes including non-CO2 climate impacts (Figure S118). The coefficients are estimated using the data and approach outlined at the start of this section (see Table S292).

Additional energy demand (electricity) is attributed to this process to account for liquifaction. It has been assumed that energy for liquifaction is 10% of the fuel supplied, based on industry published values for low-energy liquification (CNBC, 2018). The derived values are in Table S299.

Activity	Process	Resource	Inputs	Outputs	Unit
Aviation	LNGT	Methane	-0.027		Gt/10^12pkm
Aviation	LNGT	NetEmissions		0.14	Gt_CO2e/10^12pkm
Aviation	LNGT	Aviation		1.0	10^12pkm/10^12pkm

Table S299: Chosen coefficients for Liquified Natural Gas (LNG)-fuelled aviation, considering the total climate impact.

5.8.5.7 Battery Electric Aviation (total climate impact)

This delivery process describes the transportation of passengers by battery electric aeroplanes (Figure S121). Electric aircraft are only suitable for shorter flights (Gnadt et al., 2019). As such, assume here that electric aviation is only used for a small fraction of aviation provision, such that it can operate at the design efficiency. The coefficients for battery electric aviation are estimated using the data and approach outlined at the start of this section (see Table S292). The derived values are in Table S300.

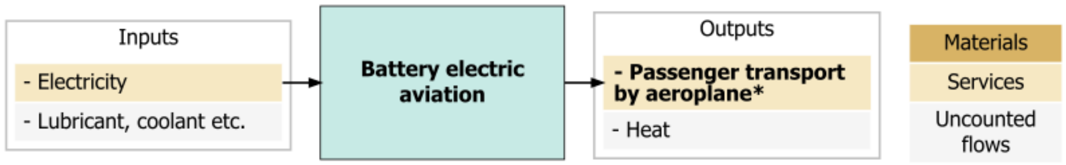

Figure S121: Inputs and outputs for transporting passengers by battery electric aeroplanes.

Activity	Process	Resource	Inputs	Outputs	Unit
Aviation	ElecT	Electricity	-0.80		EJ/10^12pkm
Aviation	ElecT	Aviation		1.0	10^12pkm/10^12pkm

Table S300: Chosen coefficients for battery electric powered aviation (commuter and regional routes only).

5.8.5.8 Synthetic Fuel Aviation (direct CO2 only)

This process describes the transportation of passengers by aeroplane using synthetic fuel gas turbine aeroplanes (Figure S117). The coefficients for Synthetic Fuel aviation excluding non-CO2 climate impacts are estimated using the data and approach outlined at the start of this section (see Table S292). The derived values are in Table S301.

Activity	Process	Resource	Inputs	Outputs	Unit
Aviation	PtL	Synfuel	**-0.025**		Gt/10^12pkm
Aviation	PtL	NetEmissions		**0.078**	Gt_CO2e/10^12pkm
Aviation	PtL	Aviation		**1.0**	10^12pkm/10^12pkm

Table S301: Chosen coefficients for synthetic fuel aviation, considering CO2 emissions only.

5.8.5.9 Synthetic Fuel Aviation (total climate impact)

This process describes the transportation of passengers by aeroplane using synthetic fuel gas turbine aeroplanes, including non-CO2 climate impacts (Figure S118). The coefficients for Synthetic Fuel aviation including non-CO2 climate impacts are estimated using the data and approach outlined at the start of this section (see Table S292). The derived values are in Table S302.

Activity	Process	Resource	Inputs	Outputs	Unit
Aviation	PtLT	Synfuel	**-0.025**		Gt/10^12pkm
Aviation	PtLT	NetEmissions		**0.13**	Gt_CO2e/10^12pkm
Aviation	PtLT	Aviation		**1.0**	10^12pkm/10^12pkm

Table S302: Chosen coefficients for synthetic fuel aviation, considering the total climate impact.

5.8.5.10 Biofuel Aviation (direct CO2 only)

This process describes the transportation of passengers by aeroplane using biofuel-powered gas turbine aeroplanes (Figure S122). The coefficients for biofuel aviation excluding non-CO2 climate impacts are estimated using the data and approach outlined at the start of this section (see Table S292). The derived values are in Table S303.

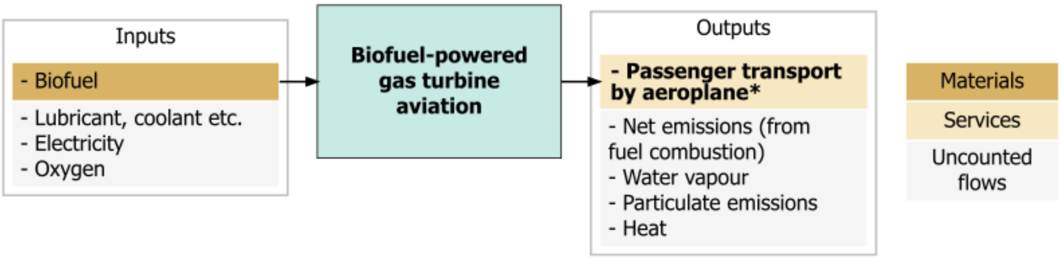

Figure S122: Inputs and outputs for transporting passengers by biofuel-powered gas-turbine fuelled aeroplanes.

Activity	Process	Resource	Inputs	Outputs	Unit
Aviation	Bio	BioFuel	-0.025		Gt/10^12pkm
Aviation	Bio	Aviation		1.0	10^12pkm/10^12pkm

Table S303: Chosen coefficients for biofuel aviation, considering CO2 emissions only.

5.8.5.11 Biofuel Aviation (total climate impact)

This process describes the transportation of passengers by aeroplane using biofuel-powered gas turbine aeroplanes, including non-CO2 climate impacts (Figure S123).

The coefficients for biofuel aviation including non-CO2 climate impacts are estimated using the data and approach outlined at the start of this section (see Table S292). The derived values are in Table S304.

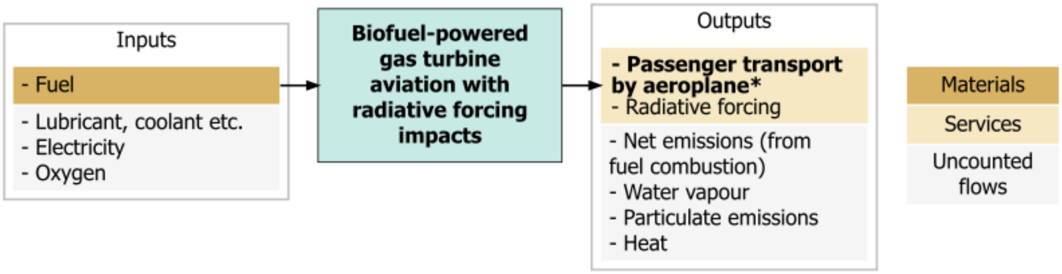

Figure S123: Inputs and outputs for transporting passengers by biofuel-powered gas-turbine fuelled aeroplanes including non-CO2 climate impacts.

Activity	Process	Resource	Inputs	Outputs	Unit
Aviation	BioT	Oil	-0.025		Gt/10^12pkm
Aviation	BioT	NetEmissions		0.13	Gt_CO2e/10^12pkm
Aviation	BioT	Aviation		1.0	10^12pkm/10^12pkm

Table S304: Chosen coefficients for biofuel aviation, considering the total climate impact.

5.8.6 Shipping

This activity describes international freight transportation by ship. Cargo-carrying vessels (including bulk vessels, container vessels and oil tankers) account for around 90% of fuel consumption in shipping (DNV GL, 2018); the remaining activities, including passenger transportation, fishing and offshore support, account for the residual 10%. As such, only freight transport is considered here. Oil transportation demands around 1/4 of the total fuel of the world cargo fleet (Figure 2.2.1 of DNV GL (2018)). This could be attributed to the resource demands for petrochemicals but is included within the category of shipping at this stage.

The energy intensity of shipping varies significantly dependent on the ship design, size, route and environmental conditions, as well as the engine-technology and load. Each delivery process here (listed in Table S305) represents an alternative fuel and/or engine technology, and the load is accounted for in the activity metric (tonne-km); no other factors are considered.

Battery-powered shipping is not included within this section as battery-powered vessels can only viably account for a small proportion of marine energy consumption - larger ships or longer ranges would require a batteries with a greater efficiency penalty. According to the Mærsk Mc-Kinney Møller Center for Zero Carbon Shipping (2021), battery-powered navigation may be suitable for inland and coastal operations but not for international shipping.

The coefficients are derived based on an estimated energy intensity of fossil-fuelled shipping from historical data (Table S306), scaled by energy demand multipliers for each of the different delivery processes (Table S307). The energy demand multipliers are estimated based on the efficiency of the engine system, and any impact on the cargo carrying capacity - for lower volume-density fuels the vessel would need to reduce the cargo to account for an increased volume of fuel, or increase the number of bunkering stops, increasing the overall energy intensity.

Activity	Delivery process	Detail
Shipping	FFICE	Fossil Fuelled
Shipping	LNG	Liquified Natural Gas Internal Combustion Engine
Shipping	BioICE	Biofuel Powered Internal Combustion Engine
Shipping	HFCEV	Hydrogen Fuel Cell Powered
Shipping	Ammonia	Ammonia Powered Internal Combustion Engine
Shipping	SynICE	Synfuel Powered Internal Combustion Engine

Table S305: Delivery processes for freight transportation by ship.

Property	Energy Intensity, MJ/t-km	Notes
Global cargo shipping (2016)	0.10	Derived from cargo fleet trade quantity and fleet fuel consumption, from Table 3.3.2 and Fig 2.1.1, DNV-GL (2018)
US waterbourne commerce on taxable waterways, 2010-2014	0.14	Average value from US transport statistics, Davis and Boundy (2022)
Lower bound for shipping and waterways of six diverse countries	0.04	Approximated from Fig 1, Gucwa and Schaefer (2013)
Upper bound	0.12	As for lower bound
**Chosen value for fossil-fuel shipping**	**0.10**	

Table S306: Summary of data considered to estimate energy intensity of fossil-fuelled shipping. The energy consumption is estimated by considering the effect of changing technologies on both the engine efficiency and on the cargo carrying capacity. References: DNV GL (2018); Davis et al. (2018); Gucwa & Schäfer (2013)

Property	Hydrogen fuel cell	Hydrogen ICE	Ammonia	Diesel	LNG	Justification
**Data and assumptions**						
Cargo capacity impact	-10%	-9%	-4%	1%	-1%	From Fig 6., Lloyd's Register and UMAS (2020). Note that the diesel ship has a small increase in cargo capacity, reflecting a 20% reduction in range against the reference ship.
Energy demand mutiplier (cargo)	1.11	1.10	1.04	0.99	1.01	1/(1 + cargo impact)
Engine system efficiency	45%	30%	50%	50%	50%	Seddiek et al. (2015), Balcombe et al. (2019), Raucci (2017) & Machaj et al. (2022)
**Calculations**						
Energy demand mutiplier (engine)	1.11	1.67	1.00	1.00	1.00	Diesel engine efficiency/engine efficiency
Total fuel demand multiplier	1.23	1.83	1.04	0.99	1.01	Cargo x Engine Energy Demand Multiplier
**Energy consumption**						
**MJ/t-km**	**0.12**	**0.18**	**0.10**	**0.10**	**0.10**	**Fossil-fuel shipping energy intensity x total fuel demand multiplier**

Note:
Both hydrogen options are based on liquid hydrogen fuel.
ICE: Internal Combustion Engine; LNG: Liquified Natural Gas.

Table S307: Summary of the approach and data used to estimate fuel consumption for shipping. Note that hydrogen ICE is included here for reference but has not been included in the delivery processes since hydrogen powered shipping using fuel cells is significantly more efficient. References: Lloyd's Register & UMAS (2020); Seddiek et al. (2015); Balcombe et al. (2019); Raucci et al. (2017); Machaj et al. (2022)

5.8.6.1 Fossil-Fuelled (Conventional)

This delivery process describes fossil-fuelled freight transport by long distance shipping. The fuel consumption value is based on the sources shown in Table S307.

The derived coefficients are shown in Table S308. These are derived based on the assumptions in Table S309.

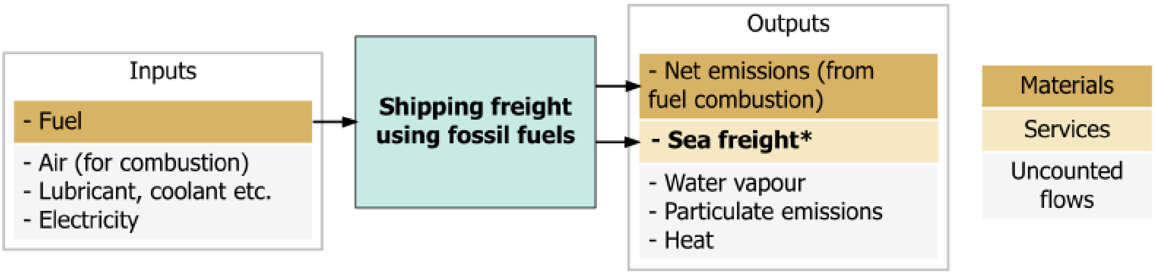

Figure S124: Inputs and outputs for shipping freight, powered by fossil fuels.

Property	Quantity	Unit	Justification
**Data and assumptions**			
Fuel consumption	0.10	MJ/ tonne km	Refer to the introduction to this section for the service: Shipping
Fuel Energy Density	41	GJ/t	UK Government Conversion Factors (BEIS)
Emissions Factor	77	Mt CO2e/EJ	UK Government Conversion Factors (BEIS)
**Calculated coefficients (inputs)**			
Fuel consumption	0.0024	Gt/ 10^12_t_km	Fuel consumption / fuel energy density
**Calculated coefficients (outputs)**			
Residual emissions	0.0076	Gt CO2e/ 10^12_t_km	Emissions factor x Fuel consumption

Table S309: Key assumptions for estimating the coefficients for conventional fossil-fuelled shipping. See Table S307 for the justification of the fuel consumption value. References: Department for Business Energy & Industrial Strategy (2018)

Activity	Process	Resource	Inputs	Outputs	Unit
Shipping	FFICE	Oil	-0.0024		Gt/10^12tkm
Shipping	FFICE	NetEmissions		0.0076	Gt_CO2e/10^12tkm
Shipping	FFICE	Shipping		1.0	10^12tkm/10^12tkm

Table S308: Chosen coefficients for conventional fossil-fuelled shipping.

5.8.6.2 LNG-Fuelled

This delivery process is for LNG-fuelled freight transport by long distance shipping. The fuel consumption value is based on the sources shown in Table S307, and the coefficients are derived as described in Table S310.

Property	Quantity	Unit	Justification
**Data and assumptions**			
Fuel consumption	0.10	MJ/ tonne km	Refer to the introduction to this section for the service: Shipping
Fuel Energy Density	45	GJ/t	UK Government Conversion Factors (BEIS)
Fuel consumption	0.0022	Gt/ 10^12_t_km	Fuel consumption / fuel energy density
Emissions Factor	57	Mt CO2e/EJ	UK Government Conversion Factors (BEIS)
Liquifaction energy	10%	of input fuel	https://www.cnbc.com/advertorial/2018/10/08/innovation-using-less-energy-to-liquefy-natural-gas.html
**Calculated coefficients (inputs)**			
Total fuel demand including liquification	0.0025	Gt/ 10^12_t_km	Fuel consumption/ (1 - liquifaction energy %)
**Calculated coefficients (outputs)**			
Residual emissions	0.006	Gt CO2e/ 10^12_t_km	Emissions factor x Total fuel demand

Table S310: Key assumptions for estimating the coefficients for LNG-fuelled shipping. See Table S307 for the justification of the fuel consumption value. References: Department for Business Energy & Industrial Strategy (2018)

Activity	Process	Resource	Inputs	Outputs	Unit
Shipping	LNG	Methane	**-0.0022**		Gt/10^12tkm
Shipping	LNG	NetEmissions		**0.0057**	Gt_CO2e/10^12tkm
Shipping	LNG	Shipping		**1.0**	10^12tkm/10^12tkm

Table S311: Chosen coefficients for conventional LNG-fuelled shipping.

5.8.6.3 Biofuel Powered Internal Combustion Engine

This delivery process is for biofuel powered freight transport by long distance shipping. The efficiency of an ICE (Internal Combustion Engine) vessel powered by biofuel is assumed to be similar to a diesel vessel. The process to produce biofuels is accounted for in Section 5.6.12.

The derived coefficients are shown in Table S312. These are derived based on the assumptions in Table S313.

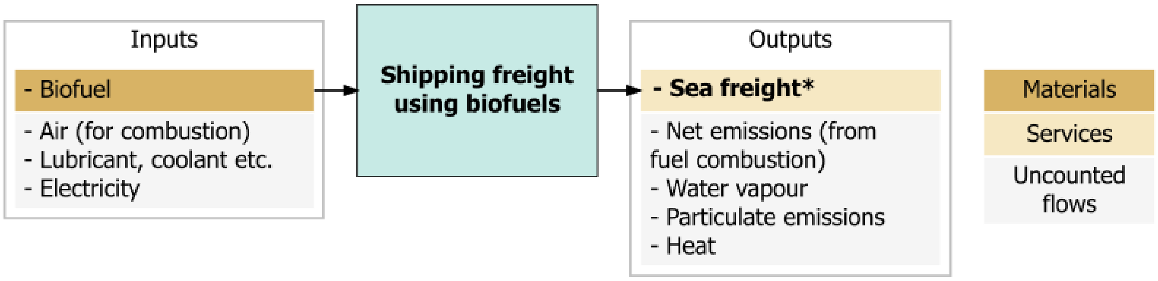

Figure S125: Inputs and outputs for shipping freight, powered by biofuels.

Property	Quantity	Unit	Justification
**Data and assumptions**			
Fuel consumption	0.10	MJ/ tonne km	Refer to the introduction to this section for the service: Shipping
Bio-fuel Energy Density	41	GJ/t	UK Government Conversion Factors (BEIS) give values from 27 (for bio-ethanol) to 44 (for Hydrogenated Veg Oil, HVO bio-diesel) GJ/t
**Calculated coefficients (inputs)**			
Bio-fuel use	0.0024	Gt/ 10^12_t_km	Fuel consumption / fuel energy density
**Calculated coefficients (outputs)**			
Residual Emissions	0	Gt CO2e/ 10^12_t_km	Emissions from the carbon in biomass (and production of biomass) are accounted for in the biomass production process. Emissions from biofuel production are accounted for in the biofuel production process.

Table S313: Key assumptions for estimating the coefficients for biofuel powered shipping. See Table S307 for the justification of the fuel consumption value. References: Department for Business Energy & Industrial Strategy (2018)

Activity	Process	Resource	Inputs	Outputs	Unit
Shipping	BioICE	BioFuel	**-0.0024**		Gt/10^12tkm
Shipping	BioICE	Shipping		**1.0**	10^12tkm/10^12tkm

Table S312: Chosen coefficients for biofuel powered shipping.

5.8.6.4 Hydrogen Fuel Cell Powered

This delivery process is for hydrogen fuel cell electric long distance freight shipping. The fuel consumption value is based on the sources shown in Table S307.

The derived coefficients are shown in Table S314. These are derived based on the assumptions in Table S315.

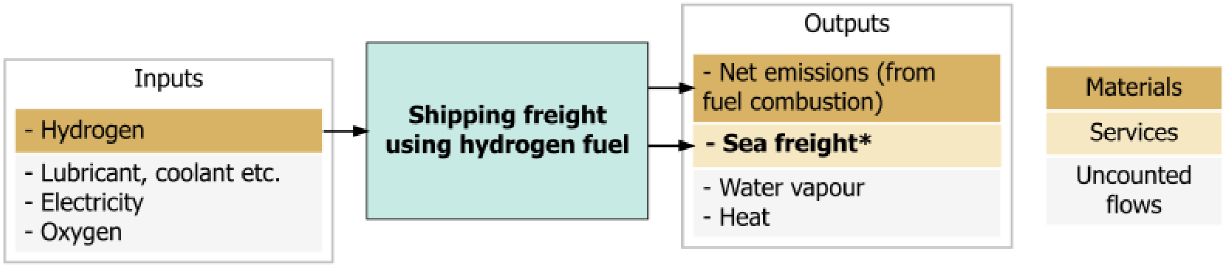

Figure S126: Inputs and outputs for shipping freight, powered by hydrogen.

Property	Quantity	Unit	Justification
**Data and assumptions**			
Fuel consumption	0.12	MJ/ tonne km	Refer to the introduction to this section for the service: Shipping
Hydrogen energy density	120	GJ/ t Hydogen	LHV - Giddey et al. (2013)
Compression energy	22	GJ elec/ t liquid fuel	Gardiner and Satypal (2009)
**Calculated coefficients (inputs)**			
Fuel demand	1.0	g fuel/ t_km	Fuel consumption / fuel energy density
Electricity to liquify hydrogen	22	GJ elec/ t_km	Gardiner and Satypal (2009)

Table S315: Key assumptions for estimating the coefficients for shipping by hydrogen fuel cell electric vessel (HFCEV). Note that the hydrogen is liquified which requires additional compression energy. See Table S307 for the justification of the fuel consumption value. References: Giddey et al. (2013)

Activity	Process	Resource	Inputs	Outputs	Unit
Shipping	HFCEV	Hydrogen	-0.0010		Gt/10^12tkm
Shipping	HFCEV	Electricity	-0.022		EJ/10^12tkm
Shipping	HFCEV	Shipping		1.0	10^12tkm/10^12tkm

Table S314: Chosen coefficients for transportation by hydrogen fuel cell electric vessel (HFCEV).

5.8.6.5 Ammonia-Fuelled

This delivery process is for ammonia powered long distance freight shipping. The fuel consumption value is based on the sources shown in Table S307.

The derived coefficients are shown in Table S316. These are derived based on the assumptions in Table S317.

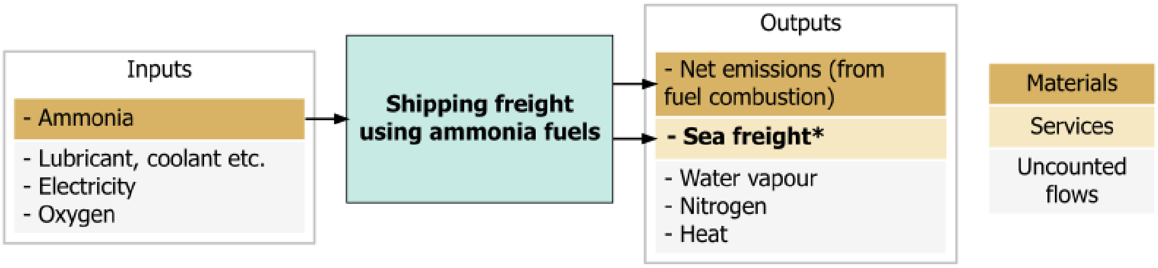

Figure S127: Inputs and outputs for shipping freight, powered by ammonia

Property	Quantity	Unit	Justification
**Data and assumptions**			
Fuel consumption	0.10	MJ/ tonne km	Refer to the introduction to this section for the service: Shipping
Ammonia energy density	18.6	EJ/Gt Ammonia	LHV - Giddey et al. (2013)
**Calculated coefficients (inputs)**			
Fuel demand	0.0056	Gt/ 10^12_t_km	Fuel consumption / fuel energy density

Table S317: Key assumptions for estimating the coefficients for shipping by ammonia fuel cell electric vessel (Ammonia). See Table S307 for the justification of the fuel consumption value. References: Giddey et al. (2013)

Activity	Process	Resource	Inputs	Outputs	Unit
Shipping	Ammonia	Ammonia	-0.0056		Gt/10^12tkm
Shipping	Ammonia	Shipping		1.0	10^12tkm/10^12tkm

Table S316: Chosen coefficients for transportation by ammonia fuel cell electric vessel.

5.8.6.6 Synfuel Powered Internal Combustion Engine

This delivery process is for synthetic-fuel powered freight shipping. The process and efficiency of an ICE (Internal Combustion Engine) vessel powered by synthetic fuel is assumed to be similar to a diesel vessel (Figure S124). The process to produce synthetic hydrocarbon fuels is accounted for in Section 5.6.11.

The derived coefficients are shown in Table S318. These are derived based on the assumptions in Table S319.

Property	Quantity	Unit	Justification
**Data and assumptions**			
Fuel consumption	0.10	MJ/ tonne km	Refer to the introduction to this section for the service: Shipping
Synthetic fuel Energy Density	43	GJ/t	Van Der Giesen et al. (2014)
CO2 input for Synfuel production	3.2	kg/ kg synfuel	From Synfuel production process by Fischer-Tropsch
**Calculated coefficients (inputs)**			
Synthetic fuel use	0.0023	Gt/ 10^12_t_km	Fuel consumption / fuel energy density
**Calculated coefficients (outputs)**			
Residual Emissions	0.0074	Gt CO2e/ 10^12_t_km	carbon dioxide to produce synfuel must be released during combustion. Residual emissions are thus: CO2 content of SynFuel x SynFuel consumption.

Table S319: Key assumptions for estimating the coefficients for synthetic-fuel powered shipping. See Table S307 for the justification of the fuel consumption value. References: Van Der Giesen et al. (2014)

Activity	Process	Resource	Inputs	Outputs	Unit
Shipping	SynICE	Synfuel	-0.0023		Gt/10^12tkm
Shipping	SynICE	NetEmissions		0.0074	Gt_CO2e/10^12tkm
Shipping	SynICE	Shipping		1.0	10^12tkm/10^12tkm

Table S318: Chosen coefficients for synthetic-fuel powered shipping.

5.8.7 Rail Freight Transportation

This activity describes freight transportation by rail. Each delivery process describes rail freight powered by a different drive-train technology and/or fuel (Table S320), and the coefficients are derived from assumptions of energy intensity. There is limited data available so energy intensities are estimated based on the current global energy intensity of rail freight and the current share of diesel and electrically powered rail freight. These values are shown in Table S321.

The energy intensity of rail freight varies significantly with load as well as drive-train technology. Gucwa & Schäfer (2013) demonstrated this correlation, plotting the energy intensity of existing freight rail for a range of countries; values were found to be mostly within the range 0.18 to 0.4 MJ/tkm. This is of a similar order to the values in Table S321. Gucwa & Schäfer (2013) do not include China in their analysis which may partly explain the difference compared to the values provided by International Energy Agency (2019a).

Activity	Delivery process	Detail
RailFreight	FFICE	Fossil Fuelled
RailFreight	Elec	Electrified
RailFreight	BioICE	Biofuel Powered Internal Combustion Engine
RailFreight	SynICE	Synfuel Powered Internal Combustion Engine

Table S320: Delivery processes for freight transportation by rail.

Rail category	Drive/fuel type	Note/ Source	Energy intensity (MJ/ tonne-km)
All rail freight	Current mix	2016 world average as estimated in IEA Future of Rail (2019). High efficiencies in China and Russia reduce the global intensity significantly from IEA member values.	0.13
**Chosen model values**	**Diesel**	**Currently 68% of global freight tonne-km (IEA, The Future of Rail, 2019)**	**0.14**
**Chosen model values**	**Electrically driven**	**Currently 32% of global freight tonne-km (IEA, The Future of Rail, 2019)**	**0.10**

Table S321: Summary of the data and chosen values for fuel consumption. Chosen values are estimated from current global energy intensity of rail freight and the current share of diesel and electrically powered rail freight. References: International Energy Agency (2019a)

5.8.7.1 Diesel-Fuelled (Conventional)

This delivery process is for conventional diesel-fuelled rail freight. The coefficients are derived based on the assumptions in Table S322. The fuel consumption estimate is estimated based on current global energy intensity of rail freight and the current share of diesel and electrically powered rail freight, as shown in Table S321. The derived coefficients are shown in Table S323.

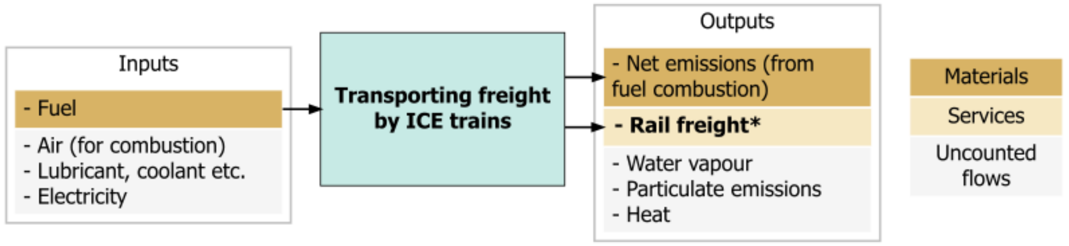

Figure S128: Inputs and outputs for transporting freight by rail, powered by internal combustion engines (ICEs).

Property	Quantity	Unit	Justification
**Data and assumptions**			
Fuel consumption	0.14	MJ/ tonne km	Refer to the introduction to this section for the service: Rail Freight
Diesel Energy Density	33	GJ/t	UK Government Conversion Factors
Diesel Emissions Factor	71	Mt CO2e/EJ	UK Government Conversion Factors (BEIS)
**Calculated coefficients (inputs)**			
Diesel fuel consumption	0.0042	Gt/ 10^12_t_km	Fuel consumption / fuel energy density
**Calculated coefficients (outputs)**			
Diesel residual emissions	0.010	Gt CO2e/ 10^12_t_km	Emissions factor x Fuel consumption

Table S322: Key assumptions for estimating the coefficients for conventional fossil-fuelled rail freight. References: Department for Business Energy & Industrial Strategy (2018)

Activity	Process	Resource	Inputs	Outputs	Unit
RailFreight	FFICE	Oil	-0.0042		Gt/10^12tkm
RailFreight	FFICE	NetEmissions		0.0099	Gt_CO2e/10^12tkm
RailFreight	FFICE	RailFreight		1.0	10^12tkm/10^12tkm

Table S323: Chosen coefficients for conventional fossil-fuelled rail freight.

5.8.7.2 Electrified

This delivery process is for Battery Electric Vehicle (Elec) rail freight. The electricity consumption estimate is justified in Table S321. The derived coefficients are shown in Table S324.

Activity	Process	Resource	Inputs	Outputs	Unit
RailFreight	Elec	Electricity	-0.10		EJ elec/10^12tkm
RailFreight	Elec	RailFreight		1.0	10^12tkm/10^12tkm

Table S324: Chosen coefficients for battery electric rail freight.

5.8.7.3 Biofuel Powered

This delivery process is for biofuel powered rail freight. The efficiency of a locomotive powered by biofuel is assumed to be similar to a diesel powered locomotive The process to produce biofuels is accounted for in Section 5.6.12.

The coefficients are derived based on the assumptions in Table S325. The fuel consumption estimate is estimated based on current global energy intensity of rail freight and the current share of diesel and electrically powered rail freight, as shown in Table S321. The derived coefficients are shown in Table S326.

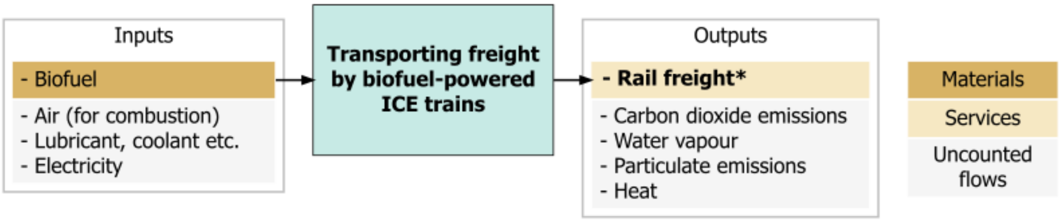

Figure S129: Inputs and outputs for transporting freight by rail, powered by biofuel-powered Internal Combustion Engine (ICE).

Property	Quantity	Unit	Justification
**Data and assumptions**			
Fuel consumption	0.14	MJ/ tonne km	Refer to the introduction to this section for the service: Rail Freight
Bio-fuel Energy Density	41	GJ/t	UK Government Conversion Factors (BEIS) give values from 27 (for bio-ethanol) to 44 (for Hydrogenated Veg Oil, HVO bio-diesel) GJ/t
**Calculated coefficients (inputs)**			
Bio-fuel use	0.0034	Gt/ 10^12_t_km	Fuel consumption / fuel energy density
**Calculated coefficients (outputs)**			
Residual Emissions	0	Gt CO2e/ 10^12_t_km	Emissions from the carbon in biomass (and production of biomass) are accounted for in the biomass production process. Emissions from biofuel production are accounted for in the biofuel production process.

Table S325: Key assumptions for estimating the coefficients for biofuel powered rail freight. References: Department for Business Energy & Industrial Strategy (2018).

Activity	Process	Resource	Inputs	Outputs	Unit
RailFreight	BioICE	BioFuel	-0.0034		Gt/10^12tkm
RailFreight	BioICE	RailFreight		1.0	10^12tkm/10^12tkm

Table S326: Chosen coefficients for biofuel powered rail freight.

5.8.7.4 Synfuel Powered Internal Combustion Engine

This delivery process is for synthetic-fuel powered rail freight (Figure S128). The efficiency of a locomotive powered by synthetic fuel is assumed to be similar to a diesel-fuelled locomotive. The process to produce synthetic hydrocarbon fuels is accounted for in Section 5.6.11.

The coefficients are derived based on the assumptions in Table S327. The fuel consumption estimate is estimated based on current global energy intensity of rail freight and the current share of diesel and electrically powered rail freight, as shown in Table S321. The derived coefficients are shown in Table S328.

Property	Quantity	Unit	Justification
**Data and assumptions**			
Fuel consumption	0.14	MJ/ tonne km	Refer to the introduction to this section for the service: Rail Freight
Synthetic fuel Energy Density	43	GJ/t	Van Der Giesen et al. (2014)
CO2 input for Synfuel production	3.2	kg/ kg synfuel	From Synfuel production process by Fischer-Tropsch
**Calculated coefficients (inputs)**			
Synthetic fuel use	0.0032	Gt/ 10^12_t_km	Fuel consumption / fuel energy density
**Calculated coefficients (outputs)**			
Residual Emissions	0.010	Gt CO2e/ 10^12_t_km	Carbon dioxide to produce synfuel must be released during combustion: Emissions = (CO2 content of SynFuel) x (SynFuel consumption)

Table S327: Key assumptions for estimating the coefficients for synthetic-fuel powered rail freight. References: Van Der Giesen et al. (2014)

Activity	Process	Resource	Inputs	Outputs	Unit
RailFreight	SynICE	Synfuel	**-0.0032**		Gt/10^12tkm
RailFreight	SynICE	NetEmissions		**0.010**	Gt_CO2e/10^12tkm
RailFreight	SynICE	RailFreight		**1.0**	10^12tkm/10^12tkm

Table S328: Chosen coefficients for synthetic-fuel powered rail freight.

5.8.8 Road Freight Transportation

This activity describes freight transportation by road. The fuel consumption varies widely depending on a wide range of factors but is significantly affected by the load size and vehicle type; light trucks may have an energy intensity around 15 MJ/tkm but large trucks are more likely closer to 1 MJ/tkm (Gucwa & Schäfer, 2013). To account for this, fuel consumption data for different size vehicles are combined to provide a scaled average, weighted by the share of road freight currently carried by that segment of the sector. The distribution of freight carried is based on 2015 values provided by International Energy Agency (2017) for three vehicle groupings: light commercial vehicles (LCVs), medium-freight trucks (MFTs), and heavy-freight trucks (HFTs) as shown in Table S329. Data for the fuel consumption of the alternative drive trains making up the delivery processes are taken from a different source which groups trucks by size into two, rather than three types; the smaller group is assumed equivalent to both LCVs and MFTs combined. The estimated fuel consumption values based on these data are given in Table S330.

Emissions are calculated from the fuel consumption using an emissions factor for each fuel (given in the SI Part 2).

Activity	Delivery process	Detail
RoadFreight	FFICE	Fossil Fuelled
RoadFreight	BEV	Battery Electric Vehicle
RoadFreight	CEV	Catenary Electric Vehicle
RoadFreight	BioICE	Biofuel Powered Internal Combustion Engine
RoadFreight	HFCEV	Hydrogen Fuel Cell Powered
RoadFreight	SynICE	Synfuel Powered Internal Combustion Engine

Table S331: Delivery processes for freight transportation by road.

Property	LCVs	MFTs	HFTs	Total or Average (weighted by tkm carried)	Justification
Assumed average load (tonne)	0.5	8	15		Central value in the load range for the category, as used in the data source
Approximate share of freight carried (share of tkm)	3%	33%	64%	**100%**	Approximated from data in the International Energy Agency report, The Future of Trucks (2017). Base year is 2015.
**Energy intensity, global stock efficiency (MJ/tkm)**	**7.0**	**1.5**	**1.4**	**1.6**	**Taken from Fig 18 in the International Energy Agency report, The Future of Trucks (2017). Base year is 2015.**

Acronyms
LCVs: light commercial vehicle; MFTs: medium-freight trucks; HFTs: heavy-freight trucks

Table S329: Summary of the approximations used to scale energy intensity of varying loaded trucks. References: International Energy Agency (2017)

	Scaled average fuel consumption (MJ/ tkm)				
Basis	Diesel	BEV	CEV	HFCEV	Justification for estimation
2015 estimate	1.6				Data from IEA (2017), The Future of Trucks, weighted by approximate shares of tkm
2016 estimate	1.5	0.77	0.65	1.1	Data from Röck, Martin and Hausberger (2020) scaled such that ”group 4” trucks contribute 34% of tkm (equivalent to LCVs and MFTs in IEA (2017) data)
2025 estimate	1.4	0.64	0.58	0.95	Scaled data from Röck, Martin and Hausberger (2020)
**Chosen values**	**1.4**	**0.64**	**0.58**	**0.95**	**Based on estimates above**

Table S330: Summary of the scaled data and chosen values for fuel consumption used in this analysis. The value in the first row is estimated using the approach in Table S329. Acronyms: BEV, Battery Electric Vehicle; CEV, Catenary Electric Vehicle; HFCEV, Hydrogen Fuel Cell Electric Vehicle. References: Röck et al. (2020), International Energy Agency (2017)

5.8.8.1 Fossil-Fuelled (Conventional)

This delivery process describes conventional fossil-fuelled road freight. The coefficients are derived based on the assumptions in Table S332. The fuel consumption is derived from average fuel consumption estimates for different sizes of vehicles, and the current distribution of freight carried by each of these vehicle classes, as shown in Table S330. The derived coefficients are shown in Table S333.

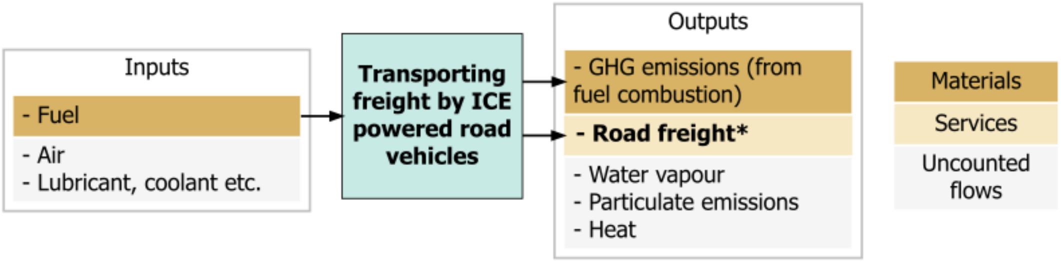

Figure S130: Inputs and outputs for transporting freight by internal combustion engine (ICE) large road vehicles.

Property	Quantity	Unit	Justification
**Data and assumptions**			
Fuel consumption	1.4	MJ/ t km = EJ/10^12tkm	Röck, Martin & Hausberger, (2020); IEA (2017) The Future of Trucks
Diesel Energy Density	33	GJ/t = EJ/Gt	UK Government Conversion Factors (BEIS)
Diesel Emissions Factor	71	Mt CO2e/EJ	UK Government Conversion Factors (BEIS)
**Calculated coefficients (inputs)**			
Diesel fuel consumption	0.042	Gt/ 10^12_t_km	Fuel consumption / fuel energy density
**Calculated coefficients (outputs)**			
Diesel residual emissions	0.097	Gt CO2e/ 10^12_t_km	Emissions factor x Fuel consumption

Table S332: Key assumptions for estimating the coefficients for conventional fossil-fuelled road freight. References: Röck et al. (2020); International Energy Agency (2017); Department for Business Energy & Industrial Strategy (2018)

Activity	Process	Resource	Inputs	Outputs	Unit
RoadFreight	FFICE	Oil	**-0.042**		Gt/10^12tkm
RoadFreight	FFICE	NetEmissions		**0.097**	Gt_CO2e/10^12tkm
RoadFreight	FFICE	RoadFreight		**1.0**	10^12tkm/10^12tkm

Table S333: Chosen coefficients for conventional fossil-fuelled road freight.

5.8.8.2 Battery Electric

This delivery process describes Battery Electric Vehicle (BEV) road freight (Figure S131). In this case the vehicle is powered by an electric motor and a battery, without the option for charging while driving; the process for Catenary Vehicles (Section 5.8.8.3) describes the alternative.

The electricity consumption is derived from average consumption estimates for different sizes of vehicles, and the current distribution of freight carried by each of these vehicle classes, as shown in Table S330. The derived coefficients are shown in Table S334.

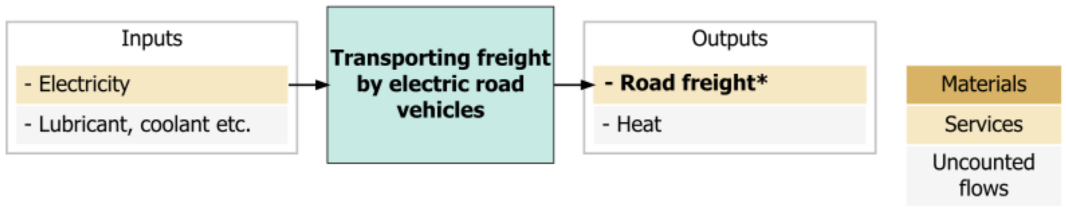

Figure S131: Inputs and outputs for transporting freight by large road vehicles powered by electric motors.

Activity	Process	Resource	Inputs	Outputs	Unit
RoadFreight	BEV	Electricity	-0.64		EJ elec/10^12tkm
RoadFreight	BEV	RoadFreight		1.0	10^12tkm/10^12tkm

Table S334: Chosen coefficients for battery electric road freight.

5.8.8.3 Catenary Electric

This delivery process is for Catenary Electric Vehicle (CEV) road freight(Figure S131). In this delivery process, road infrastructure provides additional charging for a Battery Electric Vehicle and so allows the battery size and weight to be reduced.

The electricity consumption is derived from average consumption estimates for different sizes of vehicles, and the current distribution of freight carried by each of these vehicle classes, as shown in Table S330. The derived coefficients are shown in Table S335.

Activity	Process	Resource	Inputs	Outputs	Unit
RoadFreight	CEV	Electricity	-0.58		EJ elec/10^12tkm
RoadFreight	CEV	RoadFreight		1.0	10^12tkm/10^12tkm

Table S335: Chosen coefficients for catenary electric road freight.

5.8.8.4 Biofuel Powered Internal Combustion Engine

This delivery process is for biofuel powered road freight (Figure S132. The efficiency of an ICE (Internal Combustion Engine) road freight powered by biofuel is assumed to be similar to diesel road freight. The process to produce biofuels is accounted for in Section 5.6.12.

The coefficients are derived based on the assumptions in Table S332. The fuel consumption is derived from average fuel consumption estimates for different sizes of vehicles, and the current distribution of freight carried by each of these vehicle classes, as shown in Table S330. The derived coefficients are shown in Table S336.

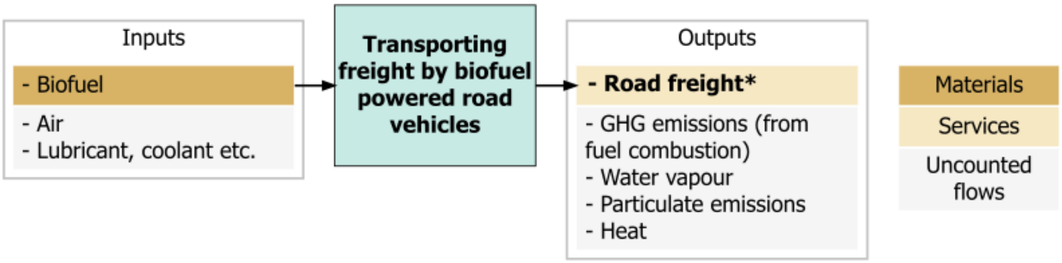

Figure S132: Inputs and outputs for transporting freight by biofuel powered large road vehicles.

Property	Quantity	Unit	Justification
**Data and assumptions**			
Fuel consumption	1	MJ/ t km	Röck, Martin & Hausberger, (2020); IEA (2017) The Future of Trucks
Bio-fuel Energy Density	13	GJ/t	UK Government Conversion Factors (BEIS) give values from 27 (for bio-ethanol) to 44 (for Hydrogenated Veg Oil, HVO bio-diesel) GJ/t
**Calculated coefficients (inputs)**			
Bio-fuel use	0.10	Gt/ 10^12_t_km	Fuel consumption / fuel energy density
**Calculated coefficients (outputs)**			
Residual Emissions	0	Gt CO2e/ 10^12_t_km	Emissions from the carbon in biomass (and production of biomass) are accounted for in the biomass production process. Emissions from biofuel production are accounted for in the biofuel production process.

Table S337: Key assumptions for estimating the coefficients for biofuel powered road freight. References: Röck et al. (2020); International Energy Agency (2017); Department for Business Energy & Industrial Strategy (2018).

Activity	Process	Resource	Inputs	Outputs	Unit
RoadFreight	BioICE	BioFuel	-0.10		Gt/10^12tkm
RoadFreight	BioICE	RoadFreight		1.0	10^12tkm/10^12tkm

Table S336: Chosen coefficients for biofuel powered road freight.

5.8.8.5 Hydrogen Fuel Cell Powered

This delivery process describes Hydrogen Fuel Cell Electric Vehicle (HFCEV) road freight (Figure S133). The coefficients are derived based on the assumptions in Table S338. The fuel consumption is derived from average fuel consumption estimates for different sizes of vehicles, and the current distribution of freight carried by each of these vehicle classes, as shown in Table S330. The derived coefficients are shown in Table S339.

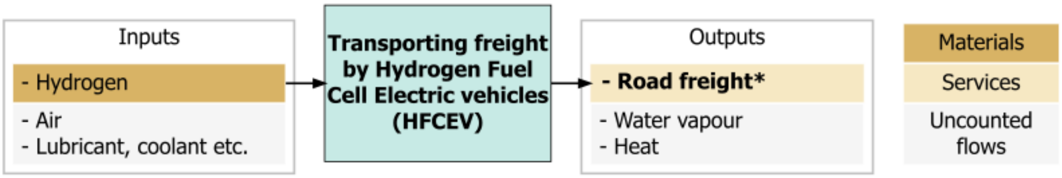

Figure S133: Inputs and outputs for transporting freight by HFCEVs.

Property	Quantity	Unit	Justification
**Data and assumptions**			
Fuel consumption	1	MJ/ t km	Röck, Martin & Hausberger, (2020); IEA (2017) The Future of Trucks
Hydrogen energy density	120	EJ/Gt Hydogen	LHV - Giddey et al. (2013)
**Calculated coefficients (inputs)**			
Fuel demand	0.0079	Gt/ 10^12_t_km	Fuel consumption / fuel energy density

Table S338: Key assumptions for estimating the coefficients for hydrogen powered road freight. References: Giddey et al. (2013); Röck et al. (2020); International Energy Agency (2017)

Activity	Process	Resource	Inputs	Outputs	Unit
RoadFreight	HFCEV	Hydrogen	-0.0079		Gt/10^12tkm
RoadFreight	HFCEV	RoadFreight		1.0	10^12tkm/10^12tkm

Table S339: Chosen coefficients for hydrogen fuel cell electric road freight.

5.8.8.6 Synfuel Powered Internal Combustion Engine

This delivery process is for synthetic-fuel powered road freight. The efficiency of an ICE (Internal Combustion Engine) road freight powered by synthetic fuel is assumed to be similar to diesel road freight (Figure S130). The process to produce synthetic hydrocarbon fuels is accounted for in Section 5.6.11.

The coefficients are derived based on the assumptions in Table S340. The fuel consumption is derived from average fuel consumption estimates for different sizes of vehicles, and the current distribution of freight carried by each of these vehicle classes, as shown in Table S330. The derived coefficients are shown in Table S341.

Property	Quantity	Unit	Justification
**Data and assumptions**			
Fuel consumption	1	MJ/ t km	Röck, Martin & Hausberger, (2020); IEA (2017) The Future of Trucks
Synthetic fuel Energy Density	43	GJ/t	Van Der Giesen et al. (2014)
CO2 input for Synfuel production	3.2	kg/ kg synfuel	From Synfuel production process by Fischer-Tropsch
**Calculated coefficients (inputs)**			
Synthetic fuel use	0.032	Gt/ 10^12_t_km	Fuel consumption / fuel energy density
**Calculated coefficients (outputs)**			
Residual Emissions	0.10	Gt CO2e/ 10^12_t_km	carbon dioxide to produce synfuel must be released during combustion. Residual emissions are thus: CO2 content of SynFuel x SynFuel consumption.

Table S340: Key assumptions for estimating the coefficients for synthetic-fuel powered road freight. References: Van Der Giesen et al. (2014); Röck et al. (2020); International Energy Agency (2017)

Activity	Process	Resource	Inputs	Outputs	Unit
RoadFreight	SynICE	Synfuel	**-0.032**		Gt/10^12tkm
RoadFreight	SynICE	NetEmissions		**0.10**	Gt_CO2e/10^12tkm
RoadFreight	SynICE	RoadFreight		**1.0**	10^12tkm/10^12tkm

Table S341: Chosen coefficients for synthetic-fuel powered road freight.

5.8.9 Transport Coefficient Summary

ResourceFlow	Aviation_JetA	Aviation_JetAT	Aviation_LH2	Aviation_LH2T	Aviation_LNG	Aviation_LNGT	Aviation_ElecT	Aviation_PtL	Aviation_PtLT	Aviation_Bio	Aviation_BioT	BusUse_FFICE	BusUse_BEV	BusUse_HFCEV	BusUse_BioICE	BusUse_SynICE
Oil, Gt	-0.025	-0.025									-0.025	-0.014				
NetEmissions, Gt_CO2e	0.080	0.14		0.076	0.062	0.14		0.078	0.13		0.13	0.042				0.044
Aviation, 10^12pkm	1.0	1.0	1.0	1.0	1.0	1.0	1.0	1.0	1.0	1.0	1.0					
Hydrogen, Gt			-0.0093	-0.0093										-0.0046		
Electricity, EJ			-0.20	-0.20			-0.80									
Methane, Gt					-0.027	-0.027										
Synfuel, Gt								-0.025	-0.025							-0.014
BioFuel, Gt										-0.025					-0.017	
BusUse, 10^12pkm												1.0	1.0	1.0	1.0	1.0
Electricity, EJ elec													-0.20			
CarUse, 10^12_car_km																
RailFreight, 10^12tkm																
RailP, 10^12pkm																
RoadFreight, 10^12tkm																
Shipping, 10^12tkm																
Ammonia, Gt																

Table S342: Coefficients for transportation services. Part 1 of 3

ResourceFlow	CarUse__FFICE	CarUse__BEV	CarUse__HFCEV	CarUse__BioICE	CarUse__SynICE	RailFreight__FFICE	RailFreight__Elec	RailFreight__BioICE	RailFreight__SynICE	RailP__FFICE	RailP__Elec	RailP__HFCEV	RailP__BioICE	RailP__SynICE
Oil, Gt	-0.044					-0.0042				-0.0060				
NetEmissions, Gt__CO2e	0.15				0.15	0.0099			0.010	0.014				0.015
Aviation, 10^12pkm														
Hydrogen, Gt			-0.0075									-0.00092		
Electricity, EJ														
Methane, Gt														
Synfuel, Gt					-0.046				-0.0032					-0.0046
BioFuel, Gt				-0.056				-0.0034					-0.0049	
BusUse, 10^12pkm														
Electricity, EJ elec		-0.55					-0.10				-0.15			
CarUse, 10^12__car__km	1.0	1.0	1.0	1.0	1.0									
RailFreight, 10^12tkm						1.0	1.0	1.0	1.0					
RailP, 10^12pkm										1.0	1.0	1.0	1.0	1.0
RoadFreight, 10^12tkm														
Shipping, 10^12tkm														
Ammonia, Gt														

Table S343: Coefficients for transportation services. Part 2 of 3

ResourceFlow	RoadFreight__FFICE	RoadFreight__BEV	RoadFreight__CEV	RoadFreight__HFCEV	RoadFreight__BioICE	RoadFreight__SynICE	Shipping__FFICE	Shipping__LNG	Shipping__Ammonia	Shipping__BioICE	Shipping__SynICE
Oil, Gt	-0.042						-0.0024				
NetEmissions, Gt__CO2e	0.097					0.10	0.0076	0.0057			0.0074
Aviation, 10^12pkm											
Hydrogen, Gt				-0.0079							
Electricity, EJ											
Methane, Gt								-0.0022			
Synfuel, Gt						-0.032					-0.0023
BioFuel, Gt					-0.10					-0.0024	
BusUse, 10^12pkm											
Electricity, EJ elec		-0.64	-0.58								
CarUse, 10^12__car__km											
RailFreight, 10^12tkm											
RailP, 10^12pkm											
RoadFreight, 10^12tkm	1.0	1.0	1.0	1.0	1.0	1.0					
Shipping, 10^12tkm							1.0	1.0	1.0	1.0	1.0
Ammonia, Gt									-0.0056		

Table S344: Coefficients for transportation services. Part 3 of 3

5.9 Energy-use in Buildings

This section outlines the derivation of coefficients for building-use, i.e. the quantities of resources needed to provide activities in the built environment such as the provision of heat, light, and the use of appliances.

All energy use and emissions associated with the using the built environment is assumed to arise in provision of:

- Space heating;
- Water heating;
- Cooking;
- Space cooling;
- Lighting; and
- Appliance-use (IT equipment, fridges, vacuum cleaners, hair dryers, washing machines etc).

Although these activities represent the ISIC codes in Table S345, the ISIC codes are not sufficient to describe the activities in this sector. For example, the activities in this sector include consumption by households, excluding fuels used for transport, whereas ISIC codes only represent households with employed persons, which makes up only a small proportion of total energy-use.

Charging vehicles is not included within building operational activities but within car-use (Section 5.8). Waste is also considered in a separate category (Section 5.10). Building-use emissions are assumed to be from fuel combustion only.

Sector	Activity	Model Name	ISIC Code	End-Use
Buildings	Cooking	Cooking		✓
Buildings	Cooling the Built Environment	Cooling		✓
Buildings	Lighting	Lighting		✓
Buildings	Space Heating	SpaceHeat		✓
Buildings	Use of Appliances	Appliances		✓
Buildings	Water Heating	WaterHeating		✓

Table S345: Model flows categorised by ISIC divisions, groups and classes for this sector. Note that the ISIC codes listed here are not sufficient to decribe the activities in the sector, Building-Use. The activities in this sector include consumption by households, excluding fuels used for transport, whereas ISIC codes only represent households with employed persons, making up a small proportion of total energy-use. For activities labelled as 'End-Use', the activity rate is set by the model inputs, rather than the demands of other activities.

5.9.1 Sector data sources

The coefficients for energy use in buildings have been derived from 2018 global energy use data from the IEA's world energy balances (2021a). Coefficients for each delivery process are derived by considering its relative efficiency compared to the conventional processes in 2018.

Using top-down global averages makes it feasible to represent this inhomogeneous sector with only a small set of processes. In reality, energy-use (and related emissions) within buidings covers a wide range of activities which each have large variation around the world (varying, for example with local climate, culture and affluence). There are also significant differences between residential, commercial and public buildings, and even within those groups, the structure and form of buildings vary regionally.

5.9.1.1 Energy consumption

The quantity of energy used in buildings is based on the *Residential* and *Commercial & Public Activities* categories within the IEA's world energy balances (2021a).

- *Residential* includes "consumption by households, excluding fuels used for transport…" (ISIC Rev. 4 Divisions 97 and 98);
- *Commercial & public activities* includes [ISIC Rev. 4 Divisions 33, 36-39, 45-47, 52, 53, 55-56, 58-66, 68-75, 77-82, 84 (excluding Class 8422), 85-88, 90-96 and 99] (International Energy Agency - IEA, 2020). These activities include warehousing, education and veterinary activities. This category also includes water and waste treatment activities. To avoid double counting, the delivery processes for waste (Section 5.10) do not include the energy demands but only consider process emissions.

IEA energy data for does not disaggregate between the energy used for industrial building services (such as lighting) and industrial processes; industrial building energy use is therefore not accounted for here. It is assumed here that the energy used for providing building services in industry is small compared to to commercial and domestic building services, and compared to industrial processes. The first of these appears reasonable (UK energy consumption data shows that heating and lighting for industrial buildings demanded only 6% of all building services energy consumption in 2018) but the second may be a rough approximation in some regions (16% of energy use in industrial buildings was for building services in 2018); these estimates are based on UK Government datasets, as shown in Table S346 (BEIS, 2021a).

The distribution of energy amongst different building activities is based on data used by Ürge-Vorsatz et al. (2015) as shown in Table S347.

The distribution of Ürge-Vorsatz et al. (2015) is used as the main basis of the overall distribution, since it is globally relevant. The energy distribution for the commercial sector does not, however, distinguish between appliances and cooking. UK energy consumption data (from BEIS (2021a), in Table S348) is used to approximate this, and to provide a cross-reference for the overall distribution in Table S347.

The UK distribution in Table S348 shows a greater proportion of energy used for heating (expected for a relatively cool country) but generally follows a similar pattern to the global data in Table S347. For the commercial sector, the UK shows an approximately equal quantity of energy used for commercial appliances and commercial cooking[8]. Based on this, the Commercial "other category" in the global distribution (Table S347) is split equally into appliance-use and cooking.

The final distribution of energy amongst activities is shown in the *share* column in Table S349.

	Building Type			
Building service	Residential	Industrial	Commercial	Total
**Share of energy use for all UK building activities**				
Space heating	41%	3%	16%	61%
Water	12%	0%	2%	14%
Cooking/ Catering	2%	0%	4%	6%
Lighting/ Appliances	11%	3%	6%	20%
**Building type total**	**66%**	**6%**	**28%**	**100%**
**Absolute consumption of energy**				
**Total sectoral building services, EJ**	**2.1**	**0.1**	**1.1**	**3.1**

Table S346: Energy use for building activities in the UK in 2018, split into residential, industrial and commercial buildings. (BEIS, 2021a)

[8]Noting that the "lighting/appliances" category in Table S348 includes both lighting and appliances, where lighting was around one third of lighting, appliances and cooking in Table S347.

Building service	Residential	Commercial	Total
Space heating	63%	57%	61%
Water	18%	8%	14%
Cooking/ Catering	3%	13%	6%
Lighting/ Appliances	17%	22%	20%
**Total**	**100%**	**100%**	**100%**

Table S348: The distribution of energy use within each sector of (non industrial) buildings in the UK in 2018(BEIS, 2021a)

Building Service	Assumed share of energy use	Attributed 2018 demand, EJ
**Residential**		
Space heating	32%	28
Water heating	24%	21
Lighting	4%	4
Cooling	2%	2
Appliances	9%	8
Cooking	29%	25
**TOTAL**	**100%**	**88**
**Commercial**		
Space heating	33%	11
Water heating	12%	4
Lighting	16%	5
Cooling	7%	2
Other, including IT equipment etc	32%	11
**TOTAL**	**100%**	**33**

Table S347: Assumed proportions of energy use across activities in buildings as used by Ürge-Vorsatz et al. (2015). The absolute quantity of energy for each activity is quantified for 2018 using these proportions and totals from International Energy Agency (2021a). For deriving the delivery process coefficients in this section, the Commercial: Other category here is split equally into appliance-use and cooking, based on UK distributions (Table S348).

5.9.1.2 Baseline delivery process shares

The resource demands for building activities are estimated from the relative efficiency of each delivery process, compared to the current globally averaged efficiency of that activity. The current activity efficiency is derived by estimating the distribution of fuel usage - and so related delivery processes - for each activity for 2018, and their respective efficiencies. The resource demands for each delivery process are found by estimating the approximate input energy to provide the same quantity of useful energy as in 2018.

The fuel split in 2018 is estimated by allocating the 2018 fuels consumed in buildings (from IEA world energy balances (2021a)) amongst the building activities to achieve the baseline energy use for each activity in Table S350. The share of each fuel is assigned using a judgement of the types and distributions of technologies used for delivering each activity. The values used are in Table S349.

Service	Coal & coal products	Oil products	Natural gas	Geothermal	Solar/ wind/ other	Biofuels & waste	Electricity	Heat	Total, EJ	Share
**Residential**										
Space heating	1.1	3.4	7.6	0.3	0.4	10	2.6	2.3	**28**	**32%**
Water heating	0.8	2.6	5.7	0.3	0.3	7.8	1.3	2.3	**21**	**24%**
Lighting							3.5		**3.5**	**4%**
Cooling							1.8		**1.8**	**2%**
Appliances							7.9		**7.9**	**9%**
Cooking	1.0	3.1	6.9		0.4	9.4	4.6		**25**	**29%**
**Residential Total**	**2.9**	**9.1**	**20**	**0.6**	**1.1**	**28**	**22**	**4.6**	**88**	**100%**
**Commercial**										
Space heating	0.8	2.4	3.4	0.1		0.8	2.3	1.2	**11**	**33%**
Water heating	0.3	0.9	1.2			0.3		0.4	**4.0**	**12%**
Lighting					0.1		5.3		**5.4**	**16%**
Cooling							2.3		**2.3**	**7%**
Appliances							5.3		**5.4**	**16%**
Cooking			4.0				1.3		**5.3**	**16%**
**Commercial Total**	**1.1**	**3.3**	**8.6**	**0.2**	**0.2**	**1.1**	**17**	**1.7**	**33**	**100%**
**All Building Types**										
**TOTAL**	**4.0**	**12**	**29**	**0.8**	**1.3**	**29**	**39**	**6.3**	**121**	

Table S349: The assumed distribution of fuels consumed (in EJ) for provision of each activity. The total fuel consumption is from IEA world energy balance data (2021a) and share of energy consumption for each activity from Table S350.

5.9.2 2018 Activity Rates

Building end-user activities are difficult to quantify and measure; true end-user activities may include thermal comfort, sanitation, and entertainment, each of which is a subjective measure. For simplicity, energy-use in buildings is used as a proxy for the activity measure. The 2018 use for each building activity is used as a "comfort baseline". This is a reasonable approach since residential energy use is not the focus of the study but a contribution to the overall aggregated demands. The approach is likely to be conservative since heating and cooling degree days will increase as climate change occurs, and given the growing affluence and high levels of population density in highly affected regions of the world (Kennard et al., 2022). On the other hand, technology developments may counterbalance this to some extent.

The baseline quantities were derived using the distribution described in Section 5.9.1 and the total energy consumption in 2021 from IEA World Energy balances (International Energy Agency, 2021a). Values are given in Table S350.

Service	2018 end-use total	Unit
Lighting	8.9	EJ_Lighting
Appliances	13	EJ_Appliances
Water heating	25	EJ_WaterHeating
Space Heating	39	EJ_SpaceHeat
Cooling	4.1	EJ_Cooling
Cooking	31	EJ_Cooking
**TOTAL (check)**	**121**	**EJ**

Table S350: Baseline quantities for building activities. Data sources: (BEIS, 2021a) ; (Ürge-Vorsatz et al., 2015); (International Energy Agency, 2021a)

5.9.3 Lighting

This activity describes the provision of illuminated spaces. In the absence of a measurable metric for this, energy use for lighting in 2018 is used as a proxy (see page S298). Only one delivery process is considered for lighting, which is electrically powered.

5.9.3.1 Electrically-powered

This process describes providing lighting using electric power. The coefficients assume that the amount of lighting powered by non-electric power today is negligible. Technology efficiency improvements are not considered here, giving the resulting coefficients in Table S351.

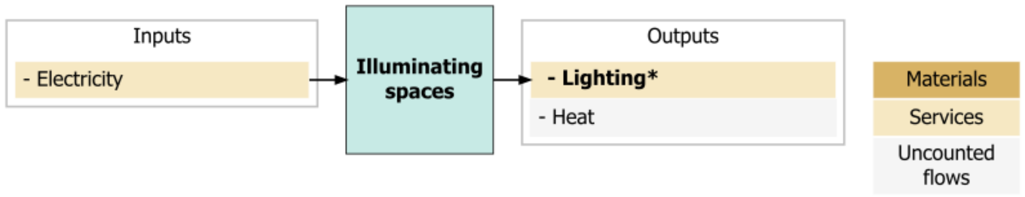

Figure S134: Inputs and outputs for Lighting production'

Activity	Process	Resource	Inputs	Outputs	Unit
Lighting	Elec	Electricity	-1.0		EJ/EJ_Lighting
Lighting	Elec	Lighting		1.0	EJ_Lighting/EJ_Lighting

Table S351: Coefficients for lighting by electric power

5.9.4 Appliance Use

This activity describes powering appliances. In the absence of a measurable metric for this, energy use for appliances in 2018 is used as a proxy (see page S298). Only one delivery process is considered for appliance use, which is electrically powered. Charging electric vehicles is not included in this activity, but in car-use (Section 5.8).

5.9.4.1 Electrically-powered

This process describes powering appliances with electricity. Appliances are predominantly powered by electricity today. The coefficients assume that the amount of energy used to power appliances by non-electric power today is negligible, and do not account for technology efficiency improvements, giving the resulting coefficients in Table S352.

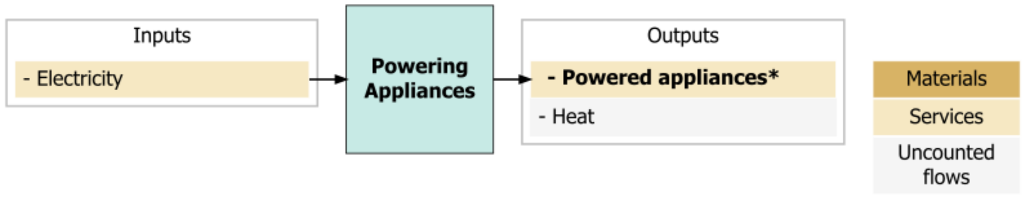

Figure S135: Inputs and outputs for steel production

Activity	Process	Resource	Inputs	Outputs	Unit
Appliances	Elec	Electricity	-1.0		EJ/EJ_Appliances
Appliances	Elec	Appliances		1.0	EJ_Appliances/EJ_Appliances

Table S352: Coefficients for powering appliances by electric power

5.9.5 Space Cooling

This activity describes cooling spaces (air-conditioning). Only electrically powered space cooling is currently included in the model. Other processes for space cooling could be added to the model in future, as described in SI Part 2.3.

5.9.5.1 Electrically powered space cooling

This process describes providing cooled spaces using electrically-powered air conditioning units, as are used currently. As such, there is no change in the efficiency of conversion from electricity to final energy used for cooling, giving the coefficients in Table S353. Leakage of GHG refrigerants are not accounted for.

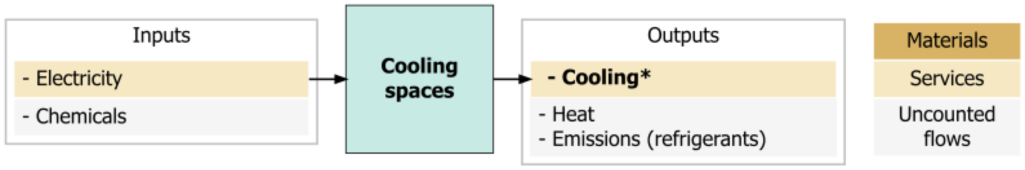

Figure S136: Inputs and outputs for space cooling.

Activity	Process	Resource	Inputs	Outputs	Unit
Cooling	Elec	Cooling		1.0	EJ_Cooling/EJ_Cooling
Cooling	Elec	Electricity	-1.0		EJ/EJ_Cooling

Table S353: Coefficients for cooling with electrically powered air-conditioning units.

5.9.6 Water Heating

This activity describes provision of hot water for washing and cleaning; hot water for cooking and drinking is accounted for in Cooking (Section 5.9.8). The coefficients for water heating delivery processes are estimated based on the relative efficiency values of each process, compared to the average for 2018, as shown in Table S354. Emissions from combustion are estimated using the emissions factors in the SI Part 2.

Activity	Process	Process Long Name
WaterHeating	Gas	Gas boiler
WaterHeating	Elec	Direct Electrical
WaterHeating	Bio	Biomass Fuelled
WaterHeating	H2Boiler	Hydrogen Boiler
WaterHeating	HeatPump	Heat Pump (20% Ground; 80% Air Source)

Table S355: Delivery processes for this activity. The coefficients for each delivery process are given and explained in the remainder of this section.

Water heating delivery process	2018 final energy use, EJ	2018 consumption share	Process efficiency
Biomass and waste	8	32%	75%
Coal, gas or liquid fuel	11	46%	90%
Direct electrical	2	6%	95%
Heat pumps	0	4%	270%
District heating	3	12%	90%
Hydrogen boilers	0	0%	80%
**All water heating in 2018**	**25**	**100%**	**92%**

Table S354: The assumed process efficiency of each delivery process for conventional water heating is estimated based on the assumptions used by Paoli et al. (2018), Element Energy Ltd (2021), Lund et al. (2010), and Element Energy Ltd (2015). The share of consumption in 2018 is based on the distribution of final energy fuel use given in Table S349, where 10% of heating needs (based on the share of consumption and efficiency) is assumed to be from heat pumps (IEA, 2022b). All heat energy is assumed to provide district heating. The share of consumption for each process is used to find a scaled average process efficiency for water heating in 2018.

5.9.6.1 Gas fuelled water heating

This process describes producing hot water using gas boilers. The demand for methane (natural gas) is estimated from the efficiency values in Table S354 and energy density values in the SI Part 2. This is combined with the emissions factor for methane (as given in the SI Part 2) to estimate the combustion emissions for water heating with natural gas. The derived values are given in Table S356.

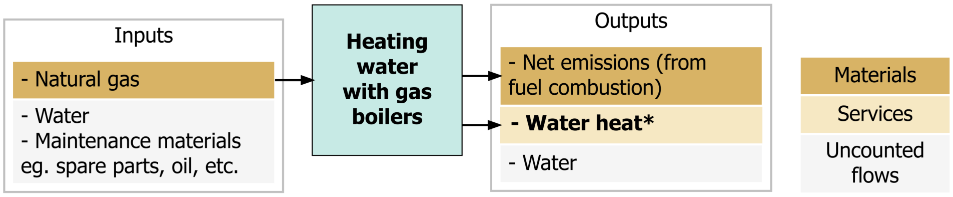

Figure S137: Inputs and outputs for heating water with gas boilers.

Activity	Process	Resource	Inputs	Outputs	Unit
WaterHeating	Gas	Methane	-0.023		Gt/EJ_WaterHeat
WaterHeating	Gas	NetEmissions		0.061	Gt_CO2e/EJ_WaterHeat
WaterHeating	Gas	WaterHeating		1.0	EJ_WaterHeat/EJ_WaterHeat

Table S356: Coefficients for gas powered water heating.

5.9.6.2 Direct electrical water heating

This process describes producing hot water using electric boilers (direct electrical water heating); this includes electric resistive heating and storage heating. The coefficients, derived from the efficiency values in Table S354 are given in Table S357.

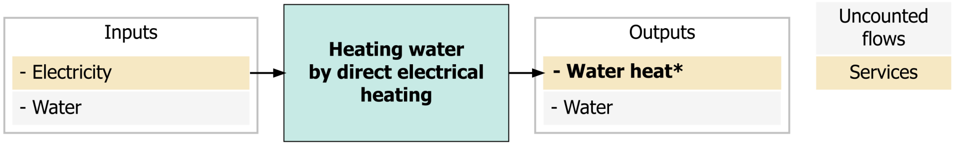

Figure S138: Inputs and outputs for direct electrical water heating.

Activity	Process	Resource	Inputs	Outputs	Unit
WaterHeating	Elec	Electricity	-0.97		EJ/EJ_WaterHeat
WaterHeating	Elec	WaterHeating		1.0	EJ_WaterHeat/EJ_WaterHeat

Table S357: Coefficients for electrical water heating.

5.9.6.3 Biomass-fuelled water heating

This process describes producing hot water using biomass boilers. The demand for biomass is estimated from the efficiency values in Table S354 and energy density values in the SI Part 2. This is combined with the emissions factor for biomass (as given in the SI Part 2) to estimate the combustion emissions for water heating with biomass. The resulting values are given in Table S358.

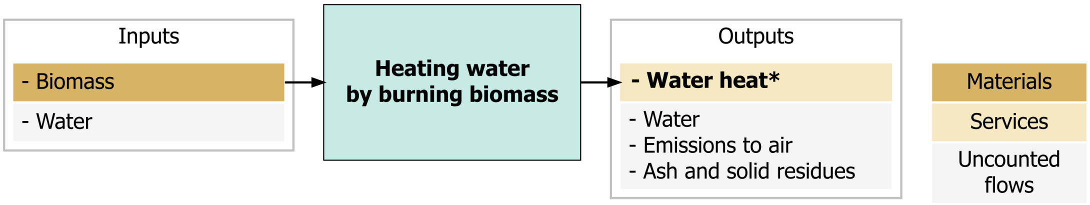

Figure S139: Inputs and outputs for heating water by burning biomass.

Activity	Process	Resource	Inputs	Outputs	Unit
WaterHeating	Bio	NonWoodBiomass	-0.041		Gt_DryBio/EJ_WaterHeat
WaterHeating	Bio	Wood	-0.041		Gt_DryBio/EJ_WaterHeat
WaterHeating	Bio	WaterHeating		1.0	EJ_WaterHeat/EJ_WaterHeat

Table S358: Coefficients for biomass-fuelled water heating.

5.9.6.4 Hydrogen fuelled water heating

This process describes producing hot water using hydrogen-fuelled boilers. The demand for hydrogen is estimated from the efficiency values in Table S354 and energy density values in the SI Part 2. This is combined with the emissions factor for hydrogen (as given in the SI Part 2) to estimate the combustion emissions. The resulting values are given in Table S359.

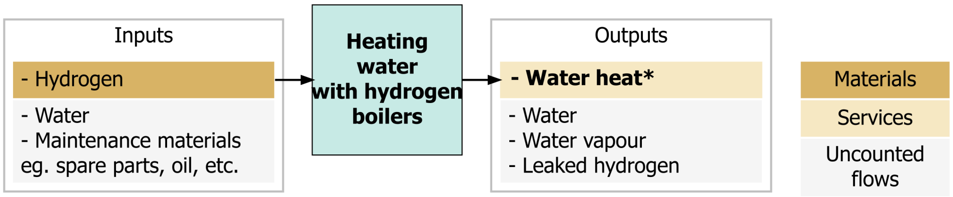

Figure S140: Inputs and outputs for heating water with hydrogen boilers.

Activity	Process	Resource	Inputs	Outputs	Unit
WaterHeating	H2Boiler	Hydrogen	-0.0096		Gt/EJ_WaterHeat
WaterHeating	H2Boiler	WaterHeating		1.0	EJ_WaterHeat/EJ_WaterHeat

Table S359: Coefficients for water heating using hydrogen boilers.

5.9.6.5 Water heating using heat pumps

This process describes producing hot water using heat pumps. The process assumes that the heat source is available without any additional resource demands; the only demand is for the electricity to drive the heat pump. This is derived from an estimate of the Seasonal coefficient of Performance (SPF) where,

$$SPF = \frac{Heat\ energy\ output}{Electricity\ input}$$

Both ground source and air source heat pumps (GSHP and ASHP respectively) are included within this process. In the absence of data, it is assumed that 20% of the installations would be GSHP while the remainder are ASHP; consistent with the ratio of the two technologies considered in the modelling by Element Energy Ltd (2021). The estimated value is given in Table S360 and the resulting coefficients in Table S361.

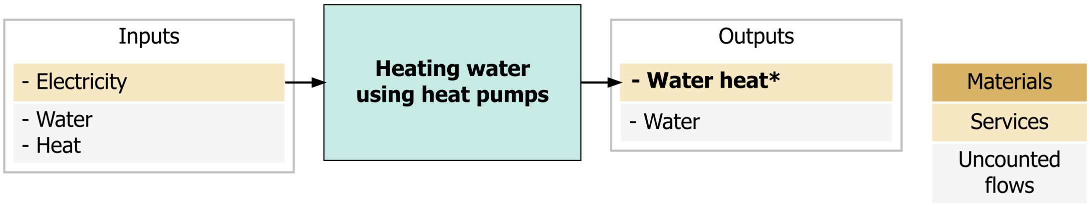

Figure S141: Inputs and outputs for heating water with heat pumps.

Property	Value	Unit	Justification
ASHP SPF (water)	2.6	EJ_WaterHeat/EJ_Elec	Mid range estimate from (element energy, 2021)
GSHP SPF (water)	3.0	EJ_WaterHeat/EJ_Elec	Mid range estimate from (element energy, 2021)
Share which are ground source	20%		Approx ratio in (element energy, 2021)
**Average SPF**	**2.7**	**EJ_WaterHeat/EJ_Elec**	**Scaled average**

Note:
ASHP: Air-Source Heat Pump; GSHP: Ground-Source Heat Pump; SPF: Seasonal Coefficient of Performance

Table S360: Seasonal coefficient of Performance (SPF) assumptions used to derive the coefficients for water heating using heat pumps. Data sources: (Element Energy Ltd, 2021)

Activity	Process	Resource	Inputs	Outputs	Unit
WaterHeating	HeatPump	Electricity	-0.37		EJ/EJ_WaterHeat
WaterHeating	HeatPump	WaterHeating		1.0	EJ_WaterHeat/EJ_WaterHeat

Table S361: Coefficients for water heating using heat pumps.

5.9.7 Space Heating

This activity describes the provision of heat to maintain comfortable temperatures in buildings. The coefficients for space heating delivery processes are estimated based on the relative efficiency values of each process, compared to the average for 2018, as shown in Table S362. Emissions from combustion are estimated using the emissions factors in the SI Part 2.

Activity	Process	Process Long Name
SpaceHeat	Gas	Gas boiler
SpaceHeat	Elec	Direct Electrical
SpaceHeat	Bio	Biomass Fuelled
SpaceHeat	H2Boiler	Hydrogen Boiler
SpaceHeat	HeatPump	Heat Pump (20% Ground; 80% Air Source)

Table S363: Delivery processes for this activity. The coefficients for each delivery process are given and explained in the remainder of this section.

Space heating delivery process	2018 final energy use, EJ	2018 consumption share	Process efficiency
Biomass and waste	11	29%	75%
Coal, gas or liquid fuel	19	48%	90%
Direct electrical	5	11%	97%
Heat pumps	0	3%	350%
District heating	4	10%	90%
Hydrogen boilers	0	0%	80%
**All space heating in 2018**	**39**	**100%**	**93%**

Table S362: The assumed process efficiency of each delivery process for conventional space heating is estimated based on the assumptions used by Paoli et al. (2018), Element Energy Ltd (2021), Lund et al. (2010), and Element Energy Ltd (2015). The share of consumption in 2018 is based on the distribution of final energy fuel use given in Table S349, where 10% of heating needs (based on the share of consumption and efficiency) is assumed to be from heat pumps (IEA, 2022b). All heat energy is assumed to provide district heating. The share of consumption for each process is used to find a scaled average process efficiency for space heating in 2018.

5.9.7.1 Gas fuelled space heating

This process describes heating space using gas boilers. The demand for methane (natural gas) is estimated from the efficiency values in Table S362 and energy density values in the SI Part 2. This is combined with the emissions factor for methane (as given in the SI Part 2) to estimate the combustion emissions for space heating with natural gas. The chosen values are given in Table S364.

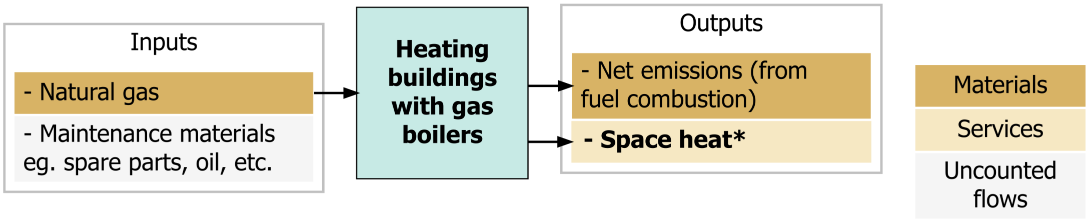

Figure S142: Inputs and outputs for space heating with gas boilers.

Activity	Process	Resource	Inputs	Outputs	Unit
SpaceHeat	Gas	Methane	-0.023		Gt/EJ_SpaceHeat
SpaceHeat	Gas	NetEmissions		0.062	Gt_CO2e/EJ_SpaceHeat
SpaceHeat	Gas	SpaceHeat		1.0	EJ_SpaceHeat/EJ_SpaceHeat

Table S364: Coefficients for gas powered space heating.

5.9.7.2 Direct electrical space heating

This process describes direct electrical space heating, including electric resistive heating and storage heating. The coefficients, derived from the efficiency values in Table S362 are given in Table S365.

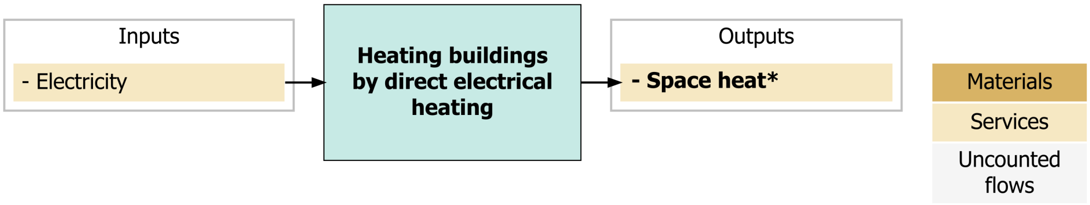

Figure S143: Inputs and outputs for space heating by direct electrical heating.

Activity	Process	Resource	Inputs	Outputs	Unit
SpaceHeat	Elec	Electricity	-0.97		EJ/EJ_SpaceHeat
SpaceHeat	Elec	SpaceHeat		1.0	EJ_SpaceHeat/EJ_SpaceHeat

Table S365: Coefficients for electrical space heating.

5.9.7.3 Biomass-fuelled space heating

This process describes producing heat using biomass boilers. The demand for biomass is estimated from the efficiency values in Table S362 and energy density values in the SI Part 2. This is combined with the emissions factor for biomass (as given in the SI Part 2) to estimate the combustion emissions for space heating with biomass. The resulting values are given in Table S366.

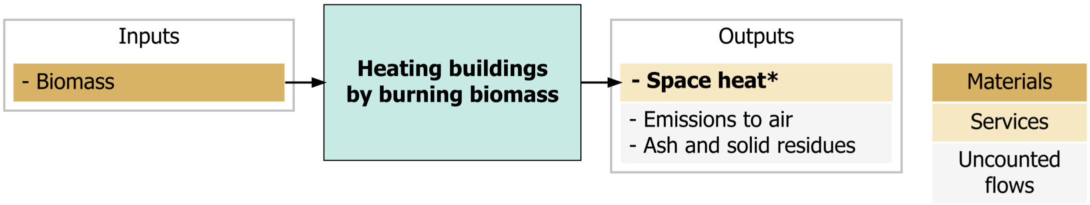

Figure S144: Inputs and outputs for space heating by burning biomass.

Activity	Process	Resource	Inputs	Outputs	Unit
SpaceHeat	Bio	NonWoodBiomass	-0.041		Gt_DryBio/EJ_SpaceHeat
SpaceHeat	Bio	Wood	-0.041		Gt_DryBio/EJ_SpaceHeat
SpaceHeat	Bio	SpaceHeat		1.0	EJ_SpaceHeat/EJ_SpaceHeat

Table S366: Coefficients for biomass-fuelled space heating.

5.9.7.4 Hydrogen fuelled space heating

This delivery process describes space heating using hydrogen-fuelled boilers. The demand for hydrogen is estimated from the efficiency values in Table S362 and energy density values in the SI Part 2. This is combined with the emissions factor for hydrogen (as given in the SI Part 2) to estimate the combustion emissions. The resulting values are given in Table S367.

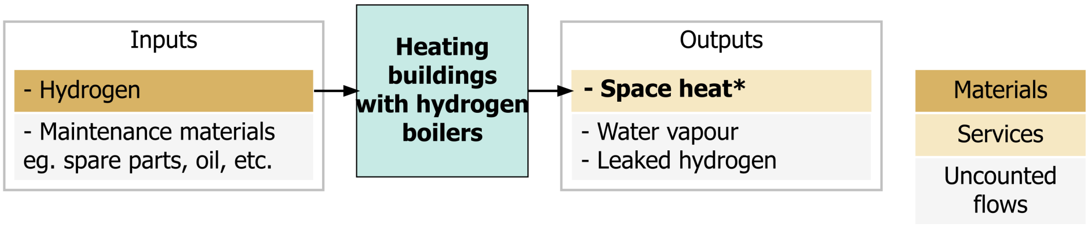

Figure S145: Inputs and outputs for space heating with hydrogen boilers.

Activity	Process	Resource	Inputs	Outputs	Unit
SpaceHeat	H2Boiler	Hydrogen	-0.0096		Gt/EJ_SpaceHeat
SpaceHeat	H2Boiler	SpaceHeat		1.0	EJ_SpaceHeat/EJ_SpaceHeat

Table S367: Coefficients for space heating using hydrogen boilers.

5.9.7.5 Space heating using heat pumps

This process space heating using heat pumps. It assumes that the heat source is available without any additional resource demands; the only demand is for the electricity to drive the heat pump. This is derived from an estimate of the Seasonal coefficient of Performance (SPF) where,

$$SPF = \frac{Heat\ energy\ output}{Electricity\ input}$$

Both ground source and air source heat pumps (GSHP and ASHP respectively) are included within this process. It is assumed that 20% of the installations would be GSHP while the remainder are ASHP; consistent with the ratio of the two technologies considered in the modelling by Element Energy Ltd (2021). Currently 85% of worldwide sales of heatpumps for buildings are air-source as they are easier to install (IEA, 2022b); the share may be expected to change with more support for ground-source installations or with increasing energy prices. The estimated value is given in Table S368 and the resulting coefficients in Table S369.

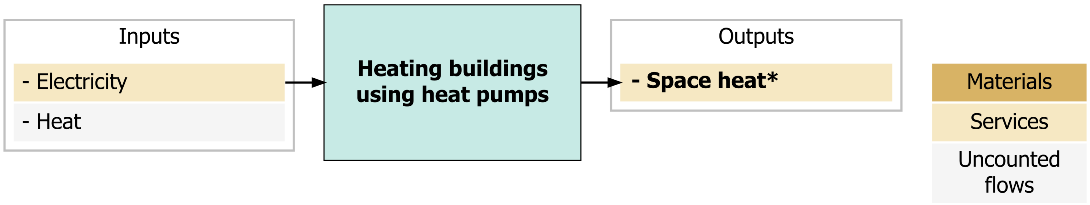

Figure S146: Inputs and outputs for space heating with heat pumps.

Property	Value	Unit	Justification
ASHP SPF (space heating)	3.4	EJ_SpaceHeat/EJ_Elec	Mid range estimate from (element energy, 2021)
GSHP SPF (space heating)	4.0	EJ_SpaceHeat/EJ_Elec	Mid range estimate from (element energy, 2021)
Share which are ground source	20%		Approx ratio in (element energy, 2021)
**Average SPF**	**3.5**	**EJ_SpaceHeat/EJ_Elec**	**Scaled average**

Note:
ASHP: Air-Source Heat Pump; GSHP: Ground-Source Heat Pump; SPF: Seasonal Coefficient of Performance

Table S368: Seasonal coefficient of Performance (SPF) assumptions used to derive the coefficients for space heating using heat pumps. Data sources: (Element Energy Ltd, 2021)

Activity	Process	Resource	Inputs	Outputs	Unit
SpaceHeat	HeatPump	Electricity	-0.29		EJ/EJ_SpaceHeat
SpaceHeat	HeatPump	SpaceHeat		1.0	EJ_SpaceHeat/EJ_SpaceHeat

Table S369: Coefficients for space heating using heat pumps.

5.9.8 Cooking

This activity describes cooking food for consumption. Three delivery processes are currently included in the model (Table S370).

The 2018 averaged global efficiency for cooking is estimated as shown in Table S371. Coefficients for each delivery process are estimated in the following sections by comparing their efficiency against this 2018 baseline. Emissions from combustion are estimated using the emissions factors in the SI Part 2.

Activity	Process	Process Long Name
Cooking	Bio	Biomass Fuelled
Cooking	Gas	Gas powered
Cooking	Elec	Electrically Powered

Table S370: Delivery processes for this activity. The coefficients for each delivery process are given and explained in the remainder of this section.

Cooking delivery process	2018 final energy use, EJ	2018 consumption share	Process efficiency
Burning fuels (not modern gas cookers)	13	44%	30%
Natual gas	11	35%	40%
Direct electrical	6	21%	75%
**All cooking in 2018**	**31**	**100%**	**43%**

Table S371: The assumed process efficiency of each delivery process for cooking is estimated based on the values given by Manibog (1984), IEA ETSAP (2012) and Hager & Morawicki (2013). The share of consumption in 2018 is based on the assumed final energy use given in Table S349. The share of consumption for each process is used to find a scaled average process efficiency for cooking in 2018.

5.9.8.1 Biomass powered cooking

This process describes cooking using traditional biomass fuels. This type of cooking can have a wide range of efficiency depending on the fuel and stove type (2-40% according to Manibog (1984)). 30% is used for this analysis.

The demand for biomass is estimated from the efficiency values in Table S371 and energy density values in the SI Part 2. This is combined with the emissions factor for biomass (as given in the SI Part 2) to estimate the combustion emissions for cooking using biomass. The derived values are given in Table S372.

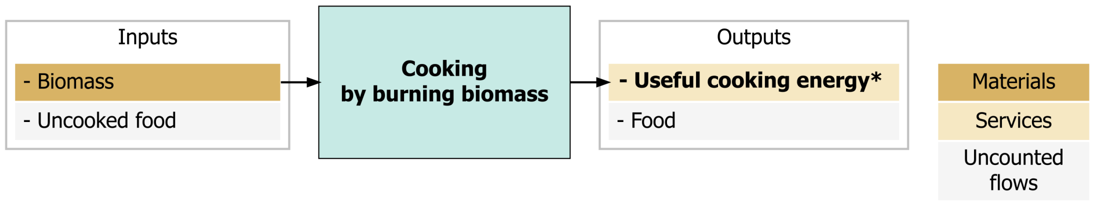

Figure S147: Inputs and outputs for cooking on biomass.

Activity	Process	Resource	Inputs	Outputs	Unit
Cooking	Bio	Cooking		1.0	EJ_Cooking/EJ_Cooking
Cooking	Bio	NonWoodBiomass	-0.048		Gt_DryBio/EJ_Cooking
Cooking	Bio	Wood	-0.048		Gt_DryBio/EJ_Cooking

Table S372: Coefficients for cooking on biomass fuels.

5.9.8.2 Natural gas powered cooking

This process describes cooking using natural gas. The demand for methane (natural gas) is estimated from the efficiency values in Table S371 and energy density values in the SI Part 2. This is combined with the emissions factor for methane (as given in the SI Part 2) to estimate the combustion emissions for cooking using natural gas. The derived values are given in Table S373.

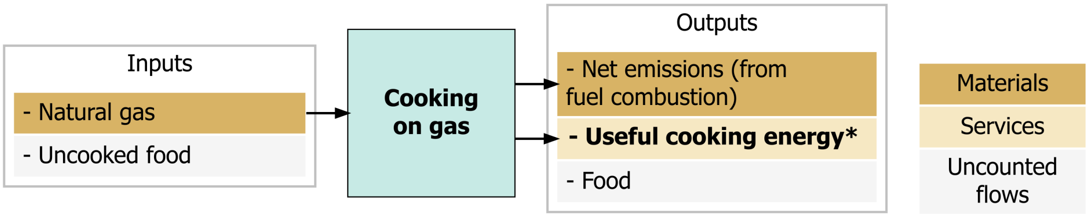

Figure S147: Inputs and outputs for cooking on biomass.

Activity	Process	Resource	Inputs	Outputs	Unit
Cooking	Gas	Cooking		1.0	EJ_Cooking/EJ_Cooking
Cooking	Gas	Methane	-0.024		EJ_Gas/EJ_Cooking
Cooking	Gas	NetEmissions		0.064	Gt_CO2e/EJ_Cooking

Table S373: Coefficients for cooking powered by natural gas.

5.9.8.3 Electrically powered cooking

This process describes cooking using electrically powered appliances (induction and coil hobs and electrically powered ovens). The coefficients, derived from the efficiency values in Table S371 are given in Table S374.

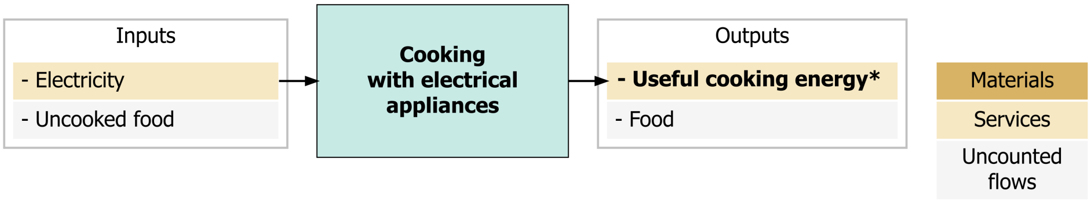

Figure S148: Inputs and outputs for cooking powered by electricity.

Activity	Process	Resource	Inputs	Outputs	Unit
Cooking	Elec	Cooking		1.0	EJ_Cooking/EJ_Cooking
Cooking	Elec	Electricity	-0.57		EJ/EJ_Cooking

Table S374: Coefficients for cooking powered by electricity.

5.9.9 Building Activities Coefficient Summary

ResourceFlow	Appliances_Elec	Cooking_Bio	Cooking_Elec	Cooking_Gas	Cooling_Elec	Lighting_Elec	SpaceHeat_Bio	SpaceHeat_Elec	SpaceHeat_Gas	SpaceHeat_H2Boiler	SpaceHeat_HeatPump	WaterHeating_Bio	WaterHeating_Elec	WaterHeating_Gas	WaterHeating_H2Boiler	WaterHeating_HeatPump
Electricity, EJ	-1.0		-0.57		-1.0	-1.0		-0.97			-0.29		-0.97			-0.37
Appliances, EJ_Appliances	1.0															
Cooking, EJ_Cooking		1.0	1.0	1.0												
NonWood-Biomass, Gt_DryBio		-0.048					-0.041					-0.041				
Wood, Gt_DryBio		-0.048					-0.041					-0.041				
Methane, EJ_Gas				-0.024												
NetEmissions, Gt_CO2e				0.064					0.062					0.061		
Cooling, EJ_Cooling					1.0											
Lighting, EJ_Lighting						1.0										
SpaceHeat, EJ_SpaceHeat							1.0	1.0	1.0	1.0	1.0					
Methane, Gt									-0.023					-0.023		
Hydrogen, Gt										-0.0096					-0.0096	
WaterHeating, EJ_WaterHeat												1.0	1.0	1.0	1.0	1.0

Table S375: Coefficients for building activities

5.10 Waste Management

This section outlines the derivation of coefficients for processing and managing waste, represented by ISIC divisions 36, 37 and 382 (Table S376). This is modelled as two activities: management of solid waste (WasteSolid) and liquid waste (wastewater).

Sector	Activity	Model Name	ISIC Code	End-Use
Waste	Management of Municipal Solid Waste	WasteSolid	382	✓
Waste	Wastewater treatment	Wastewater	36, 37	✓

Table S376: Model flows categorised by ISIC divisions, groups and classes for this Sector. For activities labelled as 'End-Use', the activity rate is set by the model inputs, rather than the demands of other activities. Note that ISIC 382 also includes production of biofuel and synthetic fuel which are not accounted here but in Section 5.6 Fuels and Feedstocks.

5.10.1 Sector-wide data sources

The coefficients within this section are mostly based on two key sources: (Kaza et al., 2018),and (Bogner et al., 2007). The quantity of waste produced and its distribution are taken from a report containing data compiled by the World Bank solid waste experts (Kaza et al., 2018). The values used here are linearly interpolated from 2016 and 2030 estimates to give a 2018 baseline. The emissions from waste management and processing are taken from a chapter focussing on Waste Management within the IPCC Fourth Assessment Report (Bogner et al., 2007) and from the EDGAR database.

Energy demand for waste processing activities is not included within these Activities as it is accounted for in Building Services (Section 5.9.1.1). The delivery processes for energy-use in buildings use IEA's world energy balances (2021a) to estimate energy use in buildings. The *Commercial & Public Activities* category used to estimate energy use in commercial buildings also includes water and waste treatment activities (ISIC Rev. 4 Divisions 33, 36-39) and so are not accounted for here to avoid double counting.

5.10.2 Solid Waste Management

This activity describes management of solid waste; the transformation of unwanted solid waste into safely stored waste, or other resources and services. In current approaches to waste management, the dominant emissions are carbon dioxide from incineration of waste containing fossil carbon (e.g. plastics), and methane from landfill (Eggleston et al., 2006). Only these sources of emissions are considered to derive the coefficients for this activity.

Although emissions from incineration can be simply modelled as linearly related to waste incinerated within a year, this is likely to be a gross oversimplification for methane from landfill since emissions continue for decades after waste disposal[9]. In this case, the linear approach has been used for landfill emissions (where methane emissions are assumed to be linearly related to waste landfilled within the same year) on the assumption that there is not significant variation in the quantity of waste landfilled over time. Given there are also large uncertainties in quantifying waste management emissions (Bogner et al., 2007), this simplified approach is only valid in the context of the whole system; the approach used here should not be used for analysis of the waste sector itself.

Activity	Delivery process	Detail
WasteSolid	Landfill	Solid waste management by depositing onto or into land (landfill)
WasteSolid	MethaneUse	Methane captured from landfill for use
WasteSolid	Incineration	Electricity generation from incineration of solid waste
WasteSolid	Recycling	Waste recycled and/or reused
WasteSolid	Feedstock	Use of waste as a downstream fuel or as a feedstock for fuel production

Table S378: Delivery processes for management of solid waste.

[9]Methane emissions from landfill are dependent on the environmental conditions of the site, the constitution of the waste, and continue "for several decades after waste disposal" (Bogner et al., 2007). Since the coefficients are derived based on methane emissions from landfill in 2018, they are only valid if there is a small temporal variation in the quantity of waste landfilled, global temperatures and the operation of landfill sites.

5.10.2.1 Solid-waste deposited into or onto land (landfill)

This delivery process describes solid waste deposited on land. This may be landfill, composting, or on an open dump. The coefficients are derived by estimating the methane emissions per unit of waste deposited from the 2018 landfill methane emissions and 2018 quantity of waste deposited on land. Emissions may in reality be higher since open dumps are likley to have been turned to landfill sites by 2050 which have higher associated methane emissions (Bogner et al., 2007). This may be compensated to some extent by increased use of methane management techniques such as "biocovers" which maximise oxidation of waste, reducing the formation of methane (Bogner et al., 2007).

The coefficients are derived from the assumptions in Table S379 and are listed in Table S380. To derive the coefficients, it was assumed that landfill methane dominate, consistent with Bogner et al. (2007). Energy use for waste management is accounted for in Building Activities, as discussed in Section 5.10.1. Residual emissions are estimated from the values given by Bogner et al. (2007).

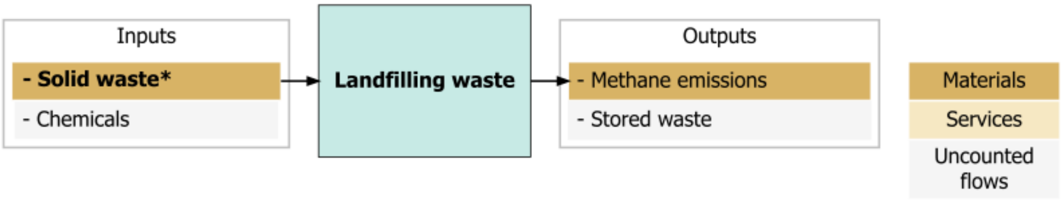

Figure S149: Inputs and outputs for landfilling waste.

Parameter	Quantity	Unit	Justification
**Summary of key data inputs and assumptions**			
Global solid waste generation, 2018	2.1	Gt	Interpolated from p25 of Kaza et al. (2018)
Methane emissions from landfill	0.85	Gt CO2e	Estimated from Bognor et al. 2007 projections
Share of solid waste deposited on land (landfill, composting, open dumps)	75%		2016 share as given in Fig 2.12 of Kaza et al. (2018)
Waste disposed on land in 2018	1.5	Gt	Share of waste disposed on land in 2016 x 2018 global waste
**Calculated coefficients (outputs)**			
Rate of methane generation (2018)	0.55	Gt CO2e/Gt waste	Methane generated/landfilled waste

Table S379: Assumptions used to derive coefficients for solid waste management via conventional processes and the 2018 global distribution of approaches. References: (Kaza et al., 2018), (Bogner et al., 2007)

Activity	Process	Resource	Inputs	Outputs	Unit
WasteSolid	Landfill	NetEmissions		0.55	Gt_CO2e/Gt_WasteSolid
WasteSolid	Landfill	WasteSolid	-1.0		Gt_WasteSolid/Gt_WasteSolid

Table S380: Coefficients for solid waste management via conventional processes and the 2018 global distribution of approaches.

5.10.2.2 Landfill with methane capture and use

This process describes landfilling waste using technologies to capture the methane produced for use by other processes (eg electricity generation). Unlike the delivery process which considers landfill as any form of waste management that deposits waste on land, this delivery process does not include composting and open-dump waste to find the rate of methane emissions. This is because methane emissions from landfill sites are assumed to dominate.

The coefficients are derived from the assumptions in Table S379 and are listed in Table S380. Methane generated per t waste is estimated from waste production data from Kaza et al. (2018) and landfill emissions estimates from Bogner et al. (2007). It is assumed that 85% of the methane generated is collected, representative of modern landfill management with comprehensive collection system according to Jardine et al. (2003). Energy use for waste management is accounted for in Building Activities, as discussed in Section 5.10.1.

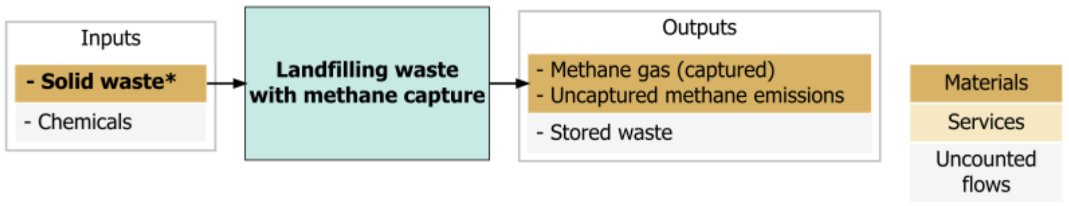

Figure S150: Inputs and outputs for landfilling waste with methane capture.

Parameter	Quantity	Unit	Justification
**Summary of key data inputs and assumptions**			
Global solid waste generation, 2018	2.1	Gt	Interpolated from p25 of Kaza et al. (2018)
Share of solid waste sent to landfill	37%		2016 share as given in Fig 2.12 of Kaza et al. (2018)
Landfill quantity in 2018	0.8	Gt	Share of waste sent to landfill in 2016 x 2018 global waste
Methane emissions from landfill, 2018	0.85	Gt CO2e	Estimated from Bognor et al. 2007 projections
Share of methane collected	85%		Representative of modern landfill management with comprehensive collection system (Jardine et al., 2003)
Methane GWP	27	Gt CO2e/Gt Methane	100 year Global Warming Potential IPCC AR6 values
**Calculated coefficients (outputs)**			
Methane generation	0.042	Gt Methane/Gt waste	Methane emissions / landfilled waste / GWP
Methane capture	0.036	Gt Methane/Gt waste	Share captured x generation
Residual methane emissions	0.17	Gt CO2e/Gt waste	(methane generation - capture) x GWP

Table S381: Assumptions used to derive coefficients for landfill with methane capture. References: (Kaza et al., 2018), (Bogner et al., 2007)

Activity	Process	Resource	Inputs	Outputs	Unit
WasteSolid	MethaneUse	NetEmissions		0.17	Gt_CO2e/Gt_WasteSolid
WasteSolid	MethaneUse	Methane		0.036	Gt/Gt_WasteSolid
WasteSolid	MethaneUse	WasteSolid	-1.0		Gt_WasteSolid/Gt_WasteSolid

Table S382: Coefficients for landfill with methane capture.

5.10.2.3 Incineration

This process describes incineration of solid waste. The coefficients are derived from the assumptions in Table S379 and are listed in Table S380. Electricity generated per t waste is estimated to be similar to the current dominant form

of waste to energy technology, according to Klinghoffer et al. (2013). Residual emissions are estimated by assuming a carbon content of waste. Energy use for waste management is accounted for in Building Activities, as discussed in Section 5.10.1.

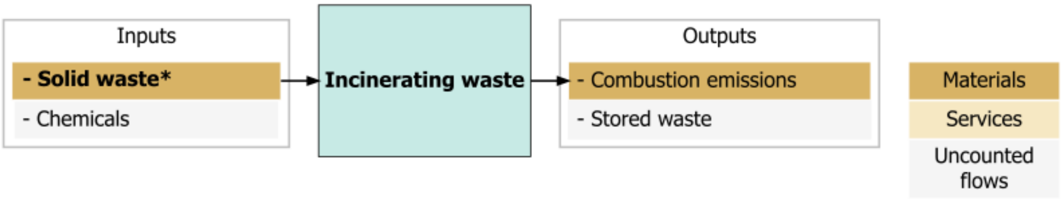

Figure S151: Inputs and outputs for incinerating waste.

Parameter	Quantity	Unit	Justification
**Summary of key data inputs and assumptions**			
Energy content of incinerated waste	11	MJ/kg	Chemical analysis of waste suggests an energy content of around 12 MJ/kg, EU waste after recycling contains 10 MJ/kg (Klinghoffer 2013)
Approximate efficiency of generation (check)	20%		(energy generated) / (energy content) NB: Bogner et al. 2007 note efficiencies in the range 15 - >20%
Assumed carbon content of waste	30%		US waste carbon content, according to Klinghoffer (2013)
**Calculated coefficients (outputs)**			
Energy generation	2.2	GJ/t waste	The dominant WTE technology (gate combustion) can generate 1.1 to 2.5 GJ/ton (Klinghoffer 2013)
Emissions from combustion	1.1	Gt CO2e/Gt waste	(Carbon content) / (Carbon atomic mass) x (CO2 mass)

Table S383: Assumptions used to derive coefficients for incinerating waste. References: (Kaza et al., 2018), (Bogner et al., 2007)

Activity	Process	Resource	Inputs	Outputs	Unit
WasteSolid	Incineration	NetEmissions		1.1	Gt_CO2e/Gt_WasteSolid
WasteSolid	Incineration	Electricity		2.2	EJ/Gt_WasteSolid
WasteSolid	Incineration	WasteSolid	-1.0		Gt_WasteSolid/Gt_WasteSolid

Table S384: Coefficients for incinerating waste.

5.10.2.4 Recycling

This process describes processing of waste to provide secondary materials for other processes. In the current model, the resource flows of most materials are not interlinked between processes, but instead treated as exogenous. This is is the case for the main output of recycling - scrap materials for secondary production processes.

Energy use for waste management is accounted for in Building Activities, as discussed in Section 5.10.1 and energy use for production of secondary materials is accounted for in the Industry Sector (Section 5.7). There may be emissions which arise from cleaning and pre-processing stages but these are assumed to be relatively small. As such there are no resource requirements for recycling accounted for in this process.

Although waste includes both recyclable and unrecyclable components, waste input to this process is assumed to be 100% recyclable for simplicity. This means that the share of this delivery process represents the share of waste recycled. An alternative (more granular) approach would be to disaggregate the resource waste into waste types with a separate activity to manage each type of waste. This could be added into the model in future work (see SI Part 2.3).

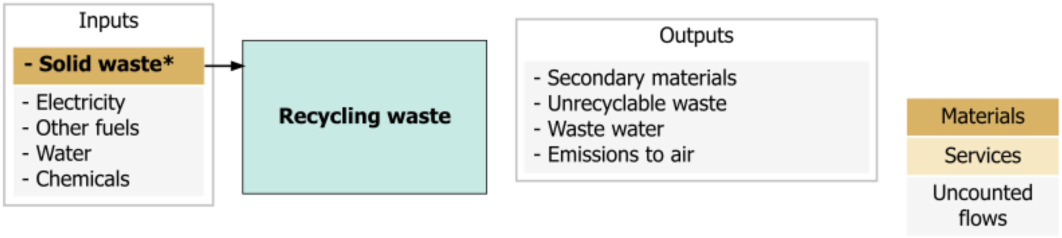

Figure S152: Inputs and outputs for recycling waste.

Activity	Process	Resource	Inputs	Outputs	Unit
WasteSolid	Recycling	WasteSolid	-1.0		Gt_WasteSolid/Gt_WasteSolid

Table S385: Coefficients for recycling waste.

5.10.2.5 Processing biogenic waste for use as a fuel or feedstock

This process describes processing waste such that biomass can be used as a fuel or feedstock for other processes. Although waste includes both biogenic and fossil carbon, for simplicity and transparency, only biogenic waste is assumed for the coefficients. This means that the share of this delivery process represents the share of waste extracted as biogenic materials for downstream uses. An alternative (more granular) approach would be to disaggregate the resource waste into biogenic and fossil waste with a separate activity to manage each type of waste. This could be added into the model in future work (see SI Part 2.3). This assumption means that the share of this delivery process should be constrained to be less than the expected share of biogenic waste.

Energy use for waste management is accounted for in Building Activities, as discussed in Section 5.10.1

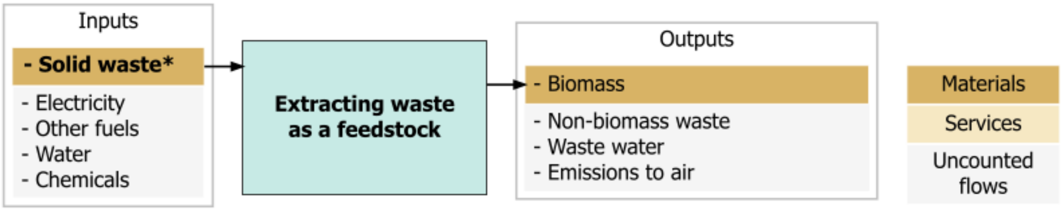

Figure S153: Inputs and outputs for using waste as a fuel or feedstock.

Activity	Process	Resource	Inputs	Outputs	Unit
WasteSolid	Feedstock	NonWoodBiomass		1.0	Gt/Gt_WasteSolid
WasteSolid	Feedstock	WasteSolid	-1.0		Gt_WasteSolid/Gt_WasteSolid

Table S386: Coefficients for extracting biogenic waste for use as fuels or feedstocks.

5.10.3 Wastewater Treatment

This activity describes processing and cleaning wastewater. Wastewater is broadly defined as the return flows from domestic and industrial water use (Jones et al., 2021); this is essentially 'used' water excluding agricultural runoff, which is rarely collected (Jones et al., 2021). Wastewater may be either discharged directly into the environment (e.g. by open defecation or direct river discharge) or collected and treated. Collection may be via sewerage systems (pipe transport) with centralised treatment centres, or 'on-site' collection, for instance in pit latrines or using septic tanks (Orner & Mihelcic, 2018). The main sources of emissions from wastewater are methane and nitrous oxide. These may arise at any stage source to disposal but are much more significant where collection and treatment are lacking (Bogner et al., 2007). Jones et al. (2021) estimate that around 63% of wastewater produced globally is currently collected, of which 84% is treated. However, the level of treatment is difficult to determine (Jones et al., 2021), and emissions are dependent on the collection and treatment system, local conditions, and wastewater composition (Bogner et al., 2007) which makes detailed analysis challenging. As such, the analysis here has been simplified into two delivery processes: the first assumes current levels and approaches of collection and treatment, the second assumes a best-case scenario of elimination of emissions, based on the review of Bogner et al. (2007) that "when efficiently applied, wastewater transport and treatment technologies reduce or eliminate GHG generation and emissions".

The activity metric for wastewater treatment has been taken as the volume of wastewater produced globally, since this is consistent with a mass balanced approach, and there is available data. It is worth noting, however, that if the delivery processes were more detailed (represented different collection and treatment systems), the choice of technology would impact the necessary volume of wastewater; this can be seen in the flow diagram below since as we increase the volume of water used to process human waste (labelled sewage water) we are increasing the volume of wastewater. Jones et al. (2021) found that the volume of wastewater produced at a country level could be predicted well based on total population, GDP per capita, and access to wastewater activities, including flushing toilets and piped sewers. In future work, a flow of human sewage waste and industrial wastes might be a more appropriate activity metric.

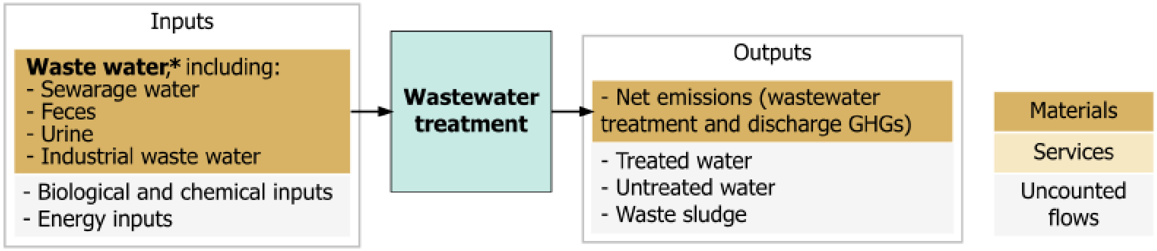

Figure S154: Inputs and outputs for wastewater treatment.

5.10.3.1 Conventional treatment processes (wastewater)

This delivery process describes the current processes and technologies of wastewater collection and treatment (Figure S154: Inputs and outputs for wastewater treatment.). The coefficients are derived from the assumptions in Table S387 and are listed in Table S388. Energy use for wastewater treatment is accounted for in Building Activities, as discussed in Section 5.10.1. Residual emissions are estimated from the EDGAR emissions inventory value for *wastewater treatment and discharge* summed for all countries (European Commission JRC (Datasets), 2022).

Parameter	Quantity	Unit	Justification
**Summary of key data inputs and assumptions**			
GHG emissions from wastewater, 2018	1.35	Gt CO2e	EDGAR v7, converted to CO2e using GWP-100 AR4
Global wastewater production	360	×10^9 m3/yr	As estimated by Jones et al. (2021) equivalent to 49 m3/person/yr
Global wastewater mass flow	360	Gt	Using an estimated density of 1000 kg/m3
**Calculated coefficients (outputs)**			
Total rate of water treatment emissions, 2018	0.004	GtCO2e/ Gt	Total emissions/mass flow wastewater

Table S387: Assumptions used to derive coefficients for wastewater management via conventional processes and the 2018 global distribution of approaches. References: (European Commission JRC (Datasets), 2022; Jones et al., 2021)

Activity	Process	Resource	Inputs	Outputs	Unit
Wastewater	NET	NetEmissions		0.0037	Gt_CO2e/Gt_Wastewater
Wastewater	NET	Wastewater	-1.0		Gt_Wastewater/Gt_Wastewater

Table S388: Coefficients for wastewater management via conventional processes and the 2018 global distribution of approaches.

5.10.3.2 Best practice wastewater treatment

This delivery process describes wastewater treatment, assuming that best practice wastewater transport and treatment technologies are in place, eliminating GHG generation and emissions (as noted possible in efficient applications by Bogner et al. (2007)). There are therefore zero other resource demands for this process since energy use for waste management is accounted for in the section on energy-use in buildings, as discussed in Section 5.10.1.

Activity	Process	Resource	Inputs	Outputs	Unit
Wastewater	BestPractice	Wastewater	-1.0		Gt_Wastewater/Gt_Wastewater

Table S389: Coefficients for wastewater management, assuming best practice. Since energy demands are not accounted for in this activity (see Section 5.10.1) and it is assumed that emissions can be eliminated, the only flow accounted in this process currently is the inflow of wastewater.

5.10.4 Waste Processing Coefficient Summary

ResourceFlow	WasteSolid_Feedstock	WasteSolid_Landfill	WasteSolid_MethaneUse	WasteSolid_Incineration	WasteSolid_Recycling	Wastewater_NET	Wastewater_BestPractice
NonWoodBiomass, Gt	1.0						
WasteSolid, Gt_WasteSolid	-1.0	-1.0	-1.0	-1.0	-1.0		
NetEmissions, Gt_CO2e		0.55	0.17	1.1		0.0037	
Methane, Gt			0.036				
Electricity, EJ				2.2			
Wastewater, Gt_Wastewater						-1.0	-1.0

Table S390: Coefficients for waste management processes.

5.11 Negative Emissions Technologies, NETs

This section outlines the basis to derive the coefficients for Negative Emissions Technologies (NETs). The term Negative Emissions Technologies (NETs) describes technologies (and sometimes practices and approaches) which extract Carbon Dioxide (CO_2) from the atmosphere and store them over a long duration. For this analysis, only carbon sequestration in geological reservoirs is considered permanent and so included in the model, as described in the Analysis Framework SI Document. NETs are modelled by two activities: one to capture atmospheric carbon dioxide, and the second to store captured carbon dioxide in a permanent form. These are listed in Table S391.

Sector	Activity	Activity Long Name	Produces
NETs	NetEmissions	Residual emissions management (negative emissions tech.)	Carbon dioxide gas
NETs	CO2Product	Carbon dioxide gas management	Stored carbon dioxide gas

Table S391: Activities within this chapter

5.11.1 Residual emissions management

This activity describes the process of converting residual emissions into carbon dioxide (for downstream use or storage). Downstream storage of carbon-dioxide gas is accounted for in the carbon storage activity (Section 5.11.2). Delivery processes for management of emissions are outlined in Table S392. The details for each process are outlined in the rest of this section.

Activity	Delivery process	Detail
NetEmissions	BECC	Bio Energy with Carbon Capture
NetEmissions	DAC	Direct Air Capture

Table S392: Delivery Processes for emissions management.

5.11.1.1 Direct Air Capture, DAC

This process describes the removal of carbon dioxide from the atmosphere by Direct Air Capture (DAC) to produce high purity carbon-dioxide gas at near ambient pressure. DAC is a technology which typically uses fans to blow air over chemicals which bind carbon dioxide at room temperature and pressure. The carbon dioxide is then re-released using higher temperatures, lower pressures or chemical reactions. Although there are many different technologies proposed for DAC only two technologies (liquid solvent and solid sorbent systems) are at a pilot level and so currently considered in this process. Processes could be added in future to represent other technologies, or to split this process, following the guidance in SI Part 2.3.

There are used by three dominant DAC ventures (Climeworks, Global Thermostat and Carbon Engineering) which use these two technologies. Liquid solvent based processes are used by Carbon Engineering, and solid sorbent processes, used by the other two ventures (McQueen et al., 2021). There is significant variation in estimates of energy demand for both systems, likely because most studies are based on theoretical modelling, rather than real operational systems. For example Keith et al. (2018) estimate the thermal energy requirement for a liquid solvent to be 5.3 GJ/t CO_2 captured while NASEM (2019) estimate a lower bound of 8.4 GJ/t for these types of systems; Keith et al. (2018) assume significant heat integration which NASEM (2019) do not include because there is a “lack of clarity about heat integration approaches in the open literature”. Similarly, NASEM (2019) estimate liquid solvents have significantly higher overall energy demands compared to solid sorbent systems (8.44-12.4 GJ/t and 4.0-5.9 GJ/t, respectively) but the IEA (2022a) estimate the opposite (liquid systems demanding around 6.5 GJ/t compared to 9 GJ/t for solid sorbent systems).

Given this wide variation in energy demand estimates, the two technologies are considered together in this process. From the literature reviewed in this section (see Table S393 and Table S394), all of the near-commercial approaches appear to have very broadly similar energy requirements, and within that, distribution of thermal and electrical energy requirements. In both cases, thermal energy is needed for regeneration of the sorbent or solvent, and electricity is needed for fans and pumps. Solvent-based systems however require higher temperatures for regeneration than solid

sorbent systems (up to 900°C compared to <150°C), and so are more limited in the heat sources they can use (NASEM, 2019). This has not been considered in the current process coefficients and could increase the overall demands.

The coefficients are estimated from data for Climeworks solid sorbent technology, assuming the mid-range value between future targets and measured data where a heat-pump is used (as given in Table S393). This is comparable with the lower estimates from other literature in 2015 (Table S394) if we assume that heat-pumps are combined with waste heat to provide the thermal energy. This is an optimistic assumption, especially given potential scarcity of waste heat; real demands may be higher. If natural gas were used as the fuel instead of electricity, the process would produce around 0.5 kg CO_2/kg CO_2 captured (using the values for a gas powered process in Table S393 and an emissions factor of 60 kg/GJ). This has not been included. The chosen coefficient is consistent with the value chosen by Fasihi et al. (2019) for fully electrified high temperature (HT) aqueous solution DAC for their techno-economic assessment of DAC technologies (5.5 GJ electricity/t CO_2).

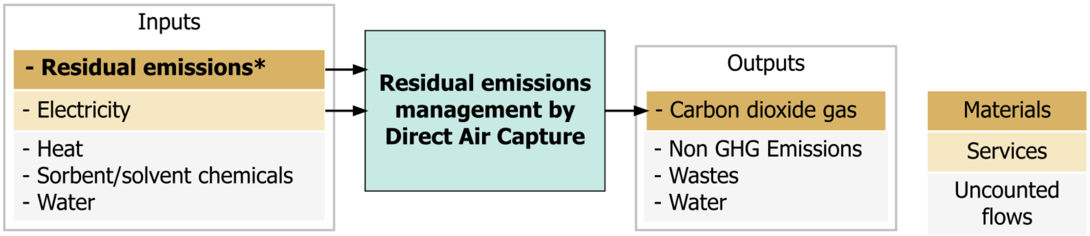

Figure S155: Inputs and outputs for emissions management by Direct Air Capture (DAC).

DAC type	Estimation basis	Source	Elec-tricity	Gas	Thermal	Electricity for a heat pump
Solvent	Carbon Engineering	McQueen et al. (2021)		8.8		
Solvent	Carbon Engineering	McQueen et al. (2021)	1.3	5.2		
Solid sorbent	Climeworks long term energy requirement projections	Buettler et al. (2019) - Climeworks	1.4		5.8	
Solid sorbent	Climeworks: measured data	Deutz & Bardow (2021)	2.5		11.9	
Solid sorbent	Climeworks: measured data	Deutz & Bardow (2021)	2.5			4.7
Solid sorbent	Climeworks: future targets	Deutz & Bardow (2021)	1.8		5.4	
Solid sorbent	Climeworks: future targets	Deutz & Bardow (2021)	1.8			2.2

Table S393: Summary of estimated energy requirements of pilot stage commercial ventures. References: (McQueen et al., 2021), (Beuttler et al., 2019), (Deutz & Bardow, 2021)

Literature survey values	Thermal	Electrical	TOTAL
Lower	6	1.1	7.1
Middle	8	1.5	9.5
Upper	10	1.9	11.9

Table S394: Summary of energy demand data from from the literature review by Broehm et al. (2015).

Activity	Process	Resource	Inputs	Outputs	Unit
NetEmissions	DAC	CO2Product		1.0	Gt_CO2/Gt_CO2
NetEmissions	DAC	Electricity	-5.6		EJ/Gt_CO2
NetEmissions	DAC	NetEmissions	-1.0		Gt_CO2e/Gt_CO2

Table S395: Coefficients for Direct Air Capture (DAC) of carbon dioxide.

5.11.1.2 Bio-Energy with Carbon Capture (and Storage), BECC

This process describes the removal of carbon dioxide from the atmosphere by Bio-Energy with Carbon Capture (BECC) as shown in Figure S156. Storage is not included in this process but in the process for carbon dioxide storage (Section 5.11.2) to allow for Carbon Capture and Use (CCU) applications. BECC is a technology which uses biomass to generate electricity or heat, and captures the carbon dioxide produced in the process from the concentrated flue gas. The captured carbon dioxide can then be used in chemical or industrial processes, or compressed for storage in geological formations. Since the carbon in biomass originated as carbon in atmospheric carbon dioxide (which the plant sequestered into biomass by photosynthesis), any captured carbon represents a reduction in atmospheric emissions.

For any potential facility, the design and operation may prioritise efficiency of either electricity generation or carbon capture (Almena et al., 2022). To reflect this breadth there are two processes for BECCS in this model; one of which is this process, representing a system optimised for carbon capture, the other is optimised for electricity generation and quantified in Section 5.4.2.7.

The coefficients are based on a process optimised for carbon carbon capture, modelled by Almena et al. (2022). The modelled process uses wheat straw in the original paper but it is assumed that using wood biomass would not significantly change the estimated resource flows. The values modelled by Almena et al. (2022) are shown in Table S396 and the resulting coefficients are given in Table S74.

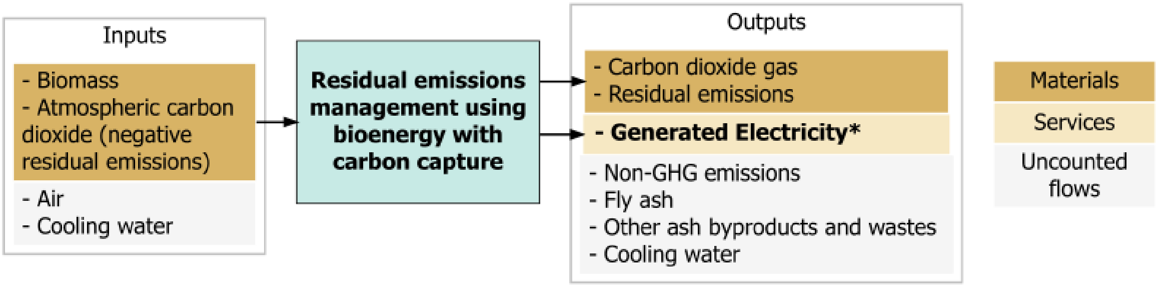

Figure S156: Inputs and outputs for emissions management by Bio-Energy with Carbon Capture (BECC). Carbon sequestered during biomass growth and then captured in this process is accounted for here (giving net-negative emissions) as explained in the SI Part 1.

Variable	Value	Unit	Source
**Summary of key data and assumptions**			
CO2 captured per electricity generated	3800	kg CO2/MWh	Almena et al. 2022
Net power output	22	MW	Almena et al. 2022
Biomass input per hour	58	t/ hr	Almena et al. 2022
CO2 captured per hour	83	t CO2/hr	Captured CO2 x Net power output
**Calculated coefficients (inputs)**			
Biomass	0.70	kg/kg CO2	Biomass input/CO2 captured (per hour)
Carbon dioxide sequestered	1.00	kg CO2/ kg CO2	Uncaptured emissions are compensated by carbon sequestration in growing biomass but sequestered emissions are net-negative
**Calculated coefficients (outputs)**			
Electricity	0.95	GJ/ t CO2	Net power output/CO2 captured (per hour)

Table S396: Key assumptions for estimating the coefficients for Bio-Energy with Carbon Capture (BECC). Data is taken from Tables 4 and 5 of Almena et al. (2022). Carbon sequestered in biomass during growth is not accounted for the model unless it is permanently sequestered, as explained in the SI Part 1.

Activity	Process	Resource	Inputs	Outputs	Unit
NetEmissions	BECC	Wood	-0.70		Gt_DryBio/Gt_CO2e
NetEmissions	BECC	CO2Product		1.0	Gt_CO2/Gt_CO2e
NetEmissions	BECC	Electricity		0.95	EJ/Gt_CO2e
NetEmissions	BECC	NetEmissions	-1.0		Gt_CO2e/Gt_CO2e

Table S397: Coefficients for Bio-Energy with Carbon Capture (BECC). Carbon sequestered during biomass growth and then captured in this process is accounted for here (giving net-negative emissions) as explained in the SI Part 1.

5.11.2 Carbon dioxide storage

This activity describes how high-purity carbon dioxide gas is managed: the processes either store carbon-dioxide gas permanently by compression and injection into geological reservoirs, or release carbon-dioxide gas as *NetEmissions*. The model is configured in this way to enable comparison with historical data (where there is no carbon storage), and to allow for the possibility of carbon capture and use (CCU) applications.

Sector	Activity	Activity Long Name	Produces
NETs	NetEmissions	Residual emissions management (negative emissions tech.)	Carbon dioxide gas
NETs	CO2Product	Carbon dioxide gas management	Stored carbon dioxide gas

Table S398: Activities within this chapter

5.11.2.1 Net-Zero Carbon Dioxide Storage

This delivery process describes the compression and storage of captured carbon dioxide in geological formations (Figure S157). The energy for compression and storage of the gas is estimated to be around 0.5 MJ / kg CO_2, supplied by electricity, consistent with the value used by NASEM (2019) (0.48 MJ/ kg CO_2). This value is between estimates of current requirements and theoretical modelling used in other studies.

According to Lask et al. (2021), energy demands are around 1.6 EJ/Gt CO_2 considering current electricity requirement for a case in Croatia (1.1 MJ/kg CO_2 for compression and 0.54 MJ/kg CO_2 for injection), but Madhu et al. (2021) estimate 0.43 EJ/Gt CO_2 (assuming compression and transport for 300km + storage in geological formation), while Deutz & Bardow (2021) assume 0.36 MJ/ kg CO_2 is needed for geological storage (assuming an 8-stage compressor and intercooling to 40°C is used to achieve an injection pressure of 150 bar from 1 bar).

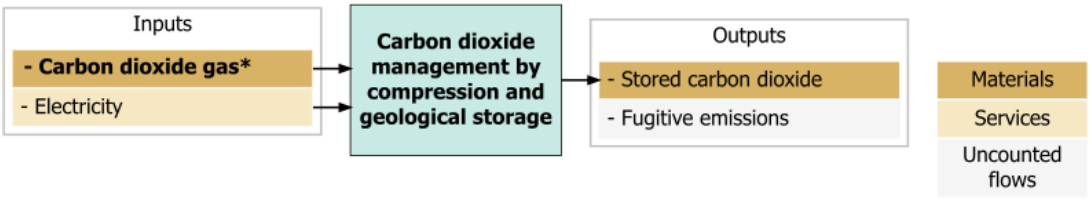

Figure S157: Inputs and outputs for the process to release captured carbon dioxide.

Activity	Process	Resource	Inputs	Outputs	Unit
CO2Product	NetZero	CO2Product	-1.0		Gt_CO2/Gt_CO2
CO2Product	NetZero	CO2Storage		1.0	Gt_CO2e/Gt_CO2
CO2Product	NetZero	Electricity	-0.50		EJ/Gt_CO2

Table S399: Coefficients for storing captured carbon dioxide.

5.11.2.2 No Carbon Dioxide Storage

This delivery process describes the release of high-purity carbon dioxide into the atmosphere (Figure S158). In this delivery process all remaining carbon-dioxide gas produced by upstream processes but not consumed is released again as *NetEmissions*. This delivery process is unlikely to be used for net-zero proposals but is needed to validate the the model against historical data.

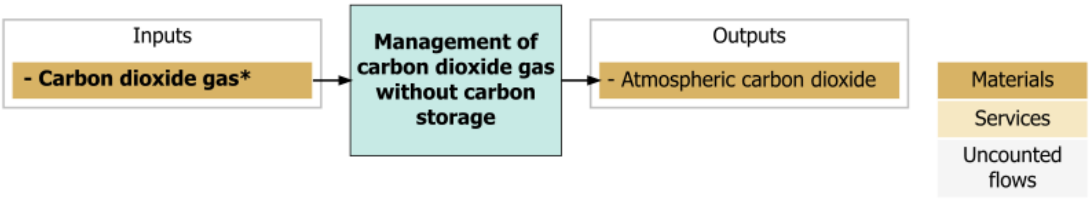

Figure S158: Inputs and outputs for the process which allows for residual emissions.

Activity	Process	Resource	Inputs	Outputs	Unit
CO2Product	NoStorage	CO2Product	-1.0		Gt_CO2/Gt_CO2
CO2Product	NoStorage	NetEmissions		1.0	Gt_CO2e/Gt_CO2

Table S400: Coefficients for re-releasing captured carbon dioxide.

5.11.3 NETs Coefficient Summary

ResourceFlow	CO2Product_NetZero	CO2Product_NoStorage	NetEmissions_DAC	NetEmissions_BECC
CO2Product, Gt_CO2	-1.0	-1.0	1.0	1.0
CO2Storage, Gt_CO2e	1.0			
Electricity, EJ	-0.50		-5.6	0.95
NetEmissions, Gt_CO2e		1.0	-1.0	-1.0
Wood, Gt_DryBio				-0.70

Table S401: Coefficients for Negative Emissions Technologies (NETs).

6 Model Inputs

6.1 Overview

This document contains an overview of the model inputs used in the main paper, and their justifications. Sections 6.2 and 6.3 summarise the final demands ($\mathbf{q_{Apparent}}$) and process shares (α), respectively, for all of the model runs. The tables use short names for the activities and processes, which each have a longer description, given in Section 6.8.

The following model runs are used in the main paper:

- **2018 run** - intended to act as a reference case, and to validate the model (as described in Part 4 of the SI).
- **The IEA Net-Zero Energy by 2050 scenario**, IEANZE (Section 6.4) - intended to represent the scenario developed by the IEA in their 2021 report (IEA, 2021b).
- **2050 Industry Accumulated Demands** - intended to represent the demand implied by prominant corporate strategies (Section 6.5).
- **CCS, Electrification, and Biomass Dominant Approaches** - each given to demonstrate changing technology choices without changing demand. The delivery process shares for each approach are determined manually, without following any particular defined scenario, and are listed in Section 6.3. The final activity rates are the same as the 2050 Accumulated Industry Demands run.
- **UK Government Strategy** - intended to represent the approach being taken by the UK government (and other similar countries with strong climate commitments) - Section 6.6.
- **The Low-Zero Emissions Resource Delivery scenario (Low ZER)** - given as an example of a proposal which would achieve net-zero greenhouse gases, given the probable constraints on supply of the three Zero Emissions Resources (ZERs) - Section 6.7.

Sections 6.4, 6.5, and 6.6 provide more detailed information on the inputs for the IEA Net-Zero Energy by 2050 scenario, the 2050 Industry Accumulated Demands scenario and the UK Government Strategy, respectively, while Section 6.7 describes the process to derive the delivery process shares for the Low ZER scenario.

6.2 Summary of final apparent activity rates ($q_{Apparent}$) for all runs

The end-user demands for all model runs used in the final paper are listed in Table S402 for easy comparison. The justifications for the choice of these rates are given in other sections, as described in Section 6.1.

Table S402: Summary of final activity rates for all runs. The Industry Demands rates are used for scenarios which are not otherwise specified (CCS, Electrification, and Biomass Dominant runs, and the UK Government Strategy run).

Sector	Activity	Units	2018	IEA NZE	Industry demands	Low ZER
Buildings	Appliances	EJ used for appliances	13	18	18	11
	Cooking	EJ used for cooking	31	41	41	26
	Cooling	EJ used for cooling	4.1	6.5	5.9	3.4
	Lighting	EJ used for lighting	8.9	12	12	7.4
	SpaceHeat	EJ used for space heating	39	48	48	33
	WaterHeating	EJ used for heating water	25	45	45	21
FuelsFeedstocks	OtherPetChem	Gt petrochemicals, not otherwise accounted	0.25	0.30	0.75	0.066
	Plastics	Gt plastics	0.35	0.43	0.58	0.093
Industry	Aluminium	Gt aluminium ingot	0.096	0.14	0.15	0.080
	Cement	Gt cement	4.0	3.2	3.9	0.15
	Construction	Gt 100% cement + 50% steel	4.9	4.2	5.2	4.1
	Food	10^15 kcal food supply	8.2	9.2	10	6.8
	Glass	Gt glass	0.16	0.15	0.18	0.087
	OtherIndustry	Billion Units: (Vehicles + Smartphones/10)	0.25	0.22	0.31	0.20
	Paper	Gt paper and board produced	0.40	0.52	0.58	0.33
	Steel	Gt steel	1.8	1.9	2.5	0.48
	Textiles	Gt textiles	0.096	0.087	0.14	0.025
Mining	MinedMetalsMinerals	Gt run-of-mine metals and minerals extracted	53	52	58	44
Transport	Aviation	10^12 revenue passenger km	8.5	9.8	11	1.1
	BusUse	10^12 passenger km	11	18	11	9.2
	CarUse	10^12 vehicle km	15	20	24	13
	RailFreight	x10^12 t km	13	21	27	11
	RailP	10^12 passenger km	4.4	7.0	8.8	3.7
	RoadFreight	x10^12 t km	27	30	39	22
	Shipping	x10^12 t km	110	140	190	28
Waste	WasteSolid	Gt Solid Waste	-2.0	-2.7	-3.4	-0.54
	Wastewater	Gt Wastewater	-360	-450	-460	-300

6.3 Summary of delivery process shares (α) for all runs

The delivery process shares for all model runs used in the final paper are listed in Table S403 for easy comparison. The justifications for the choice of these proportions are given in other sections, as described in Section 6.1.

Table S403: Summary of delivery process shares for all runs.

Activity	Process	Yr_2018	IEANZE	TechShare2050	CCS dominant	Elec. dominant	Biomass dominant	UK_2050	Low ZER
NonWoodBiomass	Residues	18%	26%	26%	26%	26%	26%	26%	26%
	Pasture	29%	20%	20%	20%	20%	20%	20%	20%
	ElecLowFertiliser	0%	54%	54%	54%	54%	54%	54%	54%
	NETs	53%	0%	0%	0%	0%	0%	0%	0%
RawFood	NonRuminantMeat	11%	16%	9%	9%	9%	9%	16%	0%
	PlantBasedFood	82%	0%	0%	0%	0%	0%	0%	0%
	PlantFoodLowMethaneRice	0%	50%	83%	83%	83%	83%	73%	0%
	PlantFoodNoRice	0%	23%	0%	0%	0%	0%	0%	100%
	RuminantFeedAdditives	0%	11%	8%	8%	8%	8%	12%	0%
	RuminantMeat	8%	0%	0%	0%	0%	0%	0%	0%
Wood	Elec	0%	90%	90%	0%	100%	50%	90%	100%
	NETs	100%	10%	10%	100%	0%	50%	10%	0%
Appliances	Elec	100%	100%	100%	100%	100%	100%	100%	100%
Cooking	Bio	31%	15%	15%	0%	0%	100%	0%	0%
	Elec	36%	80%	80%	0%	100%	0%	100%	100%
	Gas	33%	5%	5%	100%	0%	0%	0%	0%
Cooling	Elec	100%	100%	100%	100%	100%	100%	100%	100%
Lighting	Elec	100%	100%	100%	100%	100%	100%	100%	100%
SpaceHeat	Bio	23%	20%	20%	0%	0%	23%	0%	0%
	Elec	11%	5%	5%	11%	26%	11%	1%	20%
	Gas	47%	0%	0%	47%	0%	47%	7%	0%
	H2Boiler	0%	0%	0%	23%	0%	0%	14%	0%
	HeatPump	19%	75%	75%	19%	74%	19%	78%	80%
WaterHeating	Bio	26%	8%	8%	0%	0%	26%	0%	0%
	Elec	6%	4%	4%	6%	26%	6%	1%	20%
	Gas	46%	0%	0%	46%	0%	46%	10%	0%
	H2Boiler	0%	2%	2%	26%	0%	0%	20%	0%
	HeatPump	22%	85%	85%	22%	74%	22%	69%	80%
Electricity	Distribution	100%	100%	100%	100%	100%	100%	100%	100%
GeneratedElectricity	BECC	0%	1%	1%	5%	0%	5%	6%	0%
	Bio	2%	3%	4%	4%	0%	20%	2%	20%
	Coal	41%	0%	0%	0%	0%	0%	0%	0%
	CoalCCS	0%	1%	1%	1%	0%	0%	0%	0%
	Gas	23%	0%	0%	0%	0%	0%	0%	0%
	GasCCS	0%	1%	1%	20%	0%	0%	24%	0%
	NonEmitting	33%	93%	93%	70%	100%	75%	68%	80%

(continued)

Activity	Process	Yr_2018	IEANZE	TechShare2050	CCS dominant	Elec. dominant	Biomass dominant	UK_2050	Low ZER
Ammonia	CG	29%	0%	0%	0%	0%	0%	0%	0%
	Elec	0%	60%	70%	27%	100%	0%	30%	100%
	SMR	71%	0%	3%	3%	0%	100%	0%	0%
	SMRCCS	0%	40%	27%	70%	0%	0%	70%	0%
BioFuel	AdvBioFuel	0%	79%	79%	79%	79%	100%	79%	79%
	BioDiesel	30%	7%	7%	7%	7%	0%	7%	7%
	BioEthanol	70%	14%	14%	14%	14%	0%	14%	14%
HVCs	BioEth	0%	12%	12%	0%	12%	100%	12%	0%
	CCS	0%	32%	70%	100%	70%	0%	32%	0%
	MTOA	0%	50%	12%	0%	12%	0%	50%	0%
	NET	100%	6%	6%	0%	6%	0%	6%	100%
Hydrogen	ATRCCS	0%	0%	19%	25%	0%	19%	0%	0%
	CG	47%	0%	0%	0%	0%	0%	0%	0%
	CGCCS	0%	0%	0%	10%	0%	0%	0%	0%
	Elec	5%	62%	62%	40%	100%	62%	70%	100%
	SMR	48%	0%	0%	0%	0%	0%	0%	0%
	SMRCCS	0%	38%	19%	25%	0%	19%	30%	0%
Methane	Biogas	0%	25%	25%	0%	25%	38%	5%	0%
	FF	100%	62%	62%	100%	62%	62%	90%	100%
	SynDAC	0%	12%	12%	0%	12%	0%	5%	0%
Methanol	BioGas	0%	25%	25%	0%	25%	50%	20%	0%
	CCS	0%	25%	25%	50%	25%	0%	60%	0%
	Coal	15%	0%	0%	0%	0%	0%	0%	0%
	FromH2	0%	50%	50%	50%	50%	50%	20%	0%
	SMR	85%	0%	0%	0%	0%	0%	0%	100%
Oil	FF	100%	100%	100%	100%	100%	100%	100%	100%
OtherPetChem	NET	100%	100%	100%	100%	100%	100%	100%	100%
Plastics	Bio	0%	0%	0%	0%	0%	65%	0%	0%
	CCS	0%	35%	35%	65%	0%	0%	35%	0%
	Elec	0%	25%	25%	0%	65%	0%	25%	46%
	NET	82%	5%	5%	0%	0%	0%	5%	0%
	RecycleElec	18%	35%	35%	35%	35%	35%	35%	54%
Synfuel	FT	100%	100%	100%	100%	100%	100%	100%	100%
Urea	Elec	0%	90%	100%	0%	100%	0%	90%	100%
	Gas	100%	10%	0%	100%	0%	100%	10%	0%
Aluminium	InertAnodes	0%	40%	44%	44%	44%	44%	40%	0%
	NET	67%	4%	1%	1%	1%	1%	4%	0%
	ScrapElec	0%	56%	55%	55%	55%	55%	56%	100%
	ScrapNG	33%	0%	0%	0%	0%	0%	0%	0%

(continued)

Activity	Process	Yr_2018	IEANZE	TechShare2050	CCS dominant	Elec. dominant	Biomass dominant	UK_2050	Low ZER
Cement	Bio	5%	20%	20%	0%	15%	100%	0%	0%
	BioCCS	0%	23%	23%	20%	0%	0%	20%	100%
	CCS	0%	32%	52%	80%	80%	0%	80%	0%
	Coal	90%	0%	0%	0%	0%	0%	0%	0%
	H2	0%	18%	5%	0%	5%	0%	0%	0%
	NGas	5%	7%	0%	0%	0%	0%	0%	0%
Construction	Elec	0%	75%	60%	75%	100%	75%	75%	100%
	H2Power	0%	15%	10%	15%	0%	15%	15%	0%
	NET	100%	10%	30%	10%	0%	10%	10%	0%
Food	BioF	0%	20%	71%	0%	0%	100%	20%	0%
	Elec	0%	75%	24%	100%	100%	0%	75%	100%
	NET	100%	5%	5%	0%	0%	0%	5%	0%
Glass	Elec	0%	95%	65%	100%	100%	65%	95%	100%
	NET	100%	5%	35%	0%	0%	35%	5%	0%
OtherIndustry	Elec	0%	95%	95%	95%	100%	90%	95%	100%
	NET	100%	5%	5%	5%	0%	10%	5%	0%
Paper	Elec	0%	95%	95%	95%	100%	95%	95%	100%
	NET	100%	5%	5%	5%	0%	5%	5%	0%
Steel	BOF	66%	0%	8%	8%	0%	8%	0%	0%
	CCS	0%	29%	13%	30%	0%	13%	29%	0%
	EAF	29%	46%	40%	40%	100%	40%	46%	100%
	HDRI	0%	23%	22%	22%	0%	22%	23%	0%
	MethaneDRI	5%	3%	17%	0%	0%	17%	3%	0%
Textiles	Elec	0%	95%	50%	95%	100%	95%	95%	100%
	NET	100%	5%	50%	5%	0%	5%	5%	0%
ExtractedOilGas	Elec	0%	36%	36%	36%	36%	36%	36%	0%
	ElecNoFugitive	0%	64%	64%	64%	64%	64%	64%	0%
	NET	100%	0%	0%	0%	0%	0%	0%	0%
	NETNoFugitive	0%	0%	0%	0%	0%	0%	0%	100%
Coal	Elec	0%	27%	27%	27%	27%	27%	27%	45%
	ElecNoFugitive	0%	33%	33%	33%	33%	33%	33%	55%
	H2	0%	18%	18%	18%	18%	18%	18%	0%
	H2NoFugitive	0%	22%	22%	22%	22%	22%	22%	0%
	NET	100%	0%	0%	0%	0%	0%	0%	0%
MinedMetalsMinerals	Elec	0%	60%	60%	60%	100%	60%	60%	100%
	H2	0%	40%	40%	40%	0%	40%	40%	0%
	NET	100%	0%	0%	0%	0%	0%	0%	0%
CO2Product	NetZero	0%	100%	100%	100%	100%	100%	100%	100%
	NoStorage	100%	0%	0%	0%	0%	0%	0%	0%
NetEmissions	BECC	0%	67%	67%	67%	0%	100%	94%	100%
	DAC	0%	33%	33%	33%	100%	0%	6%	0%

(continued)

Activity	Process	Yr_2018	IEANZE	TechShare2050	CCS dominant	Elec. dominant	Biomass dominant	UK_2050	Low ZER
Aviation	Bio	0%	45%	38%	38%	20%	75%	25%	45%
	ElecT	0%	2%	2%	2%	10%	2%	6%	6%
	JetA	100%	20%	0%	0%	0%	0%	23%	16%
	LH2	0%	0%	23%	23%	30%	23%	21%	0%
	PtL	0%	33%	38%	38%	40%	0%	25%	33%
BusUse	BEV	4%	79%	79%	79%	100%	45%	61%	100%
	BioICE	1%	5%	5%	5%	0%	50%	0%	0%
	FFICE	95%	5%	5%	5%	0%	5%	39%	0%
	HFCEV	0%	5%	5%	5%	0%	0%	0%	0%
	SynICE	0%	5%	5%	5%	0%	0%	0%	0%
CarUse	BEV	1%	86%	86%	70%	100%	70%	98%	100%
	BioICE	1%	2%	0%	0%	0%	30%	0%	0%
	FFICE	98%	2%	5%	0%	0%	0%	2%	0%
	HFCEV	0%	9%	9%	0%	0%	0%	0%	0%
	SynICE	0%	0%	0%	30%	0%	0%	0%	0%
RailFreight	BioICE	1%	1%	1%	1%	0%	33%	0%	0%
	Elec	34%	92%	92%	60%	100%	60%	90%	100%
	FFICE	65%	7%	7%	7%	0%	7%	10%	0%
	SynICE	0%	0%	0%	32%	0%	0%	0%	0%
RailP	BioICE	1%	1%	1%	1%	0%	31%	0%	0%
	Elec	83%	92%	92%	60%	100%	62%	96%	100%
	FFICE	16%	2%	2%	2%	0%	2%	0%	0%
	HFCEV	0%	5%	5%	37%	0%	5%	4%	0%
RoadFreight	BEV	0%	30%	60%	30%	55%	30%	96%	50%
	BioICE	1%	10%	0%	0%	0%	30%	0%	0%
	CEV	0%	30%	10%	10%	45%	10%	0%	50%
	FFICE	99%	10%	0%	0%	0%	0%	4%	0%
	HFCEV	0%	20%	30%	60%	0%	30%	0%	0%
Shipping	Ammonia	0%	43%	43%	63%	63%	23%	24%	43%
	BioICE	0%	20%	20%	0%	0%	40%	24%	20%
	FFICE	100%	16%	16%	16%	16%	16%	3%	16%
	HFCEV	0%	20%	20%	20%	20%	20%	24%	20%
	LNG	0%	0%	0%	0%	0%	0%	0%	0%
	SynICE	0%	1%	1%	1%	1%	1%	24%	1%
WasteSolid	Feedstock	0%	20%	20%	20%	20%	20%	16%	20%
	Incineration	11%	5%	5%	5%	5%	5%	3%	5%
	MethaneUse	0%	20%	20%	20%	20%	20%	16%	20%
	Landfill	75%	0%	0%	0%	0%	0%	0%	0%
	Recycling	14%	55%	55%	55%	55%	55%	65%	55%
Wastewater	BestPractice	0%	100%	100%	100%	100%	100%	20%	100%
	NET	100%	0%	0%	0%	0%	0%	80%	0%

6.4 IEA Net-Zero Energy by 2050 Scenario

This set of inputs is intended to represent the IEA's Net Zero Energy by 2050 Scenario (IEA, 2021b). Where possible the data is taken directly from the original report (IEA, 2021b). Where that is not available, data is taken from the updates to the original report (IEA, 2023b), other IEA reports, policy documents or other industry sources (in that order of preference). The shares of each delivery process, and the final activity rates, are given in the remainder of this section. The tables use short names for the activities and processes, which each have a longer description, given in Section 6.8.

6.4.1 IEA NZE delivery process shares (α)

The following tables list the delivery process shares used for the IEA Net-Zero Energy by 2050 scenario, and their justifications.

Table S404: Data used as the input activity delivery process shares for the IEA Net-Zero Energy by 2050 scenario. Sources and notes are listed in Table S405 and Table S406.

Activity	Process Share	Process	IEANZE Source	IEANZE Note
NonWoodBiomass	26%	Residues	1	T1
	20%	Pasture	1	T1
	54%	ElecLowFertiliser	N/A	T2
RawFood	16%	NonRuminantMeat	2	T3
	50%	PlantFoodLowMethaneRice	2	T3
	23%	PlantFoodNoRice	2	T3
	11%	RuminantFeedAdditives	2	T3
Wood	90%	Elec	5, 6	T4
	10%	NETs	5, 6	T4
Appliances	100%	Elec	N/A	T5
Cooking	15%	Bio	3, 5	T6
	80%	Elec	3, 5	T6
	5%	Gas	3, 5	T6
Cooling	100%	Elec	N/A	T5
Lighting	100%	Elec	N/A	T5
SpaceHeat	20%	Bio	3	T7
	5%	Elec	3	T8
	0%	Gas	3	T9
	75%	HeatPump	3	T10
WaterHeating	8%	Bio	3	T11
	4%	Elec	3	T11
	0%	Gas	3	T11
	2%	H2Boiler	3	T11
	85%	HeatPump	3	T11
Electricity	100%	Distribution	N/A	T5
	1%	BECC	3	T12
	3%	Bio	3	T12

(continued)

Activity	Process Share	Process	IEANZE Source	IEANZE Note
GeneratedElectricity	1%	CoalCCS	3	T12
	0%	Gas	3	T12
	1%	GasCCS	3	T12
	93%	NonEmitting	3	T12
Ammonia	60%	Elec	3	T13
	40%	SMRCCS	3	T14
BioFuel	79%	AdvBioFuel	3	T15
	7%	BioDiesel	3	T15
	14%	BioEthanol	3	T15
HVCs	12%	BioEth	3	T16
	32%	CCS	3	T16
	50%	MTOA	3	T16
	6%	NET	3	T16
Hydrogen	62%	Elec	3	T17
	38%	SMRCCS	3	T17
Methane	25%	Biogas	3	T18
	62%	FF	3	T18
	12%	SynDAC	3	T18
Methanol	25%	BioGas	3	T19
	25%	CCS	3	T19
	50%	FromH2	3	T19
Oil	100%	FF	N/A	T5
OtherPetChem	100%	NET	N/A	T5
Plastics	35%	CCS	3	T20
	25%	Elec	3	T20
	5%	NET	3	T20
	35%	RecycleElec	3	T21
Synfuel	100%	FT	N/A	T5
Urea	90%	Elec	3	T22
	10%	Gas	3	T22
Aluminium	40%	InertAnodes	7	T23
	4%	NET	7	T23
	56%	ScrapElec	7	T23
Cement	20%	Bio	7	T24
	23%	BioCCS	7	T24
	32%	CCS	7	T24
	18%	H2	7	T24
	7%	NGas	7	T24
Construction	75%	Elec	9	T25
	15%	H2Power	9	T25
	10%	NET	9	T25
	20%	BioF	3	T26

(continued)

Activity	Process Share	Process	IEANZE Source	IEANZE Note
Food	75%	Elec	3	T26
	5%	NET	3	T26
Glass	95%	Elec	3	T22
	5%	NET	3	T22
OtherIndustry	95%	Elec	3	T22
	5%	NET	3	T22
Paper	95%	Elec	3	T22
	5%	NET	3	T22
Steel	29%	CCS	3	T27
	46%	EAF	3	T27
	23%	HDRI	3	T27
	3%	MethaneDRI	3	T27
Textiles	95%	Elec	3	T22
	5%	NET	3	T22
ExtractedOilGas	36%	Elec	3	T28
	64%	ElecNoFugitive	10	T29
Coal	27%	Elec	11	T30
	33%	ElecNoFugitive	11	T30
	18%	H2	11	T30
	22%	H2NoFugitive	11	T30
MinedMetalsMinerals	60%	Elec	12	T31
	40%	H2	12	T31
CO2Product	100%	NetZero	N/A	T32
NetEmissions	67%	BECC	3	T33
	33%	DAC	3	T33
Aviation	45%	Bio	3	T34
	2%	ElecT	3	T34
	20%	JetA	3	T34
	33%	PtL	3	T34
BusUse	79%	BEV	3	T35
	5%	BioICE	3	T35
	5%	FFICE	3	T35
	5%	HFCEV	3	T35
	5%	SynICE	3	T35
CarUse	86%	BEV	3	T36
	2%	BioICE	3	T37
	2%	FFICE	3	T37
	9%	HFCEV	3	T38
RailFreight	1%	BioICE	3	T39
	92%	Elec	3	T39
	7%	FFICE	3	T39
	1%	BioICE	3	T39

(continued)

Activity	Process Share	Process	IEANZE Source	IEANZE Note
RailP	92%	Elec	3	T39
	2%	FFICE	3	T39
	5%	HFCEV	3	T39
RoadFreight	30%	BEV	3	T40
	10%	BioICE	3	T41
	30%	CEV	3	T40
	10%	FFICE	3	T37
	20%	HFCEV	3	T42
Shipping	43%	Ammonia	3	T43
	20%	BioICE	3	T43
	16%	FFICE	3	T43
	20%	HFCEV	3	T43
	1%	SynICE	3	T43
WasteSolid	20%	Feedstock		T44
	5%	Incineration		
	20%	MethaneUse		
	55%	Recycling	3	T45
Wastewater	100%	BestPractice	N/A	Q46

Table S405: Sources used to assign the delivery process shares used for the IEA Net-Zero Energy by 2050 scenario, given in Table S404.

IEANZE Tech Share Reference Key	IEANZE Tech Share Reference
1	ZERs SI Part 1 - Analysis Framework Assumptions and Supply Estimations
2	Barrett, J., Pye, S., Betts-Davies, S., Broad, O., Price, J., Eyre, N., Anable, J., Brand, C., Bennett, G., Carr-Whitworth, R., Garvey, A., Giesekam, J., Marsden, G., Norman, J., Oreszczyn, T., Ruyssevelt, P., & Scott, K. (2022). Energy demand reduction options for meeting national zero-emission targets in the United Kingdom. Nature Energy 2022, 1–10. https://doi.org/10.1038/s41560-022-01057-y
3	IEA. (2021). Net Zero by 2050: A Roadmap for the Global Energy Sector. https://www.iea.org/reports/net-zero-by-2050
4	IEA. (2022). World Energy Outlook 2022. https://www.iea.org/reports/world-energy-outlook-2022
5	BEIS. (2021). Net Zero Strategy: Build Back Greener (Issue October). https://www.gov.uk/government/publications/net-zero-strategy
6	Committee on Climate Change. (2019). Net Zero Technical Report. May, 19–292. https://www.theccc.org.uk/publication/net-zero-technical-report/
7	IEA. (2023). Net Zero Roadmap: A Global Pathway to Keep the 1.5 °C Goal in Reach. https://www.iea.org/reports/net-zero-roadmap-a-global-pathway-to-keep-the-15-0c-goal-in-reach
8	IEA (2023), Cement, IEA, Paris https://www.iea.org/reports/cement-3, Licence: CC BY 4.0
9	Bellona.org. (2023). Database: Emission-free Construction Equipment (by manufacturer). https://bellona.org/database-emission-free-construction-equipment-by-manufacturer
10	McKinsey & Company. (2020). The future of oil and gas is now: How companies can decarbonize (Issue January). https://www.mckinsey.com/industries/oil-and-gas/our-insights/the-future-is-now-how-oil-and-gas-companies-can-decarbonize
11	IEA (2023), Global Methane Tracker 2023, IEA, Paris https://www.iea.org/reports/global-methane-tracker-2023, License: CC BY 4.0; McKinsey & Company. (2021). Creating the zero-carbon mine. https://www.mckinsey.com/industries/metals-and-mining/our-insights/creating-the-zero-carbon-mine
12	McKinsey & Company. (2021). Creating the zero-carbon mine. https://www.mckinsey.com/industries/metals-and-mining/our-insights/creating-the-zero-carbon-mine

Table S406: Notes about delivery process shares used for the IEA Net-Zero Energy by 2050 scenario, given in Table S404.

IEANZE Tech Share Note Key	IEANZE Tech Share Note
T1	Based on the distribution estimated for maximum availability of Biomass in 2050.
T2	Best case strategy assumed in absence of other info.
T3	No mention of diet in IEA (2021) document - instead, this data is based on p19 of Barrett et al. (2022) SI file (diet choices) for the UK and assumed that best farming practices are used.
T4	BEIS (2021): 100% "low carbon farming practices as a percentage of total farmers"; CCC (2019) assume 90% reduction in powertrain emissions by replacing fuels with hydrogen and electricity (although only 75% update by farmers of "on-farm practices").
T5	Only one delivery process
T6	Distribution of cooking technologies for the emerging market is given in Fig 4.11. In absence of other data, this is assumed to be 50 % of demand in 2050 with remaining 50% electrified, as suggested in UK strategy documents

(continued)

IEANZE Tech Share Note Key	IEANZE Tech Share Note
T7	IEA (2021) p 145: ”Bioenergy meets … more than 20% [of space heating energy needs] by 2050”
T8	Direct electrical space heating is estimated to be 5%, based on IEA (2021) p 145: ”homes using electricity for heating rise … [to] 55% in 2050”, and p20: ”50% of [buildings] heating demand met by heat pumps” in 2045
T9	IEA (2021) p 145: ”homes heated by natural gas falling… to less than 0.5% in 2050”
T10	In the absence of a district heating process, it is included under heat pumps. 55% space heating is assumed to come from heat pumps based on p20: ”50% of [buildings] heating demand met by heat pumps” in 2045. 20% assigned to district heating based on p145: ”District heat networks … provide more than 20% of final energy demand for space heating”
T11	IEA (2021) Fig 3.29. Heat pump process also include the share of heating from district heat and solar thermal (in the absence of more suitable processes). The ;other’ category is assigned to direct electrical.
T12	Table A.3 pg 198
T13	Ammonia assumed to mirror Hydrogen production
T14	Ammonia assumed to mirror Hydrogen production but with a higher proportion of SMR with CCS to achieve the split given for all chemicals in Fig 3.19, p126
T15	Estimated from Fig 3.7 p 108. NOTE: the model does not currently include a process for production with CCUS but this makes up 50% in the NZE report.
T16	Very little evidence. Estimated from Fig 3.19, p126, considering all fuels and feedstocks toegther
T17	p109: ”water electrolysis accounts for more than 60% of global production, and natural gas in combination with CCUS for almost 40%”
T18	Table A.2 pg 196
T19	Methanol is included as a low-carbon fuel so it is assumed here that it must be made from biogas or hydrogen, or with CCS. The share is loosely based on Fig 3.19, p126.
T20	Little direct infomation - assumed to follow strategy of primary chemicals (p126)
T21	Table 3.3 p131
T22	p20: More than 90% of industrial production is low-emissions.
T23	p95: 96% near zero emission production, and 56% from secondary production
T24	Approximated based on p96: 7% assigned to gas since 93% clinker production is ”near-zero emission”. Residual assigned based on the share of low emissions fuel . Hydrogen and electricity fuelled have been combined. NOTE that the model processes with CCS currently only include capture from the precalciner and so have significant residual emissions. The IEA likely also assume capture from the kiln, needed for true ”near-zero emission” production. Refer to the SI part 2B section on cement with CCS for more details of the model coefficient choices.
T25	No detailed information on construction in IEA (2021). Majority of low carbon construction equipment listed on the Bellona database (2023) are electically driven. Assume that ~10% of legacy equipment is still used by 2050.
T26	p20: More than 90% of industrial production is low-emissions. Assume both electrified and biofuel powered since both make up the final distribution of energy use in industry (biomass contributes 16% fuel compared to 46% electricity)
T27	Based on Table 3.3, p129
T28	p104: ”The end of all flaring … and significant electrification of upstream operations”
T29	McKinsey (2020) suggest around 85% of fugitive emissions are from upstream (including flaring and venting). These are easier to manage by reducing flaring & using leak detection and repair systems. Downstream leaks are more disperse and so harder to monitor and fix. Assume around 70% of upstream emissions are prevented and around 30% of downstream emissions.
T30	The IEA (2023) estimate that it’s possible to reduce over half of coal mine methane with existing technologies. Note that energy supply from recovered coal mine methane is not accounted for in the model (although this is only one of three key approaches mentioned in the text). Assume 55% fugitive emissions are avoided. There is no clear strategy for zero-emissions trucking and industry but McKinsey (2021) suggest electric vehicles are a front runner. Assume 60% electrified processes and 40% hydrogen powered.
T31	There is no clear strategy for zero-emissions trucking and industry but McKinsey (2021) suggest electric vehicles are a front runner. Assume 60% electrified processes and 40% hydrogen powered.

(continued)

IEANZE Tech Share Note Key	IEANZE Tech Share Note
T32	Assume all residual CO2 is stored
T33	1.9 Gt removed by BECC and DAC, of which 633 MtCO2 is DAC (p 55 and 199)
T34	Figure 3.25, p137. Additional warming impacts from aviation are not accounted for.
T35	79% of bus stock in 2050 assumed to be battery electric (table 2.5, p72). Residual assumed to be equally distributed amongst other modes.
T36	86% of car stock in 2050 is battery electric from Table 2.5, p72. BEV share of sales is 90% (Fig 3.23)
T37	Residual shares
T38	Around 10% sales from Fig 3.23, p134 (compared to 90% electric)
T39	Fig 3.25, p 137. No distinction between passenger and freight rail in the figure so this is assigned based on available delivery processes.
T40	59% share of heavy truck stock is electric by 2050, compared to 84% for vans (Table 2.5, p72). Assume 50% electric vehicles are catenery powered.
T41	pg 134: ”biofuels still meet about 10% of fuel needs for heavy-duty trucks in 2050”.
T42	From Fig 3.24, battery electric cover about 3/4 of driving distance with fuel cell electric covering remaining 1/4.
T43	Fig 3.25, p 137.
T44	pg 91: 44% of bioenergy in NZE is from organic waste streams (including MSW, other wastes and residues). Organic waste is around 40% of waste, according to Chen et al. (2020)
T45	Based on 2050 target rate for recycling

6.4.2 IEA NZE final activity rates ($q_{Apparent}$)

The following tables list the final activity rates used for the IEA Net-Zero Energy by 2050 scenario, and their justifications.

Table S407: Data used as the input activity demand for the IEA Net-Zero Energy by 2050 scenario. The data is given in terms of the *relative demand* (activity demand in 2050 compared to 2018), and *efficiency saving* (conversion device, passive system, and operational efficiency improvements which are not accounted for elsewhere). This framework for accounting for efficiency savings is described in SI Document 2A (Mathematical Framework). Sources and notes referred to in this table are given in Table S408 and Table S408.

Activity	Relative Demand	Actual Demand	Efficiency Saving	Process Rate	Units	Demand Note	Efficiency Note	Reference
Appliances	250%	33	46%	18	EJ used for appliances	D1	E1	1
Cooking	250%	77	46%	41	EJ used for cooking	D1	E1	1
Cooling	291%	12	46%	6.5	EJ used for cooling	D2	E2	1
Lighting	250%	22	46%	12	EJ used for lighting	D1	E1	1
SpaceHeat	250%	98	50%	48	EJ used for space heating	D1	E3	1
WaterHeating	250%	63	28%	45	EJ used for heating water	D1	E4	1
OtherPetChem	128%	0.32	5%	0.30	Gt petrochemicals, not otherwise accounted	D3	E5	1, 2
Plastics	128%	0.45	5%	0.43	Gt plastics	D3	E5	1
Aluminium	152%	0.15	5%	0.14	Gt aluminium ingot	D4	E5	3, 1, 2
Cement	101%	4.0	20%	3.2	Gt cement	D5	E6	1, 3
Construction				4.2	Gt 100% cement + 50% steel	D6	E7	N/A
Food	125%	10	10%	9.2	10^15 kcal food supply	D7	E5	1
Glass	100%	0.16	10%	0.15	Gt glass	D8	E5	1
OtherIndustry	100%	0.25	10%	0.22	Billion Units: (Vehicles + Smartphones/10)	D8	E5	1
Paper	145%	0.58	10%	0.52	Gt paper and board produced	D9	E5	4, 1, 2
Steel	109%	2.0	5%	1.9	Gt steel	D10	E5	1
Textiles	100%	0.096	10%	0.087	Gt textiles	D8	E5	1
MinedMetalsMinerals	110%	58	10%	52	Gt run-of-mine metals and minerals extracted	D11	E5	N/A
Aviation	192%	16	40%	9.8	10^12 revenue passenger km	D12	E8	1, 5
BusUse	200%	22	20%	18	10^12 passenger km	D13	E9	
CarUse	160%	24	20%	20	10^12 vehicle km	D14	E10	1
RailFreight	200%	27	20%	21	x10^12 t km	D15	E11	1
RailP	200%	8.8	20%	7.0	10^12 passenger km	D15	E9	1
RoadFreight	225%	60	50%	30	x10^12 t km	D16	E12	1, 6
Shipping	270%	290	50%	140	x10^12 t km	D17	E13	1 7
WasteSolid	166%	-3.4	20%	-2.7	Gt Solid Waste	D18	E14	1, 8
Wastewater	125%	-450	0%	-450	Gt Wastewater	D19		1

IEANZE Reference Key	IEANZE Reference
1	IEA. (2021). Net Zero by 2050: A Roadmap for the Global Energy Sector. https://www.iea.org/reports/net-zero-by-2050
2	IEA. (2022). World Energy Outlook 2022. https://www.iea.org/reports/world-energy-outlook-2022
3	IEA. (2023). Net Zero Roadmap: A Global Pathway to Keep the 1.5 °C Goal in Reach. https://www.iea.org/reports/net-zero-roadmap-a-global-pathway-to-keep-the-15-0c-goal-in-reach
4	IEA. (2020). Global paper and paperboard production in the Sustainable Development Scenario, 2010-2030. https://www.iea.org/data-and-statistics/charts/global-paper-and-paperboard-production-in-the-sustainable-development-scenario-2010-2030, Licence: CC BY 4.0
5	Mission Possible Partnership. (2022). Making Net-Zero Aviation Possible (Issue July). https://missionpossiblepartnership.org/wp-content/uploads/2023/01/Making-Net-Zero-Aviation-possible.pdf
6	Mulholland et al. (2018). The long haul towards decarbonising road freight – A global assessment to 2050
7	DNV GL. (2018). Energy Transition outlook 2018 - Maritime Forecast to 2050.
8	Kaza et al. (2018) What a Waste 2.0: A Global Snapshot of Solid Waste Management to 2050

Table S408: Sources used to assign the final activity rates used for the IEA Net-Zero Energy by 2050 scenario, given in Table S407.

IEANZE Demand Key	IEANZE Demand Note
D1	IEA (2021) p142: increase in energy activity demand related to rising population, increased floor area and income per capita (calculated as the multiplication of 29% increase to 2030 & 96% subsequent increase to 2050 as given in Fig 3.27)
D2	IEA (2021) p146: ”60% of households have an air conditioner in 2050, up from 35% in 2020” and, from Table A.5, ’Residential’ and ’Services’ floor areas increase by 80% & 60% respectively.
D3	From IEA (2021) Table A.5 (p200) production of primary chemicals increases by 28% compared to 2019 levels.
D4	From IEA (2023) p95, 146 Mt production in 2050
D5	From IEA (2021), Table A.5 (p200) production of cement stays approximately flat from 2018 levels to 4032Mt in 2050.
D6	Sum of 50% steel and 100% cement
D7	Assume scales linearly with population. From IEA (2021) p 50, population increases by 25% by 2050. From own logic: Additional increases likely if increased global wealth but these may be compensated by decreases in food waste.
D8	No obvious information, assume no change
D9	IEA (2020): Projected demand increase based on extrapolation of IEA Sustainable Development Scenario.
D10	From Table A.5 (p200) production of steel increases to 1987 Mt
D11	Some growth to account for metal demand growth while cement stays constant
D12	IEA (2021) Table A.5 (p200): passenger km increases by 92% compared to 2019 levels.
D13	No obvious information, assume scales similarly to passenger rail.
D14	IEA (2021) Table A.5 (p200): vehicle km driven increases by 60% compared to 2019 levels.
D15	Global transport final consumption for rail is approx. twice as large in 2050 as 2020 based on IEA (2021) Fig 3.22 p133
D16	IEA (2021) Table A.5 (p200): tkm increases by 125% compared to 2019 levels.
D17	IEA (2021) Table A.5 (p200): tkm increases by 170% compared to 2019 levels.
D18	Projected global waste generation from Kaza et al. (2018) Fig 2.5
D19	From IEA (2021) p 50, population increases by around 25% by 2050.

Table S409: Notes about the relative demand values used for the IEA Net-Zero Energy by 2050 scenario, given in Table

S407.

IEANZE Efficiency Key	IEANZE Efficiency Note
E1	IEA (2021) Table 2.3 (p66): Appliances consume 60% electricity compared to those in 2020. From Fig 3.27: behaviour changes & ”avoided demand” (e.g. via digitilisation) reduce emissions by ~10% to 2050
E2	IEA (2021) p146: ”High-performance building envelopes, including bioclimatic designs and insulation, can reduce the demand for space cooling by 30-50%”. From Fig 3.27: behaviour changes & ”avoided demand” (e.g. via digitilisation) reduce emissions by ~10% to 2050.
E3	IEA (2021) p146: ”High-performance building envelopes, including bioclimatic designs and insulation, can reduce the demand for space cooling by 30-50%”. From Fig 3.27: behaviour changes & ”avoided demand” (e.g. via digitilisation) reduce emissions by ~10% to 2050.
E4	Assume a 20% decrease in energy demands due to efficiency improvements (such as pipe insulation). From IEA (2021) Fig 3.27: behaviour changes & ”avoided demand” (e.g. via digitilisation) reduce emissions by ~10% to 2050
E5	IEA (2021) p128: efficiency improvements in industry are mentioned but are not quantified. If not specified explicitly, we assume a 10% saving across industrial production where the model coefficents are not based on best available technology, and 5% where coefficients already include efficiency improvements. This justified as the NZE industrial ”total final consumption ... is nearly 10% lower than in the STEPS by 2030” due energy and materials efficiency (from IEA (2022) WEO, p142).
E6	From IEA (2023) p96, kiln energy intensity is reduced by around 20% by 2050
E7	Sum of 50% steel and 100% cement
E8	IEA (2021) p132: ”more efficient operations”, including ”air traffic management, e.g. landing and take-off scheduling in aviation”. Value used is based the Mission Possible Partnership report on aviation which assumes 40% fuel savings due to aircraft and operational efficiency improvements.
E9	p132: ”more efficient operations across passenger transport modes”
E10	IEA (2021) Table 2.4: ”Eco-driving and motorway speed limits of 100 km/h introduced”, and ”lightweighting reduces the weight of an average passenger car by 10%” in 2030
E11	Assume the same as passenger rail
E12	p132: ”more efficient operations across passenger transport modes”, including ”logistics measures in road freight, e.g. backhauling, night-time deliveries, real-time routing”. There is no quantification of this in IEA (2021) so efficiency savings are based on the operational efficiency measures listed in IEA (2017) The Future of Trucks (p58). The product of energy demands from rough energy savings estimates of each of these measures gives an aggregate saving of around 50%.
E13	p136: ”slow steaming and the use of wind-assistance technologies”. No quantification is given in IEA (2021) or IEA World Energy Outlook (2020). Instead, potential efficiency saving is estimated from DNV-GL (2018) ”cost-effective CO2 emissions-reduction potential for technical and operational measures, excluding fuel choices, is in the range 20–30%, rising to about 50–60% if the more expensive and novel technologies and solutions are included”.
E14	In IEA (2021) various mentions of using wastes for other uses. Not quantified, assumed to be 20% saving here.

Table S410: Notes about the efficiency saving values used for the IEA Net-Zero Energy by 2050 scenario, given in Table S407.

6.5 2050 Industry Accumulated Demands Example

This set of inputs is intended to represent the demand implied by current corporate and political strategies. The input data is primarily gathered from publicly available reports from relevant industry groups, considering a global perspective. Where that is not available, data is taken from regional, national, or company reports (in that order of preference), or from the IEA Net Zero by 2050 scenario (Section 6.4) where no other sources are found. The tables use short names for the activities and processes, which each have a longer description, given in Section 6.8.

6.5.1 2050 Accumulated Demands delivery process shares (α)

Table S411: Data used as the input activity delivery process shares for the IEA Net-Zero Energy by 2050 scenario.

Activity	Process Share	Process	TechShare2050 Note	TechShare2050 Source
NonWoodBiomass	26%	Residues	Q1	P1
NonWoodBiomass	20%	Pasture	Q1	P1
NonWoodBiomass	54%	ElecLowFertiliser	Q1	P1
RawFood	9%	NonRuminantMeat	Q2	P2
RawFood	83%	PlantFoodLowMethaneRice	Q3	P2
RawFood	8%	RuminantFeedAdditives	Q4	P2
Wood	90%	Elec	Q5	P3, P4
Wood	10%	NETs	Q5	P3, P4
Appliances	100%	Elec	Q6	N/A
Cooking	15%	Bio	Q7	P3, P5
Cooking	80%	Elec	Q7	P3, P5
Cooking	5%	Gas	Q7	P3, P5
Cooling	100%	Elec	Q6	N/A
Lighting	100%	Elec	Q6	N/A
SpaceHeat	20%	Bio	Q8	P5
SpaceHeat	5%	Elec	Q9	P5
SpaceHeat	0%	Gas	Q10	P5
SpaceHeat	75%	HeatPump	Q11	P5
WaterHeating	8%	Bio	Q12	P5
WaterHeating	4%	Elec	Q12	P5
WaterHeating	0%	Gas	Q12	P5
WaterHeating	2%	H2Boiler	Q12	P5
WaterHeating	85%	HeatPump	Q12	P5
Electricity	100%	Distribution	Q6	N/A
GeneratedElectricity	1%	BECC	Q13	P5
GeneratedElectricity	4%	Bio	Q13	P5
GeneratedElectricity	1%	CoalCCS	Q13	P5
GeneratedElectricity	0%	Gas	Q13	P5
GeneratedElectricity	1%	GasCCS	Q13	P5
GeneratedElectricity	93%	NonEmitting	Q13	P5
Ammonia	70%	Elec	Q14	P6
Ammonia	3%	SMR	Q14	P6
Ammonia	27%	SMRCCS	Q14	P6
BioFuel	79%	AdvBioFuel	Q15	P5
BioFuel	7%	BioDiesel	Q15	P5
BioFuel	14%	BioEthanol	Q15	P5
HVCs	12%	BioEth	Q16	P5
HVCs	70%	CCS	Q16	P5
HVCs	12%	MTOA	Q16	P5
HVCs	6%	NET	Q16	P5
Hydrogen	19%	ATRCCS	Q17	P5
Hydrogen	62%	Elec	Q17	P5

(continued)

Activity	Process Share	Process	TechShare2050 Note	TechShare2050 Source
Hydrogen	19%	SMRCCS	Q17	P5
Methane	25%	Biogas	Q18	P5
Methane	62%	FF	Q18	P5
Methane	12%	SynDAC	Q18	P5
Methanol	25%	BioGas	Q19	P5
Methanol	25%	CCS	Q19	P5
Methanol	50%	FromH2	Q19	P5
Oil	100%	FF	Q6	N/A
OtherPetChem	100%	NET	Q6	N/A
Plastics	35%	CCS	Q20	P5
Plastics	25%	Elec	Q20	P5
Plastics	5%	NET	Q20	P5
Plastics	35%	RecycleElec	Q21	P5
Synfuel	100%	FT	Q6	N/A
Urea	100%	Elec	Q22	P7
Aluminium	44%	InertAnodes	Q23	P8, P9
Aluminium	1%	NET	Q23	P8, P9
Aluminium	55%	ScrapElec	Q23	P8, P9
Cement	20%	Bio	Q24	P10
Cement	23%	BioCCS	Q24	P10
Cement	52%	CCS	Q24	P10
Cement	5%	H2	Q25	P10
Construction	60%	Elec	Q26	P11
Construction	10%	H2Power	Q26	P11
Construction	30%	NET	Q26	P11
Food	71%	BioF	Q27	P5
Food	24%	Elec	Q27	P5
Food	5%	NET	Q27	P5
Glass	65%	Elec	Q28	P12
Glass	35%	NET	Q28	P12
OtherIndustry	95%	Elec	Q27	P5
OtherIndustry	5%	NET	Q27	P5
Paper	95%	Elec	Q27	P5
Paper	5%	NET	Q27	P5
Steel	8%	BOF	Q29	P13
Steel	13%	CCS	Q29	P13
Steel	40%	EAF	Q29	P13
Steel	22%	HDRI	Q29	P13
Steel	17%	MethaneDRI	Q29	P13
Textiles	50%	Elec	Q30	P14
Textiles	50%	NET	Q30	P14
ExtractedOilGas	36%	Elec	Q31	P5
ExtractedOilGas	64%	ElecNoFugitive	Q32	P15
Coal	27%	Elec	Q33	P16, P17
Coal	33%	ElecNoFugitive	Q33	P16, P17
Coal	18%	H2	Q33	P16, P17
Coal	22%	H2NoFugitive	Q33	P16, P17
MinedMetalsMinerals	60%	Elec	Q34	P17
MinedMetalsMinerals	40%	H2	Q34	P17
CO2Product	100%	NetZero	Q35	N/A
NetEmissions	67%	BECC	Q36	P5
NetEmissions	33%	DAC	Q36	P5
Aviation	38%	Bio	Q37	P13
Aviation	2%	ElecT	Q37	P13
Aviation	23%	LH2	Q37	P13

(continued)

Activity	Process Share	Process	TechShare2050 Note	TechShare2050 Source
Aviation	38%	PtL	Q37	P13
BusUse	79%	BEV	Q38	P5
BusUse	5%	BioICE	Q38	P5
BusUse	5%	FFICE	Q38	P5
BusUse	5%	HFCEV	Q38	P5
BusUse	5%	SynICE	Q38	P5
CarUse	86%	BEV	Q39	P5
CarUse	5%	FFICE	Q40	P5
CarUse	9%	HFCEV	Q41	P5
RailFreight	1%	BioICE	Q42	P5
RailFreight	92%	Elec	Q42	P5
RailFreight	7%	FFICE	Q42	P5
RailP	1%	BioICE	Q42	P5
RailP	92%	Elec	Q42	P5
RailP	2%	FFICE	Q42	P5
RailP	5%	HFCEV	Q42	P5
RoadFreight	60%	BEV	Q43	P18
RoadFreight	10%	CEV	Q43	P18
RoadFreight	30%	HFCEV	Q43	P18
Shipping	43%	Ammonia	Q44	P5
Shipping	20%	BioICE	Q44	P5
Shipping	16%	FFICE	Q44	P5
Shipping	20%	HFCEV	Q44	P5
Shipping	1%	SynICE	Q44	P5
WasteSolid	20%	Feedstock		
WasteSolid	5%	Incineration		
WasteSolid	20%	MethaneUse		
WasteSolid	55%	Recycling	Q45	P5, P19
Wastewater	100%	BestPractice	Q46	N/A

Table S412: Sources for for the Accumulated Demands run, given in Table S411.

TechShare2050 Source Key	TechShare2050 Source
P1	ZERs SI Part 1 - Analysis Framework Assumptions and Supply Estimations
P2	FAO (2018) The future of food and agriculture – Alternative pathways to 2050; https://www.fao.org/global-perspectives-studies/resources/detail/en/c/1157074/
P3	BEIS. (2021). Net Zero Strategy: Build Back Greener (Issue October). https://www.gov.uk/government/publications/net-zero-strategy
P4	Committee on Climate Change. (2019). Net Zero Technical Report. May, 19–292. https://www.theccc.org.uk/publication/net-zero-technical-report/
P5	IEA. (2021). Net Zero by 2050: A Roadmap for the Global Energy Sector. https://www.iea.org/reports/net-zero-by-2050
P6	Mission Possible Partnership (2022) Making Net-Zero Ammonia Possible; https://missionpossiblepartnership.org/wp-content/uploads/2022/09/Making-1.5-Aligned-Ammonia-possible.pdf
P7	Fertilizers Europe. (2023). Decarbonising Fertilizers by 2050 The industry's roadmap towards climate neutrality. https://www.fertilizerseurope.com/wp-content/uploads/2023/11/DEF__2023__Decarbonisation__Roadmap__digital.pdf
P8	Mission Possible Partnership (2023) Making Net-Zero Aluminium Possible, https://missionpossiblepartnership.org/wp-content/uploads/2023/04/Making-1.5-Aligned-Aluminium-possible.pdf
P9	IEA. (2022). Aluminium, IEA, Paris https://www.iea.org/reports/aluminium, License: CC BY 4.0
P10	Global Cement and Concrete Association (GCCA). (2023). Getting to Net Zero. https://gccassociation.org/concretefuture/getting-to-net-zero/
P11	Bellona.org. (2023). Database: Emission-free Construction Equipment (by manufacturer). https://bellona.org/database-emission-free-construction-equipment-by-manufacturer
P12	British Glass (2021) Glass Sector Net Zero Strategy; www.britglass.org.uk/knowledge-base/resources-and-publications/glass-sector-net-zero-strategy-2050
P13	Mission Possible Partnership. (2022). Making Net-Zero Aviation Possible. https://missionpossiblepartnership.org/wp-content/uploads/2023/01/Making-Net-Zero-Aviation-possible.pdf
P14	Ellen MacArthur Foundation (2017) A new textiles economy: Redesigning fashion's future; http://www.ellenmacarthurfoundation.org/publications
P15	McKinsey & Company. (2020). The future of oil and gas is now: How companies can decarbonize (Issue January). https://www.mckinsey.com/industries/oil-and-gas/our-insights/the-future-is-now-how-oil-and-gas-companies-can-decarbonize
P16	IEA (2023), Global Methane Tracker 2023, IEA, Paris https://www.iea.org/reports/global-methane-tracker-2023, License: CC BY 4.0
P17	McKinsey & Company. (2021). Creating the zero-carbon mine. https://www.mckinsey.com/industries/metals-and-mining/our-insights/creating-the-zero-carbon-mine
P18	Mission Possible Partnership. (2022). Making Zero-Emissions Trucking Possible. https://missionpossiblepartnership.org/wp-content/uploads/2022/11/Making-Zero-Emissions-Trucking-Possible.pdf
P19	UMAS & the Getting to Zero Coalition (2021) A Strategy for the Transition to Zero-Emission Shipping; www.globalmaritimeforum.org/content/2021/10/A-Strategy-for-the-Transition-to-Zero-Emission-Shipping.pdf

Table S413: Notes about delivery process shares for the Accumulated Demands run, given in Table S411.

TechShare2050 Note Key	TechShare2050 Note
Q1	Based on the distribution estimated for maximum availability of Biomass in 2050. Best case strategy assumed in absence of other info.
Q2	94% compared to 2012 consumption of animal products, based on Table 4.4

(continued)

TechShare2050 Note Key	TechShare2050 Note
Q3	123% increase in fruit and vegetable supply compared to 2012, based on Table 4.4. Assume all rice farmed using methods to reduce methane production.
Q4	94% compared to 2012 consumption of animal products, based on Table 4.4. Assume all ruminent products are based on farming with feed additives.
Q5	BEIS (2021): 100% ”low carbon farming practices as a percentage of total farmers”; CCC (2019) assume 90% reduction in powertrain emissions by replacing fuels with hydrogen and electricity (although only 75% update by farmers of ”on-farm practices”).
Q6	Only one delivery process
Q7	Distribution of cooking technologies for the emerging market is given in IEA (2021) Fig 4.11. In absence of other data, this is assumed to be 50 % of demand in 2050 with remaining 50% electrified, as suggested in UK strategy documents
Q8	IEA (2021) p 145: ”Bioenergy meets … more than 20% [of space heating energy needs] by 2050”
Q9	Direct electrical space heating is estimated to be 5%, based on IEA (2021) p 145: ”homes using electricity for heating rise … [to] 55% in 2050”, and p20: ”50% of [buildings] heating demand met by heat pumps” in 2045
Q10	IEA (2021) p 145: ”homes heated by natural gas falling… to less than 0.5% in 2050”
Q11	In the absence of a district heating process, it is included under heat pumps. 55% space heating is assumed to come from heat pumps based on p20: ”50% of [buildings] heating demand met by heat pumps” in 2045. 20% assigned to district heating based on p145: ”District heat networks … provide more than 20% of final energy demand for space heating”
Q12	IEA (2021) Fig 3.29. Heat pump process also include the share of heating from district heat and solar thermal (in the absence of more suitable processes). The ;other’ category is assigned to direct electrical.
Q13	IEA (2021) Table A.3 pg 198
Q14	Based on the Mission Possible Partnership (2022) LC Trajectory (”ambitious but realistic” net-zero by 2050 at the lowest cost), Exhibit 2.5, p56
Q15	Estimated from IEA (2021) Fig 3.7 p 108. NOTE: the model does not currently include a process for production with CCUS but this makes up 50% in the NZE report.
Q16	Estimated from IEA (2021) Fig 3.19, p126, considering all fuels and feedstocks toegther
Q17	IEA (2021) p109: ”water electrolysis accounts for more than 60% of global production, and natural gas in combination with CCUS for almost 40%”
Q18	IEA (2021) Table A.2 pg 196
Q19	Methanol is included as a low-carbon fuel so it is assumed here that it must be made from biogas or hydrogen, or with CCS. The share is based on IEA (2021) Fig 3.19, p126.
Q20	Little direct infomation - assumed to follow IEA (2021) strategy of primary chemicals (p126)
Q21	IEA (2021) Table 3.3 p131
Q22	”By 2050, European fertilizer production will be climate-neutral”
Q23	From MPP (2023) p40: 245% growth in secondary production, 4% growth in primary production; 2018 secondary production is 34% (IEA (2022), Aluminium) meaning total growth of 152%. 98% primary production uses inert anodes from MPP (2023) p 43.
Q24	Alternative fuel use in the GCC scenario increases to 43% in 2050 (from current 6%). CCS accounts for around 3/4 of emissions reductions after resource efficiency. NOTE: Recarbonation is not currently accounted for in the model but the GCC scenario assumes 6% of emssions reductions from recarbonation).
Q25	”Innovations such as use of hydrogen and kiln electrification are forecast to play a small role from 2040.”
Q26	No detailed information on construction in IEA (2021). Majority of low carbon construction equipment listed on the Bellona database (2023) are electically driven. Assume that ~30% of legacy equipment is still used by 2050.
Q27	IEA (2021) p20: More than 90% of industrial production is low-emissions. Assume both electrified and biofuel powered since both make up the final distribution of energy use in industry (biomass contributes 16% fuel compared to 46% electricity)

(continued)

TechShare2050 Note Key	TechShare2050 Note
Q28	In the absence of a global industry strategy for the glass sector, the proposal published by the British Glass Industry. From Fig 11, 56% fuel switch to electricity and 9% fuel switch to hydrogen are approximated as electrificiation (in the absence of a process for hydrogen-fuelled production). CCS, alternative raw materials and increased cullet use are assumed to provide 7%, 2% & 3% process emissions savings but are not accounted for here.
Q29	Carbon Cost Scenario used since it is compatible with 1.5 deg budget. Technology split is based on exhibit 2.6 (p45).
Q30	The textile industry aims to ”move to renewable inputs” but this is not quantified. Here it is assumed that 50% of production energy is electrified.
Q31	IEA (2021) p104: ”The end of all flaring … and significant electrification of upstream operations”
Q32	McKinsey (2020) suggest around 85% of fugitive emissions are from upstream (including flaring and venting). These are easier to manage by reducing flaring & using leak detection and repair systems. Downstream leaks are more disperse and so harder to monitor and fix. Assume around 70% of upstream emissions are prevented and around 30% of downstream emissions.
Q33	The IEA (2023) estimate that it's possible to reduce over half of coal mine methane with existing technologies. Note that energy supply from recovered coal mine methane is not accounted for in the model (although this is only one of three key approaches mentioned in the text). Assume 55% fugitive emissions are avoided. There is no clear strategy for zero-emissions trucking and industry but McKinsey (2021) suggest electric vehicles are a front runner. Assume 60% electrified processes and 40% hydrogen powered.
Q34	There is no clear strategy for zero-emissions mining but McKinsey (2021) suggest electric vehicles are a front runner. Assume 60% electrified processes and 40% hydrogen powered.
Q35	Assume all residual CO2 is stored
Q36	Follows distribution of IEA (2021) 1.9 Gt removed by BECC and DAC, of which 633 MtCO2 is DAC (p 55 and 199)
Q37	Split is based on energy shares given on p16. SAFs split 50:50 between PtL and biofuel based on the report's PRU scenario ”data explorer” online at https://dash-mpp.plotly.host/mpp-aviation-net-zero-explorer/
Q38	IEA (2021) assumes 79% of bus stock in 2050 is battery electric (table 2.5, p72). Residual assumed to be equally distributed amongst other modes.
Q39	IEA (2021) assumes 86% of car stock in 2050 is battery electric from Table 2.5, p72. BEV share of sales is 90% (Fig 3.23)
Q40	Residual share
Q41	Around 10% sales from IEA (2021) Fig 3.23, p134 (compared to 90% electric)
Q42	IEA (2021) Fig 3.25, p 137. No distinction between passenger and freight rail in the figure so this is assigned based on available delivery processes.
Q43	Process split is based on the MPP (2022) zero-emissions scenario and estimated from energy consumption (p51). CEVs are assumed to take a small proportion of electric powered vehicles since the report writes: ”Innovative charging technologies (e.g., catenaries...are likely to be used in discrete contexts [but] they will not represent the backbone of the system by 2050” (p25).
Q44	Fuel split is as suggested by the IEA (Fig 3.25, p 137) since it is not given by UMAS & the Getting to Zero Coalition (2021)
Q45	Based on 2050 target rate for recycling
Q46	Best case strategy assumed in absence of other info.

6.5.2 2050 Accumulated Demands final activity rates ($q_{Apparent}$)

Table S414: Data used as the input activity demand for the industry-led proposals scenario. *Relative demand* is the activity demand in 2050 compared to 2018 values (Table S415). *Efficiency saving* is used to account for conversion device, passive system, and operational efficiency improvements which are not accounted for elsewhere. This framework for accounting for efficiency savings is described in SI Document 2A (Mathematical Framework). Notes and sources referred to in this table are given in Table S416 and Table S417.

Activity	Relative Demand	Actual Demand	Efficiency Saving	Process Rate	Units	Note	Reference
Appliances	250%	33	46%	18	EJ used for appliances	N1	R2
Cooking	250%	77	46%	41	EJ used for cooking	N1	R2
Cooling	291%	12	50%	5.9	EJ used for cooling	N1	R2
Lighting	250%	22	46%	12	EJ used for lighting	N1	R2
SpaceHeat	250%	98	50%	48	EJ used for space heating	N1	R2
WaterHeating	250%	63	28%	45	EJ used for heating water	N1	R2
OtherPetChem	300%	0.75	N/A	0.75	Gt petrochemicals, not otherwise accounted	N3	R5
Plastics	234%	0.83	30%	0.58	Gt plastics	N4	R6
Aluminium	152%	0.15	0%	0.15	Gt aluminium ingot	N5	R7, R8
Cement	109%	4.4	11%	3.9	Gt cement	N6	N/A
Construction	N/A	N/A	N/A	5.2	Gt 100% cement + 50% steel	N7	R9, R12
Food	127%	10	N/A	10	10^15 kcal food supply	N8	R1
Glass	108%	0.18	N/A	0.18	Gt glass	N9	R10
OtherIndustry	128%	0.31	N/A	0.31	Billion Units: (Vehicles + Smartphones/10)	N10	R1
Paper	145%	0.58	N/A	0.58	Gt paper and board produced	N11	N/A
Steel	140%	2.5	0%	2.5	Gt steel	N12	R12
Textiles	301%	0.29	50%	0.14	Gt textiles	N13	R13
MinedMetalsMinerals	N/A	N/A	N/A	58	Gt run-of-mine metals and minerals extracted	N14	N/A
Aviation	216%	18	40%	11	10^12 revenue passenger km	N15	R15
BusUse	100%	11	0%	11	10^12 passenger km	N16	R2
CarUse	160%	24	0%	24	10^12 vehicle km	N/A	R2
RailFreight	200%	27	0%	27	x10^12 t km	N/A	R2
RailP	200%	8.8	0%	8.8	10^12 passenger km	N/A	R2
RoadFreight	181%	48	20%	39	x10^12 t km	N20	R16
Shipping	250%	270	30%	190	x10^12 t km	N21	R17
WasteSolid	166%	-3.4	0%	-3.4	Gt Solid Waste	N/A	R18
Wastewater	127%	-460	N/A	-460	Gt Wastewater	N22	R1

Table S416: Notes for the final activity rates used for the Accumulated Demands run, given in Table S414.

2050 Demand Key	2050 Demand Note
N1	In the absence of a global industry-led strategy for energy use in buildings, values are based on IEA. (2021). Net Zero by 2050: A Roadmap for the Global Energy Sector. https://www.iea.org/reports/net-zero-by-2050.
N2	low cost scenario is about 550 Mt and fastest abatement scenario is about 800 Mt (exhibit 2.5 p56)
N3	Solvents may need to scale 200-fold to meet the demand for CCS (Energy Transitions Commission (2023) p37). Assuming these make up 1% of current 'otherpetrochemicals', total scale up would be 300%.
N4	p 19: although overall demand increases by over 100% to 2050, 30% demand is reduced by value chain reductions, substitution and reuse
N5	From MPP (2023) p40: 245% growth in secondary production, 4% growth in primary production; 2018 secondary production is 34% (IEA (2022), Aluminium) meaning total growth of 152%
N6	140% increase in demand from 2020 (societal need) but assumed 22% demand reduction (equal to emissions reductions from "efficiency in design and construction"). 11% emissions savings from "efficiency in concrete production".
N7	Sum of 50% steel and 100% cement
N8	Table 4.2 gives calorific supply TSS (Towards Sustainability Scenario) of 2938 kcal/person/day, population predicted to be 9.7 billion in 2050 according to United Nations (p8)
N9	Demand for flat glass in 2050 estimated from Westbroek et al. (2021) global dynamic material flow analysis of glass (Fig 3). Container glass demand assumed to be unchanged.
N10	Assume increase in other industry scales with population growth, as predicted by United Nations: population increase from 7.6 billion in 2018 to 9.7 b in 2050 (p8).
N11	Projected demand increase based on extrapolation of IEA Sustainable Development Scenario
N12	Carbon Cost Scenario used as compatible with 1.5 deg budget. Production grows to 2.5 Gt/yr by 2050 (Exhibit 2.6, p45). Material efficiency could decrease production demand by 40% to 1.5 Gt/yr (Exhibit D, p13) but these are not included in the scenario.
N13	Appendix B3: CAGR of 3.5% for fibre demand. Based on text on p36-7, there is potential for increased utilisation and recycling, approximated here to account for a 50% overall reduction in demand for fibre production.
N14	9.4 Gt and 44 Gt extraction of metals and non-metallic minerals in 2018 (UN IRP database). Assume metals demand increases by 50% (following trend of alumnium and steel) but non-metallic minerals stay stable (following trend for cement).
N15	Relative demand from p51; p37 for efficiency savings - "the global aircraft fleet could be about 40% more fuel efficient in 2050 than in 2019"
N16	In the absence of a global industry-led strategy specifically for passenger land transport, values are based on IEA. (2021). Net Zero by 2050: A Roadmap for the Global Energy Sector. https://www.iea.org/reports/net-zero-by-2050.
N17	In the absence of a global industry-led strategy specifically for passenger land transport, values are based on IEA. (2021). Net Zero by 2050: A Roadmap for the Global Energy Sector. https://www.iea.org/reports/net-zero-by-2050. From Table A.5 (p200) vehicle km driven increases by 60% compared to 2019 levels.
N18	In the absence of a global industry-led strategy specifically for rail frieght transport, values are based on IEA. (2021). Net Zero by 2050: A Roadmap for the Global Energy Sector. https://www.iea.org/reports/net-zero-by-2050. Global transport final consumption for rail is approx. twice as large in 2050 as 2020 based on Fig 3.22 p133.
N19	In the absence of a global industry-led strategy specifically for passenger land transport, values are based on IEA. (2021). Net Zero by 2050: A Roadmap for the Global Energy Sector. https://www.iea.org/reports/net-zero-by-2050. Global transport final consumption for rail is approx. twice as large in 2050 as 2020 based on Fig 3.22 p133.
N20	Demand is estimated from the emissions trajectory in the 'do nothing' scenario (p42). From p29 activity efficiency measures (mode shift to rail, supply chain and logisical efficiency) could reduce demand by 20%.
N21	Shipping projected ton-miles increase from Fig 2 ; "the magnitude of further efficiency-led carbon intensity reduction (as a fleet average) is between 25% and 30% from current levels" p20
N22	p8 - United Nations prediction: population of 9.7 billion in 2050 is a 27% increase on 2018 population (from FAOSTAT)

Table S417: Sources used to assign the final activity rates used for the Accumulated Demands run, given in Table S414.

2050 Demand Reference Key	2050 Demand Reference
R1	FAO (2018) The future of food and agriculture – Alternative pathways to 2050; https://www.fao.org/global-perspectives-studies/resources/detail/en/c/1157074/
R2	IEA. (2021). Net Zero by 2050: A Roadmap for the Global Energy Sector. https://www.iea.org/reports/net-zero-by-2050
R3	Mission Possible Partnership (2022) Making Net-Zero Ammonia Possible; https://missionpossiblepartnership.org/wp-content/uploads/2022/09/Making-1.5-Aligned-Ammonia-possible.pdf
R4	Saygin, D., Gielen, D., & Moutinho, F. (2021). Zero-Emission Pathway for the Global Chemical and Petrochemical Sector. https://doi.org/10.3390/en14133772
R5	Energy Transitions Commission (ETC). (2023). Material and Resource Requirements for the Energy Transition. July. https://www.energy-transitions.org/publications/material-and-resource-energy-transition
R6	Energy Transitions Commission. (2019). Mission Possible sectoral focus: plastics. https://www.energy-transitions.org/publications/mission-possible-sectoral-focus-plastics/
R7	Mission Possible Partnership. (2023). Making net-zero aluminium possible. https://www.missionpossiblepartnership.org/action-sectors/aluminium/
R8	IEA. (2022). Aluminium, IEA, Paris https://www.iea.org/reports/aluminium, License: CC BY 4.0
R9	GCCA. (n.d.). Getting to Net Zero. Retrieved September 10, 2024, from https://gccassociation.org/concretefuture/getting-to-net-zero/
R10	Westbroek, C. D., Bitting, J., Craglia, M., Azevedo, J. M. C., & Cullen, J. M. (2021). Global material flow analysis of glass: From raw materials to end of life. Journal of Industrial Ecology, 25(2), 333–343. https://doi.org/10.1111/jiec.13112
R11	IEA. (2020). Global paper and paperboard production in the Sustainable Development Scenario, 2010-2030. https://www.iea.org/data-and-statistics/charts/global-paper-and-paperboard-production-in-the-sustainable-development-scenario-2010-2030
R12	Mission Possible Partnership (2022) Making Net-Zero Steel Possible; https://missionpossiblepartnership.org/wp-content/uploads/2022/09/Making-Net-Zero-Steel-possible.pdf
R13	Ellen MacArthur Foundation (2017) A new textiles economy: Redesigning fashion's future; http://www.ellenmacarthurfoundation.org/publications
R14	Shell (2021) Shell Energy Transition Strategy; https://www.shell.com/energy-and-innovation/the-energy-future/shell-energy-transition-strategy
R15	Mission Possible Partnership. (2022). Making Net-Zero Aviation Possible. https://missionpossiblepartnership.org/wp-content/uploads/2023/01/Making-Net-Zero-Aviation-possible.pdf
R16	Mission Possible Partnership. (2022). Making Zero-Emissions Trucking Possible. https://missionpossiblepartnership.org/wp-content/uploads/2022/11/Making-Zero-Emissions-Trucking-Possible.pdf
R17	UMAS & the Getting to Zero Coalition (2021) A Strategy for the Transition to Zero-Emission Shipping; www.globalmaritimeforum.org/content/2021/10/A-Strategy-for-the-Transition-to-Zero-Emission-Shipping.pdf
R18	Kaza, S., Yao, L. C., Bhada-Tata, P. & Van Woerden, F. What a Waste 2.0: A Global Snapshot of Solid Waste Management to 2050. doi:10.1596/978-1-4648-1329-0.

6.6 UK Government Strategy

This scenario is based on published UK government strategy, published as of May 2023. The starting point for the scenario is the UK: Build Back Greener Strategy document (BEIS, 2021b) but most of the data is not given explicitly in this report. The numbers used here are instead based on a range of documents published by government departments, referenced in the Strategy document. In general, even technical reports from specific departments generally do not publish explicit data on the quantity of services they expect to supply, nor how they intend to provide them. For this model scenario these inputs have been deduced from the text, or data on attributed emissions abatement. Where there is not sufficient information available in the UK government strategy documents, the inputs are instead based on the "Further Ambition" scenario compiled by the Climate Change Committee (CCC) for their Net-Zero Advice Report (2019) to the UK Government. This scenario is developed to include "measures that will definitely be needed for a net-zero emissions target" (Committee on Climate Change, 2019).

There are many examples (particularly for industry) where either there is no obvious strategy, or where production is dominantly overseas. In these cases, the values are based on the IEA Net Zero run (Section 6.4).

The tables use short names for the activities and processes, which each have a longer description, given in Section 6.8.

6.6.1 UK Government Strategy final activity rates ($q_{Apparent}$)

The UK Government Strategy final activity run uses the global activity demand data for the accumulated demands run (Section 6.5) to make the run comparable with other global model runs.

6.6.2 UK Government Strategy delivery process shares (α)

The following tables list the delivery process shares used for the UK Government Strategy run, and their justifications.

Table S418: Data used as the input activity delivery process shares for the UK Government Strategy run. Sources and notes are listed in Table S419 and Table S420.

Activity	Process Share	Process	UK_2050 Source	UK_2050 Note
NonWoodBiomass	26%	Residues	U1	K1
	20%	Pasture	U1	K1
	54%	ElecLowFertiliser	U2, U3	K2
RawFood	16%	NonRuminantMeat	U2, U3, U4	K3
	73%	PlantFoodLowMethaneRice	U2, U3, U4	K3
	12%	RuminantFeedAdditives	U2, U3, U4	K3
Wood	90%	Elec	U2, U3	K2
	10%	NETs	U2, U3	K2
Appliances	100%	Elec	U5	N/A
Cooking	100%	Elec	U3	K4
Cooling	100%	Elec	U5	N/A
Lighting	100%	Elec	U5	N/A
SpaceHeat	1%	Elec	U2, U3	K5
	7%	Gas	U3	K6
	14%	H2Boiler	U2	K7
	78%	HeatPump	U2, U3	K8

(continued)

Activity	Process Share	Process	UK_2050 Source	UK_2050 Note
WaterHeating	1%	Elec	U2, U3	K9
	10%	Gas	U2, U3	K9
	20%	H2Boiler	U2, U3	K9
	69%	HeatPump	U2, U3	K9
Electricity	100%	Distribution	U5	N/A
GeneratedElectricity	6%	BECC	U2, U3	K10
	2%	Bio	U2, U3	K11
	24%	GasCCS	U2, U3	K11
	68%	NonEmitting	U2, U3	K11
Ammonia	30%	Elec	See Hydrogen	K12
	70%	SMRCCS	See Hydrogen	K12
BioFuel	79%	AdvBioFuel	U6	K13
	7%	BioDiesel	U6	K13
	14%	BioEthanol	U6	K13
HVCs	12%	BioEth	U6	K14
	32%	CCS	U6	K14
	50%	MTOA	U6	K14
	6%	NET	U6	K14
Hydrogen	70%	Elec	U7	K15
	30%	SMRCCS	U7	K15
Methane	5%	Biogas	U8	K16
	90%	FF	U2	K17
	5%	SynDAC	U2	K17
Methanol	20%	BioGas	U2	K17
	60%	CCS	U2	K17
	20%	FromH2	U2	K17
Oil	100%	FF	U5	N/A
OtherPetChem	100%	NET	U5	N/A
Plastics	35%	CCS	U9, U6	K18
	25%	Elec	U9, U6	K18
	5%	NET	U9, U6	K18
	35%	RecycleElec	U9, U6	K18
Synfuel	100%	FT	U5	N/A
Urea	90%	Elec	U6	K19
	10%	Gas	U6	K19
Aluminium	40%	InertAnodes	U10	K19
	4%	NET	U10	K19
	56%	ScrapElec	U10	K19
	20%	BioCCS	U11	K20

(continued)

Activity	Process Share	Process	UK_2050 Source	UK_2050 Note
Cement	80%	CCS	U11	K20
Construction	75%	Elec	U12	K21
	15%	H2Power	U12	K21
	10%	NET	U12	K21
Food	20%	BioF	U6	K19
	75%	Elec	U6	K19
	5%	NET	U6	K19
Glass	95%	Elec	U6	K19
	5%	NET	U6	K19
OtherIndustry	95%	Elec	U6	K19
	5%	NET	U6	K19
Paper	95%	Elec	U6	K19
	5%	NET	U6	K19
Steel	29%	CCS	U6	K19
	46%	EAF	U6	K19
	23%	HDRI	U6	K19
	3%	MethaneDRI	U6	K19
Textiles	95%	Elec	U6	K19
	5%	NET	U6	K19
ExtractedOilGas	36%	Elec	U3, U13	K22
	64%	ElecNoFugitive	U3, U13	K22
Coal	27%	Elec	U14, U15	K23
	33%	ElecNoFugitive	U14, U15	K23
	18%	H2	U14, U15	K23
	22%	H2NoFugitive	U14, U15	K23
MinedMetalsMinerals	60%	Elec	U15	K24
	40%	H2	U15	K24
CO2Product	100%	NetZero	N/A	K25
NetEmissions	94%	BECC	U2	K26
	6%	DAC	U2	K26
Aviation	25%	Bio	U17, U18	K27
	6%	ElecT	U17, U18	K27
	23%	JetA	U17, U18	K27
	21%	LH2	U17, U18	K27
	25%	PtL	U17, U18	K27
BusUse	61%	BEV	U19	K28
	39%	FFICE	U19	K28
CarUse	98%	BEV	U20	
	2%	FFICE	U20	

(continued)

Activity	Process Share	Process	UK_2050 Source	UK_2050 Note
RailFreight	90%	Elec	U21	K30
	10%	FFICE	U21	K30
RailP	96%	Elec	U21	K31
	4%	HFCEV	U21	K31
RoadFreight	96%	BEV	U20	K32
	4%	FFICE	U20	K32
Shipping	24%	Ammonia	U2	K33
	24%	BioICE	U2	K33
	3%	FFICE	U2	K33
	24%	HFCEV	U2	K33
	24%	SynICE	U2	K33
WasteSolid	16%	Feedstock	U3	K34
	3%	Incineration	U2, U22, U23	K35
	16%	MethaneUse	U2, U22, U23	
	65%	Recycling	U2, U22, U23	
Wastewater	20%	BestPractice	U2, U3	K36
	80%	NET	U2, U3	K36

Table S421: Sources used to assign the delivery process shares used for the UK Government Strategy run, given in Table S422.

UK_2050 Tech Share Reference Key	UK_2050 Tech Share Reference
U1	ZERs SI Part 1 - Analysis Framework Assumptions and Supply Estimations
U2	BEIS. (2021). Net Zero Strategy: Build Back Greener (Issue October). https://www.gov.uk/government/publications/net-zero-strategy
U3	Committee on Climate Change. (2019). Net Zero Technical Report. May, 19–292. https://www.theccc.org.uk/publication/net-zero-technical-report/
U4	FAO. (2022). FAOSTAT Food Balances. Licence: CC BY-NC-SA 3.0 IGO. https://www.fao.org/faostat/en/?#data/FBS
U5	Only one delivery process
U6	IEA. (2021). Net Zero by 2050: A Roadmap for the Global Energy Sector. https://www.iea.org/reports/net-zero-by-2050
U7	Department for Energy Security and Net Zero. (2021). UK Hydrogen Strategy. https://www.gov.uk/government/publications/uk-hydrogen-strategy
U8	Anthesis Consulting Group, & E4 Tech. (2017). Review of Bioenergy Potential. https://cadentgas.com/getmedia/e3a0df47-7c1e-4c7a-98e1-8013adc649b3/Cadent-Bioenergy-Market-Review-SUMMARY-Report-FINAL-amended_1.pdf
U9	Drewniok, M., Cullen, J., & Serrenho, A. C. (2020). THE ‘P’WORD–Plastic in the UK: practical and pervasive... but problematic. https://doi.org/10.17863/CAM.58737
U10	IEA. (2023). Net Zero Roadmap: A Global Pathway to Keep the 1.5 °C Goal in Reach. https://www.iea.org/reports/net-zero-roadmap-a-global-pathway-to-keep-the-15-0c-goal-in-reach
U11	BEIS. (2021). Industrial Decarbonisation Strategy. https://www.gov.uk/government/publications/net-zero-strategy
U12	Bellona.org. (2023). Database: Emission-free Construction Equipment (by manufacturer). https://bellona.org/database-emission-free-construction-equipment-by-manufacturer
U13	McKinsey & Company. (2020). The future of oil and gas is now: How companies can decarbonize (Issue January). https://www.mckinsey.com/industries/oil-and-gas/our-insights/the-future-is-now-how-oil-and-gas-companies-can-decarbonize
U14	IEA (2023), Global Methane Tracker 2023, IEA, Paris https://www.iea.org/reports/global-methane-tracker-2023, License: CC BY 4.0
U15	McKinsey & Company. (2021). Creating the zero-carbon mine. https://www.mckinsey.com/industries/metals-and-mining/our-insights/creating-the-zero-carbon-mine
U16	BEIS. (2021). Net Zero Strategy: Build Back Greener (Issue October). https://www.gov.uk/government/publications/net-zero-strategy
U17	Department for Transport. (2022). Jet Zero strategy: delivering net zero aviation by 2050. https://www.gov.uk/government/publications/jet-zero-strategy-delivering-net-zero-aviation-by-2050
U18	Graver, B., Zhang, K., & Rutherford, D. (2019). CO2 emissions from commercial aviation, 2018. https://theicct.org/publication/co2-emissions-from-commercial-aviation-2018/
U19	Department for Energy Security and Net Zero. (2023). Net Zero Growth Plan and Carbon Budget Delivery Plan. https://www.gov.uk/government/publications/powering-up-britain
U20	Department for Transport. (2021). Transport decarbonisation plan. https://www.gov.uk/government/publications/transport-decarbonisation-plan
U21	Network Rail. (2020). Traction Decarbonisation Network Strategy Interim Programme Business Case. https://www.networkrail.co.uk/wp-content/uploads/2020/09/Traction-Decarbonisation-Network-Strategy-Interim-Programme-Business-Case.pdf
U22	Department for Environment Food & Rural Affairs. (2022). UK Statistics on Waste. https://www.gov.uk/government/statistics/uk-waste-data
U23	Hardy, L., & Benton, D. (2022). The Global Methane Pledge. https://green-alliance.org.uk/wp-content/uploads/2022/10/Global-methane-pledge.pdf

Table S423: Notes about delivery process shares used for the UK Government Strategy run, given in Table S422.

UK_2050 Tech Share Note Key	UK_2050 Tech Share Note
K1	Based on the distribution estimated for maximum availability of Biomass in 2050
K2	BEIS (2021): 100% "low carbon farming practices as a percentage of total farmers"; CCC (2019) assume 90% reduction in powertrain emissions by replacing fuels with hydrogen and electricity (although only 75% update by farmers of "on-farm practices").
K3	"Diet" is only mentioned once in the BEIS (2021) Net Zero Strategy Document simply to say that they may be a market opportunity for "alternative proteins" as consumer dietary trends change. CCC (2019) Further Ambition scenario assumes a 20% reduction in the consumption of beef, lamb and dairy. This is taken against the distribution of food types consumed in the UK in 2018, estimated from FAOSTAT Food Balances, with the gap met by plant based food. The lowest emissions delivery processes for this diet have been assumed, although (since not mentioned) no global reduction in rice consumption is considered.
K4	Electrification of catering. The proposal does mention hydrogen cooking may be considered for homes with hydrogen heating. It's assumed here that total energy use for hydogen cooking would be small.
K5	Derived from estimates of homes with each technology installed by 2050 according to the CCC (2018) Further Ambition scenario and BEIS (2021) Technical Annex Table 5, and the CCC (2018) scenario for industrial buildings ("half of heat demand is assumed to convert to low-carbon heat networks and the other half to heat pumps"). Around 30% energy use assumed from non-residential.
K6	10% of most expensive homes remain fossil-fuelled in CCC (2019) Further Ambition Scenario
K7	0-14 million homes converted to hydrogen by 2050 accoring to BEIS (2021) Technical Annex Table 5.
K8	Low carbon heat networks have been assigned to heat pumps in the absence of a more appropriate process.
K9	Water heating assumed to follow residential space heating since water heating is comparatively small in the non-residential sector.
K10	BEIS (2021): 100% "low carbon generation" by 2050. Assumed to be composed of non-emitting, biofuel, and CCS generation. The distribution amongst these is based on the CCC 2019 net-zero technical report (p40-41)
K11	BEIS (2021) Technical Annex Table 5: "low carbon generation" is assumed to be composed of non-emitting, biofuel, and CCS generation. The distribution amongst these is based on the CCC 2019 net-zero technical report (p40-41)
K12	Assume ammonia follows similar distribution as Hydrogen
K13	Fig 3.7 p 108. NOTE: the model does not currently include a process for production with CCUS but this makes up 50% in the NZE report.
K14	As for plastics, delivery processes for HVCs assumed to follow global distribution (the IEA scenario)
K15	Hydrogen technology proportions vary significantly between the three scenarios given by BEIS (2021). UK Hydrogen Strategy stated in 2050 "hydrogen will be supplied through a mix of [SMR] with CCUS, electrolysis ..., and [BECC]". The Hydrogen Strategy update to market in Dec 2023 stated that at least half of low carbon production would be electrolytic by 2030.
K16	UK Biomethane potential in 2050 around 100 TWh/yr
K17	Methanol is included by BEIS (2021) as a low-carbon fuel so it is assumed here that it must be made from biogas or hydrogen, or with CCS.
K18	Around 70% plastics are imported (Drewniok, Cullen, & Serrenho, 2020) so estimated from global scenario from IEA (2021)
K19	Based on global IEA scenario
K20	Estimated based on the emissions abatement curve on p147. Data chosen is for the National Networks Scenario which has greater emissions reductions.
K21	Little available information in UK documents so follows IEA global scenario: Majority of low carbon construction equipment listed on the Bellona database (2023) are electically driven. Assume that ~10% of legacy equipment is still used by 2050.

(continued)

UK_2050 Tech Share Note Key	UK_2050 Tech Share Note
K22	Little change in methane leakage in pipes but potentially ~90% reduction in flare and vent operations. Box B4.4 from CCC (2019) suggests only expected decreases in fugitive emissions are from monitoring and reducing production.
K23	The IEA (2023) estimate that it's possible to reduce over half of coal mine methane with existing technologies. Note that energy supply from recovered coal mine methane is not accounted for in the model (although this is only one of three key approaches mentioned in the text). Assume 55% fugitive emissions are avoided. There is no clear strategy for zero-emissions trucking and industry but McKinsey (2021) suggest electric vehicles are a front runner. Assume 60% electrified processes and 40% hydrogen powered.
K24	McKinsey (2021) suggest electric vehicles are a front runner. Assume 60% electrified processes and 40% hydrogen powered.
K25	Assume all residual CO2 is stored
K26	Technical Annex Table 5 , assuming all emissions are offset in the proportions given in the report. The strategy only accounts for 80 MtCO2 engineered removals in total (for the UK which is around 1% total global emissions today). The strategy also includes additional sequestration in land-sinks via tree planting and peatland. The CCC (2019) "Further Ambition", engineered removals total 54.2 MtCO2e/yr (of which 94% is BECC, 2% is DACCS, 4% is timber construction).
K27	Based on the DfT (2022) Jet Zero Strategy 'high ambition' scenario outlined in the analytical annex (p9). In the absence of other info, SAF is split between PtL and biofuel in equal shares, while "zero emission aircraft" are assumed to be electrically and hydrogen powered. Electric flight is assumed to provide the share of flights under 500km, estimated from the 2018 distribution, given by Graver et al. (2019). The residual is met by conventional kerosene flight.
K28	Department for Energy Security and Net Zero (2023) Table 7
K29	Network Rail (2020) p77
K30	For freight, around 90% of train kilometres could be operated electrically with the remaining 10% requiring either diesel or alternative traction locomotives.
K31	96% of passenger unit kilometres operated using electric traction with the remaining 4% operated using hydrogen and battery units.
K32	Assumed loads of 0.5t and 10t for vans and HGV respectively, 2018 values are based on 2022
K33	BEIS (2021) Technical Annex Table 5 gives the proportion of low carbon fuel use (97%). Low carbon fuels are distributed among non-fossil fuel options equally in the absence of other data.
K34	CCC (2019) Further Ambition Scenario includes a 20% reduction in avoidable food waste (which is around 50% of all food waste from p 192).
K35	Recycling rates are increased to 65% based on BEIS (2021) target for municipal recycling. Other shares (with biogenic waste as energy subtracted) are based on baseline data from 2018. It is assumed that all landfill reduces to account for this and has full methane capture applied in 2050. 2018 shares are mostly based on UK official data (UK Statistics on Waste published by DEFRA) but the share of landfill gas collected (share allocated to MethaneUse rather than landfill) is estimated from a report produced by The Green Alliance (2022) which suggests landfill gas capture rates were around 70% in 2020 (compared to 85% as assumed for delivery process).
K36	BEIS (2021): "Wastewater emissions will decrease due to improved treatment processes and expected data improvements" but CCC Further Ambition scenario shows only 20% reduction in wastewater handling emissions (p245).

6.7 Low Zero Emissions Resources (Low ZER) Example

The 'Low ZER' demand example is also included in the main paper as an example of a proposal which would achieve net-zero greenhouse gases within the probable supply constraints of the three Zero Emissions Resources (ZERs). The example is not an optimised scenario, since that would require value-based judgements, but instead aims to stimulate innovation in mitigation strategies, which draw on sufficiency and efficiency strategies. Similarly, the feasibility of deployment of the technology mixes used in this example has not been assessed.

The process to derive the example is as follows:

1. **Assign delivery processes to minimise the need for Carbon Storage.** The technology mix, shown in Table S424, has been assigned by:

- Setting all electrification delivery processes to 100%, where the option exists;
- Using processes with Carbon Capture and Storage (CCS) only where the quantity of carbon dioxide produced is less than the residual emissions from all other process options (only cement fuelled by biomass with CCS falls into this category); and
- Using the IEA Net-Zero by 2050 scenario for technologies which are not easily specified by these rules (such as for biofuel production); and
- Methanol, Methane and HVCs are assigned to 100% fossil-fueled processes to avoid any carbon sequestration in plastics.[10]
- Some additional assumptions were needed for some activities. It was assumed that:
  - Electricity generation could be supplied by 80% non-emitting generation with 20% from biomass to help match peaking loads.
  - 20% of buildings will not be compatible for heat-pumps and so need direct electrical heating. All others are assigned to heat-pumps since those are the most energy efficient option.
  - Diets are 100% plant based, without rice.
  - Aviation is supplied by a mix of fuels (based on the IEA Net-Zero by 2050 scenario) rather than fully electrified because electrical aviation is only feasible for very short distance flights. 6% flights are assumed possible by electric aviation; today flights under 500km make up around 6% of carbon dioxide emissions from aviation (Graver et al., 2019).
  - Fugitive emissions from fossil fuel production can be avoided to the same extent as the IEA Net-Zero by 2050 scenario.

2. **Scale down rates of emitting activities** (and those which require carbon storage, e.g. cement production with CCS) until the demand is within the carbon storage "budget". Since some processes produce a much larger share of the carbon storage budget than others, reduce these more strongly so that the emissions from each are similar (i.e. no one activity dominates storage demand).
3. **Scale down rates of non-emitting activities** by the same proportion so demand is within the Non-Emitting Electricity "budget".

[10] We decided to exclude these options in this scenario because carbon sequestration in products is not accounted anywhere else in the analysis and there are high uncertainties surrounding the permanence of this kind of sequestration. See the SI Part 1.3.1 for details. It was not included here given the relative share of (uncertain) carbon sequestered in products would be high compared to (lower risk) geological storage. Using the IEA Net-Zero by 2050 Scenario process shares for these processes would give around 50% of total carbon sequestration in products (otherwise following the 'Low ZER' approach described here). In other scenarios, the carbon sequestered in products is a much smaller proportion of the total carbon sequestered and does not obscure the main conclusions.

6.7.1 Low ZER delivery process shares (α)

Table S424 lists the assigned delivery process shares. The table uses short names for the activities and processes, which each have a longer description, given in Section 6.8.

SectorName	Activity	Process Share	Process
AgricultureForests	NonWoodBiomass	26%	Residues
		20%	Pasture
		54%	ElecLowFertiliser
	RawFood	100%	PlantFoodNoRice
	Wood	100%	Elec
Buildings	Appliances	100%	Elec
	Cooking	100%	Elec
	Cooling	100%	Elec
	Lighting	100%	Elec
	SpaceHeat	20%	Elec
		80%	HeatPump
	WaterHeating	20%	Elec
		80%	HeatPump
Electricity	Electricity	100%	Distribution
	GeneratedElectricity	20%	Bio
		80%	NonEmitting
FuelsFeedstocks	Ammonia	100%	Elec
	BioFuel	79%	AdvBioFuel
		7%	BioDiesel
		14%	BioEthanol
	HVCs	100%	NET
	Hydrogen	100%	Elec
	Methane	100%	FF
	Methanol	100%	SMR
	Oil	100%	FF
	OtherPetChem	100%	NET
	Plastics	46%	Elec
		54%	RecycleElec
	Synfuel	100%	FT
	Urea	100%	Elec
Industry	Aluminium	100%	ScrapElec
	Cement	100%	BioCCS
	Construction	100%	Elec
	Food	100%	Elec
	Glass	100%	Elec
	OtherIndustry	100%	Elec
	Paper	100%	Elec
	Steel	100%	EAF
	Textiles	100%	Elec

(continued)

SectorName	Activity	Process Share	Process
Mining	ExtractedOilGas	100%	NETNoFugitive
	Coal	45%	Elec
		55%	ElecNoFugitive
	MinedMetalsMinerals	100%	Elec
NETs	CO2Product	100%	NetZero
	NetEmissions	100%	BECC
Transport	Aviation	45%	Bio
		6%	ElecT
		16%	JetA
		33%	PtL
	BusUse	100%	BEV
	CarUse	100%	BEV
	RailFreight	100%	Elec
	RailP	100%	Elec
	RoadFreight	50%	BEV
		50%	CEV
	Shipping	43%	Ammonia
		20%	BioICE
		16%	FFICE
		20%	HFCEV
		1%	SynICE
Waste	WasteSolid	20%	Feedstock
		5%	Incineration
		20%	MethaneUse
		55%	Recycling
	Wastewater	100%	BestPractice

Table S424: Shares provided by each delivery process for the Low ZER Scenario.

6.8 Model processes

Table S425: List of activities and processes currently included in the model.

Model Activity	Activity description	Process	Process description
**Agriculture and forests**			
NonWoodBiomass	Plant Agriculture	Residues	Use of residues
		Pasture	Use of pasture
		Elec	Electrified
		ElecLowFertiliser	Low fertiliser, electrified crop agriculture
		LowFertiliser	Low fertiliser crop agriculture
		NETs	Conventional crop agriculture
RawFood	Farming Food	NonRuminant-Meat	Animal agriculture, non-ruminant
		PlantBasedFood	Plant-based food only agriculture
		PlantFood-LowMethaneRice	Plant-based food only agriculture with low-methane rice
		PlantFoodNoRice	Plant-based food only agriculture without rice farming (increasing the share of this mode decreases the proportion of rice in the average global diet)
		RuminantFeedAd-ditives	Ruminant only agriculture with feed additives
		RuminantMeat	Ruminant only agriculture
Wood	Forestry	Elec	Electrified
		NETs	Conventional
**Buildings (energy use)**			
Appliances	Use of Appliances	Elec	Electrically Powered
Cooking	Cooking	Bio	Biomass Fuelled
		Elec	Electrically Powered
		Gas	Gas powered
Cooling	Cooling the Built Environment	Elec	Electrified
Lighting	Lighting	Elec	Electrically Powered
SpaceHeat	Space Heating	Bio	Biomass Fuelled
		Elec	Direct Electrical
		Gas	Gas boiler
		H2Boiler	Hydrogen Boiler
		HeatPump	Heat Pump (20% Ground; 80% Air Source)
WaterHeating	Water Heating	Bio	Biomass Fuelled
		Elec	Direct Electrical
		Gas	Gas boiler
		H2Boiler	Hydrogen Boiler
		HeatPump	Heat Pump (20% Ground; 80% Air Source)
**Electricity generation and distribution**			
Electricity	Distribution of Electricity	Distribution	Distribution of electricity with existing systems and losses
GeneratedElectricity	Electricity Generation	BECC	Bio-Energy with carbon capture (BECC)
		Bio	From Biomass (without carbon capture)
		Coal	Coal Powered
		CoalCCS	Coal Powered with integrated carbon capture
		Gas	Gas Powered
		GasCCS	Gas Powered with integrated carbon capture
		NonEmitting	Non-Emitting generation (renewable and nuclear)
**Fuels and feedstocks**			
Ammonia	Ammonia Production	CG	Ammonia from coal gasification
		Elec	Ammonia from electrolysis of water
		SMR	Steam Methane Reforming and Haber Bosch process
		SMRCCS	Steam Methane Reforming with Carbon Capture

(continued)

Model Activity	Activity description	Process	Process description
BioFuel	Biofuel Production	AdvBioFuel	Advanced (2nd generation) biofuels - based on the process for cellulosic ethanol by thermochemical methods
		BioDiesel	Conventional (1st generation) bio-diesel from farmed biomass
		BioEthanol	Conventional (1st generation) bio-ethanol from farmed biomass
HVCs	HVCs Production	BioEth	Bio-compatible route: Ethylene from Bioethanol dehydration, other HVCs from methanol
		CCS	Production with carbon capture
		MTOA	Methanol (methyl alcohol) to olefins/aromatics
		NET	Conventional Production with NETs
Hydrogen	Hydrogen Production	ATR	AutoThermal Reforming, ATR, of methane
		ATRCCS	AutoThermal Reforming, ATR, of methane with Carbon Capture
		CG	Coal Gasification
		CGCCS	Coal Gasification with carbon capture
		Elec	Electrolysis of water
		SMR	Steam Methane Reformation
		SMRCCS	Steam Methane Reformation with carbon capture
Methane	Methane Production	Biogas	Bio-methane production via anaerobic digestion of biomass
		FF	Methane from fossil-fuel reserves
		SynDAC	Synthetic methane production via the Sabatier Process with Carbon Dioxide as a feedstock
Methanol	Methanol Production	BioGas	Bio-Methanol (by gasification)
		CCS	Production from methane with carbon capture
		Coal	Coal Gasification
		FromH2	Synthetic Methanol (from Hydrogen)
		SMR	Steam Methane Reforming
Oil	Oil Processing and Refining	FF	Fossil Fuel Feedstock - processing and refining extracted oil
OtherPetChem	Production of other petrochemical products, not accounted for elsewhere	NET	Combination of chemical feedstocks to produce chemical substances not accounted for elsewhere in the model. Energy inputs are assumed negligible in comparison to the feedstock demands.
Plastics	Plastics Production - end-use quantity excludes plastics for fabrics	Bio	BioFuels replace Fossil Fuels
		CCS	Plastic production with integrated carbon capture
		Elec	Electrical Processes replace Fossil Fuels
		NET	Conventional Methods
		RecycleElec	Electrified mechanical recycling
Synfuel	Synfuel Production	FT	Production of synthetic hydrocarbon fuel from carbon dioxide and hydrogen by Fischer-Tropsch
Urea	Urea Production	Elec	Electrical fuelled steam production
		Gas	Natural gas fuelled steam production
**Industry**			
Aluminium	Aluminium Production	InertAnodes	Inert Anodes
		NET	Conventional primary production
		ScrapElec	Electrified secondary production from scrap
		ScrapNG	Secondary production from scrap, fuelled by natural gas
Cement	Cement Production	Bio	Conventional production fuelled by biomass
		BioCCS	Production with carbon capture (oxyfuel), fuelled 100% by woody fuels
		CCS	Production with Carbon Capture (oxyfuel)
		Coal	Conventional production fuelled by coal
		H2	Conventional production fuelled by hydrogen
		NGas	Conventional production fuelled by natural gas

(continued)

Model Activity	Activity description	Process	Process description
Construction	Construction	Elec	Electrified
		H2Power	Fuelled by hydrogen
		NET	Conventional construction technologies and techniques
Food	Food Processing	BioF	BioFuels replace Fossil Fuels
		Elec	Electrical Processes replace Fossil Fuels
		NET	Conventional Processing Methods
Glass	Glass Production	Elec	Electrified process (45% cullet rate)
		NET	Conventional processes (45% cullet rate)
OtherIndustry	Product Manufacturing and other Industrial Processes	Elec	Electrical processes replace fossil fuels (assumed completely)
		NET	Conventional industrial processes
Paper	Paper Production	Elec	Electrical Processes replace Fossil Fuels
		NET	Conventional Methods
Steel	Steel Production	BOF	Basic Oxygen Furnace primary steel
		CCS	Blast Furnace with Top Gas Recycling and Carbon Capture
		EAF	Scrap Steel Recycling via Electric Arc Furnace
		HDRI	Hydrogen DRI (Direct Iron Reduction)
		MethaneDRI	Natural gas fuelled steelmaking (MIDREX technology)
Textiles	Textiles Production	Elec	Electrical Processes replace Fossil Fuels
		NET	Conventional Methods
**Mining**			
ExtractedOilGas	Oil and Gas Extraction	Elec	Electrically Powered
		ElecNoFugitive	Electrical Processes replace Fossil Fuels with elimination of fugitive emissions
		NET	Conventional Methods
		NETNoFugitive	Conventional Methods with elimination of fugitive emissions
Coal	Coal Mining	Elec	Electrical Processes replace Fossil Fuels
		ElecNoFugitive	Electrical Processes replace Fossil Fuels with elimination of fugitive emissions
		H2	Hydrogen replaces Fossil Fuels
		H2NoFugitive	Hydrogen replaces Fossil Fuels with elimination of fugitive emissions
		NET	Conventional Methods
		NETNoFugitive	Conventional Methods with elimination of fugitive emissions
MinedMetalsMinerals	Minerals and Metals Mining	Elec	Electrical Processes replace Fossil Fuels
		H2	Hydrogen replaces Fossil Fuels
		NET	Conventional Methods
**Negative emissions technologies**			
CO2Product	Carbon dioxide gas management	NetZero	Storing captured carbon dioxide gas
		NoStorage	Releasing captured carbon dioxide gas
NetEmissions	Residual emissions management (negative emissions tech.)	BECC	Bio Energy with Carbon Capture
		DAC	Direct Air Capture
**Transport**			
Aviation	Aviation	Bio	Biofuel powered aviation - direct CO2 emissions only
		BioT	Biofuel powered aviation - including non-CO2 warming impacts
		ElecT	Battery electric aviation (only valid for short flights)
		JetA	JetA kerosene conventional aviation - direct CO2 emissions only

(continued)

Model Activity	Activity description	Process	Process description
		JetAT	JetA kerosene conventional aviation - including non-CO2 warming impacts
		LH2	Liquified hydrogen aviation - direct CO2 emissions only
		LH2T	Liquified hydrogen aviation - including non-CO2 warming impacts
		LNG	Liquified natural gas fuelled aviation - direct CO2 emissions only
		LNGT	Liquified natural gas fuelled aviation - including non-CO2 warming impacts
		PtL	Synthetic fuel powered aviation - direct CO2 emissions only
		PtLT	Synthetic fuel powered aviation - including non-CO2 warming impacts
BusUse	Bus Transportation	BEV	Battery electric bus passenger transport
		BioICE	Biofuel powered bus passenger transport
		FFICE	Conventional fossil-fuelled bus transport
		HFCEV	Hydrogen fuel cell bus passenger transport
		SynICE	Synthetic fuel powered bus passenger transport
CarUse	Car Transportation	BEV	Battery Electric
		BioICE	Biofuel Powered Internal Combustion Engine
		FFICE	Fossil Fuelled
		HFCEV	Hydrogen Fuel Cell Powered
		SynICE	Synfuel Powered Internal Combustion Engine
RailFreight	Rail Freight	BioICE	Biofuel Powered Internal Combustion Engine
		Elec	Electrified
		FFICE	Fossil Fuelled
		SynICE	Synfuel Powered Internal Combustion Engine
RailP	Passenger Rail Transportation	BioICE	Biofuel Powered Internal Combustion Engine
		Elec	Electrically Powered
		FFICE	Fossil Fuelled
		HFCEV	Hydrogen Fuel Cell Powered
		SynICE	Synfuel Powered Internal Combustion Engine
RoadFreight	Road Freight	BEV	Battery Electric Vehicle
		BioICE	Biofuel Powered Internal Combustion Engine
		CEV	Catenary Electric Vehicle
		FFICE	Fossil Fuelled
		HFCEV	Hydrogen Fuel Cell Powered
		SynICE	Synfuel Powered Internal Combustion Engine
Shipping	Shipping	Ammonia	Ammonia Powered Internal Combustion Engine
		BioICE	Biofuel Powered Internal Combustion Engine
		FFICE	Fossil Fuelled
		HFCEV	Hydrogen Fuel Cell Powered
		LNG	Liquified Natural Gas Internal Combustion Engine
		SynICE	Synfuel Powered Internal Combustion Engine
**Waste**			
WasteSolid	Solid Waste Management	Feedstock	Use of waste as a downstream fuel or as a feedstock for fuel production
		Incineration	Electricity generation from incineration of solid waste
		MethaneUse	Methane captured from landfill for use
		Landfill	Solid waste management by depositing onto or into land (landfill)
		Recycling	Waste recycled and/or reused
Wastewater	Wastewater treatment	BestPractice	Best practice wastewater management, eliminating CH4 and N2O emissions
		NET	Current wastewater management

Part 5 and 6 References

Adhya, T. K., Linquist, B., Searchinger, T., & Wassmann, R. (2014). Wetting and Drying : Reducing Greenhouse Gas Emissions and Saving Water from Rice Production. *World Resources Institute*, *December*, 1–28.

Almena, A., Thornley, P., Chong, K., & Röder, M. (2022). Carbon dioxide removal potential from decentralised bioenergy with carbon capture and storage (BECCS) and the relevance of operational choices. *Biomass and Bioenergy*, *159*, 106406. https://doi.org/10.1016/J.BIOMBIOE.2022.106406

Annamalai, K., Priyadarsan, S., Arumugam, S., & Sweeten, J. M. (2014). Conversion: Coal, Animal Waste, and Biomass Fuel Principles. In *Encyclopedia of energy engineering and technology, second edition* (pp. 299–317). CRC Press. https://doi.org/10.1081/e-eee2-120041557

Arens, M., Worrell, E., Eichhammer, W., Hasanbeigi, A., & Zhang, Q. (2017). Pathways to a low-carbon iron and steel industry in the medium-term – the case of Germany. *Journal of Cleaner Production*, *163*, 84–98. https://doi.org/10.1016/j.jclepro.2015.12.097

Arrigoni, Alessandro., & Bravo Diaz, Laura. (2022). *Hydrogen emissions from a hydrogen economy and their potential global warming impact. KJ-NA-31-188-EN-N (online).* https://doi.org/10.2760/065589 (online)

Atuonwu, J., & Tassou, S. (2021). *Decarbonisation of food manufacturing by the electrification of heat: A review of developments, technology options and future directions* (Vol. 107, pp. 168–182). Elsevier. https://doi.org/10.1016/j.tifs.2020.10.011

Bajželj, B., Richards, K. S., Allwood, J. M., Smith, P., Dennis, J. S., Curmi, E., & Gilligan, C. A. (2014). Importance of food-demand management for climate mitigation. *Nature Climate Change*, *4*(10), 924–929. https://doi.org/10.1038/nclimate2353

Balcombe, P., Brierley, J., Lewis, C., Skatvedt, L., Speirs, J., Hawkes, A., & Staffell, I. (2019). How to decarbonise international shipping: Options for fuels, technologies and policies. *Energy Conversion and Management*, *182*, 72–88.

Balcombe, P., Speirs, J., Johnson, E., Martin, J., Brandon, N., & Hawkes, A. (2018). *The carbon credentials of hydrogen gas networks and supply chains.* https://doi.org/10.1016/j.rser.2018.04.089

Barker, D. J., Turner, S. A., Napier-Moore, P. A., Clark, M., & Davison, J. E. (2009). CO2 Capture in the Cement Industry. *Energy Procedia*, *1*(1), 87–94. https://doi.org/https://doi.org/10.1016/j.egypro.2009.01.014

Beer, J. de, Cihlar, J., Hensing, I., & Zabeti, M. (2017). *Status and prospects of co- processing of waste in EU cement plants* (April; p. 13). Ecofys-Navigant. https://cembureau.eu/media/rjqiyqca/2017-05-11_ecofys_publication_alternativefuels_report.pdf

BEIS. (2021a). *Energy Consumption in the UK (ECUK).* https://www.gov.uk/government/statistics/energy-consumption-in-the-uk-2021

BEIS. (2021b). *Net Zero Strategy: Build Back Greener.* https://www.gov.uk/government/publications/net-zero-strategy

Beuttler, C., Charles, L., & Wurzbacher, J. (2019). The Role of Direct Air Capture in Mitigation of Anthropogenic Greenhouse Gas Emissions. *Frontiers in Climate*, *1*, 10. https://doi.org/10.3389/fclim.2019.00010

Bogner, J., Abdelrafie Ahmed, M., Diaz, C., Faaij, A., Gao, Q., Hashimoto, S., Mareckova, K., Pipatti, R., & Zhang, T. (2007). Waste Management. In *Climate change 2007: Mitigation. Contribution of working group III to the fourth assessment report of the intergovernmental panel on climate change.* https://www.ipcc.ch/site/assets/uploads/2018/02/ar4-wg3-chapter10-1.pdf

Broehm, M., Strefler, J., & Bauer, N. (2015). Techno-Economic Review of Direct Air Capture Systems for Large Scale Mitigation of Atmospheric CO2. *SSRN Electronic Journal.* https://doi.org/10.2139/ssrn.2665702

Cabernard, L., Pfister, S., Oberschelp, C., & Hellweg, S. (2022). Growing environmental footprint of plastics driven by coal combustion. *Nature Sustainability*, *5*(2), 139–148. https://doi.org/10.1038/s41893-021-00807-2

Cavalett, O., Watanabe, M. D. B., Fleiger, K., Hoenig, V., & Cherubini, F. (2022). LCA and negative emission potential of retrofitted cement plants under oxyfuel conditions at high biogenic fuel shares. *Scientific Reports*, *12*(1), 8924. https://doi.org/10.1038/s41598-022-13064-w

CEMBUREAU. (2020). Cementing the European Green Deal. *The European Cement Association, Brussels*, 1–38. https://cembureau.eu/library/reports/2050-carbon-neutrality-roadmap/ https://cembureau.eu/media/kuxd32gi/cembureau-2050-roadmap_final-version_web.pdf

CNBC. (2018). *Innovation: Using less energy to liquefy natural gas.* https://www.cnbc.com/advertorial/2018/10/08/innovation-using-less-energy-to-liquefy-natural-gas.html

Committee on Climate Change. (2019). *Net Zero Technical Report. May*, 19–292. https://www.theccc.org.uk/publication/net-zero-technical-report/

Confederation of European Paper Industries (CEPI). (2021). *European Pulp & Paper Industry - Key Statictics 2020.* https://www.cepi.org/key-statistics-2020/

Connolly, D., Lund, H., & Mathiesen, B. V. (2016). *Renewable and Sustainable Energy Reviews*, *60*, 1634–1653.

https://doi.org/10.1016/J.RSER.2016.02.025

Connolly, D., Mathiesen, B. V., & Ridjan, I. (2014). *Energy*, *73*, 110–125. https://doi.org/10.1016/J.ENERGY.2014.05.104

Correa, G., Muñoz, P. M., & Rodriguez, C. R. (2019). A comparative energy and environmental analysis of a diesel, hybrid, hydrogen and electric urban bus. *Energy*, *187*, 115906. https://doi.org/10.1016/j.energy.2019.115906

Cusano, G., Rodrigo Gonzalo, M., Farrell, F., Remus, R., Roudier, S., & Delgado Sancho, L. (2017). *Best Available Techniques (BAT) Reference Document for the Non-Ferrous Metals Industries.* doi:10.2760/8224

Daioglou, V., Stehfest, E., Wicke, B., Faaij, A., & Vuuren, D. P. van. (2016). Projections of the availability and cost of residues from agriculture and forestry. *GCB Bioenergy*, *8*(2), 456–470. https://doi.org/10.1111/GCBB.12285

Dalena, F., Senatore, A., Marino, A., Gordano, A., Basile, M., & Basile, A. (2018). *Chapter 1 - Methanol Production and Applications: An Overview* (A. Basile & F. B. T.-. M. Dalena, Eds.; pp. 3–28). Elsevier. https://doi.org/https://doi.org/10.1016/B978-0-444-63903-5.00001-7

Davis, S. J., Lewis, N. S., Shaner, M., Aggarwal, S., Arent, D., Azevedo, I. L., Benson, S. M., Bradley, T., Brouwer, J., Chiang, Y. M., Clack, C. T. M., Cohen, A., Doig, S., Edmonds, J., Fennell, P., Field, C. B., Hannegan, B., Hodge, B. M., Hoffert, M. I., … Caldeira, K. (2018). *Net-zero emissions energy systems* (6396; Vol. 360). American Association for the Advancement of Science. https://doi.org/10.1126/science.aas9793

Department for Business Energy & Industrial Strategy. (2018). *Government conversion factors for company reporting of greenhouse gas emissions.* https://www.gov.uk/government/collections/government-conversion-factors-for-company-reporting

Department for Transport. (2020). *ENV0103: Average new car fuel consumption: Great Britain.* https://www.gov.uk/government/statistical-data-sets/energy-and-environment-data-tables-env

Derwent, R. G. (2018). *Hydrogen for Heating: Atmospheric Impacts.* BEIS. https://assets.publishing.service.gov.uk/government/uploads/system/uploads/attachment_data/file/760538/Hydrogen_atmospheric_impact_report.pdf

Deutz, S., & Bardow, A. (2021). Life-cycle assessment of an industrial direct air capture process based on temperature–vacuum swing adsorption. *Nature Energy*, *6*(2), 203–213. https://doi.org/10.1038/s41560-020-00771-9

DNV GL. (2018). *Energy Transition outlook 2018 - Maritime Forecast to 2050.* 148. https://eto.dnvgl.com/2018/maritime

DOE Hydrogen and Fuel Cell Technologies Office. (2012). *Multi-Year Research, Development, and Demonstration Plan.* https://www.energy.gov/eere/fuelcells/articles/hydrogen-and-fuel-cell-technologies-office-multi-year-research-development

Dray, L., Schäfer, A. W., Grobler, C., Falter, C., Allroggen, F., Stettler, M. E. J., & Barrett, S. R. H. (2022). Cost and emissions pathways towards net-zero climate impacts in aviation. *Nature Climate Change*, *12*(10), 956–962. https://doi.org/10.1038/s41558-022-01485-4

Eggleston, H. S., Buendia, L., Miwa, K., Ngara, T., & Tanabe, K. (2006). *2006 IPCC guidelines for national greenhouse gas inventories.* https://www.ipcc-nggip.iges.or.jp/public/2006gl

Element Energy Ltd. (2015). *Research on district heating and local approaches to heat decarbonisation* (November). The Committee on Climate Change. https://www.theccc.org.uk/publication/element-energy-for-ccc-research-on-district-heating-and-local-approaches-to-heat-decarbonisation/

Element Energy Ltd. (2018). *Hydrogen supply chain evidence base.* Department for Business, Energy; Industrial Strategy. https://www.gov.uk/government/publications/hydrogen-supply-chain-evidence-base

Element Energy Ltd. (2021). *Development of trajectories for residential heat decarbonisation to inform the Sixth Carbon Budget* (December). The Committee on Climate Change. https://www.theccc.org.uk/publication/development-of-trajectories-for-residential-heat-decarbonisation-to-inform-the-sixth-carbon-budget-element-energy/

Ellen MacArthur Foundation. (2017). *A new textiles economy: Redesigning fashion's future.* https://ellenmacarthurfoundation.org/publications

Elsayed, M. A., Matthews, R., & Mortimer, N. D. (2003). *Carbon and energy balances for a range of biofuels options.* DTI Sustainable Energy Programmes. https://www.osti.gov/etdeweb/servlets/purl/20359706

European Commission JRC (Datasets). (2022). *EDGAR (Emissions Database for Global Atmospheric Research) Community GHG Database (a collaboration between the European Commission, Joint Research Centre (JRC), the International Energy Agency (IEA), and comprising IEA-EDGAR CO2, EDGAR CH4, EDGAR N2O, EDG.* https://edgar.jrc.ec.europa.eu/dataset_ghg70

Eyring, V., Isaksen, I. S. A., Berntsen, T., Collins, W. J., Corbett, J. J., Endresen, O., Grainger, R. G., Moldanova, J., Schlager, H., & Stevenson, D. S. (2010). Transport impacts on atmosphere and climate: Shipping. *Atmospheric Environment*, *44*(37), 4735–4771. https://doi.org/10.1016/j.atmosenv.2009.04.059

FAO. (2022a). *FAOSTAT Emissions Totals.* Licence: CC BY-NC-SA 3.0 IGO. https://www.fao.org/faostat/en/?#data/GT

FAO. (2022b). *FAOSTAT Fertilizers by Nutrient.* Licence: CC BY-NC-SA 3.0 IGO. https://www.fao.org/faostat/en/

?#data/RFN

FAO. (2022c). *FAOSTAT Food Balances.* Licence: CC BY-NC-SA 3.0 IGO. https://www.fao.org/faostat/en/?#data/FBS

FAO. (2022d). *FAOSTAT Forestry Production and Trade.* Licence: CC BY-NC-SA 3.0 IGO. https://www.fao.org/faostat/en/?#data/FO

Faramawy, S., Zaki, T., & Sakr, A. A.-E. (2016). Natural gas origin, composition, and processing: A review. *Journal of Natural Gas Science and Engineering*, *34*, 34–54. https://doi.org/https://doi.org/10.1016/j.jngse.2016.06.030

Fasihi, M., Efimova, O., & Breyer, C. (2019). Techno-economic assessment of CO2 direct air capture plants. *Journal of Cleaner Production*, *224*, 957–980. https://doi.org/https://doi.org/10.1016/j.jclepro.2019.03.086

Fertilizers Europe. (2000). *Best Available Techniques for Pollution Prevention and Control in the European Fertilizer Industry - Production of Ammonia.* https://www.fertilizerseurope.com/wp-content/uploads/2019/08/Booklet_1_final.pdf

Fisch und Fischl GmbH. (2022). *Spritmonitor.de.* https://www.spritmonitor.de/en/search.html

Gabrielli, P., Rosa, L., Gazzani, M., Meys, R., Bardow, A., Mazzotti, M., & Sansavini, G. (2023). *Net-zero emissions chemical industry in a world of limited resources.* https://doi.org/10.1016/j.oneear.2023.05.006

Gao, Y., & Cabrera Serrenho, A. (2023). Greenhouse gas emissions from nitrogen fertilizers could be reduced by up to one-fifth of current levels by 2050 with combined interventions. *Nature Food 2023*, 1–9. https://doi.org/10.1038/s43016-023-00698-w

Gardiner, M., & Satyapal, S. (2009). *DOE Hydrogen and Fuel Cells Program Record 9013: Energy requirements for hydrogen gas compression and liquefaction as related to vehicle storage needs.* US Department of Energy. https://www.hydrogen.energy.gov/pdfs/9013_energy_requirements_for_hydrogen_gas_compression.pdf

Garnett, T. (2011). Where are the best opportunities for reducing greenhouse gas emissions in the food system (including the food chain)? *Food Policy*, *36*(SUPPL. 1), S23–S32. https://doi.org/10.1016/j.foodpol.2010.10.010

Gautam, M., Pandey, B., & Agrawal, M. (2017). Carbon footprint of aluminum production. In *Environmental carbon footprints: Industrial case studies* (pp. 197–228). https://doi.org/10.1016/B978-0-12-812849-7.00008-8

GCCA. (2024). *Oxyfuel.* https://gccassociation.org/cement-and-concrete-innovation/carbon-capture-and-utilisation/oxyfuel/

Geist, H. J., & Lambin, E. F. (2002). Proximate causes and underlying driving forces of tropical deforestation. *BioScience*, *52*(2), 143–150. https://doi.org/10.1641/0006-3568(2002)052%5B0143:PCAUDF%5D2.0.CO;2

German Environment Agency. (2020). *Power-to-Liquids Potentials and Perspectives for the Future Supply of Renewable Aviation Fuel.* https://www.umweltbundesamt.de/en/publikationen/

Ghasemzadeh, K., Sadati Tilebon, S. M., Nasirinezhad, M., & Basile, A. (2018). *Chapter 23 - Economic Assessment of Methanol Production* (A. Basile & F. B. T.-. M. Dalena, Eds.; pp. 613–632). Elsevier. https://doi.org/https://doi.org/10.1016/B978-0-444-63903-5.00023-6

Giddey, S., Badwal, S. P. S., & Kulkarni, A. (2013). *Review of electrochemical ammonia production technologies and materials* (34; Vol. 38, pp. 14576–14594). Pergamon. https://doi.org/10.1016/j.ijhydene.2013.09.054

Global Cement and Concrete Association. (n.d.). *GNR Project.* Retrieved July 30, 2022, from https://gccassociation.org/gnr/

Global CSS Institute (GCCSI). (2022). *Global Status of CCS 2022.* https://status22.globalccsinstitute.com/wp-content/uploads/2022/12/Global-Status-of-CCS-2022_Download_1222.pdf

Gnadt, A. R., Speth, R. L., Sabnis, J. S., & Barrett, S. R. H. (2019). *Technical and environmental assessment of all-electric 180-passenger commercial aircraft* (Vol. 105, pp. 1–30). https://doi.org/10.1016/j.paerosci.2018.11.002

Gómez, D., Watterson, J., Americano, B., Ha, C., Marland, G., Matsika, E., Nenge Namayanga, L., Osman-Elasha, B., Kalenga Saka, J., Treanton, K., & Quadrelli, R. (2006). 2.1 Stationary Combustion. In *IPCC guidelines for national greenhouse gas inventories.* IPCC. https://www.ipcc-nggip.iges.or.jp/

Graver, B., Zhang, K., & Rutherford, D. (2019). *CO2 emissions from commercial aviation, 2018.* The ICCT. https://theicct.org/publication/co2-emissions-from-commercial-aviation-2018/

Griscom, B. W., Adams, J., Ellis, P. W., Houghton, R. A., Lomax, G., Miteva, D. A., Schlesinger, W. H., Shoch, D., Siikamäki, J. V., Smith, P., Woodbury, P., Zganjar, C., Blackman, A., Campari, J., Conant, R. T., Delgado, C., Elias, P., Gopalakrishna, T., Hamsik, M. R., ... Fargione, J. (2017). Natural climate solutions. *Proceedings of the National Academy of Sciences of the United States of America*, *114*(44), 11645–11650. https://doi.org/10.1073/PNAS.1710465114/SUPPL_FILE/PNAS.1710465114.SAPP.PDF

Gucwa, M., & Schäfer, A. (2013). The impact of scale on energy intensity in freight transportation. *Transportation Research Part D: Transport and Environment*, *23*, 41–49. https://doi.org/10.1016/j.trd.2013.03.008

Hager, T. J., & Morawicki, R. (2013). *Energy consumption during cooking in the residential sector of developed nations: A review.* https://doi.org/10.1016/j.foodpol.2013.02.003

Hardenburger, T. L., & Ennis, M. (2005). Nitrogen. In *Kirk-othmer encyclopedia of chemical technology.* Wiley Online

Library. https://onlinelibrary-wiley-com.ezp.lib.cam.ac.uk/doi/10.1002/0471238961.1409201808011804.a01.pub2

Haugen, H. A., Eldrup, N. H., Fatnes, A. M., & Leren, E. (2017). Commercial Capture and Transport of CO2 from Production of Ammonia. *Energy Procedia*, *114*(1876), 6133–6140. https://doi.org/10.1016/j.egypro.2017.03.1750

Heidelberg Materials Sement Norge. (2024). *Brevik CCS – World's first CO -capture facility in the cement industry.* https://www.brevikccs.com/en

Hill, B., Hovorka, S., & Melzer, S. (2013). Geologic carbon storage through enhanced oil recovery. *Energy Procedia*, *37*, 6808–6830. https://doi.org/10.1016/j.egypro.2013.06.614

Hills, T., Florin, N., & Fennell, P. S. (2016). Decarbonising the cement sector: A bottom-up model for optimising carbon capture application in the UK. *Journal of Cleaner Production*, *139*, 1351–1361. https://doi.org/10.1016/j.jclepro.2016.08.129

Howarth, R. W. (2014). A bridge to nowhere: Methane emissions and the greenhouse gas footprint of natural gas. *Energy Science and Engineering*, *2*(2), 47–60. https://doi.org/10.1002/ese3.35

Huss, A., & Weingerl, P. (2020). *JEC Tank-To-Wheels report v5: Passenger cars* (H. Mass, C. Herudek, J. Wind, B. Hollweck, L. De Prada, S. Deix, D. Lahaussois, R. Faucon, F. Heurtaux, B. Perrier, F. Vidal, G. Gomes Marques, M. Prussi, L. Lonza, M. Yugo, & H. Hamje, Eds.). The European Commission; Publications Office of the European Union. https://doi.org/10.2760/557004

IATA. (2024). *Air Cargo Monthly Analysis. May*, 1–5. https://www.iata.org/en/iata-repository/publications/economic-reports/air-cargo-market-analysis-may-2024/

IEA. (2021a). Ammonia Technology Roadmap. *Ammonia Technology Roadmap.* https://doi.org/10.1787/f6daa4a0-en

IEA. (2021b). *Net Zero by 2050: A Roadmap for the Global Energy Sector.* https://www.iea.org/reports/net-zero-by-2050

IEA. (2022a). Direct Air Capture: A key technology for net zero. *IEA Publications*, 1–76.

IEA. (2022b). *The Future of Heat Pumps.* https://doi.org/10.1787/2bd71107-en

IEA. (2023a). *Global Hydrogen Review 2023.* International Energy Agency (IEA). https://www.iea.org/reports/global-hydrogen-review-2023

IEA. (2023b). *Net Zero Roadmap: A Global Pathway to Keep the 1.5 °C Goal in Reach.* IEA. https://www.iea.org/reports/net-zero-roadmap-a-global-pathway-to-keep-the-15-0c-goal-in-reach

IEA. (2023c). *Strategies to reduce emissions from coal supply.* https://www.iea.org/reports/global-methane-tracker-2023/strategies-to-reduce-emissions-from-coal-supply

IEA ETSAP. (2012). *Technology Brief: Cooking Appliances.* www.etsap.org

International Aluminium Institute. (2022). *2019 Life Cycle Inventory (LCI) Data and Environmental Metrics.* https://international-aluminium.org/resource/2019-life-cycle-inventory-lci-data-and-environmental-metrics/

International Association of Oil & Gas Producers. (2000). *Flaring & venting in the oil & gas exploration & production industry. 2*, 18. https://doi.org/Report No. 2.79/288

International Energy Agency. (2017). *The Future of Trucks – Implications for Energy and the Environment.* https://www.iea.org/reports/the-future-of-trucks

International Energy Agency. (2018). *The Future of Petrochemicals.* www.iea.org

International Energy Agency. (2019a). *The Future of Rail Opportunities for energy and the environment.* https://www.iea.org/reports/the-future-of-rail www.iea.org/t&c/

International Energy Agency. (2019b). *World Energy Outlook 2019.* https://www.iea.org/reports/world-energy-outlook-2019

International Energy Agency. (2020). *Iron and Steel Technology Roadmap.* https://www.iea.org/reports/iron-and-steel-technology-roadmap

International Energy Agency. (2021a). *Dataset: International Energy Agency World Energy Balances, 1960-2020.* UK Data Service. https://doi.org/10.5257/iea/web/2021

International Energy Agency. (2021b). *Energy Efficiency Indicators.* https://doi.org/https://doi.org/10.5257/iea/eei/2021

International Energy Agency. (2022). *Electricity Information.* UK Data Service. https://doi.org/10.5257/iea/elec/2022

International Energy Agency - IEA. (2020). *World energy balances database documentation.* https://stats2.digitalresources.jisc.ac.uk/metadata/IEA/WEB/World_Energy_Balances_2020_Documentation.pdf

International Finance Corporation. (2017). *Increasing the Use of Alternative Fuels at Cement Plants: International Best Practice.* https://doi.org/10.1596/28134

IRENA. (2022). *Green hydrogen for industry: A guide to policy making.* www.irena.org

Irving, W., & Tailakov, O. (2000). CH4 emissions: coal mining and handling. *Background Papers IPCC Expert Meetings on Good Practice Guidance and Uncertainty Management in National Greenhouse Gas Inventories*, 129–144. https://www.ipcc-nggip.iges.or.jp/public/gp/bgp/2_7_Coal_Mining_Handling.pdf

Jardine, C. N., Boardman, B., Osman, A., Vowles, J., & Palmer, J. (2003). Methane UK. *Environmental Change*

Institute (ECI), *44*(0), 1–96. http://www.eci.ox.ac.uk/research/energy/downloads/methaneuk/methaneukreport.pdf

Jensen, E. S., Carlsson, G., & Hauggaard-Nielsen, H. (2020). Intercropping of grain legumes and cereals improves the use of soil N resources and reduces the requirement for synthetic fertilizer N: A global-scale analysis. *Agronomy for Sustainable Development*, *40*(1). https://doi.org/10.1007/s13593-020-0607-x

Jiang, M., Cao, Y., Liu, C., Chen, D., Zhou, W., Wen, Q., Yu, H., Jiang, J., Ren, Y., Hu, S., Hertwich, E., & Zhu, B. (2024). Tracing fossil-based plastics, chemicals and fertilizers production in China. *Nature Communications*, *15*(1), 3854. https://doi.org/10.1038/s41467-024-47930-0

Jolleys, M., & Duddy, P. (2022). *Climate TRACE - Mineral Extraction methodology document*. Hypervine. https://github.com/climatetracecoalition

Jones, E. R., Vliet, M. T. H. van, Qadir, M., & Bierkens, M. F. P. (2021). Country-level and gridded estimates of wastewater production, collection, treatment and reuse. *Earth System Science Data*, *13*(2), 237–254. https://doi.org/10.5194/essd-13-237-2021

Karacan, C. Ö., Ruiz, F. A., Cotè, M., & Phipps, S. (2011). Coal mine methane: A review of capture and utilization practices with benefits to mining safety and to greenhouse gas reduction. *International Journal of Coal Geology*, *86*(2), 121–156. https://doi.org/https://doi.org/10.1016/j.coal.2011.02.009

Kauw, M., Benders, R. M. J., & Visser, C. (2015). Green methanol from hydrogen and carbon dioxide using geothermal energy and/or hydropower in Iceland or excess renewable electricity in Germany. *Energy*, *90*, 208–217. https://doi.org/10.1016/j.energy.2015.06.002

Kaza, S., Yao, L. C., Bhada-Tata, P., & Van Woerden, F. (2018). *What a Waste 2.0: A Global Snapshot of Solid Waste Management to 2050*. Washington, DC: World Bank. https://doi.org/10.1596/978-1-4648-1329-0

Keith, D. W., Holmes, G., St. Angelo, D., & Heidel, K. (2018). A Process for Capturing CO2 from the Atmosphere. *Joule*, *2*(8), 1573–1594. https://doi.org/10.1016/j.joule.2018.05.006

Kennard, H., Oreszczyn, T., Mistry, M., & Hamilton, I. (2022). Energy & Buildings Population-weighted degree-days : The global shift between heating and cooling. *Energy & Buildings*, *271*, 112315. https://doi.org/10.1016/j.enbuild.2022.112315

Keys, A., Van Hout, M., & Daniels, B. (2019). *Decarbonisation options for the Dutch steel industry. Nov.* www.pbl.nl/en.

Kholod, N., Evans, M., Pilcher, R. C., Roshchanka, V., Ruiz, F., Coté, M., & Collings, R. (2020). Global methane emissions from coal mining to continue growing even with declining coal production. *Journal of Cleaner Production*, *256*, 120489. https://doi.org/10.1016/j.jclepro.2020.120489

Klinghoffer, N. B., Themelis, N. J., & Castaldi, M. J. (2013). 1 - Waste to energy (WTE): an introduction. In N. B. Klinghoffer & M. J. Castaldi (Eds.), *Waste to energy conversion technology* (pp. 3–14). Woodhead Publishing. https://doi.org/https://doi.org/10.1533/9780857096364.1.3

Kuramochi, T., Ramírez, A., Turkenburg, W., & Faaij, A. (2012). Comparative assessment of CO2 capture technologies for carbon-intensive industrial processes. *Progress in Energy and Combustion Science*, *38*(1), 87–112. https://doi.org/10.1016/j.pecs.2011.05.001

Kvande, H., & Haupin, W. (2001). Inert anodes for Al smelters: Energy balances and environmental impact. *Jom*, *53*(5), 29–33. https://doi.org/10.1007/s11837-001-0205-6

Lask, J., Rukavina, S., Zorić, I., Kam, J., Kiesel, A., Lewandowski, I., & Wagner, M. (2021). Lignocellulosic ethanol production combined with CCS—A study of GHG reductions and potential environmental trade-offs. *GCB Bioenergy*, *13*(2), 336–347. https://doi.org/10.1111/gcbb.12781

Lee, D. S., Fahey, D. W., Skowron, A., Allen, M. R., Burkhardt, U., Chen, Q., Doherty, S. J., Freeman, S., Forster, P. M., Fuglestvedt, J., Gettelman, A., De León, R. R., Lim, L. L., Lund, M. T., Millar, R. J., Owen, B., Penner, J. E., Pitari, G., Prather, M. J., … Wilcox, L. J. (2021). The contribution of global aviation to anthropogenic climate forcing for 2000 to 2018. *Atmospheric Environment*, *244*(February 2020). https://doi.org/10.1016/j.atmosenv.2020.117834

Levi, P. G., & Cullen, J. M. (2018). Mapping Global Flows of Chemicals: From Fossil Fuel Feedstocks to Chemical Products. *Environmental Science and Technology*, *52*(4), 1725–1734. https://doi.org/10.1021/ACS.EST.7B04573/ASSET/IMAGES/LARGE/ES-2017-04573E_0002.JPEG

Li, J., & Cheng, W. (2020). Comparative life cycle energy consumption, carbon emissions and economic costs of hydrogen production from coke oven gas and coal gasification. *International Journal of Hydrogen Energy*, *45*(51), 27979–27993. https://doi.org/https://doi.org/10.1016/j.ijhydene.2020.07.079

Li, J., Ma, X., Liu, H., & Zhang, X. (2018). Life cycle assessment and economic analysis of methanol production from coke oven gas compared with coal and natural gas routes. *Journal of Cleaner Production*, *185*, 299–308. https://doi.org/https://doi.org/10.1016/j.jclepro.2018.02.100

Li, J., Wei, Y.-M., Liu, L., Li, X., & Yan, R. (2022). The carbon footprint and cost of coal-based hydrogen production with and without carbon capture and storage technology in China. *Journal of Cleaner Production*, *362*, 132514.

https://doi.org/https://doi.org/10.1016/j.jclepro.2022.132514

Liu, Y., Li, G., Chen, Z., Shen, Y., Zhang, H., Wang, S., Qi, J., Zhu, Z., Wang, Y., & Gao, J. (2020). Comprehensive analysis of environmental impacts and energy consumption of biomass-to-methanol and coal-to-methanol via life cycle assessment. *Energy*, *204*, 117961. https://doi.org/https://doi.org/10.1016/j.energy.2020.117961

Lloyd's Register, & UMAS. (2020). *Techno-economic assessment of zero-carbon fuels* (March).

Logan, K. G., Nelson, J. D., McLellan, B. C., & Hastings, A. (2020). Electric and hydrogen rail: Potential contribution to net zero in the UK. *Transportation Research Part D: Transport and Environment*, *87*, 102523. https://doi.org/10.1016/j.trd.2020.102523

Lund, H., Möller, B., Mathiesen, B. V., & Dyrelund, A. (2010). The role of district heating in future renewable energy systems. *Energy*, *35*(3), 1381–1390. https://doi.org/10.1016/j.energy.2009.11.023

Machaj, K., Kupecki, J., Malecha, Z., Morawski, A. W., Skrzypkiewicz, M., Stanclik, M., & Chorowski, M. (2022). Ammonia as a potential marine fuel: A review. *Energy Strategy Reviews*, *44*, 100926. https://doi.org/10.1016/j.esr.2022.100926

Madhu, K., Pauliuk, S., Dhathri, S., & Creutzig, F. (2021). Understanding environmental trade-offs and resource demand of direct air capture technologies through comparative life-cycle assessment. *Nature Energy*, *6*(11), 1035–1044. https://doi.org/10.1038/s41560-021-00922-6

Mærsk Mc-Kinney Møller Center for Zero Carbon Shipping. (2021). *Position Paper: Fuel Option Scenarios*. Mærsk Mc-Kinney Møller Center for Zero Carbon Shipping.

Mahmoud, M., Garnett, R., Ferguson, M., & Kanaroglou, P. (2016). *Electric buses: A review of alternative powertrains* (Vol. 62, pp. 673–684). Elsevier Ltd. https://doi.org/10.1016/j.rser.2016.05.019

Mander, S., Anderson, K., Larkin, A., Gough, C., & Vaughan, N. (2017). The Role of Bio-energy with Carbon Capture and Storage in Meeting the Climate Mitigation Challenge: A Whole System Perspective. *Energy Procedia*, *114*(November 2016), 6036–6043. https://doi.org/10.1016/j.egypro.2017.03.1739

Manibog, F. (1984). Improved cooking stoves in developing countries: Problems and Opportunities. *Annual Review Energy.*

Material Economics. (2019). *Industrial transformation 2050 - Pathways to net-zero emissions from EU heavy industry.* https://materialeconomics.com/publications/publication/industrial-transformation-2050

McKinsey & Company. (2020). *The future of oil and gas is now: How companies can decarbonize* (January; pp. 1–11). https://www.mckinsey.com/industries/oil-and-gas/our-insights/the-future-is-now-how-oil-and-gas-companies-can-decarbonize

McQueen, N., Gomes, K. V., McCormick, C., Blumanthal, K., Pisciotta, M., & Wilcox, J. (2021). *A review of direct air capture (DAC): Scaling up commercial technologies and innovating for the future* (3; Vol. 3, p. 032001). IOP Publishing. https://doi.org/10.1088/2516-1083/abf1ce

Meessen, J. (2014). Urea synthesis. *Chemie Ingenieur Technik*, *86*(12), 2180–2189. https://doi.org/10.1002/CITE.201400064

Meili, C., Jungbluth, N., & Bussa, M. (2022). *Life cycle inventories of crude oil and natural gas extraction.* commissioned by ecoinvent. http://esu-services.ch/data/public-lci-reports/

Meys, R., Frick, F., Westhues, S., Sternberg, A., Klankermayer, J., & Bardow, A. (2020). Towards a circular economy for plastic packaging wastes – the environmental potential of chemical recycling. *Resources, Conservation and Recycling*, *162*, 105010. https://doi.org/https://doi.org/10.1016/j.resconrec.2020.105010

Mineral Products Association. (2019). *Options for switching UK cement production sites to near zero CO 2 emission fuel: Technical and financial feasibility. October 2019.*

Minx, J. C., Lamb, W. F., Andrew, R. M., Canadell, J. G., Crippa, M., Döbbeling, N., Forster, P. M., Guizzardi, D., Olivier, J., Peters, G. P., & Others. (2021). A comprehensive and synthetic dataset for global, regional, and national greenhouse gas emissions by sector 1970-2018 with an extension to 2019. *Earth System Science Data*, *13*(11), 5213–5252. https://essd.copernicus.org/articles/13/5213/2021/

Mottet, A., Haan, C. de, Falcucci, A., Tempio, G., Opio, C., & Gerber, P. (2017). Livestock: On our plates or eating at our table? A new analysis of the feed/food debate. *Global Food Security*, *14*, 1–8. https://doi.org/https://doi.org/10.1016/j.gfs.2017.01.001

Moya, J. A., & Pardo, N. (2013). The potential for improvements in energy efficiency and CO2 emissions in the EU27 iron and steel industry under different payback periods. *Journal of Cleaner Production*, *52*, 71–83. https://doi.org/10.1016/j.jclepro.2013.02.028

Moya, J., Pardo, N., & Mercier, A. (2010). *Energy efficiency and CO2 emissions : Prospective scenarios for the cement industry.* https://doi.org/10.2790/25732

Muratori, M., Kheshgi, H., Mignone, B., Clarke, L., Mcjeon, H., & Edmonds, J. (2017). Carbon capture and storage across fuels and sectors in energy system transformation pathways. *International Journal of Greenhouse Gas Control*, *57*, 34–41. https://doi.org/10.1016/j.ijggc.2016.11.026

Nabuurs, G.-J., & Mrabet, R. (2022). Agriculture, Forestry and Other Land Uses (AFOLU). In *IPCC, 2022: Climate change 2022: Mitigation of climate change. Contribution of working group III to the sixth assessment report of the intergovernmental panel on climate change* (pp. 747–860). https://doi.org/10.1017/9781009157926.009

NASEM. (2019). *Negative Emissions Technologies and Reliable Sequestration.* https://doi.org/10.17226/25259

Obrist, M. D., Kannan, R., Schmidt, T. J., & Kober, T. (2022). Long-term energy efficiency and decarbonization trajectories for the Swiss pulp and paper industry. *Sustainable Energy Technologies and Assessments*, *52*, 101937. https://doi.org/10.1016/J.SETA.2021.101937

Oni, A. O., Anaya, K., Giwa, T., Di Lullo, G., & Kumar, A. (2022). Comparative assessment of blue hydrogen from steam methane reforming, autothermal reforming, and natural gas decomposition technologies for natural gas-producing regions. *Energy Conversion and Management*, *254*, 115245. https://doi.org/https://doi.org/10.1016/j.enconman.2022.115245

Orner, K. D., & Mihelcic, J. R. (2018). A review of sanitation technologies to achieve multiple sustainable development goals that promote resource recovery. *Environmental Science: Water Research and Technology*, *4*(1), 16–32. https://doi.org/10.1039/c7ew00195a

Pamenter, S., & Myers, R. J. (2021). Decarbonizing the cementitious materials cycle: A whole-systems review of measures to decarbonize the cement supply chain in the UK and European contexts. *Journal of Industrial Ecology*, *25*(2), 359–376. https://doi.org/10.1111/jiec.13105

Paoli, L., Lupton, R. C., & Cullen, J. M. (2018). Useful energy balance for the UK: An uncertainty analysis. *Applied Energy*, *228*, 176–188. https://doi.org/10.1016/j.apenergy.2018.06.063

Pardo, N., Moya, J. A., & Vatopoulos, K. (2012). Prospective Scenarios on Energy Efficiency and CO 2 Emissions in the EU Iron & Steel Industry. *JRC Scientific and Policy Reports*. https://doi.org/10.2790/64264

Pei, M., Petäjäniemi, M., Regnell, A., & Wijk, O. (2020). Toward a Fossil Free Future with HYBRIT: Development of Iron and Steelmaking Technology in Sweden and Finland. *Metals*, *10*(7), 972. https://doi.org/10.3390/met10070972

Prussi, M., Yugo, M., De Prada, L., Padella, M., & Edwards, R. (2020). *JEC Well-To-Wheels report v5* (p. 129). The European Commission. https://doi.org/10.2760/100379

Rahimi, A., & García, J. M. (2017). Chemical recycling of waste plastics for new materials production. *Nature Reviews Chemistry*, *1*(6), 46. https://doi.org/10.1038/s41570-017-0046

Raucci, C., Smith, T., & Dodds, P. (2017). *The potential of hydrogen to fuel international shipping.*

Rinaldi, R., Lombardelli, G., Gatti, M., Visconti, C. G., & Romano, M. C. (2023). Techno-economic analysis of a biogas-to-methanol process: Study of different process configurations and conditions. *Journal of Cleaner Production*, *393*, 136259. https://doi.org/https://doi.org/10.1016/j.jclepro.2023.136259

Röck, M., Martin, R., & Hausberger, S. (2020). *JEC Tank-To-Wheels report v5: Heavy duty vehicles* (H. HANARP Per; BERSIA Carla; COLOMBANO Mauro; GRÄSER Henryk; GOMES MARQUES Gian; MIKAELSSON Helen; DE PRADA Luis; PRUSSI Matteo; LONZA Laura; YUGO Marta; HAMJE, Ed.). The European Commission; Publications Office of the European Union. https://doi.org/10.2760/541016

Rogers, J. G., Cooper, S. J., & Norman, J. B. (2018). Uses of industrial energy benchmarking with reference to the pulp and paper industries. *Renewable and Sustainable Energy Reviews*, *95*(August 2016), 23–37. https://doi.org/10.1016/j.rser.2018.06.019

Roque, B. M., Salwen, J. K., Kinley, R., & Kebreab, E. (2019). Inclusion of Asparagopsis armata in lactating dairy cows' diet reduces enteric methane emission by over 50 percent. *Journal of Cleaner Production*, *234*, 132–138. https://doi.org/10.1016/j.jclepro.2019.06.193

Rouwenhorst, K. H. R., Krzywda, P. M., Benes, N. E., Mul, G., & Lefferts, L. (2021). *Ammonia Production Technologies* (pp. 41–83). Elsevier Inc. https://doi.org/10.1016/b978-0-12-820560-0.00004-7

Sadeghi, S., Ghandehariun, S., & Rosen, M. A. (2020). Comparative economic and life cycle assessment of solar-based hydrogen production for oil and gas industries. *Energy*, *208*, 118347. https://doi.org/https://doi.org/10.1016/j.energy.2020.118347

Saygin, D., Broek, M. van den, Ramírez, A., Patel, M. K., & Worrell, E. (2013). Modelling the future CO2 abatement potentials of energy efficiency and CCS: The case of the Dutch industry. *International Journal of Greenhouse Gas Control*, *18*, 23–37. https://doi.org/10.1016/J.IJGGC.2013.05.032

Saygin, D., & Gielen, D. (2021). Zero-emission pathway for the global chemical and petrochemical sector. *Energies*, *14*(13). https://doi.org/10.3390/en14133772

Saygin, D., Patel, M. K., Tam, C., & Gielen, D. J. (2009). *IEA Information Paper: Chemical and Petrochemical Sector.* https://www.iea.org/reports/chemical-and-petrochemical-sector

Scalet, B. M., Garcia Muñoz, M., Sissa Aivi, Q., Roudier, S., & Luis, D. S. (2013). *Best Available Techniques (BAT) Reference Document for the Manufacture of Glass* (p. 485). https://doi.org/10.2791/69502

Schäfer, A. W., Barrett, S. R. H., Doyme, K., Dray, L. M., Gnadt, A. R., Self, R., O'Sullivan, A., Synodinos, A. P., & Torija, A. J. (2019). Technological, economic and environmental prospects of all-electric aircraft. *Nature Energy*,

4(2), 160–166. https://doi.org/10.1038/s41560-018-0294-x

Schlömer, S., Bruckner, T., Fulton, L., Hertwich, E., McKinnon, A., Perczyk, D., Roy, J., Schaeffer, R., Sims, R., & Smith, P. (2014). Annex III: Technology-specific cost and performance parameters. In *Climate change 2014: Mitigation of climate change: Contribution of working group III to the fifth assessment report of the intergovernmental panel on climate change* (pp. 1329–1356). Cambridge University Press. https://www.ipcc.ch/site/assets/uploads/2018/02/ipcc_wg3_ar5_annex-iii.pdf

Schmidt, P. R., Zittel, W., Weindorf, W., & Raksha, T. (2016). *Renewables in Transport 2050*. 203.

Schmitz, A., Kamiński, J., Maria Scalet, B., & Soria, A. (2011). Energy consumption and CO2 emissions of the European glass industry. *Energy Policy*, *39*(1), 142–155. https://doi.org/10.1016/j.enpol.2010.09.022

Seddiek, I. S., Elgohary, M. M., & Ammar, N. R. (2015). The hydrogen-fuelled internal combustion engines for marine applications with a case study. *Brodogradnja: Teorija i Praksa Brodogradnje i Pomorske Tehnike*, *66*(1), 23–38.

Siegemund, S., Schmidt, P., Trommler, M., & Weindorf, W. (2017). *The potential of electricity-based fuels for low-emission transport in the EU*. Ludwig-Bölkow-Systemtechnik GmbH (LBST); Deutsche Energie-Agentur GmbH (dena).

Sims, R., Taylor, M., Saddler, J., & Mabee, W. (2008). From 1st-to 2nd-generation biofuel technologies. *Paris: International Energy Agency (IEA) and Organisation for Economic Co-Operation and Development*, 16–20.

Smit, B. (2014). *Introduction to carbon capture and sequestration Berend Smit, Jeffrey A. Reimer, Curtis M. Oldenburg, Ian C. Bourg*. Imperial College P.

Smith, C., Hill, A. K., & Torrente-Murciano, L. (2020). Current and future role of Haber–Bosch ammonia in a carbon-free energy landscape. *Energy & Environmental Science*, *13*(2), 331–344. https://doi.org/10.1039/C9EE02873K

Speight, J. G. (2020). *12 - Synthesis gas and the Fischer–Tropsch process* (J. G. B. T.-. T. R. of the F. (Second. E. Speight, Ed.; pp. 427–468). Gulf Professional Publishing. https://doi.org/https://doi.org/10.1016/B978-0-12-816994-0.00012-9

SSAB, LKAB, & Vattenfall. (2017). *Summary of findings from HYBRIT Pre-Feasibility Study 2016–2017*. The Swedish Energy Agency. https://ssabwebsitecdn.azureedge.net/-/media/hybrit/files/hybrit_brochure.pdf?m=20180201085027

Staffell, I., Scamman, D., Velazquez Abad, A., Balcombe, P., Dodds, P. E., Ekins, P., Shah, N., & Ward, K. R. (2019). The role of hydrogen and fuel cells in the global energy system. *Energy Environ. Sci.*, *12*(2), 463–491. https://doi.org/10.1039/C8EE01157E

Suhr, M., Klein, G., Kourti, I., Rodrigo Gonzalo, M., Giner Santonja, G., Roudier, S., & Delgado Sancho, L. (2015). *Best Available Techniques (BAT) Reference Document for the Production of Pulp, Paper and Board*. European Commission. https://doi.org/10.2791/370629

Tassou, S. A., Kolokotroni, M., Gowreesunker, B., Stojceska, V., Azapagic, A., Fryer, P., & Bakalis, S. (2014). Energy demand and reduction opportunities in the UK food chain. *Proceedings of Institution of Civil Engineers: Energy*, *167*(3), 162–170. https://doi.org/10.1680/ENER.14.00014

Textile Exchange. (2019). *Preferred Fiber & Materials: Market Report 2019*. https://store.textileexchange.org/product/2019-preferred-fiber-materials-report/

The Royal Society. (2020). *Ammonia: zero-carbon fertiliser, fuel and energy store* (p. 40). https://royalsociety.org/-/media/policy/projects/green-ammonia/green-ammonia-policy-briefing.pdf

Uekert, T., Singh, A., DesVeaux, J. S., Ghosh, T., Bhatt, A., Yadav, G., Afzal, S., Walzberg, J., Knauer, K. M., Nicholson, S. R., Beckham, G. T., & Carpenter, A. C. (2023). Technical, Economic, and Environmental Comparison of Closed-Loop Recycling Technologies for Common Plastics. *ACS Sustainable Chemistry & Engineering*, *11*(3), 965–978. https://doi.org/10.1021/acssuschemeng.2c05497

UNEP IRP. (2021). *Global Material Flows Database*. https://www.resourcepanel.org/global-material-flows-database

Ürge-Vorsatz, D., Cabeza, L. F., Serrano, S., Barreneche, C., & Petrichenko, K. (2015). Heating and cooling energy trends and drivers in buildings. *Renewable and Sustainable Energy Reviews*, *41*, 85–98. https://doi.org/10.1016/j.rser.2014.08.039

Van Der Giesen, C., Kleijn, R., & Kramer, G. J. (2014). Energy and climate impacts of producing synthetic hydrocarbon fuels from CO2. *Environmental Science and Technology*, *48*(12), 7111–7121. https://doi.org/10.1021/es500191g

Vogl, V., Åhman, M., & Nilsson, L. J. (2018). Assessment of hydrogen direct reduction for fossil-free steelmaking. *Journal of Cleaner Production*, *203*, 736–745. https://doi.org/10.1016/j.jclepro.2018.08.279

Voldsund, M., Gardarsdottir, S. O., De Lena, E., Pérez-Calvo, J.-F., Jamali, A., Berstad, D., Fu, C., Romano, M., Roussanaly, S., Anantharaman, R., Hoppe, H., Sutter, D., Mazzotti, M., Gazzani, M., Cinti, G., & Jordal, K. (2019). Comparison of Technologies for CO 2 Capture from Cement Production-Part 1: Technical Evaluation. *Energies*, *12*(3), 559. https://doi.org/10.3390/en12030559

Westbroek, C. D., Bitting, J., Craglia, M., Azevedo, J. M. C., & Cullen, J. M. (2021). Global material flow analysis of glass: From raw materials to end of life. *Journal of Industrial Ecology*, *25*(2), 333–343. https://doi.org/10.1111/

jiec.13112

Woods, J., Williams, A., Hughes, J. K., Black, M., & Murphy, R. (2010). Energy and the food system. *Philosophical Transactions of the Royal Society B: Biological Sciences*, *365*(1554), 2991–3006. https://doi.org/10.1098/rstb.2010.0172

World Steel Association. (2019). *World Steel in Figures 2019*. https://worldsteel.org/wp-content/uploads/2019-World-Steel-in-Figures.pdf

Worrell, E., Price, L., Neelis, M., Galitsky, C., & Nan, Z. (2007). World Best Practice Energy Intensity Values for Selected Industrial Sectors. *Lawrence Berkeley National Laboratory*, *February*, 51. https://escholarship.org/uc/item/77n9d4sp

Zang, G., Sun, P., Elgowainy, A., & Wang, M. (2021). Technoeconomic and Life Cycle Analysis of Synthetic Methanol Production from Hydrogen and Industrial Byproduct CO 2. *Environmental Science & Technology*, *55*(8), 5248–5257. https://doi.org/10.1021/acs.est.0c08237

Zemo Partnership. (2022). *Zero Emission Bus Guide* (October). www.zemo.org.uk/assets/reports/ZEMO_ZERO_EMISSION_BUS_GUIDE_2022_ONLINE_VERSION.pdf

Zheng, J., & Suh, S. (2019). Strategies to reduce the global carbon footprint of plastics. *Nature Climate Change*, *9*(5), 374–378. https://doi.org/10.1038/s41558-019-0459-z

Zühlsdorf, B., Bühler, F., Bantle, M., & Elmegaard, B. (2019). Analysis of technologies and potentials for heat pump-based process heat supply above 150 °C. *Energy Conversion and Management: X*, *2*, 100011. https://doi.org/10.1016/J.ECMX.2019.100011
